# Cation Sampling Enables Regiodivergent Distal Functionalization of Ketones

**DOI:** 10.1021/jacs.6c05299

**Published:** 2026-05-18

**Authors:** Philipp Spieß, Miloš Vavrík, Jakob Frey, Uroš Vezonik, Daniel Kaiser, Nuno Maulide

**Affiliations:** Institute of Organic Chemistry, University of Vienna, Währinger Straße 38, 1090 Vienna, Austria

## Abstract

Despite notable progress in catalytic olefin isomerization processes enabling distal Csp^3^–H functionalization, most methods depend on complex metal-based catalytic systems and face inherent challenges in achieving regiodivergent outcomes. Herein, we present a conceptually distinct approach in which we leverage cationic olefin isomerization for regiodivergent Csp^3^–H functionalization, wherein the selectivity results from a ketone-assisted process we term *cation sampling*. This method enables site-selective regiodivergent functionalization of sp^3^-hybridized carbons, allowing the formation of C–heteroatom bonds in challenging positions and unlocking access to products previously unattainable through metal-based methodologies. Detailed mechanistic studies support the proposed reaction pathway, which involves the engagement of two key oxocarbenium species that govern the regioselectivity of this process.

## Graphical abstract

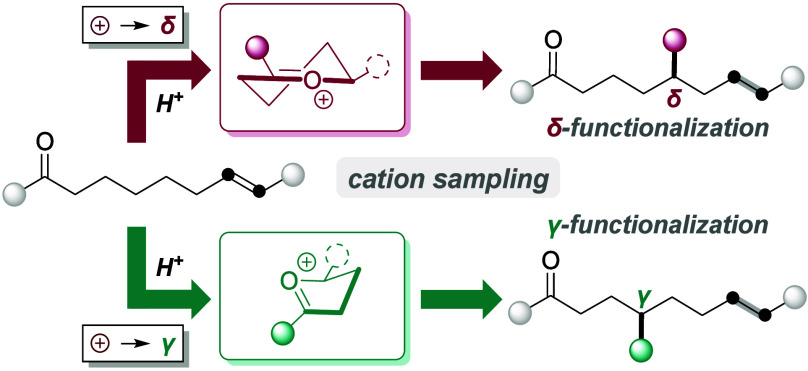

Organic chemistry has historically relied on functional groups as reactive handles to enable selective transformations of neighboring Csp^3^–H bonds through polarization. In contrast, the targeted activation of distant, non-neighboring sites represents a much greater challenge, primarily due to the absence of strong polarization effects (Scheme 1A). This is readily apparent in the case of ketones and carbonyl derivatives, where α- (and to some extent β-) functionalization are readily achieved (e.g., through enolate or enolonium intermediates for α-functionalization, or conjugate addition to a, separately prepared, α,β-unsaturated carbonyl for β-functionalization),^1–3^ whereas the direct functionalization of distal positions, such as γ and δ, remains beyond the immediate reach of classical approaches.^4^ The relatively similar dissociation energies of unbiased Csp^3^–H bonds, coupled with the high number of Csp^3^–H sites within a molecule—often nearly indistinguishable in their chemical properties—pose major hurdles to remote functionalization,^5,6^ with only relatively few mechanistically distinct approaches offering specific solutions.^7–11^

**Scheme 1:**
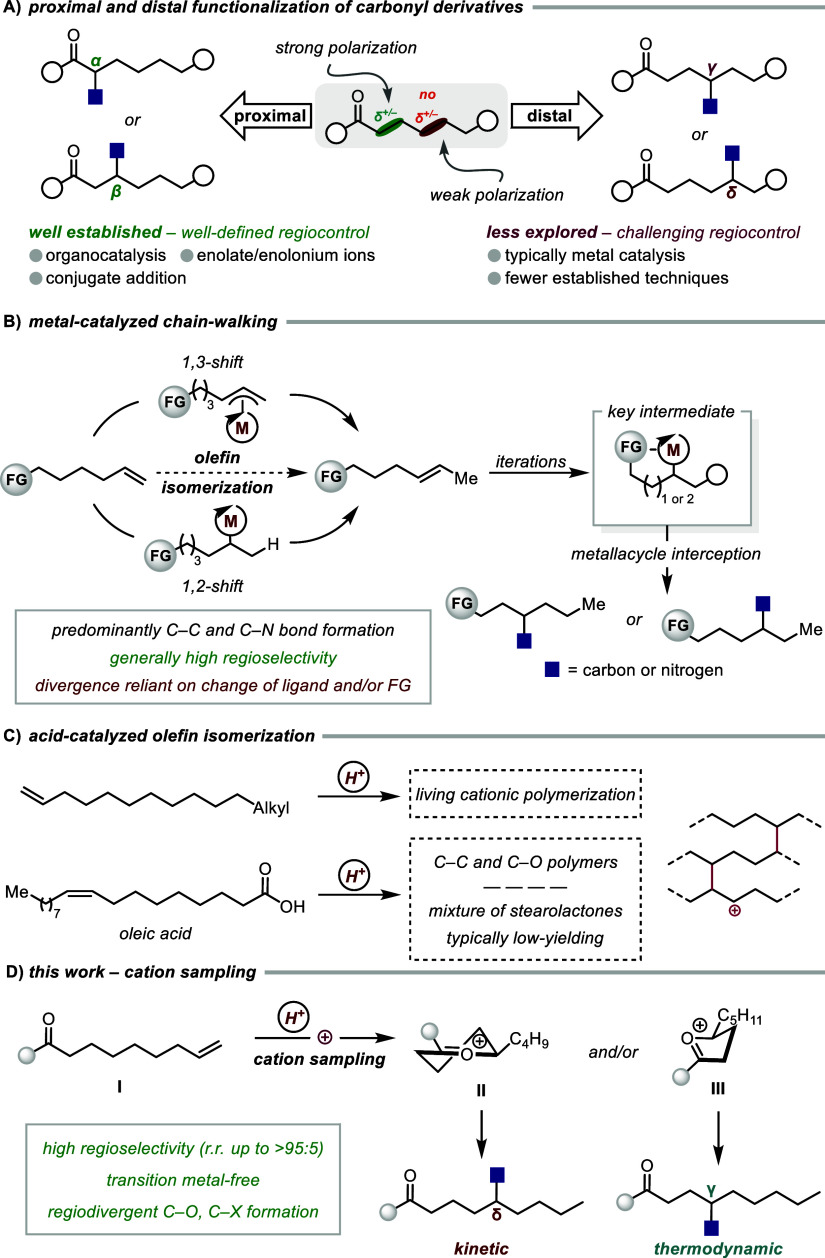
Contemporary Metal-Based Remote Csp^3^-Functionalizations and Our Approach Relying on Acid-Catalyzed Olefin Isomerization

An elegant approach to achieve such functionalizations involves leveraging alkene reactivity through transition metal-mediated double-bond isomerization, in which the metal catalyst (e.g., Ni, Pd) undergoes dynamic displacement along the alkyl chain (Scheme 1B), enabling primarily the formation of C–C or C–N bonds.^12–14^ The regioselectivity of these transformations is dictated by the formation of metastable metallacyclic intermediates, wherein the metal center engages in specific interactions with a directing group—most commonly an amide—to determine the site of functionalization.^15^ However, regiodivergent, site-selective functionalization remains challenging, especially when no alterations to the directing group are desired.^16–22^

We were intrigued by the possibility of employing Brønsted acid-catalyzed olefin isomerization, a process first described over a century ago,^23,24^ to achieve distal Csp^3^-functionalization. The development of a high-yielding and selective acid-mediated reaction would be valuable, particularly if regiodivergence could be achieved. However, competing oligo- and polymerization,Scheme 1C which dominate previous reports,^25–27^ have confined such processes to few synthetically useful examples, such as the isomerization of simple alkenes and alkene arylation.^23,28,29^

Although the acid-mediated lactonization of fatty acids^30,31^ has been studied for a range of substrates,^32^ careful analysis of the literature reveals that a tandem process consisting of both olefin isomerization and δ-lactonization has only been reported in a single instance. This limited example, using oleic acid,^33,34^ is poor yielding and highlights the dual challenges of suppressing polymerization and simultaneously controlling regioselectivity.

Owing to the inherently reversible nature of oxocarbenium ion formation, we hypothesized that ketone-substitution might enable the suppression of undesired polymerization pathways.^35,36^ We speculated that the presence of a mildly nucleophilic moiety (a ketone) might prevent C–C polymerization by stabilizing cationic intermediates through transient capture, while—in contrast to the more nucleophilic carboxylates seen in, e.g., fatty acids—also ensuring greater reversibility of deleterious intermolecular capture.^25^

Crucially, for the development of a regioselective method, protonation of an olefin (e.g., **I**; Scheme 1D) is known to generate an interconverting mixture of regioisomeric carbocations and alkenes.^37^ We anticipated that such a pool of cationic and neutral species would be resolved through covalent capture by a ketone moiety, enabling effective *sampling* of the cationic intermediates to give a single reactive species primed for further functionalization.

Initial trials, in which alkene **I** was treated with triflic acid at elevated temperature, revealed the formation of a species which was identified as the 6-membered oxocarbenium ion **II**. The concomitant detection of a minor amount of the 5-membered oxocarbenium ion **III** suggested the possibility of developing a divergent method in which distinct products could be obtained.

Herein, we detail our findings on the exploration of this possibility and present a strategy we refer to as *cation sampling*, which provides a versatile and regiodivergent approach to the distal Csp^3^–H functionalization of ketones. We further detail the discovery that this protocol enables the possibility to operate under kinetic (**II**) or thermodynamic (**III**) control, enabling the synthesis of a diverse array of C–heteroatom-substituted functionalized products.

With initial results demonstrating the possibility of forming different oxocarbenium species (**II** and **III**), we engaged in extensive optimization (see Supporting Tables S1 and S2 for details) and ultimately devised two sets of reaction conditions which provided means for the selective formation—and further transformation—of either regioisomeric oxocarbenium species (**II** or **III**). Notably, both sets of conditions allow the reaction to be performed under an atmosphere of air and differ only in the solvent and temperature.

Initial exploration of the scope of these reactions focused on the formation of ketones bearing hydroxy groups, as can be achieved by hydrolysis of the respective oxocarbenium intermediates. We first sought to showcase δ-functionalization on a range of substrates with varying chain lengths and bearing terminal olefins (Scheme 2). Pleasingly, the corresponding products **1a**–**1d** were generally formed in high yields and regioisomeric ratios.^38^ We further found internal alkene substrates to be readily transformed into the corresponding δ-hydroxy ketones (**1b**, **1e**–**1h**) in high yields and with consistently good regioselectivities. The structure of **1h**, derived from oleic acid, was unambiguously confirmed by X-ray crystallography. Additionally, product **1i**, bearing an α-substituent, was obtained in moderate yield and diastereoselectivity, but—despite the increased favorability of the double bond moving into conjugation—as a single observable regioisomer. Varying substitution on the ketone was also found to be well tolerated, with electron-donating and -depleting substituents on an aromatic, as well as heterocyclic substrates allowing the formation of products **1j**–**1w**. Interestingly, we found that the reaction outcome was only mildly influenced by the electronics of the ketones, observing that electron-deficient ketones allowed for excellent δ-selectivity at slightly lower reaction temperatures compared to electron-neutral and electron-rich congeners. Notably, a substrate containing an unprotected phenol (**1p**)—a functionality previously found to be capricious in migratory functionalization reactions involving transition metals^17^—was also well-tolerated. In addition, primary, secondary, and tertiary alkyl ketones were also viable substrates (yielding **1x**–**1aa**), and structures derived from drugs such as oxaprozin, probenecid, and julolidine demonstrated tolerance of more complex functionalities (**1ab**–**1ad**).

**Scheme 2:**
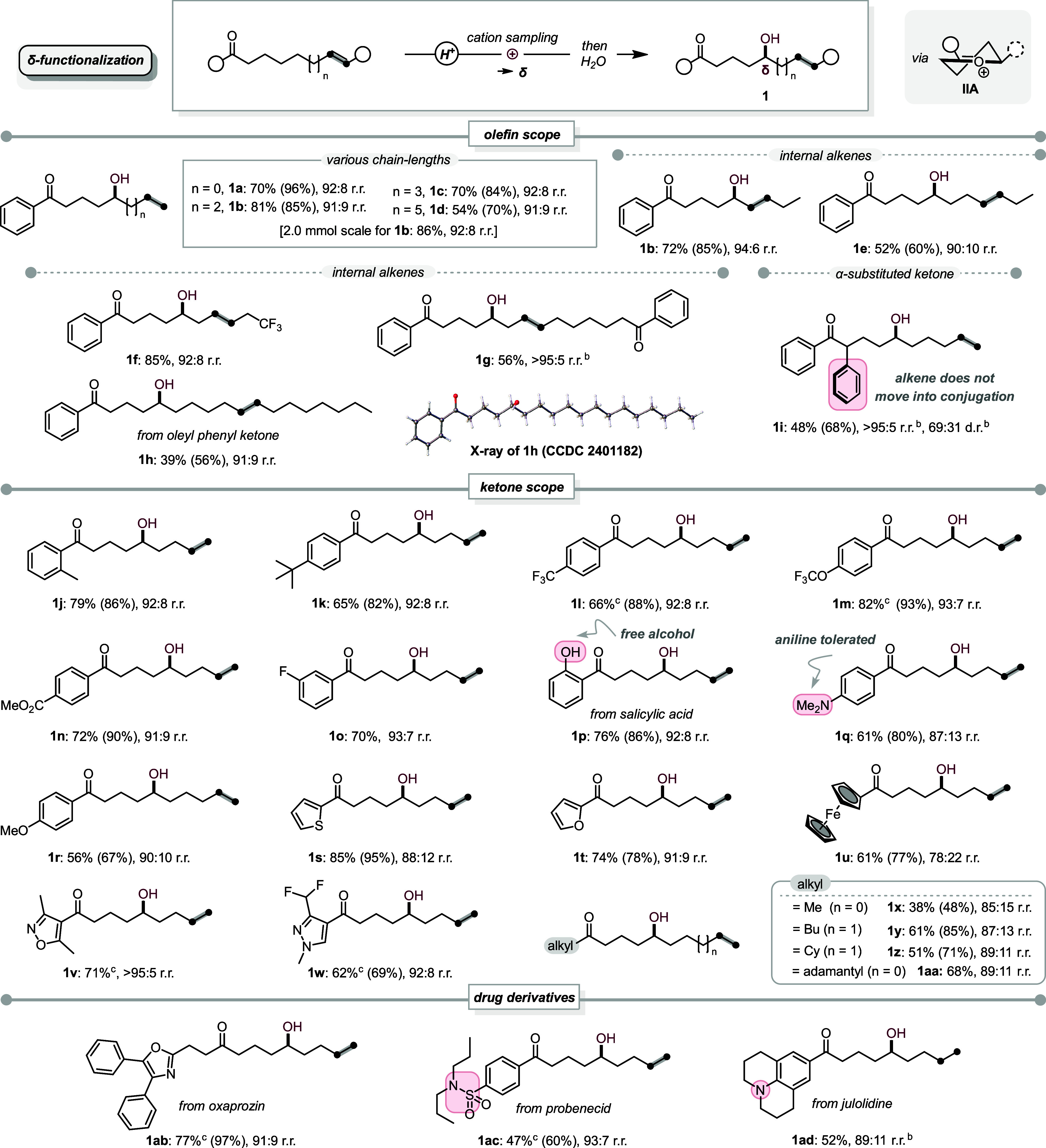
Scope of δ-Hydroxy Ketones^a^

a All yields correspond to isolated material; yields in parentheses correspond to NMR yields determined from the crude material, using mesitylene as an internal standard. The regioisomeric ratio was determined from the crude material and refers to the ratio of **1** to other regioisomers. Conditions: alkenyl ketone (0.1 mmol), TfOH (0.3 mmol), DCM (0.1 M), 65 °C, 1 h, then addition of HFIP (see the Supporting Information (section 4.2) for details on the role of HFIP), followed by an aqueous solution of NaHCO_3_. See the Supporting Information (section 2.2.6) for further details. ^b^Ratio after isolation. ^c^Conducted at a decreased temperature. See the Supporting Information (sections 2.2.6 and 2.4) for further details.

An equally broad range of substrates was amenable to the conditions developed for γ selectivity (Scheme 3), once again encompassing various alkyl chain variations (**2a**–**2h**) and a wide array of ketones, including heterocycles and drug derivatives (**2i**–**2z**). While yields were slightly lower compared to the δ-functionalized congeners, regioselectivity was excellent in almost all cases.

**Scheme 3:**
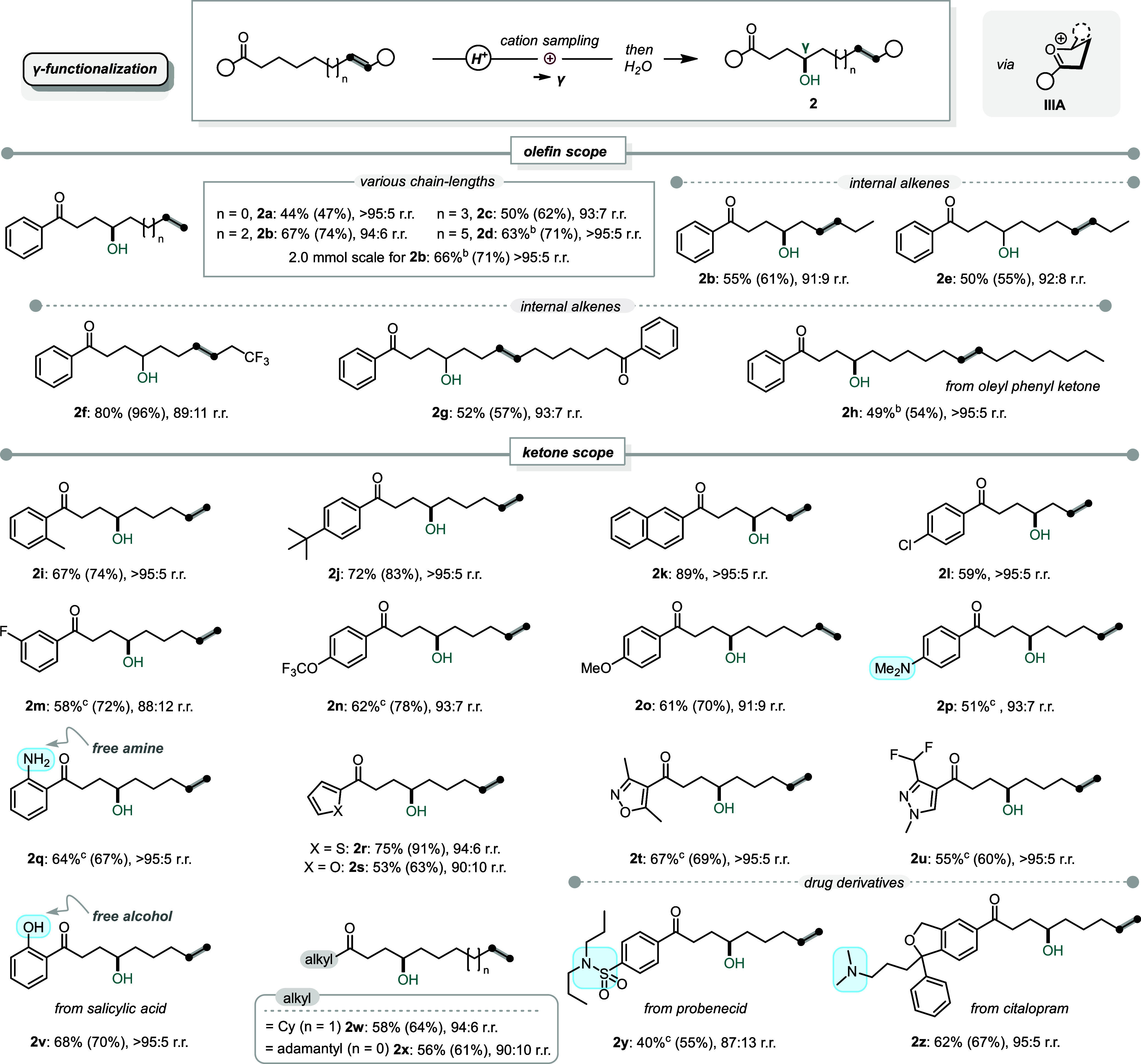
Scope of γ-Hydroxy Ketones^a^

a The regioisomeric ratio was determined from the crude material and refers to the ratio of the depicted products to other regioisomers. All yields correspond to isolated material; yields in parentheses correspond to NMR yields determined from the crude material, using mesitylene as an internal standard. Conditions: alkenyl ketone (0.1 mmol), TfOH (0.3 mmol), DCE, 120 °C, 3 h, then addition of HFIP followed by an aqueous solution of NaHCO_3_. See the Supporting Information (section 2.2.7) for further details. ^b^[HNEt_3_]­[OTf] was used as an additive. See the Supporting Information (section 2.2.8) for further details. ^c^Conducted at a decreased reaction temperature. See the Supporting Information (sections 2.2.7, 2.5 and 2.8) for further details.

Building on these promising results, we explored whether both (γ and δ) oxocarbenium species could be leveraged for diversification through alternative interception steps (Scheme 4).^35^ Indeed, by using different halide sources, a broad spectrum of alkyl halides could be accessed, yielding both γ- and δ-halogenated ketones (Scheme 4, **3**–**8**). Reductive quenching with a Hantzsch ester provided *cis*-disubstituted cyclic ethers (THP **9** and THF **10**) in good yields with excellent diastereoselectivities.^39^ Furthermore, directly subjecting ketone intermediate **1b** to hydrogenation conditions led to reduction of the carbonyl group, forming aliphatic alcohol **11** in 60% yield over two steps.^40^ Notably, this amounts to rendering the ketone a removable directing group.^41^ Similarly, Grignard reagents and allyl silane efficiently trapped the oxocarbenium intermediates, yielding trisubstituted tetrahydrofurans and tetrahydropyrans (**12**–**14**).

**Scheme 4:**
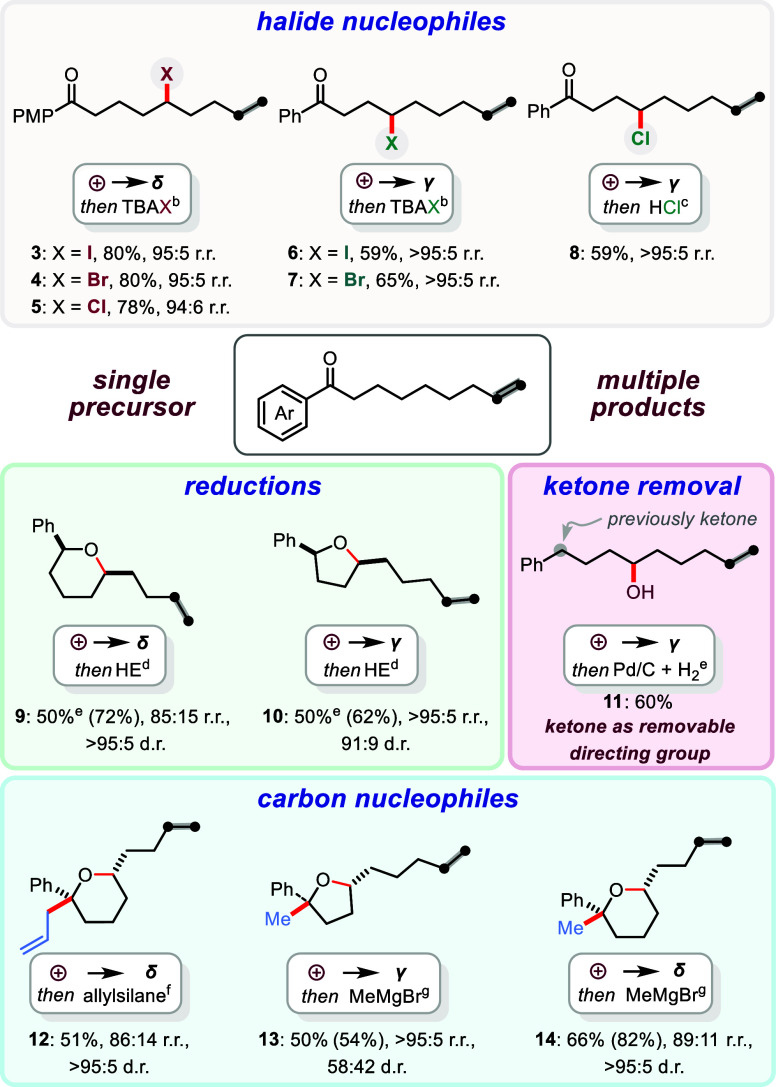
Other Oxocarbenium-Ion Functionalization Reactions^a^

a Specific information for these reactions can be found in the Supporting Information, as detailed in the following captions: ^b^Quench with corresponding tetrabutylammonium halide salts. See the Supporting Information (sections 2.2.9 and 3.1) for further details. ^c^Quench with HCl in Et_2_O. See the Supporting Information (sections 2.2.9 and 3.1) for further details. ^d^Addition of Hantzsch ester. See the Supporting Information (section 3.2) for further details. ^e^Hydrogenation with Pd/C. See the Supporting Information (section 3.3) for further details. ^f^Addition of allyl silane. See the Supporting Information (section 3.4) for further details. ^g^Addition of Grignard reagent. See the Supporting Information (section 3.5) for further details.

To probe the mechanism of cation sampling, we first examined the temperature-dependent interconversion between six- and five-membered oxocarbenium ions (Scheme 5A).

**Scheme 5:**
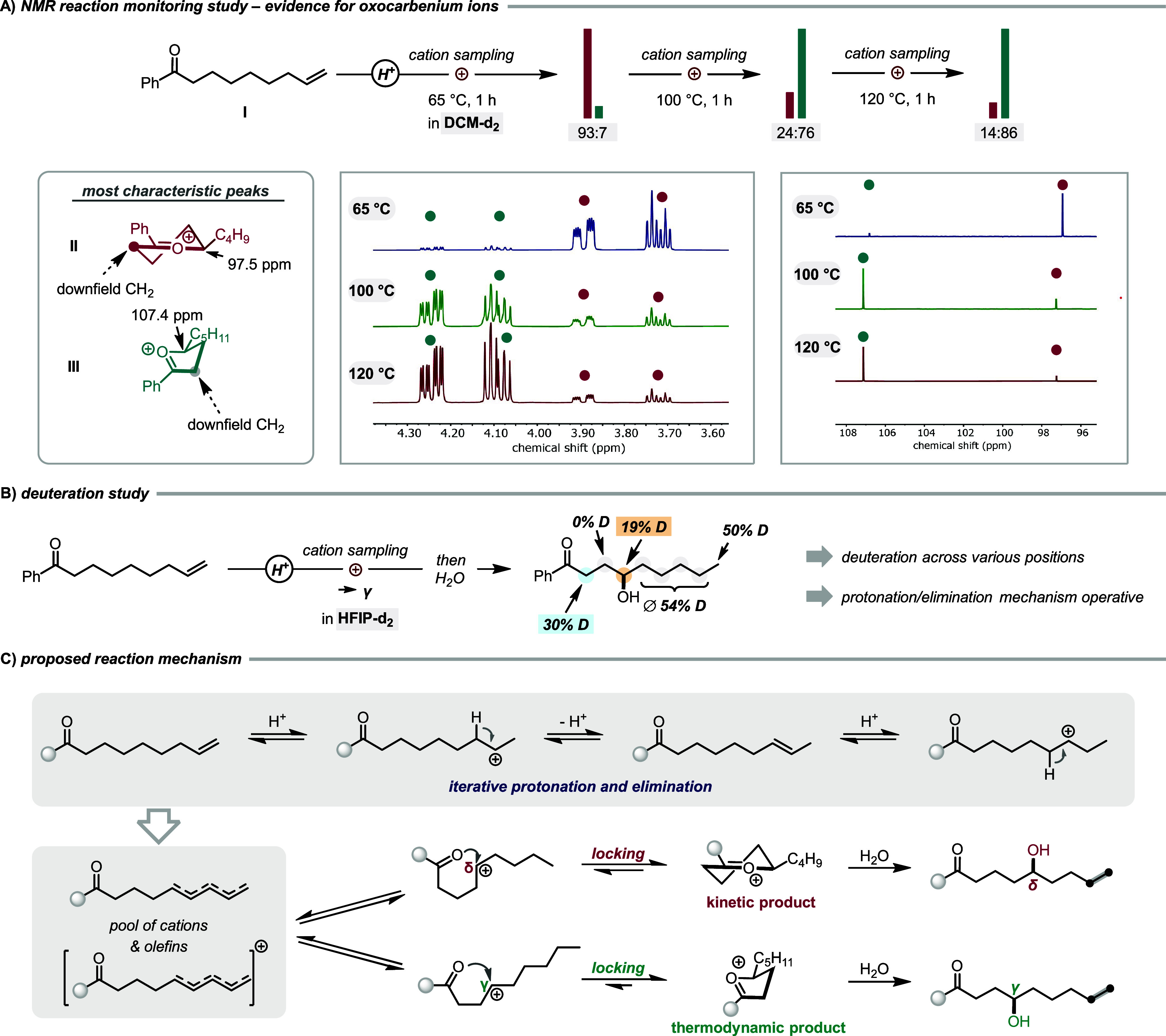
Mechanistic Studies on Cation Sampling

After 1 h at 65 °C, two distinct species were identified as the six-membered (**II**) and five-membered (**III**) oxocarbenium ions. Both species exhibited strongly downfield-shifted ^1^H and ^13^C NMR signals, consistent with their cationic character. For quantitative analysis, we monitored the α-CH_2_ group of the oxocarbenium ion and the carbon atom bridged by the ketone oxygen, as these resonances were well resolved and free of overlap (see the Supporting Information for further details). Upon increasing the temperature, the ratio **II**/**III** shifted from 93:7 at 65 °C to 24:76 at 100 °C and ultimately to 14:86 at 120 °C (1 hequilibration time each).^42^

Aiming to support our proposal of isomerization through an interconverting mixture of carbocations and olefins, we performed the reaction in the presence of an excess of D^+^. Again, oxocarbenium ions were observed as the dominant intermediates, albeit with significant deuterium incorporation along the alkyl chain (see the Supporting Information for further details). To better characterize this incorporation pattern, the reaction was repeated with an aqueous quench, enabling isolation of the corresponding deuterated γ-hydroxy ketone (Scheme 5B). Analysis revealed ≥ 50% deuterium incorporation at the first five carbon atoms of the alkyl chain. The γ-position exhibited 19% deuterium incorporation, while the α-position showed 30% incorporation.

Based on these findings, we propose the following mechanistic picture (Scheme 5C): initial protonation of the alkene at the terminal position is followed by elimination to generate a first internal alkene. Repeated protonation/elimination events subsequently generate a dynamic pool of cations (and olefins) that progressively funnels toward the δ- and γ-positions. Covalent capture by the ketone—which, given suitable distance, can outcompete elimination processes—then resolves these equilibria through formation of discrete oxocarbenium species. The temperature-dependent shift from the six-membered to the five-membered oxocarbenium ion hints at a reversible equilibration between both intermediates, with the five-membered species representing the thermodynamically favored form. The reduced deuterium incorporation at the γ-position relative to other sites suggests diminished reversibility at this stage, consistent with terminal trapping of the cation to yield an oxocarbenium intermediate. The deuterium incorporation in the α-position supports reversible enolization of the ketone or the oxocarbenium ions.

In conclusion, we have developed a reaction platform for remote Csp^3^–H functionalization, rooted in the concept of *ketone-guided cation sampling*. This approach stands out for its precise, on-demand regioselectivity (targeting either the δ- or γ-position) and its compatibility with a broad range of substrates. The versatility of oxocarbenium-ion interception was further demonstrated, allowing the synthesis of both γ- and δ-halogenated ketones as well as tetrahydopyrans and -furans. Particularly relevant is the possibility of “deleting” the carbonyl moiety, rendering it a *de facto* removable directing group. We believe that cation sampling should offer interesting opportunities in synthesis; its exploration in other contexts is underway and will be reported in due course.

## Supplementary Material



## Notes

All experimental procedures and analytical data supporting the findings of this study (PDF)
