# Supplementary material for: Cation Sampling Enables Regiodivergent Distal Functionalization of Ketones
Source: J Am Chem Soc. 2026 May 18;148(21):21230–7. doi: 10.1021/jacs.6c05299 (PMC13244450; doi:10.1021/jacs.6c05299)
Supplement: Supplementary file 1 [file ja6c05299_si_001.pdf]

Supporting Information

Cation sampling enables regiodivergent distal functionalization of ketones

Philipp Spieß‡, Miloš Vavrík‡, Jakob Frey, Uroš Vezonik, Daniel Kaiser, Nuno Maulide*

Institute of Organic Chemistry, University of Vienna, Währinger Straße 38, 1090 Vienna, Austria

‡These authors contributed equally.

Table of Contents

1. General Information 3

2. Experimental 4

2.1 Optimization 4

2.1.1 Screening of conditions for δ-products 4

2.1.2 Screening of conditions for γ-products 5

2.1.3 Discussion on discrepancies between NMR and isolated yields 6

2.1.4 Note on the importance of a correct heating setup 7

2.1.5 Note on the safety of handling TfOH 8

2.2 General procedures 9

2.2.1 General Procedure A: Synthesis of Weinreb amides from acyl chlorides 9

2.2.2 General Procedure B: Synthesis of Weinreb amides from carboxylic acids 9

2.2.3 General Procedure C: Addition of Grignard reagents to Weinreb amides 10

2.2.4 General Procedure D: Grignard addition to aldehydes 10

2.2.5 General Procedure E: Grignard addition to nitriles 11

2.2.6 General procedure F: Synthesis of δ-hydroxy ketones 12

2.2.7 General procedure G: Synthesis of γ-hydroxy ketones 13

2.2.8 General procedure H: Synthesis of γ-hydroxy ketones with triethylammonium triflate additive 14

2.2.9 General procedure I: Interception with a halide 15

2.3 Preparation of starting materials 16

2.3.1 Weinreb amides 16

2.3.2 Alkenyl ketones 24

2.4 Characterization of δ-alcohols 105

2.5 Characterization of γ-alcohols 172

2.6 Unsuccessful examples 228

2.7 Large-scale synthesis of δ-alcohol 1b 229

2.8 Large-scale synthesis of γ-alcohol 2b 230

3. Alternative Interceptions of oxocarbenium ion 231

3.1 With halides 231

3.2 With reductants 243

3.3 Reductive removal of ketone 247

3.4 Allyl silane addition 249

3.5 Grignard reagent addition 251

4. Mechanistic Studies 255

4.1 NMR study of cation sampling 255

4.2 Studies on the influence of HFIP 259

4.2.1 Explanation of the background 259

4.2.2 NMR study in HFIP-d_2 – evidence of oxocarbenium species in HFIP 259

4.2.3 NMR study with HFIP as additive 261

4.3 Deuteration Study 263

4.4 Alternative groups for cation sampling 265

5. X-ray data 267

6. References 272

1. General Information

Unless otherwise stated, all glassware was flame-dried before use and all reactions were performed under an atmosphere of argon. The given reaction temperature refers to the temperature of the surrounding (cooling/heating) medium, and not to an internally measured temperature. Anhydrous solvents and reagents were used as received from commercial suppliers unless otherwise stated. Reaction progress was monitored by thin layer chromatography (TLC) performed on aluminium plates coated with silica gel F254 with 0.2 mm thickness. Chromatograms were visualized by fluorescence quenching with UV light at 254 nm or by staining using potassium permanganate or phosphomolybdic acid, followed by heating. Flash column chromatography was performed using silica gel (15 – 40 μm), unless otherwise stated. Neat infrared spectra were recorded using a Perkin-Elmer Spectrum 100 FT-IR spectrometer. Wavenumbers (ν_{max}) are reported in cm^{-1}. Mass spectra were obtained using a Bruker maXis UHR-TOF (QQ-TOF) spectrometer, using electrospray ionization (ESI) or an Agilent 7200B GC/Q-TOF spectrometer, using electron ionization (EI). All ^{1}H NMR and ^{13}C NMR spectra were recorded using Bruker AV-400, AV-600 or AV-700 spectrometers at 298 K, unless stated otherwise. Chemical shifts (δ) are given in parts per million (ppm) and referenced to the given solvent peak as described in literature.[1] Coupling constants (*J*) are reported in Hz and the splitting patterns are designated as singlet (s), doublet (d), triplet (t) and quartet (q) as they appeared in the spectrum. If the appearance of a signal differs from the expected splitting pattern, the observed pattern is designated as apparent (app). Splitting patterns that could not be interpreted or easily visualized are designated as multiplet (m) or broad (br). DEPTQ ^{13}C NMR spectra were recorded using *jmod* or *deptqgpsp* pulse sequences and are phased such that CH_2/C signals are negative, and CH/CH_3 signals are positive. Unless otherwise stated, the compounds were isolated as racemic mixtures and the stereochemistry drawn illustrates only their relative configuration. TfOH and triflic acid are abbreviations for trifluoromethanesulfonic acid.

2. Experimental

2.1 Optimization

General procedure for the optimization:

A small vial (4 mL max. volume) was charged with 1-phenylnon-8-en-1-one (21.6 mg, 0.10 mmol, 1.00 equiv.) and the corresponding solvent (0.1 M) at 23 °C under air. TfOH was added using a microsyringe at 23 °C, the vial was immediately closed with a screwcap and the mixture was stirred at specified temperature for given time. Upon completion, the vial was placed into an ice bath for 5 min and a saturated aqueous $NaHCO_3$ solution (2 mL) was added. The mixture was poured into a separatory funnel (washing with DCM) containing more saturated aqueous $NaHCO_3$ solution (total 3 mL), the two phases were separated, and the aqueous phase was extracted with DCM (2 × 3 mL). The combined organic layers were dried over $MgSO_4$, the dried solution was filtered, and the filtrate was concentrated under reduced pressure. The NMR yield was determined by analysis of the ^{1}H NMR spectrum of the crude reaction product (DCM-d_2, 1.00 equiv. mesitylene or CH_2Br_2 as internal standard).

2.1.1 Screening of conditions for δ-products

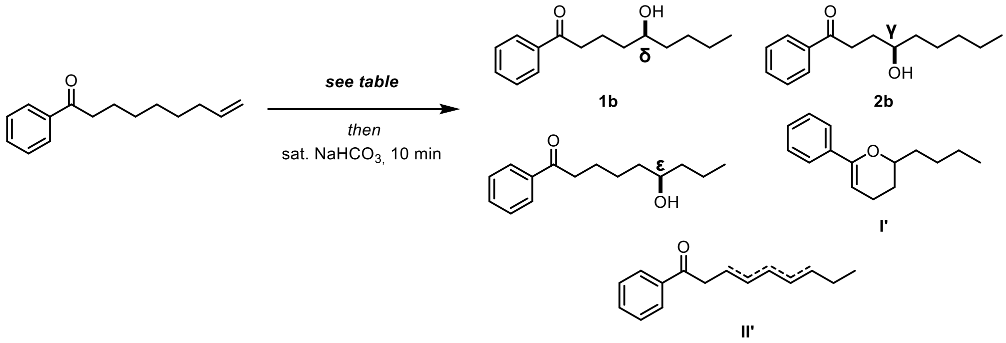

	entry	acid	solvent	T	t	total yield[a]	γ	δ	ε	I'	II'
different reaction time, reaction temperature	1	TfOH (1.2 equiv.)	HFIP	65 °C	1 h	95%	0	55	45		
	2	TfOH (1.2 equiv.)	HFIP	65 °C	16 h	20%	33	67	0	64%	
	3	TfOH (1.2 equiv.)	HFIP	23 °C	80 h	75%	0	50	50		
different acid amount and solvent	4	TfOH (3.0 equiv.)	HFIP	65 °C	1 h	(73%)	0	80	20		
	5	TfOH (3.0 equiv.)	DCM	65 °C	3 h	15%	indistinguishable			74%	
	6	TfOH (1.2 equiv.)	DCM	65 °C	1 h	n.d.	-	-	-	60%	36%
	7	TfOH (3.0 equiv.)	$MeNO_2$	65 °C	3 h	74%	36	56	8	3%	
HFIP as additive for quench	8	TfOH (3.0 equiv.)	DCM[b]	65 °C	1 h	85% (81%)	9	91	-		

Table S1. Optimization for the synthesis of δ-hydroxy ketone 1b. [a]Yields were determined by ^{1}H NMR analysis of the crude reaction products using mesitylene or CH_2Br_2 as internal standard. [b]Addition of 0.5 mL HFIP before aqueous quenching. Further information on the influence of HFIP on the aqueous workup can be found in Section 4.2. Yields in brackets indicate isolated yields.

2.1.2 Screening of conditions for γ-products

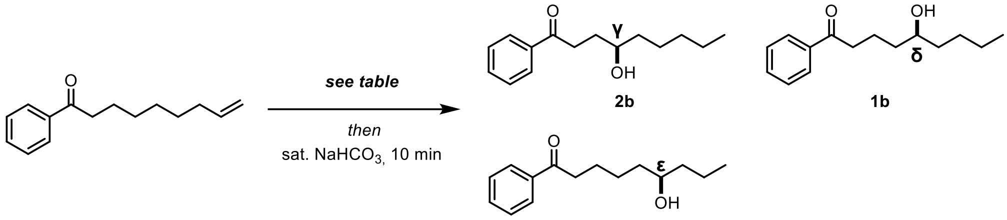

	entry	acid	solvent	*T*	*t*	total yield[a]	γ	δ	ε
different reaction time, reaction temperature	*1*	TfOH (3.0 equiv.)	HFIP	80 °C	16 h	88%	35	65	0
	2	TfOH (3.0 equiv.)	HFIP	100 °C	3 h	85%	74	26	0
	3	TfOH (3.0 equiv.)	HFIP	110 °C	3 h	80%	91	9	0
	4	TfOH (3.0 equiv.)	HFIP	120 °C	3 h	75%	95	5	0
different acid amount and solvent	*5*	TfOH (10.0 equiv.)	HFIP	100 °C	16 h	50%	100	0	0
	6	TfOH (3.0 equiv.)	TFE	120 °C	3 h	55%	95	5	0
	7	TfOH (3.0 equiv.)	DCE[b]	120 °C	3 h	73% (66%)	95	5	0

Table S2. Optimization for the synthesis of γ-hydroxy ketone 2b. [a]Yields were determined by ^{1}H NMR analysis of the crude reaction products using mesitylene or CH_2Br_2 as internal standard. [b]Addition of 0.5 mL of HFIP before aqueous quenching. Further information on the influence of HFIP on the aqueous workup can be found in Section 4.2. Yields in brackets indicate isolated yields.

2.1.3 Discussion on discrepancies between NMR and isolated yields

During our studies, we frequently observed significant (>10%) discrepancies between NMR and isolated yields (see Figure S1). We sometimes observed the formation of hemiacetals or dihydropyrans/dihydrofurans (DHP/DHF) in our optimization studies (see Sections 2.1.1, 2.1.2, 4.2), therefore we hypothesized that such discrepancies might be arising from hemiacetal or DHP/DHF formation during purification. This problem was largely circumvented by using NEt_3 as an additive (1%, to neutralize acidic SiO_2) in the eluent mixture and, most importantly, by refraining from using $CDCl_3$ as the NMR solvent for crude products—since it often contains traces of acid—and instead employing CD_2Cl_2. With these two precautions, the yield discrepancies dropped to less than 10% in most cases.

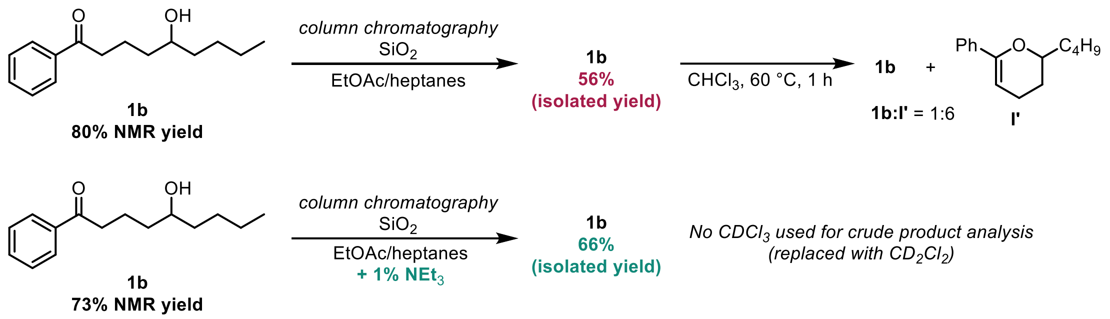

Figure S1. Product decomposition during purification and storage in $CDCl_3$. The δ-hydroxy ketone was prepared according to General Procedure F.

Thus, we recommend using both precautions (CD_2Cl_2 for analysis of crude products and 1% triethylamine additive in column chromatography) to prevent yield loss during purification. In general, we advise to always measure NMR yields of crude products, to help with troubleshooting in case the reaction does not work as expected.

2.1.4 Note on the importance of a correct heating setup

Since the regiochemical outcome of this reaction is dictated by the reaction temperature, a correct method of heating the reaction vessel is essential. Both (δ and γ) products are prepared at temperatures exceeding the solvent boiling points (65 °C for δ products in dichloromethane and 120 °C for γ products in 1,2-dichloroethane). To prevent issues with low r.r. values, we recommend:

a) using airtight screwcap vials or thick-walled Schlenk flasks, which can withstand the overpressure and corrosive properties of TfOH (no grease or septa).

b) using an oil bath or a metal heating block and immersing the vessels to at least 40% of total volume (see pictures below). We observed that the use of sand baths or insufficiently immersed vessels gave lower r.r. values than expected. We believe that this occurs due to insufficient heat flux into the reaction medium, which fails to offset the cooling effect of the surrounding air.

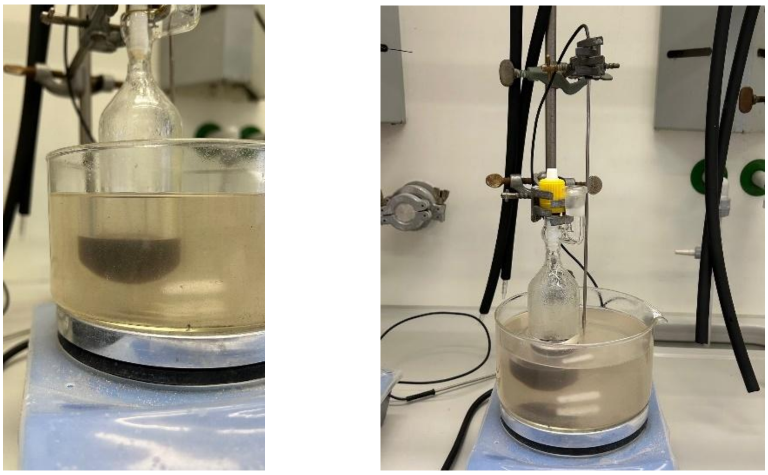

Figure S2. Correct heating setup for large-scale reactions. The Schlenk flask should be sufficiently immersed in an oil bath to ensure efficient heat transfer. Insufficient immersion may lead to heat loss to the surrounding air, resulting in lower internal temperatures and consequently reduced r.r.

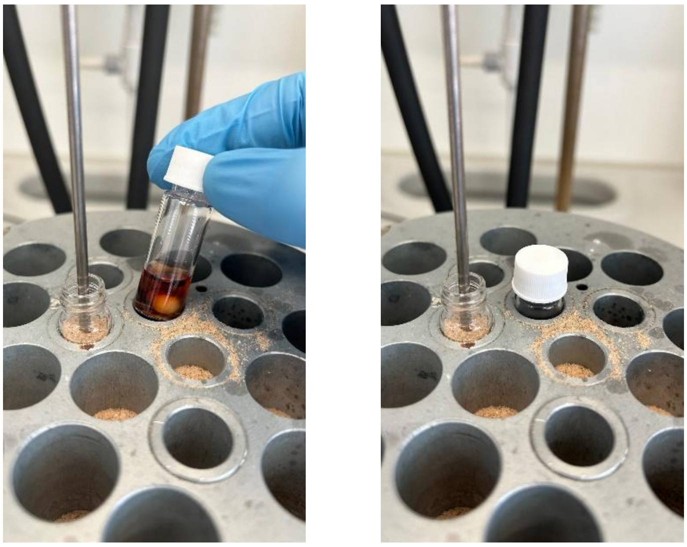

Figure S3. Correct heating setup for small-scale reactions. The vial should be sufficiently immersed in a heating medium (metal heating block or oil bath) to ensure efficient heat transfer. Insufficient immersion may lead to heat loss to the surrounding air, resulting in lower internal temperatures and consequently reduced r.r.

2.1.5 Note on the safety of handling TfOH

TfOH remains stable when stored in airtight, sealed glass containers, as provided by commercial suppliers. However, **it exhibits extreme corrosivity towards certain plastics, including syringes and needle adapters**.

For small-scale applications, TfOH can be safely transferred using glass microsyringes (25 – 100 µL capacity) with metal or Teflon plungers. We have not observed issues at this scale, though it is crucial to immediately rinse the syringe with water in a well-ventilated fume hood to prevent corrosion of the metal plungers.

For larger-scale operations, **glass syringes with glass or Teflon plungers, and metal-only needles must be used, and transferring should be strictly limited to a well-ventilated fume hood!** Using TfOH, standard syringes are susceptible to leaching, and occasionally cracking. Needles present additional problems, as the plastic components used for needle attachment to the syringe can melt upon contact with TfOH.

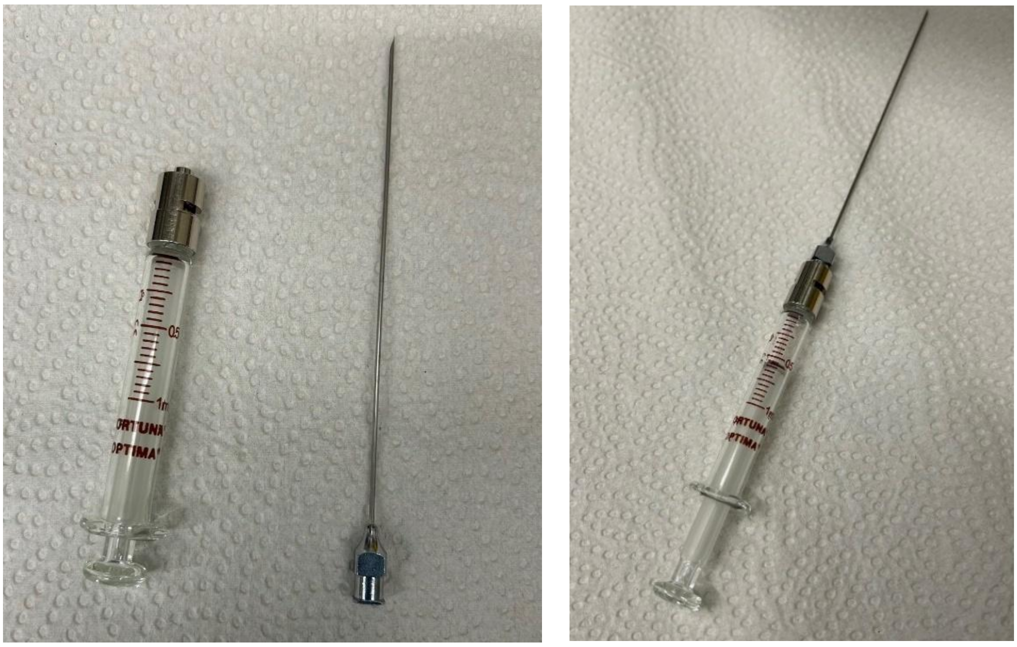

Figure S4. Correct syringe + needle combination for transferring larger quantities (>100 µL) of TfOH.

2.2 General procedures

2.2.1 General Procedure A: Synthesis of Weinreb amides from acyl chlorides

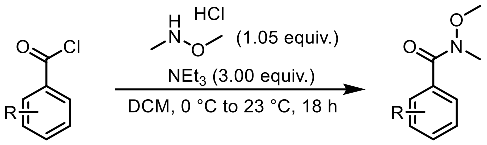

In a 50 mL Schlenk flask *N*,*O*-dimethylhydroxylamine hydrochloride (1.05 equiv.) was dissolved in DCM (0.3 M) at 23 °C. Triethylamine (3.00 equiv.) was added in one portion and the resulting suspension was cooled to 0 °C. The corresponding acyl chloride (1.00 equiv.) was added and the solution was stirred for 18 h with slow warming to 23 °C (the flask was left in ice bath which melted overnight). After this time, saturated aqueous solution of $NaHCO_3$ was added (3 mL/mmol) and the mixture was transferred into a separatory funnel. The organic phase was separated and the aqueous phase was washed with DCM (3 × 2 mL/mmol). The combined organic layers were washed with aqueous 1 M HCl solution (3 mL/mmol), followed by brine (3 mL/mmol) and then dried over anhydrous sodium sulfate. The dried solution was filtered and the filtrate was concentrated under reduced pressure. The crude residue was usually used in the next step without further purification. In cases where significant amounts of impurities were observed, the crude product was purified using flash column chromatography (ethyl acetate in heptanes, see individual products below).

2.2.2 General Procedure B: Synthesis of Weinreb amides from carboxylic acids

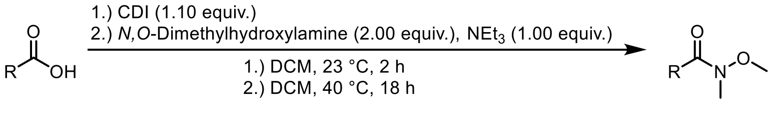

Carboxylic acid (1.00 equiv.) was suspended in DCM (0.4 M) at 23 °C and carbonyldiimidazole (CDI) (1.10 equiv.) was added in three portions as a solid. The resulting suspension was stirred at 23 °C for 2 h, during which the mixture typically turned into a clear solution. *N,O*-dimethylhydroxylamine hydrochloride (2.00 equiv.) and triethylamine (1.00 equiv.) were added at 23 °C, and the mixture was heated to 40 °C for 18 h. After cooling to 0 °C, 1 M HCl solution (4 mL/mmol) was added and the mixture was transferred into a separatory funnel. The organic phase was separated and the aqueous phase was extracted with DCM (3 × 2 mL/mmol). The combined organic phases were washed with aqueous saturated $NaHCO_3$ solution (4 mL/mmol), followed by brine (4 mL/mmol) and then dried over anhydrous sodium sulfate. The dried solution was filtered, and the filtrate was concentrated under reduced pressure. The crude residue was usually used in the next step without further purification. In cases where significant amounts of impurities were observed, the crude product was purified using flash column chromatography (ethyl acetate in heptanes, see individual products below).

2.2.3 General Procedure C: Addition of Grignard reagents to Weinreb amides

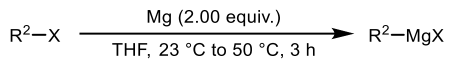

In a 2-neck flask equipped with a stirring bar, septum and a reflux condenser attached to an argon line, magnesium (chips, 2.00 equiv.) was suspended in THF (targeted product molarity 1 M) at 23 °C. A solution of alkyl halide (1.00 equiv.) in THF (0.75 mL/mmol R–X) was prepared at 23 °C. The alkyl halide solution was added to the suspension of magnesium: first approx. ¼ of the total amount was added, after which a color change and an exothermic reaction were observed. The suspension was then immersed into a 50 °C oil bath and the addition was slowly (over 15 min) continued. After the addition was done, the mixture was kept at 50 °C for 3 h. The gray suspension was then cooled to 23 °C (water bath) and stirring was stopped to allow solids to settle. The supernatant was titrated according to the procedure of Love and Jones[2] and used immediately for the next step (called "solution of the Grignard reagent"), leaving any solids behind (only the supernatant was transferred).

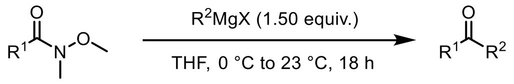

In a Schlenk flask Weinreb amide (1.00 equiv.) was dissolved in THF (0.5 M) and the solution was cooled to 0 °C. Solution of the Grignard reagent (1.50 equiv., according to titration, see above) was added dropwise at 0 °C, and the mixture was stirred for 18 h with slow warming to 23 °C (the flask was left in ice bath which melted overnight). The reaction mixture was cooled to 0 °C and the reaction was stopped by the addition of saturated aqueous NH_4Cl solution (5 mL/mmol) at 0 °C. The mixture was transferred into a separatory funnel (washing with DCM, 5 mL/mmol) and the organic phase was separated. The aqueous phase was extracted with Et_2O (3 × 5 mL/mmol), the combined organic layers were dried over anhydrous sodium sulfate, the dried solution was filtered, and the filtrate was concentrated under reduced pressure. The crude residue was purified by flash column chromatography (ethyl acetate in heptanes).

2.2.4 General Procedure D: Grignard addition to aldehydes

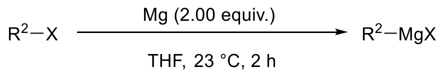

In a 2-neck flask equipped with a stirring bar, septum and a reflux condenser attached to an argon line, magnesium (chips, 2.00 equiv.) was suspended in THF (targeted product molarity 1 M) at 23 °C. A solution of alkyl halide (1.00 equiv.) in THF (0.75 mL/mmol R–X) was prepared at 23 °C. The alkyl halide solution was added to the suspension of magnesium: first approx. ¼ of the total amount was added, after which a color change and an exothermic reaction were observed. After the complete addition of the bromide solution, the mixture was stirred for additional 2 h at 23 °C. The concentration of the Grignard solution was determined by titration with iodine before use.[3]

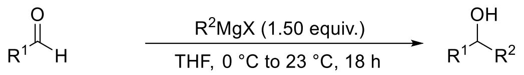

In a Schlenk flask an aldehyde (1.00 equiv.) was dissolved in THF (0.5 M) and the solution was cooled to 0 °C. Solution of the Grignard reagent (1.50 equiv., according to titration, see above) was added dropwise at 0 °C, and the mixture was stirred for 18 h with slow warming to 23 °C (the flask was left in ice bath which melted overnight). The reaction was stopped by the addition of saturated aqueous NH_4Cl solution (5 mL/mmol). The mixture was transferred into a separatory funnel (washing with DCM, 5 mL/mmol) and the organic phase was separated. The aqueous phase was extracted with DCM

(3 × 5 mL/mmol), the combined organic layers were dried over anhydrous sodium sulfate, the dried solution was filtered, and the filtrate was concentrated under reduced pressure. The crude residue was either used without further purification *or* purified by flash column chromatography (ethyl acetate in heptanes, see individual products).

2.2.5 General Procedure E: Grignard addition to nitriles

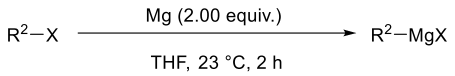

In a 2-neck flask equipped with a stirring bar, septum and a reflux condenser attached to an argon line, magnesium (chips, 2.00 equiv.) was suspended in THF (targeted product molarity 1 M) at 23 °C. A solution of alkyl halide (1.00 equiv.) in THF (0.75 mL/mmol R–X) was prepared at 23 °C. The alkyl halide solution was added to the suspension of magnesium: first approx. ¼ of the total amount was added, after which a color change and an exothermic reaction were observed. After the complete addition of the bromide solution, the mixture was stirred for additional 2 hours. The concentration of the Grignard solution was determined by titration with iodine before use.[3]

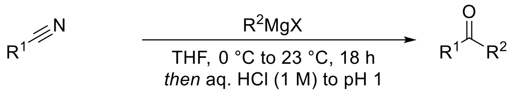

In a Schlenk flask the nitrile (1.00 equiv.) was dissolved in THF (2 mL/mmol) and the solution was cooled to 0 °C. Solution of the Grignard reagent (X equiv., see the product section below) was added dropwise at 0 °C, and the mixture was stirred for 18 h with slow warming to 23 °C (the flask was left in ice bath which melted overnight). The reaction mixture was poured into crushed ice and 1 M HCl was added until pH = 1. The mixture was allowed to stand for 15 min, after which the acid was neutralized with aqueous saturated $NaHCO_3$ solution, the mixture was transferred into a separatory funnel (washing with EtOAc) and the organic phase was separated. The aqueous phase was extracted with EtOAc (3 × 5 mL/mmol), the combined organic layers were dried over anhydrous sodium sulfate, the dried solution was filtered, and the filtrate was concentrated under reduced pressure. The crude residue was purified by flash column chromatography (ethyl acetate in heptanes).

2.2.6 General procedure F: Synthesis of δ-hydroxy ketones

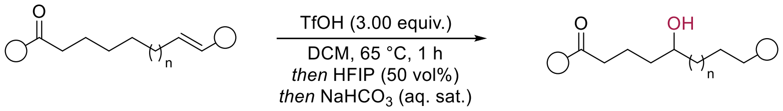

An unsaturated ketone (1.00 equiv.) was weighed into a small vial and dissolved in DCM (0.1 M) at 23 °C under air. Triflic acid (TfOH) (3.00 equiv.[a]) was added with a microsyringe at 23 °C, the vial was immediately closed with a screwcap and placed into a metal heating block (pre-heated to 65 °C[b], vial fully immersed, see Section 2.1.4) and the solution was stirred at 65 °C[b] for 1 h[b]. The vial was then placed into a cold-water bath (10 – 15 °C) for 5 min, after which the vial was opened and 50 vol% (of DCM volume) HFIP[c] were added. The vial was closed again, and the mixture was stirred for 5 min, after which aqueous saturated $NaHCO_3$ solution (20 mL/mmol) was added. The mixture was transferred into a small separatory funnel (washing with DCM), the phases were separated, and the aqueous phase was extracted with DCM (3 × 20 mL/mmol). The combined organic layers were washed with aqueous 2 M NaOH (20 mL/mmol), dried over anhydrous sodium sulfate, filtered and the filtrate was concentrated under reduced pressure (40 °C water bath).

The NMR yield was determined by analysis of the ^{1}H NMR spectrum of the crude reaction product (DCM-d_2, 1.00 equiv. mesitylene as internal standard). After measurement, the solution was concentrated under reduced pressure and the crude residue was purified by flash column chromatography (ethyl acetate in heptanes with 1% triethylamine additive).

[a]4.00 equiv. TfOH were used in cases when the substrates contained basic functionalities (*e.g.* **1q**), to account for quenching of 1.00 eq. by the base.

[b]Lower temperatures and modified reaction times were used in a few cases. Please refer to the individual products in Section 2.4.

[c]See Section 4.2 for a comprehensive discussion and analysis of the role of HFIP in the quenching procedure.

2.2.7 General procedure G: Synthesis of γ-hydroxy ketones

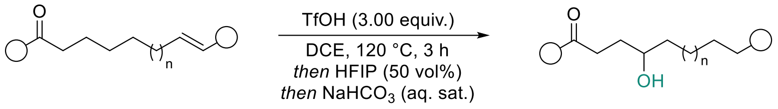

An unsaturated ketone (1.00 equiv.) was weighed into a small vial and dissolved in DCE (0.1 M) at 23 °C under air. Triflic acid (TfOH) (3.00 equiv.[a]) was added with a microsyringe at 23 °C, the vial was immediately closed with a screwcap and placed into a metal heating block (pre-heated to 120 °C[b], vial fully immersed, see Section 2.1.4) and the solution was stirred at 120 °C[b] for 3 h[b]. The vial was then placed into a cold-water bath (10 – 15 °C) for 5 min, after which the vial was opened and 50 vol% (of DCE volume) HFIP[c] were added. The vial was closed again, and the mixture was stirred for 5 min, after which aqueous saturated $NaHCO_3$ solution (20 mL/mmol) was added. The mixture was transferred into a small separatory funnel (washing with DCM), the phases were separated and the aqueous phase was extracted with DCM (3 × 20 mL/mmol). The combined organic layers were washed with aqueous 2 M NaOH (20 mL/mmol), dried over anhydrous sodium sulfate, filtered and the filtrate was concentrated under reduced pressure (40 °C water bath).

The NMR yield was determined by analysis of the ^{1}H NMR spectrum of the crude reaction product (DCM-d_2, 1.00 equiv. mesitylene as internal standard). After measurement, the solution was concentrated under reduced pressure and the crude residue was purified by flash column chromatography (ethyl acetate in heptanes with 1% triethylamine additive).

[a]4.00 equiv. TfOH were used in cases when the substrates contained basic functionalities (*e.g.* **2p**), to account for quenching of 1.00 eq. by the base.

[b]Lower temperatures and modified reaction times were used in some cases. Please refer to the individual products in Section 2.5.

[c]See Section 4.2 for a comprehensive discussion and analysis of the role of HFIP in the quenching procedure.

2.2.8 General procedure H: Synthesis of γ-hydroxy ketones with triethylammonium triflate additive

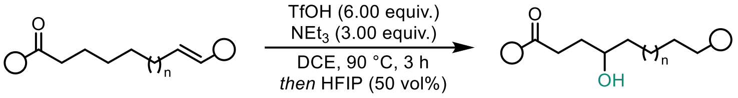

This procedure was used for substrates which suffered from polymerization issues (e.*g.* substrates with very long aliphatic chains, such as **2h**). We believe that the presence of excess TfO^- helps to suppress polymerization and/or increase the rate of migration of the positive charge. Identical results were obtained with pre-formed triethylammonium triflate (3.00 equiv. NEt_3 and 6.00 equiv. TfOH stirred in DCE for 15 min, followed by the addition of substrate).

An unsaturated ketone (1.00 equiv.) was weighed into a small vial equipped with a septum screwcap and dissolved in DCE (0.1 M) at 23 °C. Triethylamine (3.00 equiv.), followed by TfOH (6.00 equiv.) were added with a microsyringe at 23 °C, the vial was immediately closed with a screwcap and placed into a metal heating block (pre-heated to 90 °C, vial fully immersed, see Section 2.1.4) and the solution was stirred at 90 °C for 3 h. The vial was then placed into a cold-water bath (10 – 15 °C) for 5 min, after which the vial was opened and 50 vol% (of DCM volume) HFIP were added. The vial was closed again, and the mixture was stirred for 5 min, after which aqueous saturated $NaHCO_3$ solution (20 mL/mmol) was added. The mixture was transferred into a small separatory funnel (washing with DCM), the phases were separated, and the aqueous phase was extracted with DCM (3 × 20 mL/mmol). The combined organic layers were washed with aqueous 2 M NaOH (20 mL/mmol), dried over anhydrous sodium sulfate, filtered and the filtrate was concentrated under reduced pressure (40 °C water bath).

The NMR yield was determined by analysis of the ^1H NMR spectrum of the crude reaction product (DCM-d_2, 1.00 equiv. mesitylene as internal standard). After measurement, the solution was concentrated under reduced pressure and the crude residue was purified by flash column chromatography (ethyl acetate in heptanes with 1% triethylamine additive).

2.2.9 General procedure I: Interception with a halide

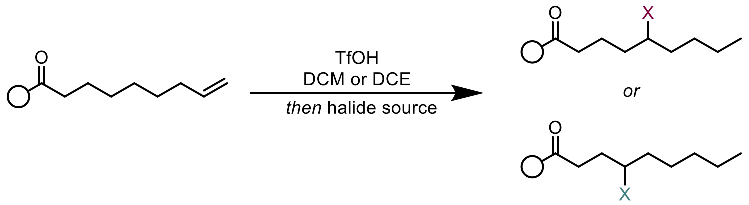

The corresponding alkenyl ketone was weighed into a small vial and dissolved either in DCM (for δ-functionalization, 0.1 M) or in DCE (for γ-functionalization, 0.1 M) at 23 °C under air. TfOH (see individual products for exact amount) was then added using a microsyringe at 23 °C, and the resulting solution was placed into a metal heating block (pre-heated to 65 °C for δ-functionalization and 120 °C for γ-functionalization, vial fully immersed, see Section 2.1.4) and the solution was stirred (see individual products for reaction time). The vial was then placed into a cold-water bath (10 – 15 °C), after which the halide source (see individual products for specification) was added in one portion, followed by further stirring (see individual products for time and temperature). Upon completion, an aqueous saturated $NaHCO_3$ solution (20 mL/mmol) was added. The mixture was transferred into a small separatory funnel (washing with DCM), and the aqueous phase was extracted with DCM (3 × 20 mL/mmol). The combined organic layers were dried over anhydrous magnesium sulfate, and the filtrate was concentrated under reduced pressure (40 °C water bath). The crude residue was purified using flash column chromatography (EtOAc in heptanes).

2.3 Preparation of starting materials

2.3.1 Weinreb amides

***N*-Methoxy-*N*-methylbenzamide**

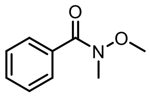

The title compound was prepared following general procedure A using benzoyl chloride (7.03 g, 50.00 mmol, 1.00 equiv.), *N,O*-dimethylhydroxylamine hydrochloride (4.87 g, 52.50 mmol, 1.00 equiv.) and triethylamine (13.9 mL, 100.00 mmol, 2.00 equiv.). The crude product (7.12 g) was used without further purification.

***N*-Methoxy-*N*-methyldec-9-enamide**

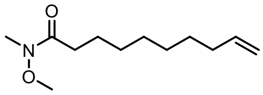

The title compound was prepared following general procedure B using 9-decenoic acid (4.59 g, 27.00 mmol, 1.00 equiv.), carbonyldiimidazole (4.96 g, 29.70 mmol, 1.10 equiv.) and *N,O*-dimethylhydroxylamine hydrochloride (5.26 g, 53.90 mmol, 2.00 equiv.). The crude product (6.34 g) was obtained as a colorless liquid and used without further purification.

***N*-Methoxy-*N*-methyloleamide**

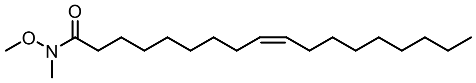

The title compound was prepared following general procedure B using oleic acid (1441 mg, 5.00 mmol, 1.00 equiv.), carbonyldiimidazole (1.09 g, 6.50 mmol, 1.30 equiv.) and *N*,*O*-dimethylhydroxylamine hydrochloride (975 mg, 10.00 mmol, 2.00 equiv.). The crude product (1.30 g) was used without further purification.

***N*-Methoxy-*N*,2-dimethylbenzamide**

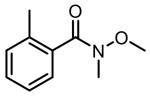

The title compound was prepared following general procedure A using *o*-toluoyl chloride (0.65 mL, 5.00 mmol, 1.00 equiv.), *N*,*O*-dimethylhydroxylamine hydrochloride (512 mg, 5.25 mmol, 1.05 equiv.) and triethylamine (2.09 mL, 15.00 mmol, 3.00 equiv.). The crude product (760 mg) was used without further purification.

4-(*tert*-Butyl)-*N*-methoxy-*N*-methylbenzamide

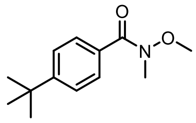

The title compound was prepared following general procedure A using 4-*tert*-butylbenzoyl chloride (983 mg, 5.00 mmol, 1.00 equiv.), *N*,*O*-dimethylhydroxylamine hydrochloride (488 mg, 5.00 mmol, 1.05 equiv.) and triethylamine (1.39 mL, 10.00 mmol, 2.00 equiv.). The crude product (1.10 g) was used without further purification.

***N*-Methoxy-*N*-methyl-4-(trifluoromethyl)benzamide**

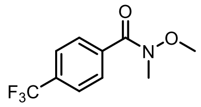

The title compound was prepared following general procedure A using 4-(trifluoromethyl)benzoyl chloride (7.66 mL, 50.00 mmol, 1.00 equiv.), *N,O*-dimethylhydroxylamine hydrochloride (5.12 g, 52.50 mmol, 1.05 equiv.) and triethylamine (20.9 mL, 150.00 mmol, 3.00 equiv.). The crude product (10.31 g) was used without further purification.

***N*-Methoxy-*N*-methyl-4-(trifluoromethoxy)benzamide**

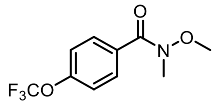

The title compound was prepared following general procedure A using 4-(trifluoromethoxy)benzoyl chloride (1.12 g, 5.00 mmol, 1.00 equiv.), *N,O*-dimethylhydroxylamine hydrochloride (512 mg, 5.25 mmol, 1.05 equiv.) and triethylamine (2.09 mL, 15.00 mmol, 3.00 equiv.). The crude product (980 mg) was used without further purification.

Methyl 4-(methoxy(methyl)carbamoyl)benzoate

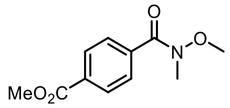

The title compound was prepared following general procedure A using methyl 4-chlorocarbonylbenzoate (1.05 g, 5.00 mmol, 1.00 equiv.), *N,O*-dimethylhydroxylamine hydrochloride (512 mg, 5.25 mmol, 1.05 equiv.) and triethylamine (2.09 mL, 15.00 mmol, 3.00 equiv.). The crude product (1.03 g) was used without further purification.

3-Fluoro-*N*-methoxy-*N*-methylbenzamide

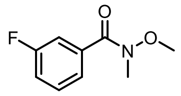

The title compound was prepared following general procedure A using 3-fluorobenzoyl chloride (634 mg, 4.00 mmol, 1.00 equiv.), *N*,*O*-dimethylhydroxylamine hydrochloride (410 mg, 4.20 mmol, 1.05 equiv.) and triethylamine (1.67 mL, 12.00 mmol, 3.00 equiv.). The crude product (710 mg) was used without further purification.

2-Hydroxy-*N*-methoxy-*N*-methylbenzamide

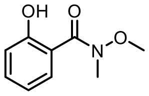

The title compound was prepared following general procedure B using salicylic acid (691 mg, 5.00 mmol, 1.00 equiv.), carbonyldiimidazole (919 mg, 5.50 mmol, 1.10 equiv.), *N*,*O*-dimethylhydroxylamine hydrochloride (975 mg, 10.00 mmol, 2.00 equiv.) and triethylamine (0.70 mL, 5.00 mmol, 1.00 equiv.). The crude product (580 mg) was obtained as yellow oil and used without further purification.

4-(Dimethylamino)-*N*-methoxy-*N*-methylbenzamide

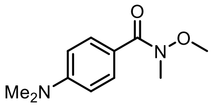

The title compound was prepared following general procedure A (washing step with 1 M HCl was omitted) using 4-(dimethylamino)benzoyl chloride (918 mg, 5.00 mmol, 1.00 equiv.), *N,O*-dimethylhydroxylamine hydrochloride (512 mg, 5.25 mmol, 1.05 equiv.) and triethylamine (2.09 mL, 15.00 mmol, 3.00 equiv.). The crude product (628 mg) was used without further purification.

***N*-Methoxy-*N*-methyl-2-(methylthio)benzamide**

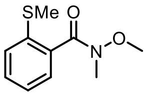

The title compound was prepared following general procedure B using 2-(methylthio)benzoic acid (841 mg, 5.00 mmol, 1.00 equiv.), carbonyldiimidazole (919 mg, 5.50 mmol, 1.10 equiv.), *N,O*-dimethylhydroxylamine hydrochloride (975 mg, 10.00 mmol, 2.00 equiv.) and triethylamine (0.70 mL, 5.00 mmol, 1.00 equiv.). Purification by flash column chromatography (10 – 40% EtOAc in heptanes) afforded the title compound as yellow oil (588 mg, 2.78 mmol, 56%).

All analytical data were in good accordance with the literature.[4]

^{1}H NMR (400 MHz, $CDCl_3$) δ 7.38 – 7.32 (m, 2H), 7.29 – 7.25 (m, 1H), 7.23 – 7.17 (m, 1H), 3.57 (br s, 3H), 3.30 (br s, 3H), 2.47 (s, 3H).

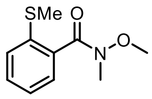

^{1}H NMR (400 MHz, $CDCl_3$)

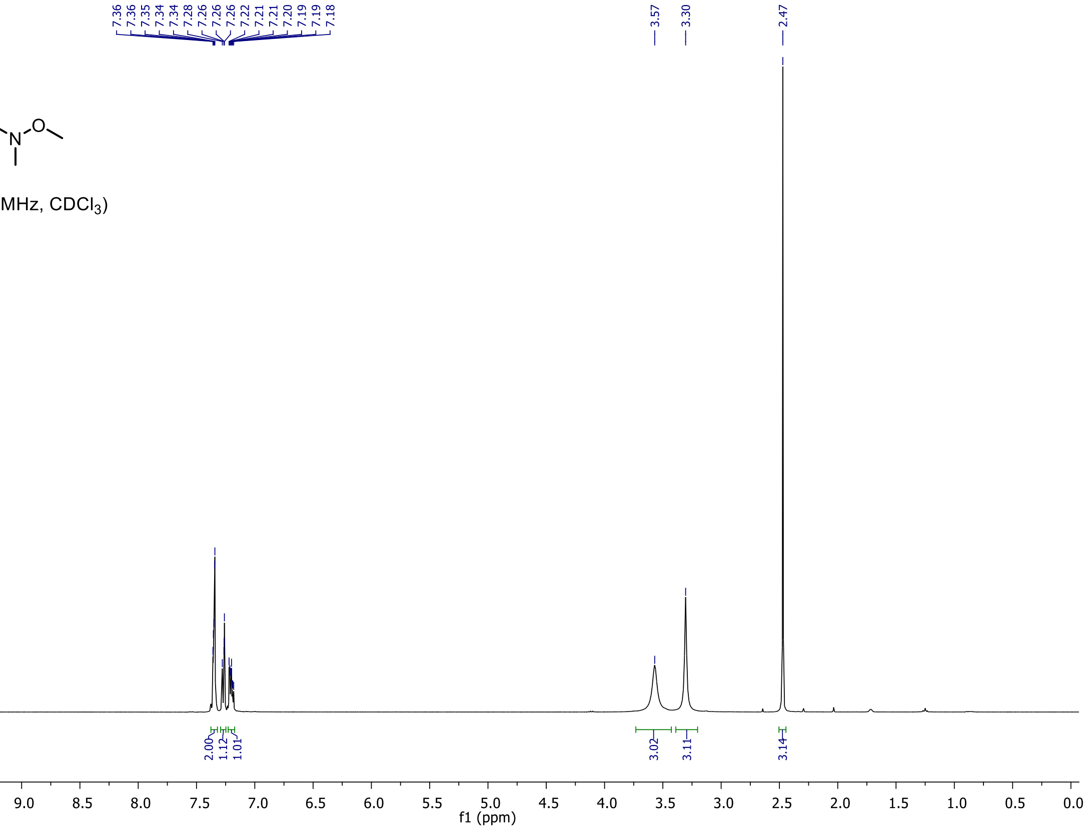

_N_,4-dimethoxy-_N_-methylbenzamide

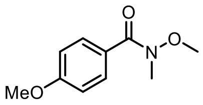

The title compound was prepared following general procedure A using 4-methoxybenzoyl chloride (1.00 g, 5.86 mmol, 1.00 equiv.), *N,O*-dimethylhydroxylamine hydrochloride (572 mg, 5.86 mmol, 1.00 equiv.) and triethylamine (1.63 mL, 11.70 mmol, 2.00 equiv.). The crude product (1.10 g) was used without further purification.

_N_-Methoxy-_N_-methylthiophene-2-carboxamide

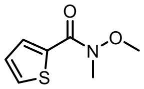

The title compound was prepared following general procedure A using 2-thienoyl chloride (1.00 g, 6.75 mmol, 1.00 equiv.), *N,O*-dimethylhydroxylamine hydrochloride (659 mg, 6.75 mmol, 1.00 equiv.) and triethylamine (1.88 mL, 13.5 mmol, 2.00 equiv.). The crude product (0.96 g) was used without further purification.

_N_-Methoxy-_N_-methylfuran-2-carboxamide

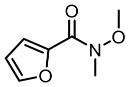

The title compound was prepared following general procedure A using 2-furoyl chloride (0.49 mL, 5.00 mmol, 1.00 equiv.), *N,O*-dimethylhydroxylamine hydrochloride (512 mg, 5.25 mmol, 1.05 equiv.) and triethylamine (1.39 mL, 15.00 mmol, 3.00 equiv.). The crude product (700 mg) was used without further purification.

_N_-Methoxy-_N_-methylferrocene-1-carboxamide

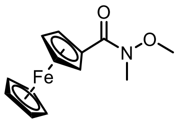

The title compound was prepared following general procedure B using ferrocene monocarboxylic acid (1.15 g, 5.00 mmol, 1.00 equiv.), carbonyldiimidazole (919 mg, 5.50 mmol, 1.10 equiv.), *N*,*O*-dimethylhydroxylamine hydrochloride (975 mg, 10.00 mmol, 2.00 equiv.) and triethylamine (0.70 mL, 5.00 mmol, 1.00 equiv.). The crude product (1.23 g) was obtained as red liquid and used without further purification.

3-(Difluoromethyl)-*N*-methoxy-*N*,1-dimethyl-1H-pyrazole-4-carboxamide

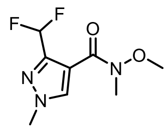

The title compound was prepared following general procedure B using 3-(difluoromethyl)-1-methyl-1H-pyrazole-4-carboxylic acid (881 mg, 5.00 mmol, 1.00 equiv.), carbonyldiimidazole (419 mg, 5.50 mmol, 1.10 equiv.) and *N,O*-dimethylhydroxylamine hydrochloride (975 mg, 10.0 mmol, 2.00 equiv.). The crude product (1.13 g) was obtained as a colorless liquid and used without further purification.

***N*-Methoxy-*N*-methyladamantane-1-carboxamide**

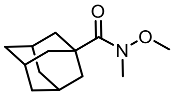

The title compound was prepared following general procedure B using 1-adamantanecarboxylic acid (920 mg, 5.00 mmol, 1.00 equiv.), carbonyldiimidazole (919 mg, 5.50 mmol, 1.10 equiv.), *N*,*O*-dimethylhydroxylamine hydrochloride (975 mg, 10.00 mmol, 2.00 equiv.) and triethylamine (0.70 mL, 5.00 mmol, 1.00 equiv.). The crude product (900 mg) was obtained as colorless liquid and used without further purification.

3-(4,5-Diphenyloxazol-2-yl)-*N*-methoxy-*N*-methylpropanamide

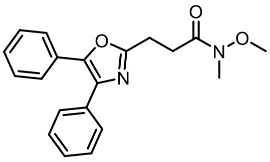

The title compound was prepared following general procedure B using oxaprozine (1.47 g, 5.00 mmol, 1.00 equiv.), carbonyldiimidazole (919 mg, 5.50 mmol, 1.10 equiv.), *N*,*O*-dimethylhydroxylamine hydrochloride (975 mg, 10.00 mmol, 2.00 equiv.) and triethylamine (0.70 mL, 5.00 mmol, 1.00 equiv.). The crude product (1.33 mg) was obtained as colorless crystals and used without further purification.

4-(*N*,*N*-Dipropylsulfamoyl)-*N*-methoxy-*N*-methylbenzamide

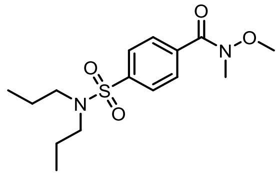

The title compound was prepared following general procedure B using probenecid (1.46 g, 5.00 mmol, 1.00 equiv.), carbonyldiimidazole (919 mg, 5.50 mmol, 1.10 equiv.), *N*,*O*-dimethylhydroxylamine hydrochloride (975 mg, 10.00 mmol, 2.00 equiv.) and triethylamine (0.70 mL, 5.00 mmol, 1.00 equiv.). Purification by flash column chromatography (10 – 40% EtOAc in heptanes) afforded the title compound (1.53 g, 4.66 mmol, 93%) as colorless solid.

All analytical data were in good agreement with those reported in literature.[5]

^{1}H NMR (700 MHz, CDCl$_3$): δ 7.86 – 7.81 (m, 2H), 7.78 (m, 2H), 3.51 (s, 3H), 3.38 (s, 3H), 3.11 – 3.07 (m, 4H), 1.55 (m, 4H), 0.87 (t, *J* = 7.4 Hz, 6H).

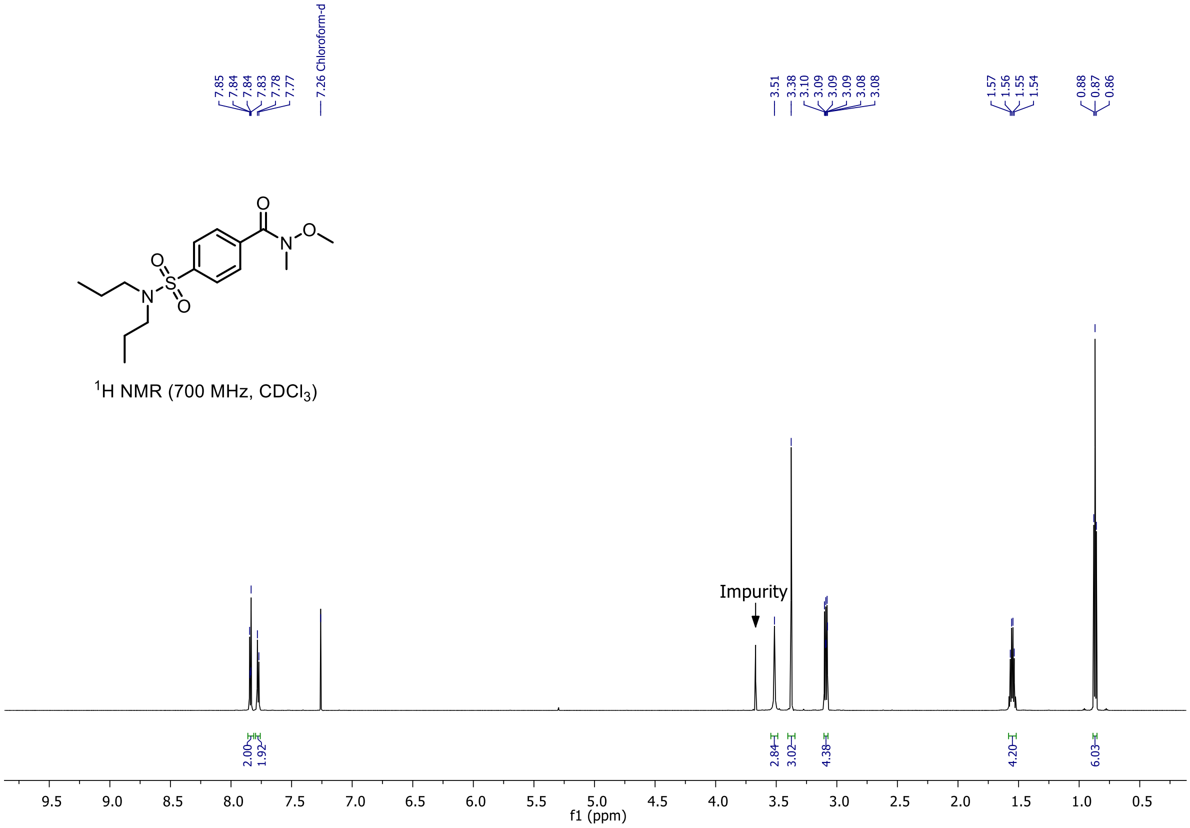

N-Methoxy-N-methyl-2-naphthamide

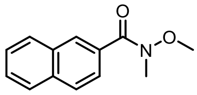

The title compound was prepared following general procedure A using 2-naphthoyl chloride (953 mg, 5.00 mmol, 1.00 equiv.), *N,O*-dimethylhydroxylamine hydrochloride (488 mg, 5.00 mmol, 1.00 equiv.) and triethylamine (1.39 mL, 10.00 mmol, 2.00 equiv.). The crude product (850 mg) was used without further purification.

4-Chloro-N-methoxy-N-methylbenzamide

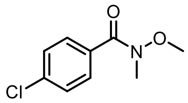

The title compound was prepared following general procedure A using 4-chlorobenzoyl chloride (3.57 g, 20.00 mmol, 1.00 equiv.), *N,O*-dimethylhydroxylamine hydrochloride (1.99 g, 20.00 mmol, 1.00 equiv.) and triethylamine (5.58 mL, 40.00 mmol, 2.00 equiv.). The crude product (3.81 g) was used without further purification.

2.3.2 Alkenyl ketones

1-Phenylhept-6-en-1-one

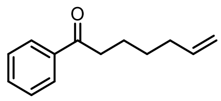

The title compound was prepared following general procedure C using *N*-methoxy-*N*-methylbenzamide (1.16 g, 7.00 mmol, 1.00 equiv.) and a solution of 5-hexenylmagnesium bromide (1.50 equiv.). Purification by flash column chromatography (0 – 20% EtOAc in heptanes) afforded the title compound (1.15 g, 87%, 6.09 mmol) as a colorless oil.

All analytical data were in good agreement with those reported in literature.[6]

^{1}H NMR (400 MHz, CDCl$_3$): δ 7.99 – 7.87 (m, 2H), 7.61 – 7.51 (m, 1H), 7.51 – 7.38 (m, 2H), 5.82 (ddt, *J* = 16.9, 10.2, 6.7 Hz, 1H), 5.08 – 4.88 (m, 2H), 3.06 – 2.82 (t, *J* = 7.4 Hz, 2H), 2.11 (dd, *J* = 14.3, 7.2 Hz, 2H), 1.77 (dt, *J* = 15.1, 7.4 Hz, 2H), 1.54 – 1.35 (m, 2H).

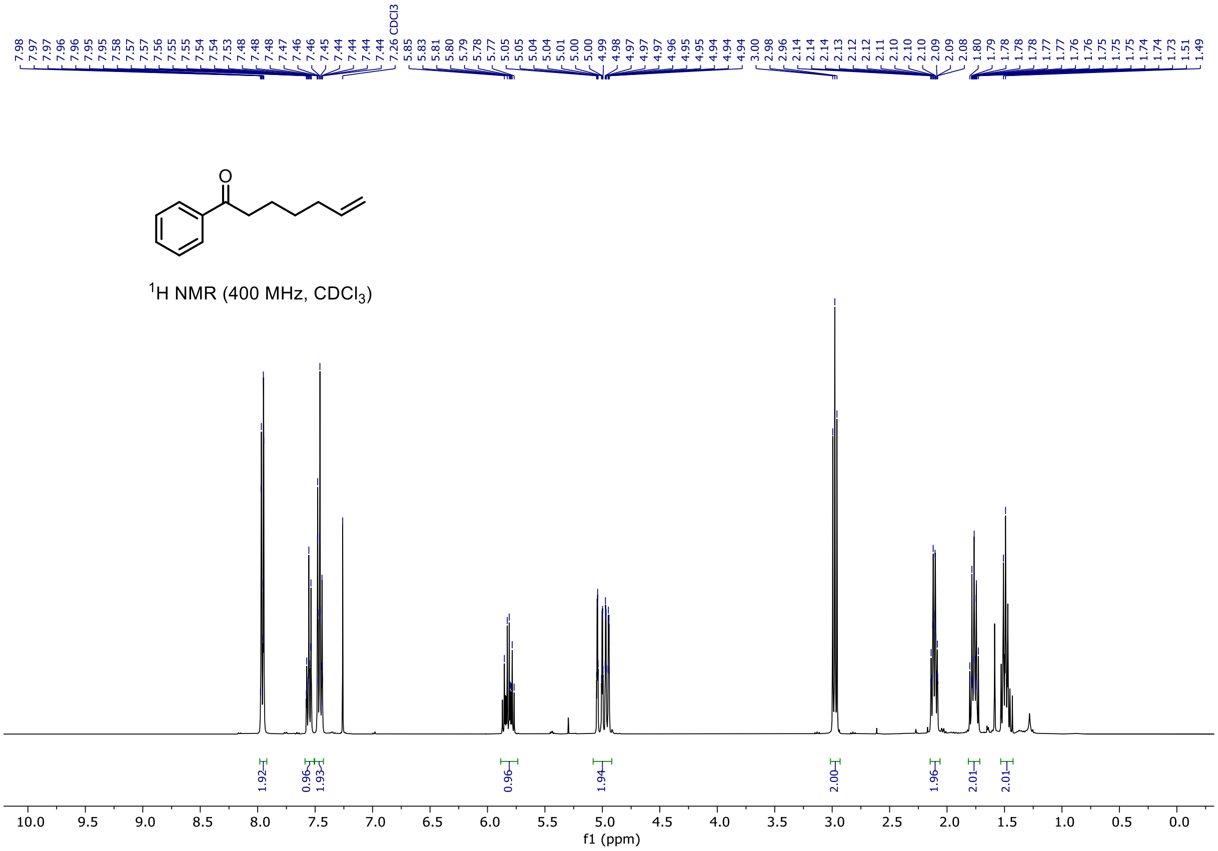

1-Phenylnon-8-en-1-one

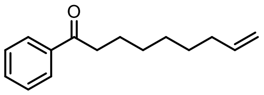

The title compound was prepared following general procedure C using *N*-methoxy-*N*-methylbenzamide (826 mg, 5.00 mmol, 1.00 equiv.) and a solution of 7-octenylmagnesium bromide (1.50 equiv.). Purification by flash column chromatography (0 – 20% EtOAc in heptanes) afforded the title compound (942 mg, 4.35 mmol, 87%) as a colorless oil.

^{1}H NMR (400 MHz, $CDCl_3$): δ 8.02 – 7.90 (m, 2H), 7.58 – 7.54 (m, 1H), 7.51 – 7.42 (m, 2H), 5.81 (ddt, *J* = 16.9, 10.2, 6.7 Hz, 1H), 5.05 – 4.87 (m, 2H), 3.02 – 2.91 (m, 2H), 2.11 – 2.00 (m, 2H), 1.81 – 1.66 (m, 2H), 1.45 – 1.30 (m, 6H).

All analytical data were in good agreement with those reported in literature.[7]

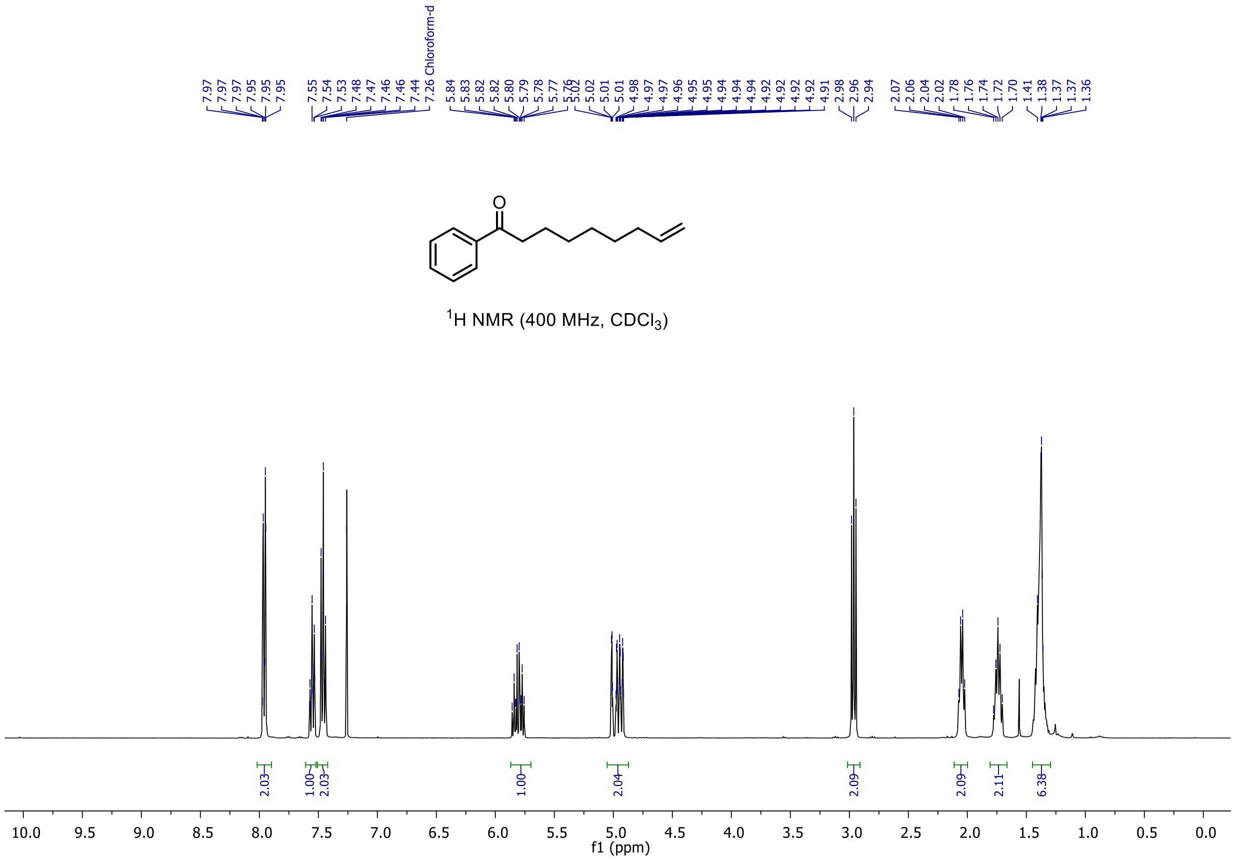

1-Phenyldec-9-en-1-one

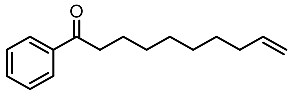

Phenylmagnesium bromide solution was prepared following general procedure C using bromobenzene (0.31 mL, 3.00 mmol, 1.00 equiv.) and magnesium (146 mg, 6.00 mmol, 2.00 equiv.).

The title compound was prepared following general procedure C using Weinreb amide *N*-methoxy-*N*-methyldec-9-enamide (427 mg, 2.00 mmol, 1.00 equiv.) and a solution of phenylmagnesium bromide (1.50 equiv.). Purification by flash column chromatography (0 – 10% EtOAc in heptanes) afforded the title compound as a colorless oil (363 mg, 1.58 mmol, 79%).

^{1}H NMR (400 MHz, $CDCl_3$) δ 8.01 – 7.92 (m, 2H), 7.59 – 7.51 (m, 1H), 7.50 – 7.42 (m, 2H), 5.81 (ddt, *J* = 16.9, 10.2, 6.7 Hz, 1H), 4.99 (ddd, *J* = 17.0, 3.7, 1.6 Hz, 1H), 4.93 (ddt, *J* = 10.2, 2.3, 1.2 Hz, 1H), 2.96 (t, *J* = 7.6 Hz, 2H), 2.08 – 2.00 (m, 2H), 1.79 – 1.68 (m, 2H), 1.44 – 1.28 (m, 8H).

^{13}C NMR (101 MHz, $CDCl_3$) δ 200.7 (C), 139.3 (CH), 137.2 (C), 133.0 (CH), 128.7 (2CH), 128.2 (2CH), 114.3 (CH_2), 38.7 (CH_2), 33.9 (CH_2), 29.5 (CH_2), 29.1 ($2CH_2$), 29.0 (CH_2), 24.5 (CH_2).

IR (neat) v_{max}: 3063, 2926, 2854, 1686, 1448, 1217, 910, 771, 740, 690.

HRMS (ESI$^+$): exact mass calculated for $[M+H]^+$ $(C_{16}H_{23}O)^+$ requires *m*/*z* 231.1743, found *m*/*z* 231.1742.

1-Phenyldec-9-en-1-one

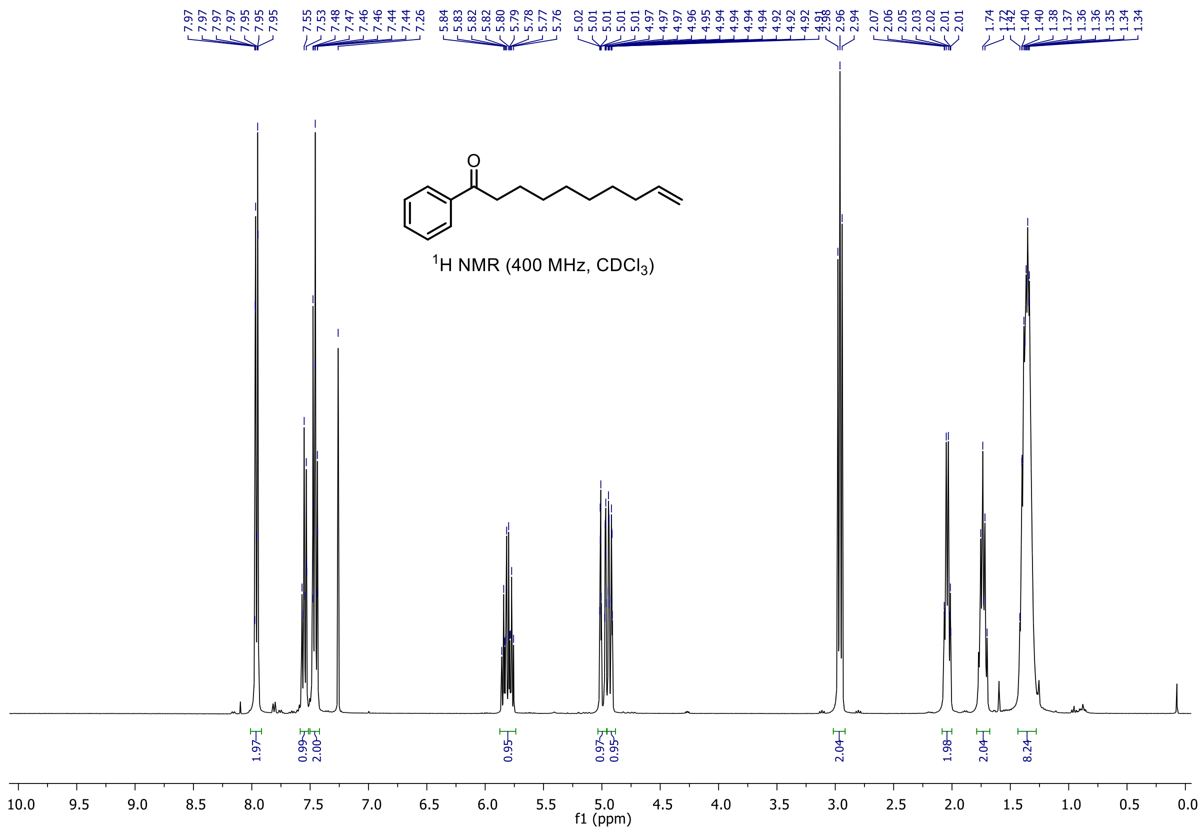

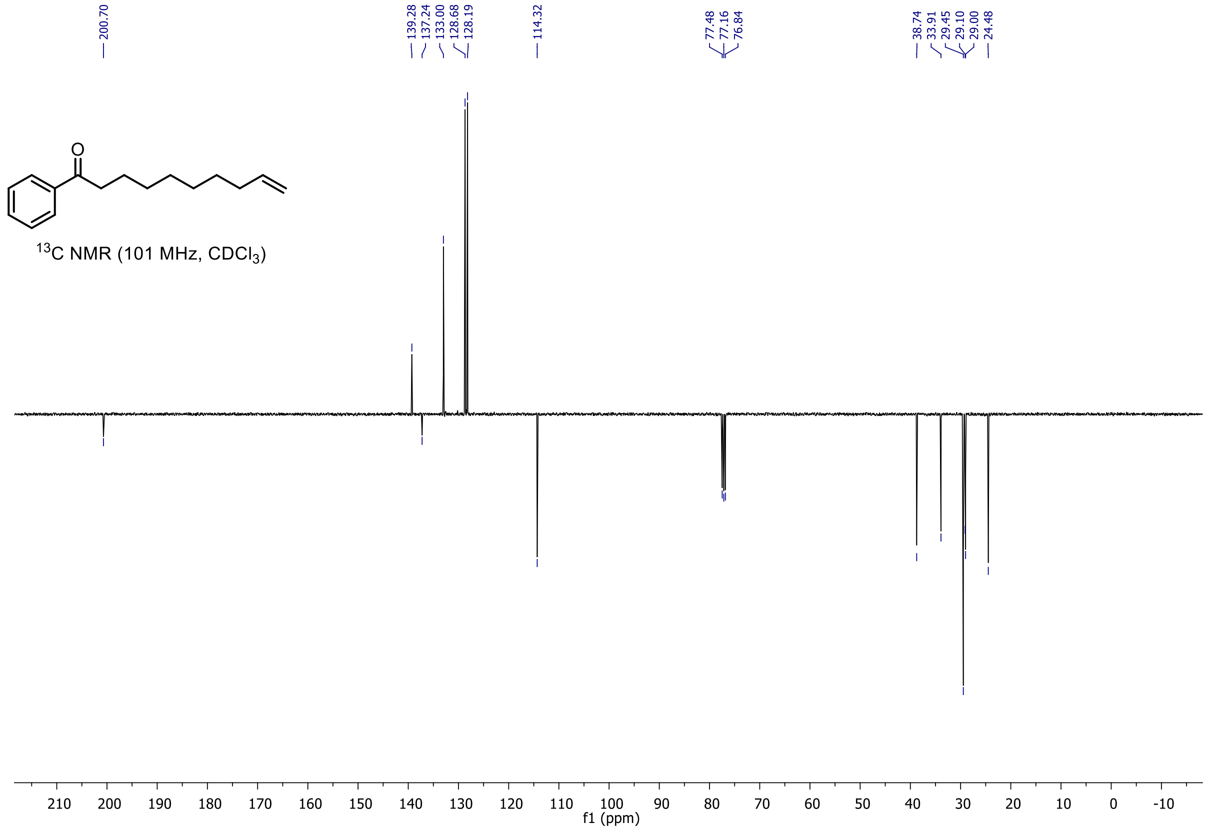

1-Phenyldodec-11-en-1-one

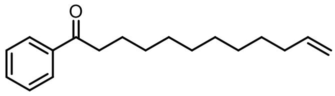

10-undecenylmagnesium bromide solution was prepared following general procedure C using 11-bromo-1-undecene (0.99 mL, 4.50 mmol, 1.00 equiv.) and magnesium (219 mg, 9.00 mmol, 2.00 equiv.).

The title compound was prepared following general procedure C using *N*-methoxy-*N*-methylbenzamide (496 mg, 3.00 mmol, 1.00 equiv.) and a solution of 10-undecenylmagnesium bromide (1.40 equiv.). Purification by flash column chromatography (0 – 10% EtOAc in heptanes) afforded the title compound as a colorless solid (504 mg, 1.95 mmol, 65%).

^{1}H NMR (400 MHz, $CDCl_3$) δ 8.01 – 7.89 (m, 2H), 7.55 (ddd, *J* = 6.7, 3.9, 1.3 Hz, 1H), 7.49 – 7.39 (m, 2H), 5.81 (ddt, *J* = 16.9, 10.2, 6.7 Hz, 1H), 4.99 (ddd, *J* = 17.1, 3.7, 1.6 Hz, 1H), 4.93 (ddt, *J* = 10.2, 2.3, 1.2 Hz, 1H), 2.96 (t, *J* = 7.4 Hz, 2H), 2.07 – 2.00 (m, 2H), 1.72 (dd, *J* = 14.7, 7.3 Hz, 2H), 1.40 – 1.23 (m, 12H).

^{13}C NMR (101 MHz, $CDCl_3$) δ 200.8 (C), 139.4 (CH), 137.3 (C), 133.0 (CH), 128.7 (2CH), 128.2 (2CH), 114.3 (CH_2), 38.8 (CH_2), 34.0 (CH_2), 29.60 (CH_2), 29.58 ($2CH_2$), 29.5 (CH_2), 29.3 (CH_2), 29.1 (CH_2), 24.5 (CH_2).

IR (neat) ν_{max}: 3073, 2924, 2853, 1686, 1448, 1218, 909, 742, 690.

HRMS (ESI$^+$): exact mass calculated for $[M+Na]^+$ $(C_{18}H_{26}ONa)^+$ requires *m*/*z* 281.1876, found *m*/*z* 281.1876.

1-Phenyldodec-11-en-1-one

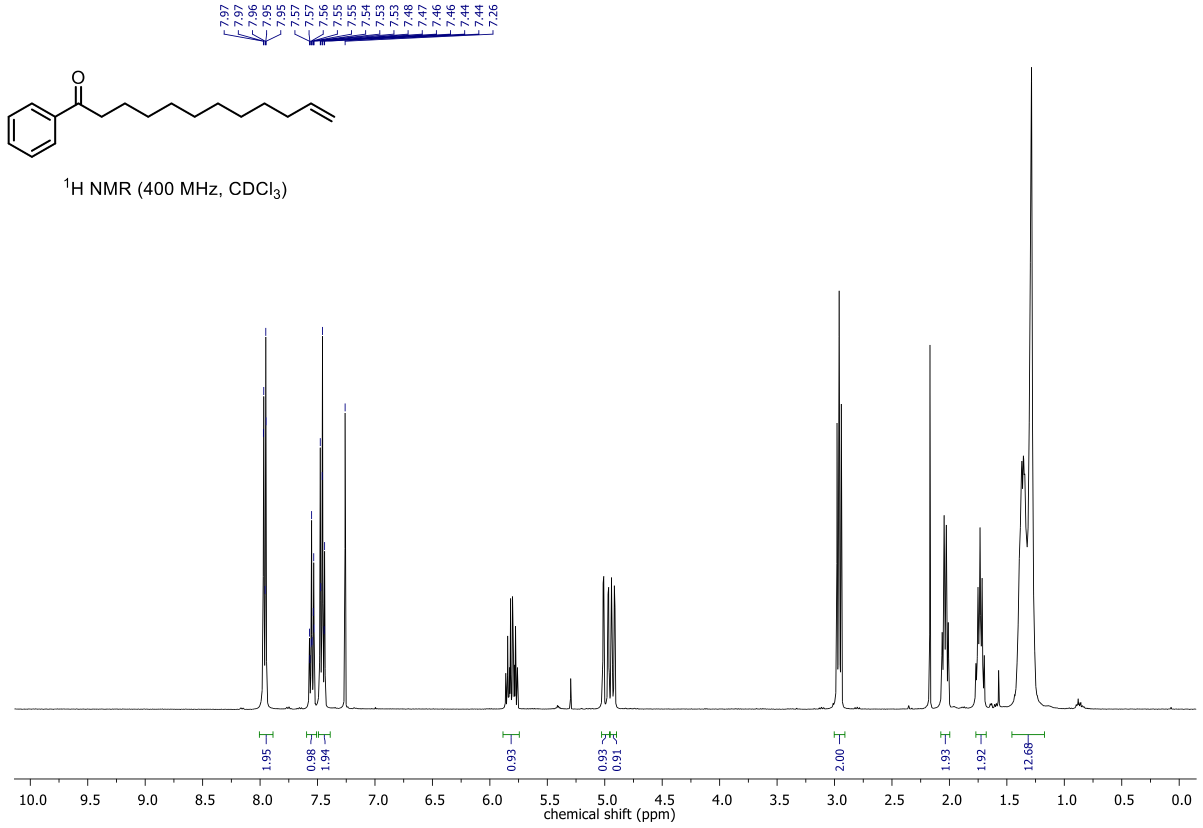

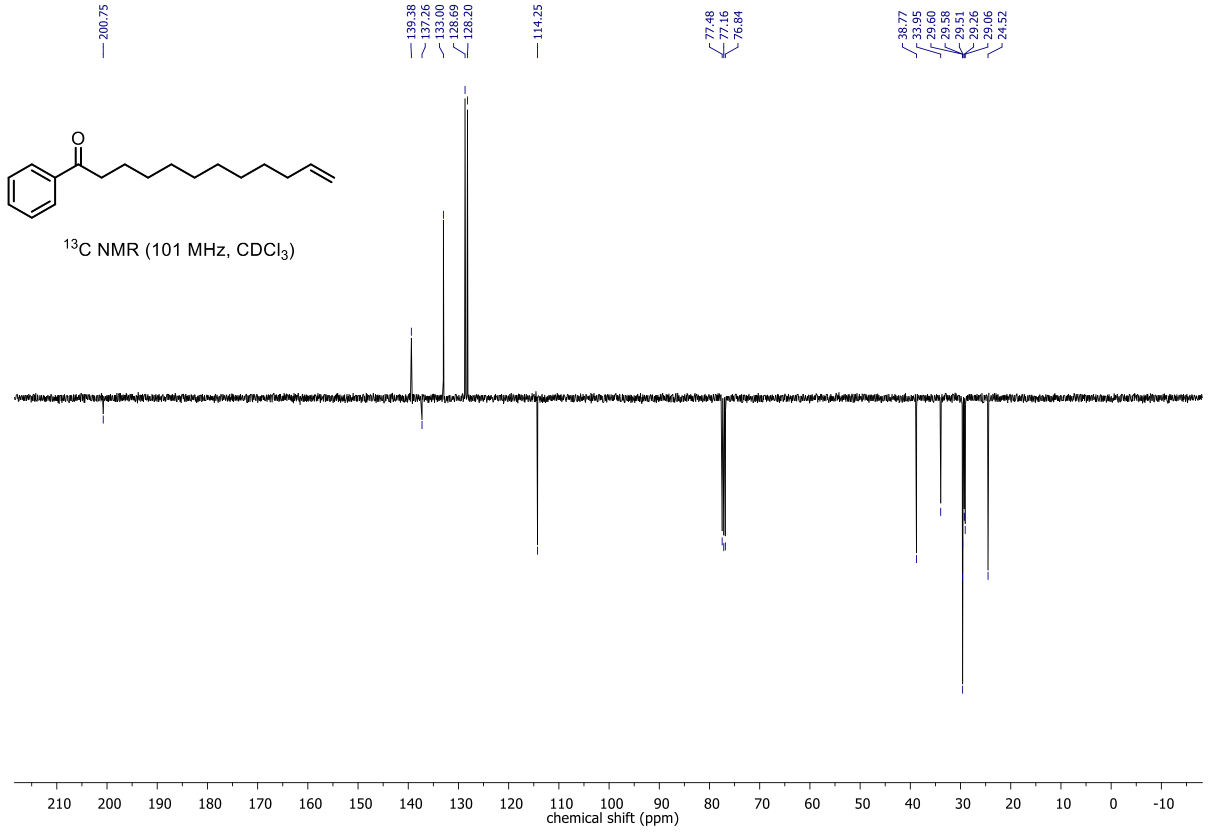

(*E*)-1-Phenylnon-6-en-1-one

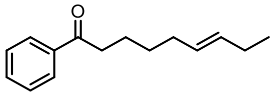

The compound was prepared following a procedure reported by Denard *et al.*[8]

In a glass vial *trans*-hex-3-ene (1.24 mL, 10.00 mmol, 10.00 equiv.) was dissolved in DCM (2 mL, 5 M) and Hoveyda-Grubbs 2nd generation catalyst (18.8 mg, 0.03 mmol, 3.00 mol%) was added. 1-Phenylhept-6-en-1-one (188 mg, 1.00 mmol, 1.00 equiv.) was dissolved in DCM (2 mL) and added to the solution over 30 min with a syringe pump. The resulting mixture was stirred for 90 min and then directly purified by flash column chromatography (0 – 50% EtOAc in heptanes), affording the title compound (100 mg, 0.64 mmol, 64%) as a colorless liquid.

^{1}H NMR (400 MHz, $CDCl_3$): δ 7.99 – 7.93 (m, 2H), 7.59 – 7.51 (m, 1H), 7.50 – 7.42 (m, 2H), 5.52 – 5.30 (m, 2H), 3.01 – 2.92 (m, 2H), 2.14 – 1.91 (m, 4H), 1.83 – 1.68 (m, 2H), 1.50 – 1.39 (m, 2H), 0.96 (t, *J* = 7.4 Hz, 3H).

^{13}C NMR (101 MHz, $CDCl_3$): δ 200.7, 137.2, 133.0, 132.6, 128.9, 128.7 (2C), 128.2 (2C), 38.6, 32.5, 29.4, 25.7, 24.0, 14.1.

IR (neat) v_{max}: 2960, 2931, 2853, 1683, 1597, 1580, 1448, 1409, 1357, 1275, 1219, 1196, 1179, 1072.

HRMS (ESI$^+$): exact mass calculated for $[M+Na]^+$ $(C_{15}H_{20}ONa)^+$ requires *m/z* 239.1406, found *m/z* 239.1406.

(*E*)-1-Phenylnon-6-en-1-one

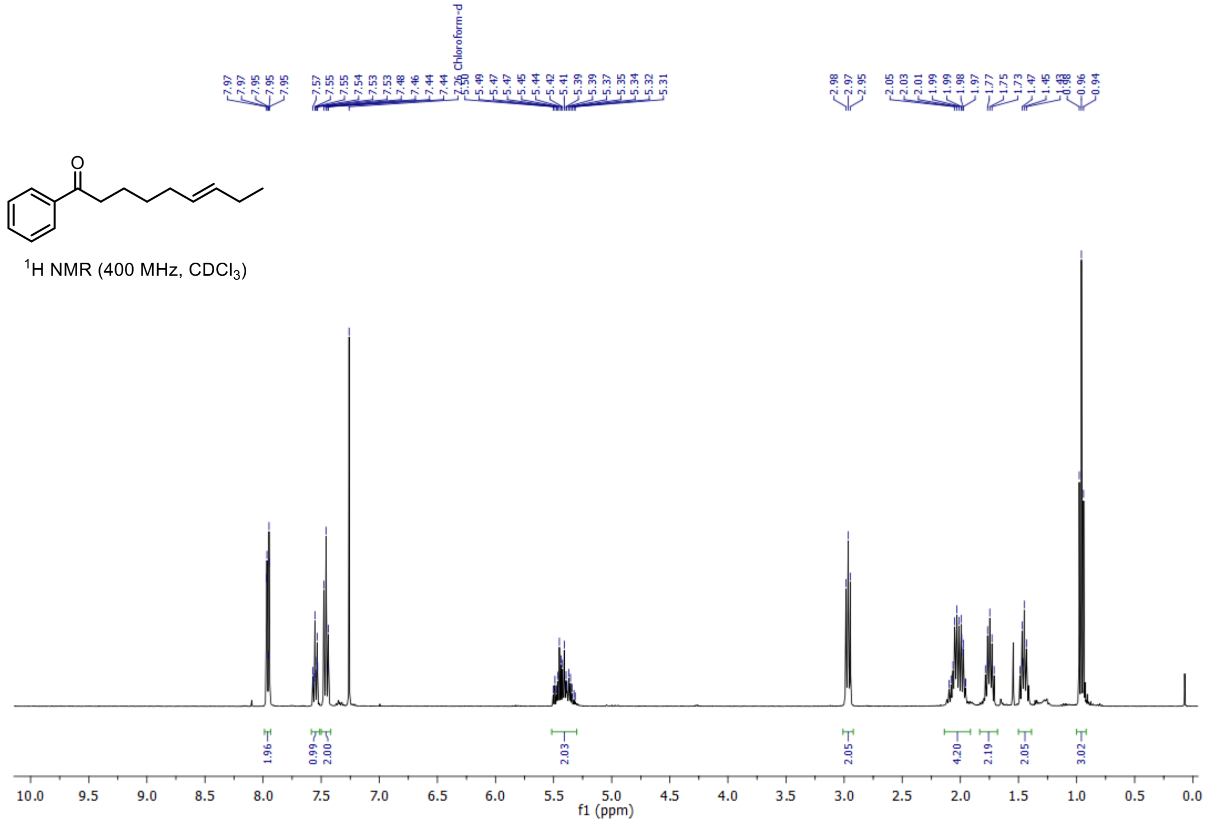

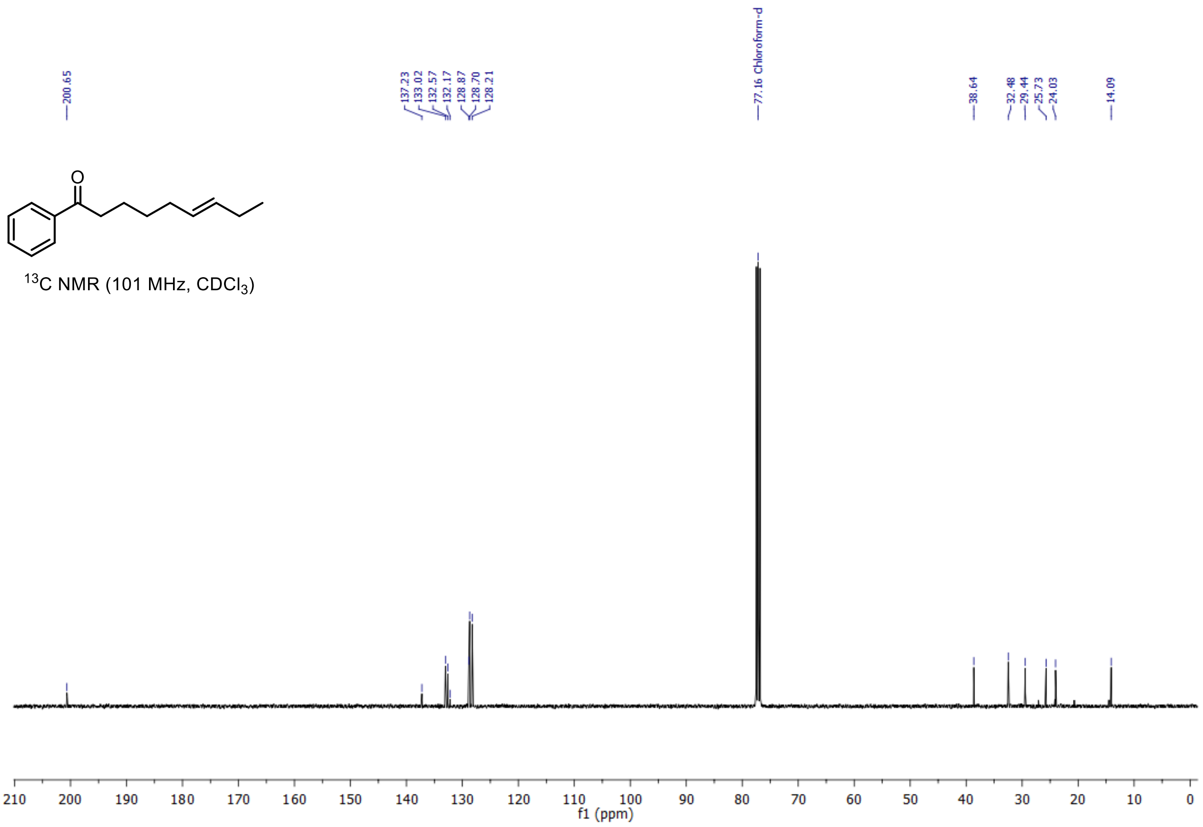

(*E*)-1-Phenylundec-8-en-1-one

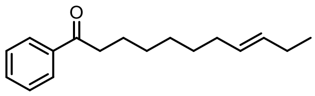

The compound was prepared following a procedure reported in literature.[8]

In a glass vial *trans*-hex-3-ene (1.24 mL, 10.00 mmol, 10.00 equiv.) was dissolved in DCM (2 mL, 5.0 M) and Hoveyda-Grubbs 2nd generation catalyst (18.8 mg, 0.03 mmol, 3.00 mol%) was added. 1-Phenylnon-8-en-1-one (216 mg, 1.00 mmol, 1.00 equiv.) was dissolved in DCM (2 mL) and added to the solution over 30 min with a syringe pump. The resulting mixture was stirred for 90 min and then directly purified by flash column chromatography (0 – 100% EtOAc in heptanes), affording the title compound (206 mg, 0.84 mmol, 84%) as a colorless liquid.

^{1}H NMR (400 MHz, $CDCl_3$): δ 8.00 – 7.92 (m, 2H), 7.58 – 7.52 (m, 1H), 7.50 – 7.42 (m, 2H), 5.51 – 5.29 (m, 2H), 2.96 (t, *J* = 7.4 Hz, 2H), 2.09 – 1.90 (m, 4H), 1.83 – 1.65 (m, 2H), 1.44 – 1.31 (m, 6H), 0.96 (t, *J* = 7.4 Hz, 3H).

^{13}C NMR (101 MHz, $CDCl_3$): δ 200.7, 137.3, 133.0, 132.2, 129.3, 128.7 (2C), 128.2 (2C), 38.8, 32.6, 29.6, 29.4, 29.1, 25.7, 24.5, 14.1.

IR (neat) v_{max}: 2925, 2853, 1685, 1597, 1580, 1448, 1409, 1368, 1211, 1179, 966, 750, 689, 656, 602, 569.

HRMS (ESI$^+$): exact mass calculated for $[M+Na]^+$ $(C_{17}H_{24}ONa)^+$ requires *m/z* 267.1719, found *m/z* 267.1713.

(*E*)-1-Phenylundec-8-en-1-one

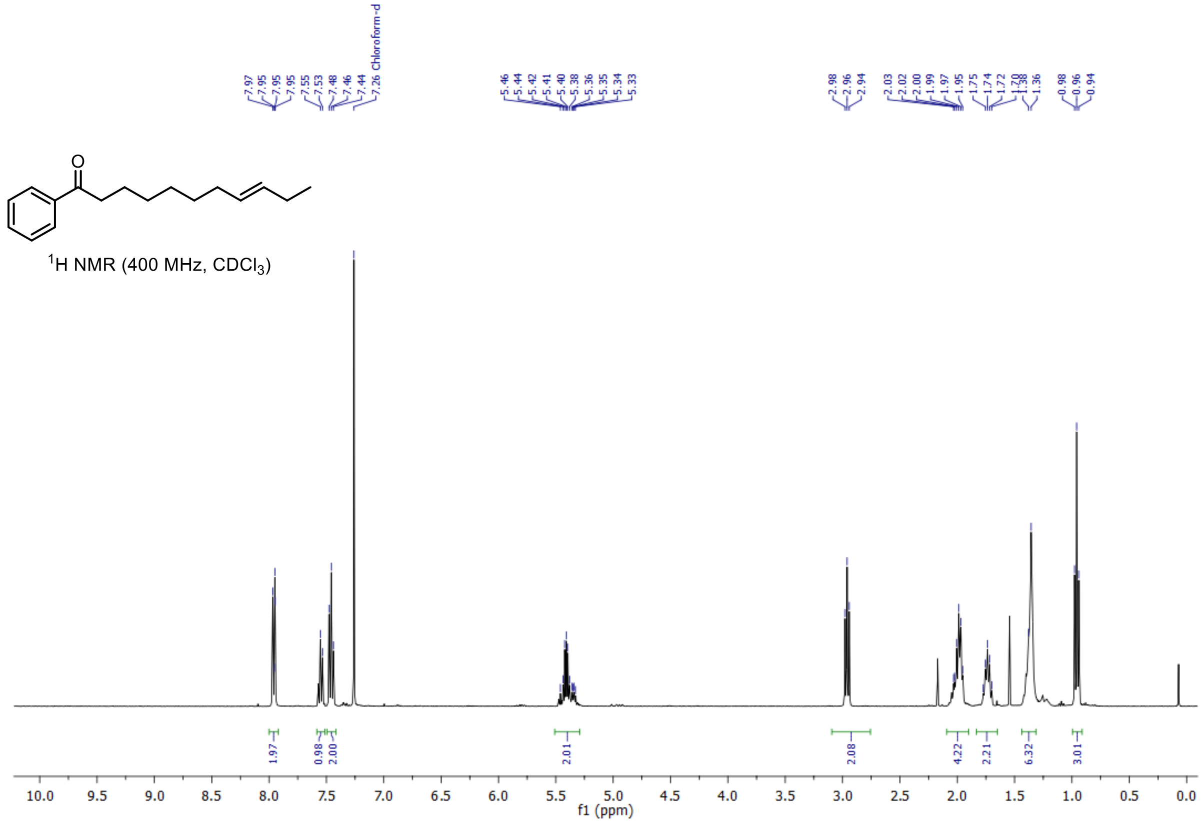

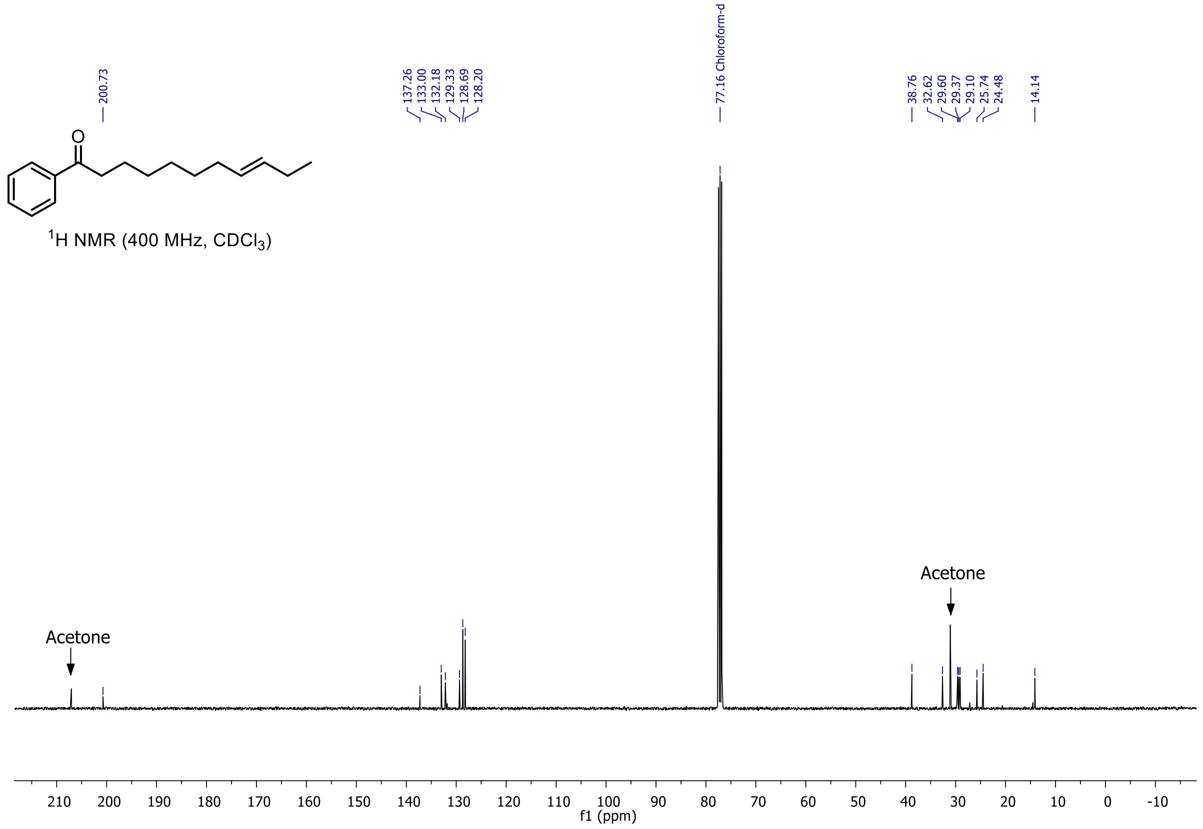

(*E*)-10,10,10-Trifluoro-1-phenyldec-7-en-1-one

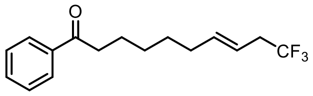

The title compound was prepared according to a literature procedure on 1.0 mmol scale.[9]

Purification by flash column chromatography (0 – 10% EtOAc in heptanes) afforded the title compound as a colorless liquid and a single isomer (91.7 mg, 0.32 mmol, 32%).

^{1}H NMR (400 MHz, $CDCl_3$): δ 8.02 – 7.88 (m, 2H), 7.59 – 7.52 (m, 1H), 7.49 – 7.43 (m, 2H), 5.76 – 5.57 (m, 1H), 5.47 – 5.29 (m, 1H), 2.96 (dd, *J* = 9.1, 5.6 Hz, 2H), 2.82 – 2.65 (m, 2H), 2.08 (q, *J* = 6.7 Hz, 2H), 1.82 – 1.70 (m, 2H), 1.49 – 1.34 (m, 4H).

^{13}C NMR (101 MHz, $CDCl_3$): δ 200.5, 138.3, 137.2, 133.1, 128.7 (2C), 128.2 (2C), 126.2 (q, *J* = 276.4 Hz), 117.9 (q, *J* = 3.4 Hz), 38.6, 37.5 (q, *J* = 29.6 Hz), 32.4, 28.9 (2C), 24.2.

^{19}F NMR (376 MHz, $CDCl_3$): δ -66.7.

IR (neat) v_{max}: 2932, 2857, 1684, 1248, 1216, 1130, 1064, 970, 731, 690.

HRMS (ESI$^+$): exact mass calculated for $[M+Na]^+$ $(C_{16}H_{19}F_3ONa)^+$ requires *m/z* 307.1280, found *m/z* 307.1284.

(*E*)-10,10,10-Trifluoro-1-phenyldec-7-en-1-one

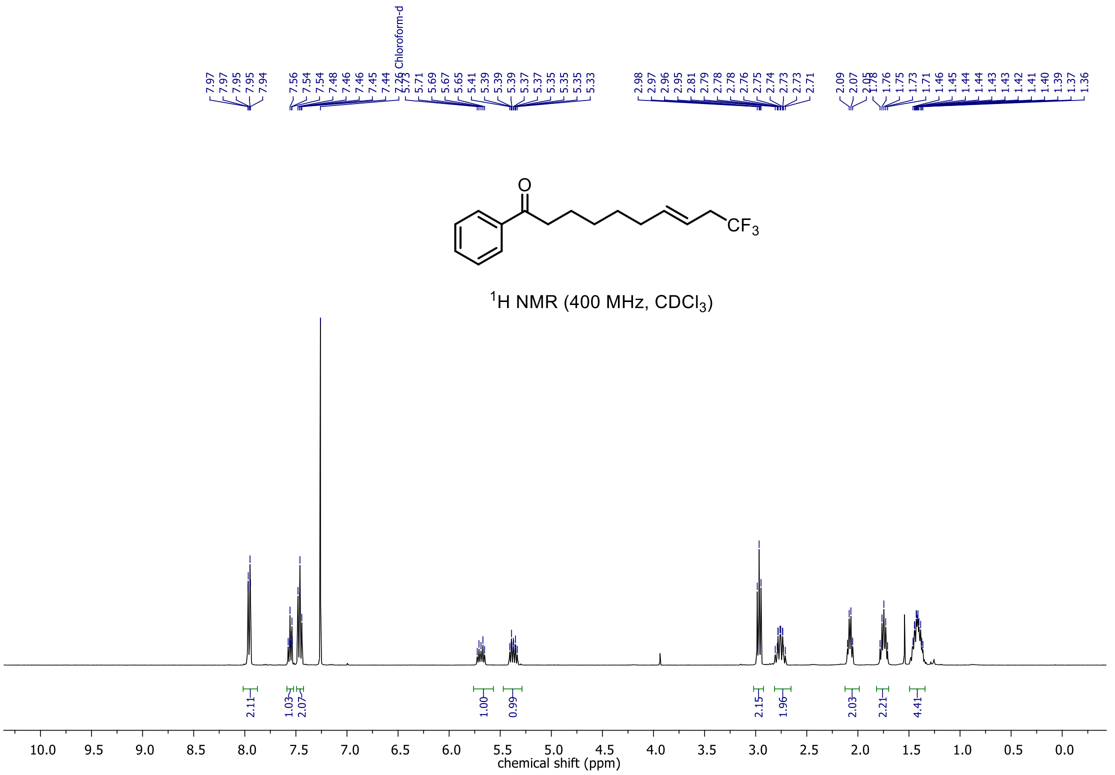

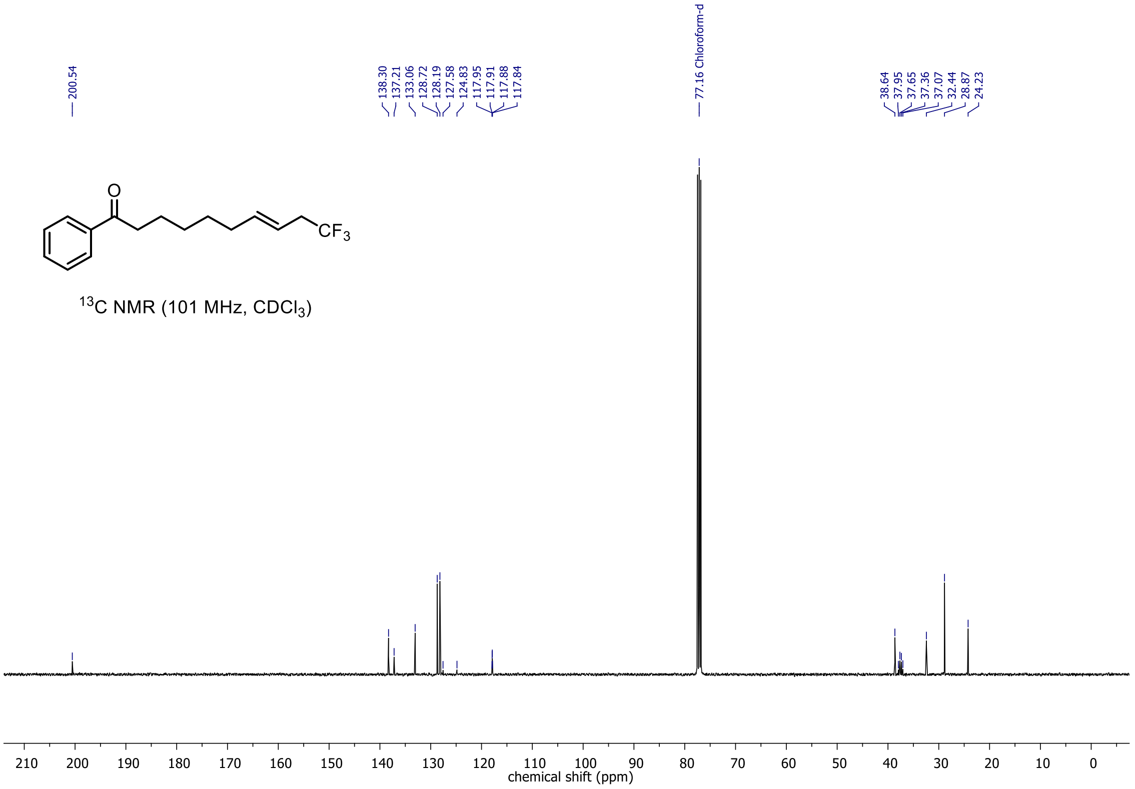

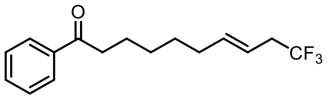

^{19}F NMR (376 MHz, $CDCl_3$)

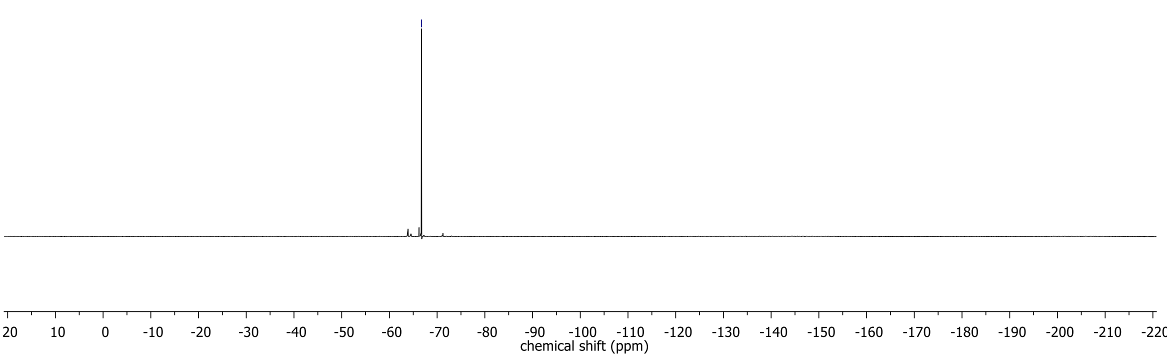

(*E*)-1,16-Diphenylhexadec-8-ene-1,16-dione

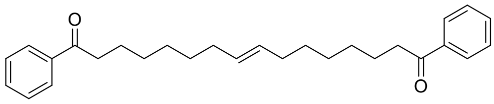

The compound was prepared following a procedure reported by Denard *et al.*[8]

In a glass vial 1-phenylnon-8-en-1-one (216 mg, 1.00 mmol, 1.00 equiv.) was dissolved in DCM (2 mL, 0.5 M) and Hoveyda-Grubbs 2nd generation catalyst (18.8 mg, 0.03 mmol, 3.00 mol%) was added. The resulting mixture was stirred for 16 h and then directly purified by flash column chromatography (0 – 100% EtOAc in heptanes), affording the title compound (292 mg, 0.78 mmol, 78%) as a colorless solid.

^{1}H NMR (600 MHz, $CDCl_3$): δ 7.99 – 7.91 (m, 4H), 7.58 –7.52 (m, 2H), 7.49 – 7.43 (m, 4H), 5.40 – 5.35 (m, 2H), 2.96 (t, *J* = 7.5 Hz, 4H), 2.00 – 1.92 (m, 4H), 1.76 – 1.69 (m, 4H), 1.41 – 1.30 (m, 12H).

^{13}C NMR (161 MHz, $CDCl_3$): δ 200.7 (2C), 137.3 (2C), 133.0 (2C), 130.5 (2C), 128.7 (4C), 128.2 (4C), 38.8 (2C), 32.7 (2C), 29.6 (2C), 29.4 (2C), 29.1 (2C), 24.5 (2C).

IR (neat) v_{max}: 2917, 2849, 1681, 1446, 1407, 1219, 1015, 963, 731, 686, 567.

HRMS (ESI$^+$): exact mass calculated for $[M+Na]^+$ $(C_{28}H_{36}O_2Na)^+$ requires *m/z* 427.2599, found *m/z* 427.2608.

(*E*)-1,16-Diphenylhexadec-8-ene-1,16-dione

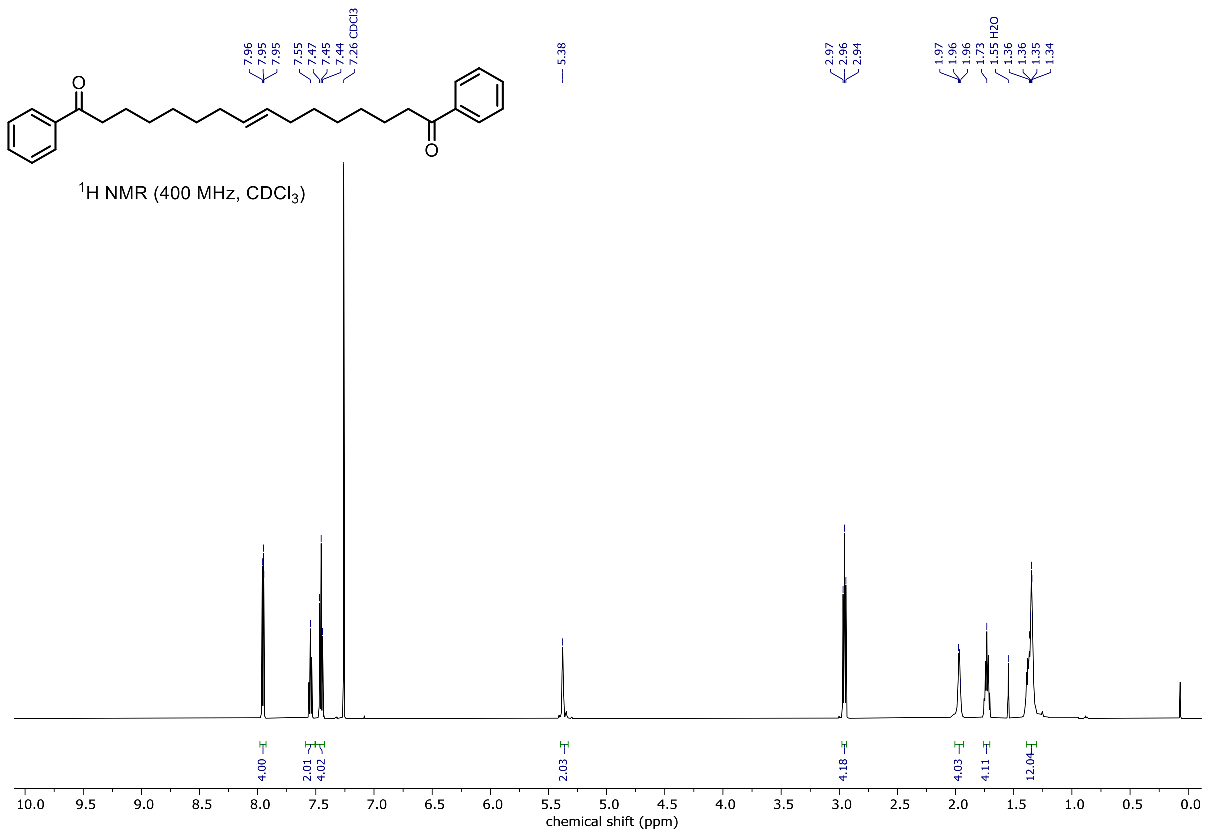

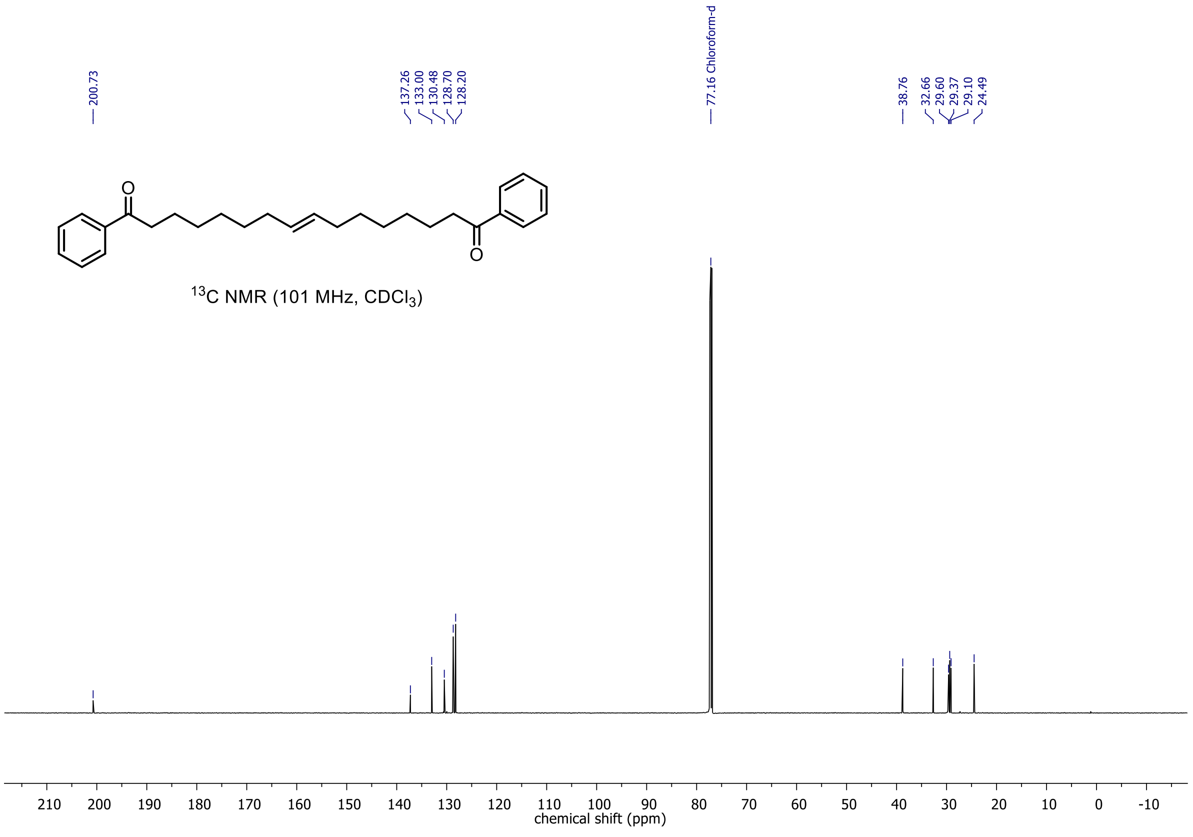

(Z)-1-Phenyloctadec-9-en-1-one

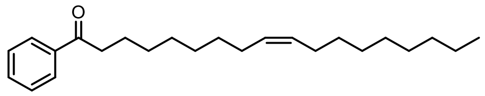

The title compound was prepared following general procedure C using *N*-methoxy-*N*-methyloleamide (651 mg, 2.00 mmol, 1.00 equiv.) and a solution of phenylmagnesium bromide (1.50 equiv.). Purification by flash column chromatography (0 – 20% EtOAc in heptanes) afforded the title compound (615 mg, 1.80 mmol, 89%) as a colorless oil.

^{1}H NMR (400 MHz, $CDCl_3$): δ 7.99 – 7.93 (m, 2H), 7.57 – 7.52 (m, 1H), 7.49 – 7.43 (m, 2H), 5.38 – 5.31 (m, 2H), 2.99 – 2.92 (m, 2H), 2.06 – 1.96 (m, 4H), 1.78 – 1.69 (m, 2H), 1.43 – 1.22 (m, 20H), 0.88 (t, *J* = 6.9 Hz, 3H).

All analytical data were in good agreement with those reported in literature.[10]

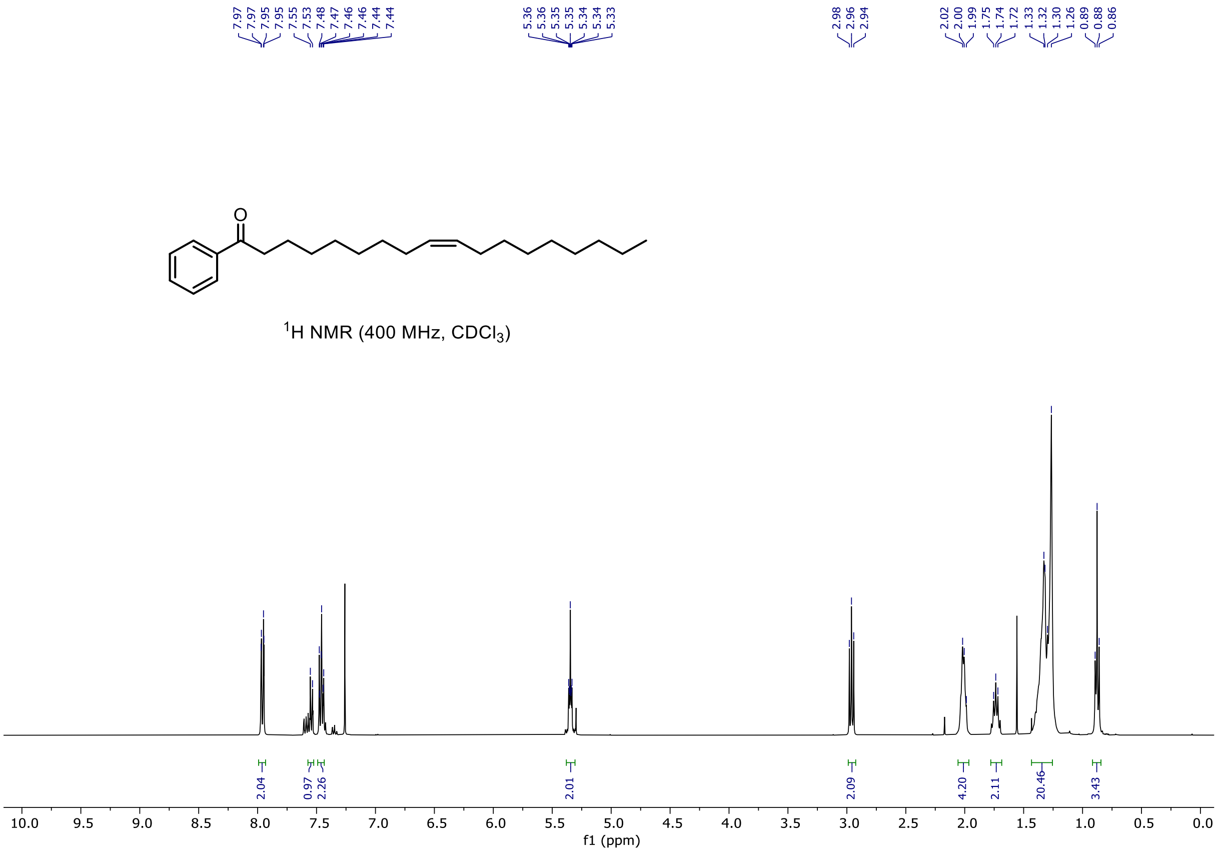

1,2-Diphenyldec-9-en-1-one

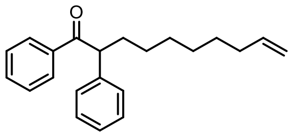

In a 25 mL round-bottom flask NaH (60% in paraffine, 80 mg, 2.00 mmol, 1.00 equiv.) was suspended in THF (7 mL). The suspension was cooled to 0 °C, and benzyl phenyl ketone (393 mg, 2.00 mmol, 1.00 equiv.) was added dropwise over 10 min. The reaction mixture was stirred for 30 min, after which 8-bromo-1-octene (382 mg, 2.00 mmol, 1.00 equiv.) was added. The ice bath was removed, and the reaction progress was monitored by TLC. Upon completion (approx. 16 h), Et_2O was added (10 mL), the biphasic mixture was separated and the organic phase was washed with water (2 × 10 mL). The ethereal solution was dried over anhydrous $MgSO_4$, filtered, and concentrated under reduced pressure. The crude residue was purified by flash column chromatography (0 – 10% EtOAc in heptanes), yielding the title compound as a colorless liquid (163 mg, 0.53 mmol, 27%).

^{1}H NMR (400 MHz, $CDCl_3$): δ 8.00 – 7.92 (m, 2H), 7.52 – 7.45 (m, 1H), 7.43 – 7.35 (m, 2H), 7.33 – 7.25 (m, 4H), 7.22 – 7.17 (m, 1H), 5.88 – 5.69 (m, 1H), 5.02 – 4.84 (m, 2H), 4.53 (t, *J* = 7.3 Hz, 1H), 2.25 – 2.11 (m, 1H), 2.06 – 1.92 (m, 2H), 1.90 – 1.72 (m, 1H), 1.44 – 1.17 (m, 8H).

^{13}C NMR (101 MHz, $CDCl_3$): δ 200.3, 134.0, 139.3, 137.2, 132.9, 129.0 (2C), 128.8 (2C), 128.6 (2C), 128.4 (2C), 127.1, 114.3, 53.8, 34.2, 33.9, 29.6, 29.1, 29.0, 27.8.

IR (neat) v_{max}: 2925, 2854, 1680, 1447, 1210, 1176, 909, 754, 696, 581.

HRMS (ESI$^+$): exact mass calculated for $[M+Na]^+$ $(C_{22}H_{26}ONa)^+$ requires *m/z* 329.1876, found *m/z* 329.1882.

1,2-Diphenyldec-9-en-1-one

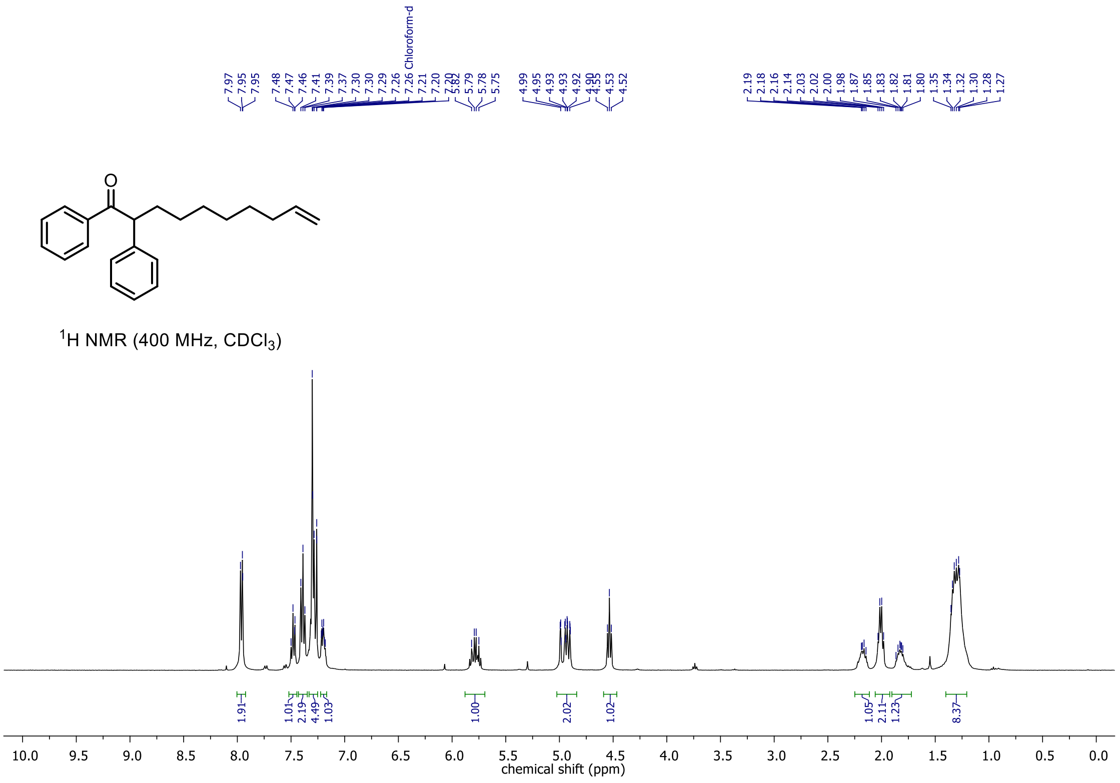

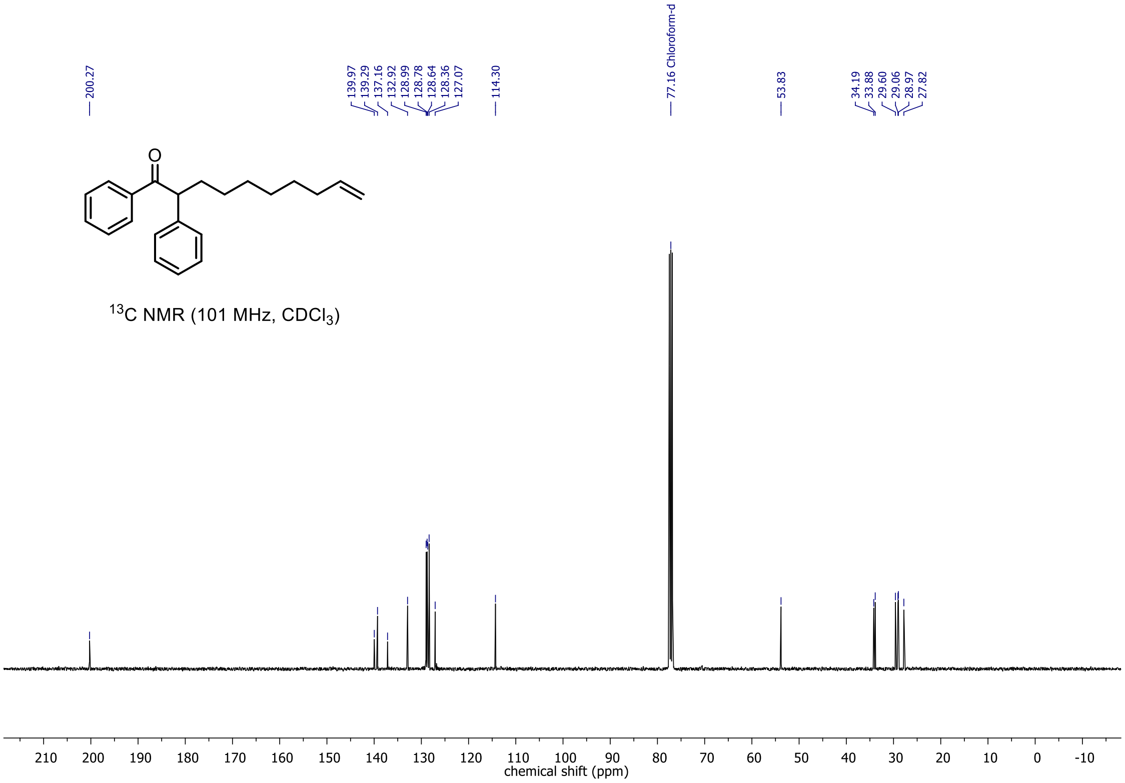

1-(*o*-Tolyl)non-8-en-1-one

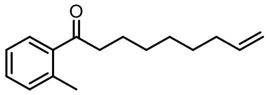

The title compound was prepared following general procedure C using *N*-methoxy-*N*,2-dimethylbenzamide (358 mg, 2.00 mmol, 1.00 equiv.) and a solution of 7-octenylmagnesium bromide (1.50 equiv.). Purification by flash column chromatography (0 – 20% EtOAc in heptanes) afforded the title compound (184 mg, 0.80 mmol, 40%) as a colorless liquid.

^{1}H NMR (400 MHz, $CDCl_3$): δ 7.62 – 7.59 (m, 1H), 7.40 – 7.32 (m, 1H), 7.25 – 7.22 (m, 2H), 5.80 (ddt, *J* = 16.9, 10.2, 6.7 Hz, 1H), 5.02 – 4.88 (m, 2H), 2.88 (t, *J* = 7.4 Hz, 2H), 2.48 (s, 3H), 2.08 – 2.00 (m, 2H), 1.77 – 1.65 (m, 2H), 1.41 – 1.30 (m, 6H).

^{13}C NMR (101 MHz, $CDCl_3$): δ 205.1, 139.2, 138.6, 137.9, 132.0, 131.1, 128.4, 125.8, 114.4, 41.8, 33.9, 29.3, 29.1, 28.9, 24.5, 21.3.

IR (neat) v_{max}: 3073, 2926, 2854, 1684, 1640, 1600, 1571, 1485, 1381, 1285, 1214, 908, 753 ,654, 612.

HRMS (ESI$^+$): exact mass calculated for $[M+Na]^+$ $(C_{16}H_{22}ONa)^+$ requires *m/z* 253.1563, found *m/z* 253.1558.

1-(*o*-Tolyl)non-8-en-1-one

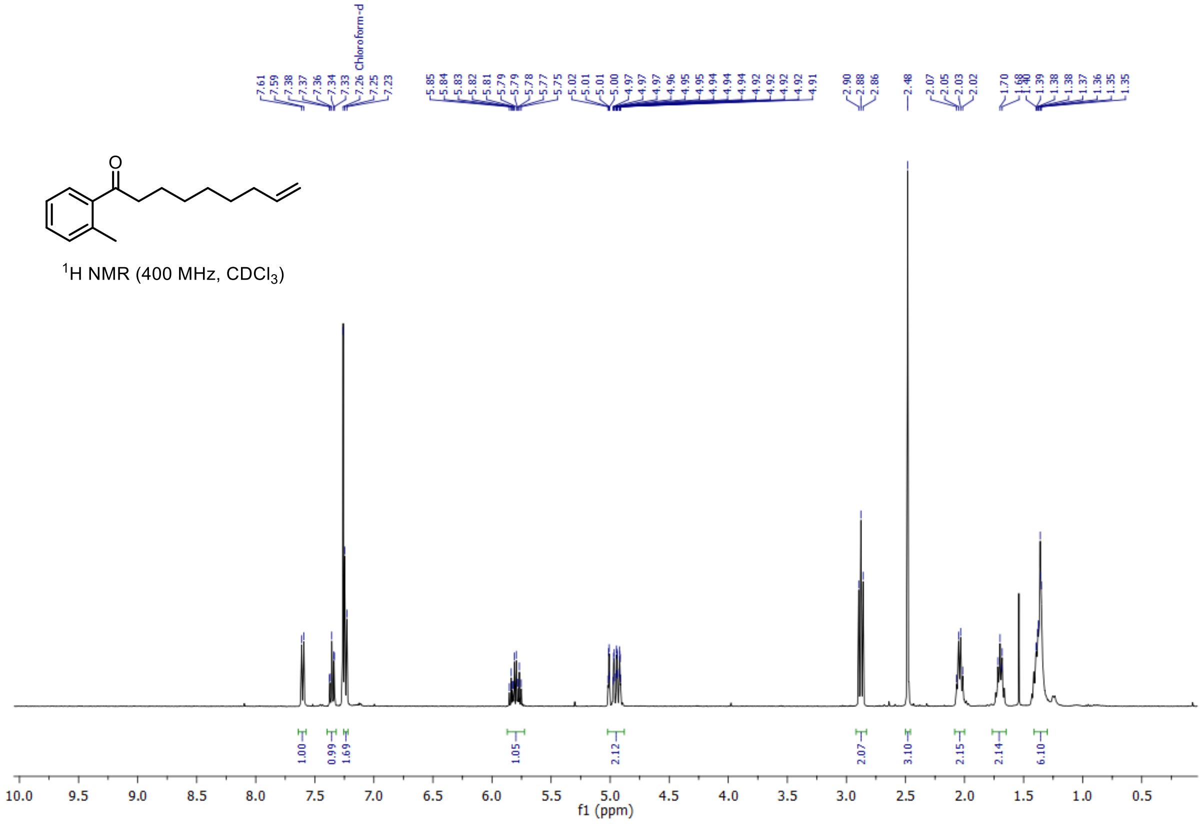

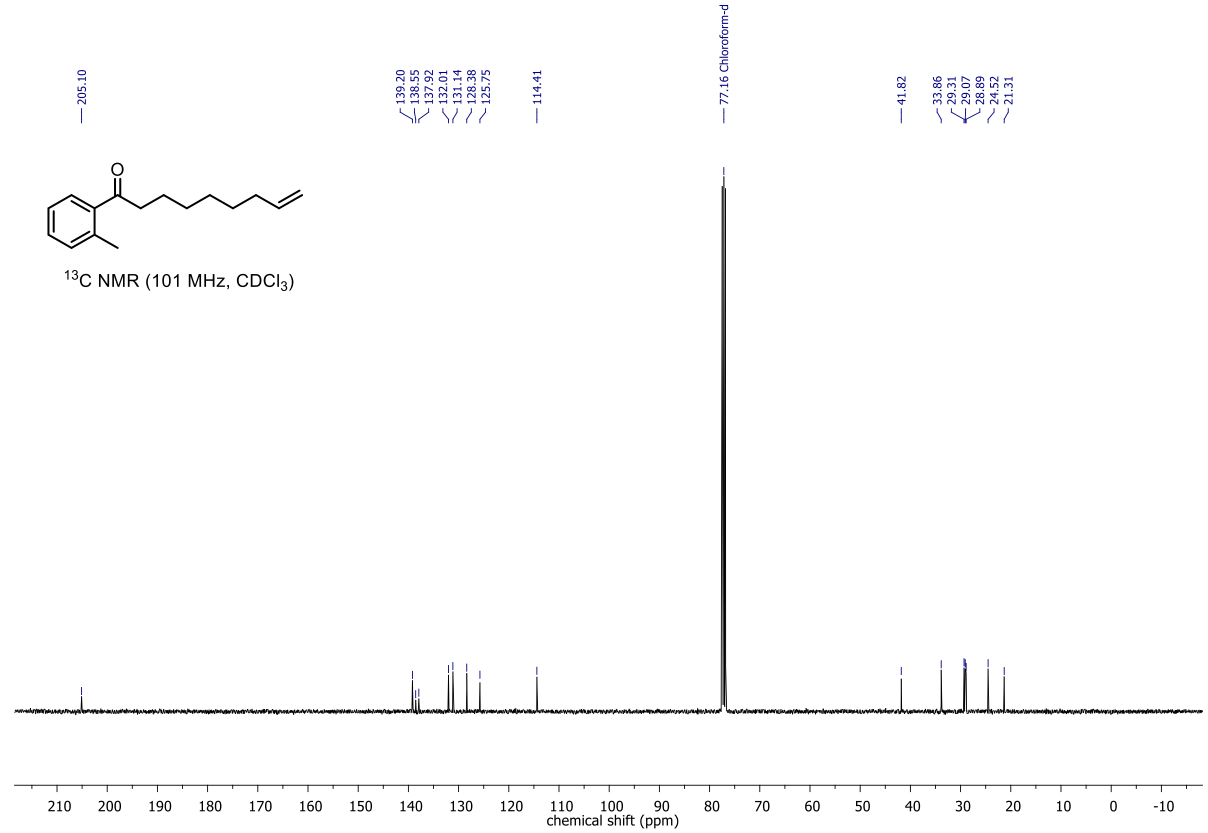

1-(4-(*tert*-Butyl)phenyl)non-8-en-1-one

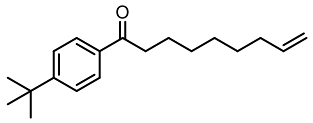

The title compound was prepared following general procedure C using the Weinreb amide 4-(*tert*-Butyl)-*N*-methoxy-*N*-methylbenzamide (8.26 g, 50.00 mmol, 1.00 equiv.) and a solution of 7-octenylmagnesium bromide (1.50 equiv.). Purification by flash column chromatography (0 – 20% EtOAc in heptanes) afforded the title compound (304 mg, 1.12 mmol, 82%) as a colorless liquid.

^{1}H NMR (400 MHz, CDCl$_3$): δ 7.90 (d, *J* = 8.6 Hz, 2H), 7.47 (d, *J* = 8.6 Hz, 2H), 5.81 (ddt, *J* = 16.9, 10.2, 6.7 Hz, 1H), 5.04 – 4.83 (m, 2H), 2.97 – 2.89 (m, 2H), 2.10 – 1.97 (m, 2H), 1.78 – 1.67 (m, 2H), 1.43 – 1.32 (m, 15H).

^{13}C NMR (101 MHz, CDCl$_3$): δ 200.4, 156.7, 139.2, 134.7, 128.2 (2C), 125.6 (2C), 114.4, 38.6, 35.2, 33.9, 31.2 (3C), 29.4, 29.1, 28.9, 24.6.

IR (neat) ν_{max}: 2962, 2927, 2855, 1681, 1605, 1463, 1406, 1363, 1268, 1220, 1190, 1107, 993, 908, 841, 824.

HRMS (ESI$^+$): exact mass calculated for $[M+Na]^+$ $(C_{19}H_{28}ONa)^+$ requires *m/z* 295.2032, found *m/z* 295.2027.

1-(4-(*tert*-Butyl)phenyl)non-8-en-1-one

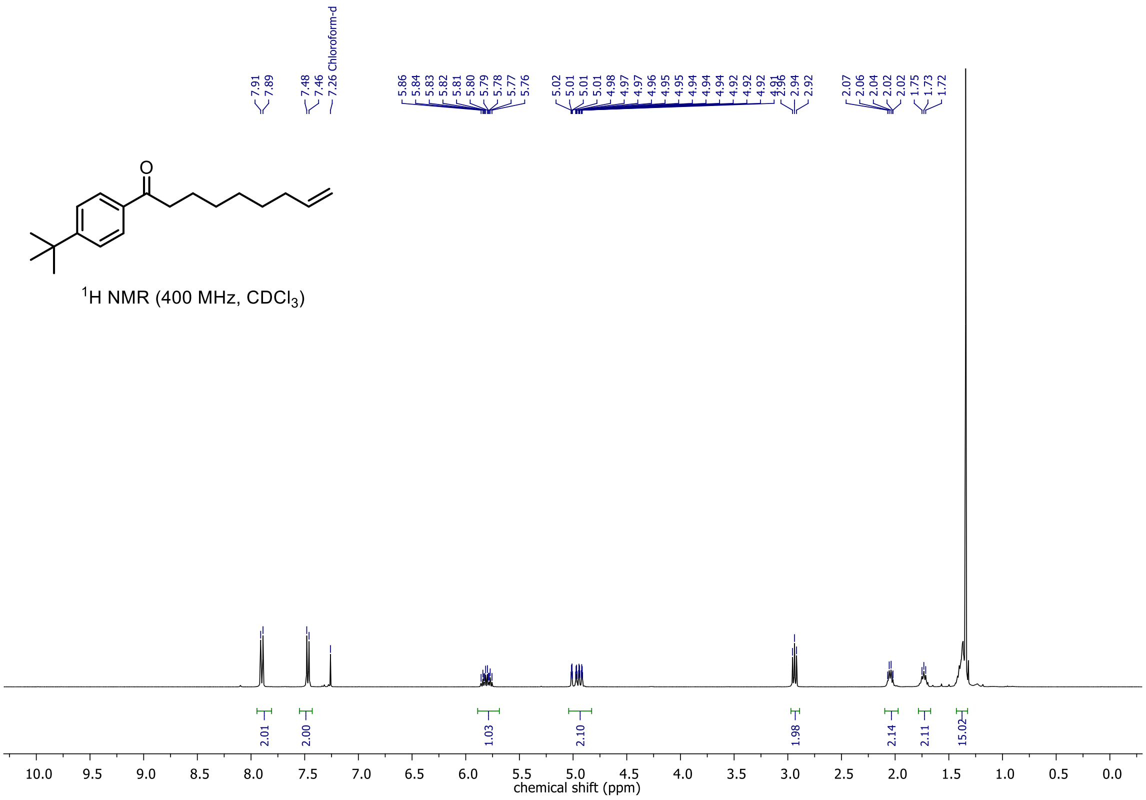

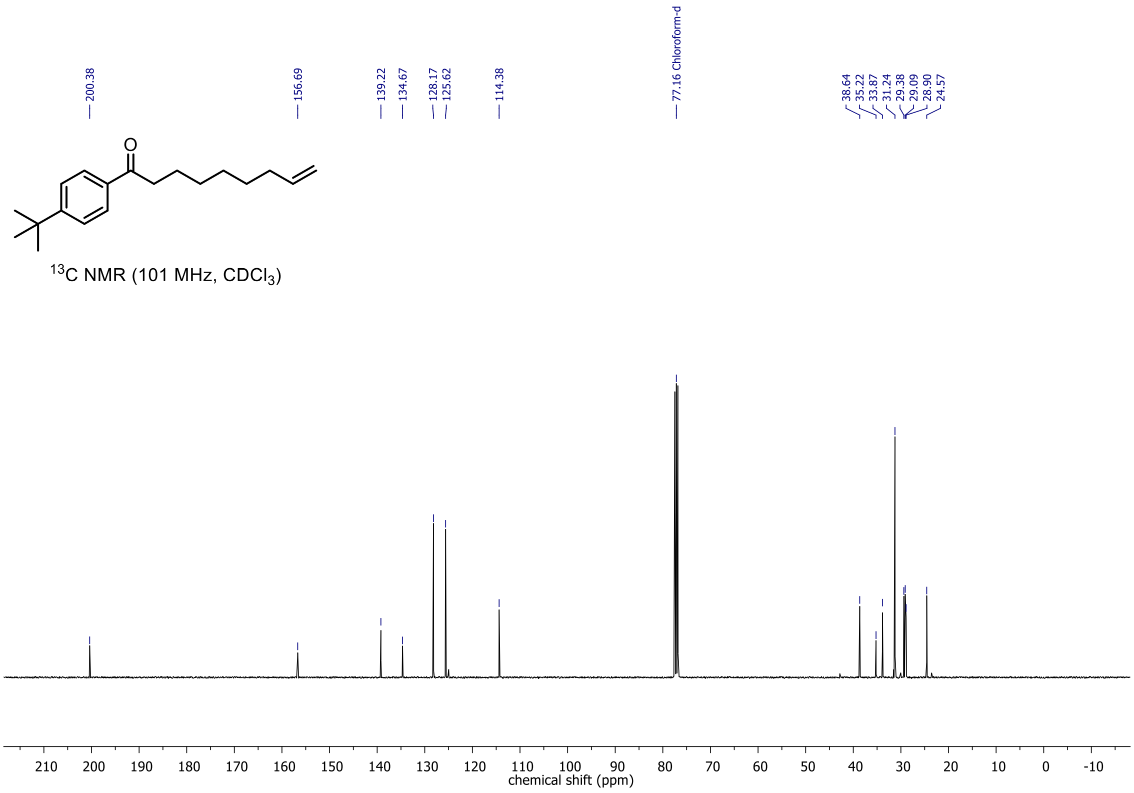

1-(3-Chlorophenyl)non-8-en-1-ol

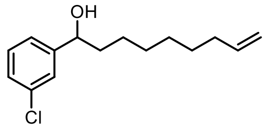

The title compound was prepared following general procedure D using 3-chlorobenzaldehyde (562 mg, 4.00 mmol, 1.00 equiv.) and a solution of 7-octenylmagnesium bromide (1.50 equiv.). Purification by flash column chromatography (0 – 60% EtOAc in heptanes) afforded the title compound (701 mg, 2.77 mmol, 69%) as colorless liquid.

^{1}H NMR (400 MHz, $CDCl_3$): δ 7.35 (m, 1H), 7.31 – 7.24 (m, 2H), 7.21 (m, 1H), 5.80 (ddt, *J* = 16.9, 10.2, 6.7 Hz, 1H), 5.10 – 4.88 (m, 2H), 4.65 (t, *J* = 6.4 Hz, 1H), 2.03 (m, 2H), 1.86 – 1.63 (m, 3H), 1.44 – 1.26 (m, 8H).

^{13}C NMR (101 MHz, $CDCl_3$): δ 147.2, 139.2, 134.5, 129.8, 127.7, 126.2, 124.2, 114.4, 74.2, 39.3, 33.9, 29.5, 29.1, 29.0, 25.8.

IR (neat) v_{max}: 3352, 2927, 2855, 1597, 1465, 1432, 1199, 997, 909, 785, 732, 697.

HRMS (ESI$^-$): exact mass calculated for $[M-H]^-$ $(C_{15}H_{20}ClO)^-$ requires *m/z* 251.1208, found *m/z* 251.1210.

1-(3-Chlorophenyl)non-8-en-1-ol

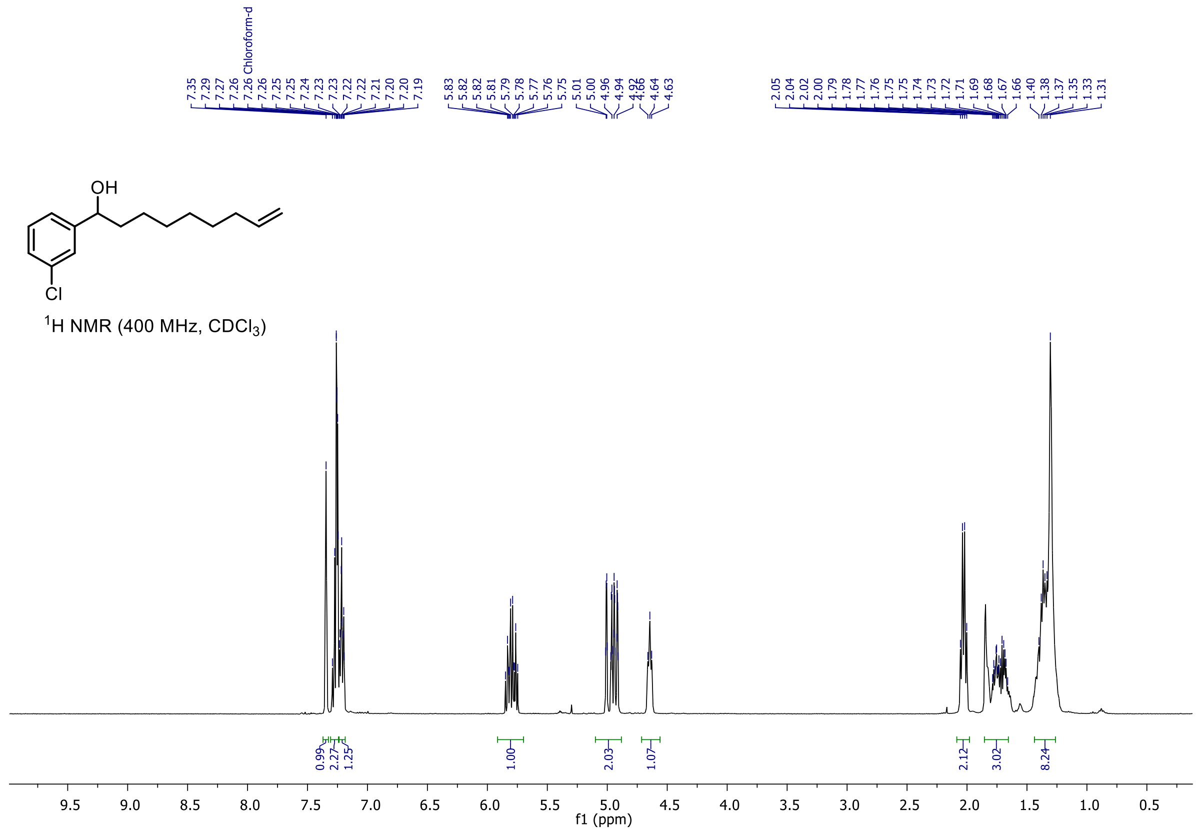

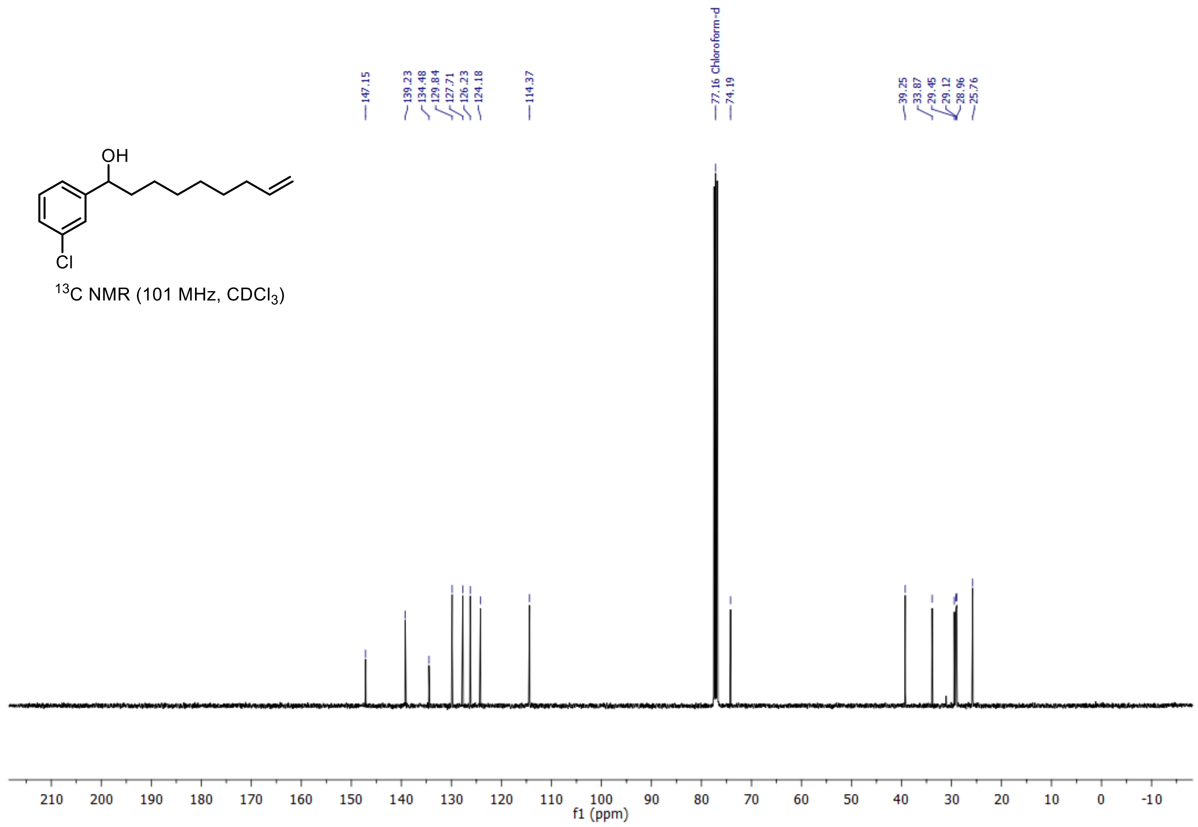

1-(3-Chlorophenyl)non-8-en-1-one

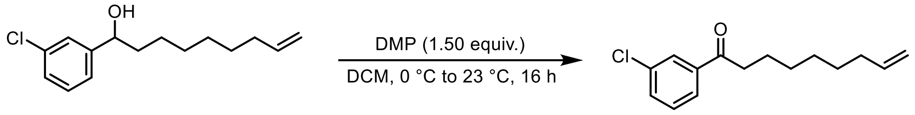

The compound was prepared following a procedure reported by He *et al.*[11]

1-(3-Chlorophenyl)non-8-en-1-ol (253 mg, 1.00 mmol, 1.00 equiv.) was dissolved in DCM (2 mL) and the mixture was cooled to 0 °C. Dess-Martin periodinane (636 mg, 1.50 mmol, 1.50 equiv.) was added and the resulting suspension was stirred at 0 °C for 3 h. The reaction mixture was diluted with DCM (10 mL) and saturated aqueous $NaHCO_3$ solution (10 mL) was added. The organic phase was separated, and the aqueous layer was extracted once with DCM (10 mL). The combined organic phases were washed with brine (10 mL), dried over anhydrous sodium sulfate, the dried solution was filtered, and the filtrate was concentrated under reduced pressure. The crude residue was purified by flash column chromatography (0 – 20% EtOAc in heptanes), affording the title compound as a colorless liquid (156 mg, 0.62 mmol, 62%).

^{1}H NMR (400 MHz, $CDCl_3$): δ 7.95 – 7.90 (m, 1H), 7.86 – 7.80 (m, 1H), 7.56 – 7.50 (m, 1H), 7.45 – 7.37 (m, 1H), 5.81 (ddt, *J* = 16.9, 10.2, 6.7 Hz, 1H), 5.05 – 4.90 (m, 2H), 2.94 (t, *J* = 7.4 Hz, 2H), 2.10 – 2.01 (m, 2H), 1.78 – 1.68 (m, 2H), 1.43 – 1.34 (m, 6H).

^{13}C NMR (101 MHz, $CDCl_3$): δ 199.3, 139.2, 138.8, 135.05, 133.0, 130.1, 128.3, 126.3, 114.5, 38.8, 33.9, 29.3, 29.1, 29.0, 24.3.

IR (neat) ν_{max}: 3074, 2927, 2854, 2360, 1639, 1571, 1419, 1365, 1209, 107, 907, 711, 567.

HRMS (ESI$^+$): exact mass calculated for $[M+Na]^+$ $(C_{15}H_{19}OClNa)^+$ requires *m/z* 273.1017, found *m/z* 273.1007.

1-(3-Chlorophenyl)non-8-en-1-one

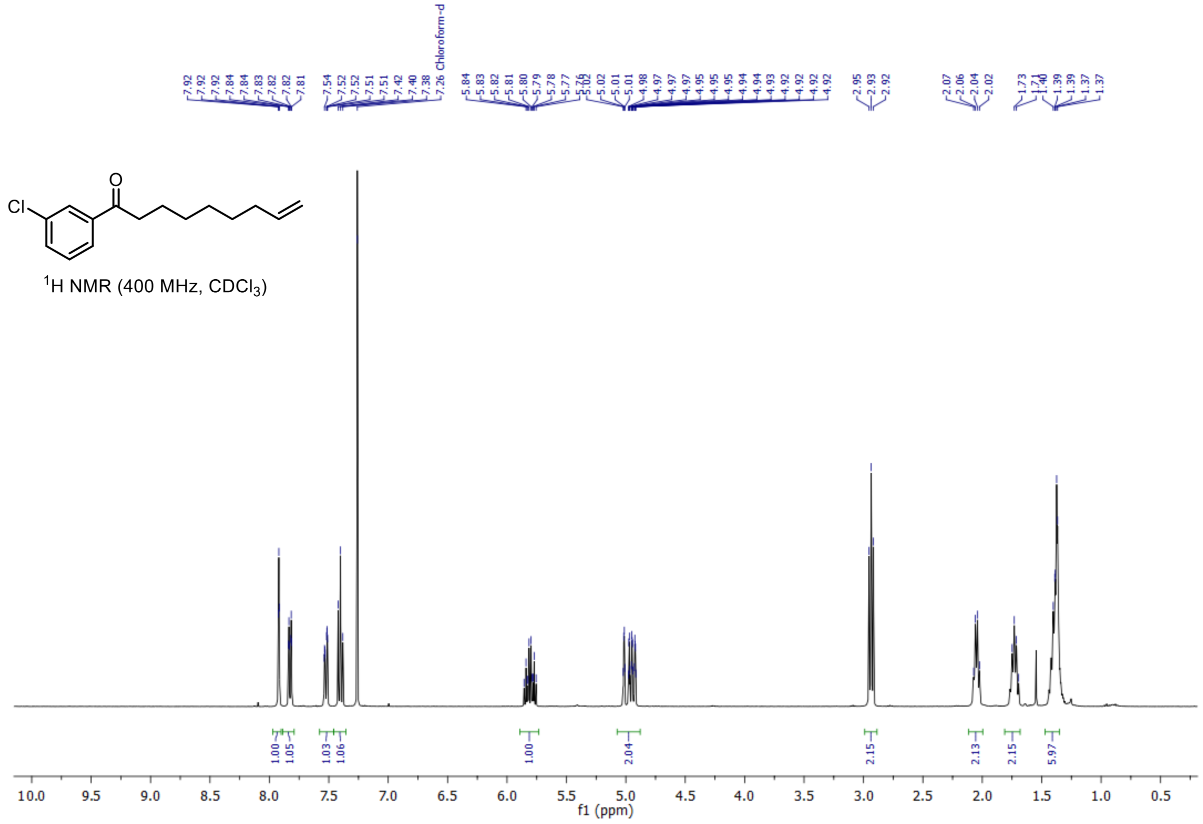

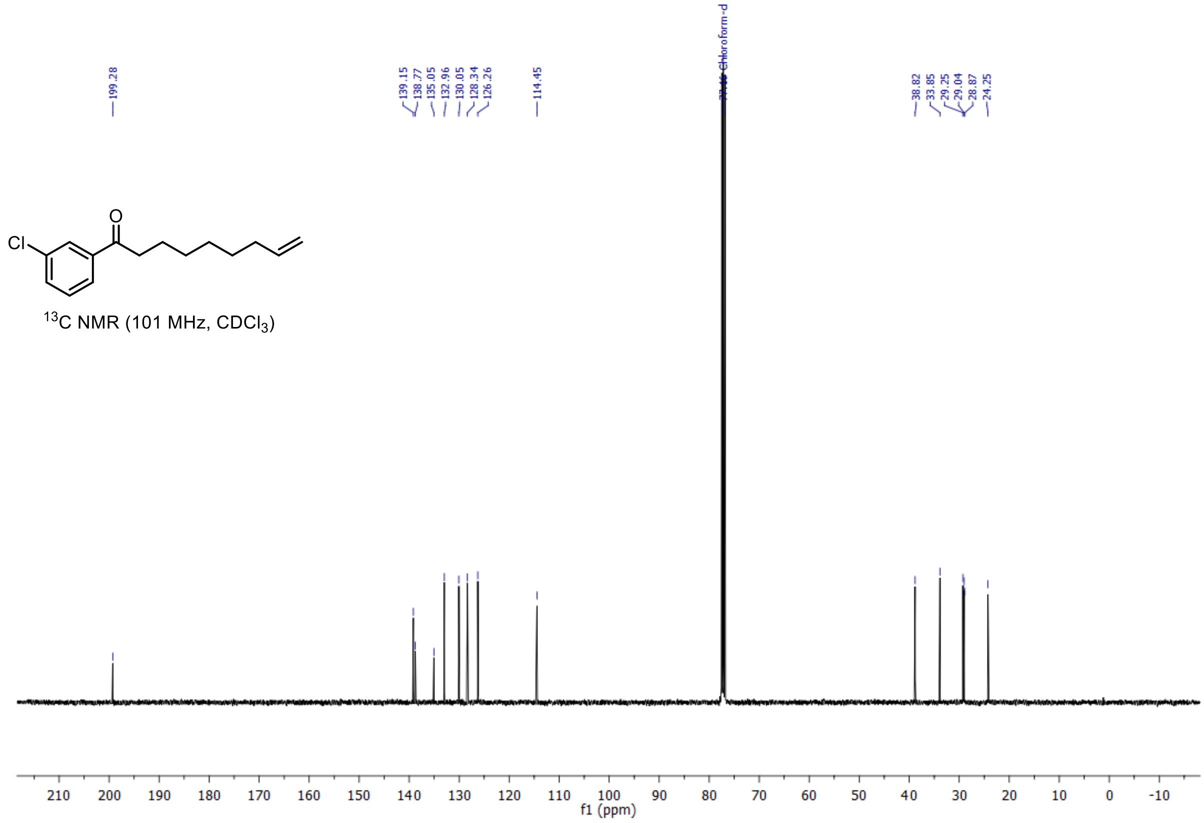

1-(4-(Trifluoromethyl)phenyl)non-8-en-1-one

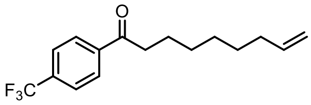

7-octenylmagnesium bromide solution was prepared following general procedure C using 8-bromo-1-octene (3.36 mL, 20.00 mmol, 1.00 equiv.) and magnesium (972 mg, 40.00 mmol, 2.00 equiv.).

The title compound was prepared following general procedure C using Weinreb amide *N*-methoxy-*N*-methyl-4-(trifluoromethyl)benzamide (491 mg, 2.00 mmol, 1.00 equiv.) and a solution of 7-octenylmagnesium bromide (1.50 equiv.). Purification by flash column chromatography (0 – 10% EtOAc in heptanes) afforded the title compound as a colorless oil (280 mg, 0.99 mmol, 49%).

^{1}H NMR (700 MHz, $CDCl_3$) δ 8.05 (d, *J* = 8.1 Hz, 2H), 7.72 (d, *J* = 8.2 Hz, 2H), 5.80 (ddt, *J* = 16.9, 10.2, 6.7 Hz, 1H), 4.99 (ddd, *J* = 17.1, 3.6, 1.6 Hz, 1H), 4.93 (ddt, *J* = 10.2, 2.2, 1.2 Hz, 1H), 2.98 (t, *J* = 7.4 Hz, 2H), 2.08 – 2.02 (m, 2H), 1.78 – 1.72 (m, 2H), 1.43 – 1.34 (m, 6H).

^{13}C NMR (176 MHz, $CDCl_3$) δ 199.6 (C), 139.9 (C), 139.1 (CH), 134.4 (q, *J* = 32.7 Hz, C), 128.5 (2CH), 125.8 (q, *J* = 3.7 Hz, 2CH), 123.8 (q, *J* = 272.7 Hz, C), 114.5 (CH_2), 39.0 (CH_2), 33.8 (CH_2), 29.2 (CH_2), 29.0 (CH_2), 28.9 (CH_2), 24.2 (CH_2).

^{19}F NMR (659 MHz, $CDCl_3$) δ -63.10 (3F).

IR (neat) ν_{max}: 3077, 2929, 2856, 1693, 1410, 1324, 1169, 1130, 1109, 1066, 1015.

HRMS (EI$^+$): exact mass calculated for $[M–C_8H_{15}^{\cdot}]^+$ $(C_8H_4F_3O)^+$ requires *m*/*z* 173.0209, found *m*/*z* 173.0219.

1-(4-(Trifluoromethyl)phenyl)non-8-en-1-one

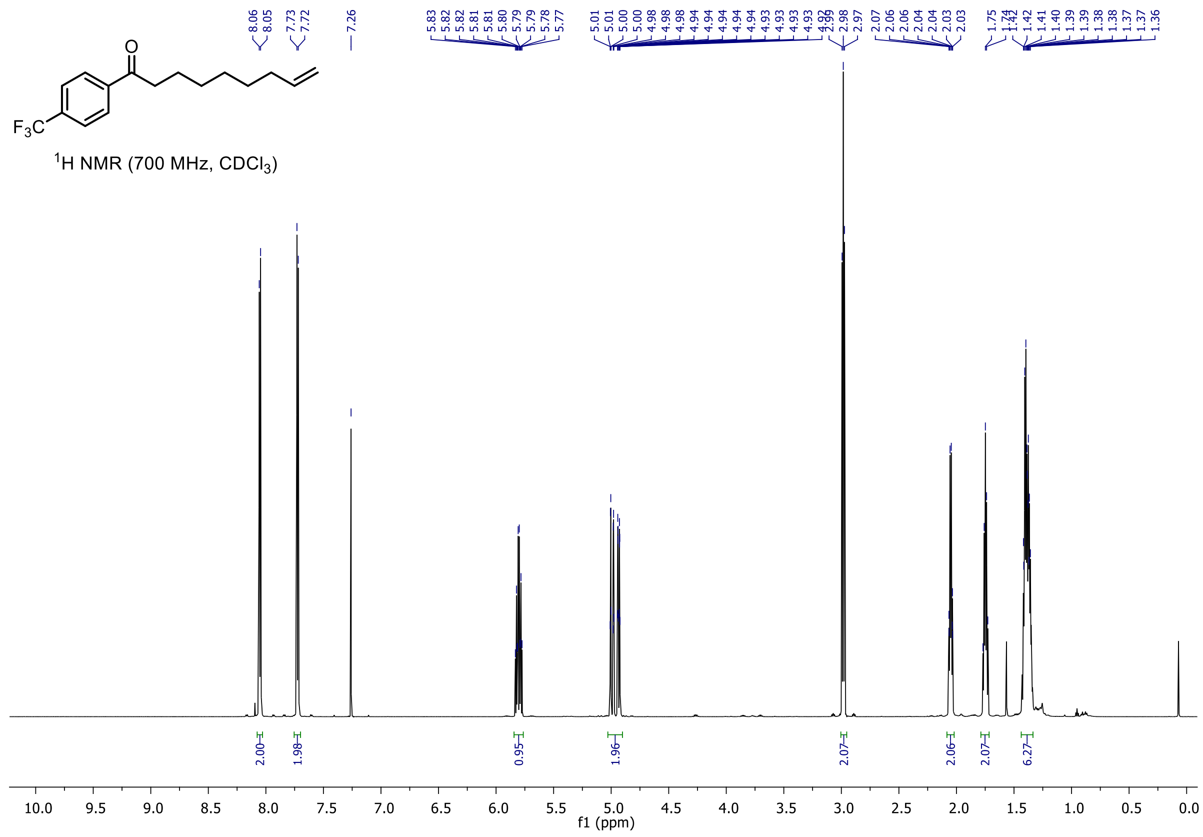

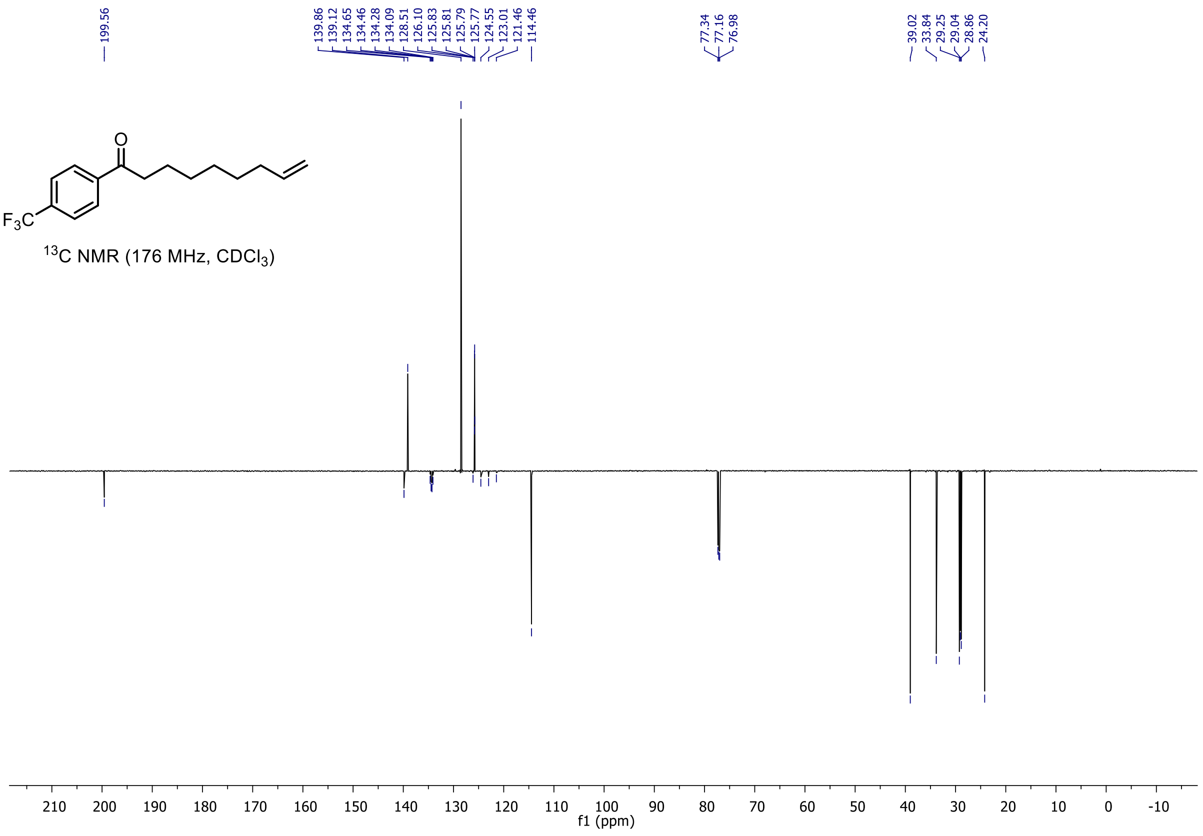

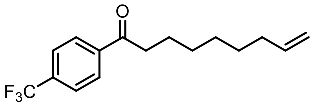

^{19}F NMR (659 MHz, $CDCl_3$)

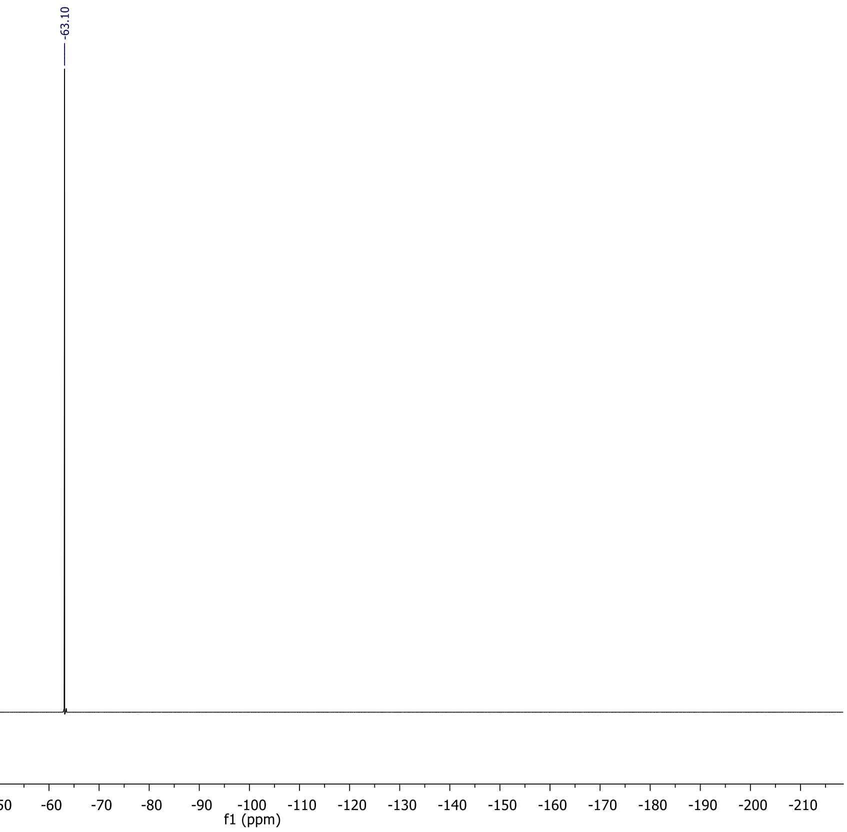

1-(4-(Trifluoromethoxy)phenyl)non-8-en-1-one

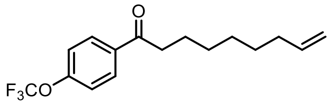

7-octenylmagnesium bromide solution was prepared following general procedure C using 8-bromo-1-octene (3.36 mL, 20.00 mmol, 1.00 equiv.) and magnesium (972 mg, 40.00 mmol, 2.00 equiv.).

The title compound was prepared following general procedure C using Weinreb amide *N*-methoxy-*N*-methyl-4-(trifluoromethoxy)benzamide (525 mg, 2.00 mmol, 1.00 equiv.) and a solution of 7-octenylmagnesium bromide (1.50 equiv.). Purification by flash column chromatography (0 – 10% EtOAc in heptanes) afforded the title compound as a colorless oil (485 mg, 1.61 mmol, 81%).

^{1}H NMR (600 MHz, $CDCl_3$) δ 8.03 – 7.97 (m, 2H), 7.28 (d, *J* = 8.1 Hz, 2H), 5.80 (ddt, *J* = 16.9, 10.2, 6.7 Hz, 1H), 5.03 – 4.96 (m, 1H), 4.96 – 4.90 (m, 1H), 2.94 (t, *J* = 7.4 Hz, 2H), 2.05 (app q, *J* = 6.9 Hz, 2H), 1.77 – 1.70 (m, 2H), 1.44 – 1.31 (m, 6H).

^{13}C NMR (151 MHz, $CDCl_3$) δ 199.0 (C), 152.6 (q*, *J* = 2.1 Hz, C), 139.1 (CH), 135.5 (C), 130.2 (2CH), 120.5 (2CH), 120.4 (q, *J* = 259.7 Hz, C), 114.4 (CH_2), 38.8 (CH_2), 33.9 (CH_2), 29.3 (CH_2), 29.1 (CH_2), 28.9 (CH_2), 24.3 (CH_2).

^{19}F NMR (565 MHz, $CDCl_3$) δ -57.62 (3F).

*Only the two more intense signals of 1:**3:3**:1 quartet could be observed.

IR (neat) ν_{max}: 3077, 2929, 2856, 1688, 1602, 1251, 1206, 1161, 994, 910, 809.

HRMS (EI^{+}): exact mass calculated for $[M]^{\cdot+}$ $(C_{16}H_{19}F_3O_2)^{\cdot+}$ requires *m*/*z* 300.1332, found *m*/*z* 300.1323.

1-(4-(Trifluoromethoxy)phenyl)non-8-en-1-one

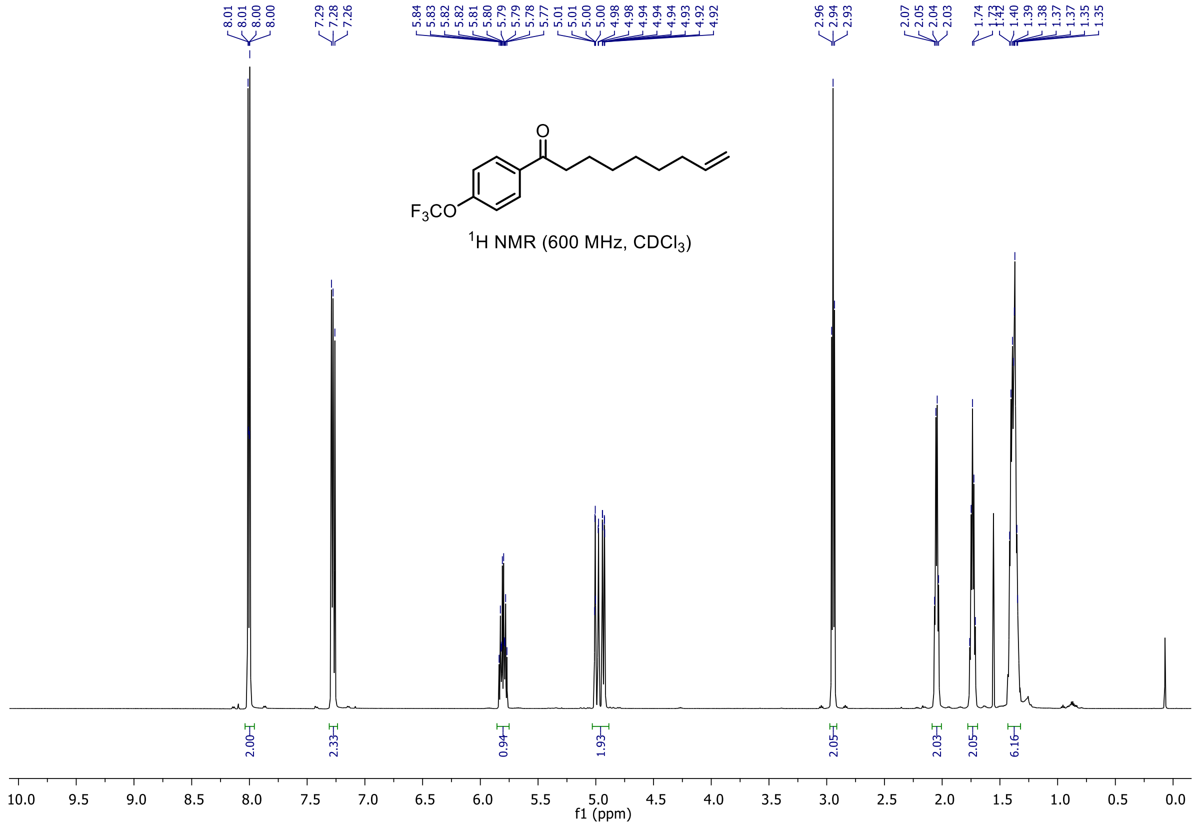

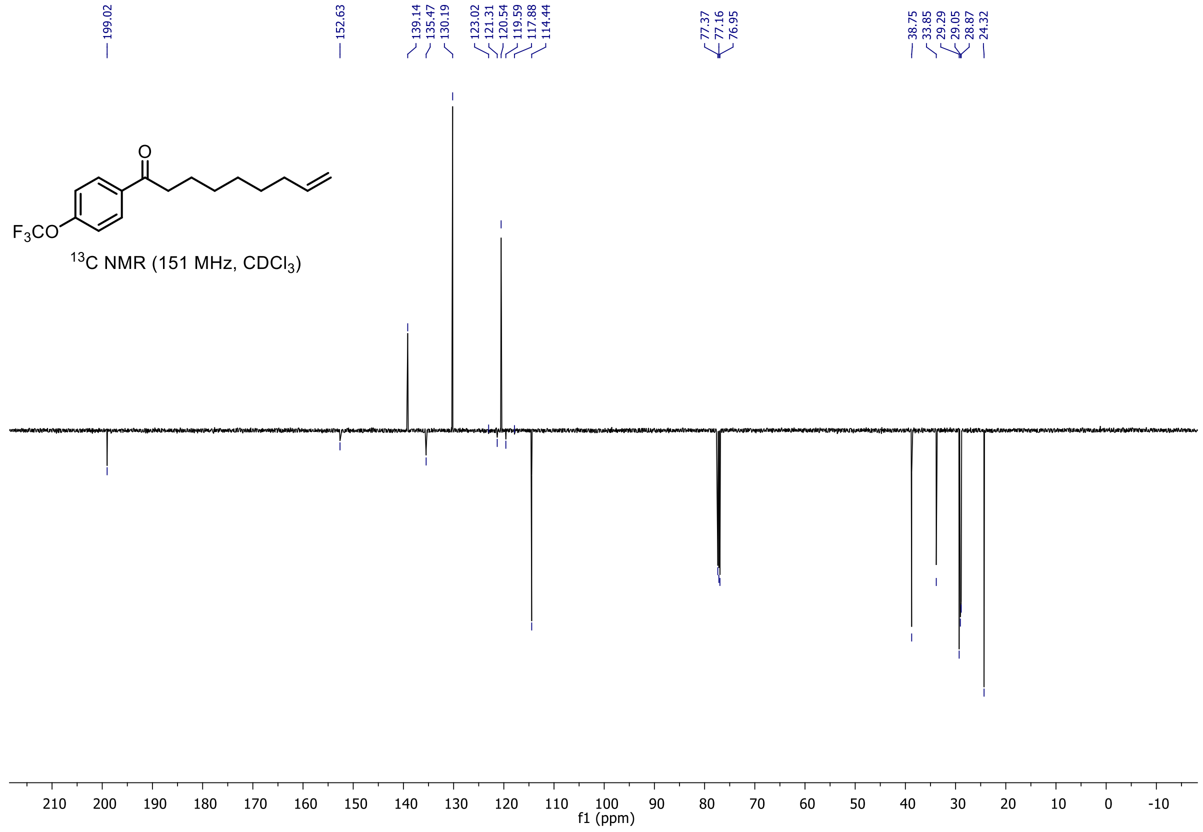

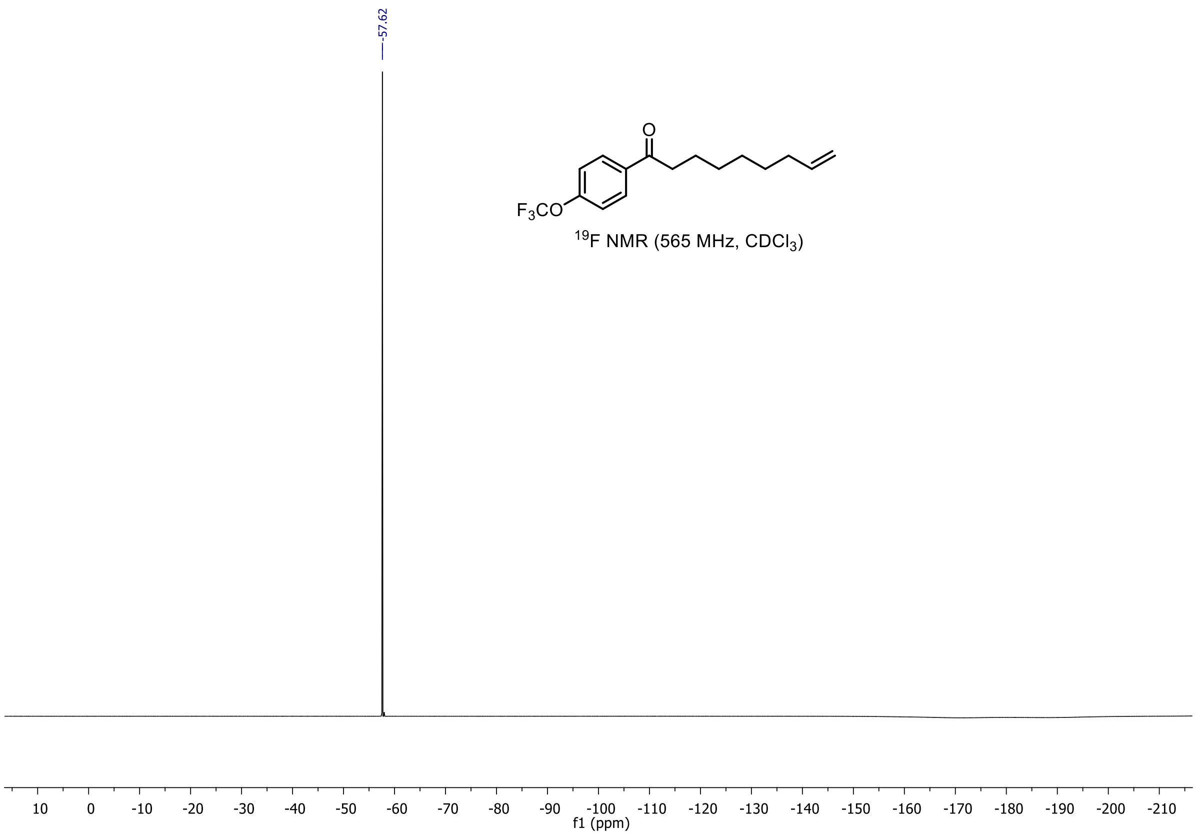
-57.62
F3CO
O
19F NMR (565 MHz, CDCl3)
10 0 -10 -20 -30 -40 -50 -60 -70 -80 -90 -100 -110 -120 -130 -140 -150 -160 -170 -180 -190 -200 -210
f1 (ppm)

Methyl 4-(non-8-enoyl)benzoate

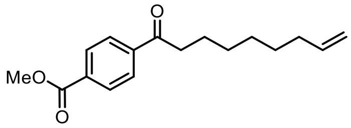

7-octenylmagnesium bromide solution was prepared following general procedure C using 8-bromo-1-octene (3.36 mL, 20.00 mmol, 1.00 equiv.) and magnesium (972 mg, 40.00 mmol, 2.00 equiv.).

The title compound was prepared following general procedure C using Weinreb amide methyl 4-(methoxy(methyl)carbamoyl)benzoate (470 mg, 2.00 mmol, 1.00 equiv.) and a solution of 7-octenylmagnesium bromide (1.50 equiv.). Purification by flash column chromatography (0 – 10% EtOAc in heptanes) afforded the title compound as a colorless solid (220 mg, 0.80 mmol, 40%).

^{1}H NMR (400 MHz, $CDCl_3$) δ 8.15 – 8.08 (m, 2H), 8.02 – 7.97 (m, 2H), 5.80 (ddt, *J* = 16.9, 10.2, 6.7 Hz, 1H), 4.99 (ddd, *J* = 17.1, 3.7, 1.6 Hz, 1H), 4.93 (ddt, *J* = 10.2, 2.2, 1.2 Hz, 1H), 3.95 (s, 3H), 2.98 (t, *J* = 7.4 Hz, 2H), 2.10 – 1.99 (m, 2H), 1.81 – 1.69 (m, 2H), 1.45 – 1.30 (m, 6H).

^{13}C NMR (101 MHz, $CDCl_3$) δ 200.1 (C), 166.4 (C), 140.4 (C), 139.1 (CH), 133.8 (C), 130.0 (2CH), 128.1 (2CH), 114.4 (CH_2), 52.6 (CH_3), 39.1 (CH_2), 33.8 (CH_2), 29.3 (CH_2), 29.1 (CH_2), 28.9 (CH_2), 24.2 (CH_2).

IR (neat) ν_{max}: 2927, 2849, 1722, 1677, 1437, 1283, 1192, 1111, 763, 738, 700.

HRMS (EI^{+}): exact mass calculated for $[M]^{\cdot+}$ $(C_{17}H_{22}O_3)^{\cdot+}$ requires *m*/*z* 274.1563, found *m*/*z* 274.1555.

Methyl 4-(non-8-enoyl)benzoate

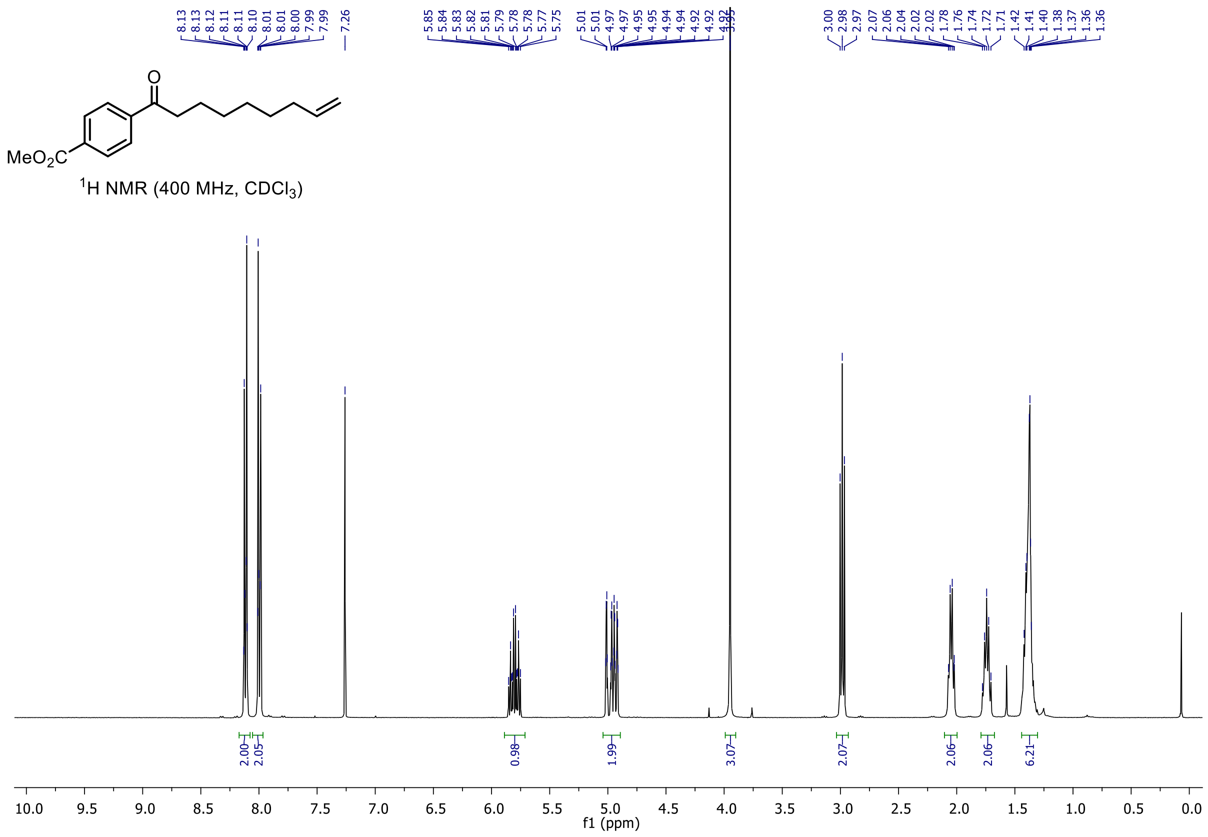

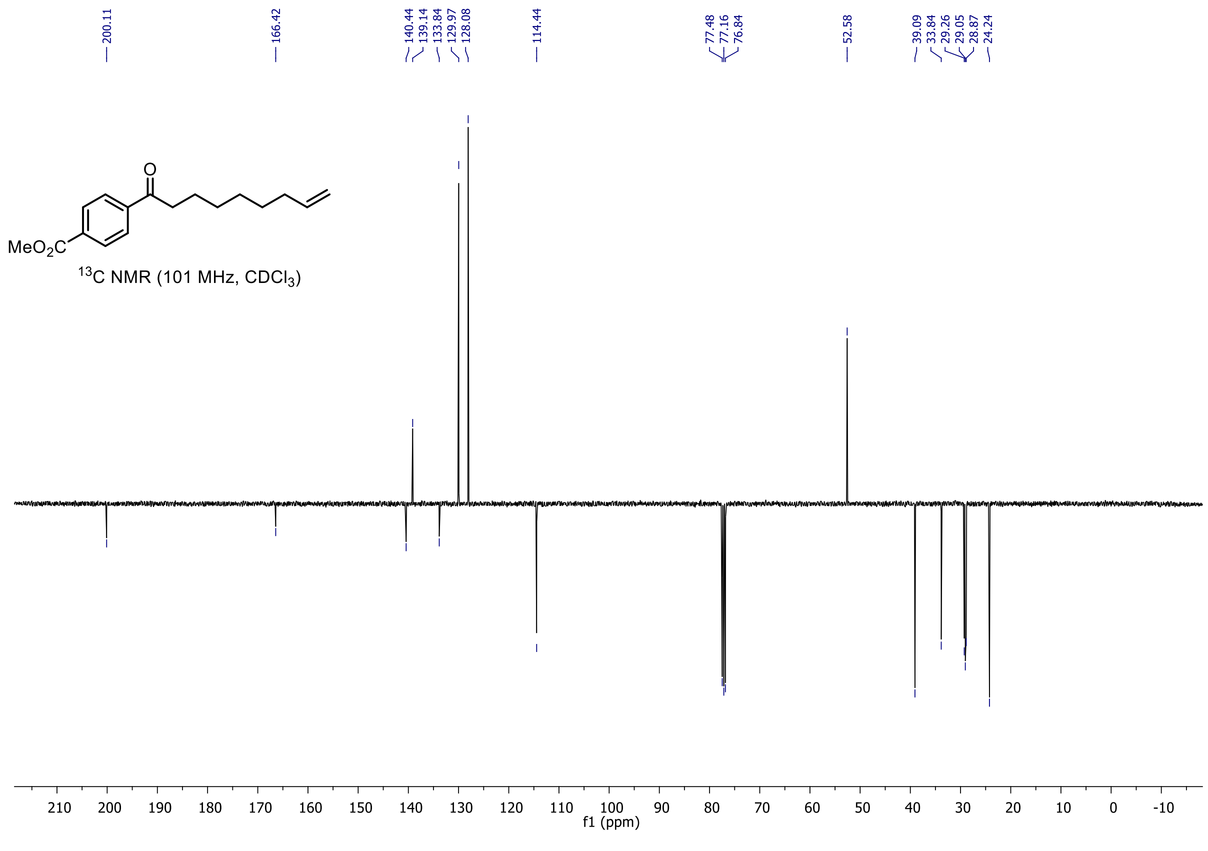

1-(3-Fluorophenyl)non-8-en-1-one

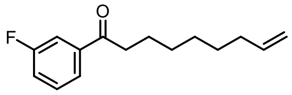

The title compound was prepared following general procedure C using 3-fluoro-*N*-methoxy-*N*-methylbenzamide (150 mg, 0.81 mmol, 1.00 equiv.) and a solution of 7-octenylmagnesium bromide (1.50 equiv.). Purification by flash column chromatography (0 – 20% EtOAc in heptaness) afforded the title compound (155 mg, 0.66 mmol, 81%) as a colorless liquid.

^{1}H NMR (400 MHz, CDCl$_3$): δ 7.77 – 7.71 (m, 1H), 7.64 (ddd, *J* = 9.5, 2.5, 1.6 Hz, 1H), 7.44 (td, *J* = 8.0, 5.6 Hz, 1H), 7.29 – 7.22 (m, 1H), 5.81 (ddt, *J* = 16.9, 10.2, 6.7 Hz, 1H), 5.05 – 4.89 (m, 2H), 3.00 – 2.89 (m, 2H), 2.11 – 2.01 (m, 2H), 1.79 – 1.69 (m, 2H), 1.45 – 1.34 (m, 6H).

^{13}C NMR (101 MHz, CDCl$_3$): δ 199.3, 163.0 (d, *J* = 247.4 Hz), 139.3 (d, *J* = 5.9 Hz), 139.1, 130.4 (d, *J* = 7.6 Hz), 123.9 (d, *J* = 2.9 Hz), 120.02 (d, *J* = 21.3 Hz), 115.0 (d, *J* = 22.2 Hz), 114.4, 38.9, 33.9, 29.3, 29.0, 28.9, 24.3.

^{19}F NMR (376 MHz, CDCl$_3$): δ -112.01 (1F).

IR (neat) v_{max}: 2927, 2855, 2360, 1687, 1640, 1588, 1441, 1366, 1242, 1151, 908, 874, 783, 681, 482.

HRMS (ESI$^+$): exact mass calculated for $[M+Na]^+$ $(C_{15}H_{19}FONa)^+$ requires *m/z* 257.1312, found *m/z* 257.1312.

1-(3-Fluorophenyl)non-8-en-1-one

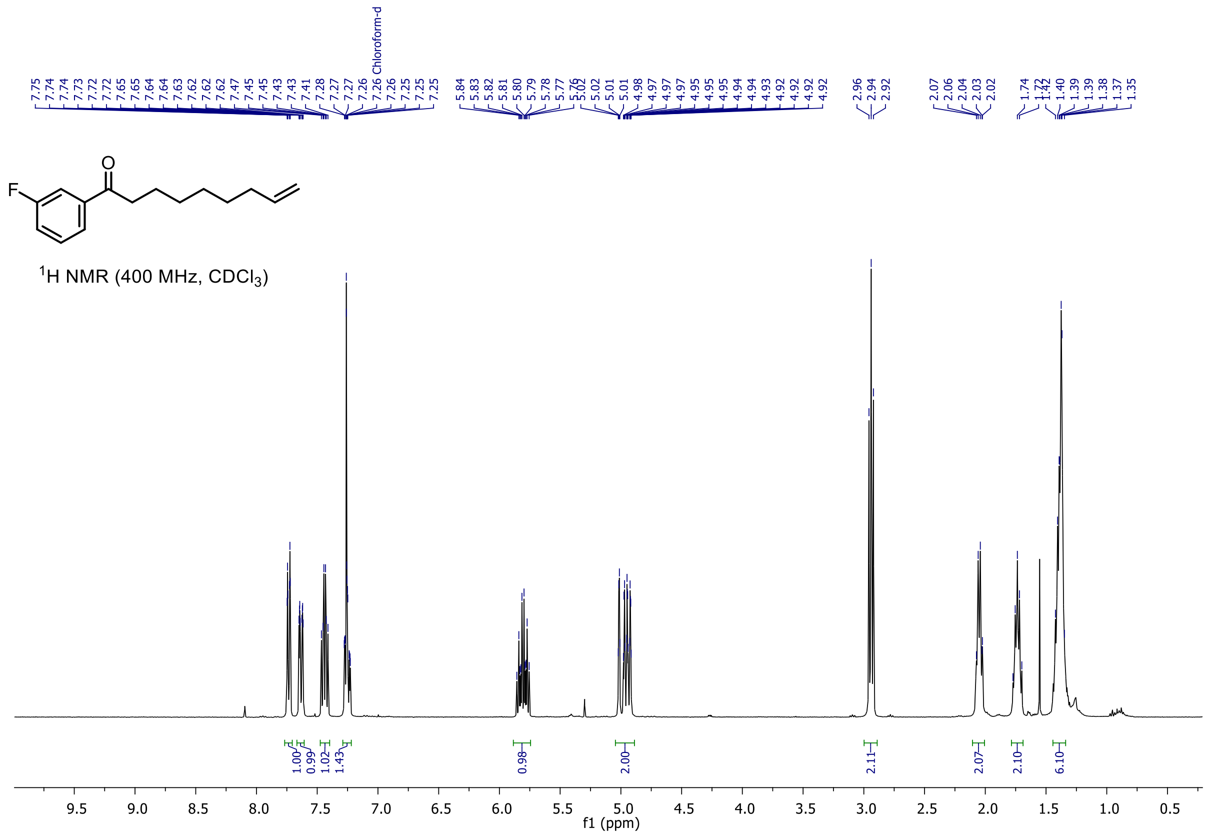

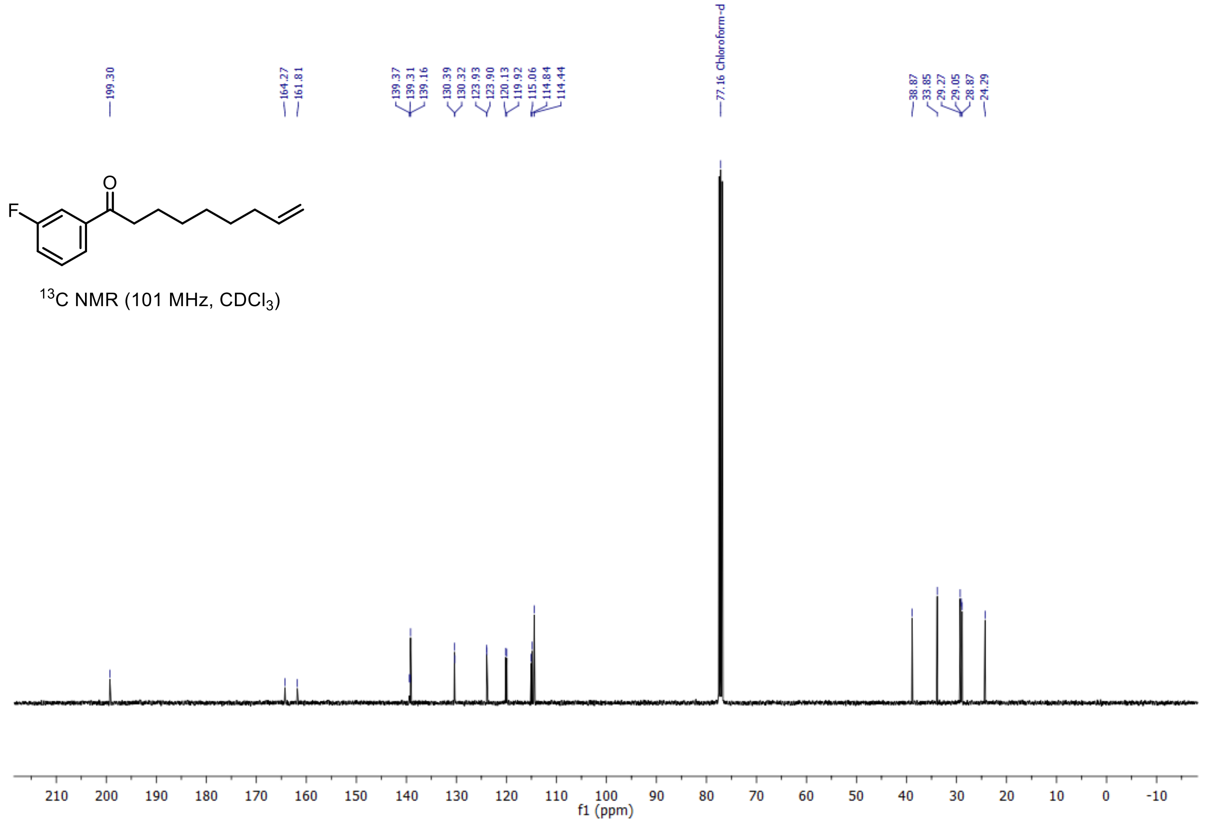

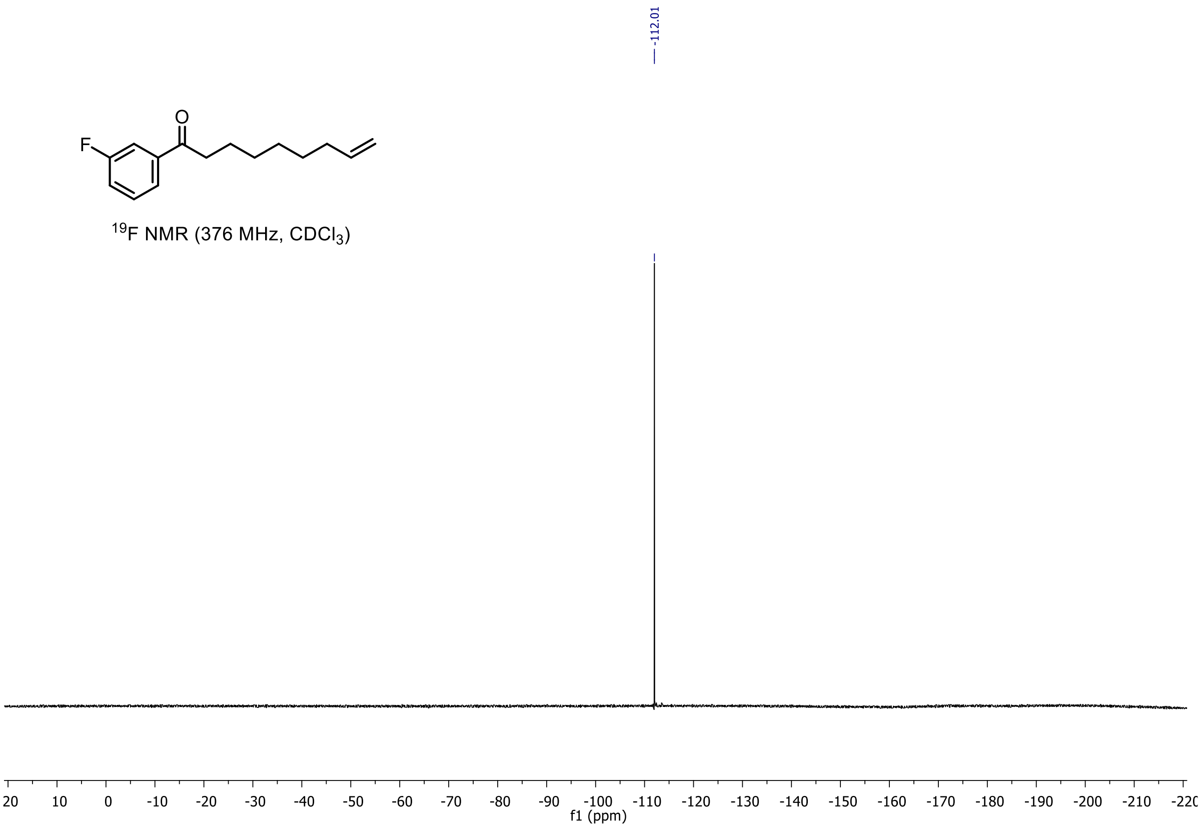
-112.01
^{19}F NMR (376 MHz, CDCl$_3$)
F
O
20 10 0 -10 -20 -30 -40 -50 -60 -70 -80 -90 -100 -110 -120 -130 -140 -150 -160 -170 -180 -190 -200 -210 -220
f1 (ppm)

1-(2-Hydroxyphenyl)non-8-en-1-one

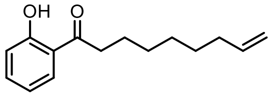

The title compound was prepared following general procedure C using 2-hydroxy-*N*-methoxy-*N*-methylbenzamide (362 mg, 2.00 mmol, 1.00 equiv.) and a solution of 7-octenylmagnesium bromide (1.50 equiv.). Purification by flash column chromatography (0 – 20% EtOAc in heptaness) afforded the title compound (206 mg, 0.88 mmol, 44%) as a colorless liquid.

^{1}H NMR (400 MHz, $CDCl_3$): δ 12.39 (s, 1H), 7.76 (dd, *J* = 8.0, 1.6 Hz, 1H), 7.51 – 7.41 (m, 1H), 6.98 (dd, *J* = 8.4, 0.8 Hz, 1H), 6.95 – 6.85 (m, 1H), 5.89 – 5.74 (m, 1H), 5.03 – 4.91 (m, 2H), 3.04 – 2.94 (m, 2H), 2.09 – 2.03 (m, 2H), 1.80 – 1.69 (m, 2H), 1.46 – 1.35 (m, 6H).

^{13}C NMR (101 MHz, $CDCl_3$): δ 207.1, 162.7, 139.1, 136.4, 130.1, 119.5, 119.0, 118.7, 114.5, 38.5, 33.9, 29.3, 29.0, 28.9, 24.6.

IR (neat) v_{max}: 3075, 2926, 2855, 1713, 1638, 1614, 1581, 1446, 1279, 1203, 1155, 1033, 908, 839, 723, 625, 561.

HRMS (ESI$^+$): exact mass calculated for $[M+Na]^+$ $(C_{15}H_{20}O_2Na)^+$ requires *m/z* 255.1356, found *m/z* 255.1350.

1-(2-Hydroxyphenyl)non-8-en-1-one

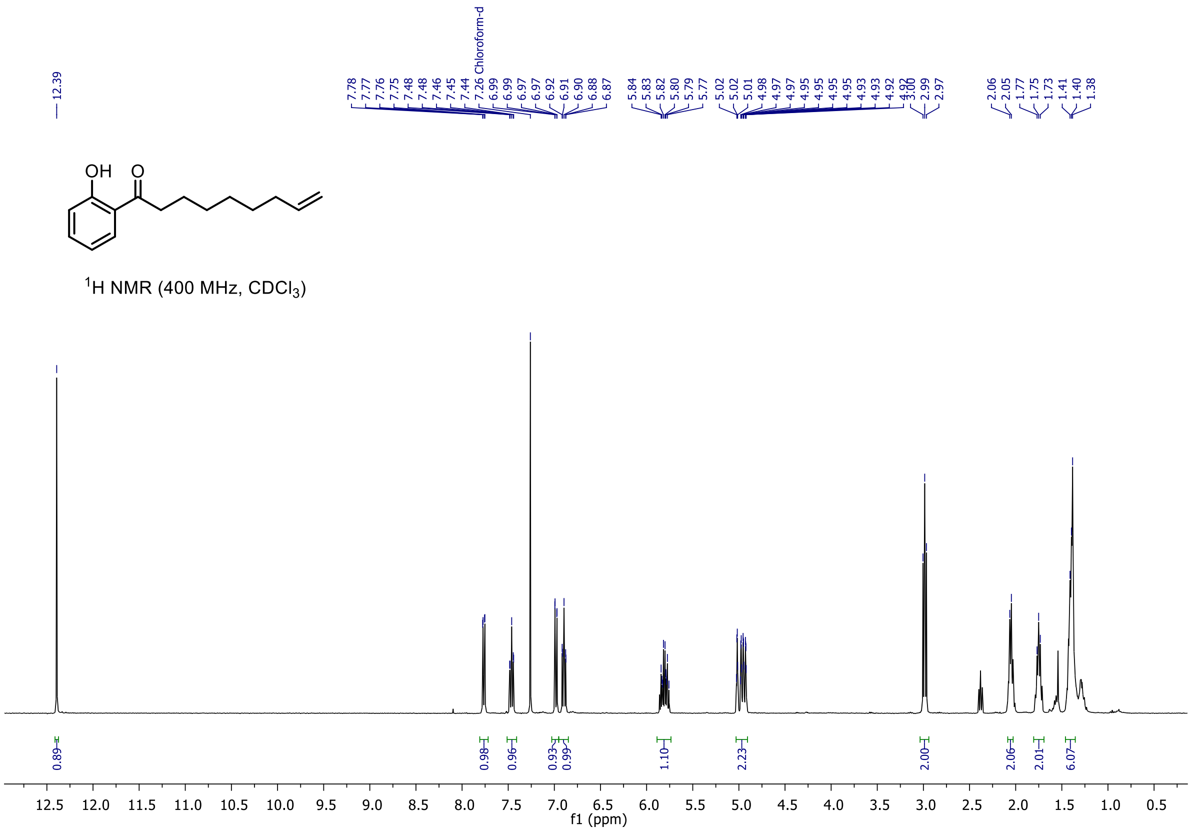

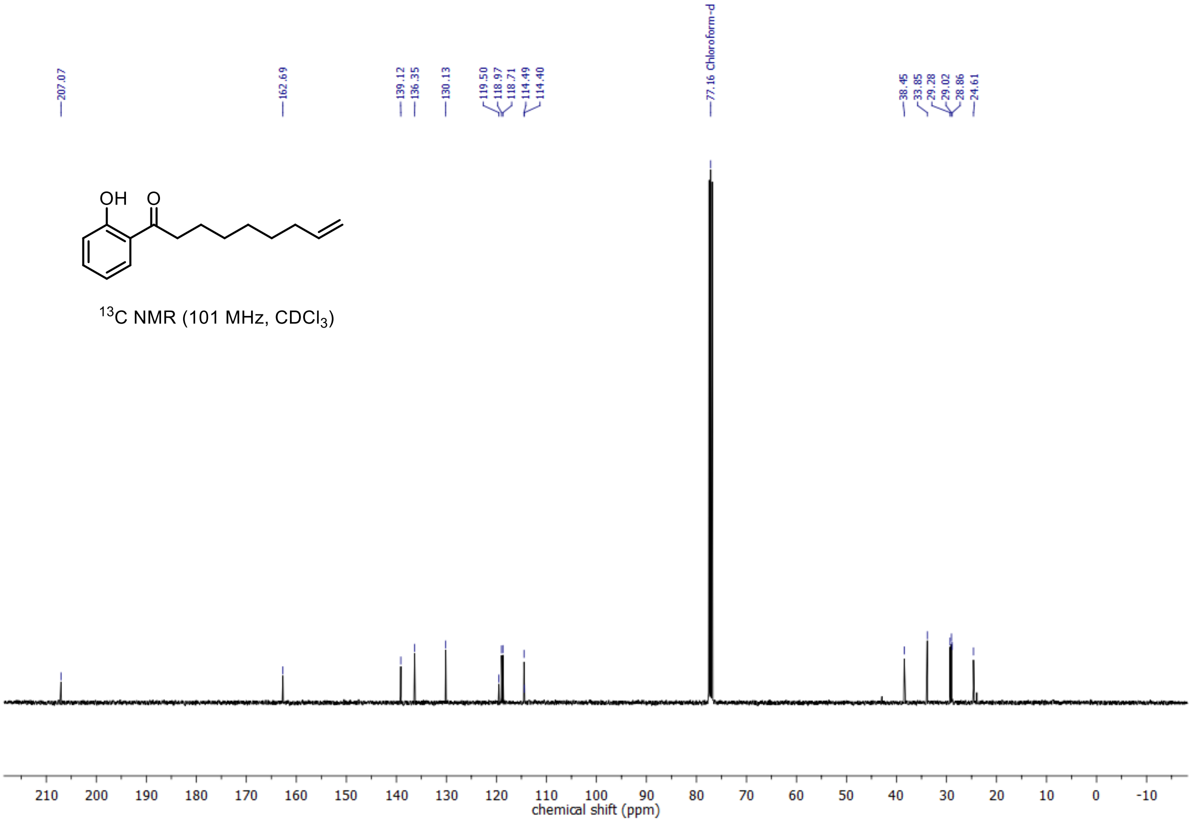

1-(4-(Dimethylamino)phenyl)non-8-en-1-one

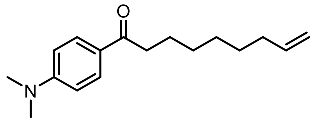

7-octenylmagnesium bromide solution was prepared following general procedure C using 8-bromo-1-octene (3.36 mL, 20.00 mmol, 1.00 equiv.) and magnesium (972 mg, 40.00 mmol, 2.00 equiv.).

The title compound was prepared following general procedure C using Weinreb amide 4-(dimethylamino)-*N*-methoxy-*N*-methylbenzamide (438 mg, 2.00 mmol, 1.00 equiv.) and a solution of 7-octenylmagnesium bromide (1.50 equiv.). Purification by flash column chromatography (0 – 10% EtOAc in heptanes) afforded the title compound as a colorless solid (176 mg, 0.68 mmol, 34%).

^{1}H NMR (700 MHz, $CDCl_3$) δ 7.89 – 7.86 (m, 2H), 6.68 – 6.62 (m, 2H), 5.80 (ddt, *J* = 16.9, 10.2, 6.7 Hz, 1H), 4.99 (ddd, *J* = 17.1, 3.6, 1.6 Hz, 1H), 4.92 (ddt, *J* = 10.2, 2.2, 1.2 Hz, 1H), 3.05 (s, 6H), 2.86 (t, *J* = 7.6 Hz, 2H), 2.07 – 2.01 (m, 2H), 1.74 – 1.69 (m, 2H), 1.42 – 1.32 (m, 6H).

^{13}C NMR (176 MHz, $CDCl_3$) δ 199.0 (C), 153.4 (C), 139.3 (CH), 130.4 (2CH), 125.3 (C), 114.3 (CH_2), 110.8 (2CH), 40.2 (2CH_3), 38.1 (CH_2), 33.9 (CH_2), 29.5 (CH_2), 29.1 (CH_2), 28.9 (CH_2), 25.2 (CH_2).

IR (neat) v_{max}: 3075, 2926, 2854, 1663, 1597, 1553, 1527, 1367, 1260, 1186, 1168.

HRMS (ESI$^+$): exact mass calculated for $[M+H]^+$ $(C_{17}H_{26}NO)^+$ requires *m*/*z* 260.2009, found *m*/*z* 260.2003.

1-(4-(Dimethylamino)phenyl)non-8-en-1-one

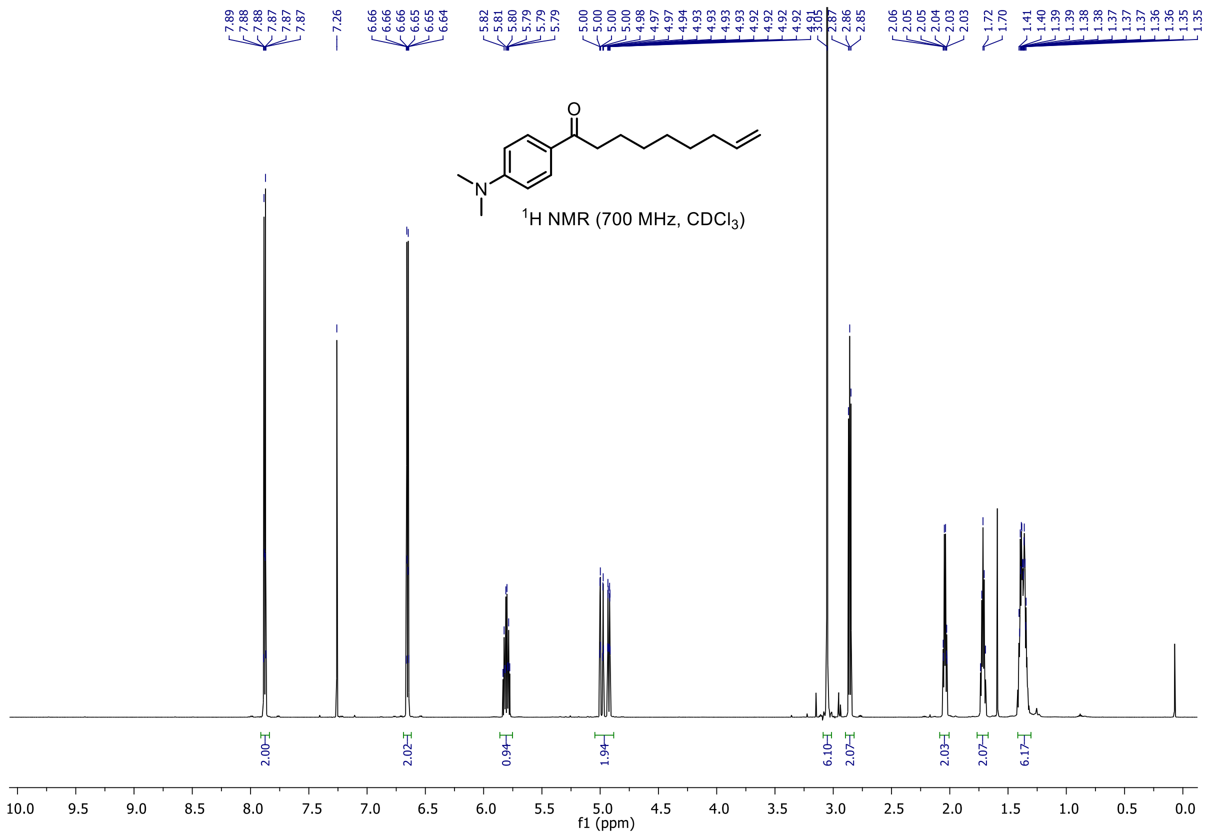

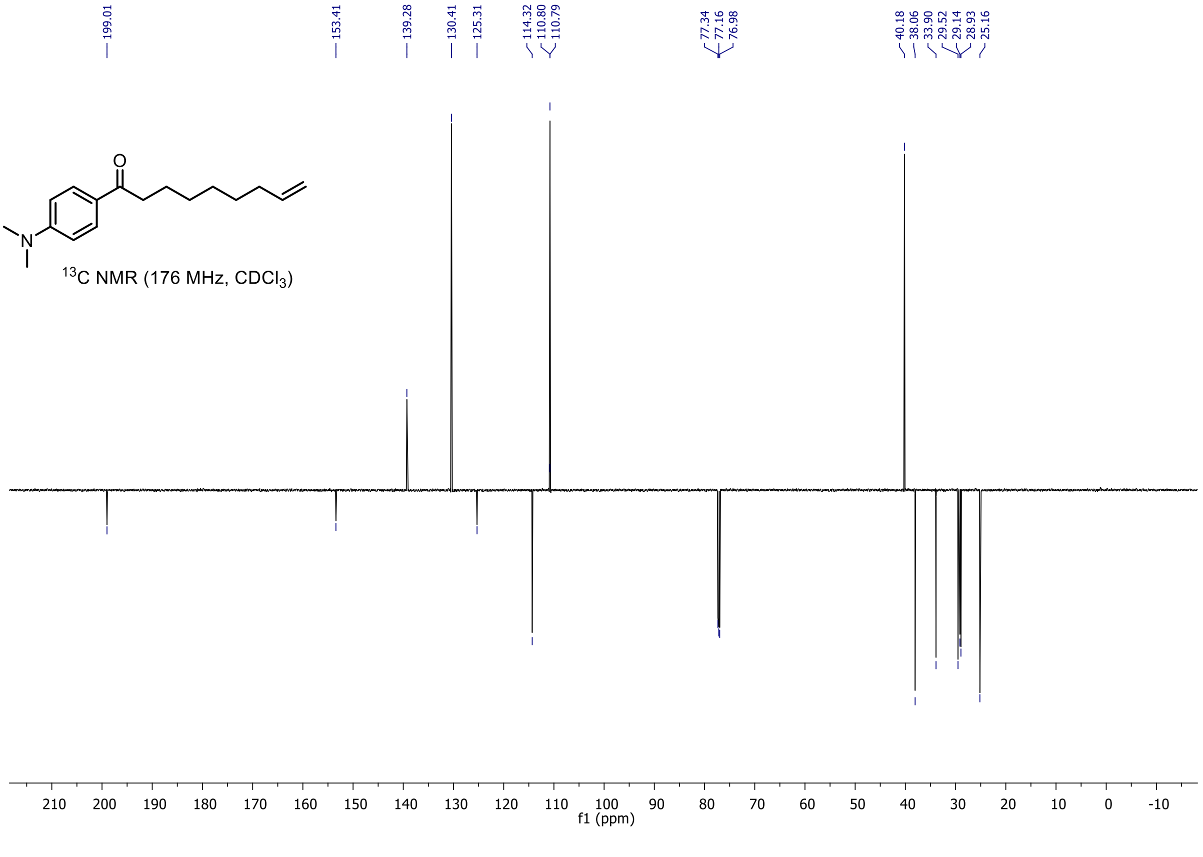

1-(2-(Methylthio)phenyl)non-8-en-1-one

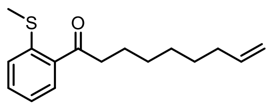

The title compound was prepared following general procedure C using *N*-methoxy-*N*-methyl-2-(methylthio)benzamide (423 mg, 2.00 mmol, 1.00 equiv.) and a solution of 7-octenylmagnesium bromide (2.00 equiv.). Purification by flash column chromatography (0 – 20% EtOAc in heptanes) afforded the title compound (302 mg, 1.15 mmol, 58%) as a yellow liquid.

^{1}H NMR (400 MHz, $CDCl_3$): δ 7.80 (dd, *J* = 7.8, 1.4 Hz, 1H), 7.49 – 7.43 (m, 1H), 7.33 (d, *J* = 8.0 Hz, 1H), 7.22 – 7.16 (m, 1H), 5.80 (ddt, *J* = 16.9, 10.2, 6.7 Hz, 1H), 5.03 – 4.89 (m, 2H), 2.97 – 2.92 (m, 2H), 2.43 (s, 3H), 2.08 – 2.00 (m, 2H), 1.78 – 1.69 (m, 2H), 1.42 - 1.33 (m, 6H).

^{13}C NMR (101 MHz, $CDCl_3$): δ 201.9, 142.2, 139.2, 135.0, 132.1, 130.1, 125.4, 123.6, 114.4, 40.2, 33.9, 29.3, 29.0, 28.9, 24.5, 16.2.

IR (neat) v_{max}: 2923, 2853, 1671, 1558, 1432, 1141, 907, 744, 648.

HRMS (ESI$^+$): exact mass calculated for $[M+Na]^+$ $(C_{16}H_{22}OSNa)^+$ requires *m/z* 285.1284, found *m/z* 285.1281.

1-(2-(Methylthio)phenyl)non-8-en-1-one

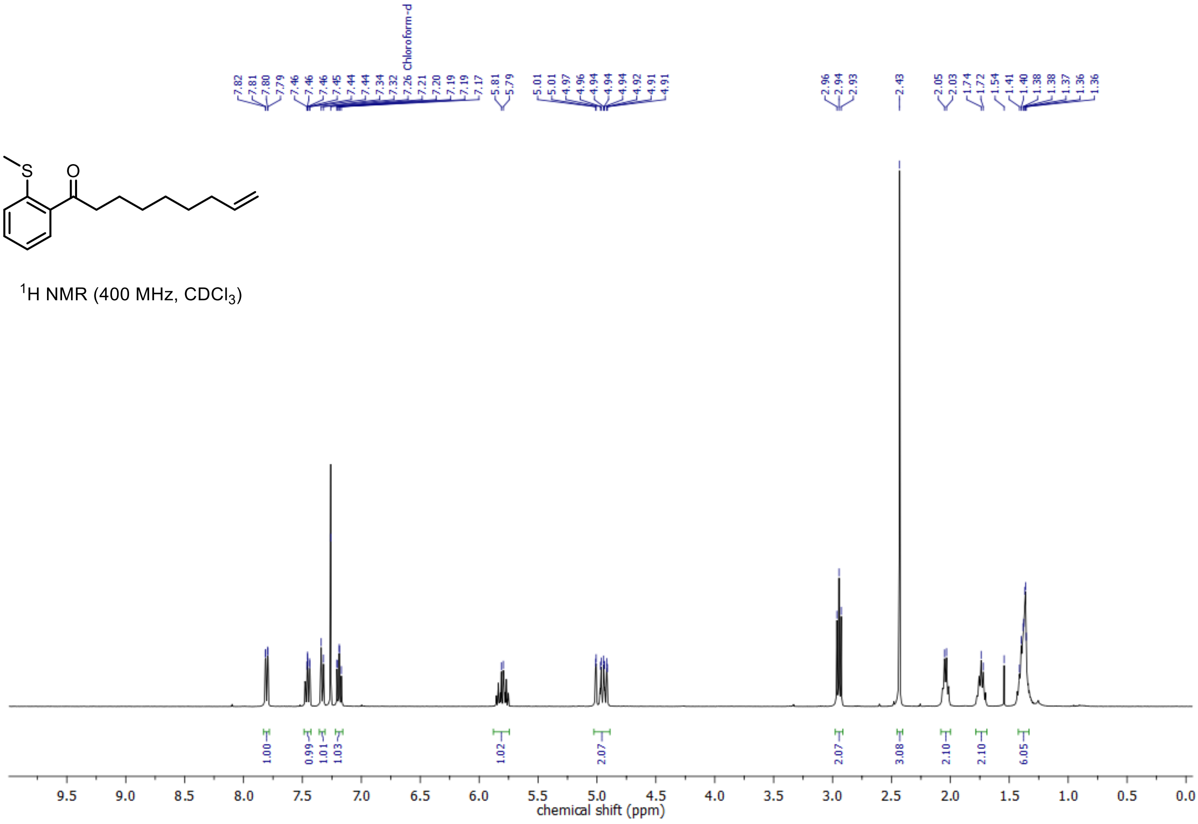

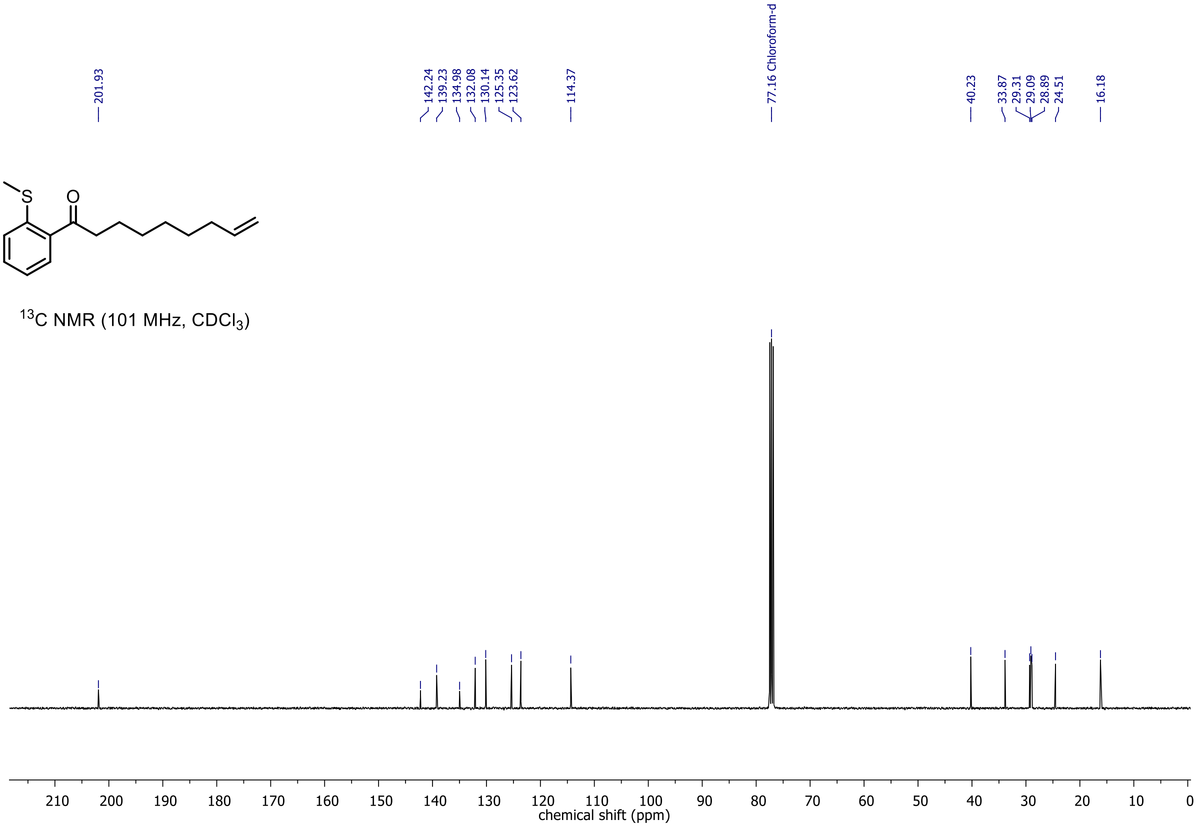

1-(4-Methoxyphenyl)dec-9-en-1-one

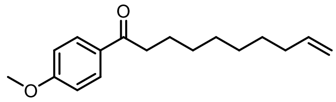

4-methoxyphenylmagnesium bromide solution was prepared following general procedure C using 4-bromoanisole (567 mg, 3.00 mmol, 1.00 equiv.) and magnesium (146 mg, 6.00 mmol, 2.00 equiv.).

The title compound was prepared following general procedure C using Weinreb amide *N*-methoxy-*N*-methyldec-9-enamide (427 mg, 2.00 mmol, 1.00 equiv.) and a solution of 4-methoxyphenylmagnesium bromide (1.50 equiv.). Purification by flash column chromatography (0 – 10% Et_2O in pentane) afforded the tile compound as a colorless solid (367 mg, 1.41 mmol, 71%).

^{1}H NMR (600 MHz, $CDCl_3$) δ 7.96 – 7.92 (m, 2H), 6.95 – 6.92 (m, 2H), 5.81 (ddt, *J* = 16.9, 10.2, 6.7 Hz, 1H), 4.99 (ddd, *J* = 17.1, 3.6, 1.6 Hz, 1H), 4.93 (ddt, *J* = 10.2, 2.2, 1.2 Hz, 1H), 3.87 (s, 3H), 2.90 (t, *J* = 7.5 Hz, 2H), 2.07 – 2.01 (m, 2H), 1.75 – 1.69 (m, 2H), 1.41 – 1.29 (m, 8H).

^{13}C NMR (151 MHz, $CDCl_3$) δ 199.4 (C), 163.5 (C), 139.3 (CH), 130.5 (2CH), 130.4 (C), 114.3 (CH_2), 113.8 (2CH), 55.6 (CH_3), 38.5 (CH_2), 33.9 (CH_2), 29.5 (CH_2), 29.4 (CH_2), 29.1 (CH_2), 29.0 (CH_2), 24.8 (CH_2).

IR (neat) v_{max}: 3075, 2926, 2853, 1675, 1599, 1575, 1509, 1307, 1255, 1168, 1030, 909, 831.

HRMS (ESI$^+$): exact mass calculated for $[M+H]^+$ $(C_{17}H_{25}O_2)^+$ requires *m/z* 261.1849, found *m/z* 261.1848.

1-(4-Methoxyphenyl)dec-9-en-1-one

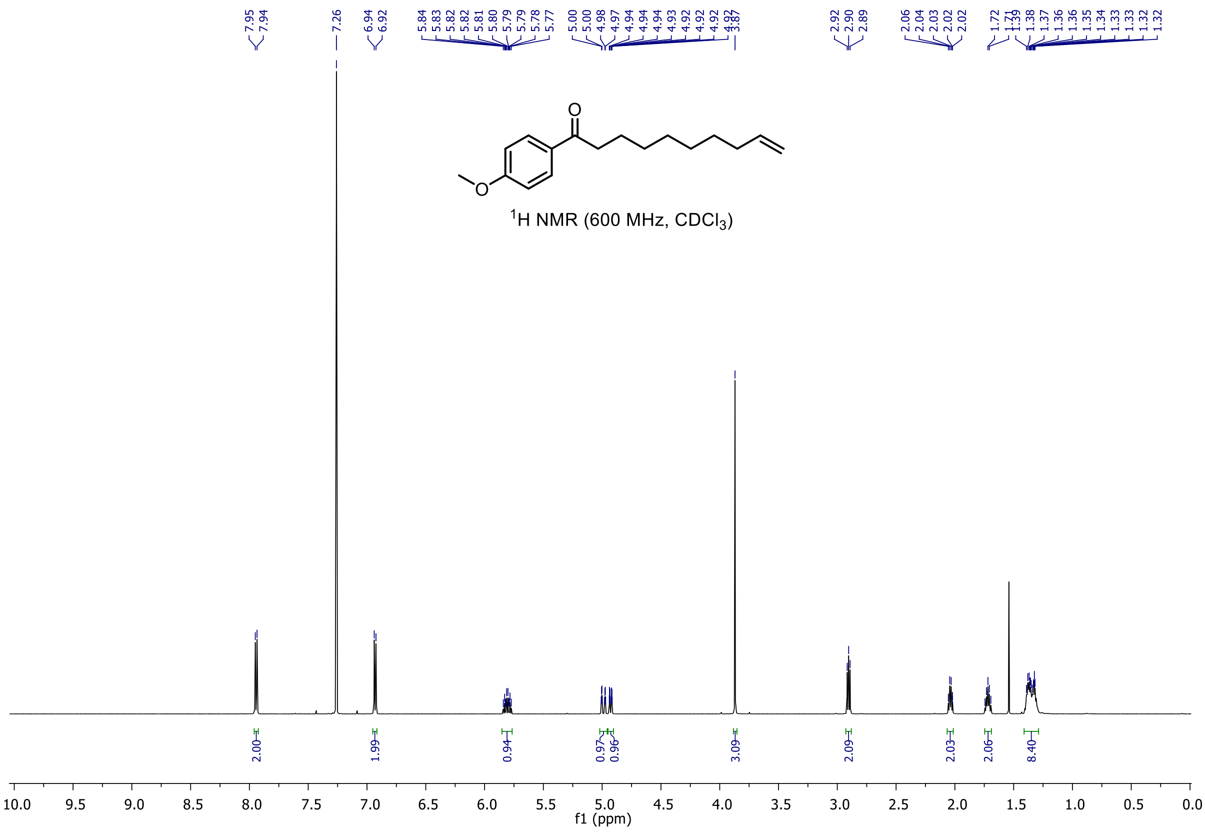

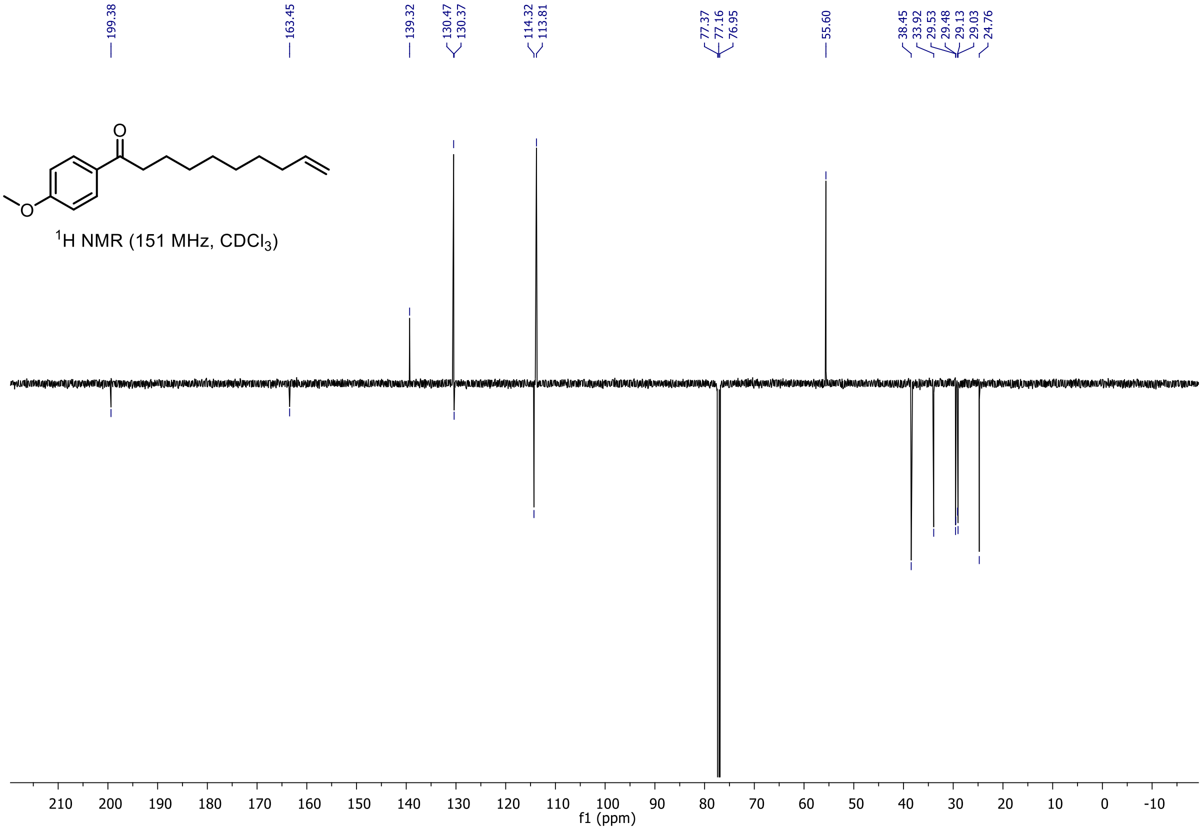

1-(Thiophen-2-yl)non-8-en-1-one

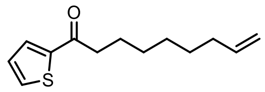

The title compound was prepared following general procedure C using *N*-methoxy-*N*-methylthiophene-2-carboxamide (350 mg, 2.04 mmol, 1.00 equiv.) and a solution of 7-octenylmagnesium bromide (1.70 equiv.). Purification by flash column chromatography (0 – 20% EtOAc in heptanes) afforded the title compound (242 mg, 1.09 mmol, 53%) as a colorless liquid.

^{1}H NMR (400 MHz, $CDCl_3$): δ 7.70 (dd, *J* = 3.8, 1.1 Hz, 1H), 7.61 (dd, *J* = 5.0, 1.1 Hz, 1H), 7.12 (dd, *J* = 4.9, 3.8 Hz, 1H), 5.80 (ddt, *J* = 16.9, 10.2, 6.7 Hz, 1H), 5.04 – 4.85 (m, 2H), 2.94 – 2.83 (m, 2H), 2.10 – 1.96 (m, 2H), 1.81 – 1.68 (m, 2H), 1.45 – 1.32 (m, 6H).

^{13}C NMR (101 MHz, $CDCl_3$): δ 193.6, 144.7, 139.2, 133.5, 131.8, 128.2, 114.4, 39.5, 33.8, 29.3, 29.0, 28.9, 24.9.

IR (neat) ν_{max}: 2926, 2854, 1658, 1518, 1414, 1355, 1256, 1233, 1057, 994, 908, 856, 718.

HRMS (ESI$^+$): exact mass calculated for $[M+Na]^+$ $(C_{13}H_{18}OSNa)^+$ requires *m/z* 245.0971, found *m/z* 245.0965.

1-(Thiophen-2-yl)non-8-en-1-one

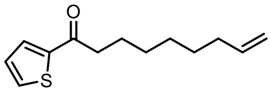

^{1}H NMR (400 MHz, $CDCl_3$)

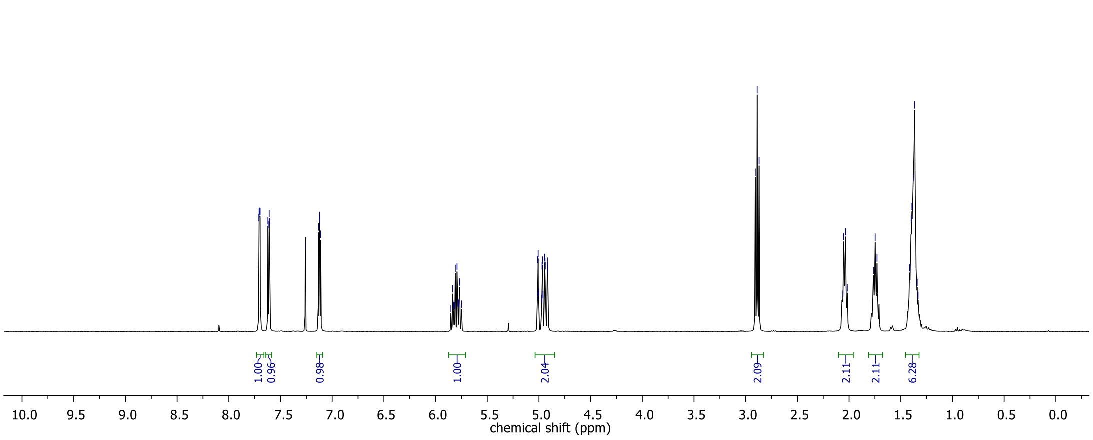

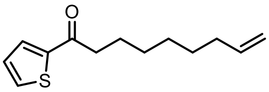

^{13}C NMR (101 MHz, $CDCl_3$)

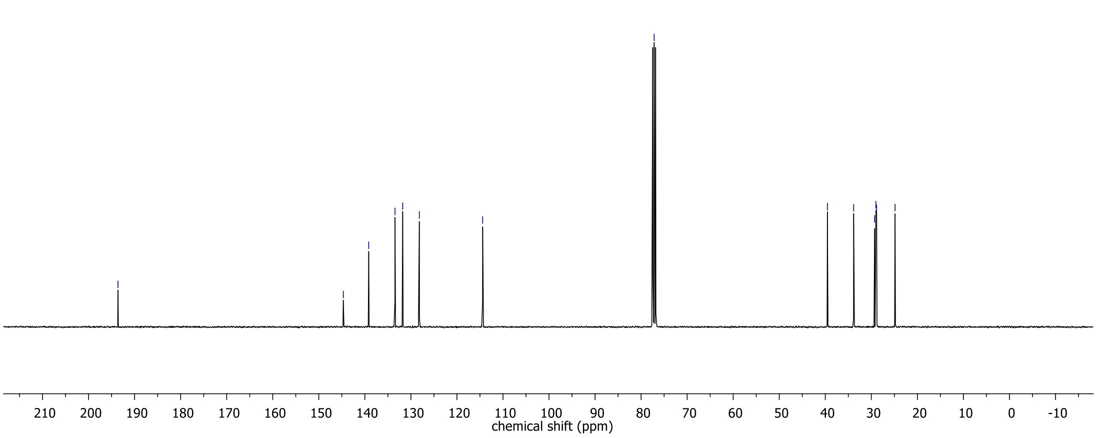

1-(Furan-2-yl)non-8-en-1-one

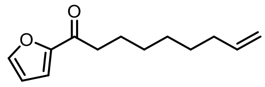

7-octenylmagnesium bromide solution was prepared following general procedure C using 8-bromo-1-octene (3.36 mL, 20.00 mmol, 1.00 equiv.) and magnesium (972 mg, 40.00 mmol, 2.00 equiv.).

The title compound was prepared following general procedure C using Weinreb amide *N*-methoxy-*N*-methylfuran-2-carboxamide (245 mg, 1.58 mmol, 1.00 equiv.) and a solution of 7-octenylmagnesium bromide (1.50 equiv.). Purification by flash column chromatography (0 – 10% EtOAc in heptanes) afforded the title compound as a yellow oil (257 mg, 1.25 mmol, 79%).

^{1}H NMR (400 MHz, $CDCl_3$) δ 7.50 (dd, *J* = 1.6, 0.6 Hz, 1H), 7.10 (dd, *J* = 3.5, 0.6 Hz, 1H), 6.45 (dd, *J* = 3.5, 1.7 Hz, 1H), 5.73 (ddt, *J* = 16.9, 10.2, 6.7 Hz, 1H), 4.92 (ddd, *J* = 17.1, 3.6, 1.6 Hz, 1H), 4.86 (ddt, *J* = 10.2, 2.2, 1.2 Hz, 1H), 2.74 (t, *J* = 7.5 Hz, 2H), 2.02 – 1.93 (m, 2H), 1.70 – 1.60 (m, 2H), 1.37 – 1.23 (m, 6H).

^{13}C NMR (101 MHz, $CDCl_3$) δ 189.9 (C), 153.0 (C), 146.3 (CH), 139.2 (CH), 116.9 (CH), 114.4 (CH_2), 112.2 (CH), 38.6 (CH_2), 33.8 (CH_2), 29.3 (CH_2), 29.0 (CH_2), 28.8 (CH_2), 24.4 (CH_2).

IR (neat) ν_{max}: 3076, 2927, 2855, 1674, 1568, 1467, 1394, 1264, 1158, 1011, 996, 760.

HRMS (EI^{+}): exact mass calculated for $[M–C_8H_{15}^{\cdot}]^+$ $(C_5H_3O_2)^+$ requires *m*/*z* 95.0128, found *m*/*z* 95.0124 and $[M–C_5H_3O_2]^+$ $(C_8H_{15})^+$ requires *m*/*z* 111.1168, found *m*/*z* 111.1162.

1-(Furan-2-yl)non-8-en-1-one

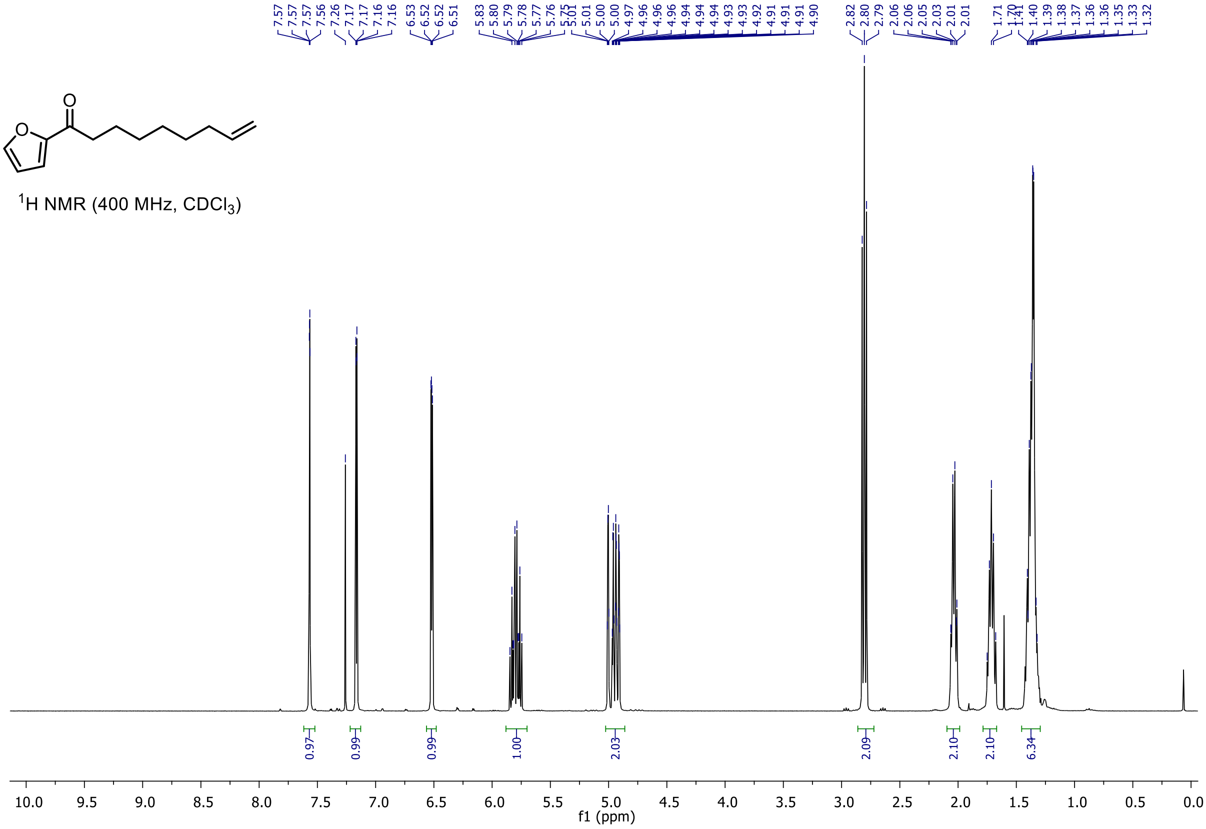

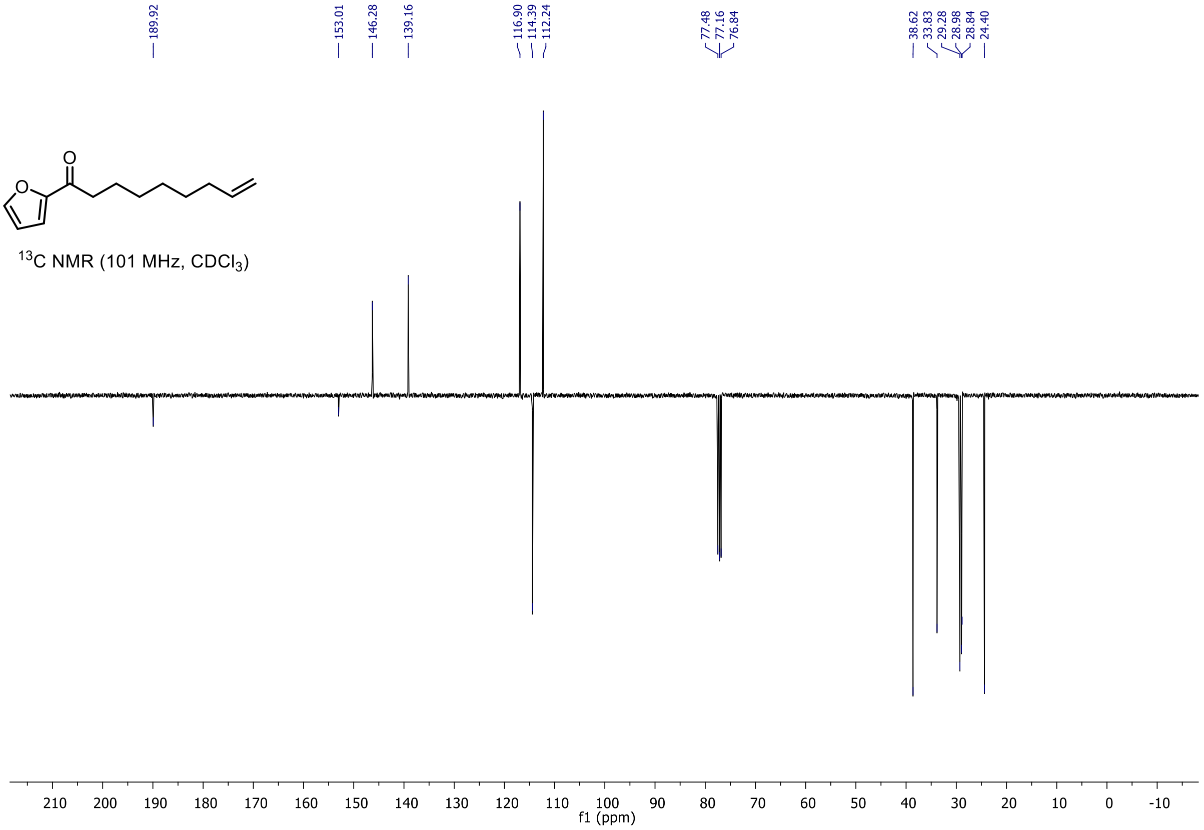

1-Ferrocene-non-8-en-1-one

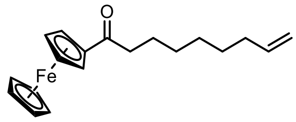

The title compound was prepared following general procedure C using *N*-methoxy-*N*-methylferrocene-1-carboxamide (546 mg, 2.00 mmol, 1.00 equiv.) and a solution of 7-octenylmagnesium bromide (1.50 equiv.). Purification by flash column chromatography (0 – 20% EtOAc in heptanes) afforded the title compound (449 mg, 1.38 mmol, 69%) as a dark red liquid.

^{1}H NMR (400 MHz, $CDCl_3$): δ 5.82 (ddt, *J* = 16.9, 10.2, 6.7 Hz, 1H), 5.04 – 4.90 (m, 2H), 4.83 – 4.75 (m, 2H), 4.53 – 4.46 (m, 2H), 4.19 (s, 5H), 2.69 (t, *J* = 7.5 Hz, 2H), 2.12 – 2.01 (m, 2H), 1.76 – 1.66 (m, 2H), 1.44 – 1.35 (m, 6H).

^{13}C NMR (101 MHz, $CDCl_3$): δ 204.8, 139.2, 114.4, 79.4, 72.2 (2C), 69.9 (5C), 69.5 (2C), 39.9, 33.9, 29.5, 29.1, 28.9, 24.7.

IR (neat) ν_{max}: 3081, 2925, 2853, 1640, 1452, 1411, 1353, 1255, 1106, 1059, 999, 732, 639, 594, 479.

HRMS (ESI$^+$): exact mass calculated for $[M+Na]^+$ ($C_{19}H_{24}FeONa$)$^+$ requires *m/z* 347.1069, found *m/z* 347.1068.

1-Ferrocene-non-8-en-1-one

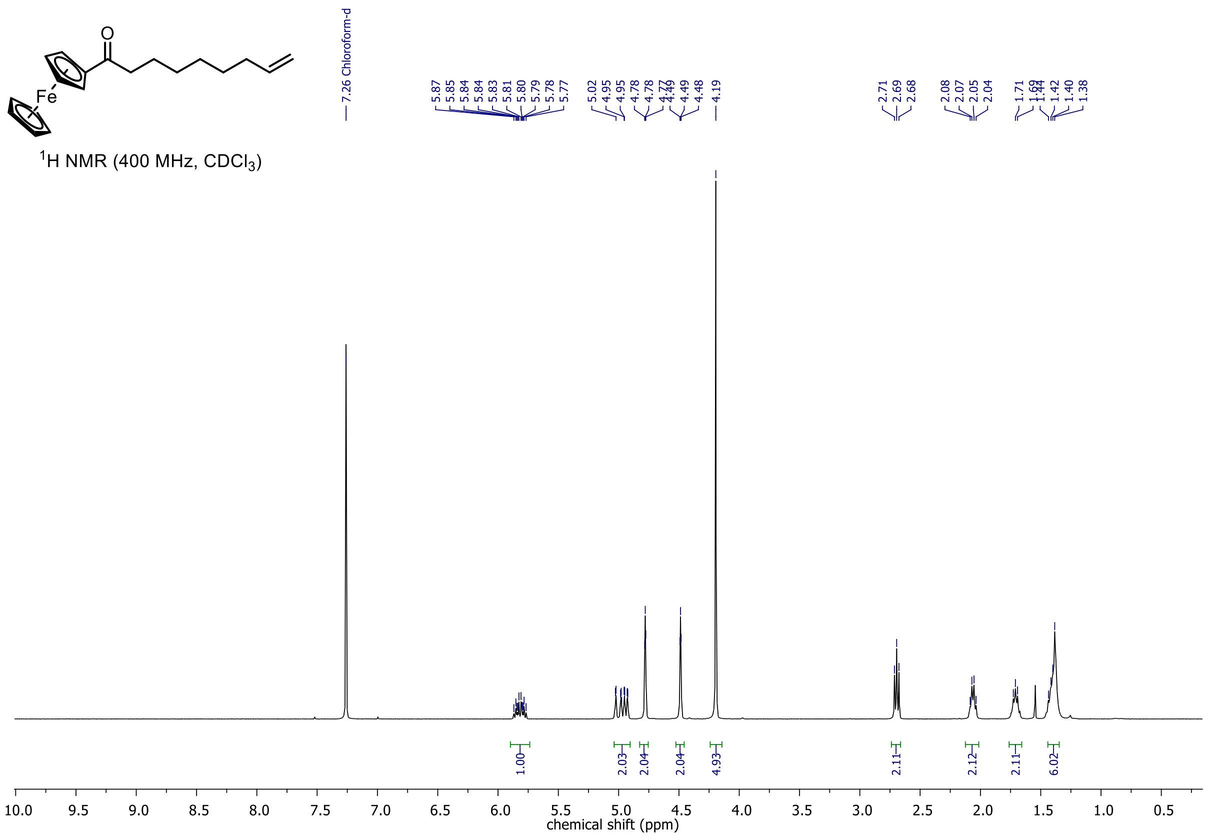

^{1}H NMR (400 MHz, $CDCl_3$)

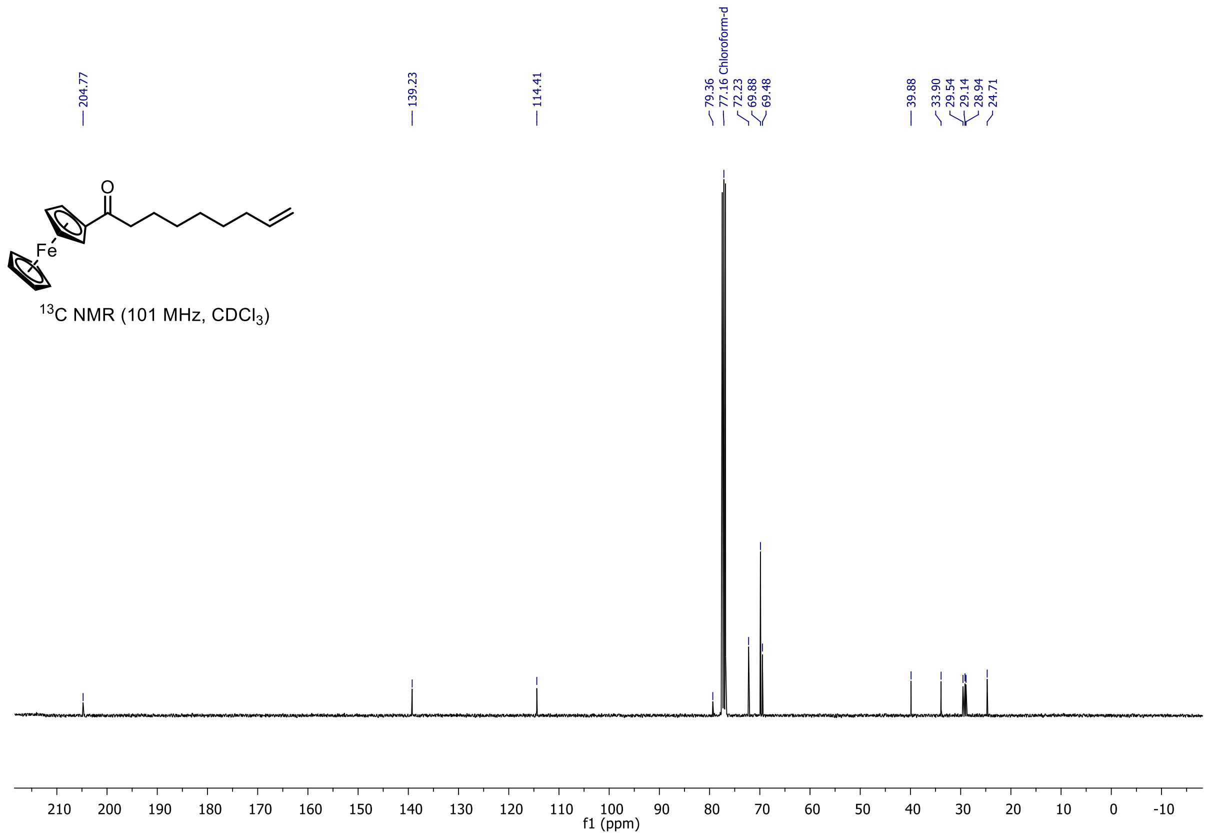

^{13}C NMR (101 MHz, $CDCl_3$)

1-(3,5-Dimethylisoxazol-4-yl)non-8-en-1-one

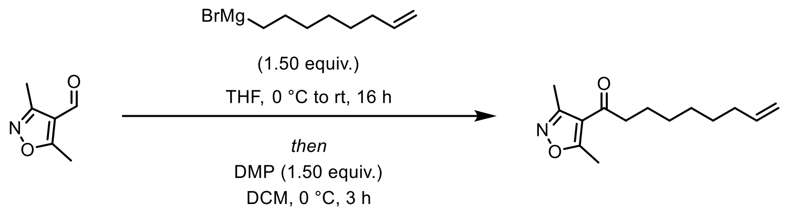

The title compound was prepared following general procedure D using 3,5-dimethyl-4-isoxazolecarbaldehyde (375 mg, 3.00 mmol, 1.00 equiv.) and a solution of 7-octenylmagnesium bromide (1.50 equiv.). The crude material obtained from this reaction (no column chromatography performed), was dissolved in DCM (5 mL) and the mixture was cooled to 0°C. Dess-Martin periodinane (1.91 g, 4.50 mmol, 1.50 equiv.) was added and the resulting suspension was stirred at 0°C for 3 h. The reaction mixture was diluted with DCM (20 mL) and aqueous saturated $NaHCO_3$ solution (20 mL). The organic phase was separated and the aqueous layer was extracted with DCM (3 × 10 mL). The combined organic phases were washed with brine (1 × 10 mL), dried over anhydrous sodium sulfate, the dried solution was filtered, and the filtrate was concentrated under reduced pressure. The crude product was purified by flash column chromatography (0 – 50% EtOAc in heptanes), affording the title compound (371 mg, 1.58 mmol, 63%) as a colorless liquid.

^{1}H NMR (400 MHz, $CDCl_3$) δ 5.80 (ddt, *J* = 16.9, 10.2, 6.7 Hz, 1H), 5.05 – 4.90 (m, 2H), 2.73 – 2.68 (m, 2H), 2.67 (s, 3H), 2.46 (s, 3H), 2.09 – 2.01 (m, 2H), 1.77 – 1.62 (m, 2H), 1.47 – 1.28 (m, 6H).

^{13}C NMR (101 MHz, $CDCl_3$ δ 195.1, 173.6, 159.3, 139.1, 117.0, 114.5, 42.5, 33.8, 29.2, 29.1, 28.8, 23.6, 14.4, 12.3.

IR (neat) v_{max}: 2927, 2855, 1675, 1640, 1584, 1420, 1278, 1064, 962, 909.

HRMS (ESI$^+$): exact mass calculated for $[M+H]^+$ $(C_{14}H_{22}NO_2)^+$ requires *m/z* 236.1645 found *m/z* 236.1652.

1-(3,5-Dimethylisoxazol-4-yl)non-8-en-1-one

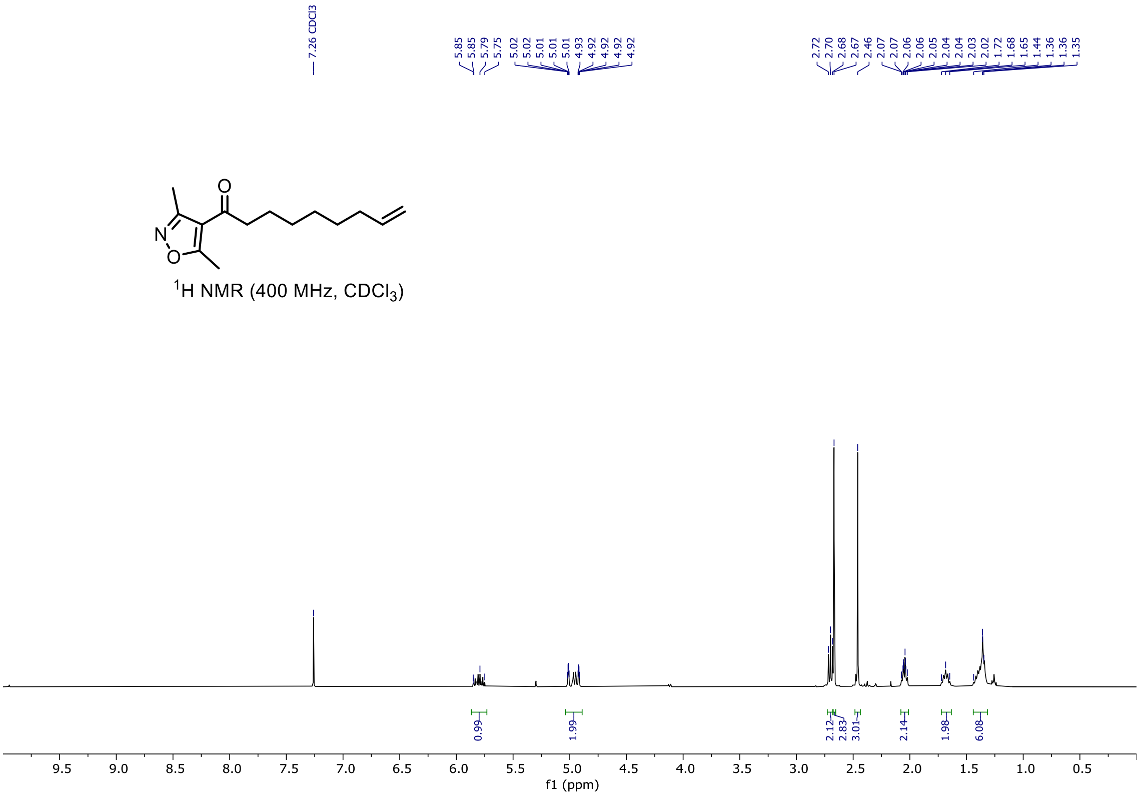

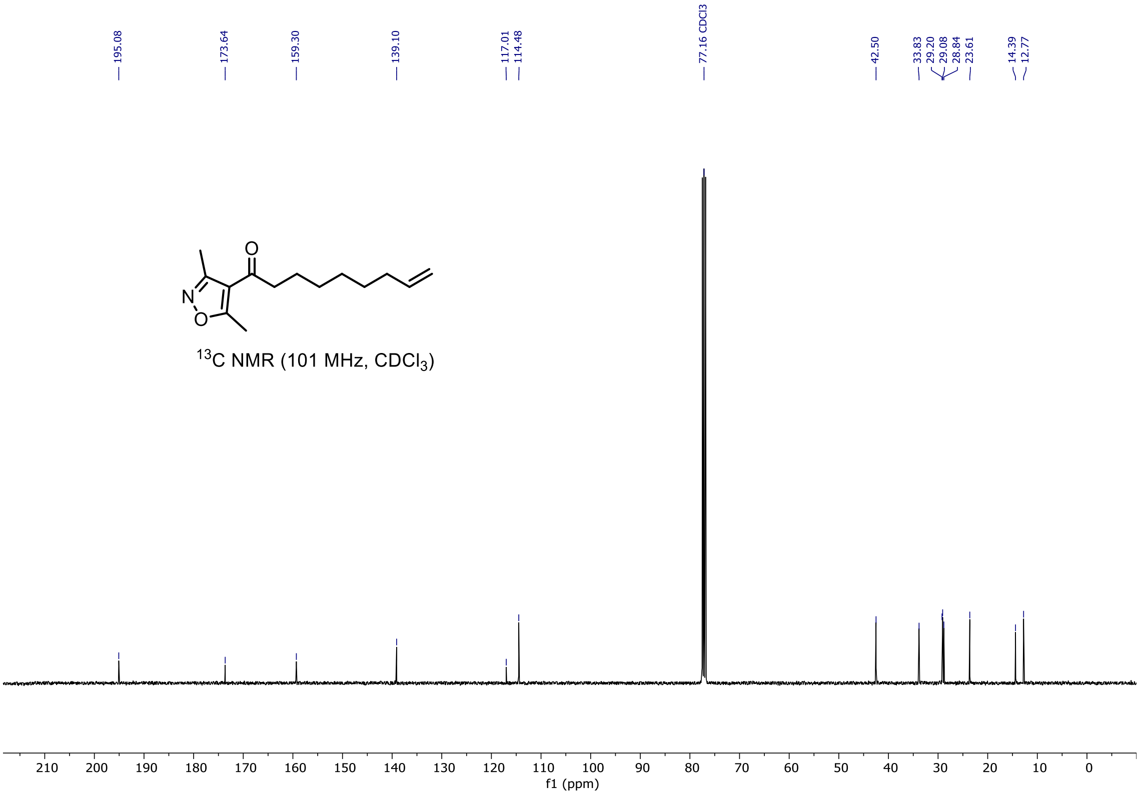

1-(3-(Difluoromethyl)-1-methyl-1*H*-pyrazol-4-yl)non-8-en-1-one

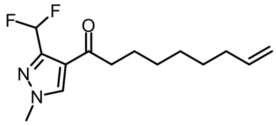

The title compound was prepared following general procedure C using the Weinreb amide 3-(difluoromethyl)-*N*-methoxy-*N*,1-dimethyl-1H-pyrazole-4-carboxamide (1.13 g, 5.00 mmol, 1.00 equiv.) and a solution of 7-octenylmagnesium bromide (1.50 equiv.). Purification by flash column chromatography (0 – 100% EtOAc in heptanes) afforded the title compound (600 mg, 2.22 mmol, 74%) as a colorless liquid.

^{1}H NMR (700 MHz, $CDCl_3$): δ 7.85 (t, *J* = 1.3 Hz, 1H), 7.13 (t, *J* = 54.1 Hz, 1H), 5.82 – 5.75 (m, 1H), 5.01 – 4.90 (m, 2H), 3.96 (s, 3H), 2.72 (t, *J* = 7.4 Hz, 2H), 2.07 – 1.99 (m, 2H), 1.71 – 1.64 (m, 2H), 1.41 – 1.29 (m, 6H).

^{13}C NMR (176 MHz, $CDCl_3$): δ 194.3, 146.2 (t, *J* = 24.1 Hz), 139.1, 134.2, 121.5 (t, *J* = 2.7 Hz), 114.4, 109.7 (t, *J* = 236.6 Hz), 41.0, 39.8, 33.8, 29.2, 29.0, 28.8, 24.1.

^{19}F NMR (659 MHz, $CDCl_3$): δ -115.85.

IR (neat) v_{max}: 2928, 2856, 1671, 1538, 1498, 1445, 1417, 1356, 1167, 1075, 1038, 910, 858.

HRMS (ESI$^+$): exact mass calculated for $[M+H]^+$ $(C_{14}H_{21}F_2N_2O)^+$ requires *m/z* 271.1616, found *m/z* 271.1617.

1-(3-(Difluoromethyl)-1-methyl-1H-pyrazol-4-yl)non-8-en-1-one

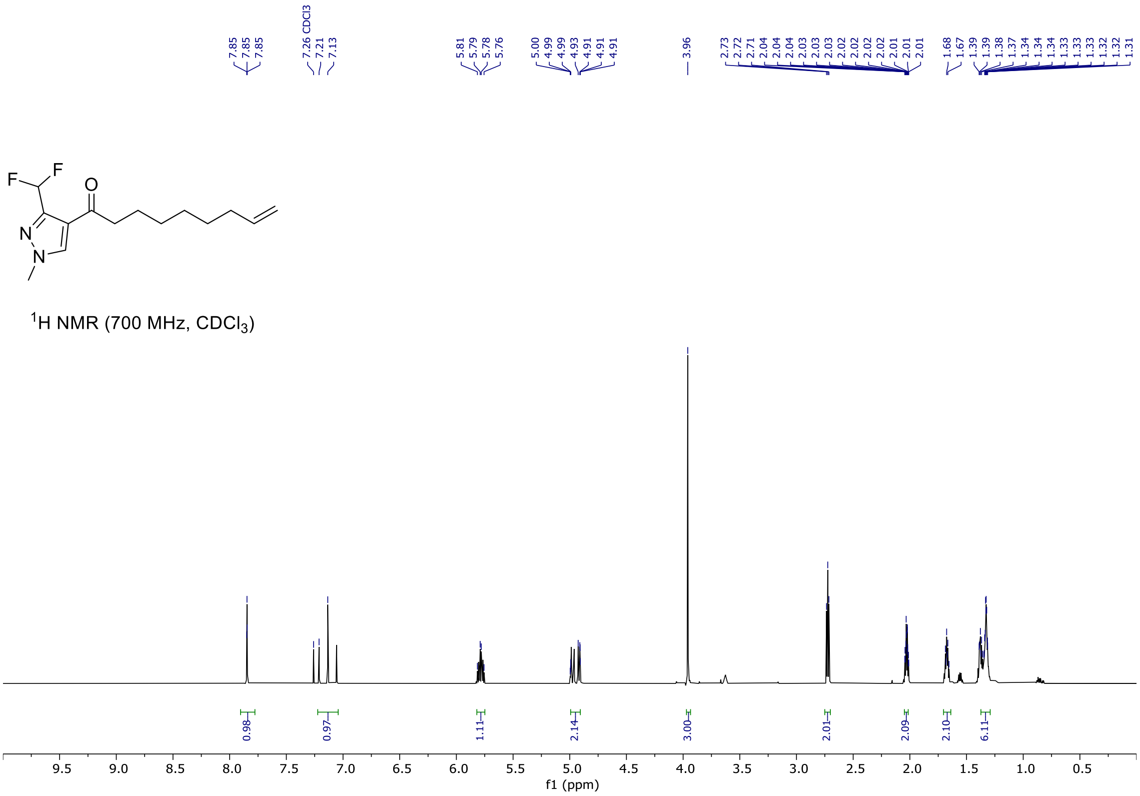

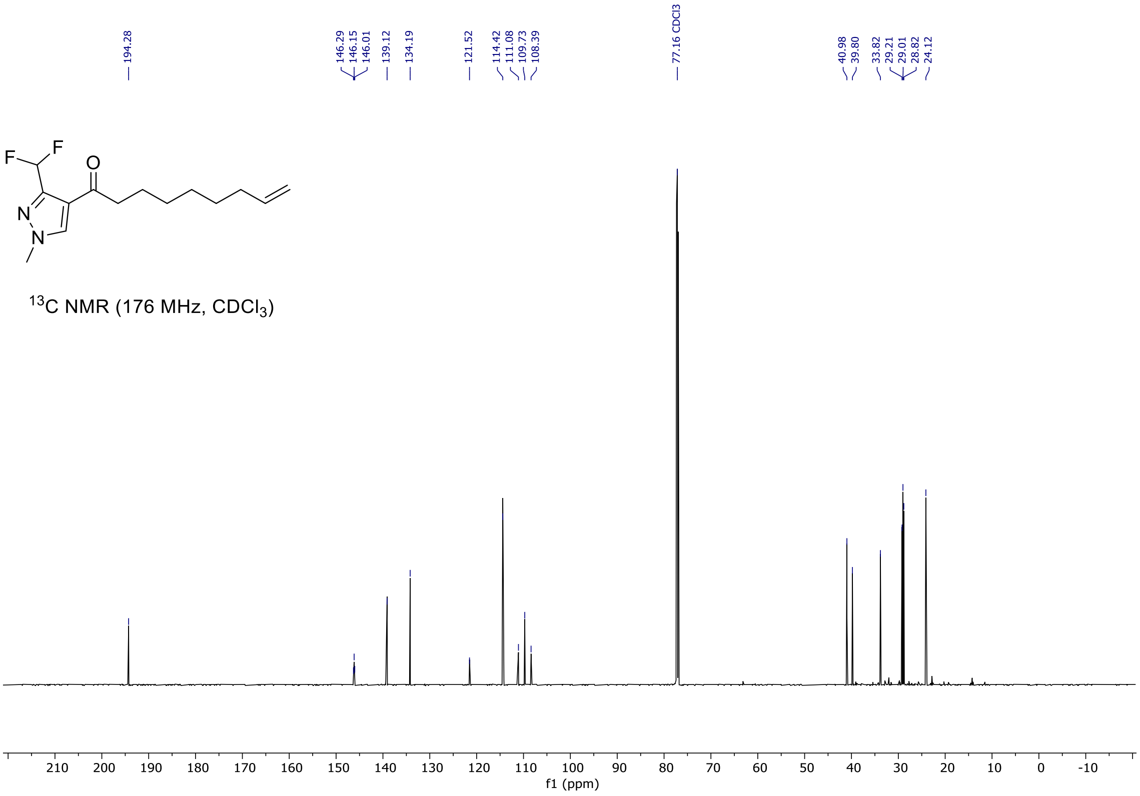

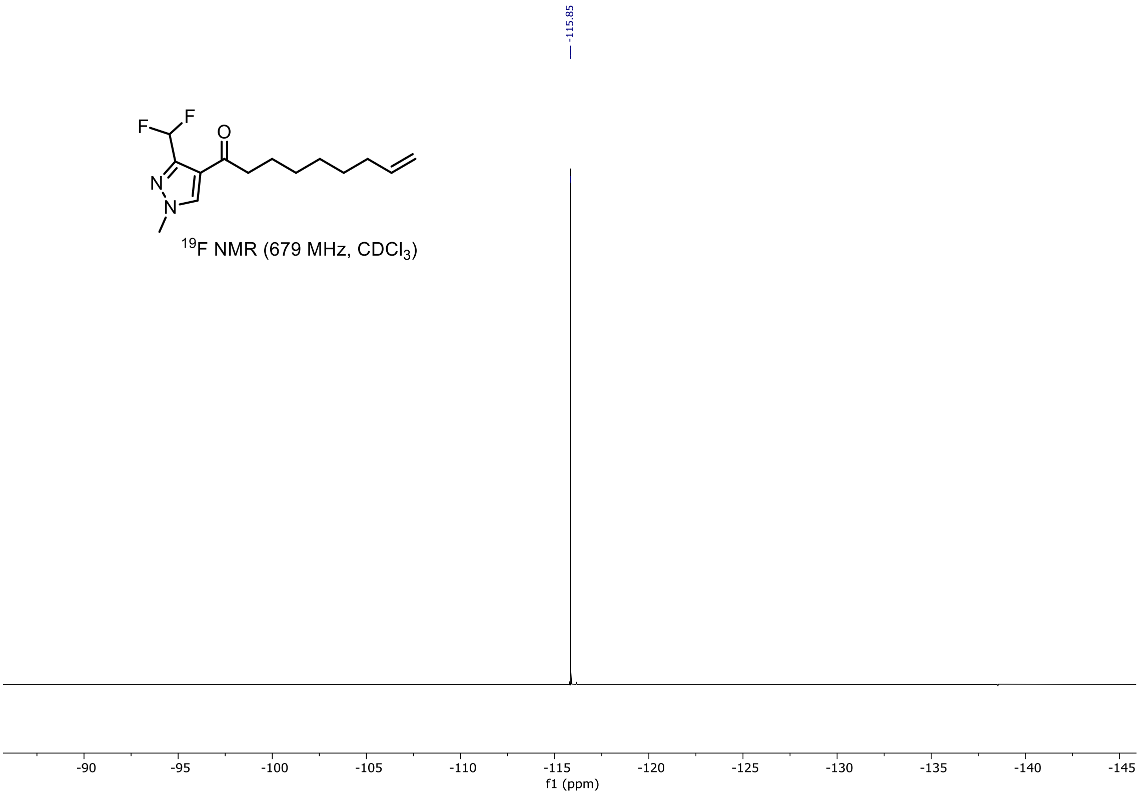
-115.85
F
F
O
N
N
19F NMR (679 MHz, CDCl3)
-90
-95
-100
-105
-110
-115
-120
-125
-130
-135
-140
-145
f1 (ppm)

Dec-9-en-2-one

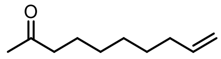

7-octenylmagnesium bromide solution was prepared following general procedure C using 8-bromo-1-octene (3.36 mL, 20.00 mmol, 1.00 equiv.) and magnesium (972 mg, 40.00 mmol, 2.00 equiv.).

The title compound was prepared following general procedure C using *N*-methoxy-*N*-methylacetamide (206 mg, 2.00 mmol, 1.00 equiv.) and a solution of 7-octenylmagnesium bromide (1.50 equiv.). Purification by flash column chromatography (0 – 10% EtOAc in heptanes) afforded the title compound as a colorless oil (151 mg, 0.98 mmol, 49%).

^{1}H NMR (700 MHz, $CDCl_3$) δ 5.79 (ddt, *J* = 16.9, 10.2, 6.7 Hz, 1H), 4.98 (ddd, *J* = 17.1, 3.6, 1.6 Hz, 1H), 4.93 (ddt, *J* = 10.2, 2.2, 1.2 Hz, 1H), 2.41 (t, *J* = 7.5 Hz, 2H), 2.13 (s, 3H), 2.06 – 2.01 (m, 2H), 1.60 – 1.54 (m, 2H), 1.40 – 1.35 (m, 2H), 1.33 – 1.26 (m, 4H).

^{13}C NMR (176 MHz, $CDCl_3$) δ 209.4 (C), 139.2 (CH), 114.4 (CH_2), 43.9 (CH_2), 33.8 (CH_2), 30.0 (CH_3), 29.1 (CH_2), 29.0 (CH_2), 28.9 (CH_2), 23.9 (CH_2).

IR (neat) v_{max}: 3077, 2927, 2855, 1715, 1640, 1413, 1359, 1163, 993, 909.

HRMS (EI$^+$): exact mass calculated for $[M–C_8H_{15}^{\cdot}]^+$ $(C_2H_3O)^+$ requires *m*/*z* 43.0178, found *m*/*z* 43.0182.

Dec-9-en-2-one

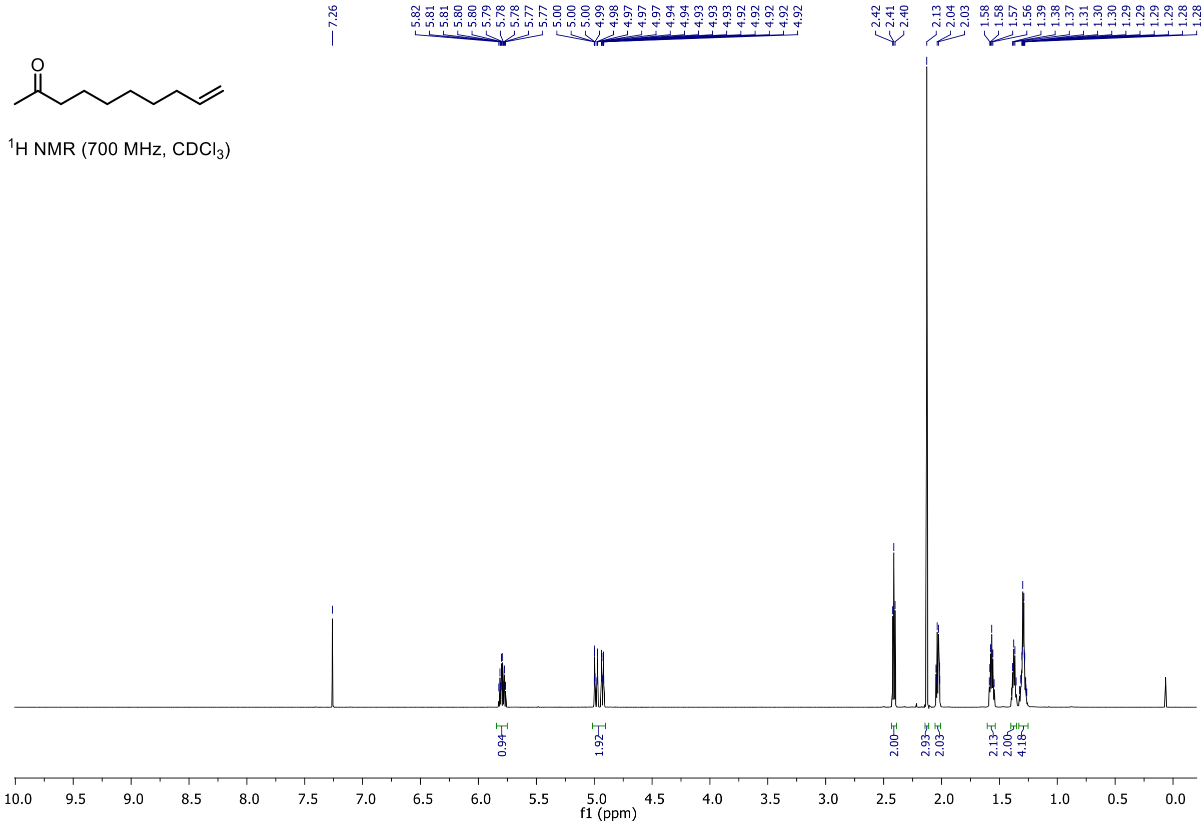
O
¹H NMR (700 MHz, CDCl3)
7.26
5.82
5.81
5.81
5.80
5.80
5.79
5.78
5.78
5.77
5.77
5.00
5.00
5.00
4.99
4.98
4.97
4.97
4.97
4.94
4.94
4.93
4.93
4.93
4.92
4.92
4.92
4.92
2.42
2.41
2.40
2.13
2.04
2.03
1.58
1.58
1.57
1.56
1.39
1.38
1.37
1.31
1.30
1.29
1.29
1.29
1.28
1.28
0.94
1.92
2.00
2.93
2.03
2.13
2.00
4.18
f1 (ppm)

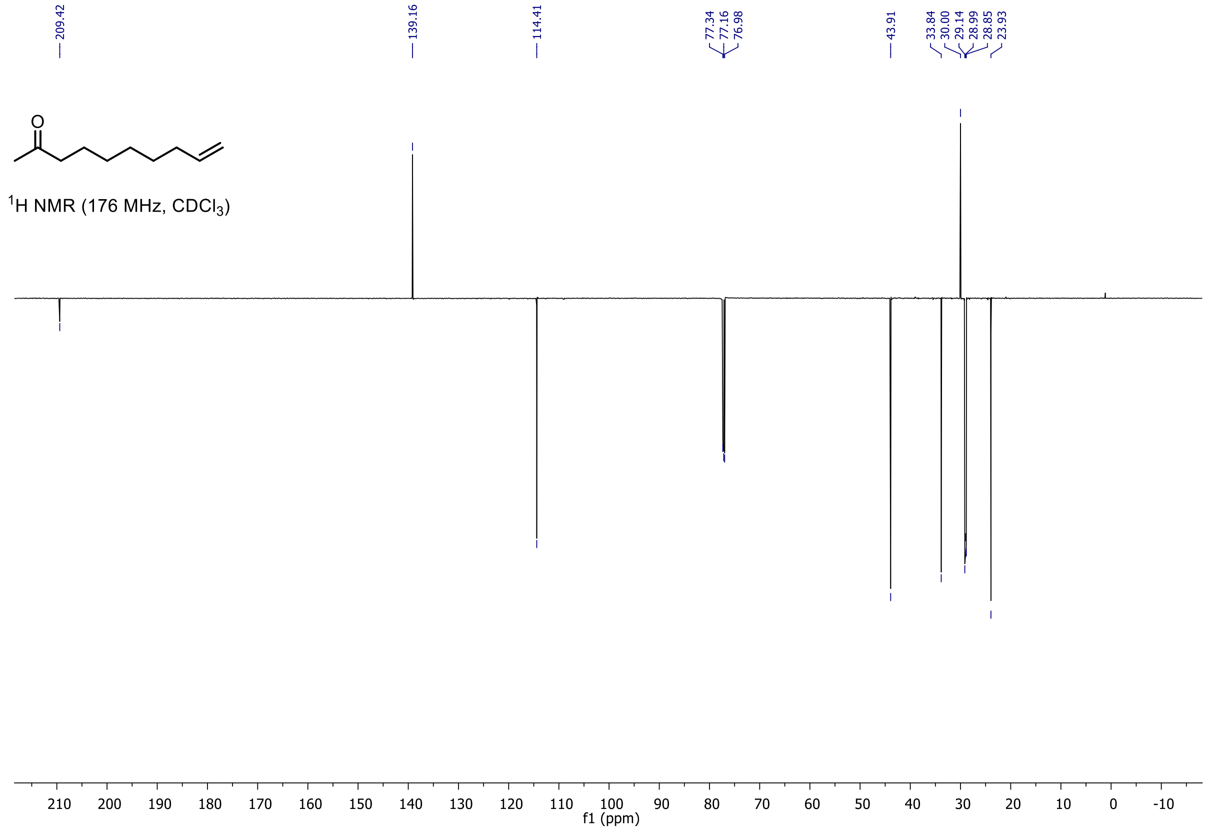
O
¹H NMR (176 MHz, CDCl3)
209.42
139.16
114.41
77.34
77.16
76.98
43.91
33.84
30.00
29.14
28.99
28.85
23.93
f1 (ppm)

Tetradec-13-en-5-one

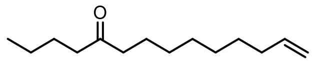

The title compound was prepared following general procedure C using *N*-methoxy-*N*-methyldec-9-enamide (309 mg, 3.00 mmol, 1.00 equiv.) and a solution of *n*-butylmagnesium bromide (1.50 equiv.). Purification by flash column chromatography (0 – 10% EtOAc in heptanes) afforded the title compound (112 mg, 0.53 mmol, 44%) as a colorless liquid.

^{1}H NMR (400 MHz, $CDCl_3$): δ 5.88 – 5.70 (m, 1H), 5.05 – 4.86 (m, 2H), 2.47 – 2.29 (m, 4H), 2.10 – 1.92 (m, 2H), 1.60 – 1.51 (m, 4H), 1.41 – 1.26 (m, 10H), 0.90 (t, *J* = 7.3 Hz, 3H).

^{13}C NMR (101 MHz, $CDCl_3$): δ 211.8, 139.3, 114.3, 43.0, 42.7, 33.9, 29.4, 29.4, 29.1, 29.0, 26.1, 24.0, 22.5, 14.0.

IR (neat) ν_{max}: 2956, 2926, 2855, 1713, 1463, 1411, 1370, 994, 908.

HRMS (ESI$^+$): exact mass calculated for $[M+H]^+$ $(C_{14}H_{27}O)^+$ requires *m/z* 211.2056, found *m/z* 211.2050.

Tetradec-13-en-5-one

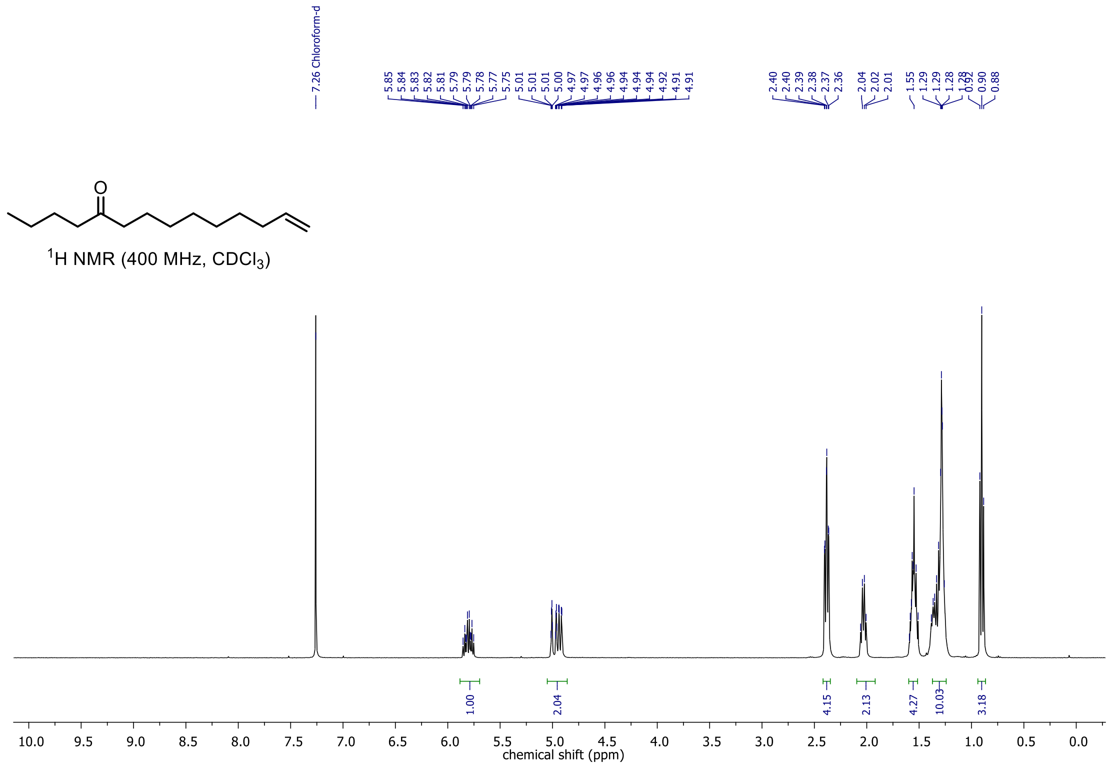

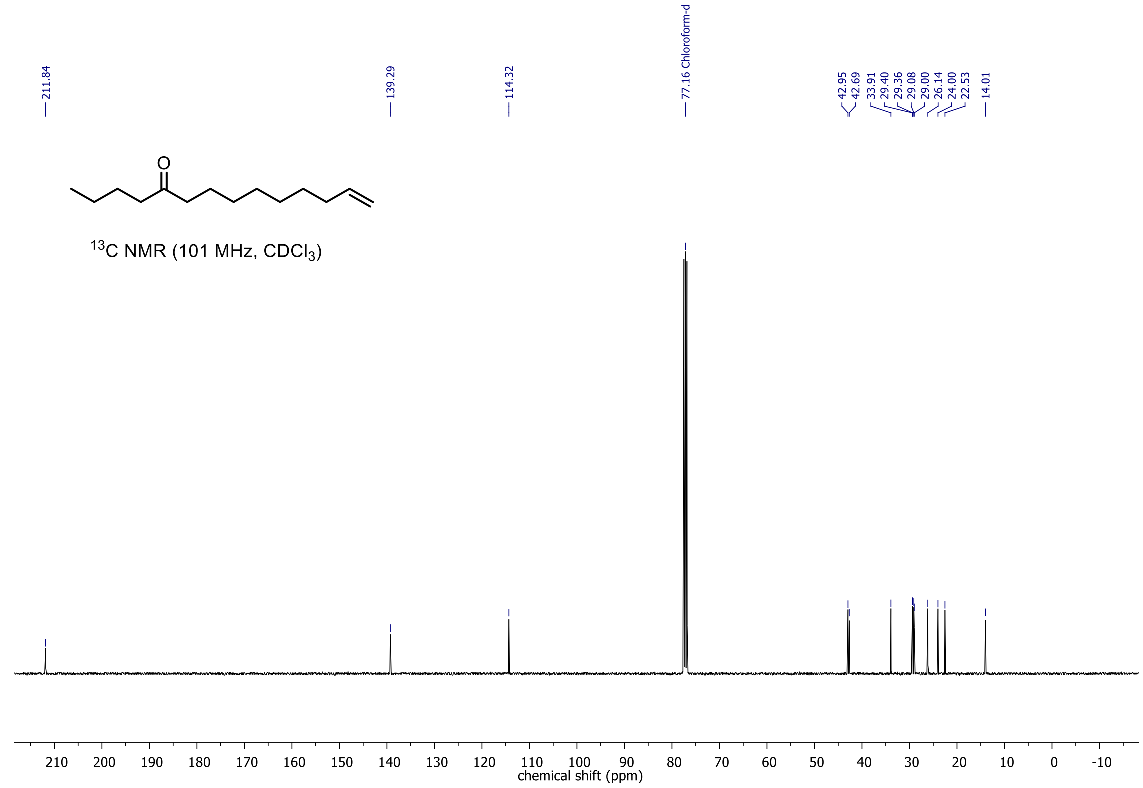

1-Cyclohexyldec-9-en-1-one

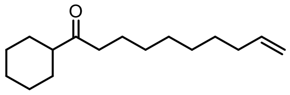

Cyclohexylmagnesium bromide solution was prepared following general procedure C using bromocyclohexane (0.37 mL, 3.00 mmol, 1.00 equiv.) and magnesium (146 mg, 6.00 mmol, 2.00 equiv.).

The title compound was prepared following general procedure C using Weinreb amide *N*-methoxy-*N*-methyldec-9-enamide (427 mg, 2.00 mmol, 1.00 equiv.) and a solution of cyclohexylmagnesium bromide (1.50 equiv.). Purification by flash column chromatography (0 – 10% EtOAc in heptanes) afforded the title compound as a colorless oil (111 mg, 0.47 mmol, 24%).

^{1}H NMR (400 MHz, $CDCl_3$) δ 5.80 (ddt, *J* = 16.9, 10.2, 6.7 Hz, 1H), 4.98 (ddd, *J* = 17.0, 3.7, 1.6 Hz, 1H), 4.92 (ddt, *J* = 10.2, 2.3, 1.2 Hz, 1H), 2.41 (t, *J* = 7.4 Hz, 2H), 2.32 (tt, *J* = 11.3, 3.4 Hz, 1H), 2.08 – 1.99 (m, 2H), 1.86 – 1.73 (m, 4H), 1.70 – 1.63 (m, 1H), 1.58 – 1.50 (m, 2H), 1.40 – 1.22 (m, 13H).

^{13}C NMR (151 MHz, $CDCl_3$) δ 214.6 (C), 139.3 (CH), 114.3 (CH_2), 51.0 (CH), 40.8 (CH_2), 33.9 (CH_2), 29.44 (CH_2), 29.43 (CH_2), 29.1 (CH_2), 29.0 (CH_2), 28.7 ($2CH_2$), 26.0 (CH_2), 25.9 ($2CH_2$), 23.9 (CH_2).

IR (neat) v_{max}: 3075, 2924, 2852, 1707, 1640, 1449, 993, 908, 737.

HRMS (ESI$^+$): exact mass calculated for $[M+H]^+$ $(C_{16}H_{29}O)^+$ requires *m*/*z* 237.2213, found *m*/*z* 237.2207.

1-Cyclohexyldec-9-en-1-one

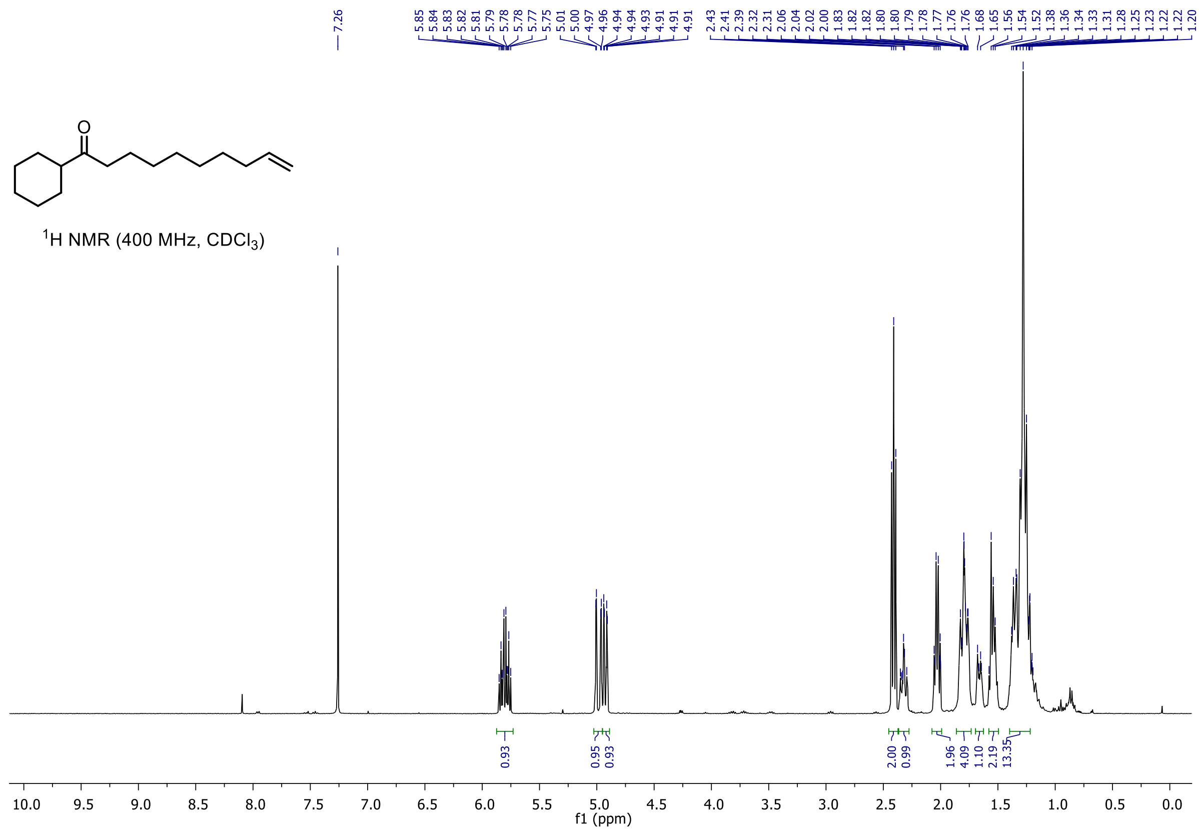

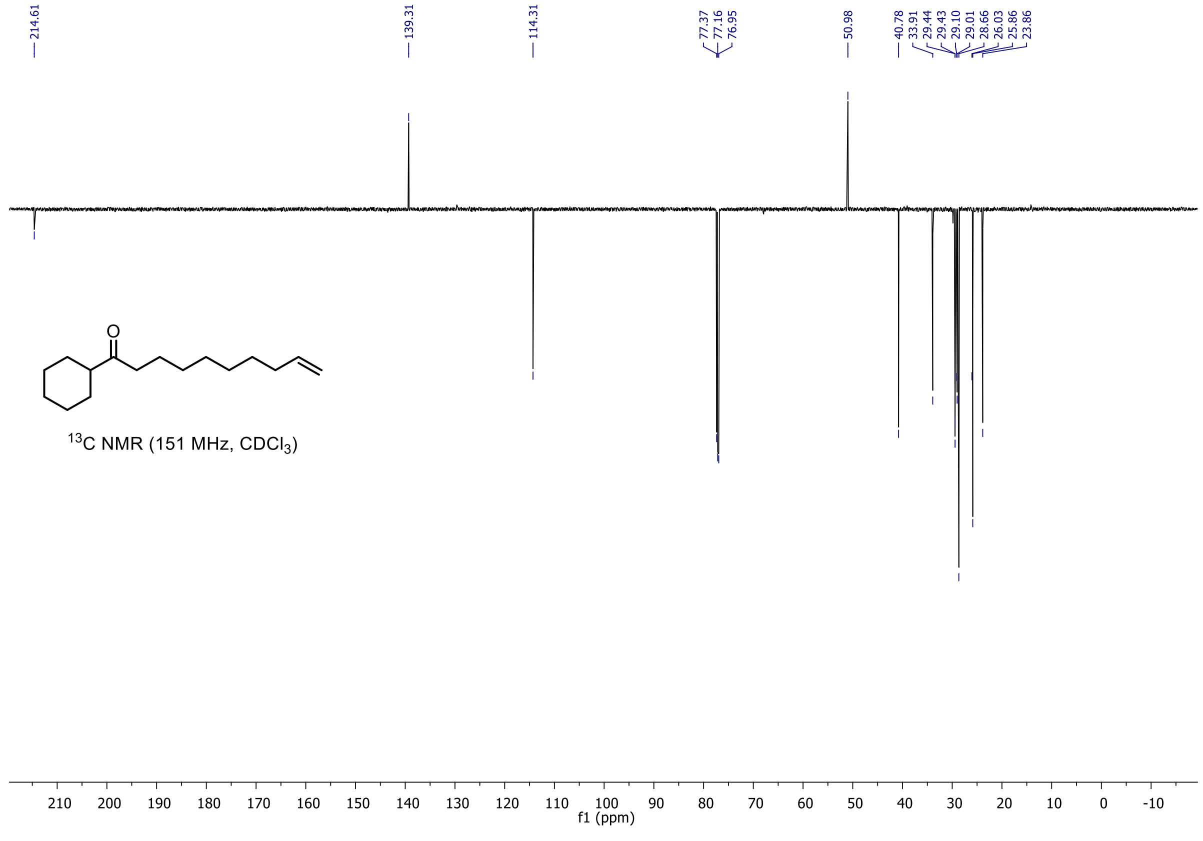

1-(Adamantan-1-yl)non-8-en-1-one

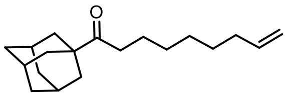

The title compound was prepared following general procedure C using *N*-methoxy-*N*-methyladamantane-1-carboxamide (411 mg, 2.00 mmol, 1.00 equiv.) and a solution of 7-octenylmagnesium bromide (1.50 equiv.). Purification by flash column chromatography (0 – 20% EtOAc in heptaness) afforded the title compound (170 mg, 0.62 mmol, 34%) as a colorless liquid.

^{1}H NMR (400 MHz, $CDCl_3$): δ 5.87 – 5.73 (m, 1H), 5.02 – 4.89 (m, 2H), 2.42 (t, *J* = 7.3 Hz, 2H), 2.11 – 1.98 (m, 5H), 1.82 – 1.79 (m, 5H), 1.71 (m, 6H), 1.55 – 1.49 (m, 2H), 1.43 – 1.21 (m, 7H).

^{13}C NMR (101 MHz, $CDCl_3$): δ 216.0, 139.3, 114.4, 46.5, 38.4 (3C), 36.8 (3C), 36.0, 33.9, 29.4, 29.1, 28.9, 28.1 (3C), 23.8.

IR (neat) ν_{max}: 3075, 2903, 2850, 2677, 1639, 1409, 1345, 1266, 1199, 1103, 1050, 995, 908, 737.

HRMS (ESI$^+$): exact mass calculated for $[M+H]^+$ $(C_{19}H_{31}O)^+$ requires *m/z* 275.2369, found *m/z* 275.2363.

1-(Adamantan-1-yl)non-8-en-1-one

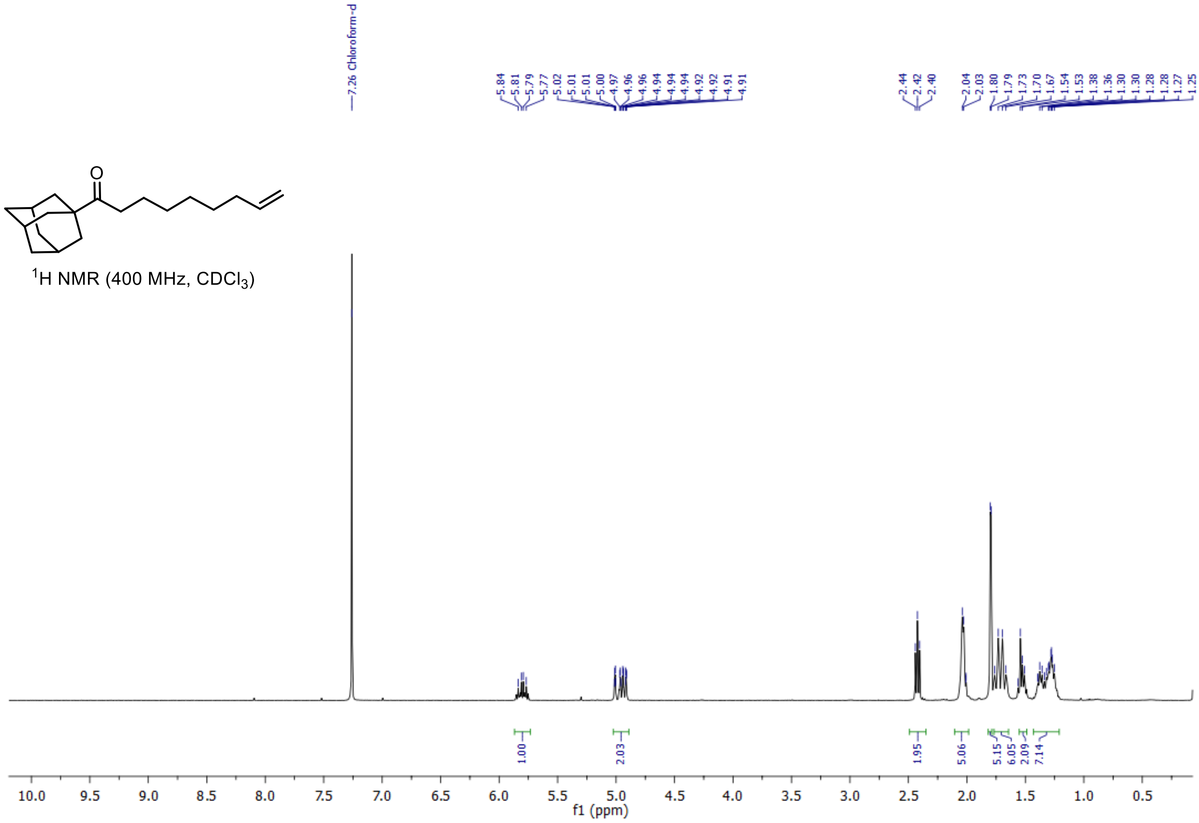

^{1}H NMR (400 MHz, $CDCl_3$)

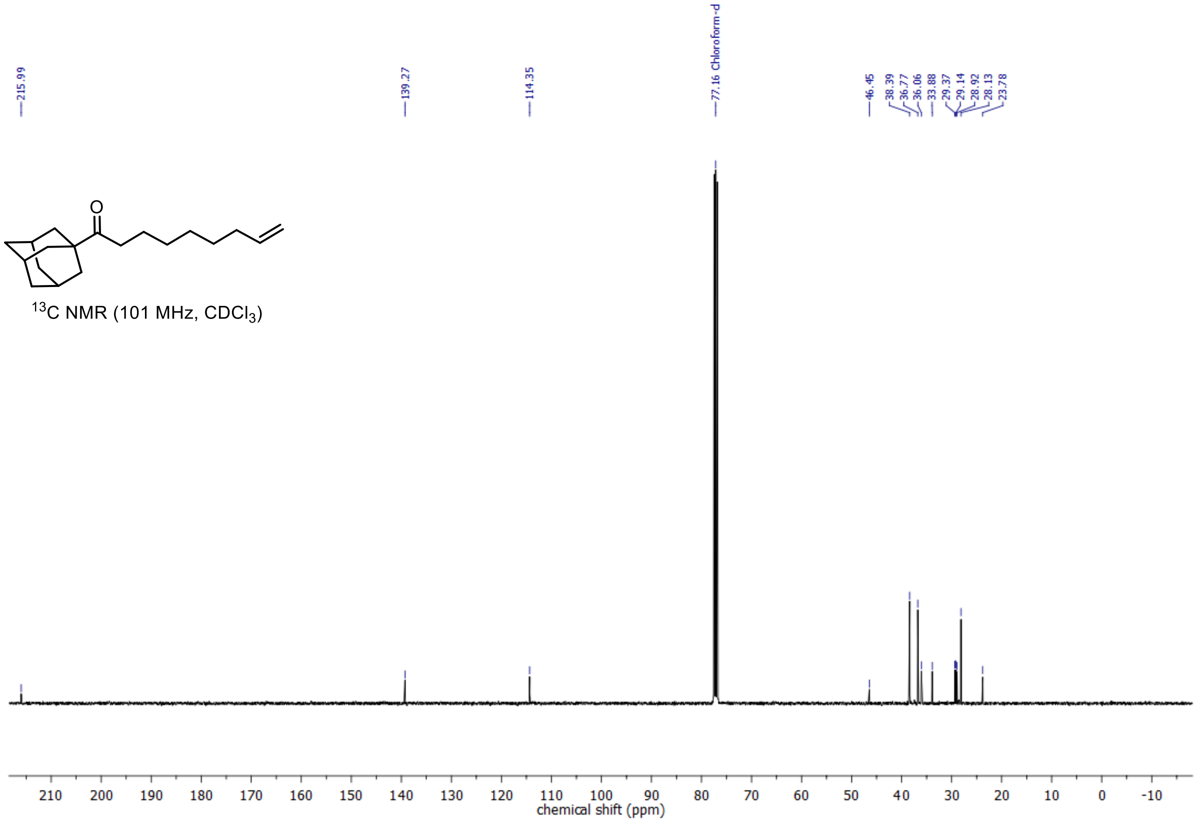

^{13}C NMR (101 MHz, $CDCl_3$)

1-(4,5-Diphenyloxazol-2-yl)undec-10-en-3-one

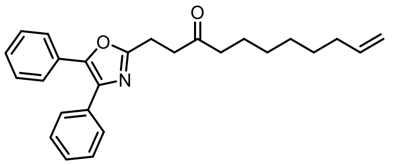

The title compound was prepared following general procedure C using 3-(4,5-diphenyloxazol-2-yl)-*N*-methoxy-*N*-methylpropanamide (673 mg, 2.00 mmol, 1.00 equiv.) and a solution of 7-octenylmagnesium bromide (1.50 equiv.). Purification by flash column chromatography (0 – 20% EtOAc in heptaness) afforded the title compound (409 mg, 1.06 mmol, 53%) as a colorless liquid.

^{1}H NMR (400 MHz, $CDCl_3$): δ 7.59 (m, 4H), 7.41 – 7.28 (m, 6H), 5.79 (ddt, *J* = 16.9, 10.2, 6.7 Hz, 1H), 5.04 – 4.89 (m, 2H), 3.18 – 2.97 (m, 4H), 2.50 (t, *J* = 7.4 Hz, 2H), 2.03 (m, 2H), 1.70 – 1.57 (m, 2H), 1.41 – 1.27 (m, 6H).

^{13}C NMR (101 MHz, $CDCl_3$): δ 209.0, 162.6, 145.5, 139.2, 135.2, 132.7, 129.2, 128.8 (2C), 128.7 (2C), 128.5, 128.2, 128.1 (2C), 126.6 (2C), 114.4, 43.1, 39.1, 33.9, 29.2, 29.0, 28.8, 24.0, 22.3.

IR (neat) ν_{max}: 2926, 2854, 1714, 1502, 1360, 1213, 1058, 910, 762, 692, 587.

HRMS (ESI$^+$): exact mass calculated for $[M+H]^+$ $(C_{26}H_{30}NO_2)^+$ requires *m/z* 388.2271, found *m/z* 388.2267.

1-(4,5-Diphenyloxazol-2-yl)undec-10-en-3-one

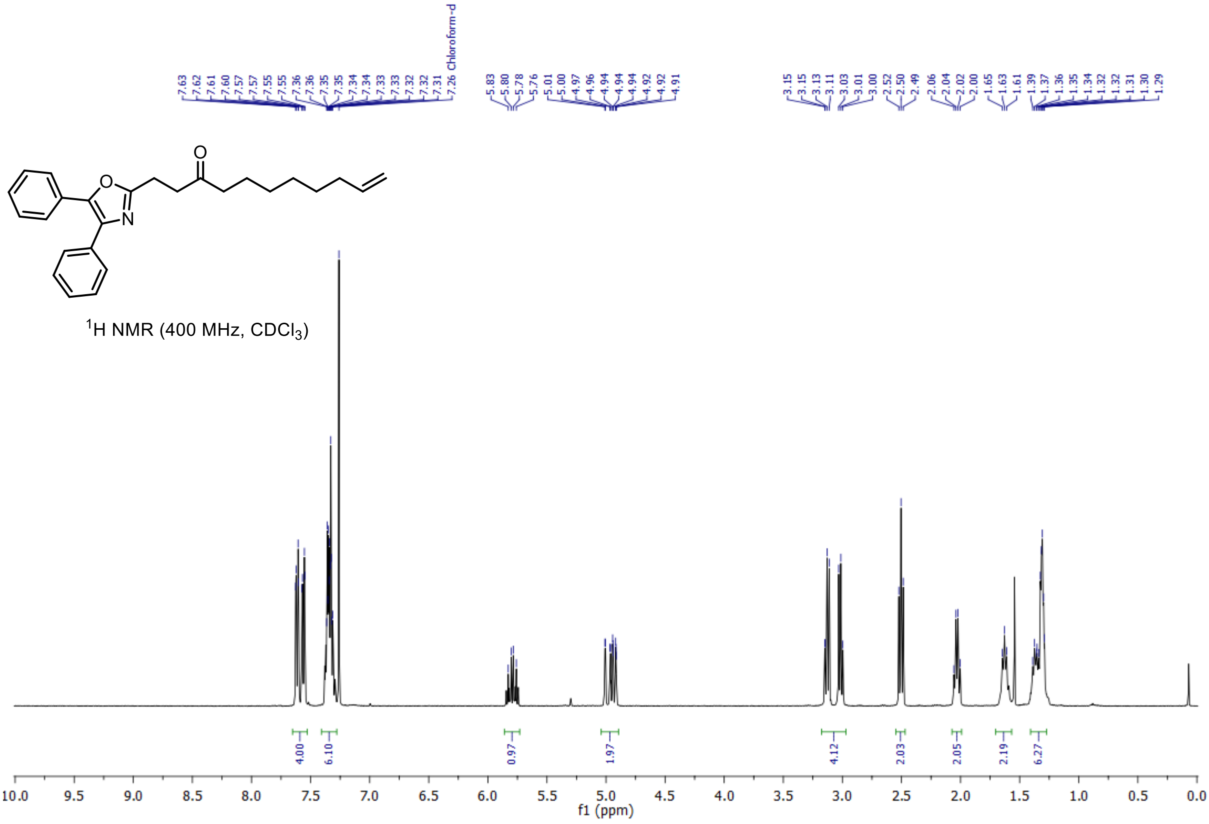

^{1}H NMR (400 MHz, $CDCl_3$)

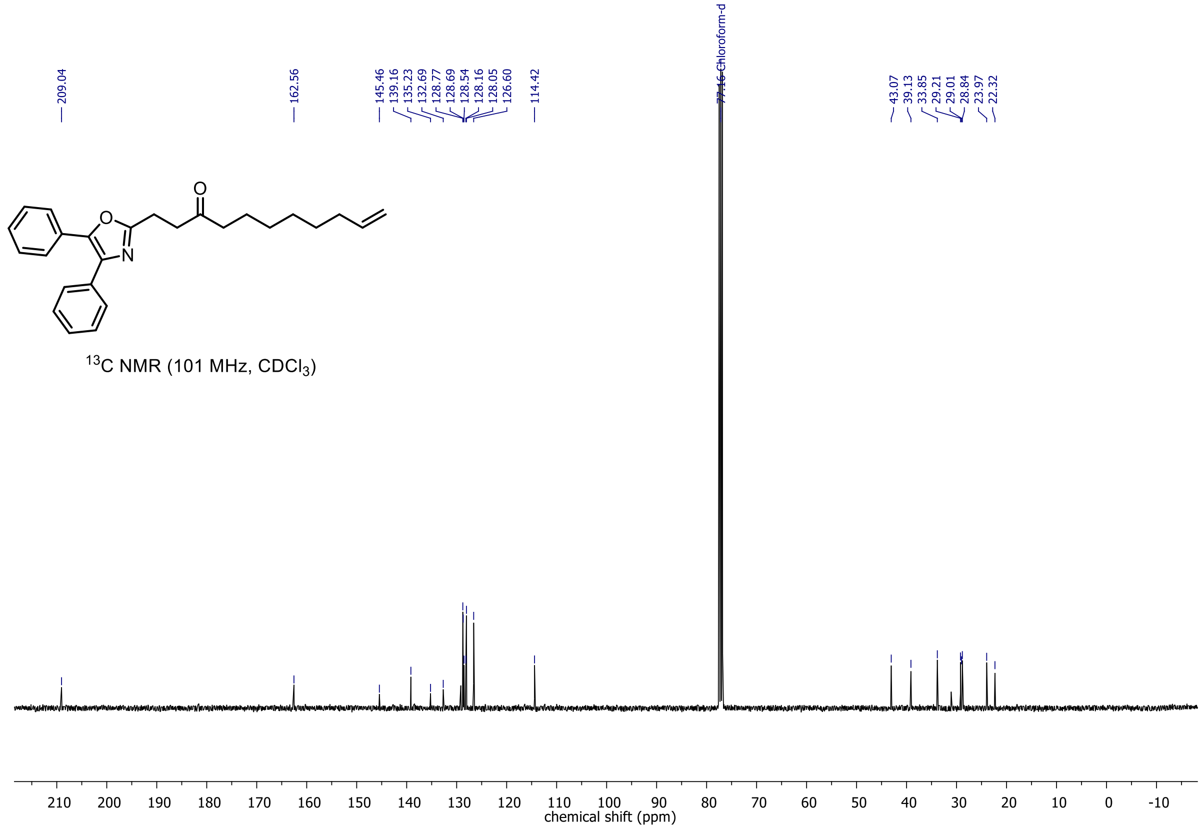

^{13}C NMR (101 MHz, $CDCl_3$)

4-(Non-8-enoyl)-*N,N*-dipropylbenzenesulfonamide

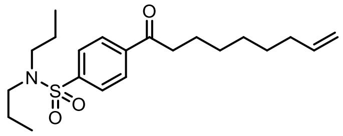

The title compound was prepared following general procedure C using 4-(*N*,*N*-dipropylsulfamoyl)-*N*-methoxy-*N*-methylbenzamide (657 mg, 2.00 mmol, 1.00 equiv.) and a solution of 7-octenylmagnesium bromide (1.50 equiv.). Purification by flash column chromatography (0 – 20% EtOAc in heptanes) afforded the title compound (454 mg, 1.20 mmol, 60%) as a colorless solid.

^{1}H NMR (400 MHz, $CDCl_3$): δ 8.07 – 8.02 (m, 2H), 7.91 – 7.86 (m, 2H), 5.80 (ddt, *J* = 16.9, 10.2, 6.7 Hz, 1H), 5.05 – 4.90 (m, 2H), 3.13 – 3.06 (m, 4H), 2.98 (t, *J* = 7.3 Hz, 2H), 2.10 – 2.01 (m, 2H), 1.80 – 1.70 (m, 2H), 1.60 – 1.50 (m, 4H), 1.44 – 1.34 (m, 6H), 0.87 (t, *J* = 7.4 Hz, 6H).

^{13}C NMR (101 MHz, $CDCl_3$): δ 199.5, 140.1, 139.9, 139.1, 128.7 (2C), 127.4 (2C), 114.5, 50.1 (2C), 39.1, 33.8, 29.2, 29.0, 28.9, 24.2, 22.1 (2C), 11.3 (2C).

IR (neat) ν_{max}: 3078, 2964, 2932, 2873, 1682, 1595, 1467, 1304, 1266, 1223, 1156, 1017, 901, 827, 731, 671, 572.

HRMS (ESI^{+}): exact mass calculated for $[M+H]^+$ $(C_{21}H_{34}NO_3S)^+$ requires *m/z* 380.2254, found *m/z* 380.2254.

4-(Non-8-enoyl)-*N*,*N*-dipropylbenzenesulfonamide

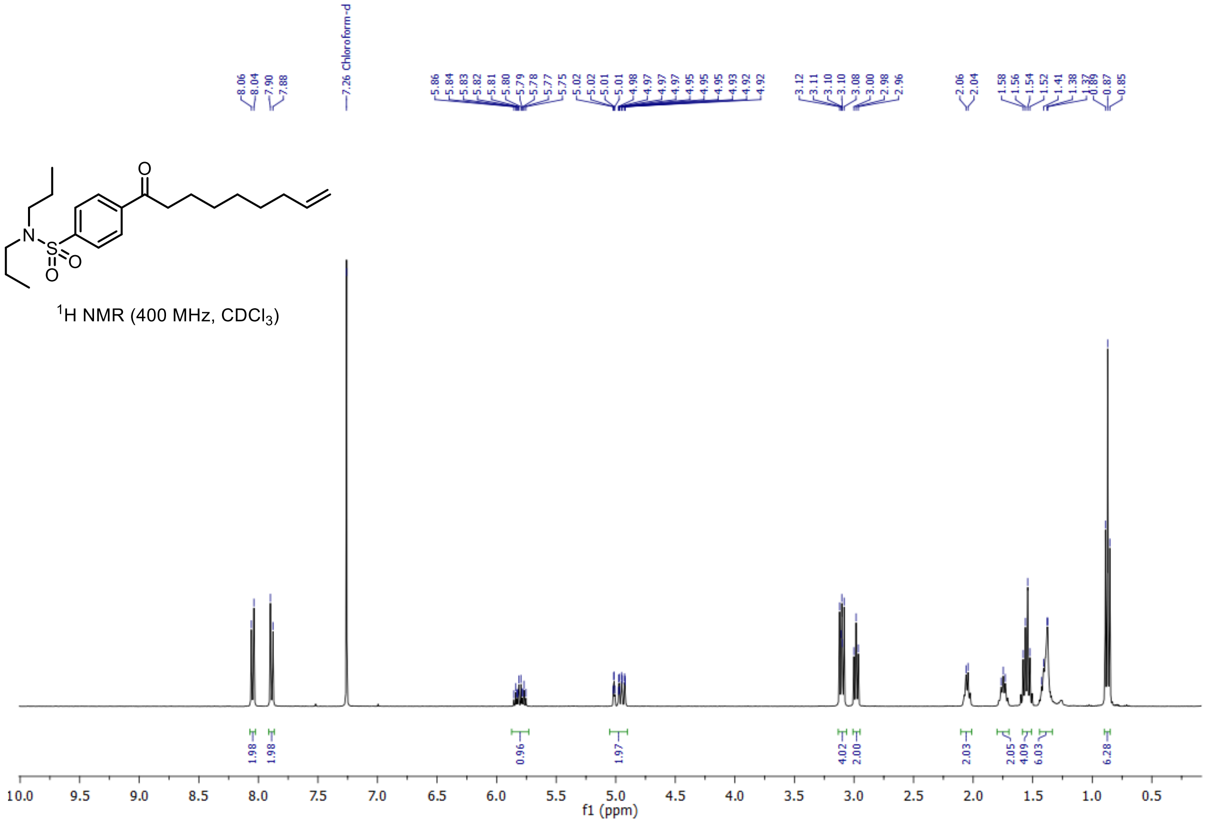

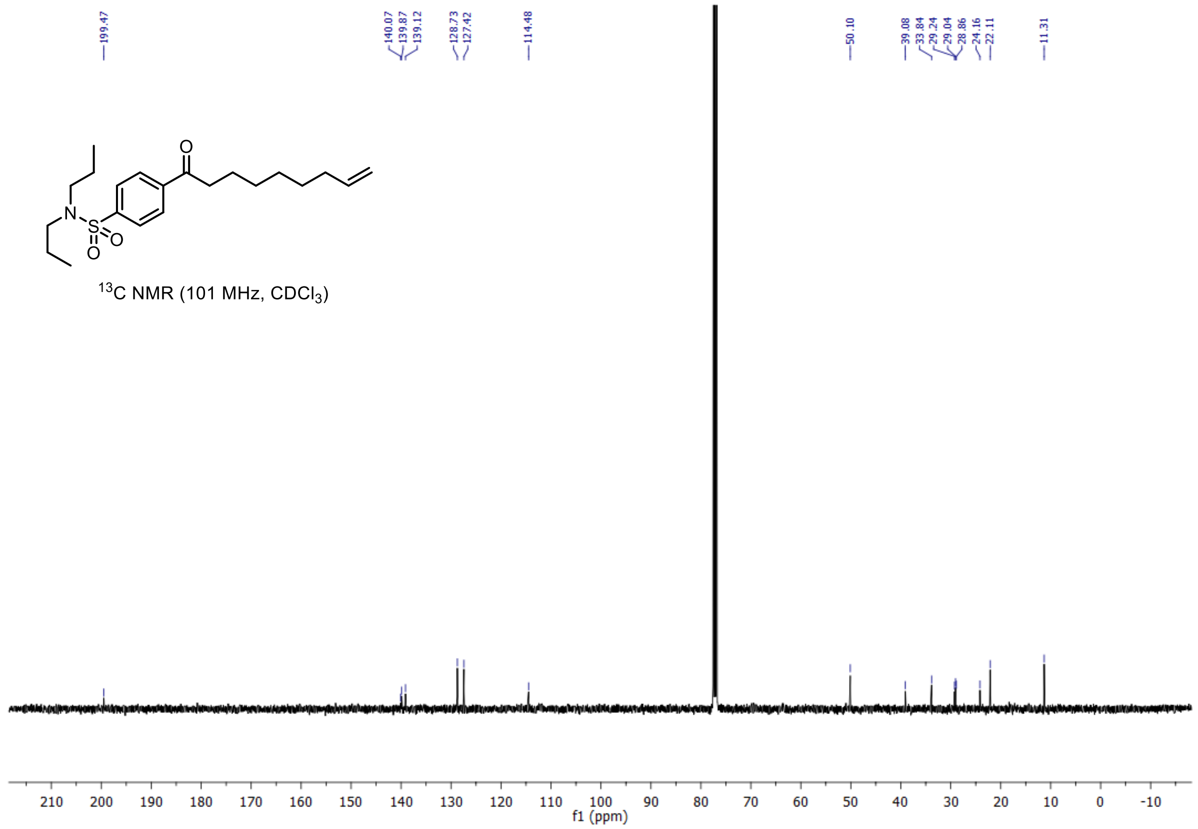

1-(2,3,6,7-Tetrahydro-1*H*,5*H*-pyrido[3,2,1-*iJ*]quinolin-9-yl)non-8-en-1-ol

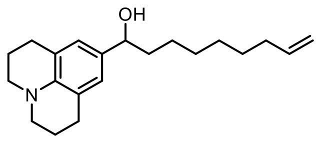

The title compound was prepared following general procedure D using 9-julolidinecarboxaldehyde (411 mg, 2.00 mmol, 1.00 equiv.) and a solution of 7-octenylmagnesium bromide (4.00 equiv.). Purification by flash column chromatography (0 – 100% EtOAc in heptanes) afforded the title compound (445 mg, 1.42 mmol, 71%) as dark red oil.

^{1}H NMR (400 MHz, $CDCl_3$): δ 6.76 (s, 2H), 5.80 (ddt, *J* = 16.9, 10.2, 6.7 Hz, 1H), 5.12 – 4.80 (m, 2H), 4.50 – 4.40 (m, 1H), 3.20 – 3.04 (m, 4H), 2.83 – 2.68 (m, 4H), 2.07 – 1.93 (m, 6H), 1.82 – 1.72 (m, 1H), 1.70 – 1.60 (m, 1H), 1.59 (m, 1H), 1.44 – 1.26 (m, 8H).

^{13}C NMR (101 MHz, $CDCl_3$): δ 142.6, 139.4, 132.2, 124.9 (2C), 121.6 (2C), 114.3, 74.8, 50.2 (2C), 38.7, 33.9, 29.6, 29.2, 29.0, 27.8 (2C), 26.3, 22.3 (2C).

IR (neat) ν_{max}: 3393, 2924, 2852, 1611, 1446, 1306, 1205, 115, 1053, 906, 735, 641.

HRMS (ESI$^+$): exact mass calculated for $[M+H]^+$ $(C_{21}H_{32}NO)^+$ requires *m/z* 314.2478, found *m/z* 314.2474.

1-(2,3,6,7-Tetrahydro-1*H*,5*H*-pyrido[3,2,1-*iJ*]quinolin-9-yl)non-8-en-1-ol

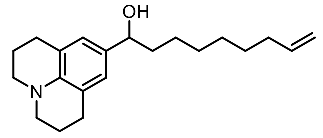

^{1}H NMR (400 MHz, $CDCl_3$)

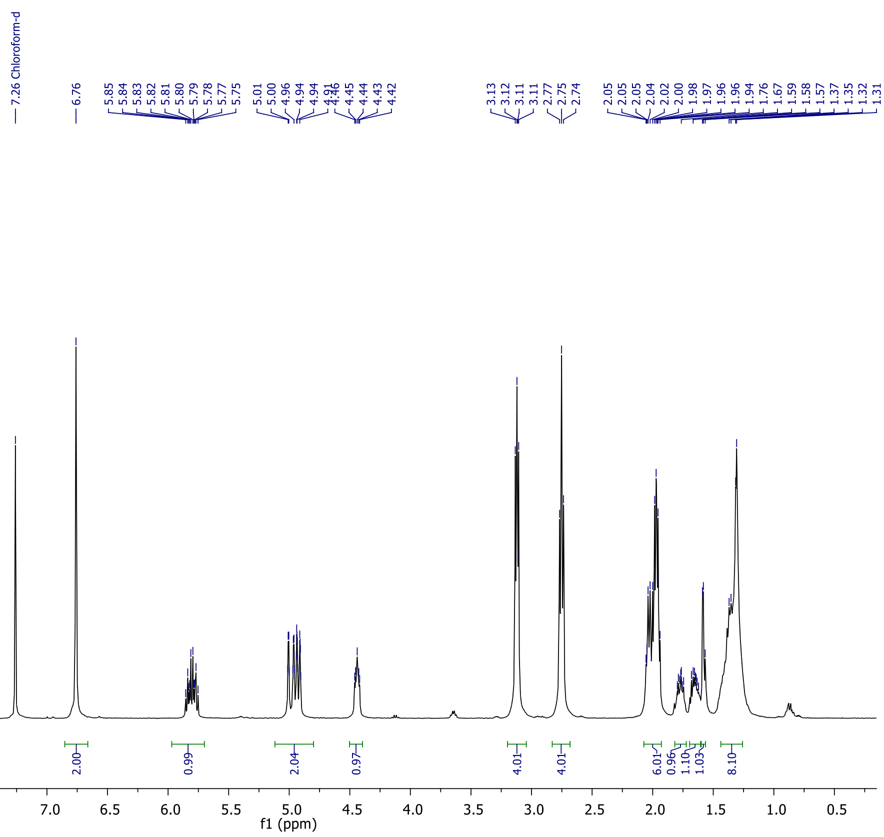

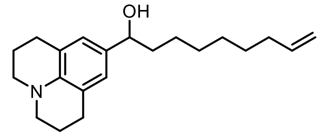

^{13}C NMR (101 MHz, $CDCl_3$)

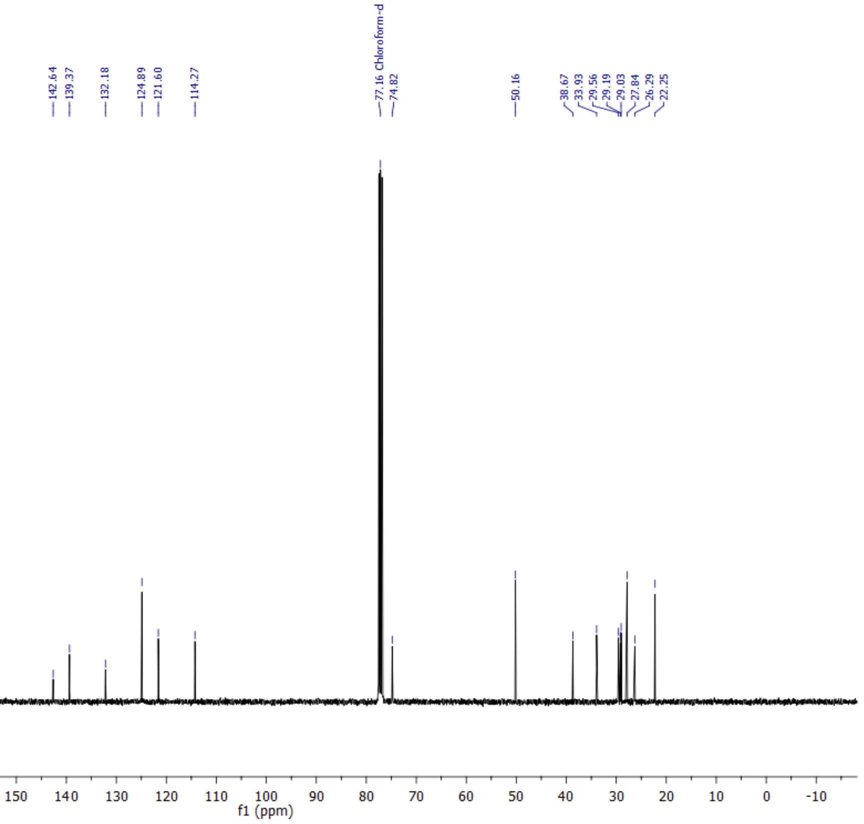

1-(2,3,6,7-Tetrahydro-1*H*,5*H*-pyrido[3,2,1-*ij*]quinolin-9-yl)non-8-en-1-one

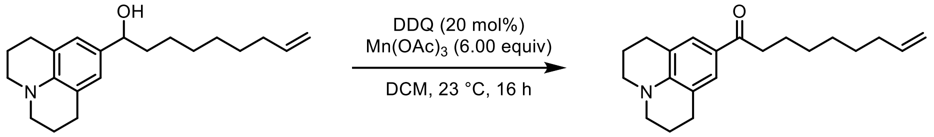

The compound was prepared according to a literature procedure.[12]

The compound 1-(2,3,6,7-Tetrahydro-1*H*,5*H*-pyrido[3,2,1-*iJ*]quinolin-9-yl)non-8-en-1-ol (157 mg, 0.50 mmol, 1.00 equiv.) was dissolved in DCM (2 mL) under air at 23 °C. $Mn(OAc)_3 \cdot 2\ H_2O$ (804 mg, 3.00 mmol, 6.00 equiv) was added in a single portion, followed by DDQ (22.7 mg, 0.10 mmol, 20 mol%). The mixture was stirred 16 h and then filtered through a pad of Celite (eluting with DCM, 3 mL). The filtrate was washed with saturated aqueous $NaHCO_3$ solution (5 mL), dried over anhydrous sodium sulfate, the dried solution was filtered and the filtrate was concentrated under reduced pressure. Purification by flash column chromatography (0 – 20% EtOAc in heptanes) afforded the title compound (42.2 mg, 0.14 mmol, 37%) as a yellow liquid.

^{1}H NMR (400 MHz, $CDCl_3$): δ 7.43 (s, 2H), 5.78 (ddt, *J* = 17.0, 10.2, 6.7 Hz, 1H), 4.93 (m, 2H), 3.27 – 3.19 (m, 4H), 2.82 – 2.71 (m, 6H), 2.09 – 1.99 (m, 2H), 1.97 – 1.90 (m, 4H), 1.76 – 1.60 (m, 2H), 1.38 – 1.29 (m, 6H).

^{13}C NMR (101 MHz, $CDCl_3$): δ 199.0, 146.7, 139.3, 128.0 (2C), 124.1, 120.0 (2C), 114.3, 50.1 (2C), 37.9, 33.9, 29.6, 29.2, 29.0, 27.9 (2C), 25.4, 21.7 (2C).

IR (neat) ν_{max}: 2927, 2852, 1659, 1597, 1572, 1519, 1437, 1313, 1162, 910, 737.

HRMS (ESI$^+$): exact mass calculated for $[M+H]^+$ $(C_{21}H_{30}NO)^+$ requires *m/z* 312.2322, found *m/z* 312.2321.

1-(2,3,6,7-Tetrahydro-1*H*,5*H*-pyrido[3,2,1-*ij*]quinolin-9-yl)non-8-en-1-one

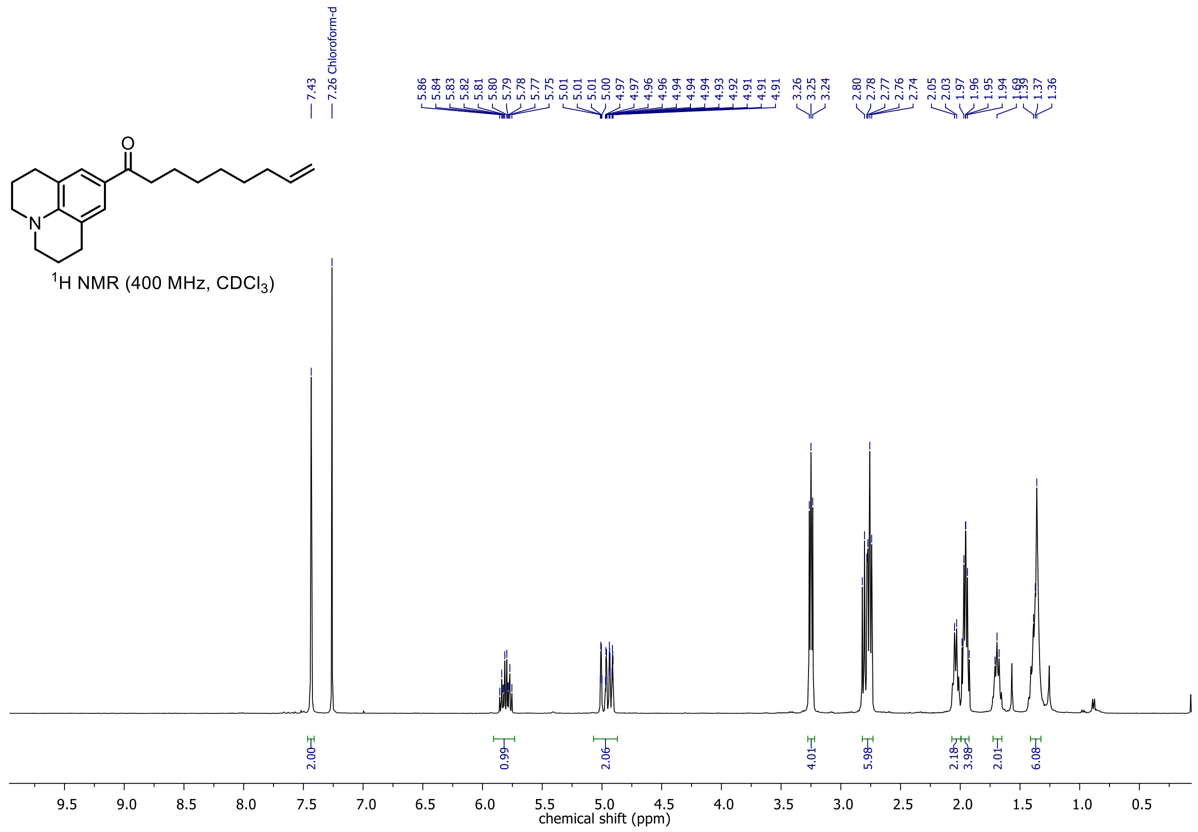

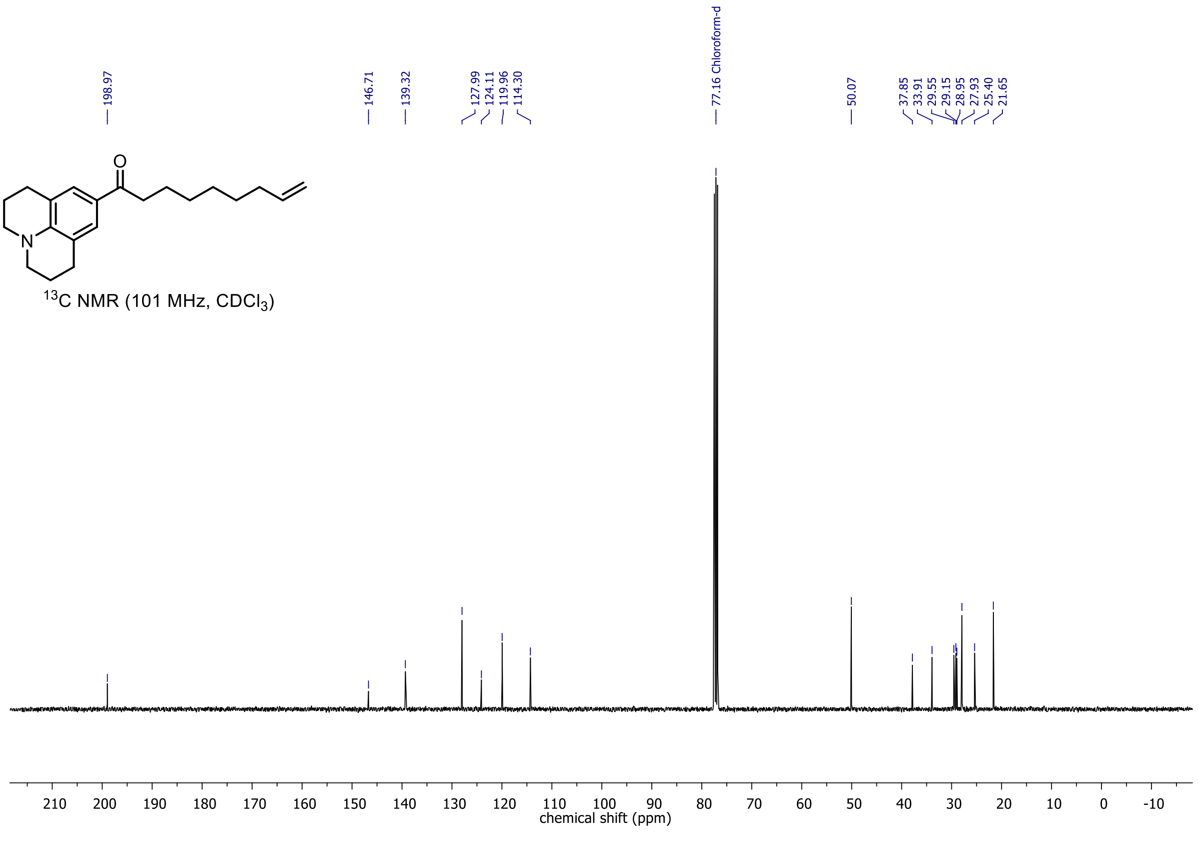

1-(1-(3-(Dimethylamino)propyl)-1-(4-fluorophenyl)-1,3-dihydroisobenzofuran-5-yl)non-8-en-1-one

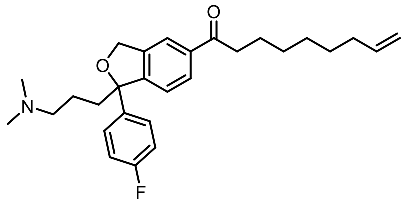

The title compound was prepared following general procedure E using citalopram hydrobromide (2.59 g, 2.00 mmol, 1.00 equiv.) and a solution of 7-octenylmagnesium bromide (6.00 equiv.). Purification by flash column chromatography (0 – 100% EtOAc/EtOH/28% NH_4OH (3:1:0.02) in heptanes) afforded the title compound (459 mg, 1.05 mmol, 52%) as yellow oil.

^{1}H NMR (400 MHz, $CDCl_3$): δ 7.89 (d, *J* = 7.9 Hz, 1H), 7.80 (s, 1H), 7.49 – 7.43 (m, 2H), 7.38 (d, *J* = 8.0 Hz, 1H), 7.02 – 6.97 (m, 2H), 5.80 (ddt, *J* = 16.9, 10.2, 6.7 Hz, 1H), 5.23 - 5.14 (m, 2H), 5.05 – 4.87 (m, 2H), 2.93 (t, *J* = 7.4 Hz, 2H), 2.36 (m, 2H), 2.30 – 2.14 (m, 8H), 2.11 – 1.99 (m, 2H), 1.77 – 1.67 (m, 2H), 1.60 – 1.47 (m, 1H), 1.39 (m, 7H).

^{13}C NMR (101 MHz, $CDCl_3$): δ 200.0, 162.1 (d, *J* = 245.5 Hz), 149.2, 140.2 (d, *J* = 3.2 Hz), 139.9, 139.2, 137.2, 128.1, 127.0 (d, *J* = 8.2 Hz, 2C), 122.1, 121.2, 115.4 (d, *J* = 21.6 Hz, 2C), 114.4, 91.0, 71.8, 59.5, 45.1 (2C), 39.1, 38.9, 33.9, 29.3, 29.1, 28.9, 24.4, 22.0.

^{19}F NMR (565 MHz, $CDCl_3$) δ -115.74.

IR (neat) $ν_{max}$: 2928, 2855, 1681, 1601, 1505, 1222, 1157, 1030, 909, 831, 735, 531.

HRMS (ESI$^+$): exact mass calculated for $[M+H]^+$ $(C_{28}H_{37}FNO_2)^+$ requires *m/z* 438.2803, found *m/z* 438.2800.

1-(1-(3-(Dimethylamino)propyl)-1-(4-fluorophenyl)-1,3-dihydroisobenzofuran-5-yl)non-8-en-1-one

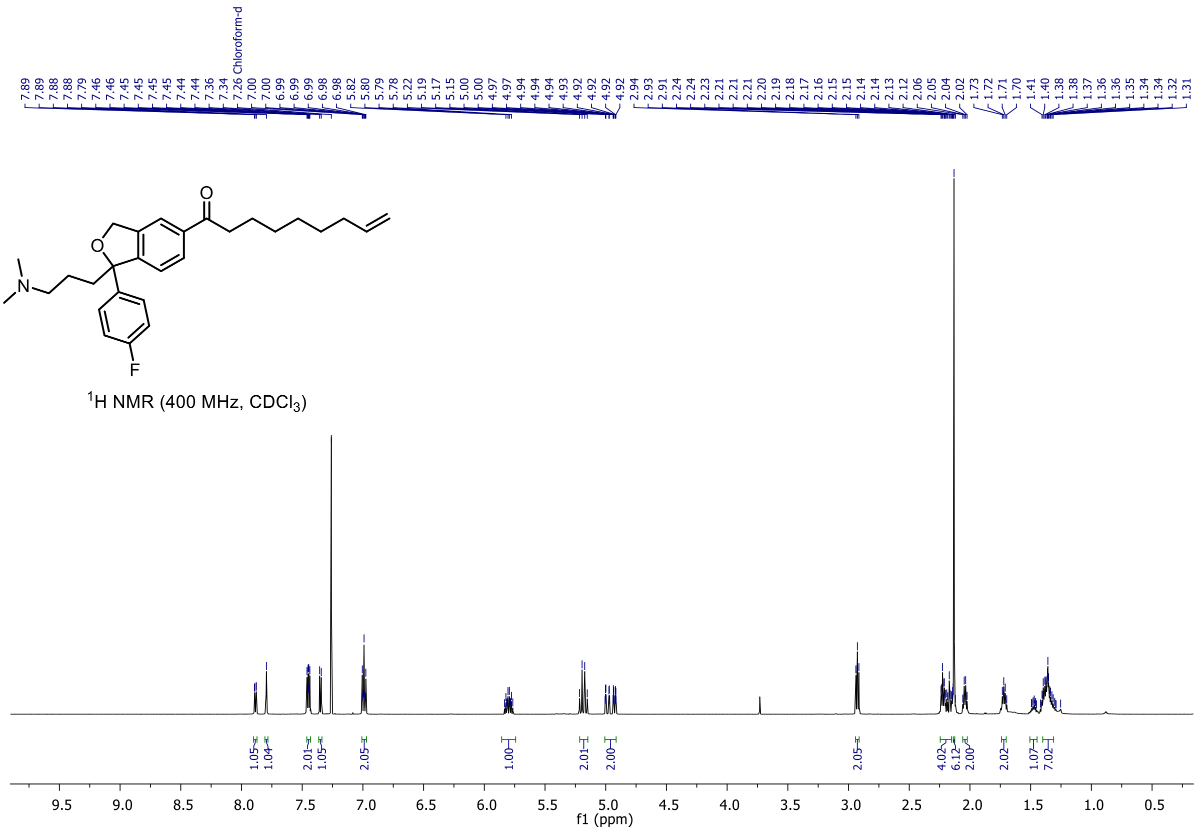

^{1}H NMR (400 MHz, $CDCl_3$)

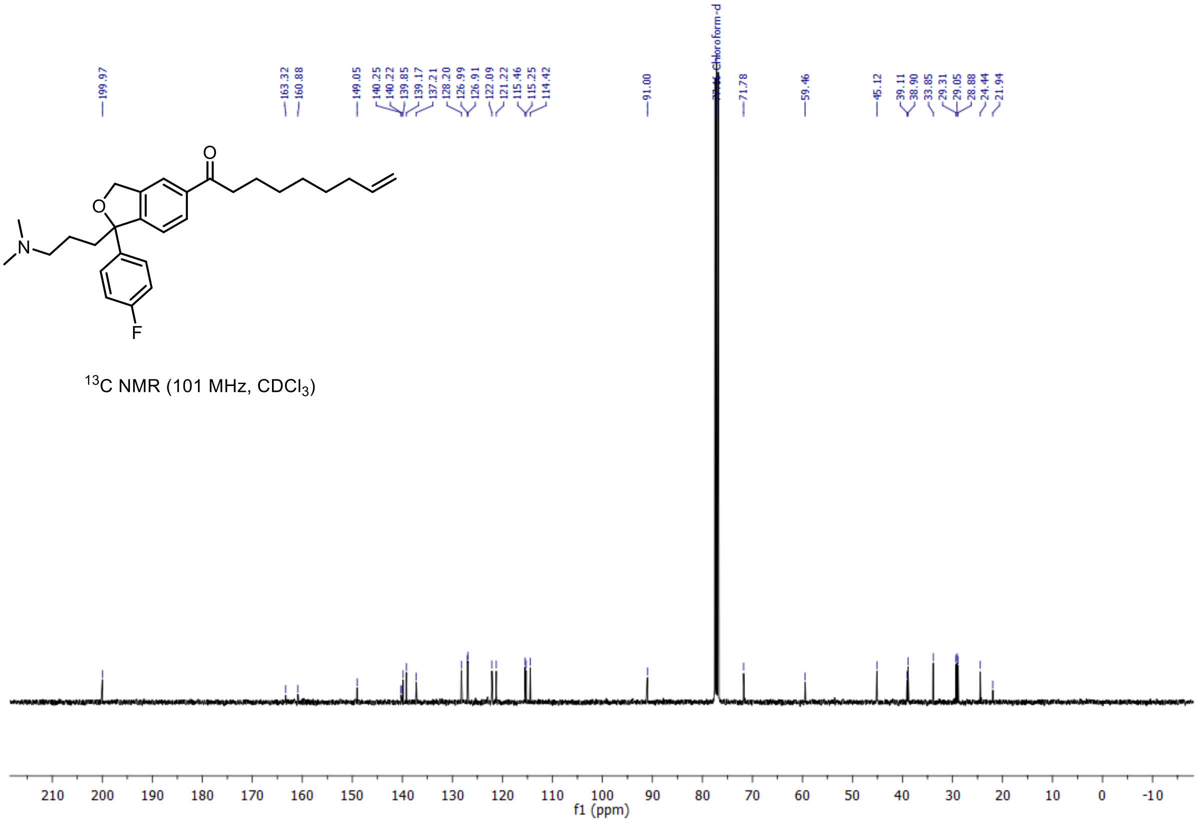

^{13}C NMR (101 MHz, $CDCl_3$)

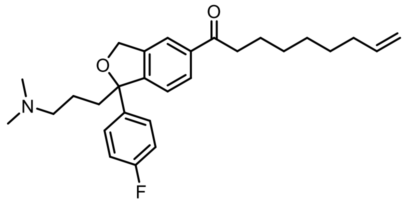

^{19}F NMR (565 MHz, $CDCl_3$)

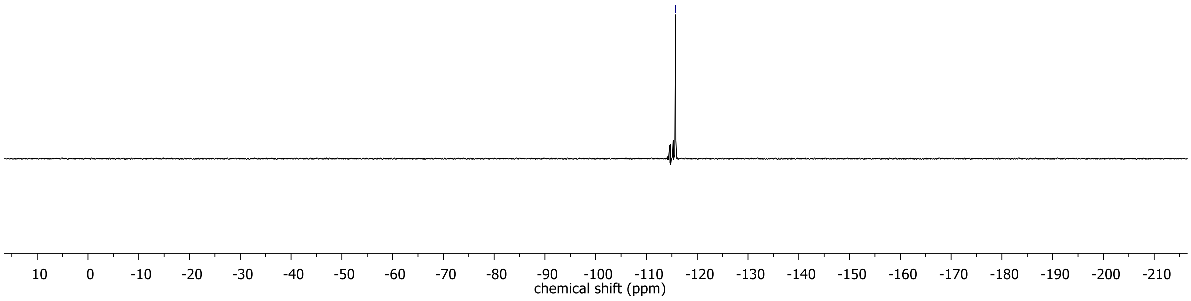

1-(Naphthalen-2-yl)hept-6-en-1-one

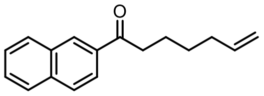

The title compound was prepared following general procedure C using *N*-methoxy-*N*-methyl-2-naphthamide (667 mg, 3.10 mmol, 1.00 equiv.) and a solution of 5-hexenylmagnesium bromide (1.50 equiv.). Purification by flash column chromatography (0 – 20% EtOAc in heptanes) afforded the title compound (471 mg, 1.98 mmol, 64%) as a colorless liquid.

All analytical data were in good agreement with those reported in literature.[13]

^{1}H NMR (400 MHz, $CDCl_3$) δ 8.47 (s, 1H), 8.04 (dd, *J* = 8.6, 1.7 Hz, 1H), 7.97 (d, *J* = 8.0 Hz, 1H), 7.93 – 7.83 (m, 2H), 7.65 – 7.51 (m, 2H), 5.92 – 5.71 (m, 1H), 5.12 – 4.86 (m, 2H), 3.11 (t, *J* = 7.4 Hz, 2H), 2.21 – 2.06 (m, 2H), 1.89 – 1.71 (m, , 2H), 1.60 – 1.48 (m, 2H).

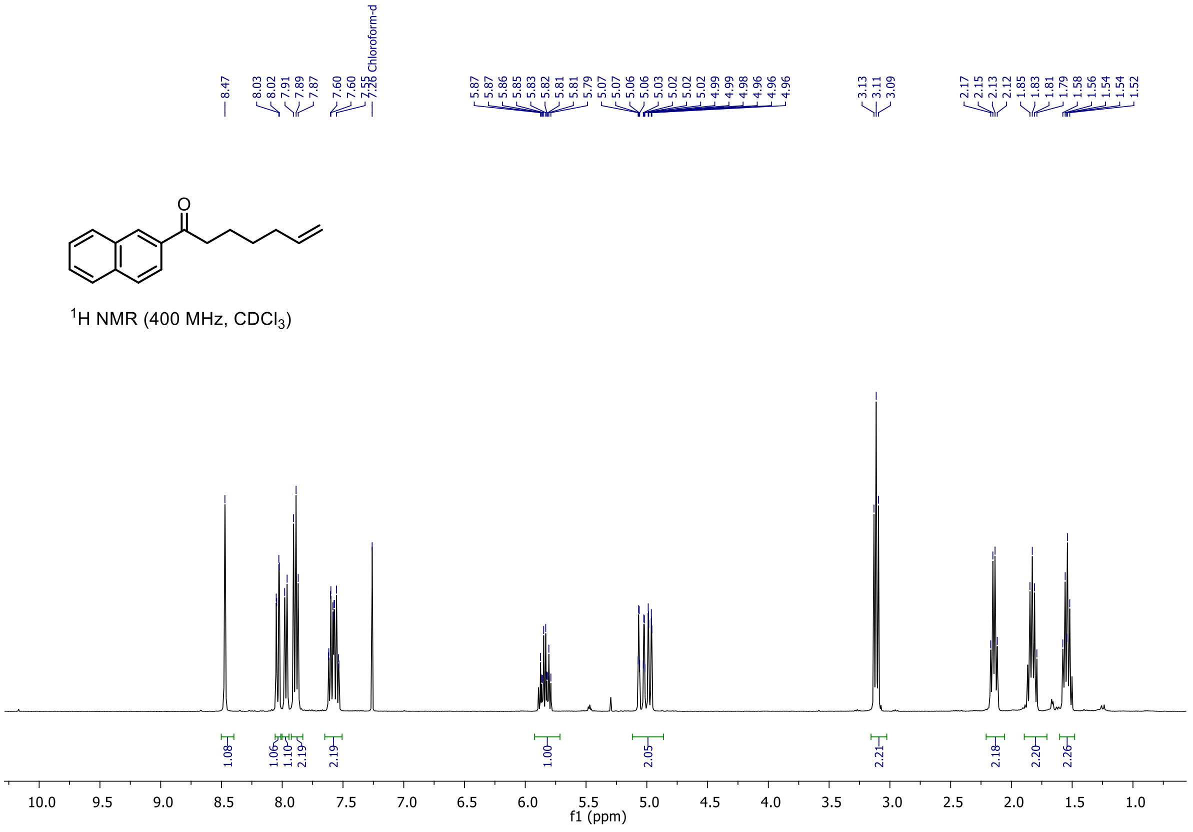

1-(4-Chlorophenyl)hept-6-en-1-one

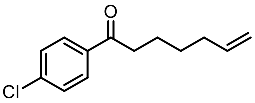

The title compound was prepared following general procedure C using 4-chloro-*N*-methoxy-*N*-methylbenzamide (1.60 g, 8.00 mmol, 1.00 equiv.) and a solution of 5-hexenylmagnesium bromide (1.50 equiv.). Purification by flash column chromatography (0 – 10% EtOAc in heptanes) afforded the title compound (891 mg, 4.00 mmol, 50%) as a colorless liquid.

^{1}H NMR (400 MHz, $CDCl_3$): δ 7.89 (d, *J* = 8.6 Hz, 2H), 7.42 (d, *J* = 8.7 Hz, 2H), 5.81 (ddt, *J* = 16.9, 10.2, 6.7 Hz, 1H), 5.11 – 4.89 (m, 2H), 3.00 – 2.82 (m, 2H), 2.15 – 2.00 (m, 2H), 1.82 – 1.68 (m, 2H), 1.52 – 1.41 (m, 2H).

All analytical data were in good agreement with those reported in literature.[13]

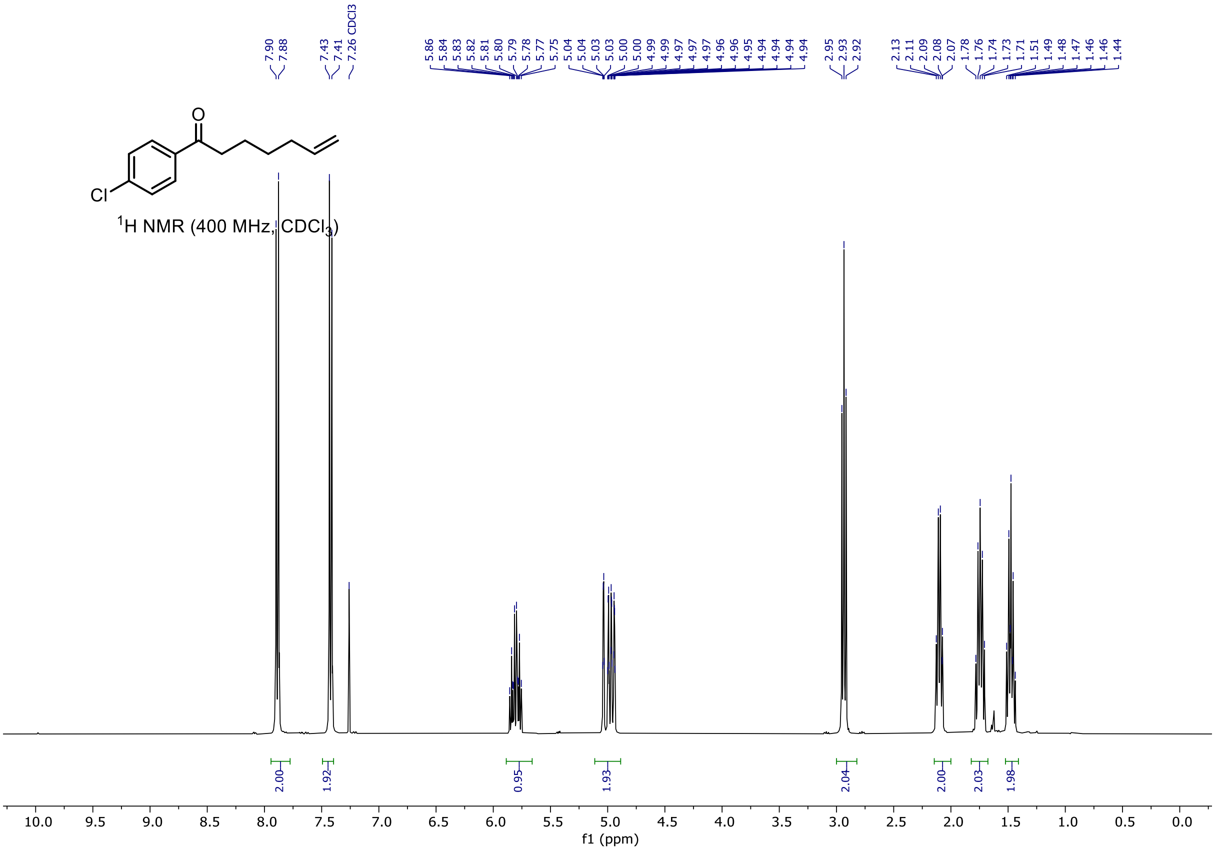

1-(2-Aminophenyl)non-8-en-1-one

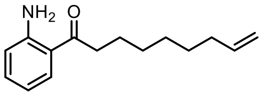

The title compound was prepared following general procedure E using 2-aminobenzonitrile (236 mg, 2.00 mmol, 1.00 equiv.) and a solution of 7-octenylmagnesium bromide (4.75 equiv.). Purification by flash column chromatography (0 – 20% EtOAc in heptanes) afforded the title compound (362 mg, 1.57 mmol, 78%) as yellow liquid.

^{1}H NMR (400 MHz, $CDCl_3$): δ 7.74 (dd, *J* = 8.3, 1.4 Hz, 1H), 7.30 – 7.20 (m, 1H), 6.73 – 6.58 (m, 2H), 6.26 (br s, 2H), 5.90 – 5.73 (m, 1H), 5.06 – 4.85 (m, 2H), 2.93 (t, *J* = 7.4 Hz, 2H), 2.13 – 1.96 (m, 2H), 1.81 – 1.63 (m, 2H), 1.48 –1.31 (m, 6H).

^{13}C NMR (101 MHz, $CDCl_3$): δ 203.3, 150.5, 139.2, 134.3, 131.4, 118.2, 117.5, 115.9, 114.4, 39.4, 33.9, 29.4, 29.1, 28.9, 25.1.

IR (neat) ν_{max}: 3473, 3343, 2926, 2853, 1613,1547, 1484, 1320, 1211, 1159, 908, 745, 656, 517.

HRMS (ESI$^+$): exact mass calculated for $[M+H]^+$ $(C_{15}H_{22}NO)^+$ requires *m/z* 232.1696, found *m/z* 232.1696.

1-(2-Aminophenyl)non-8-en-1-one

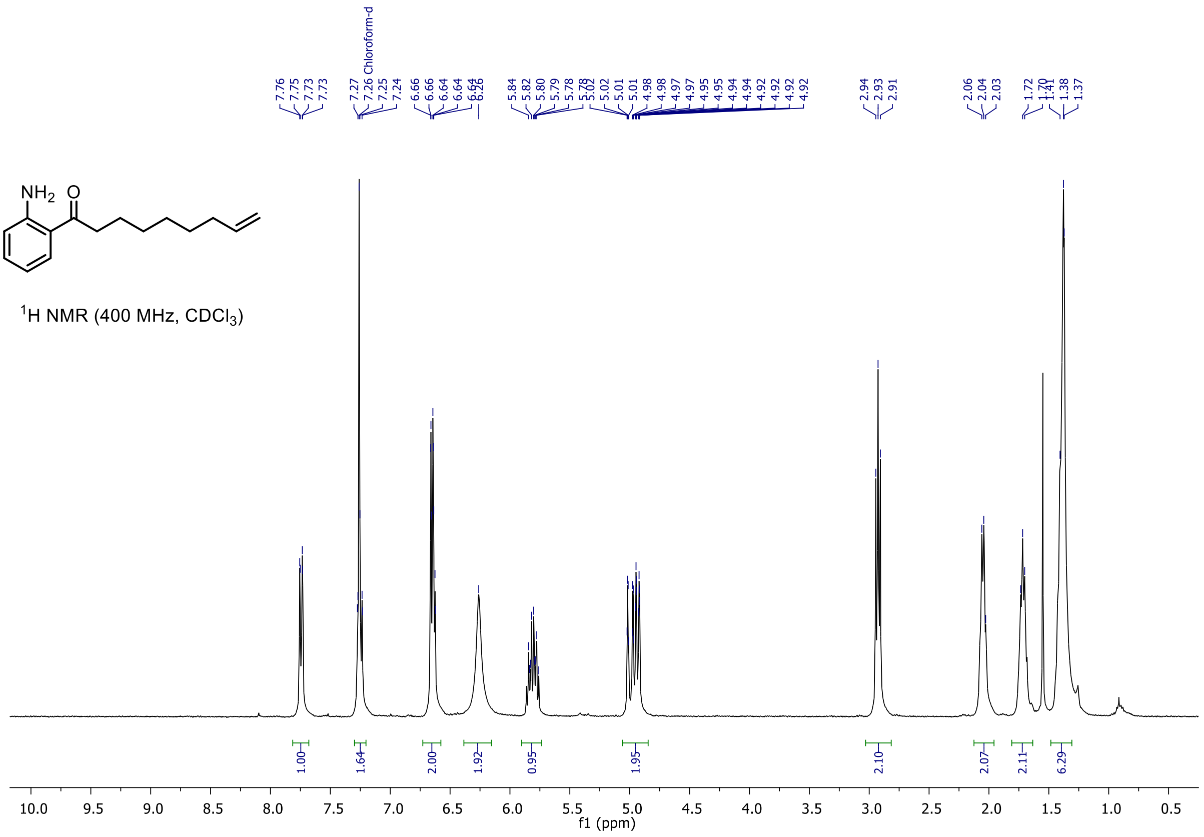

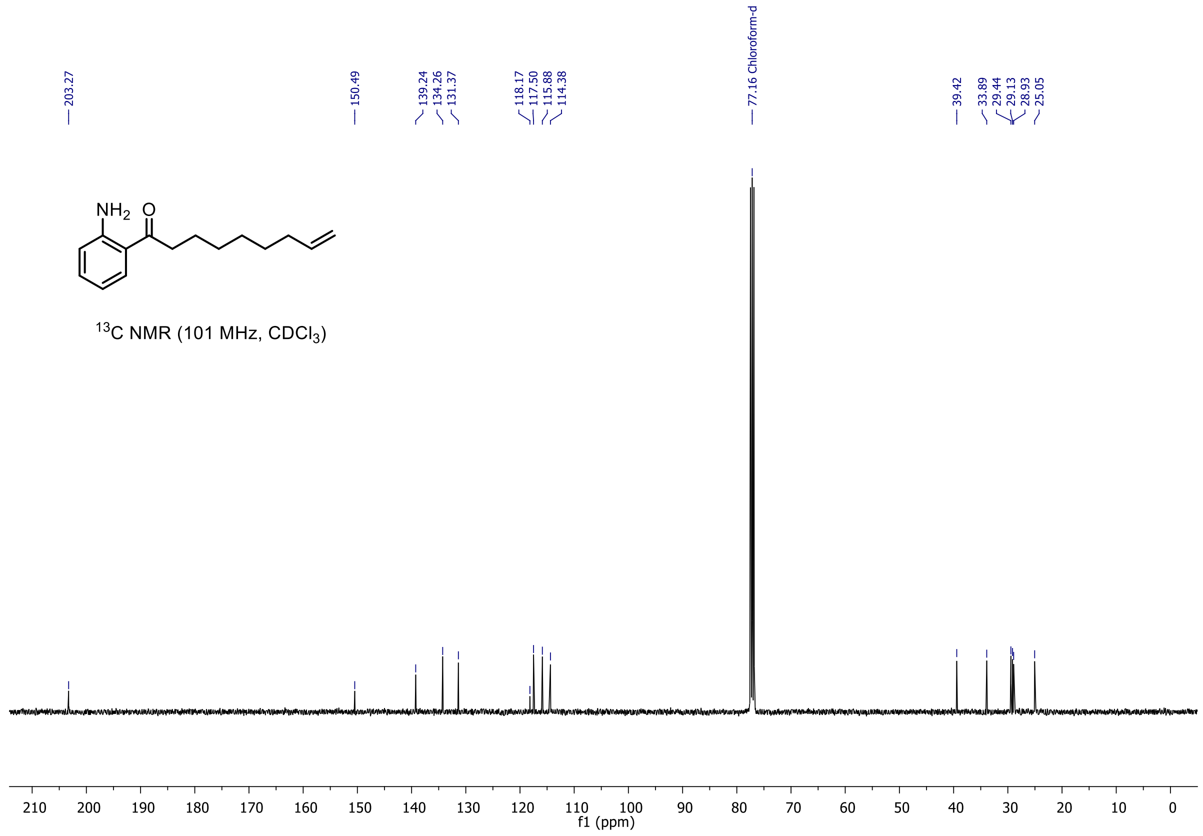

1-(4-methoxyphenyl)non-8-en-1-one

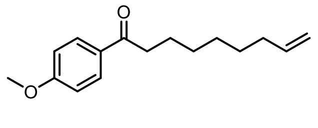

7-octenylmagnesium bromide solution was prepared following general procedure C using 8-bromo-1-octene (3.36 mL, 20.00 mmol, 1.00 equiv.) and magnesium (972 mg, 40.00 mmol, 2.00 equiv.).

The title compound was prepared following general procedure C using Weinreb amide *N*,4-dimethoxy-*N*-methylbenzamide (1.02 g, 5.00 mmol, 1.00 equiv.) and a solution of 7-octenylmagnesium bromide (1.50 equiv.). Purification by flash column chromatography (0 – 20% EtOAc in heptanes) afforded the title compound as a colorless solid (1.05 g, 4.26 mmol, 85%).

^{1}H NMR (400 MHz, $CDCl_3$) δ 7.99 – 7.90 (m, 2H), 6.96 – 6.90 (m, 2H), 5.80 (ddt, *J* = 16.9, 10.2, 6.7 Hz, 1H), 4.99 (dq, *J* = 17.1, 1.7 Hz, 1H), 4.93 (ddt, *J* = 10.1, 2.3, 1.2 Hz, 1H), 3.87 (s, 3H), 2.90 (t, *J* = 7.5 Hz, 2H), 2.04 (q, *J* = 6.8 Hz, 2H), 1.78 – 1.67 (m, 2H), 1.46 – 1.31 (m, 6H).

^{13}C NMR (101 MHz, $CDCl_3$) δ 199.3 (C), 163.4 (C), 139.2 (CH), 130.4 (2CH), 130.3 (C), 114.4 (CH_2), 113.8 (2CH), 55.6 (CH_3), 38.4 (CH_2), 33.9 (CH_2), 29.4 (CH_2), 29.1 (CH_2), 28.9 (CH_2), 24.7 (CH_2).

IR (neat) ν_{max}: 2927, 2853, 1675, 1599, 1576, 1509, 1461, 1417, 1308, 1255, 1169, 1031, 995, 910, 834, 807.

HRMS (ESI$^+$): exact mass calculated for $[M+Na]^+$ $(C_{16}H_{22}O_2Na)^+$ requires *m*/*z* 269.1512, found *m*/*z* 269.1510.

1-(4-methoxyphenyl)non-8-en-1-one

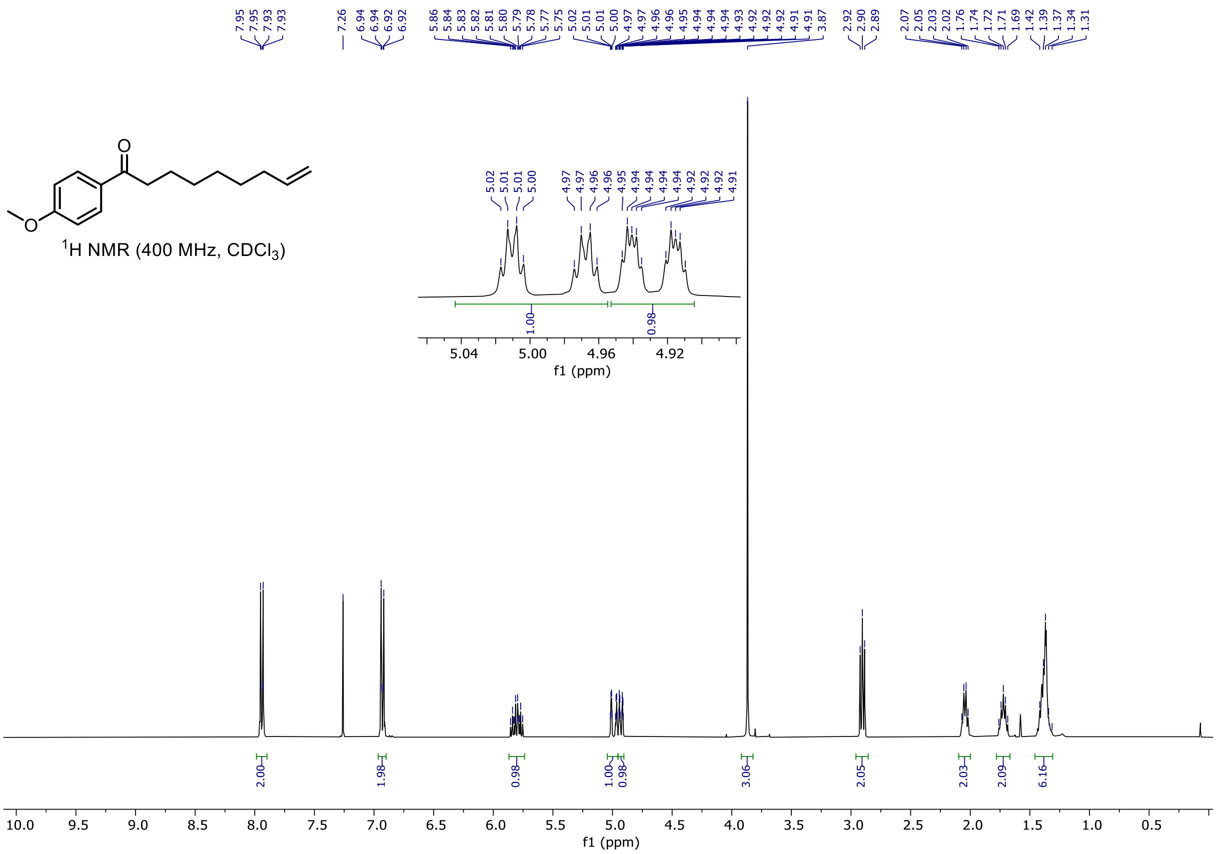

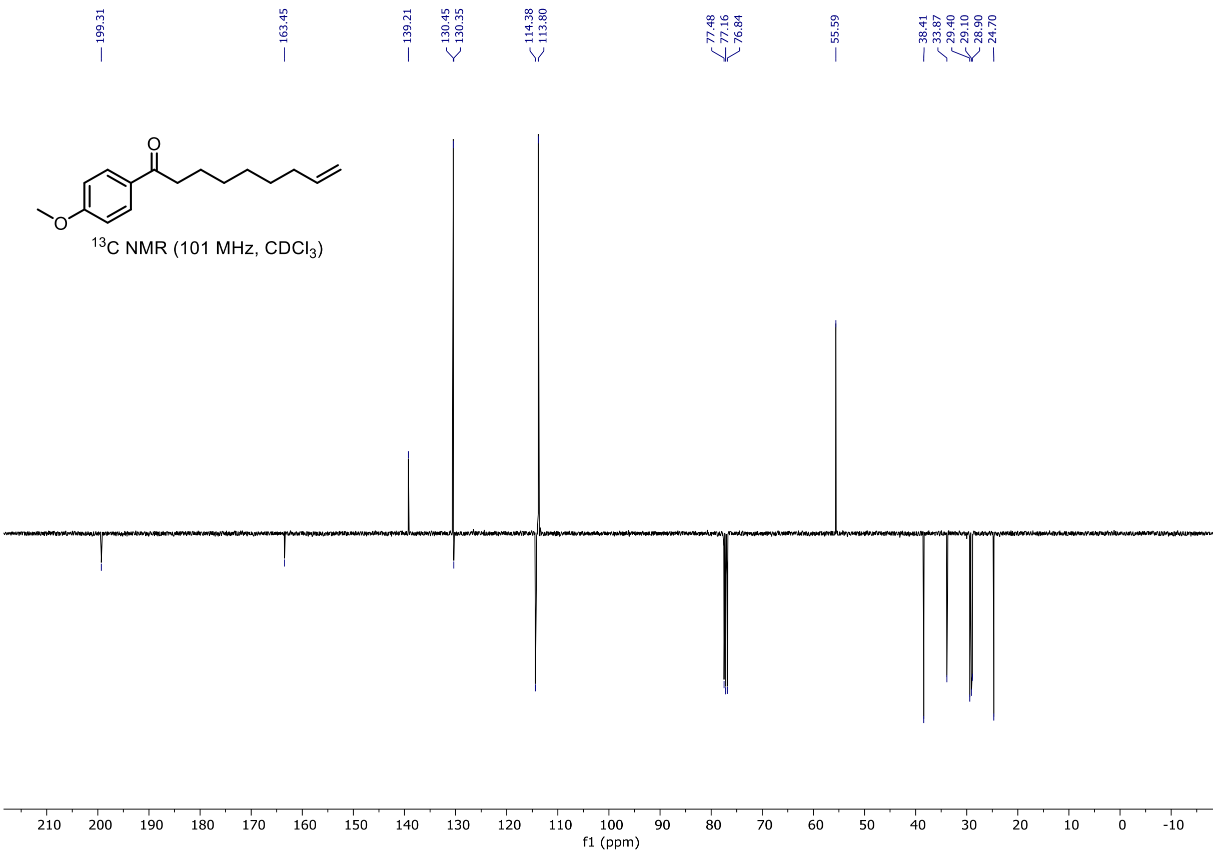

2.4 Characterization of δ-alcohols

5-Hydroxy-1-phenylheptan-1-one (1a)

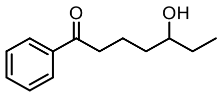

The title compound was prepared following general procedure F using 1-phenylhept-6-en-1-one (18.8 mg, 0.10 mmol, 1.00 equiv.) and TfOH (27 µL, 0.30 mmol, 3.00 equiv.). Purification by flash column chromatography (0 – 70% EtOAc in heptanes) afforded the title compound (14.4 mg, 70 µmol, 70%) as a yellow oil and a single regioisomer.

Crude product: 96% NMR yield, r.r. (δ_{OH}: γ_{OH}) = 92:8.

^{1}H NMR (400 MHz, CD_2Cl_2): δ 7.98 – 7.94 (m, 2H), 7.57 (ddd, *J* = 6.7, 3.9, 1.3 Hz, 1H), 7.50 – 7.44 (m, 2H), 3.52 (ddd, *J* = 12.2, 8.0, 4.5 Hz, 1H), 3.01 (t, *J* = 7.2 Hz, 2H), 1.93 – 1.75 (m, 2H), 1.70 (s, 1H), 1.61 – 1.38 (m, 4H), 0.93 (t, *J* = 7.5 Hz, 3H).

^{13}C NMR (101 MHz, CD_2Cl_2): δ 200.8, 137.7, 133.4, 129.1 (2C), 128.5 (2C), 73.3, 38.9, 37.0. 30.8, 20.7, 10.3.

IR (neat) v_{max}: 3413, 2959, 2931, 2875, 1678, 1448, 1228, 1201, 753, 689.

HRMS (ESI$^+$): exact mass calculated for $[M+Na]^+$ $(C_{13}H_{18}O_2Na)^+$ requires *m/z* 229.1190, found *m/z* 229.1197.

5-Hydroxy-1-phenylheptan-1-one (1a)

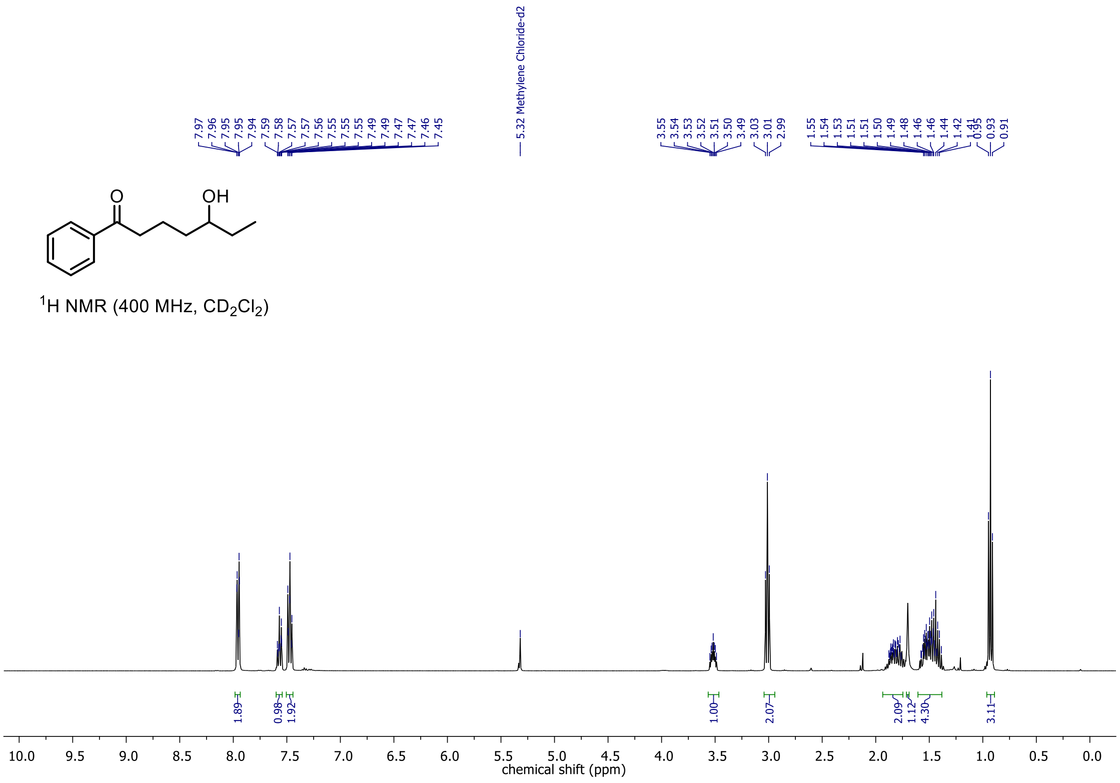

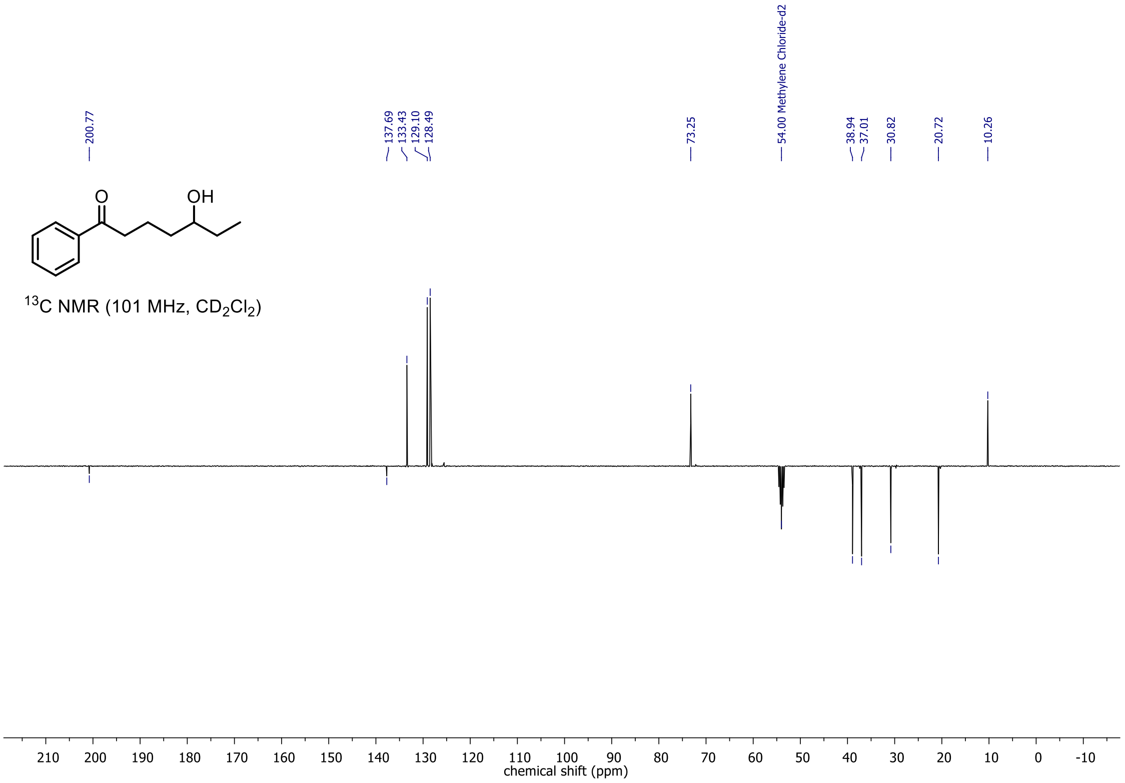

5-Hydroxy-1-phenylnonan-1-one (1b)

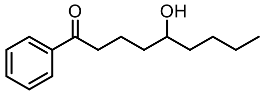

The title compound was prepared following general procedure E using 1-phenylnon-8-en-1-one (21.6 mg, 0.10 mmol, 1.00 equiv.) and TfOH (27 µL, 0.30 mmol, 3.00 equiv.). Purification by flash column chromatography (8 – 40% EtOAc in heptanes with 1% triethylamine additive) afforded title compound (19.0 mg, 81 µmol, 81%) as a colorless solid and a single regioisomer.

Crude product: 85% NMR yield (δ_{OH}), r.r. (δ_{OH}:γ_{OH}) = 91:9

^{1}H NMR (400 MHz, CD_2Cl_2) δ 7.99 – 7.93 (m, 2H), 7.60 – 7.54 (m, 1H), 7.50 – 7.45 (m, 2H), 3.63 – 3.54 (m, 1H), 3.01 (t, *J* = 7.2 Hz, 2H), 1.92 – 1.72 (m, 2H), 1.69 (br s, 1H), 1.60 – 1.26 (m, 8H), 0.91 (t, *J* = 7.0 Hz, 3H).

^{13}C NMR (101 MHz, CD_2Cl_2) δ 200.6 (C), 137.5 (C), 133.2 (CH), 128.9 (2CH), 128.3 (2CH), 71.7 (CH), 38.8 (CH_2), 37.7 (CH_2), 37.4 (CH_2), 28.3 (CH_2), 23.2 (CH_2), 20.6 (CH_2), 14.3 (CH_3).

IR (neat) ν_{max}: 3439, 2954, 2928, 2858, 1683, 1448, 1273, 1070, 742, 713, 690.

HRMS (ESI$^+$): exact mass calculated for $[M+Na]^+$ $(C_{15}H_{22}O_2Na)^+$ requires *m/z* 257.1512, found *m/z* 257.1510.

5-Hydroxy-1-phenylnonan-1-one (1b)

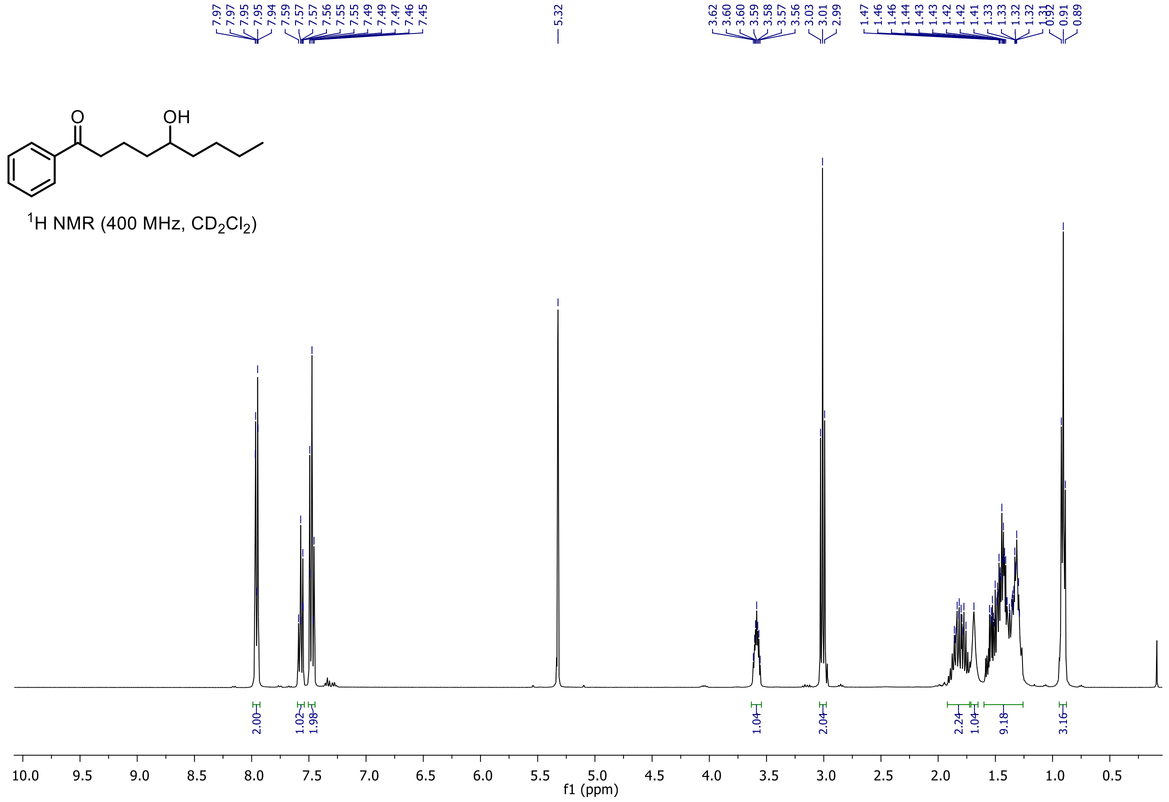

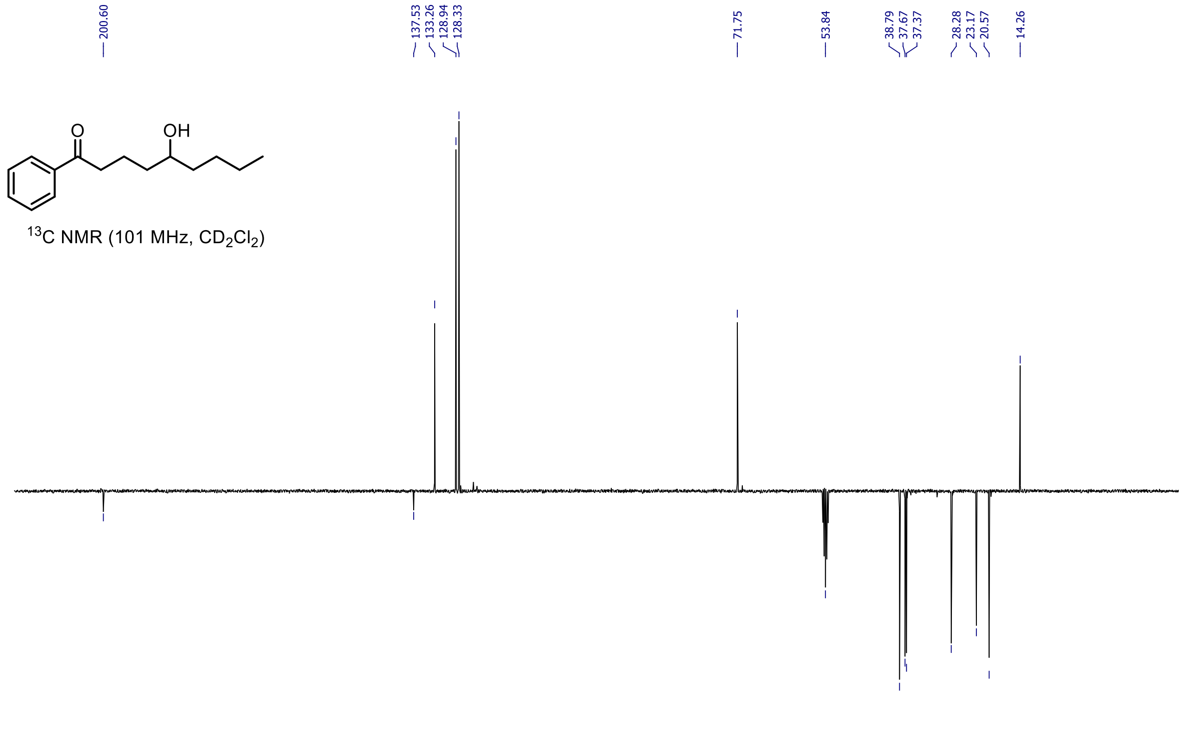

5-Hydroxy-1-phenyldecan-1-one (1c)

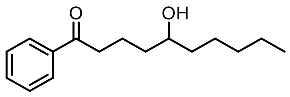

The title compound was prepared following general procedure F using 1-phenyldec-9-en-1-one (24.0 mg, 0.10 mmol, 1.00 equiv.) and TfOH (27 µL, 0.30 mmol, 3.00 equiv.). Purification by flash column chromatography (10 – 40% EtOAc in heptanes) afforded the title compound (17.4 mg, 70 µmol, 70%) as a colorless oil and single regioisomer.

Crude product: 84% NMR yield, r.r. (δ_{OH}:γ_{OH}) = 92 : 8

^{1}H NMR (400 MHz, CD_2Cl_2) δ 7.99 – 7.92 (m, 2H), 7.61 – 7.53 (m, 1H), 7.51 – 7.43 (m, 2H), 3.64 – 3.53 (m, 1H), 3.01 (t, *J* = 7.2 Hz, 2H), 1.91 – 1.73 (m, 2H), 1.65 (br s, 1H), 1.59 – 1.25 (m, 10H), 0.89 (t, *J* = 6.9 Hz, 3H).

^{13}C NMR (101 MHz, CD_2Cl_2) δ 200.6 (C), 137.5 (C), 133.3 (CH), 128.9 (2CH), 128.3 (2CH), 71.8 (CH), 38.8 (CH_2), 38.0 (CH_2), 37.4 (CH_2), 32.3 (CH_2), 25.8 (CH_2), 23.1 (CH_2), 20.6 (CH_2), 14.2 (CH_3).

IR (neat) ν_{max}: 3412, 2951, 2926, 2855, 1681, 1447, 1199, 753, 733, 688.

HRMS (ESI$^+$): exact mass calculated for $[M+Na]^+$ $(C_{16}H_{24}O_2Na)^+$ requires *m*/*z* 271.1669, found *m*/*z* 271.1666.

5-Hydroxy-1-phenyldecan-1-one (1c)

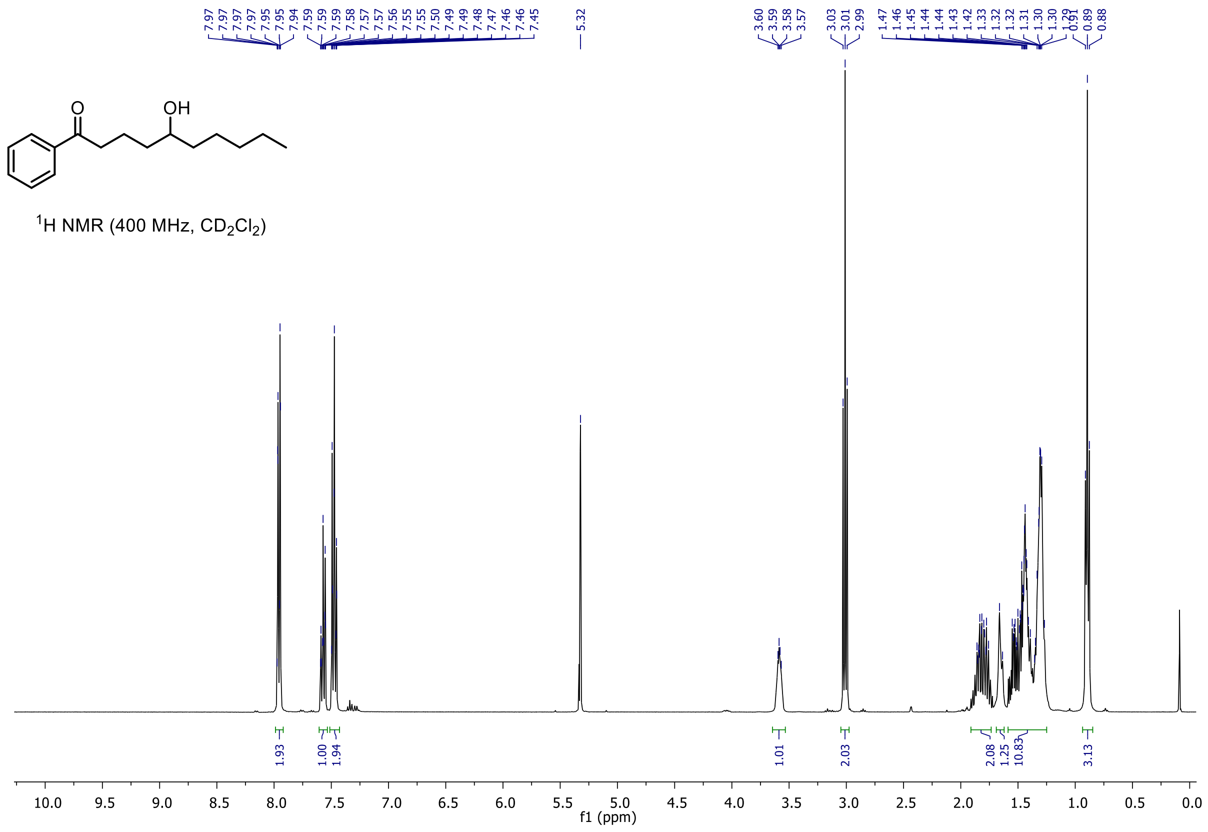

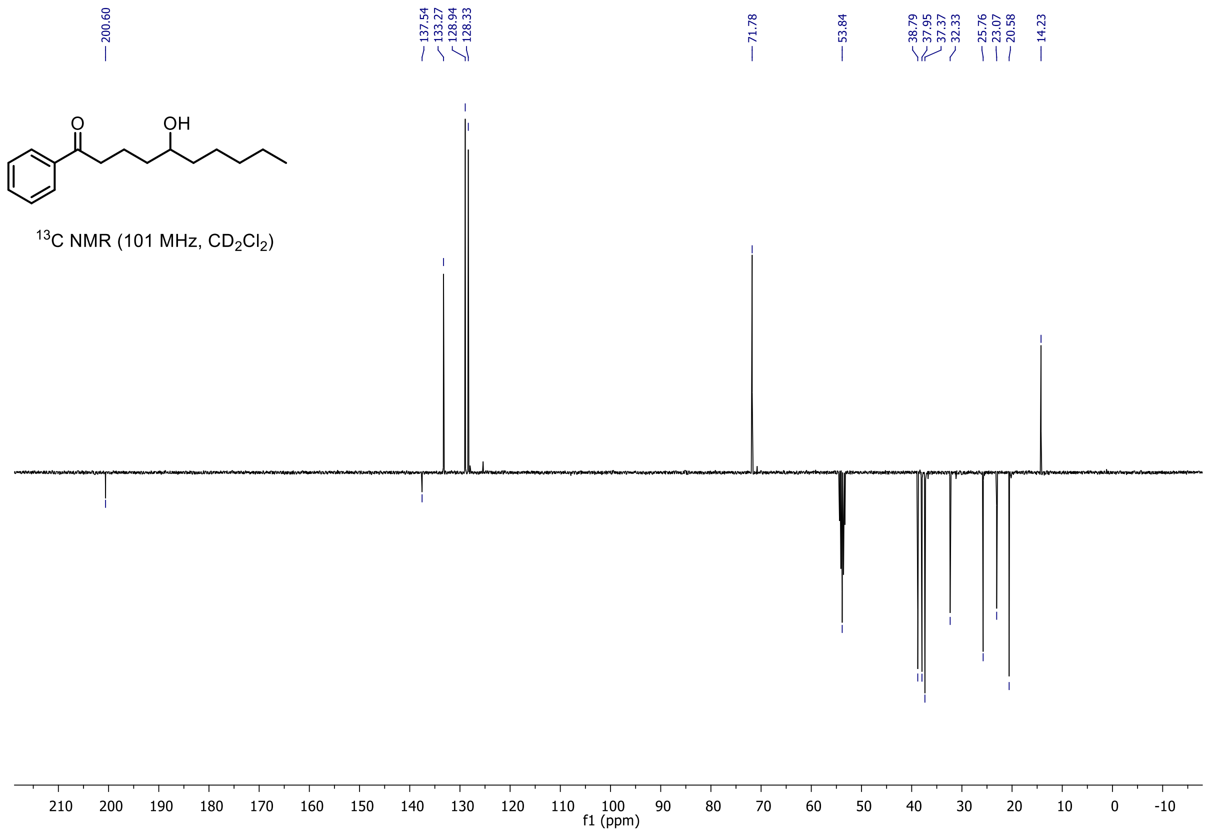

5-Hydroxy-1-phenyldodecan-1-one (1d)

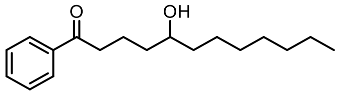

The title compound was prepared following general procedure F using 1-phenyldodec-11-en-1-one (25.8 mg, 0.10 mmol, 1.00 equiv.) and TfOH (27 µL, 0.30 mmol, 3.00 equiv.). Purification by flash column chromatography (8 – 30% EtOAc in heptanes) afforded the title compound (15.4 mg, 56 µmol, 56%) as a colorless solid and a single regioisomer.

Crude product: 70% NMR yield, r.r. (δ_{OH}:γ_{OH}) = 91 : 9

^{1}H NMR (400 MHz, CD_2Cl_2) δ 7.99 – 7.92 (m, 2H), 7.60 – 7.54 (m, 1H), 7.51 – 7.44 (m, 2H), 3.58 (tt, *J* = 8.5, 4.3 Hz, 1H), 3.01 (t, *J* = 7.2 Hz, 2H), 1.90 – 1.73 (m, 2H), 1.63 (br s, 1H), 1.58 – 1.23 (m, 14H), 0.88 (t, *J* = 6.9 Hz, 3H).

^{13}C NMR (101 MHz, CD_2Cl_2) δ 200.6 (C), 137.5 (C), 133.3 (CH), 128.9 (2CH), 128.3 (2CH), 71.8 (CH), 38.8 (CH_2), 38.0 (CH_2), 37.4 (CH_2), 32.3 (CH_2), 30.1 (CH_2), 29.7 (CH_2), 26.1 (CH_2), 23.1 (CH_2), 20.6 (CH_2), 14.3 (CH_3).

IR (neat) ν_{max}: 3452, 2924, 2854, 1716, 1688, 1449, 1271, 1109, 1069, 1026, 750, 711, 690.

HRMS (ESI$^+$): exact mass calculated for $[M+Na]^+$ $(C_{18}H_{28}O_2Na)^+$ requires *m*/*z* 299.1982, found *m*/*z* 299.1975.

5-Hydroxy-1-phenyldodecan-1-one (1d)

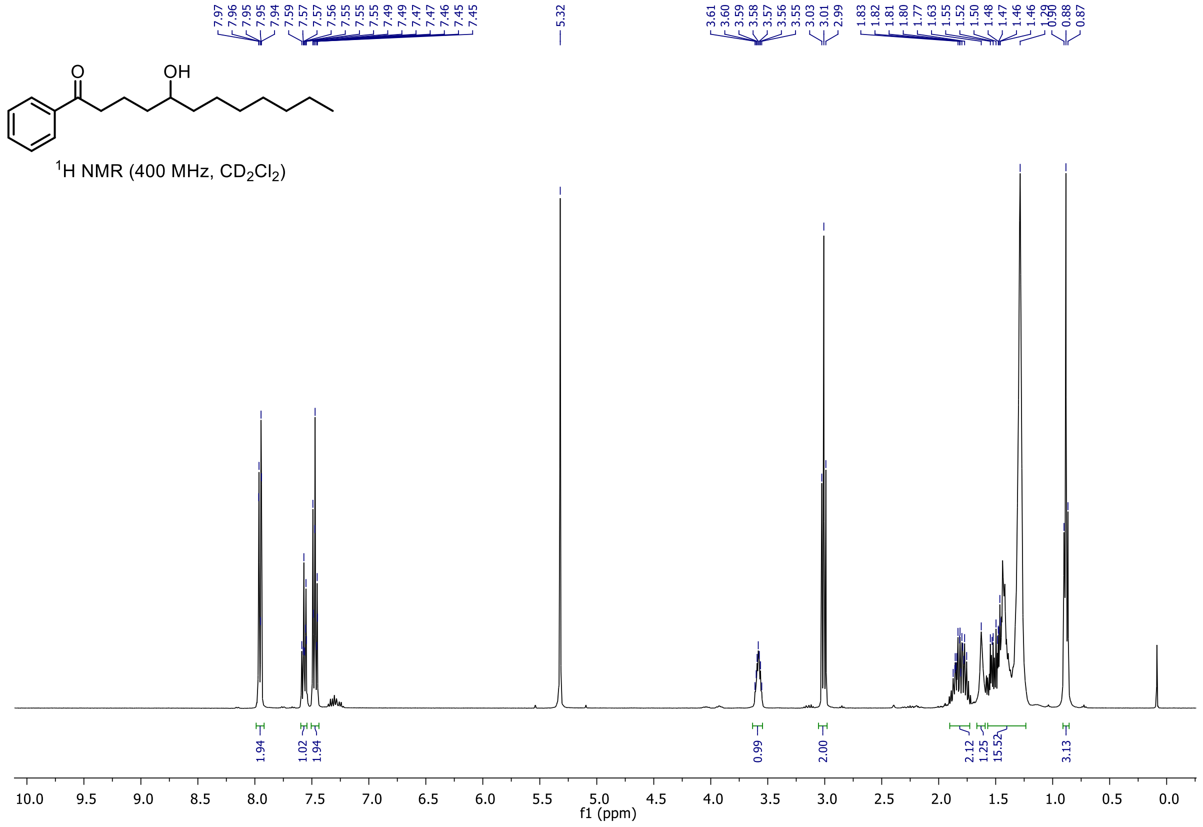

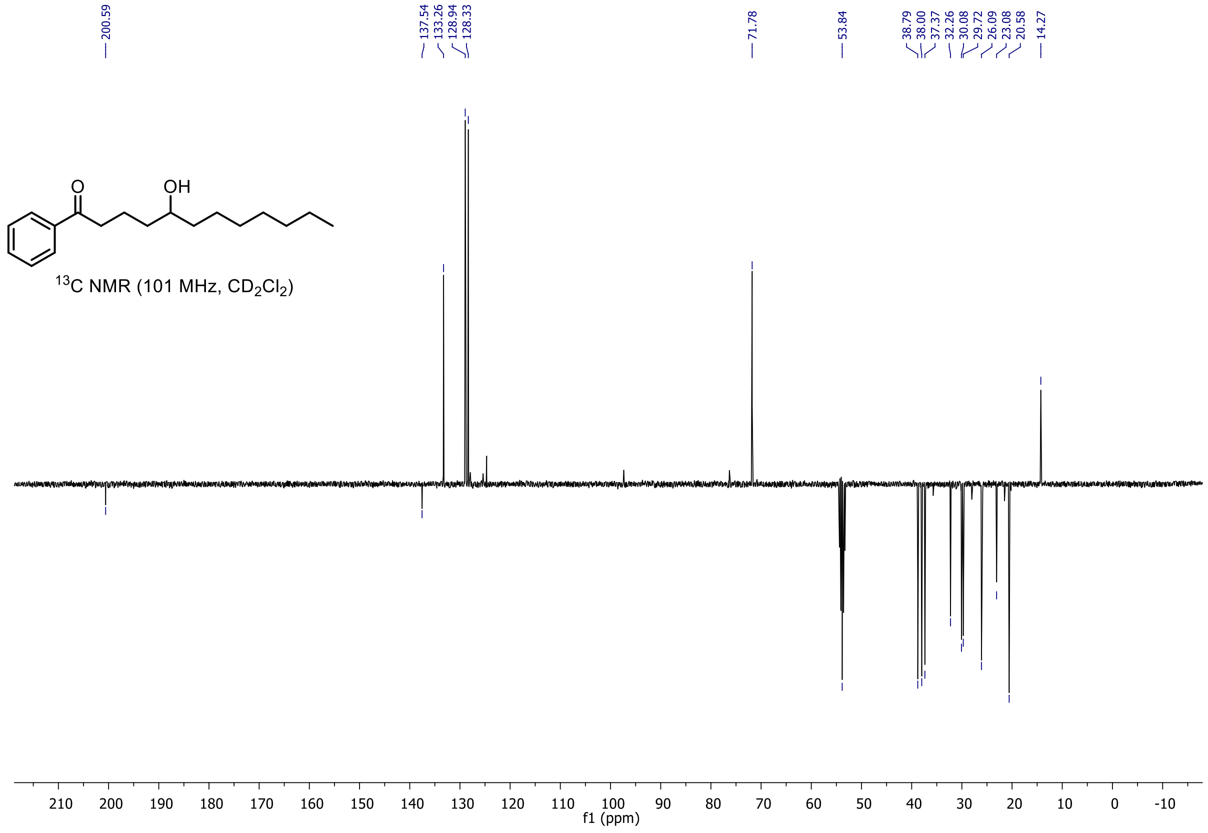

5-Hydroxy-1-phenylnonan-1-one (1b)

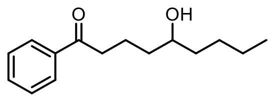

The title compound was prepared following general procedure F using 1-phenylnon-6-en-1-one (21.6 mg, 0.10 mmol, 1.00 equiv.) and TfOH (27 µL, 0.30 mmol, 3.00 equiv.). Purification by flash column chromatography (0 – 100% EtOAc in heptanes) afforded the title compound (16.8 mg, 72 µmol, 72%) as a colorless solid and a single regiosiomer.

Crude product: 85% NMR yield, r.r. (δ_{OH}:γ_{OH}) = 94:6.

*Analytical data was identical to **1b** synthesized from 1-phenylnon-8-en-1-one (see above). An additional NMR spectrum in acetone-d_6 is provided here:*

^{1}H NMR (400 MHz, $(CD_3)_2CO$): δ 8.04 – 7.97 (m, 2H), 7.64 – 7.57 (m, 1H), 7.55 – 7.47 (m, 2H), 3.61 – 3.53 (m, 1H), 3.39 (d, *J* = 5.4 Hz, 1H), 3.11 – 3.00 (m, 2H), 1.94 – 1.82 (m, 1H), 1.82 – 1.69 (m, 1H), 1.56 – 1.39 (m, 5H), 1.35 – 1.26 (m, 3H), 0.89 (t, *J* = 7.2 Hz, 3H).

^{13}C NMR (101 MHz, $(CD_3)_2CO$): δ 200.5, 138.2, 133.6, 129.4 (2C), 128.8 (2C), 71.2, 39.2, 38.3, 37.9, 28.8, 23.1, 21.4, 14.4.

5-Hydroxy-1-phenylnonan-1-one (1b)

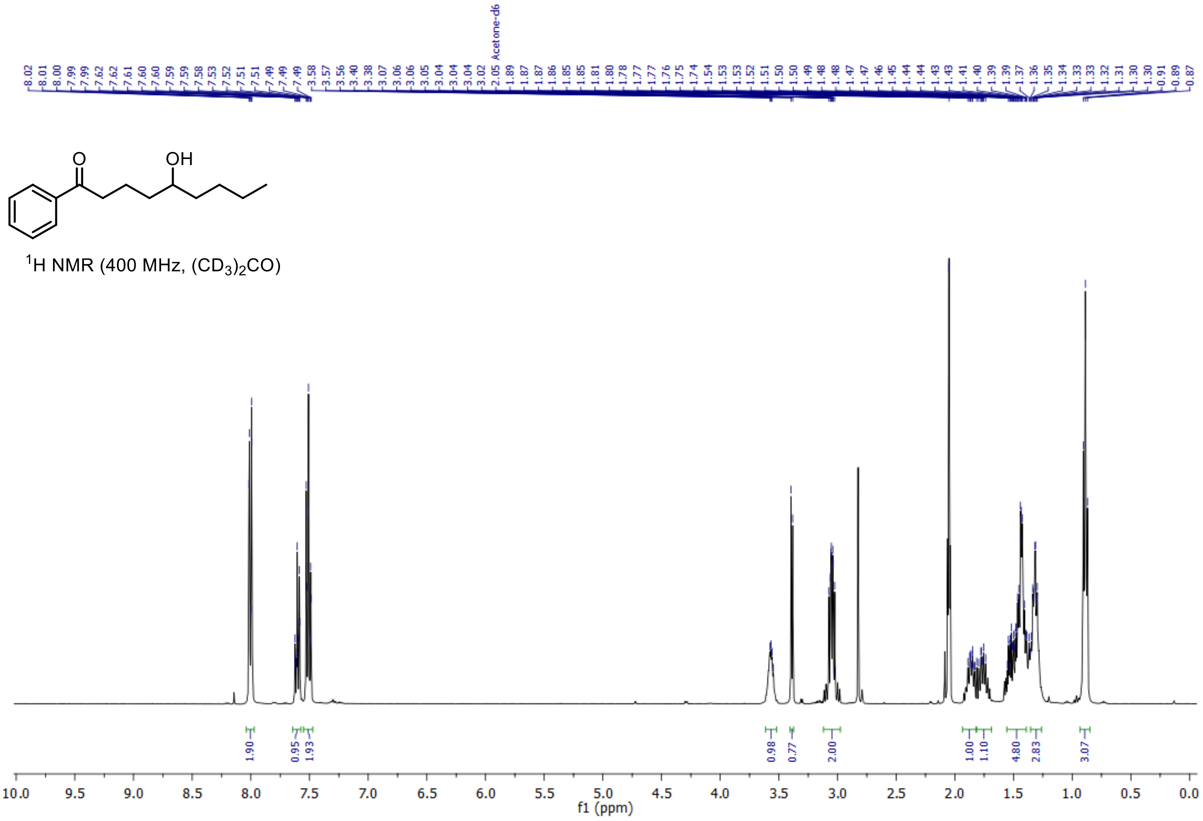

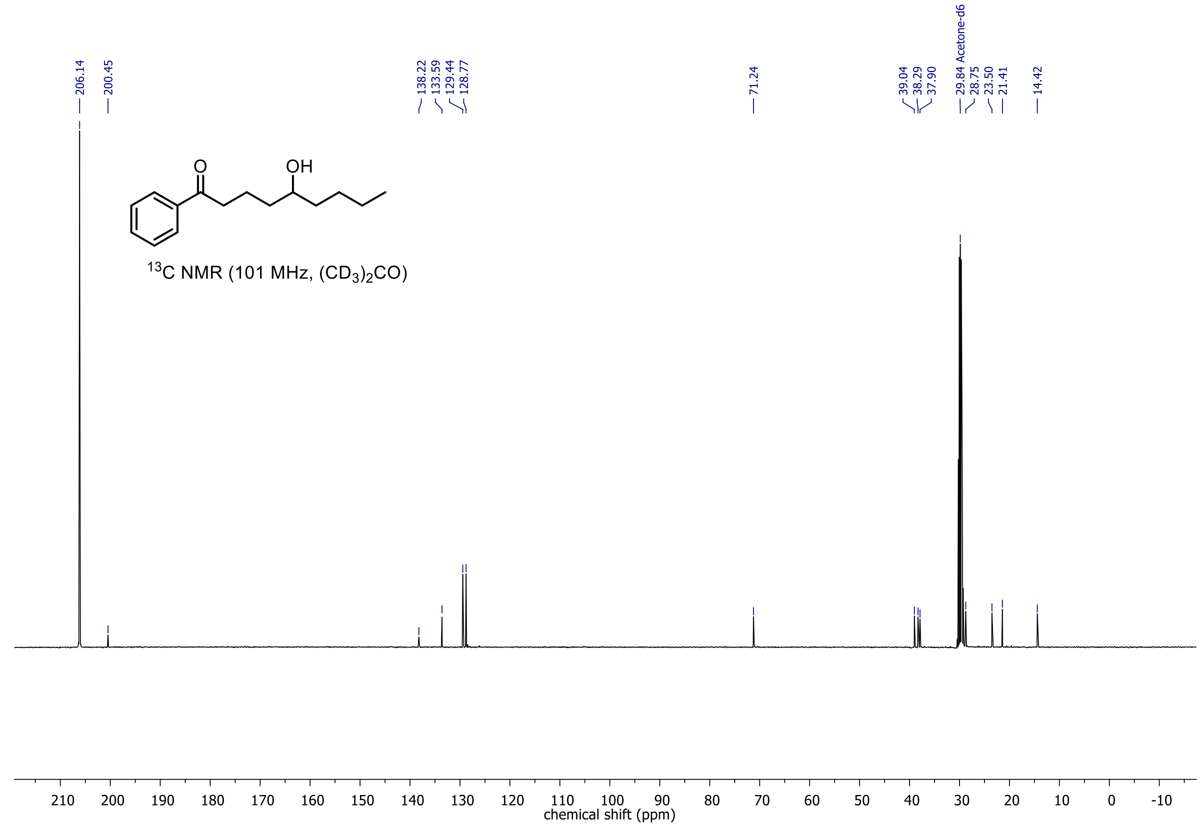

5-Hydroxy-1-phenylundecan-1-one (1e)

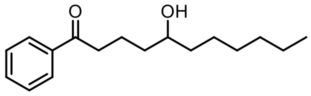

The title compound was prepared following general procedure F using 1-phenylundec-8-en-1-one (21.6 mg, 89 µmol, 1.00 equiv.) and TfOH (24 µL, 0.27 mmol, 3.00 equiv.). Purification by flash column chromatography (0 – 100% EtOAc in heptanes) afforded the title compound (12 mg, 46 µmol, 52%) as a colorless oil and a single regioisomer.

Crude product: 60% NMR yield, r.r. (δ_{OH}:γ_{OH}) = 90:10.

^{1}H NMR (400 MHz, CD_2Cl_2): δ 8.00 – 7.92 (m, 2H), 7.61 – 7.53 (m, 1H), 7.53 – 7.43 (m, 2H), 3.62 – 3.54 (m, 1H), 3.01 (t, *J* = 7.2 Hz, 2H), 1.87 – 1.73 (m, 3H),1.61 (s, 1H) 1.47 – 1.40 (m, 3H), 1.35 – 1.26 (m, 8H), 0.90 – 0.85 (m, 3H).

^{13}C NMR (101 MHz, CD_2Cl_2): δ 200.8, 137.7, 133.4, 129.1 (2C), 128.5 (2C), 71.9, 39.0, 38.2, 37.5, 32.4, 29.9, 26.2, 23.2, 20.7, 14.4.

IR (neat) ν_{max}: 3437, 2927, 2854, 1684, 1449, 1266, 974, 741, 690.

HRMS (ESI$^+$): exact mass calculated for $[M+Na]^+$ $(C_{17}H_{26}O_2Na)^+$ requires *m/z* 285.1825, found *m/z* 285.1818.

5-Hydroxy-1-phenylundecan-1-one (1e)

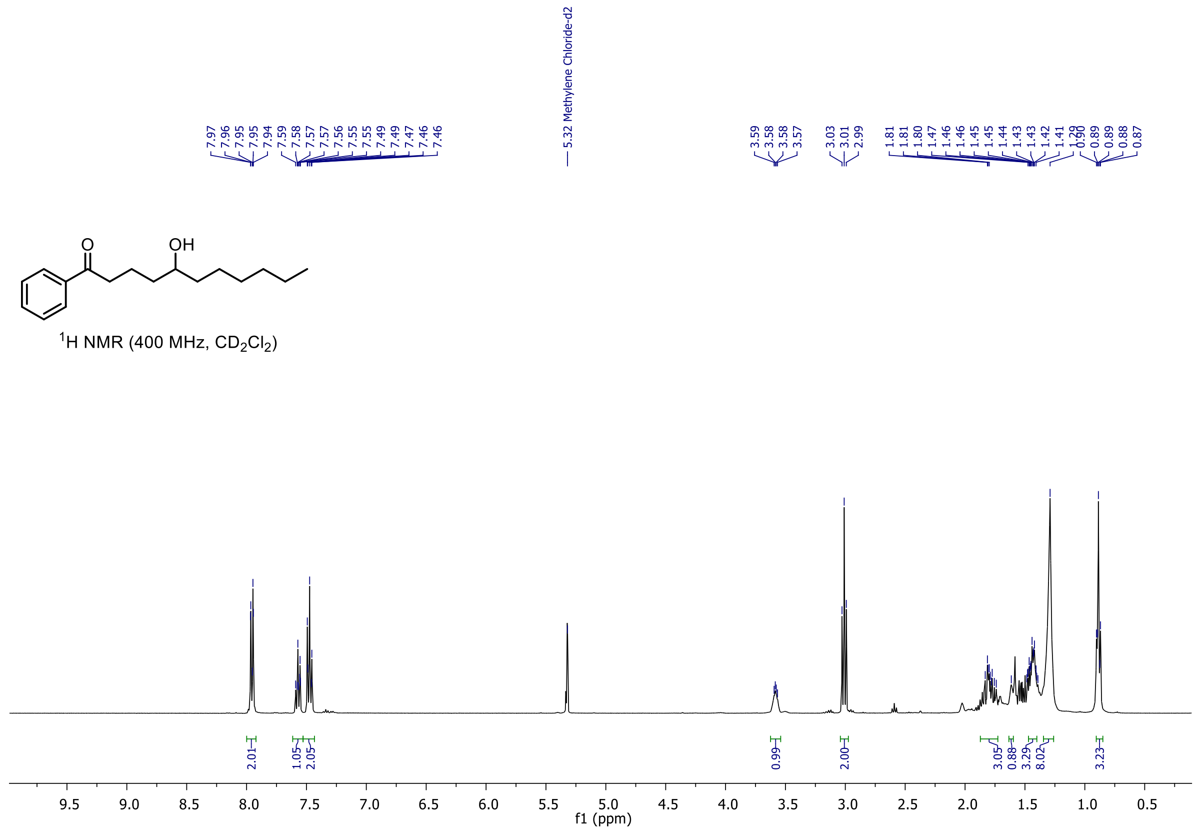

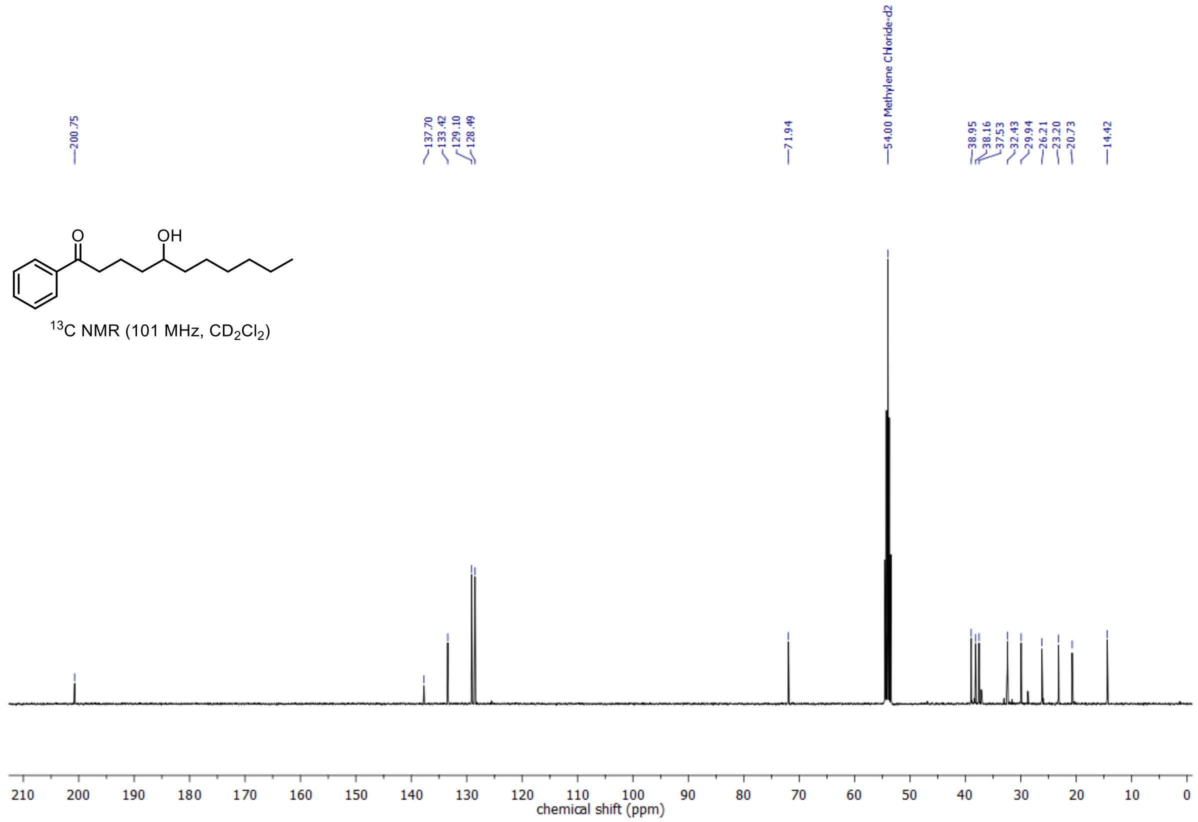

10,10,10-Trifluoro-5-hydroxy-1-phenyldecan-1-one (1f)

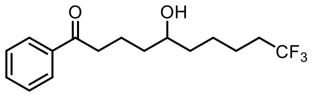

The title compound was prepared following general procedure F using (*E*)-10,10,10-trifluoro-1-phenyldec-7-en-1-one (28.4 mg, 0.10 mmol, 1.00 equiv.) and TfOH (27 µL, 0.30 mmol, 3.00 equiv.). Purification by flash column chromatography (0 – 50% EtOAc in heptanes) afforded the title compound (25.8 mg, 85 µmol, 85%) as a colorless solid and a mixture of regioisomers (δ:γ = 93:7).

Crude product: 86% NMR yield, r.r. (δ_{OH}: γ_{OH}) = 92:8.

A full list of signals arising from the minor γ-regioisomer can be found in Section 2.5.

^{1}H NMR (400 MHz, CD_2Cl_2): δ 8.03 – 7.81 (m, 2H), 7.61 – 7.54 (m, 1H), 7.53 – 7.39 (m, 2H), 3.59 (s, 1H), 3.02 (t, *J* = 7.1 Hz, 2H), 2.21 – 2.02 (m, 2H), 1.93 – 1.69 (m, 3H), 1.62 – 1.41 (m, 8H).

^{13}C NMR (101 MHz, CD_2Cl_2): δ 200.7, 137.7, 133.5, 129.1 (2C), 128.5 (2C), 128.0 (q, *J* = 276.3 Hz), 71.6, 38.8, 37.6, 37.6, 34.1 (q, *J* = 28.2 Hz), 25.4, 22.5 (q, *J* = 2.9 Hz), 20.6.

^{19}F NMR (377 MHz, CD_2Cl_2): δ -66.75 (t, *J* = 11.1 Hz).

IR (neat) ν_{max}: 3423, 2929, 1711, 1679, 1362, 1253, 1223, 1137, 1098, 1044, 688, 654.

HRMS (ESI^{+}): exact mass calculated for $[M+Na]^+$ $(C_{16}H_{21}F_3O_2Na)^+$ requires *m/z* 325.1386, found *m/z* 325.1396.

10,10,10-Trifluoro-5-hydroxy-1-phenyldecan-1-one (1f)

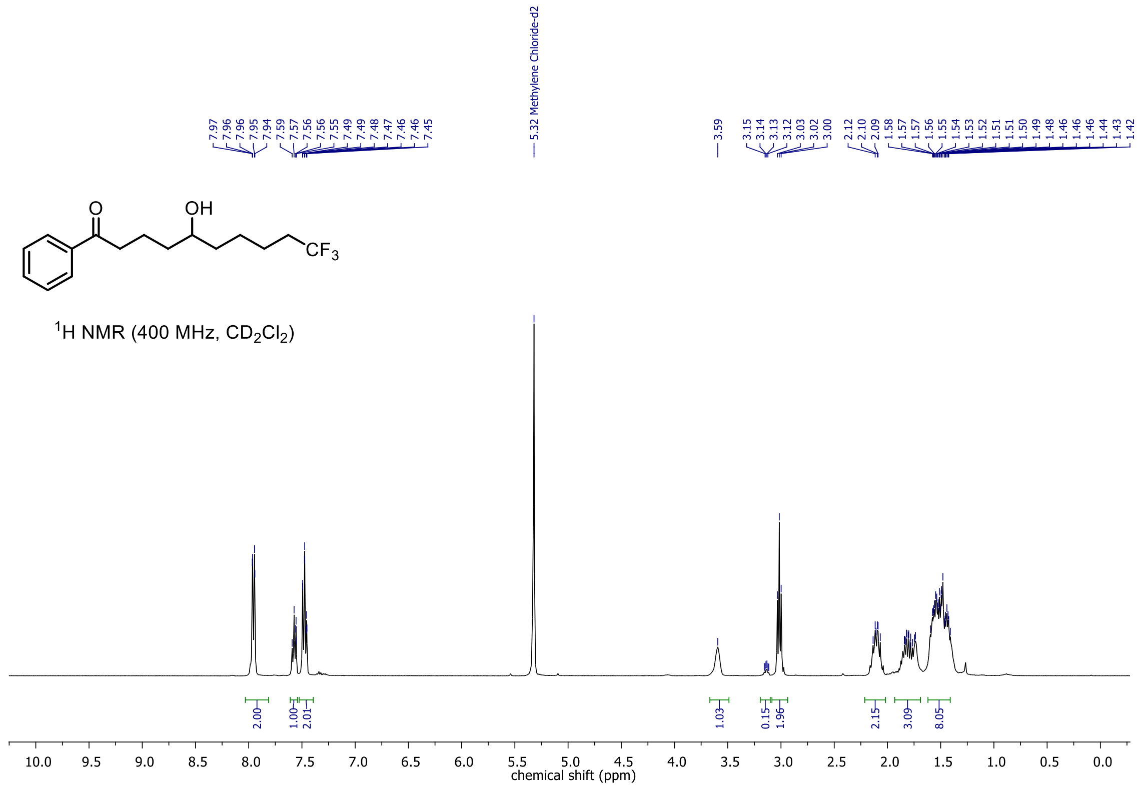

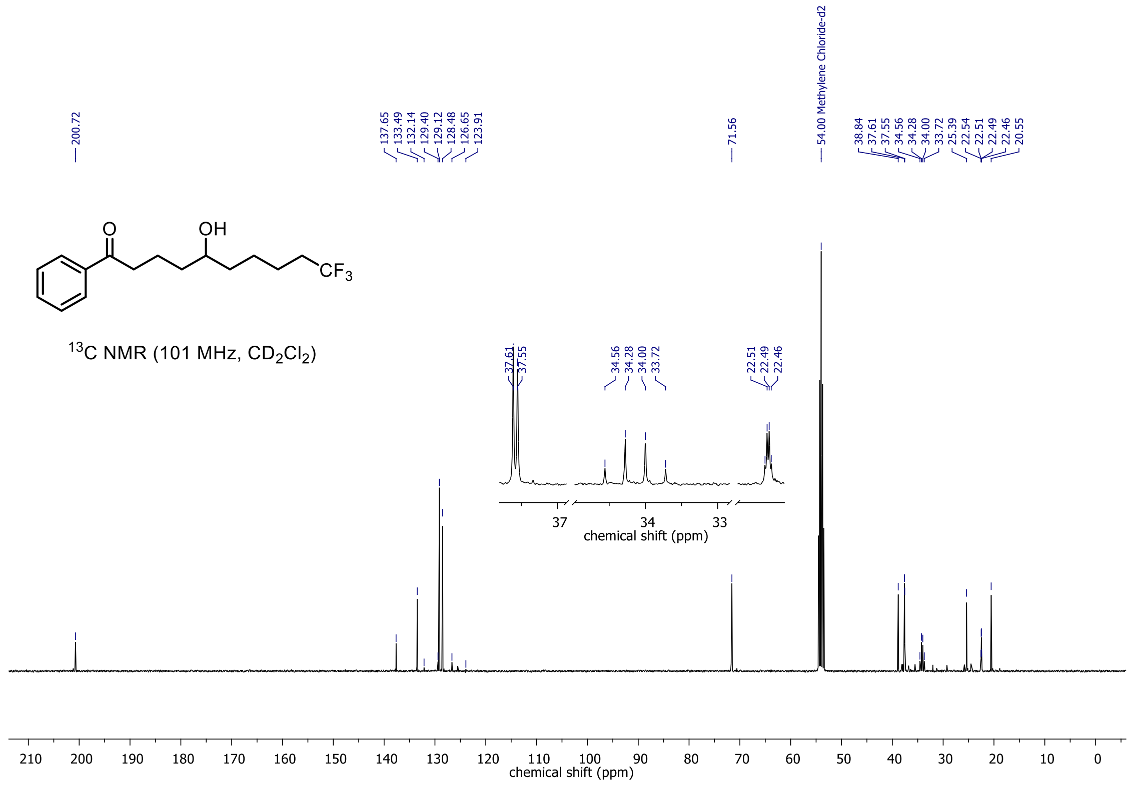

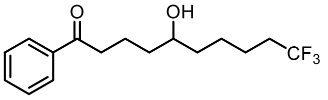

^{19}F NMR (377 MHz, CD_2Cl_2)

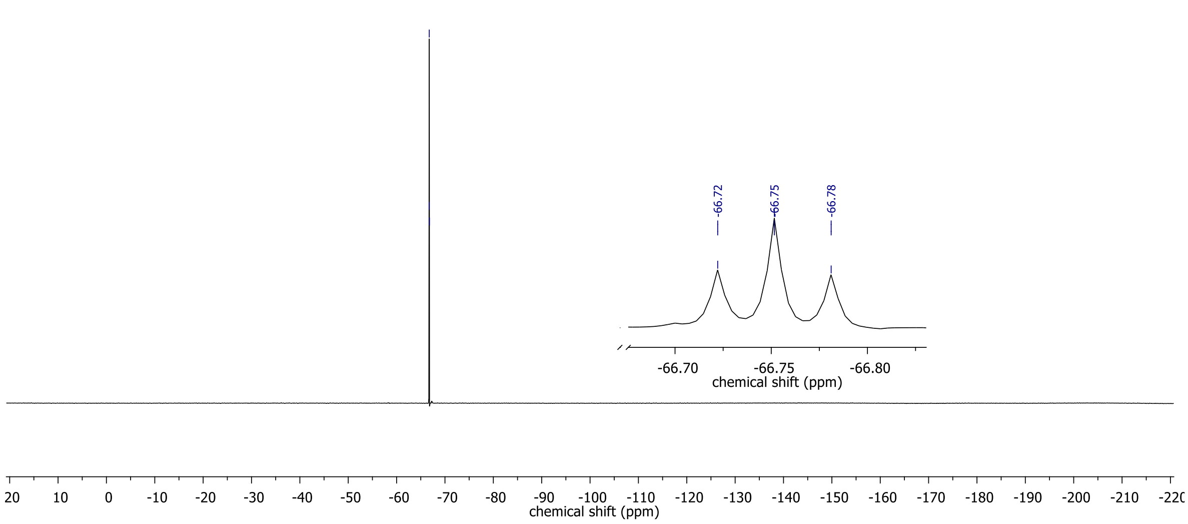

5-Hydroxy-1,16-diphenylhexadecane-1,16-dione (1g)

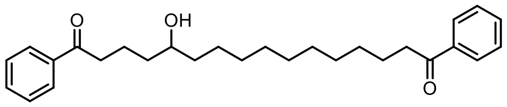

The title compound was prepared following general procedure F using 1,16-diphenylhexadec-8-ene-1,16-dione (37.7 mg, 0.10 mmol, 1.00 equiv.) and TfOH (27 µL, 0.30 mmol, 3.00 equiv.). Purification by flash column chromatography (0 – 100% EtOAc in heptanes) afforded the title compound (22 mg, 56 µmol, 56%) as a colorless solid and a mixture of regioisomers (δ:γ = 95:5).

Crude product: Neither NMR yield nor r.r. could be determined due to signal overlap.

A full list of signals arising from the minor γ-regioisomer can be found in Section 2.5.

^{1}H NMR (400 MHz, CD_2Cl_2): δ 7.95 (m, 4H), 7.61 – 7.53 (m, 2H), 7.51 – 7.43 (m, 4H), 3.64 – 3.54 (m, 1H), 3.01 (t, *J* = 7.2 Hz, 2H), 2.98 – 2.93 (t, *J* = 7.2 Hz, 2H), 1.90 – 1.75 (m, 2H), 1.71 (m, 3H), 1.49 (m, 5H), 1.32 (m, 13H).

^{13}C NMR (101 MHz, CD_2Cl_2): δ 200.83, 200.75, 137.8, 137.7, 133.4, 133.3, 129.10 (2C), 129.07 (2C), 128.5 (4C), 71.9, 39.1, 39.0, 38.2, 37.5, 30.3, 30.2, 30.12, 30.06, 30.04, 29.9, 26.2, 24.9, 20.7.

IR (neat) ν_{max}: 3411, 2914, 2848, 2360, 1712, 1684, 1446, 1365, 1249, 1210, 732, 687, 569, 531.

HRMS (ESI$^+$): exact mass calculated for $[M+Na]^+$ $(C_{28}H_{38}O_3Na)^+$ requires *m*/*z* 445.2713, found *m*/*z* 445.2709.

5-Hydroxy-1,16-diphenylhexadecane-1,16-dione (1g)

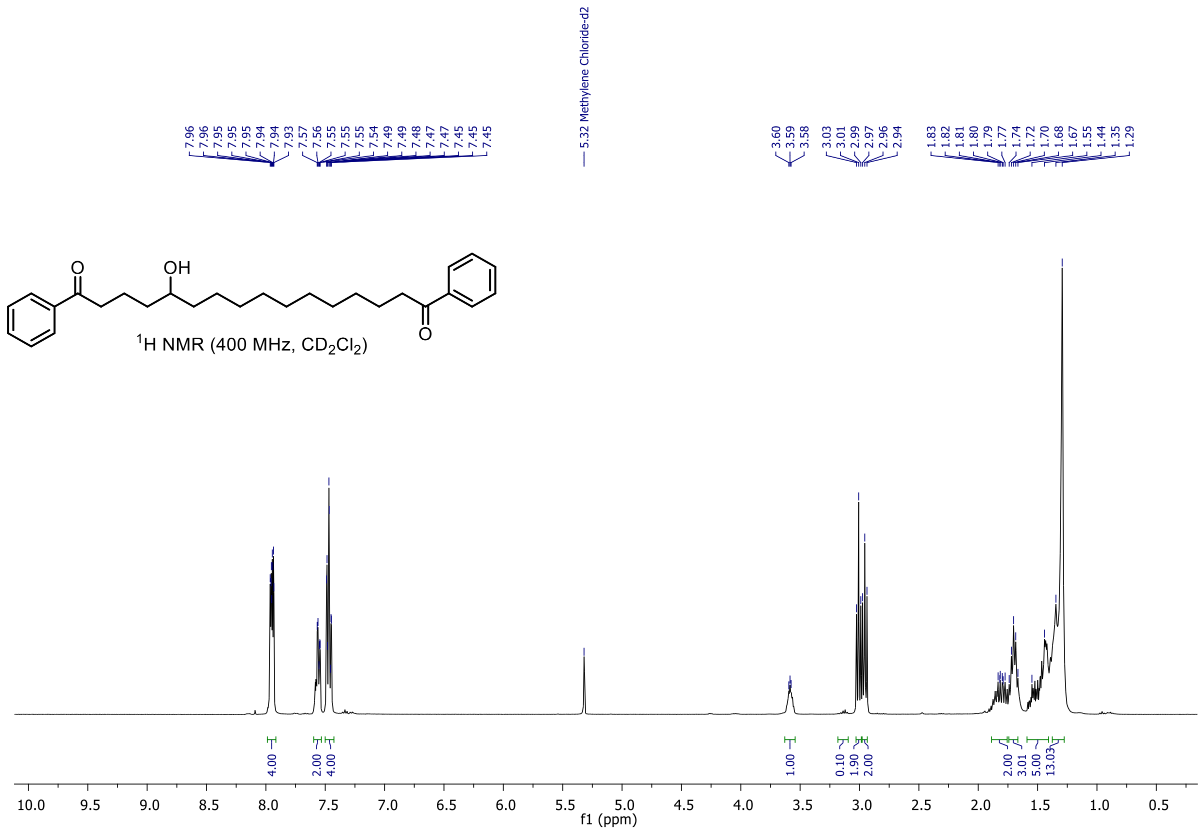

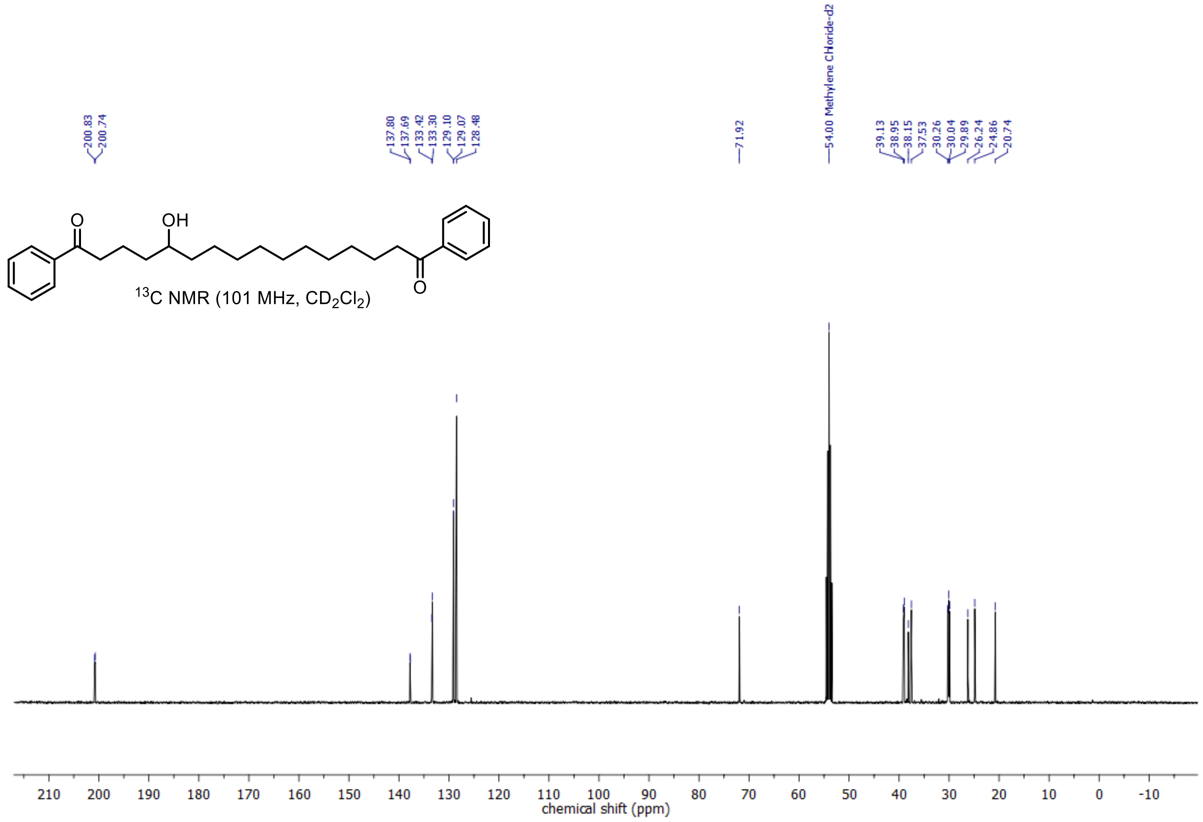

5-Hydroxy-1-phenyloctadecan-1-one (1h)

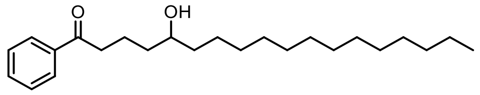

The title compound was prepared following general procedure F using (*Z*)-1-phenyloctadec-9-en-1-one (34.3 mg, 0.10 mmol, 1.00 equiv.) and TfOH (27 µL, 0.30 mmol, 3.00 equiv.). Purification by flash column chromatography (0 – 50% EtOAc in heptanes) afforded the title compound (14.0 mg, 39 µmol, 39%) as a colorless solid and a single regioisomer.

Crude product: 56% NMR yield, r.r. (δ_{OH}: γ_{OH}) = 91:9.

^{1}H NMR (600 MHz, CD_2Cl_2): δ 7.97 – 7.92 (m, 2H), 7.59 – 7.53 (m, 1H), 7.50 – 7.45 (m, 2H), 3.63 – 3.51 (m, 1H), 3.01 (t, *J* = 7.2 Hz, 2H), 1.90 – 1.74 (m, 2H), 1.61 (d, *J* = 4.7 Hz, 1H), 1.56 – 1.36 (m, 6H), 1.32 – 1.24 (m, 20H), 0.90 – 0.85 (m, 3H).

^{13}C NMR (151 MHz, CD_2Cl_2): δ 200.8, 137.7, 133.4, 129.1 (2C), 128.5 (2C), 72.0, 39.0, 38.2, 37.5, 32.5, 30.3 (4C), 30.2 (3C), 29.9, 26.3, 23.3, 20.7, 14.5.

IR (neat) ν_{max}: 2916, 2848, 1717, 1682, 1273, 1254, 1110, 1070, 731, 711, 688.

HRMS (ESI$^+$): exact mass calculated for $[M+Na]^+$ ($C_{24}H_{40}O_2Na)^+$ requires *m/z* 383.2921, found *m/z* 383.2914.

SC-XRD: see Section 5.

5-Hydroxy-1-phenyloctadecan-1-one (1h)

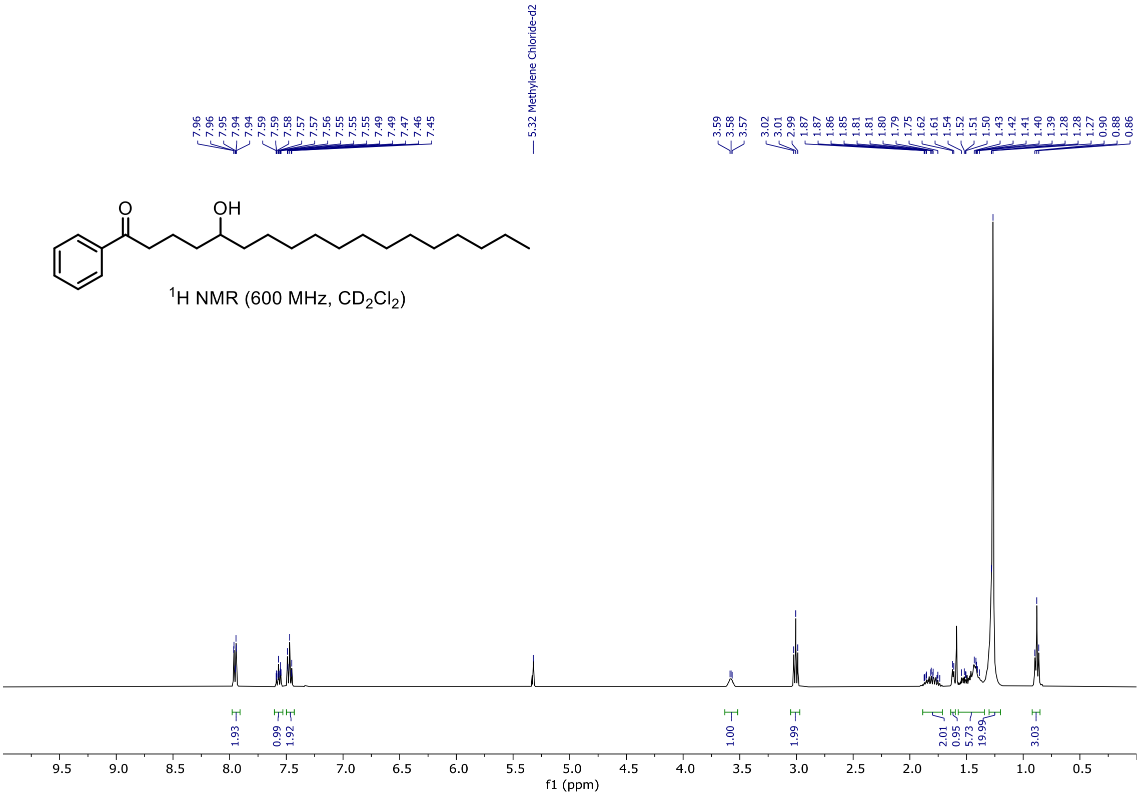

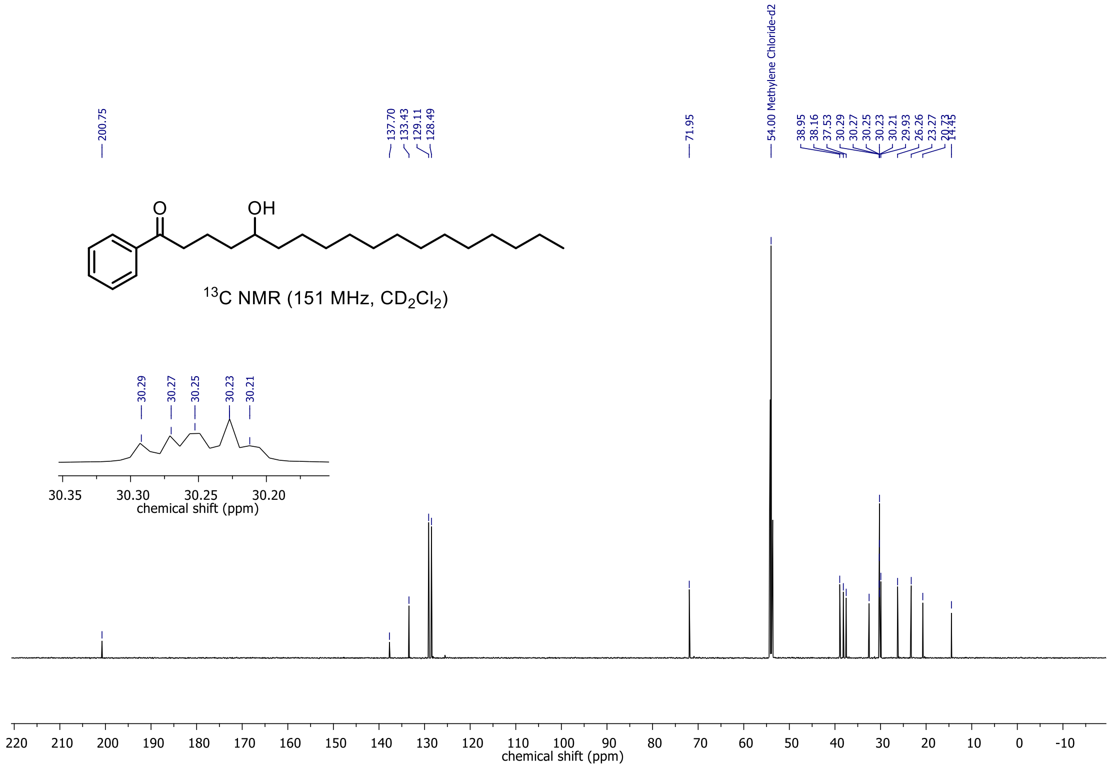

5-Hydroxy-1,2-diphenyldecan-1-one (1i)

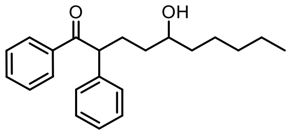

The title compound was prepared following general procedure F using 1,2-diphenyldec-9-en-1-one (30.6 mg, 0.10 mmol, 1.00 equiv.) and TfOH (27 µL, 0.30 mmol, 3.00 equiv.). Purification by flash column chromatography (0 – 50% EtOAc in heptanes) afforded the title compound (15.6 mg, 48 µmol, 48%) as a colorless oil and a single regioisomer, consisting of two diastereoisomers (approximate d.r. value based on ^{13}C NMR = 69:31).

Crude product: 68% NMR yield, r.r. could not determined, d.r. = 62:38.

^{1}H NMR (400 MHz, CD_2Cl_2): δ 8.00 – 7.92 (m, 2H), 7.53 – 7.47 (m, 1H), 7.43 – 7.37 (m, 2H), 7.33 – 7.28 (m, 4H), 7.23 – 7.18 (m, 1H), 4.59 (t, *J* = 7.3 Hz, 1H), 3.62 – 3.51 (m, 1H), 2.36 – 2.15 (m, 1H), 2.00 – 1.80 (m, 1H), 1.53 – 1.21 (m, 11H), 0.91 – 0.82 (m, 3H).

An asterisk (*) denotes signals that unambiguously arise from the minor diastereomer. Due to low abundance and signal overlap, some ^{13}C NMR signals could not be unambiguously assigned to the major or minor diastereomers.

^{13}C NMR (101 MHz, CD_2Cl_2): δ 200.3, 200.1*, 140.26*, 140.24, 137.33*, 137.29, 133.25, 133.22*, 129.27 (2C), 129.26* (2C) 128.98 (2C), 128.96* (2C), 128.92* (2C), 128.91 (2C), 128.68* (2C), 128.66 (2C), 127.38, 127.37*, 72.2*, 71.8, 53.94, 53.92, 37.9, 37.8*, 35.8, 35.7*, 32.28*, 32.27, 30.5*, 30.4, 25.71, 25.65*, 23.04, 23.03*, 14.22, 14.21*.f

IR (neat) ν_{max}: 3389, 2953, 2926, 1678, 1447, 754, 693.

HRMS (ESI$^+$): exact mass calculated for $[M+Na]^+$ $(C_{22}H_{28}O_2Na)^+$ requires *m/z* 347.1982, found *m/z* 347.1979.

5-Hydroxy-1,2-diphenyldecan-1-one (1i)

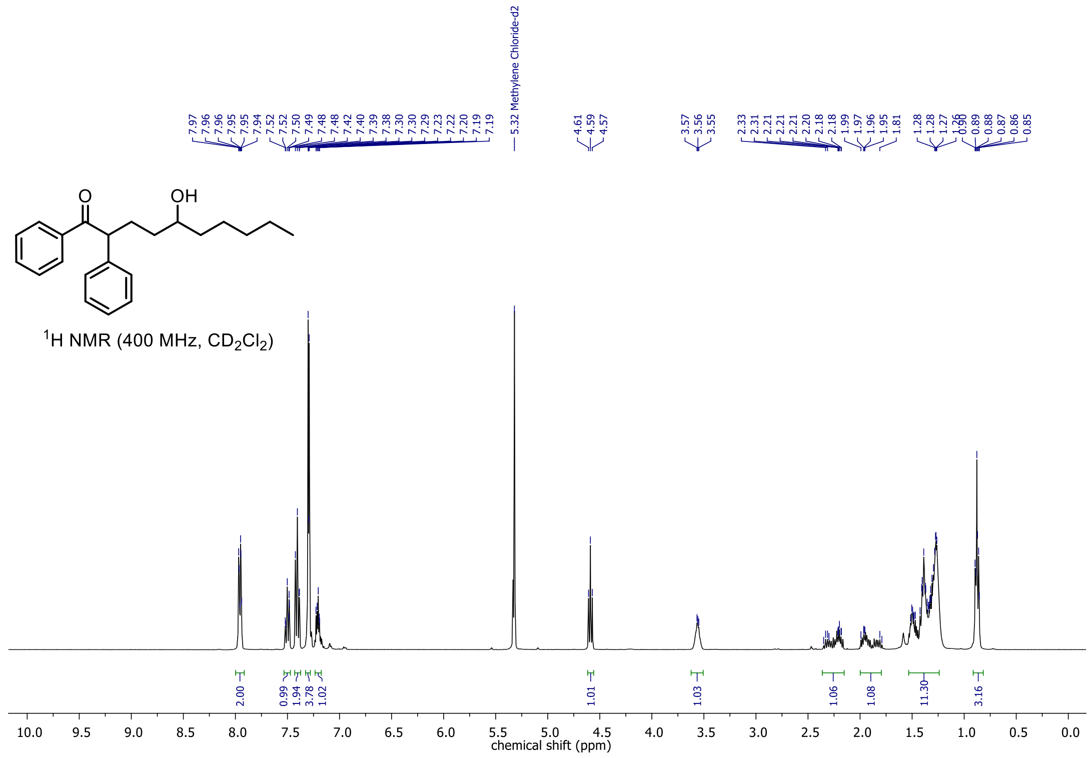

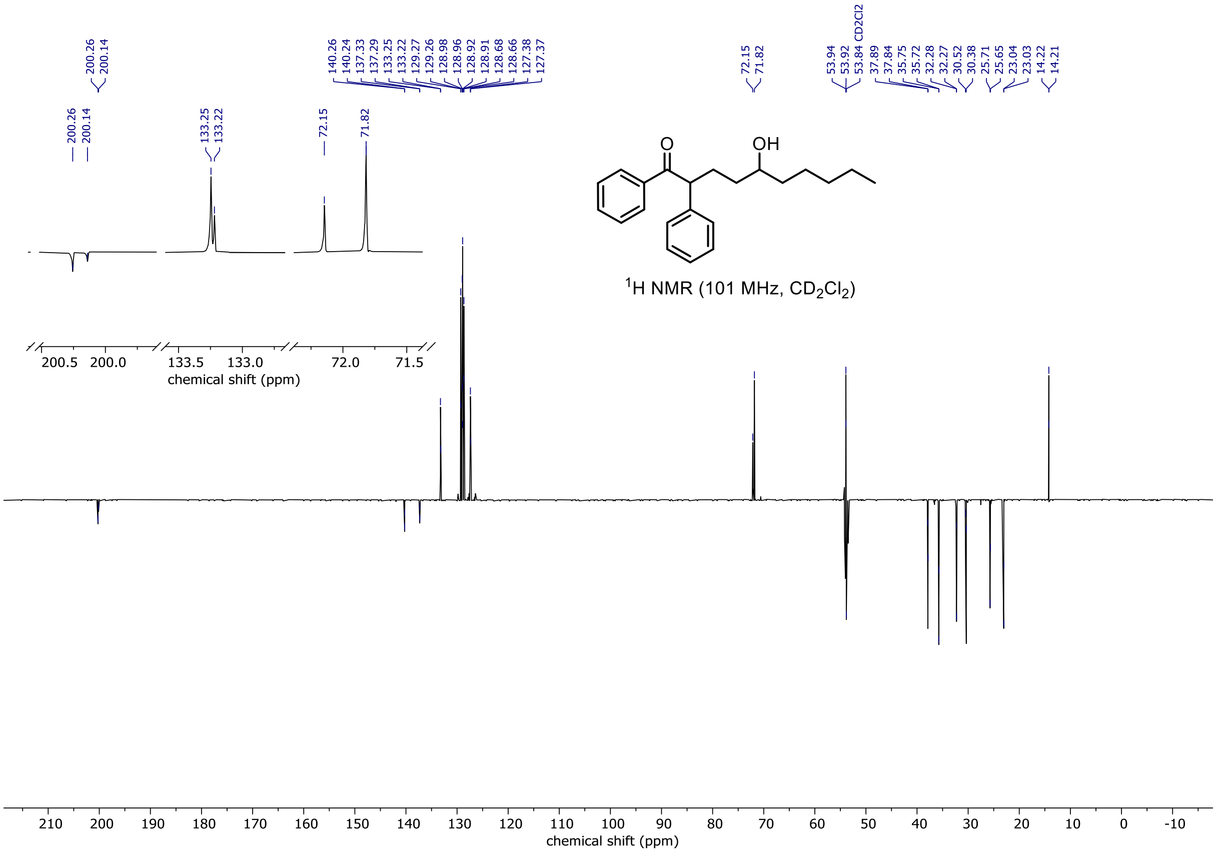

5-Hydroxy-1-(*o*-tolyl)nonan-1-one (1j)

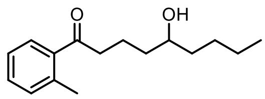

The title compound was prepared following general procedure F using 1-(*o*-tolyl)non-8-en-1-one (23 mg, 0.10 mmol, 1.00 equiv.) and TfOH (27 µL, 0.30 mmol, 3.00 equiv.). Purification by flash column chromatography (0 – 100% EtOAc in heptanes) afforded the title compound (19.6 mg, 79 µmol, 79%) as a colorless solid and a single regioisomer.

Crude product: 86% NMR yield, r.r. (δ_{OH}:γ_{OH}) = 92:8.

^{1}H NMR (400 MHz, $(CD_3)_2CO$): δ 7.72 (d, *J* = 7.8 Hz, 1H), 7.39 (td, *J* = 7.5, 1.3 Hz, 1H), 7.34 – 7.25 (m, 2H), 3.60 – 3.51 (m, 1H), 3.39 (d, *J* = 5.4 Hz, 1H), 3.02 – 2.87 (m, 2H), 2.43 (s, 3H), 1.90 – 1.77 (m, 1H), 1.77 – 1.66 (m, 1H), 1.54 – 1.38 (m, 5H), 1.37 – 1.27 (m, 3H), 0.89 (t, *J* = 7.1 Hz, 3H).

^{13}C NMR (101 MHz, $(CD_3)_2CO$): δ 204.9, 139.7, 138.0, 132.4, 131.7, 129.1, 126.6, 71.2, 42.2, 38.3, 37.9, 28.7, 23.5, 21.5, 21.1, 14.1.

IR (neat) ν_{max}: 3440, 2954, 2929, 2859, 2359, 1682, 1455, 1407, 1379, 1285, 1228, 1127, 971, 754, 655.

HRMS (ESI$^+$): exact mass calculated for $[M+Na]^+$ $(C_{16}H_{24}O_2Na)^+$ requires *m/z* 271.1669, found *m/z* 271.1661.

5-Hydroxy-1-(*o*-tolyl)nonan-1-one (1j)

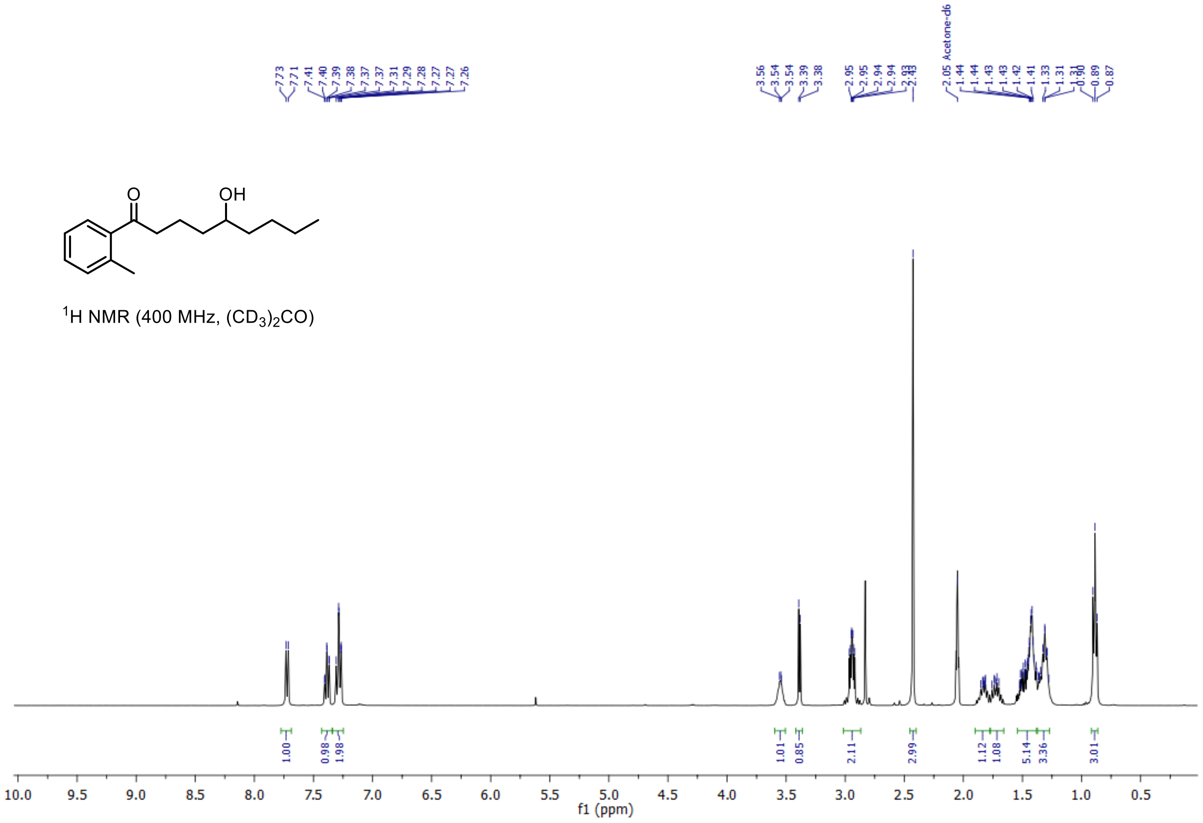

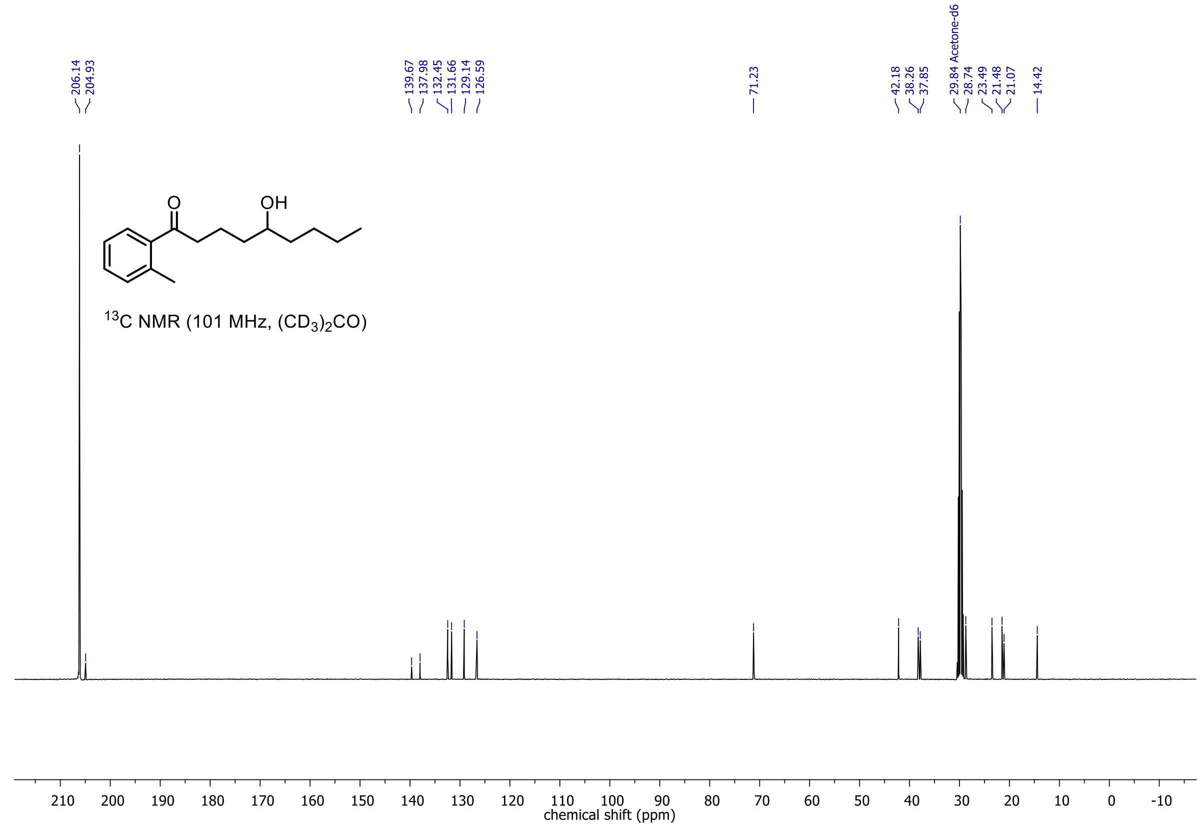

1-(4-(*tert*-Butyl)phenyl)-5-hydroxynonan-1-one (1k)

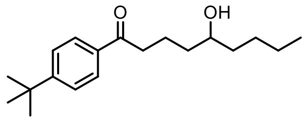

The title compound was prepared following general procedure F using 1-(4-(*tert*-butyl)phenyl)non-8-en-1-one (27.2 mg, 0.10 mmol, 1.00 equiv.) and TfOH (27 µL, 0.30 mmol, 3.00 equiv.). Purification by flash column chromatography (0 – 70% EtOAc in heptanes) afforded the title compound (19.0 mg, 65 µmol, 65%) as a colorless oil and a single regioisomer.

Crude product: 82% NMR yield, r.r. (δ_{OH}: ε_{OH}) = 92:8.

^{1}H NMR (400 MHz, CD_2Cl_2): δ 7.92 – 7.85 (m, 2H), 7.52 – 7.46 (m, 2H), 3.65 – 3.51 (m, 1H), 2.98 (t, *J* = 7.2 Hz, 2H), 1.89 – 1.26 (m, 20H), 0.90 (t, *J* = 7.1 Hz, 3H).

^{13}C NMR (101 MHz, CD_2Cl_2): δ 200.5, 157.2, 135.1, 128.4 (2C), 126.1 (2C), 71.9, 38.9, 37.8, 37.6, 35.5, 31.4 (3C), 28.5, 23.3, 20.8, 14.4.

IR (neat) ν_{max}: 2956, 2930, 2869, 1677, 1605, 1461, 1406, 1363, 1268, 1231, 1190, 1108, 981, 822.

HRMS (ESI$^+$): exact mass calculated for $[M+Na]^+$ $(C_{19}H_{30}O_2Na)^+$ requires *m/z* 313.2138, found *m/z* 313.2132.

1-(4-(*tert*-Butyl)phenyl)-5-hydroxynonan-1-one (1k)

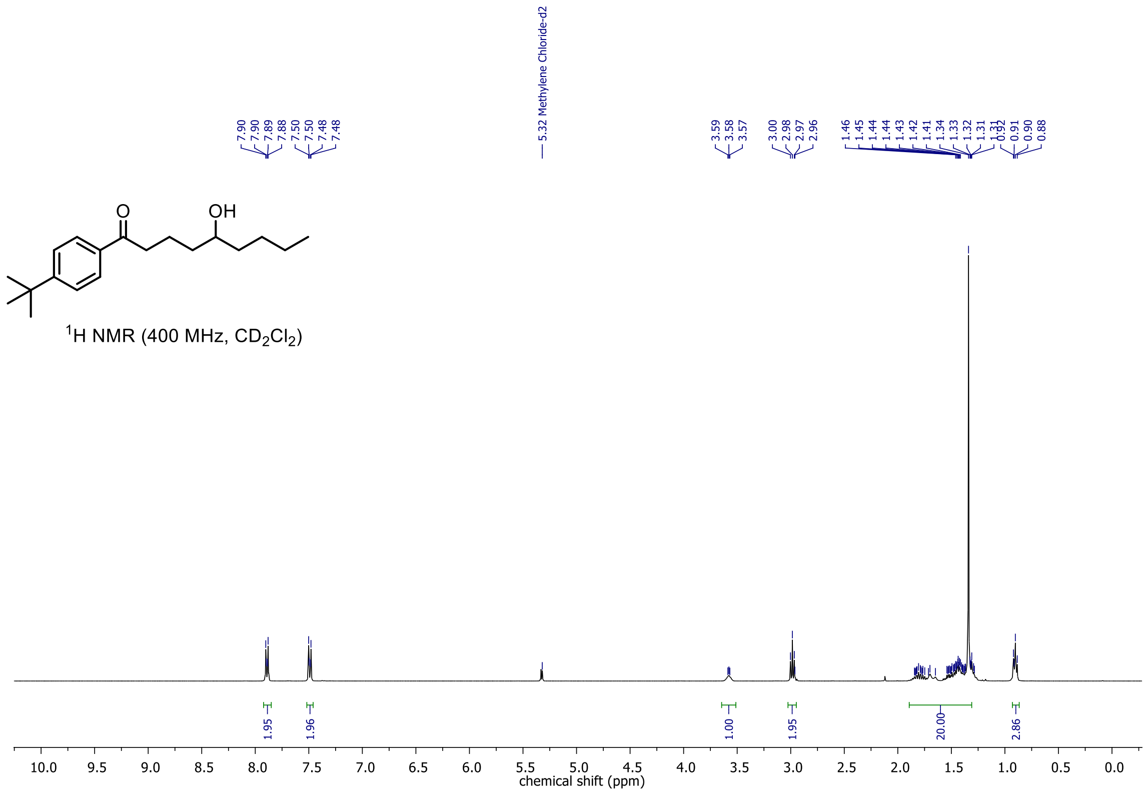

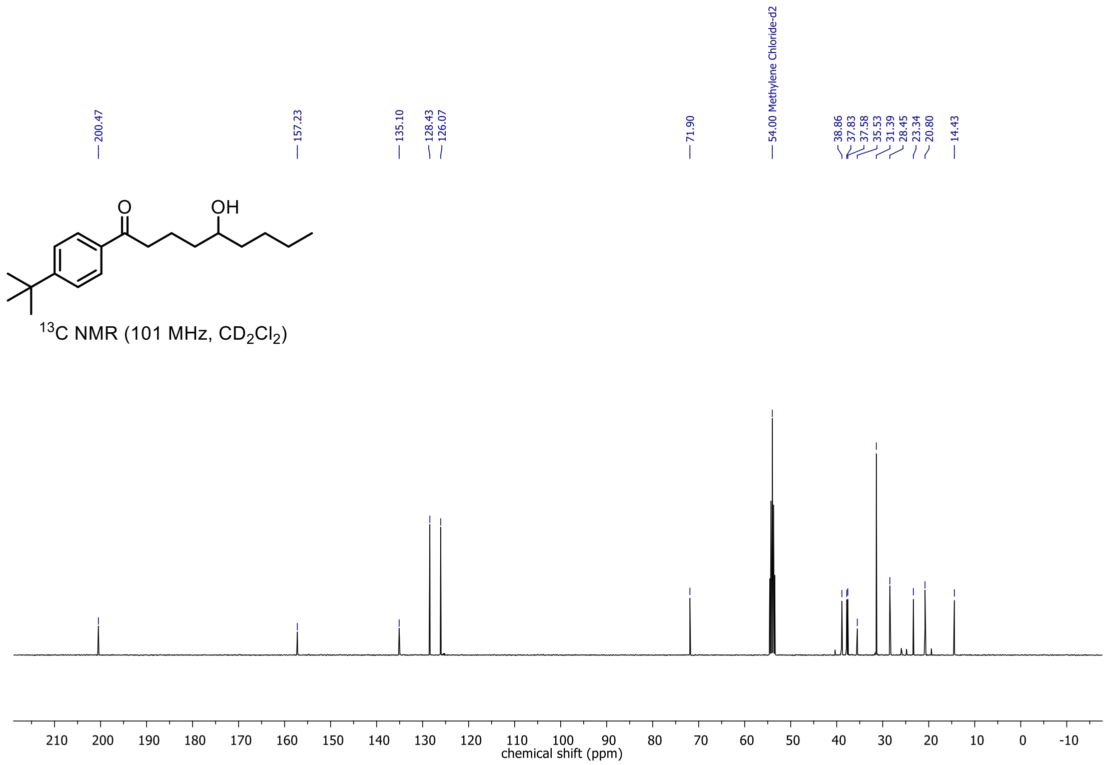

5-Hydroxy-1-(4-(trifluoromethyl)phenyl)nonan-1-one (1l)

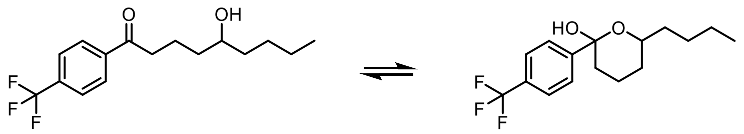

4.8 : 1

The title compound was prepared following general procedure F with a modification to the reaction temperature and time (45 °C instead of 65 °C for 2 h) using 1-(4-(trifluoromethyl)phenyl)non-8-en-1-one (28.4 mg, 0.10 mmol, 1.00 equiv.) and TfOH (27 µL, 0.30 mmol, 3.00 equiv.). Purification by flash column chromatography (10 – 40% EtOAc in heptanes with 1% triethylamine additive) afforded the tile compound (20.0 mg, 66 µmol, 66%) as a colorless oil and a mixture of regioisomers (δ:γ = 92:8). The δ-hydroxy ketone is in equilibrium with its hemiacetal form (ratio = 4.8:1).

Crude product: 88% NMR yield, r.r. ($\delta_{OH+Hemiacetal}$:γ_{OH}) = 92 : 8

An asterisk (*) denotes signals that unambiguously arise from the hemiacetal. Although the γ-isomer was not prepared independently, the minor product obtained in this reaction was determined to be the γ-isomer by analogy (chemical shifts & splitting patterns) with the other reported compounds.

^{1}H NMR (400 MHz, CD_2Cl_2) δ 8.11 – 8.04 (m, 1.6H), 7.78 – 7.66 (m, 2H), 7.61 (d, *J* = 8.2 Hz, 0.4H*), 4.08 – 4.01 (m, 0.2H*), 3.69 – 3.53 (m, 0.8H), 3.24 – 3.11 (m, 0.2H), 3.04 (t, *J* = 7.2 Hz, 1.5H), 2.05 – 1.73 (m, 2.3H), 1.60 – 1.20 (m, 9H), 0.97 – 0.84 (m, 3H).

Due to low abundance, only signals arising from the major component (δ-hydroxy ketone) are reported in ^{13}C NMR.

^{13}C NMR (101 MHz, CD_2Cl_2) δ 199.7 (C), 140.3 (C), 134.1 (q, *J* = 33.1 Hz, C), 128.8 (2CH), 126.0 (q, *J* = 3.9 Hz, 2CH), 124.2 (q, *J* = 272.0 Hz, C), 71.8 (CH), 39.1 (CH_2), 37.7 (CH_2), 37.2 (CH_2), 28.3 (CH_2), 23.2 (CH_2), 20.5 (CH_2), 14.3 (CH_3).

^{19}F NMR (377 MHz, CD_2Cl_2) δ -62.74 (0.6F*), -63.41 (3F).

IR (neat) ν_{max}: 3383, 2955, 2930, 2860, 1688, 1511, 1323, 1166, 1126, 1108, 1066, 1016, 822.

HRMS (ESI$^+$): exact mass calculated for $[M+Na]^+$ $(C_{16}H_{21}F_3O_2Na)^+$ requires *m*/*z* 325.1386, found *m*/*z* 325.1380.

5-Hydroxy-1-(4-(trifluoromethyl)phenyl)nonan-1-one (1l)

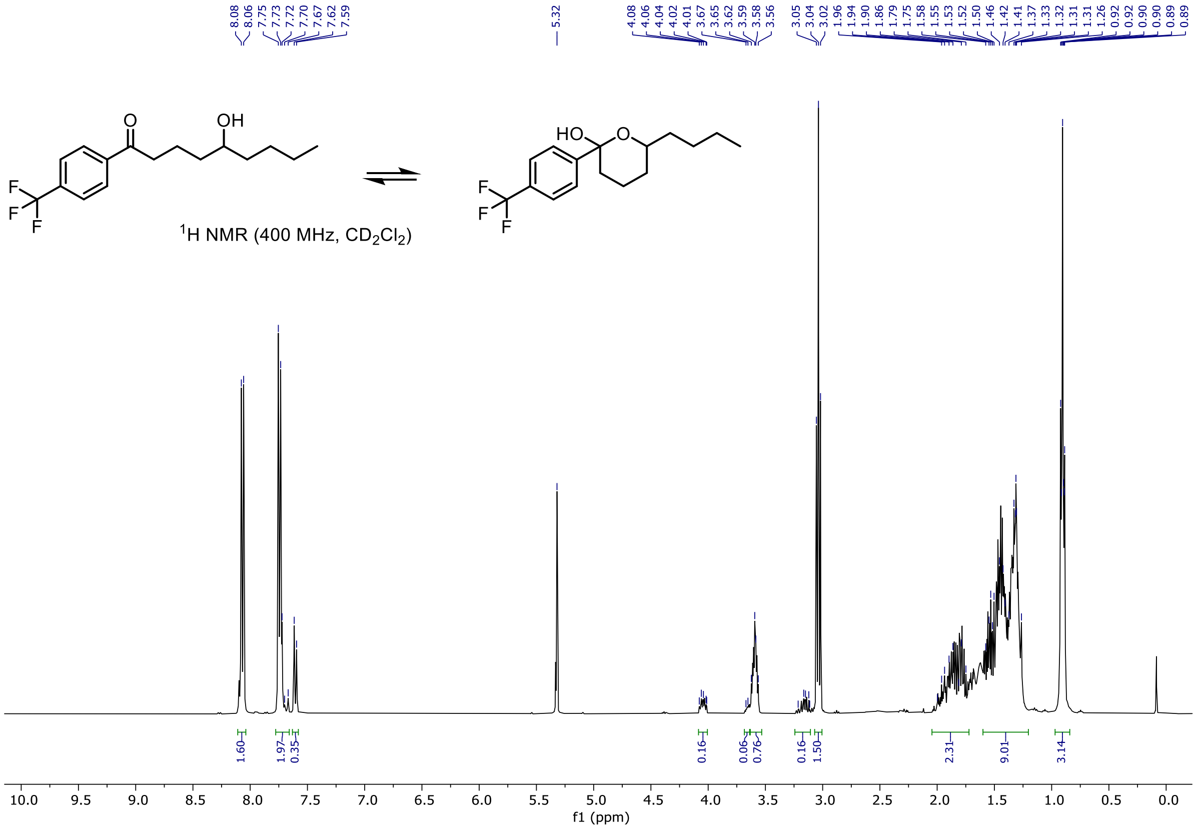

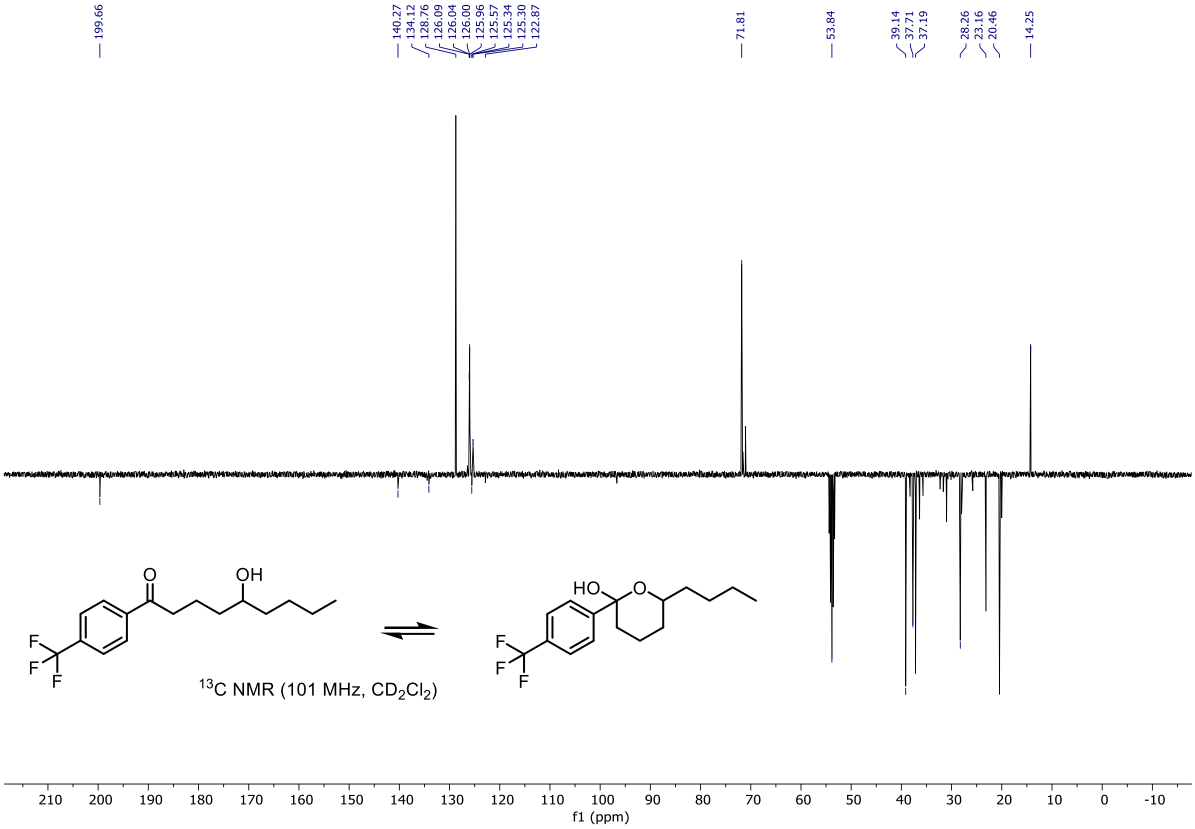

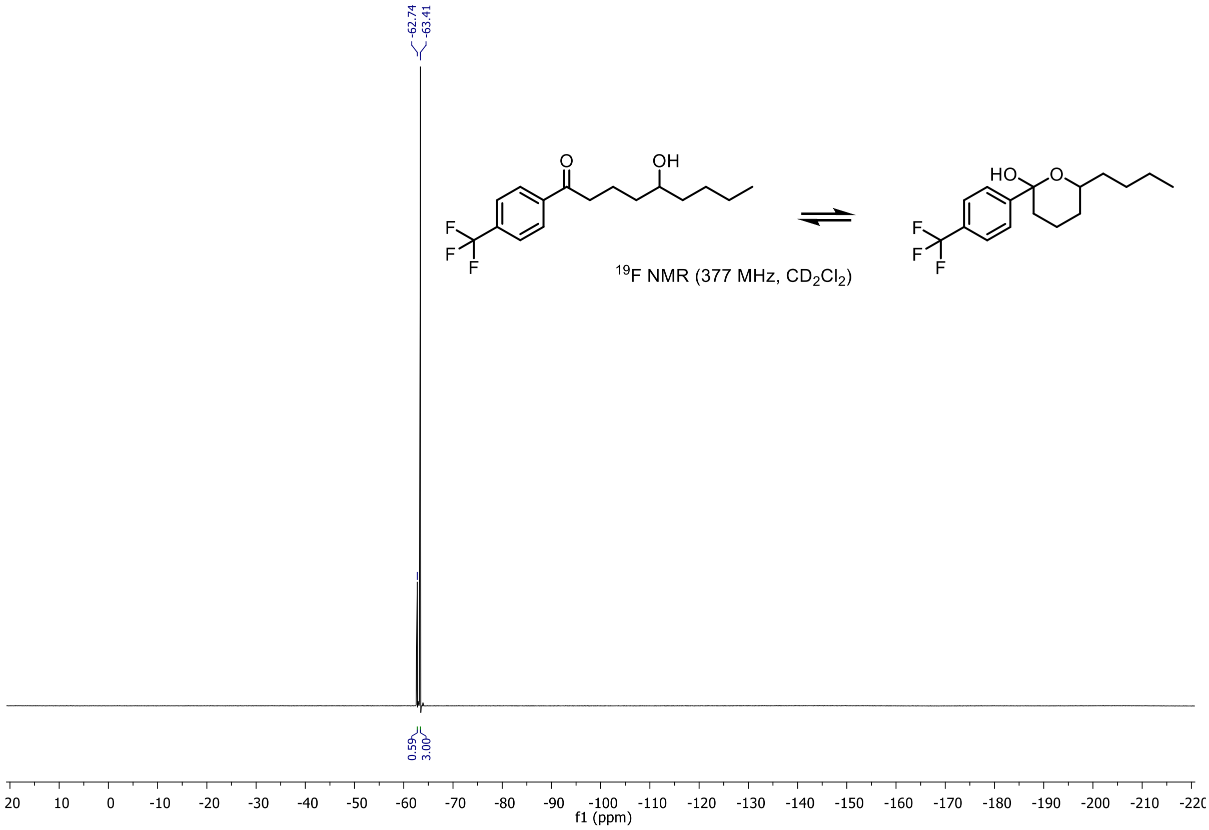

-62.74
-63.41
O
OH
HO
O
F
F
F
F
F
F
^{19}F NMR (377 MHz, CD_2Cl_2)
0.59
3.00
20 10 0 -10 -20 -30 -40 -50 -60 -70 -80 -90 -100 -110 -120 -130 -140 -150 -160 -170 -180 -190 -200 -210 -220
f1 (ppm)

5-Hydroxy-1-(4-(trifluoromethoxy)phenyl)nonan-1-one (1m)

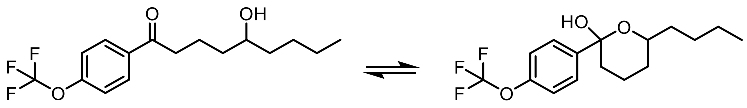

14.2 : 1

The title compound was prepared following general procedure F with a modification to the reaction temperature and time (45 °C instead of 65 °C for 2 h) using 1-(4-(trifluoromethoxy)phenyl)non-8-en-1-one (30.0 mg, 0.10 mmol, 1.00 equiv.) and TfOH (27 µL, 0.30 mmol, 3.00 equiv.). Purification by flash column chromatography (10 – 40% EtOAc in heptanes with 1% triethylamine additive) afforded the title compound (26.0 mg, 82 µmol, 82%) as a colorless oil and a mixture of regioisomers (δ:γ = 91:9). The δ-hydroxy ketone is in equilibrium with its hemiacetal form (ratio = 14.2:1).

Crude product: 93% NMR yield; r.r. ($\delta_{OH+Hemiacetal}$:γ_{OH}) = 93 : 7

An asterisk (*) denotes signals that unambiguously arise from the hemiacetal. A full list of signals arising from the minor γ-regioisomer can be found in Section 2.5.

^{1}H NMR (600 MHz, CD_2Cl_2) δ 8.06 – 7.98 (m, 1.8H), 7.65 – 7.62 (m, 0.2H*), 7.31 (dd, *J* = 8.8, 0.9 Hz, 1.8H), 7.19 (dd, *J* = 8.8, 0.9 Hz, 0.2H*), 4.06 – 4.00 (m, 0.1H*), 3.64 (app br s, 0.1H), 3.59 (app br s, 0.8H), 3.17 – 3.06 (m, 0.2H), 3.00 (t, *J* = 7.2 Hz, 1.7H), 2.43 (br s, 0.1H*), 1.98 – 1.66 (m, 2H), 1.56 – 1.26 (m, 9H), 0.93 – 0.87 (m, 3H).

Due to low abundance, only signals arising from the major component (δ-hydroxy ketone) are reported in ^{13}C NMR.

^{13}C NMR (151 MHz, CD_2Cl_2) δ 199.1 (C), 152.8 (q, *J* = 1.7 Hz, C), 135.9 (C), 130.4 (2CH), 120.8 (2CH), 120.7 (q, *J* = 258.4 Hz), 71.8 (CH), 38.9 (CH_2), 37.7 (CH_2), 37.3 (CH_2), 28.3 (CH_2), 23.2 (CH_2), 20.5 (CH_2), 14.25 (CH_3).

^{19}F NMR (376 MHz, CD_2Cl_2) δ -58.03 (3F), -58.24 (0.2F*).

IR (neat) ν_{max}: 3421, 2956, 2932, 2862, 1685, 1602, 1254, 1208, 1164, 982.

HRMS (ESI$^+$): exact mass calculated for $[M+Na]^+$ $(C_{16}H_{21}F_3O_3Na)^+$ requires *m/z* 341.1335, found *m/z* 341.1326.

5-Hydroxy-1-(4-(trifluoromethoxy)phenyl)nonan-1-one (1m)

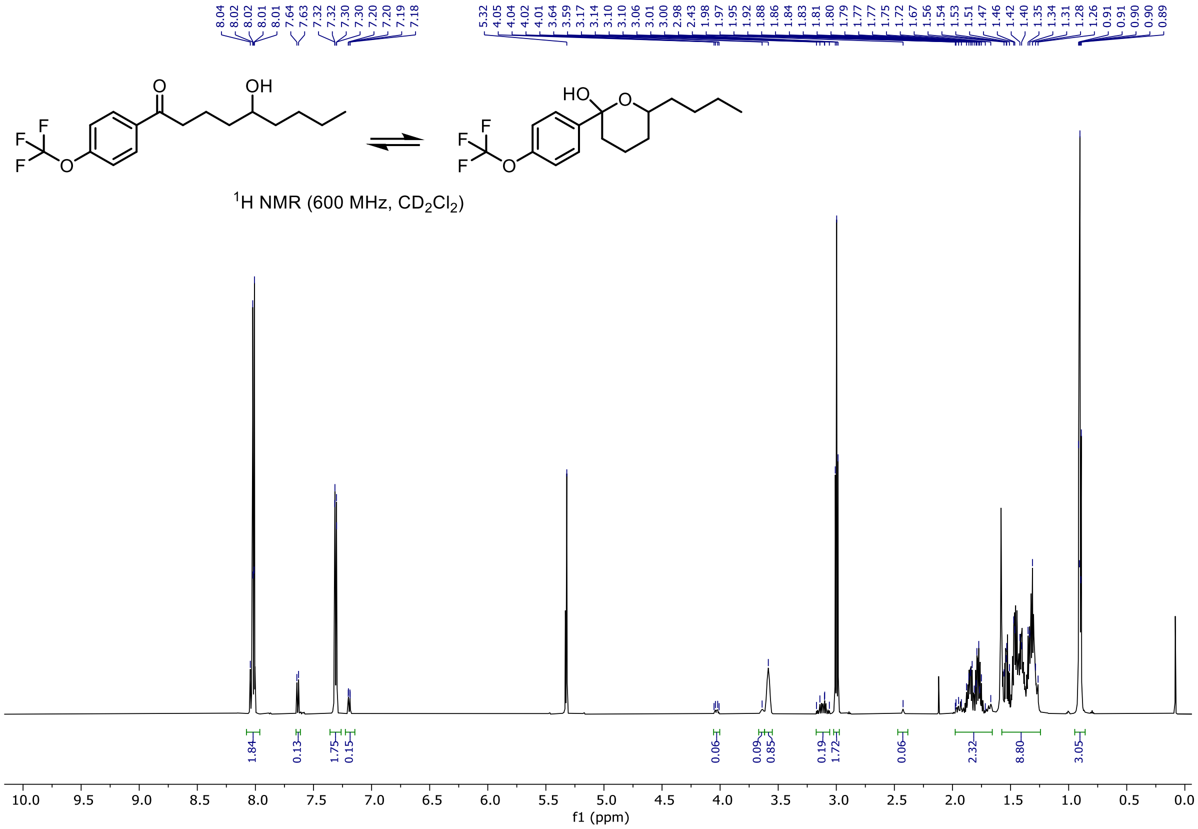

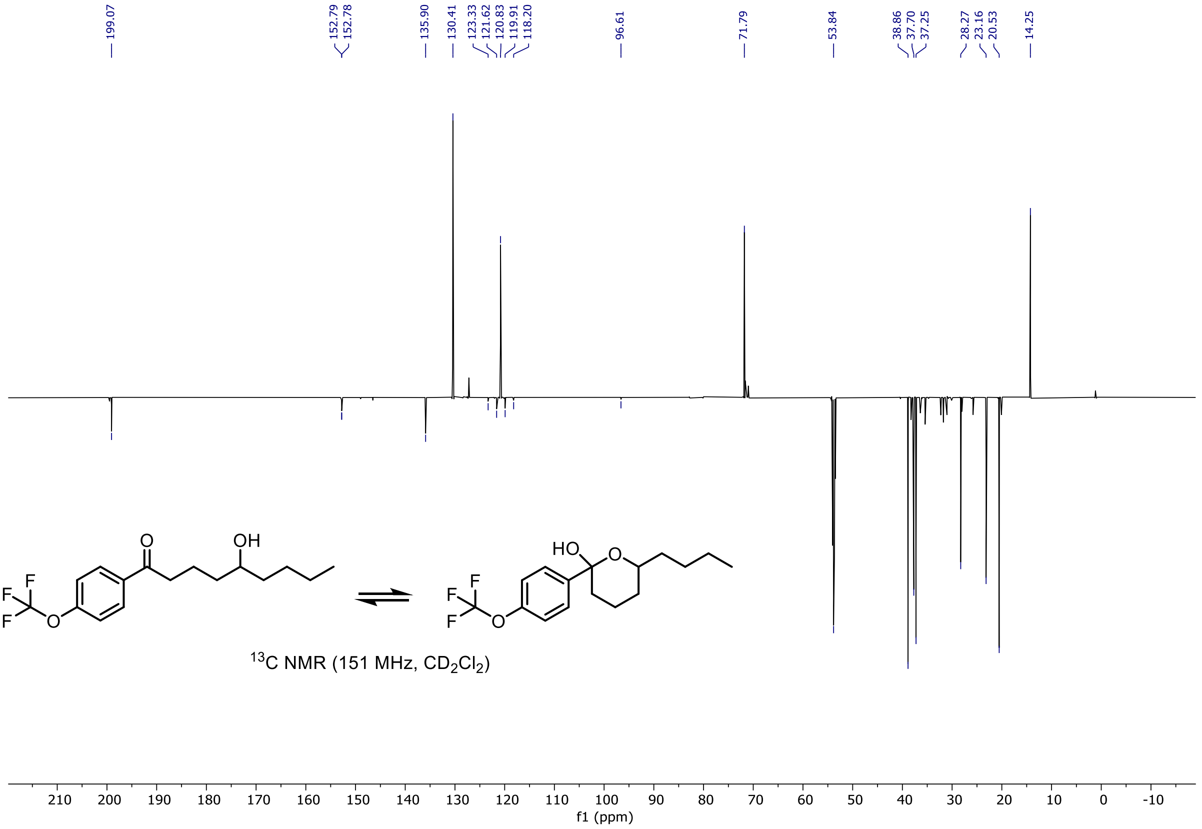

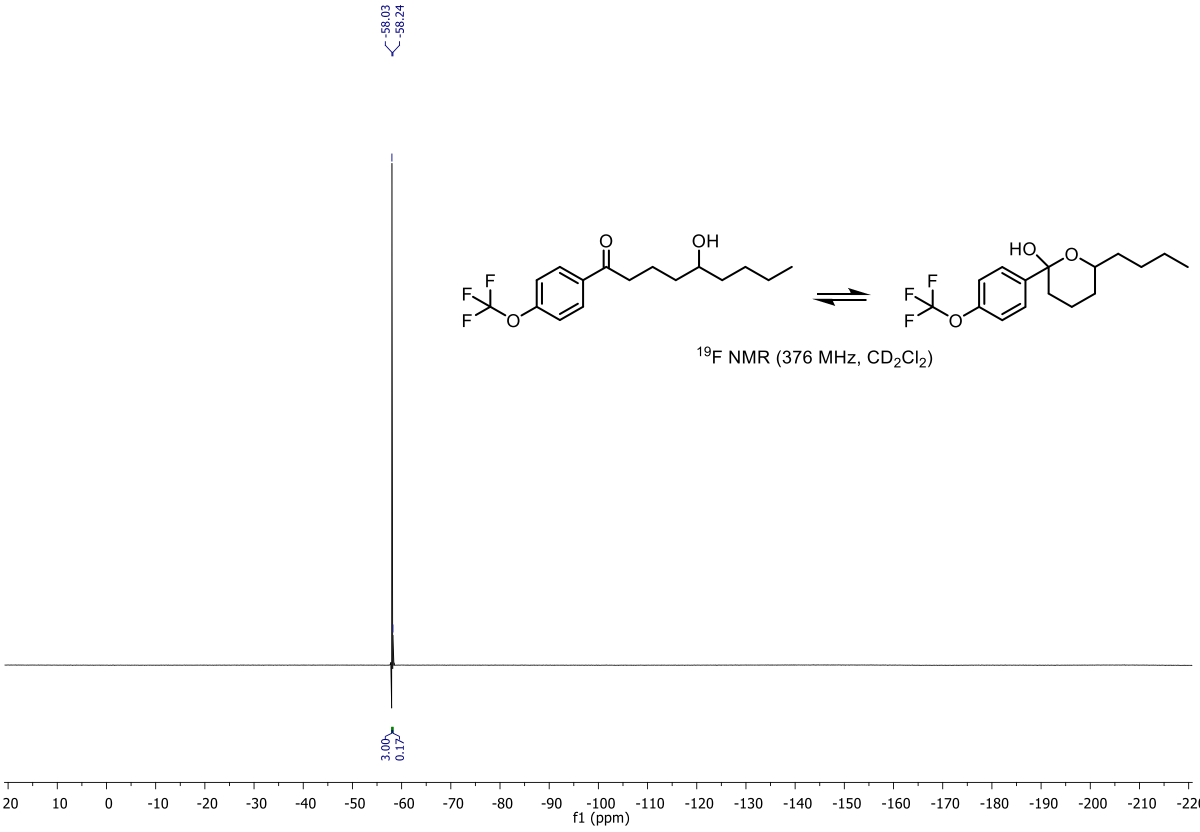

-58.03
-58.24
3.00
0.17
^{19}F NMR (376 MHz, CD_2Cl_2)
f1 (ppm)

Methyl 4-(5-hydroxynonanoyl)benzoate (1n)

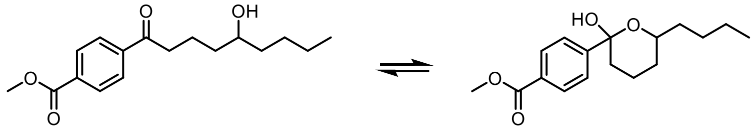

6.7 : 1

The title compound was prepared following general procedure F with a modification to the reaction temperature and time (45 °C instead of 65 °C for 2 h) using methyl 4-(non-8-enoyl)benzoate (27.4 mg, 0.10 mmol, 1.00 equiv.) and TfOH (27 µL, 0.30 mmol, 3.00 equiv.). Purification by flash column chromatography (8 – 40% EtOAc in heptanes with 1% triethylamine additive) afforded the title compound (21.1 mg, 72 µmol, 72%) as a colorless oil and a mixture of regioisomers (δ:γ = 90:10). The δ-hydroxy ketone is in equilibrium with its hemiacetal form (ratio = 6.7:1).

Crude product: 90% NMR yield; r.r. ($\delta_{OH+Hemiacetal}$:γ_{OH}) = 91 : 9

An asterisk (*) denotes signals that unambiguously arise from the hemiacetal. Although the γ-isomer was not prepared independently, the minor product obtained in this reaction was determined to be the γ-isomer by analogy (chemical shifts & splitting patterns) with the other reported compounds.

^{1}H NMR (400 MHz, CD_2Cl_2) δ 8.15 – 8.06 (m, 1.7H), 8.01 – 7.96 (m, 2H), 7.69 – 7.64 (m, 0.3H*), 4.08 – 4.00 (m, 0.1H*), 3.92 (s, 2.6H), 3.88 (s, 0.4H*), 3.63 – 3.55 (m, 0.9H), 3.03 (t, *J* = 7.2 Hz, 1.8H), 2.03 – 1.64 (m, 2.2H), 1.55 – 1.24 (m, 9H), 0.94 – 0.86 (m, 3H).

^{13}C NMR (101 MHz, CD_2Cl_2) δ 199.7 (C), 166.7 (C*), 166.1 (C), 151.8 (C*), 140.3 (C), 133.8 (C), 129.7 (2CH), 129.2 (2CH*), 127.9 (2CH), 125.2 (2CH*), 96.4 (C*), 71.4 (CH), 70.6 (CH*), 52.3 (CH_3), 51.9 (CH_3*), 38.8 (CH_2), 37.3 (CH_2), 36.8 (CH_2), 36.6 (CH_2*), 36.0 (CH_2*), 30.6 (CH_2*), 27.9 (CH_2), 27.6 (CH_2*), 22.8 (CH_2*), 22.7 (CH_2), 20.1 (CH_2), 19.6 (CH_2*), 13.8 (CH_3).

IR (neat) ν_{max}: 3401, 2953, 2927, 2871, 2859, 1721, 1678, 1437, 1281, 1194, 1110, 766.

HRMS (ESI$^+$): exact mass calculated for $[M+Na]^+$ $(C_{17}H_{24}O_4Na)^+$ requires *m*/*z* 315.1567, found *m*/*z* 315.1559.

Methyl 4-(5-hydroxynonanoyl)benzoate (1n)

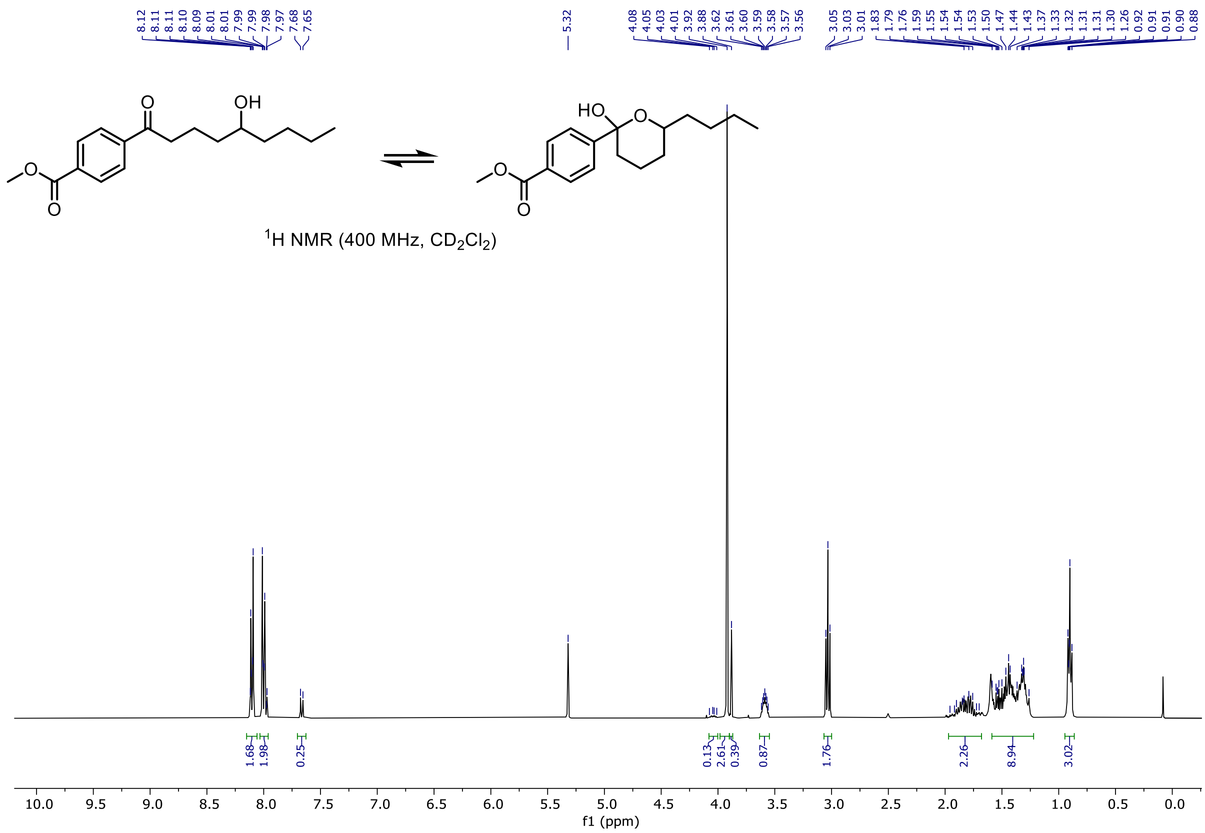

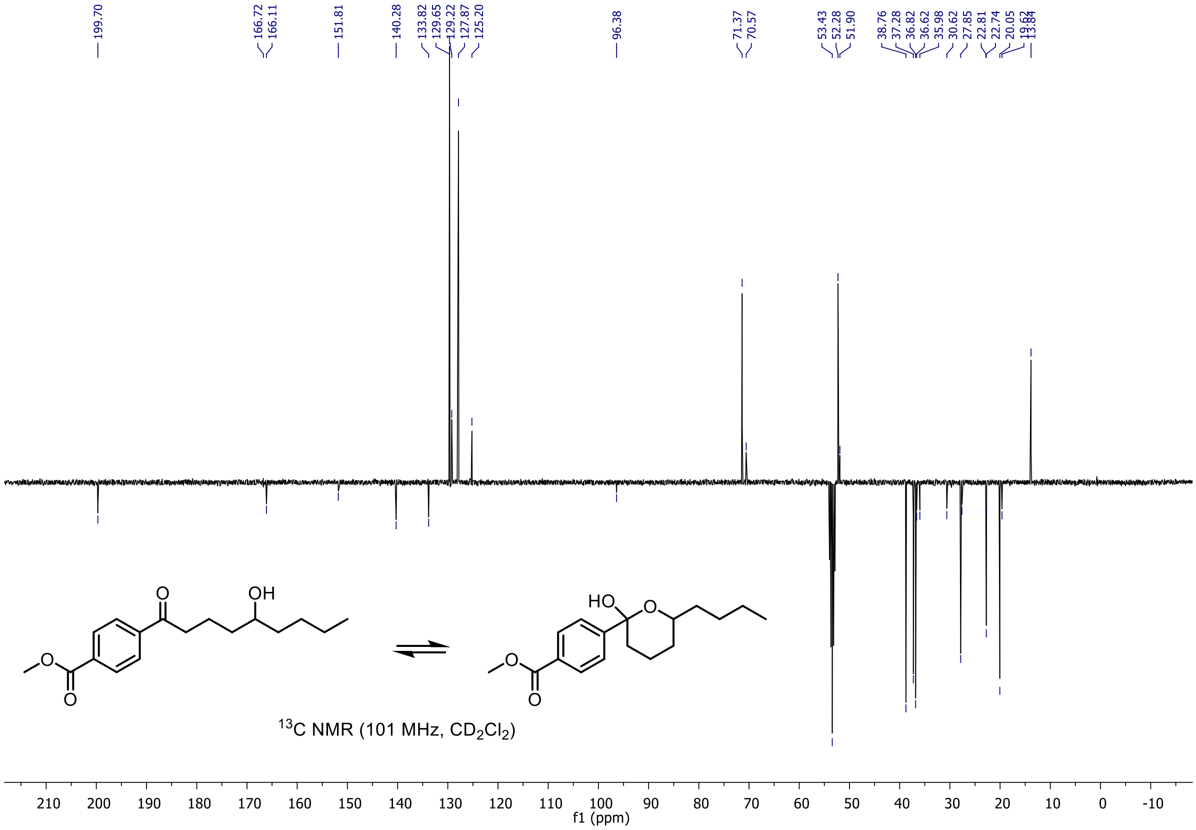

1-(3-Fluorophenyl)-5-hydroxynonan-1-one (1o)

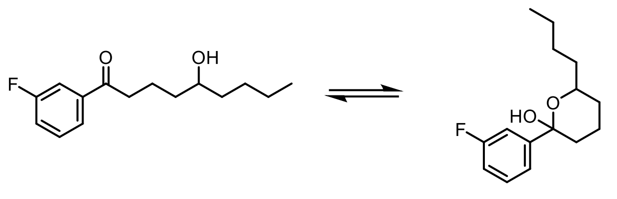

8.5 : 1

The title compound was prepared following general procedure F using 1-(3-fluorophenyl)non-8-en-1-one (23.4 mg, 0.10 mmol, 1.00 equiv.) and TfOH (27 µL, 0.30 mmol, 3.00 equiv.). Purification by flash column chromatography (0 – 100% EtOAc in heptanes) afforded the title compound (17.6 mg, 70 µmol, 70%) as a colorless liquid and a mixture of regioisomers (δ:γ = 95:5). The δ-hydroxy ketone is in equilibrium with its hemiacetal form (ratio = 8.5:1).

Crude product: NMR yield could not be determined due to signal overlap, r.r. ($\delta_{OH+Hemiacetal}$:γ_{OH}) = 93:7

An asterisk (*) denotes signals that unambiguously arise from the hemiacetal. A full list of signals arising from the minor γ-regioisomer can be found in Section 2.5.

Due to low abundance, only signals arising from the major component (δ-hydroxy ketone) are reported in ^{13}C NMR.

^{1}H NMR (400 MHz, $(CD_3)_2CO$): δ 7.89 – 7.82 (m, 0.9H), 7.69 (m, 0.9H), 7.57 (m, 0.9H), 7.39 (m, 1H), 7.34 (m, 0.1H*), 7.32 – 7.27 (m, 0.1H*), 7.04 – 6.97 (m, 0.1H*), 4.92 (d, *J* = 2.4 Hz, 0.1H*), 4.11 – 4.02 (m, 0.1H*), 3.62 – 3.54 (m, 0.9H), 3.39 (d, *J* = 5.4 Hz, 0.9H), 3.07 (m, 1.8H), 1.92 – 1.83 (m, 1H), 1.78 (m, 1H), 1.66 (m, 0.2H*), 1.58 – 1.41 (m, 5H), 1.32 (m, 3H), 0.89 (t, *J* = 7.2 Hz, 3H).

^{13}C NMR (101 MHz, $(CD_3)_2CO$) δ 199.4, 163.7 (d, *J* = 245.4 Hz), 140.5 (d, *J* = 5.8 Hz), 131.6 (d, *J* = 7.6 Hz), 124.9 (d, *J* = 2.9 Hz), 120.4 (d, *J* = 21.4 Hz), 115.1 (d, *J* = 22.1 Hz), 71.2, 39.2, 38.3, 37.8, 28.7, 23.5, 21.3, 14.4.

^{19}F NMR (659 MHz, $(CD_3)_2CO$): δ -114.00, -115.57*.

IR (neat) ν_{max}: 3455, 2932, 2359, 1737, 1589, 1442, 1366, 1229, 1216, 734.

HRMS (ESI^{+}): exact mass calculated for $[M+Na]^{+}$ $(C_{15}H_{21}FO_{2}Na)^{+}$ requires *m/z* 275.1418, found *m/z* 275.1417.

1-(3-Fluorophenyl)-5-hydroxynonan-1-one (1o)

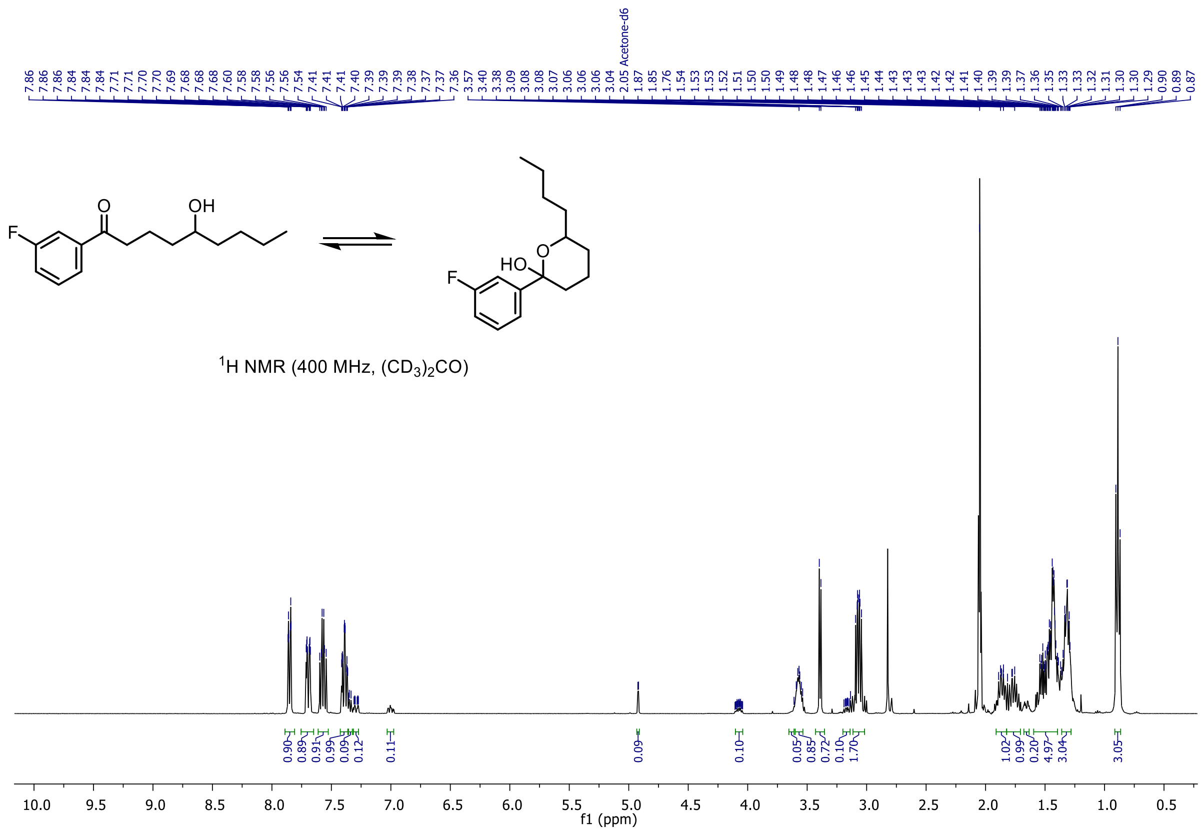

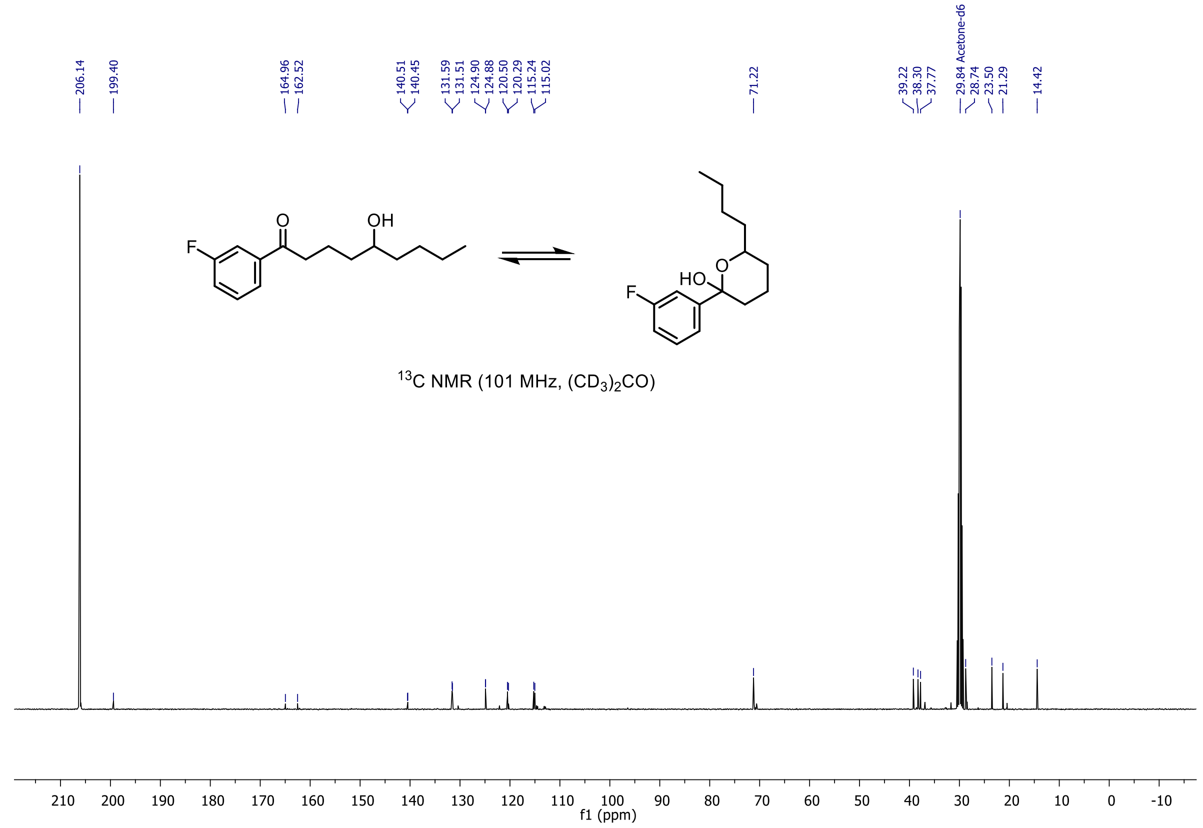

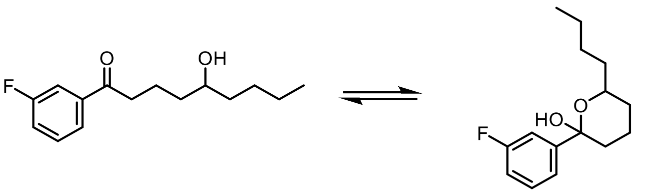

^{19}F NMR (659 MHz, $(CD_3)_2CO$)

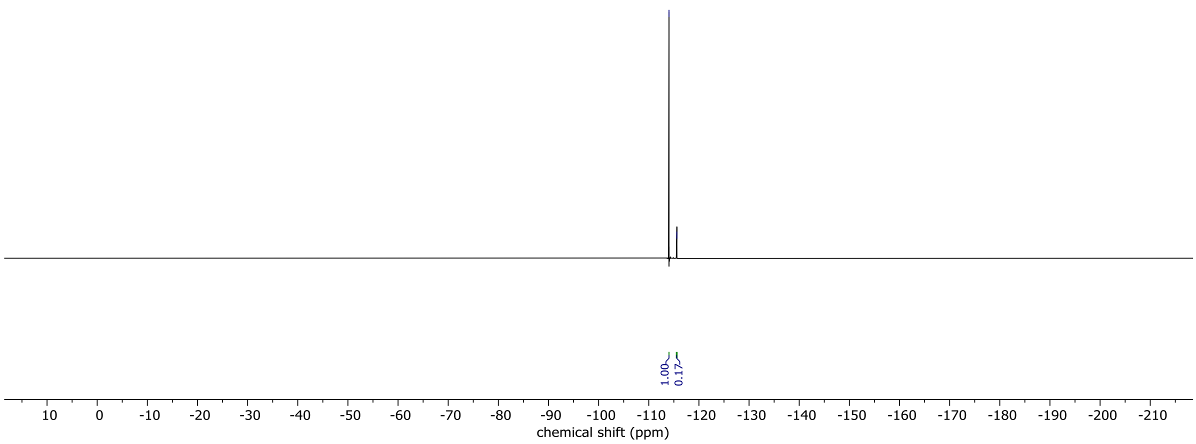

5-Hydroxy-1-(2-hydroxyphenyl)nonan-1-one (1p)

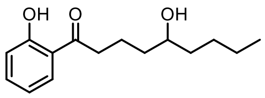

The title compound was prepared following general procedure F, with a modification to the reaction temperature (45 °C instead of 65 °C) using 1-(2-hydroxyphenyl)non-8-en-1-one (23.2 mg, 0.10 mmol, 1.00 equiv.) and TfOH (27 µL, 0.30 mmol, 3.00 equiv.). Purification by flash column chromatography (0 – 100% EtOAc in heptanes) afforded the title compound (19 mg, 76 µmol, 76%) as a colorless liquid and a mixture of regioisomers (δ:γ = 95:5).

Crude product: 86% NMR yield, r.r. (δ_{OH}:γ_{OH}) = 92:8.

A full list of signals arising from the minor γ-regioisomer can be found in Section 2.5.

^{1}H NMR (400 MHz, $(CD_3)_2CO$): δ 12.40 (s, 1H), 8.01 – 7.95 (m, 1H), 7.57 – 7.49 (m, 1H), 6.99 – 6.91 (m, 2H), , 3.63 – 3.54 (m, 1H), 3.42 (d, *J* = 5.4 Hz, 1H), 3.21 – 3.05 (m, 2H), 1.98 – 1.85 (m, 1H), 1.85 – 1.72 (m, 1H), 1.58 – 1.39 (m, 5H), 1.35 – 1.26 (m, 3H), 0.89 (t, *J* = 7.2 Hz, 3H).

^{13}C NMR (101 MHz, $(CD_3)_2CO$): δ 208.5, 163.3, 137.1, 131.5, 120.3, 119.8, 118.8, 71.2, 38.9, 38.3, 37.7, 28.7, 23.5, 21.6, 14.4.

IR (neat) ν_{max}: 3393, 2955, 2930, 2859, 2359, 2342, 1712, 1638, 1581, 1440, 1359, 1281, 1156, 726, 530.

HRMS (ESI$^+$): exact mass calculated for $[M+Na]^+$ $(C_{15}H_{22}O_3Na)^+$ requires *m/z* 273.1461, found *m/z* 273.1453.

5-Hydroxy-1-(2-hydroxyphenyl)nonan-1-one (1p)

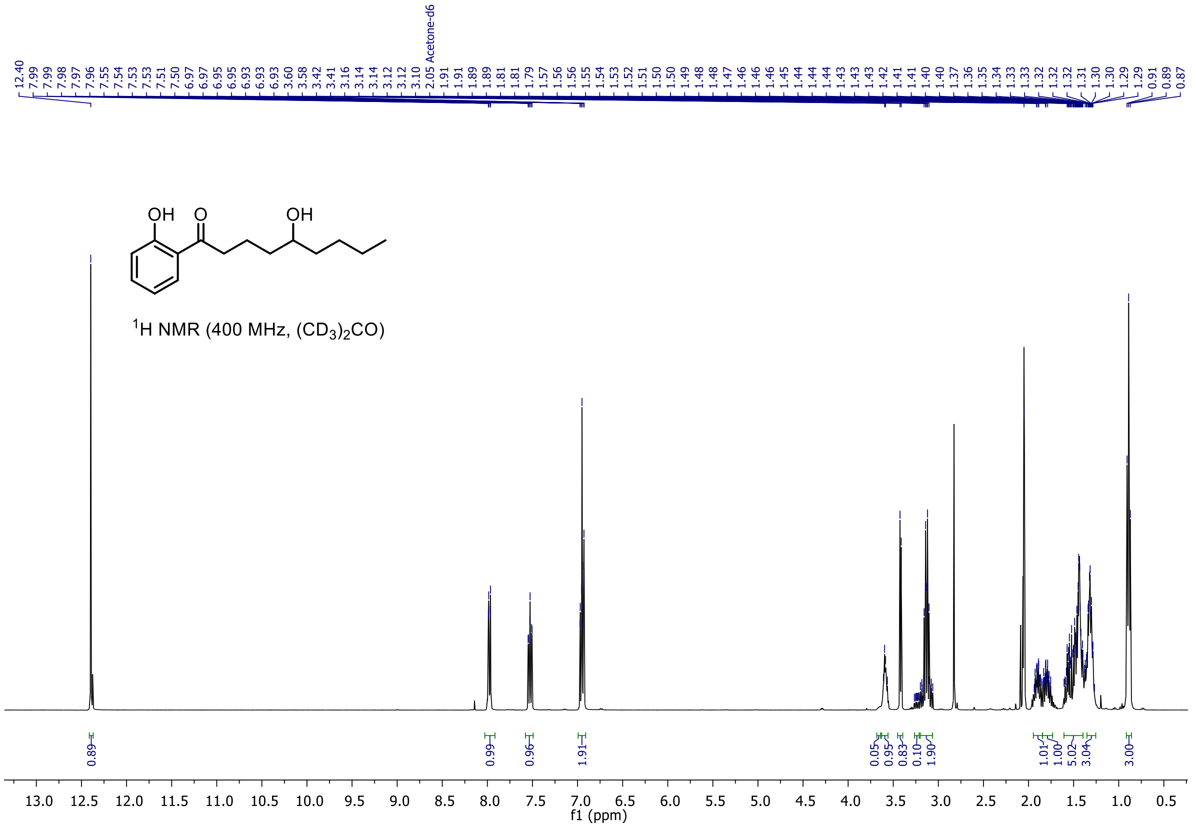

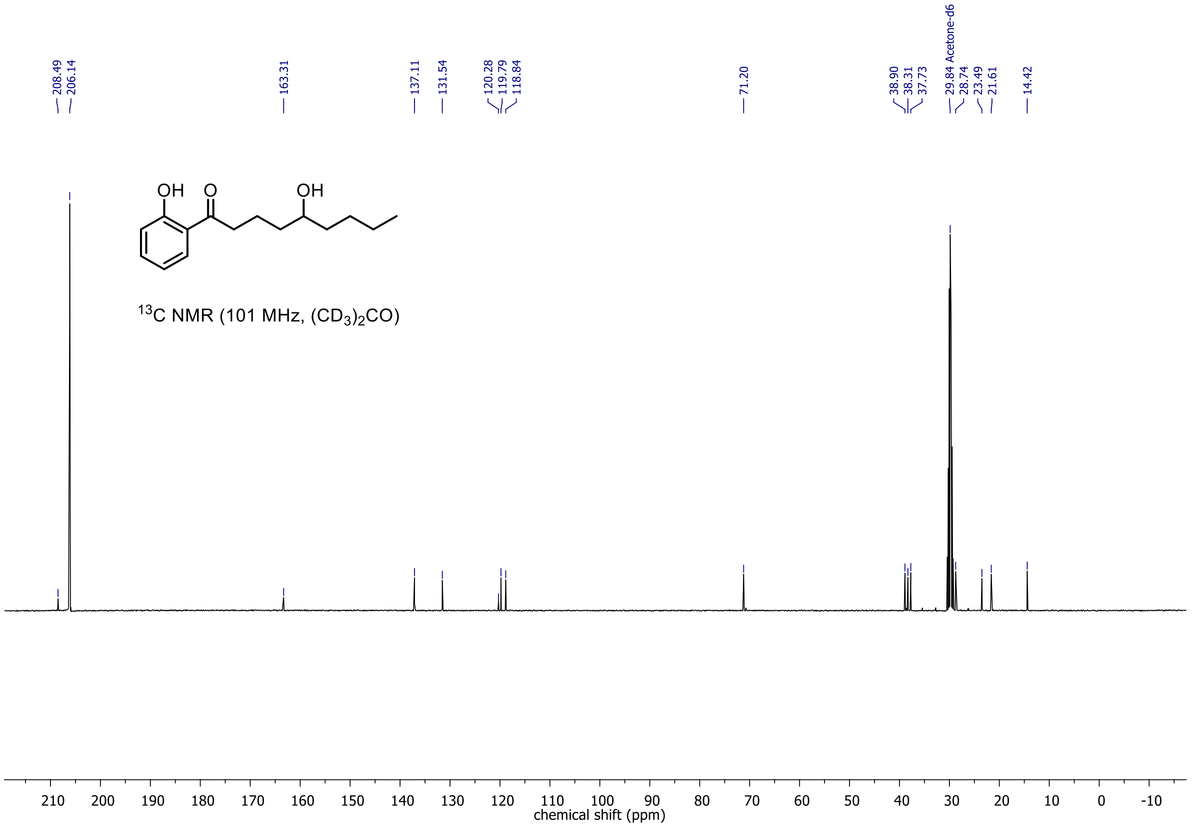

1-(4-(Dimethylamino)phenyl)-5-hydroxynonan-1-one (1q)

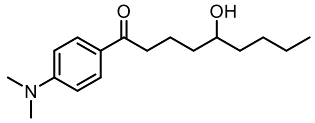

The title compound was prepared following general procedure F with a modification to the reaction temperature and time (45 °C instead of 65 °C for 2 h) using 1-(4-(dimethylamino)phenyl)non-8-en-1-one (25.9 mg, 0.10 mmol, 1.00 equiv.) and TfOH (35 µL, 0.40 mmol, 4.00 equiv.). Purification by flash column chromatography (15 – 40% EtOAc in heptanes with 1% triethylamine additive) afforded the title compound (16.8 mg, 61 µmol, 61%) as a colorless solid and a single regioisomer.

Crude product: 80% NMR yield; r.r. (δ_{OH}:γ_{OH}) = 87 : 13

^{1}H NMR (400 MHz, CD_2Cl_2) δ 7.91 – 7.80 (m, 2H), 6.70 – 6.64 (m, 2H), 3.59 – 3.51 (m, 1H), 3.04 (s, 6H), 2.89 (t, *J* = 7.2 Hz, 2H), 1.92 – 1.72 (m, 3H), 1.57 – 1.24 (m, 8H), 0.90 (t, *J* = 7.1 Hz, 3H).

^{13}C NMR (101 MHz, CD_2Cl_2) δ 198.7 (C), 153.8 (C), 130.4 (2CH), 125.3 (C), 111.0 (2CH), 71.6 (CH), 40.2 (2CH_3), 37.9 (CH_2), 37.6 (2CH_2), 28.3 (CH_2), 23.2 (CH_2), 20.9 (CH_2), 14.3 (CH_3).

IR (neat) ν_{max}: 3422, 2927, 2858, 1655, 1593, 1551, 1368, 1186, 815.

HRMS (ESI$^+$): exact mass calculated for $[M+Na]^+$ $(C_{17}H_{27}NO_2Na)^+$ requires *m/z* 300.1934, found *m/z* 300.1926.

1-(4-(Dimethylamino)phenyl)-5-hydroxynonan-1-one (1q)

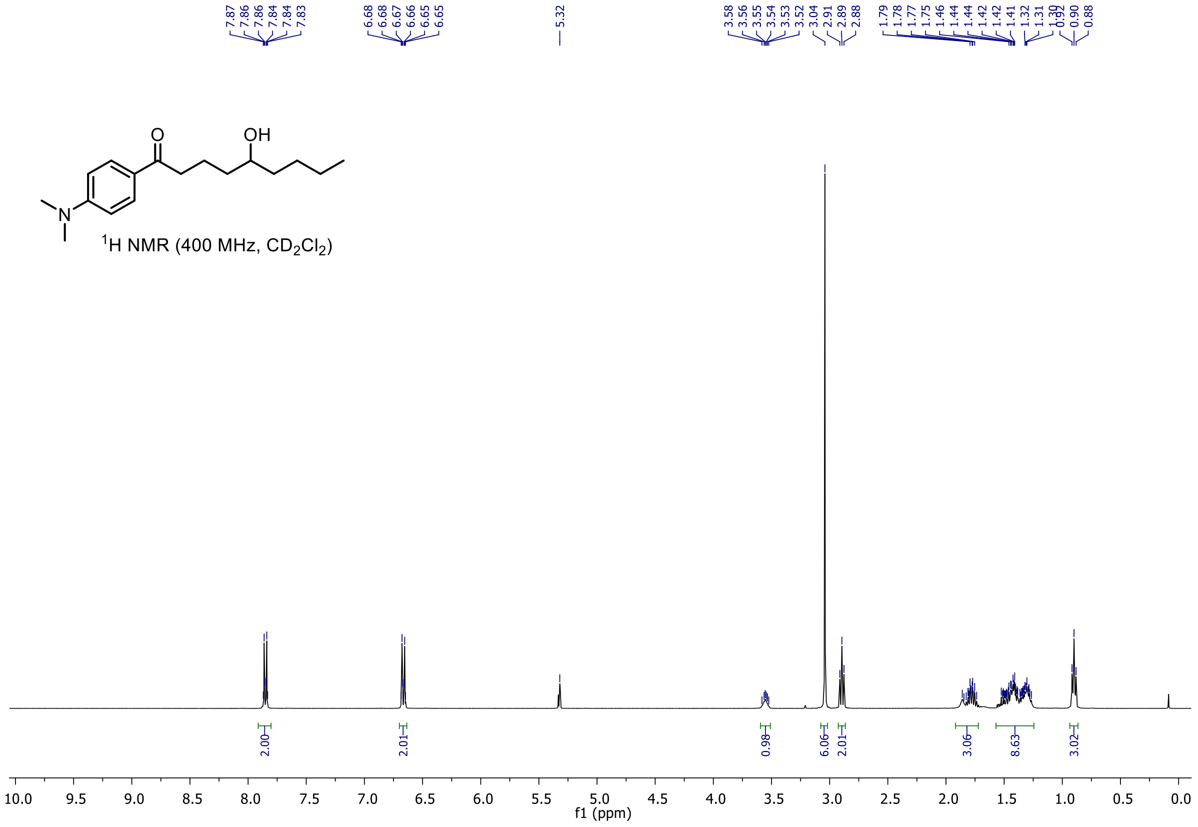

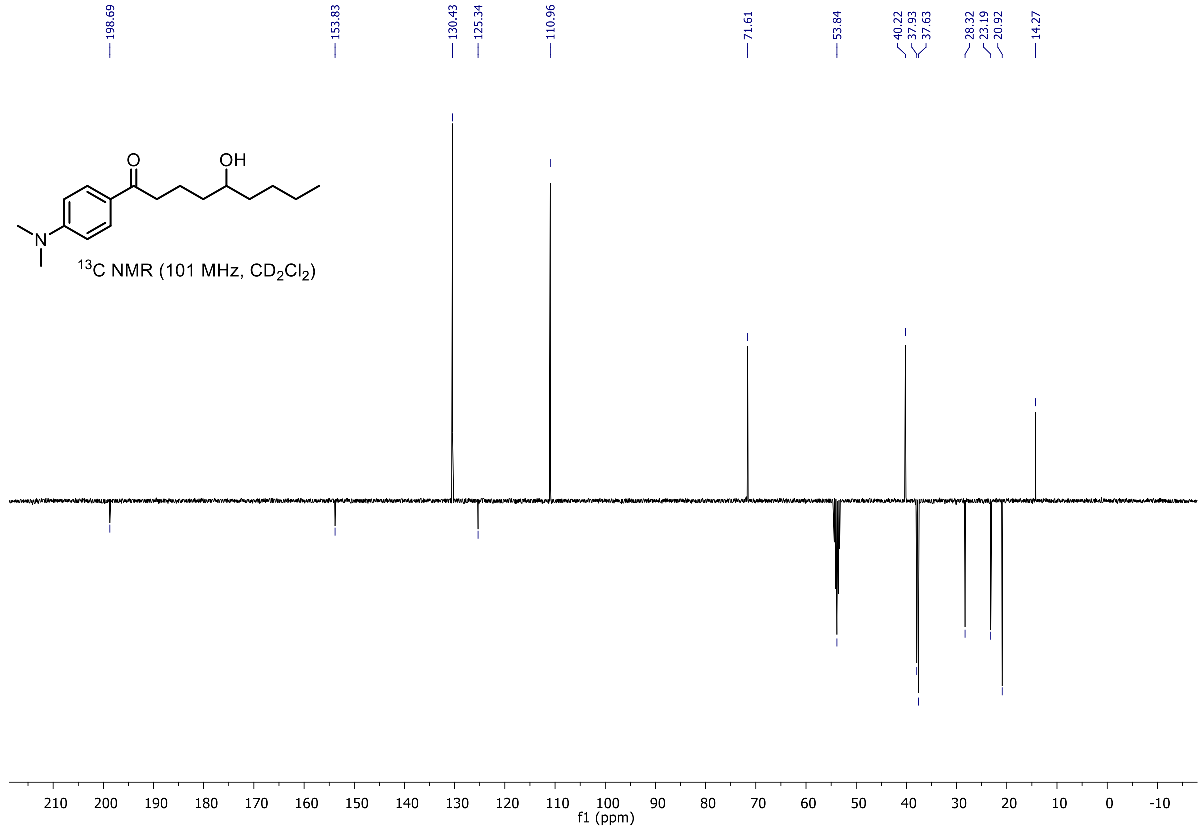

5-Hydroxy-1-(4-methoxyphenyl)decan-1-one (1r)

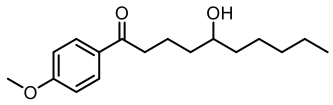

The title compound was prepared following general procedure F using 1-(4-methoxyphenyl)dec-9-en-1-one (26.0 mg, 0.10 mmol, 1.00 equiv.) and TfOH (27 µL, 0.30 mmol, 3.00 equiv.). Purification by flash column chromatography (10 – 40% EtOAc in heptanes) afforded the title compound (15.5 mg, 56 µmol, 56%) as a colorless oil and a single regioisomer.

Crude product: 67% NMR yield, r.r. (δ_{OH}:γ_{OH}) = 90 : 10

^{1}H NMR (400 MHz, CD_2Cl_2) δ 7.96 – 7.91 (m, 2H), 6.97 – 6.92 (m, 2H), 3.86 (s, 3H), 3.57 (app br s, 1H), 2.95 (t, *J* = 7.2 Hz, 2H), 1.89 – 1.72 (m, 2H), 1.69 (br s, 1H), 1.57 – 1.38 (m, 5H), 1.35 – 1.23 (m, 5H), 0.89 (t, *J* = 6.8 Hz, 3H).

^{13}C NMR (101 MHz, CD_2Cl_2) δ 199.2 (C), 163.8 (C), 130.6 (2CH), 114.0 (2CH), 71.7 (CH), 55.9 (CH_3), 53.8 (CH_2), 38.4 (CH_2), 37.9 (CH_2), 37.4 (CH_2), 32.3 (CH_2), 25.8 (CH_2), 23.1 (CH_2), 20.7 (CH_2), 14.2 (CH_3).

IR (neat) ν_{max}: 3448, 2952, 2929, 2858, 1673, 1599, 1510, 1255, 1169, 1030.

HRMS (ESI^{+}): exact mass calculated for $[M+Na]^{+}$ $(C_{17}H_{26}O_3Na)^{+}$ requires *m*/*z* 301.1774, found *m*/*z* 301.1778.

5-Hydroxy-1-(4-methoxyphenyl)decan-1-one (1r)

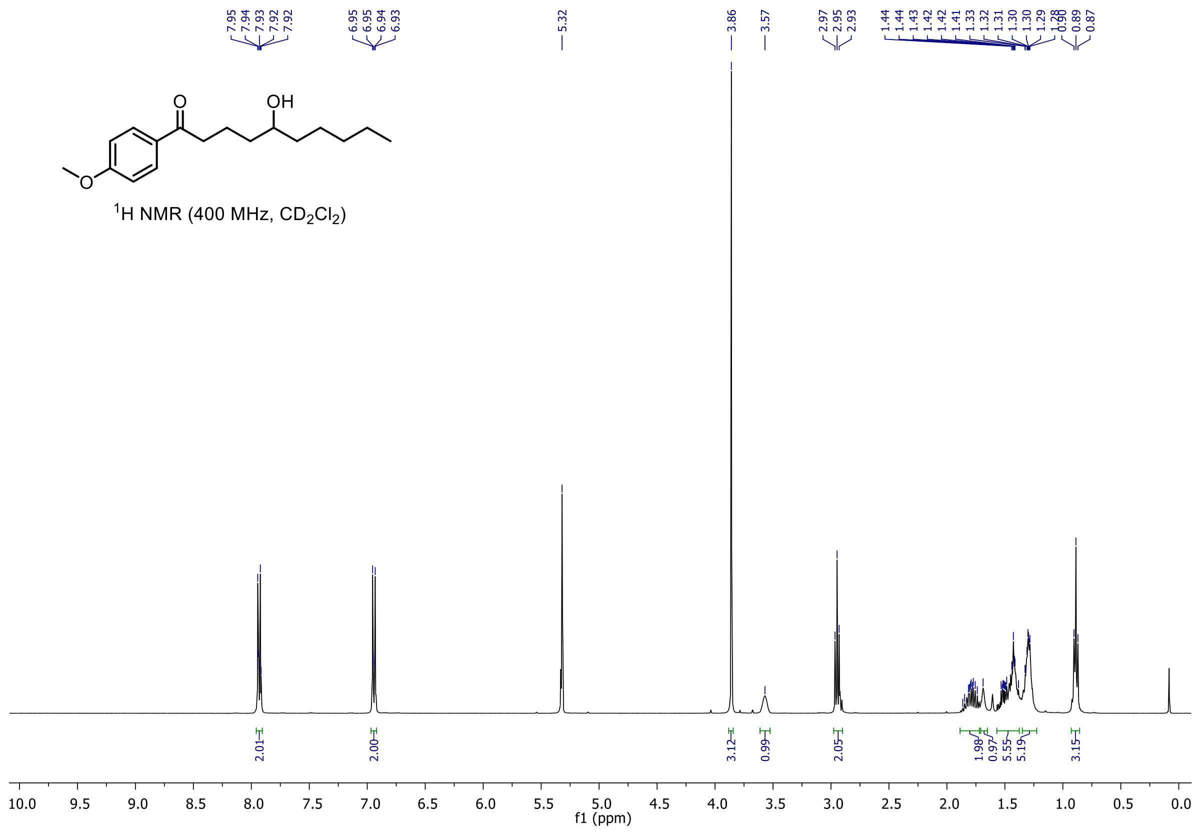

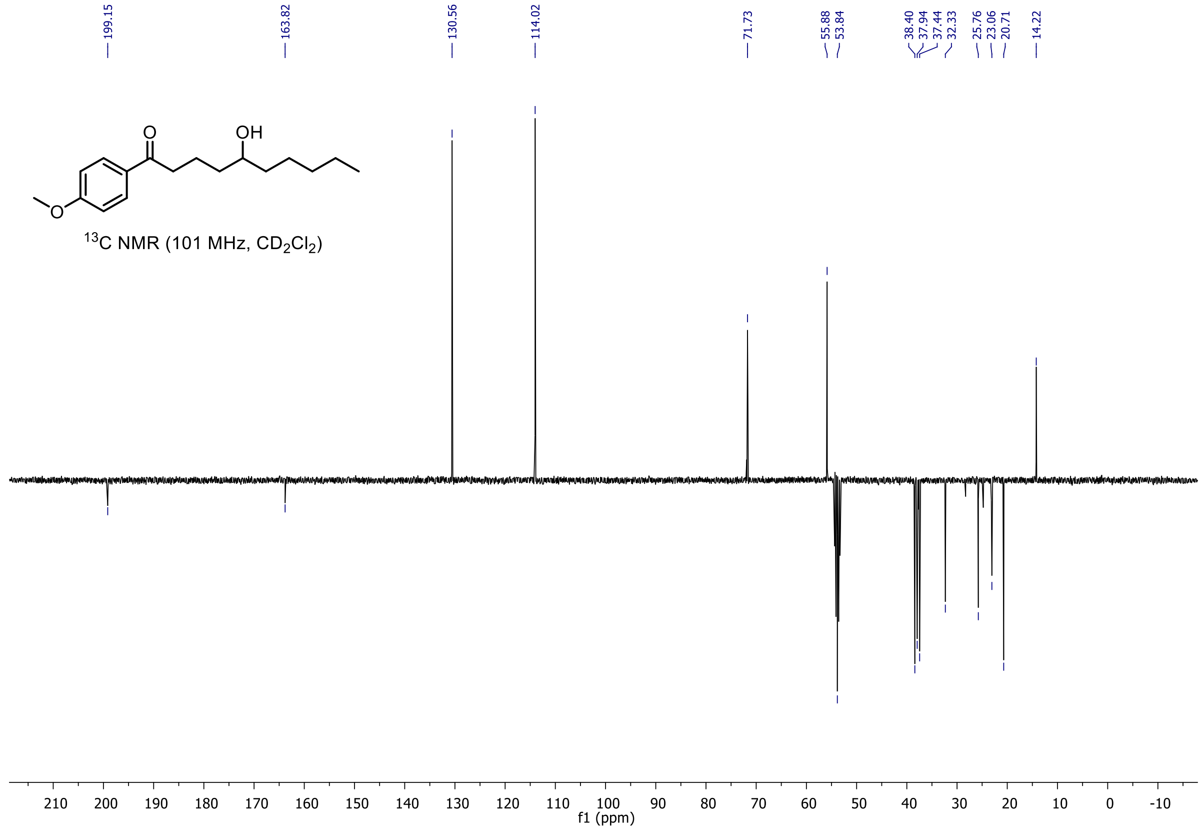

5-Hydroxy-1-(thiophen-2-yl)nonan-1-one (1s)

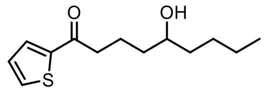

The title compound was prepared following general procedure F using 1-(thiophen-2-yl)non-8-en-1-one (22.2 mg, 0.10 mmol, 1.00 equiv.) and TfOH (27 µL, 0.30 mmol, 3.00 equiv.). Purification by flash column chromatography (0 – 70% EtOAc in heptanes) afforded the title compound (20.3 mg, 85 µmol, 85%) as a colorless liquid and a mixture of regioisomers (δ:γ = 91:9).

Crude product: 95% NMR yield, r.r. (δ_{OH}: γ_{OH}) = 88:12.

A full list of signals arising from the minor γ-regioisomer can be found in Section 2.5.

^{1}H NMR (400 MHz, CD_2Cl_2): δ 7.78 – 7.71 (m, 1H), 7.67 – 7.63 (m, 1H), 7.14 (dd, *J* = 5.0, 3.8 Hz, 1H), 3.68 – 3.50 (m, 1H), 2.97 – 2.89 (m, 2H), 1.93 – 1.73 (m, 2H), 1.67 (s, 1H), 1.58 – 1.25 (m, 8H), 0.96 – 0.84 (m, 3H).

^{13}C NMR (101 MHz, CD_2Cl_2): δ 193.8, 145.1, 133.9, 132.4, 128.7, 71.9, 39.6, 37.8, 37.4, 28.4, 23.3, 21.1, 14.4.

IR (neat) ν_{max}: 3415, 2958, 2928, 2859, 1654, 1413, 1236, 1058, 855, 719, 589.

HRMS (ESI$^+$): exact mass calculated for $[M+Na]^+$ $(C_{13}H_{20}O_2SNa)^+$ requires *m/z* 263.1076, found *m/z* 263.1070.

5-Hydroxy-1-(thiophen-2-yl)nonan-1-one (1s)

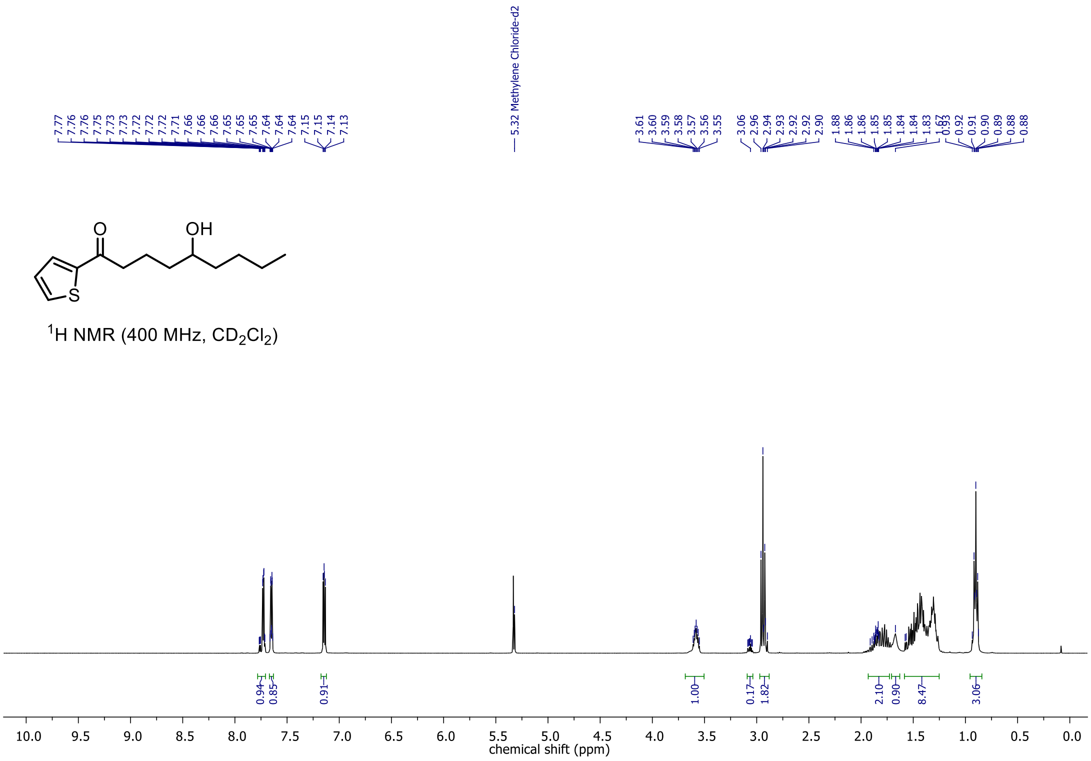

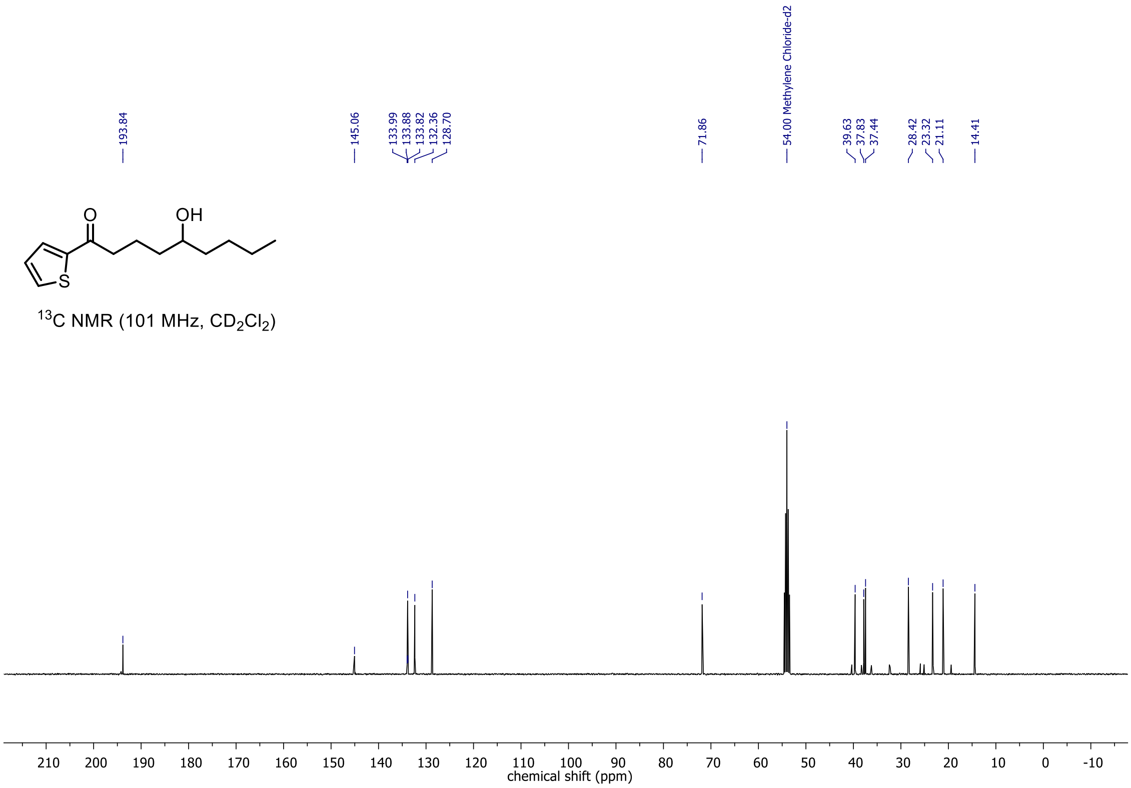

1-(Furan-2-yl)-5-hydroxynonan-1-one (1t)

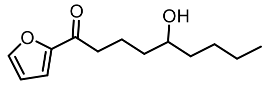

The title compound was prepared following general procedure F using 1-(furan-2-yl)non-8-en-1-one (20.6 mg, 0.10 mmol, 1.00 equiv.) and TfOH (27 µL, 0.30 mmol, 3.00 equiv.). Purification by flash column chromatography (20 – 50% EtOAc in heptanes) afforded the title compound (16.6 mg, 74 µmol, 74%) as a colorless solid and a single regioisomer.

Crude product: 78% NMR yield, r.r. (δ_{OH}:γ_{OH}) = 91 : 9

^{1}H NMR (400 MHz, CD_2Cl_2) δ 7.66 – 7.53 (m, 1H), 7.18 (dd, *J* = 3.5, 0.4 Hz, 1H), 6.54 (dd, *J* = 3.5, 1.7 Hz, 1H), 3.57 (tt, *J* = 8.5, 4.4 Hz, 1H), 2.84 (t, *J* = 7.3 Hz, 2H), 1.89 – 1.67 (m, 2H), 1.64 (br s, 1H), 1.56 – 1.27 (m, 8H), 0.90 (t, *J* = 6.9 Hz, 3H).

^{13}C NMR (101 MHz, CD_2Cl_2) δ 189.7 (C), 153.2 (C), 146.7 (CH), 117.2 (CH), 112.4 (CH), 71.7 (CH), 38.6 (CH_2), 37.7 (CH_2), 37.3 (CH_2), 28.3 (CH_2), 23.2 (CH_2), 20.5 (CH_2), 14.3 (CH_3).

IR (neat) ν_{max}: 3412, 2954, 2929, 2860, 1669, 1567, 1467, 1081, 1011, 883, 761, 595.

HRMS (ESI$^+$): exact mass calculated for $[M+Na]^+$ $(C_{13}H_{20}O_3Na)^+$ requires *m*/*z* 247.1305, found *m*/*z* 247.1299.

1-(Furan-2-yl)-5-hydroxynonan-1-one (1t)

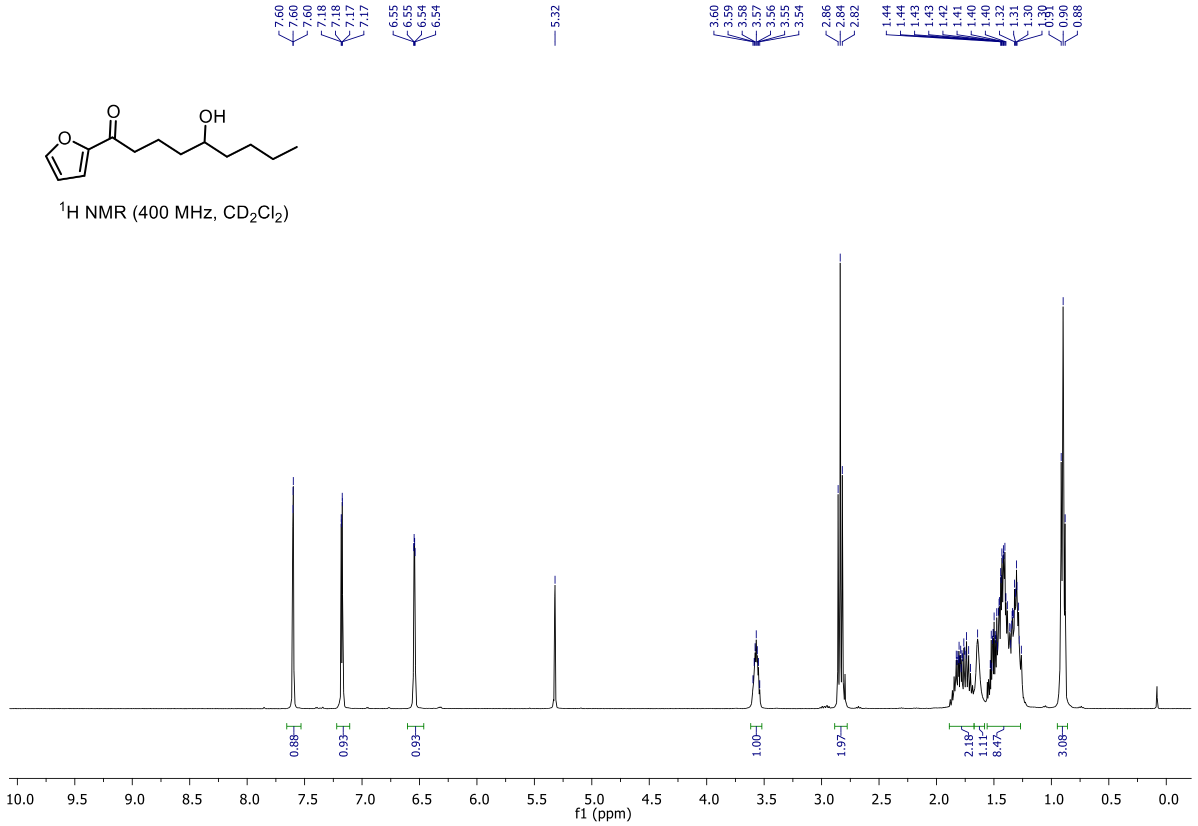

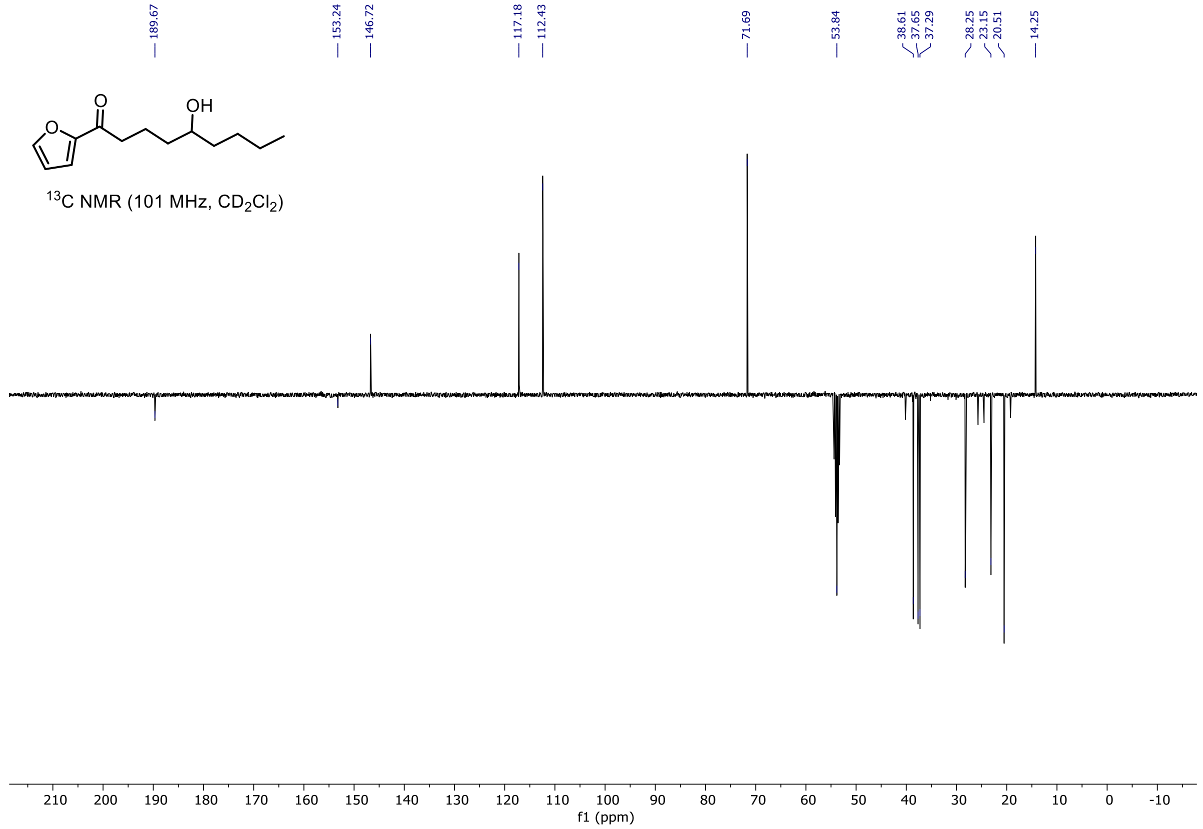

5-Hydroxy-1-ferrocene-non-8-en-1-one (1u)

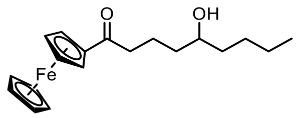

The title compound was prepared following general procedure F using 1-ferrocene-non-8-en-1-one (32.4 mg, 0.10 mmol, 1.00 equiv.) and TfOH (27 µL, 0.30 mmol, 3.00 equiv.). Purification by flash column chromatography (0 – 100% EtOAc in heptanes) afforded the title compound (20.9 mg, 61 µmol, 61%) as an orange liquid and a mixture of regioisomers (δ:ε = 86:14).

Crude product: 77% NMR Yield, r.r. (δ_{OH}: ε_{OH}:γ_{OH}) = 78:13:9.

Although the γ-isomer was not prepared independently, the minor product obtained in this reaction was determined to be the γ-isomer by analogy (chemical shifts & splitting patterns) with the other reported compounds. The r.r. value is only orientational, as it was determined from ^{13}C NMR.

^{f1}H NMR (400 MHz, $(CD_3)_2CO$): δ 4.82 – 4.78 (m, 2H), 4.53 – 4.50 (m, 2H), 4.25 – 4.20 (m, 5H), 3.61 – 3.52 (m, 1H), 3.40 (d, *J* = 5.3 Hz, 1H), 2.81 – 2.74 (m, 2H), 1.89 – 1.78 (m, 1H), 1.78 – 1.64 (m, 1H), 1.61 – 1.40 (m, 5H), 1.39 – 1.28 (m, 3H), 0.91 (t, *J* = 7.1 Hz, 3H).

^{13}C NMR (101 MHz, $(CD_3)_2CO$): δ 204.0, 80.8, 72.8 (2C), 71.5, 70.6 (5C), 70.18, 70.16, 40.3, 38.5, 38.3, 29.0, 23.7, 21.6, 14.6.

IR (neat) ν_{max}: 3431, 2929, 2859, 2360, 1712, 1658, 1454, 1411, 1378, 1251, 1106, 1057, 1002, 823, 531.

HRMS (ESI$^+$): exact mass calculated for $[M+Na]^+$ $(C_{19}H_{26}FeO_2Na)^+$ requires *m/z* 365.1175, found *m/z* 365.1172.

5-Hydroxy-1-ferrocene-non-8-en-1-one (1u)

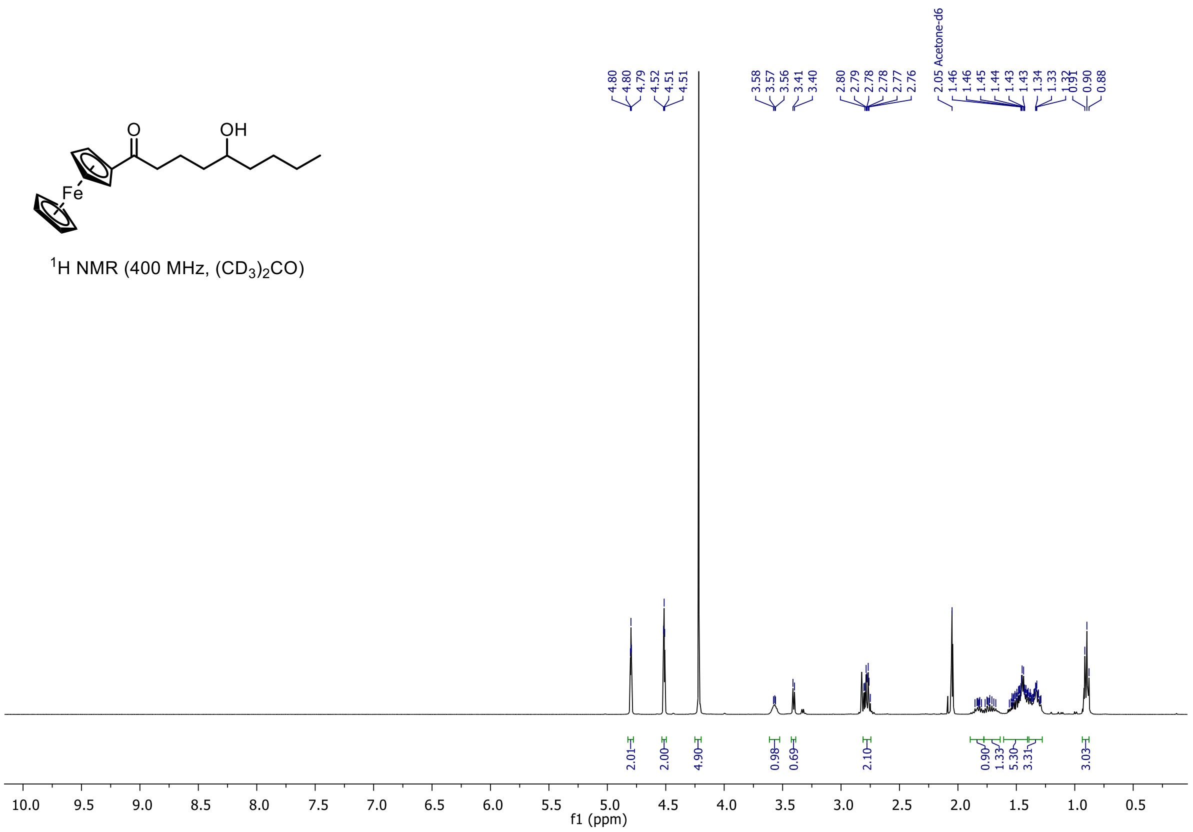

^{1}H NMR (400 MHz, $(CD_3)_2CO$)

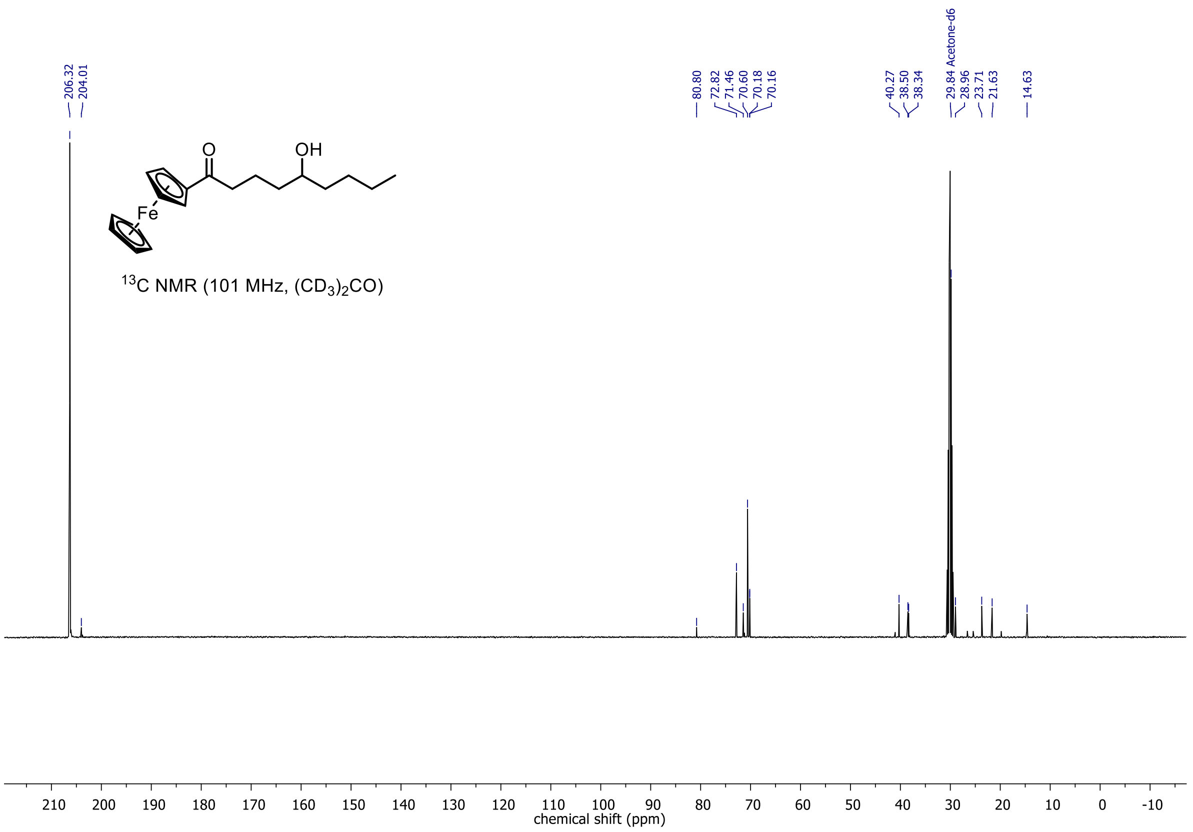

^{13}C NMR (101 MHz, $(CD_3)_2CO$)

1-(3,5-Dimethylisoxazol-4-yl)-5-hydroxynonan-1-one (1v)

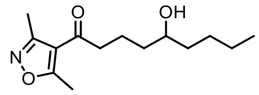

The title compound was prepared following general procedure F, with a modification to the reaction temperature and time (23 °C instead of 65 °C for 90 min), using 1-(3,5-dimethylisoxazol-4-yl)non-8-en-1-one (23.5 mg, 0.10 mmol, 1.00 equiv.) and TfOH (27 µL, 0.30 mmol, 3.00 equiv.). Purification by flash column chromatography (0 – 100% EtOAc in heptanes) afforded the title compound (18.0 mg, 71 µmol, 71%) as a colorless oil and single regioisomer.

Crude product: 73% NMR yield, r.r. (δ_{OH} : γ_{OH}) > 95:5.

^{1}H NMR (400 MHz, CDCl$_3$): δ 3.67 – 3.56 (m, 1H), 2.76 (td, *J* = 7.0, 1.3 Hz, 2H), 2.68 (s, 3H), 2.46 (s, 3H), 1.91 – 1.73 (m, 2H), 1.61 – 1.57 (m, 1H), 1.55 – 1.23 (m, 8H), 0.91 (t, *J* = 7.1 Hz 3H).

^{13}C NMR (101 MHz, CDCl$_3$): δ 195.0, 173.8, 159.3, 117.0, 71.7, 42.3, 37.4, 36.9, 28.0, 22.9, 19.6, 14.5, 14.2, 12.8.

IR (neat) ν_{max}: 3417, 2963, 2891, 1706, 1656, 1533, 1344, 1108, 1063, 1022, 955, 834.

HRMS (ESI$^+$): exact mass calculated for [M+Na]$^+$ ($C_{14}H_{23}NO_3Na$)$^+$ requires *m/z* 276.1570, found *m/z* 276.1563.

1-(3,5-Dimethylisoxazol-4-yl)-5-hydroxynonan-1-one (1v)

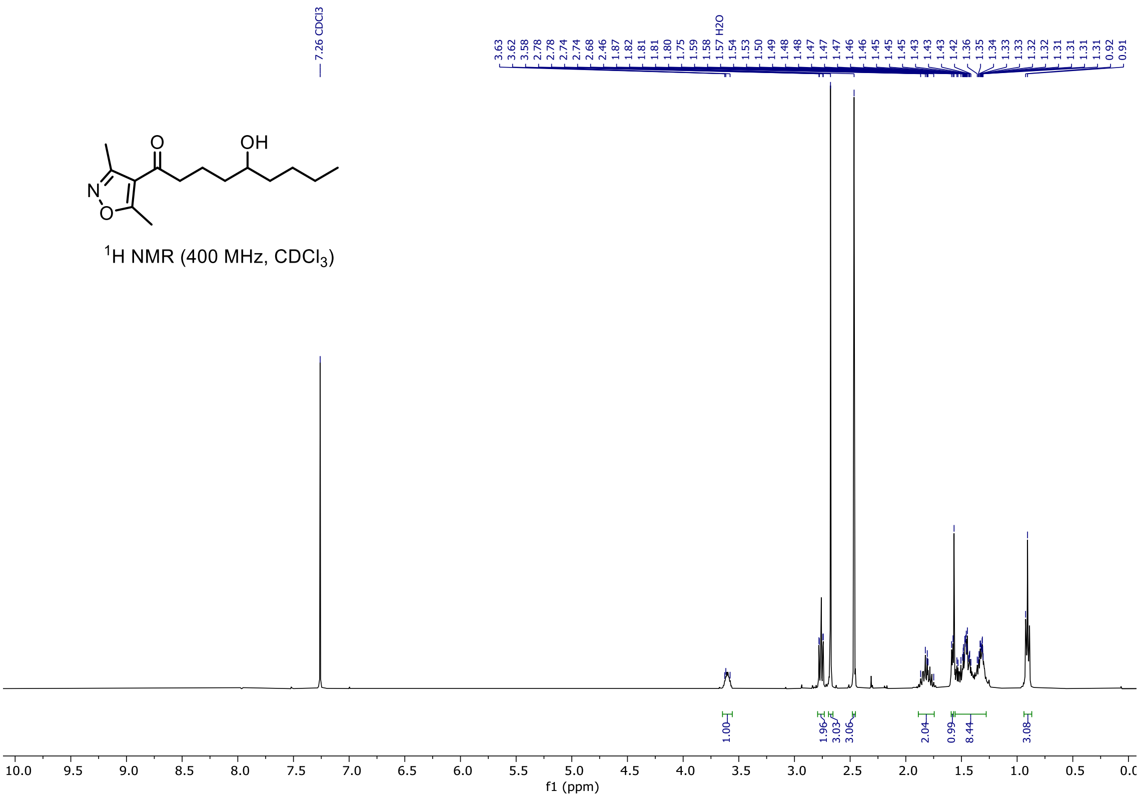

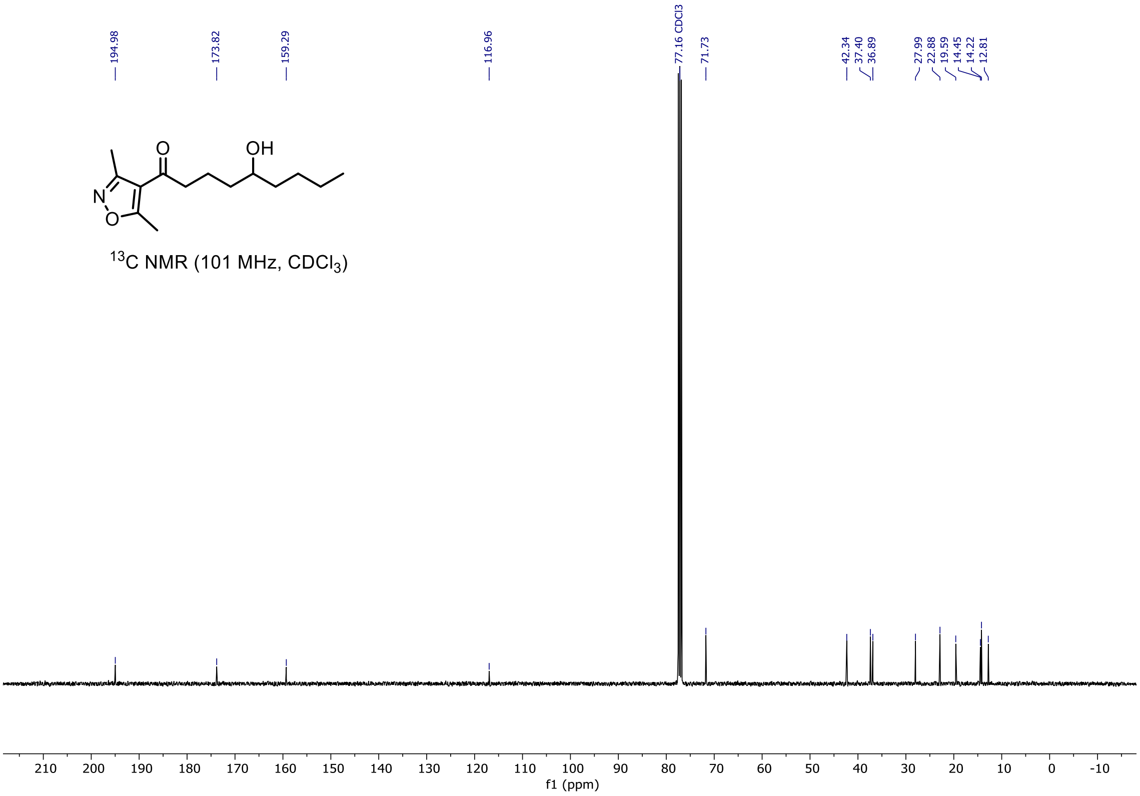

1-(3-(Difluoromethyl)-1-methyl-*1H*-pyrazol-4-yl)-5-hydroxynonan-1-one (1w)

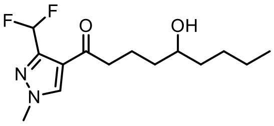

The title compound was prepared following general procedure F, with a modification to the reaction temperature and time (23 °C instead of 65 °C for 90 min), using 1-(3-(difluoromethyl)-1-methyl-1*H*-pyrazol-4-yl)non-8-en-1-one (27.0 mg, 0.10 mmol, 1.00 equiv.) and TfOH (27 µL, 0.30 mmol, 3.00 equiv.). Purification by flash column chromatography (0 – 100% EtOAc in heptanes) afforded the title compound (19.0 mg, 62 µmol, 62%) as a colorless oil and a mixture of regioisomers (δ:γ = 94:6).

Crude product: 69% NMR yield, r.r. (δ_{OH}: γ_{OH}) = 92:8.

A full list of signals arising from the minor γ-regioisomer can be found in Section 2.5.

^{1}H NMR (600 MHz, CD_2Cl_2): δ 7.89 (s, 1H), 7.13 (t, *J* = 54.1 Hz, 1H), 3.95 (s, 3H), 3.60 – 3.53 (m, 1H), 2.95 –2.77 (t, *J* = 7.2 Hz, 2H), 1.84 – 1.70 (m, 2H), 1.54 – 1.46 (m, 1H), 1.46 – 1.24 (m, 7H), 0.90 (t, *J* = 7.1 Hz, 3H).

^{13}C NMR (151 MHz, CD_2Cl_2): δ 194.7, 146.1 (t, *J* = 24.0 Hz), 121.8, 121.7 (t, *J* = 3.3 Hz), 110.4 (t, *J* = 236.0 Hz), 71.9, 41.1, 40.2, 37.9, 37.4, 28.4, 23.3, 20.7, 14.4.

^{19}F NMR (376 MHz, CD_2Cl_2): δ -116.28.

IR (neat) ν_{max}: 3377, 3016, 2971, 2913, 1707, 1680, 1300, 1181, 1097, 1073, 888.

HRMS (ESI$^+$): exact mass calculated for $[M+H]^+$ $(C_{14}H_{23}F_2N_2O_2)^+$ requires *m/z* 289.1722, found *m/z* 289.1714.

1-(3-(Difluoromethyl)-1-methyl-1*H*-pyrazol-4-yl)-5-hydroxynonan-1-one (1w)

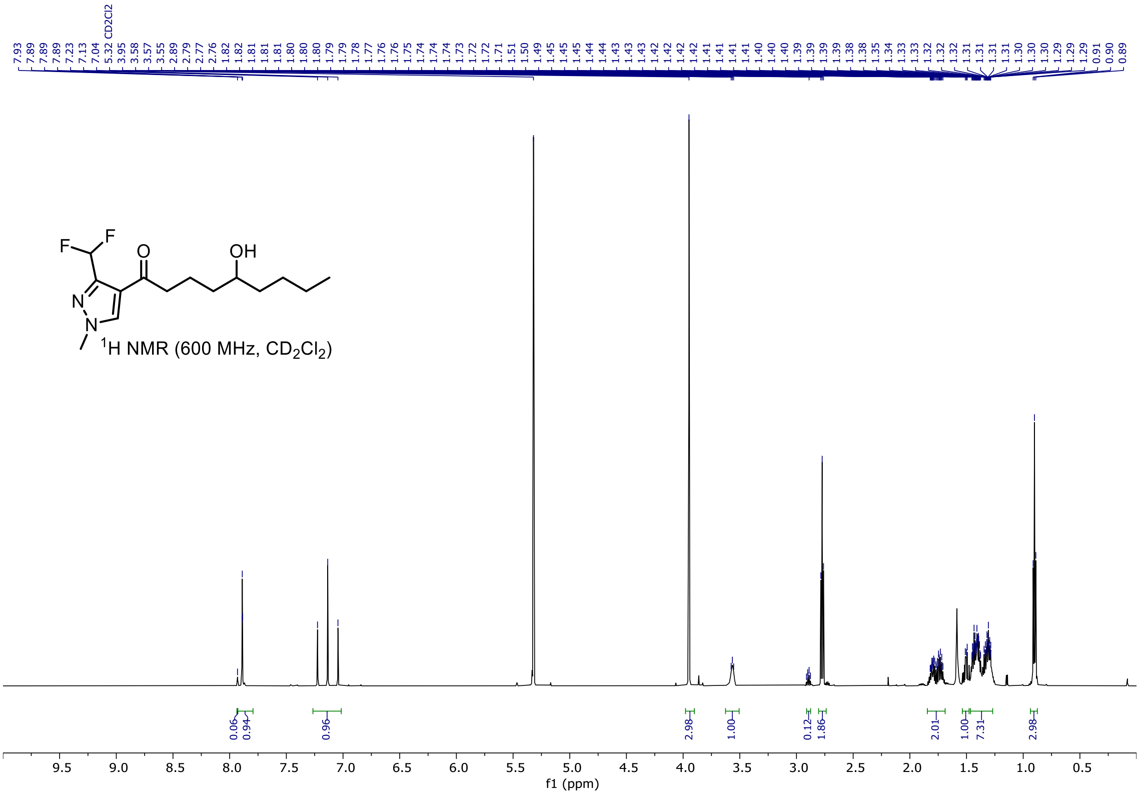

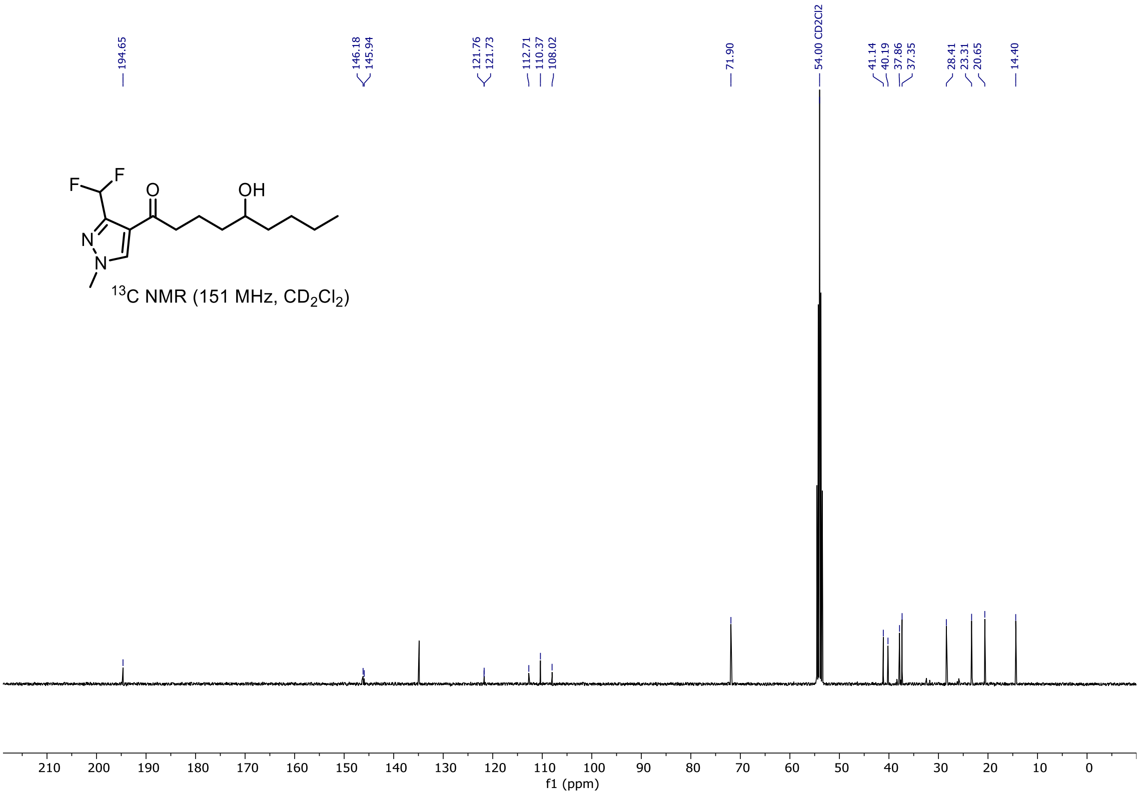

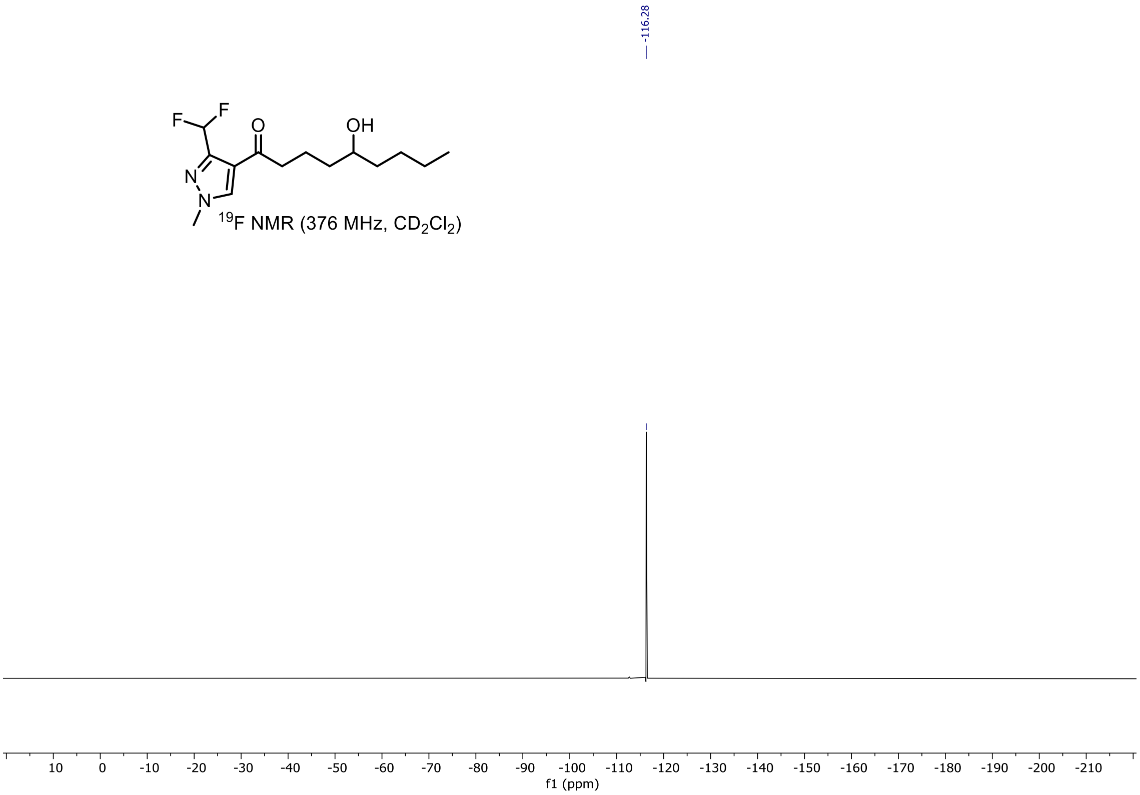

^{19}F NMR (376 MHz, CD_2Cl_2)

6-Hydroxydecan-2-one (1x)

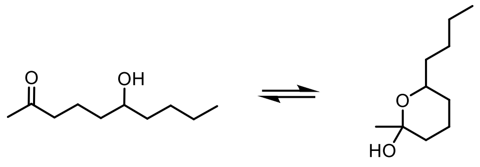

3.3 : 1

The title compound was prepared following general procedure F using dec-9-en-2-one (30.9 mg, 0.20 mmol, 1.00 equiv.) and TfOH (53 µL, 0.60 mmol, 3.00 equiv.). Purification by flash column chromatography (0–40% EtOAc in heptanes) afforded the title compound (12.9 mg, 76 µmol, 38%) as a colorless liquid and a single regioisomer. The compound is in equilibrium with its hemiacetal form (ratio = 3.3:1).

Crude product: 48% NMR yield, r.r. ($\delta_{OH+Hemiacetal}$:γ_{OH}) = 85:15.

An asterisk (*) denotes signals that unambiguously arise from the hemiacetal.

^{1}H NMR (700 MHz, CD_2Cl_2): δ 3.80 – 3.74 (m, 0.3H*), 3.55 – 3.47 (m, 0.7H), 2.44 (t, *J* = 7.2 Hz, 1.5H), 2.10 (s, 2H), 2.06 – 1.97 (m, 0.2H*), 1.82 – 1.54 (m, 3.7H), 1.45 – 1.24 (m, 8.3H), 1.14 – 1.05 (m, 0.3H), 0.92 – 0.87 (m, 3H).

^{13}C NMR (176 MHz, CD_2Cl_2): δ 209.4, 95.9*, 71.8, 70.6*, 44.0, 37.8, 37.4, 36.7*, 35.1*, 31.4*, 31.2*, 30.2, 28.4, 28.3*, 23.4*, 23.3, 20.3, 19.9*, 14.4.

IR (neat) ν_{max}: 3410, 2931, 2860, 1709, 1456, 1373, 1197, 1096, 1031, 1016, 925, 735.

HRMS (ESI^{+}): exact mass calculated for $[M+Na]^{+}$ $(C_{10}H_{20}O_2Na)^{+}$ requires *m/z* 195.1356, found *m/z* 195.1359.

6-Hydroxydecan-2-one (1x)

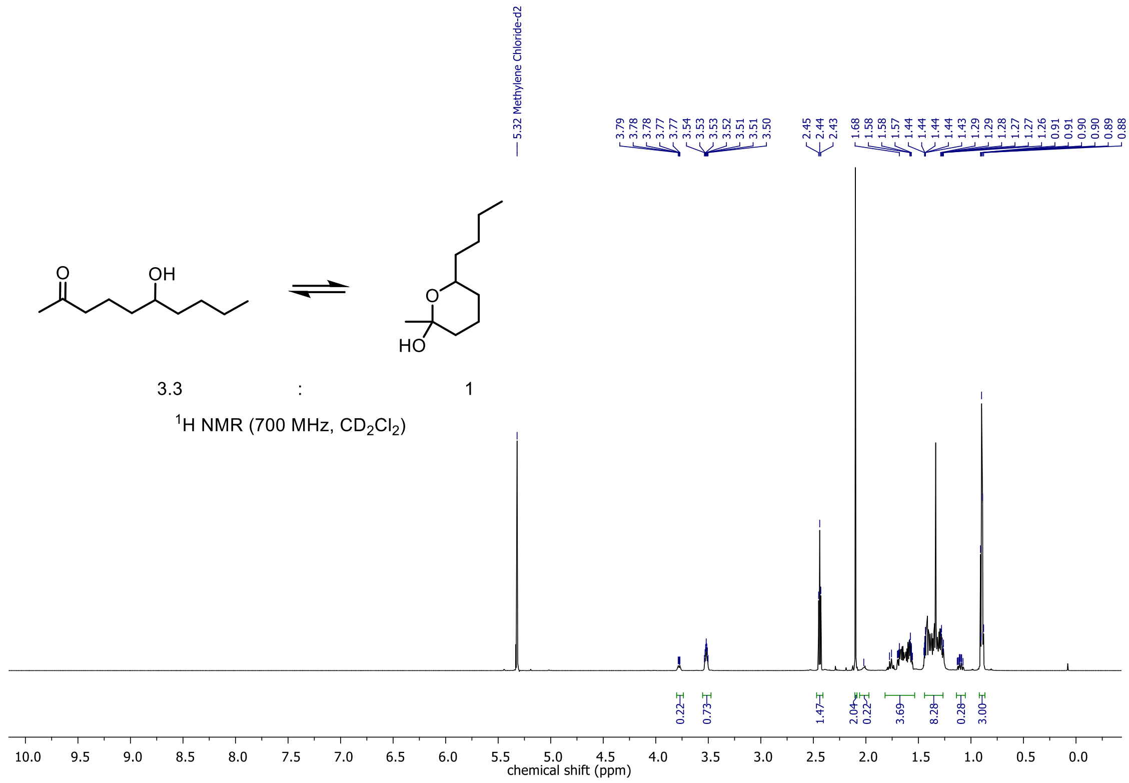

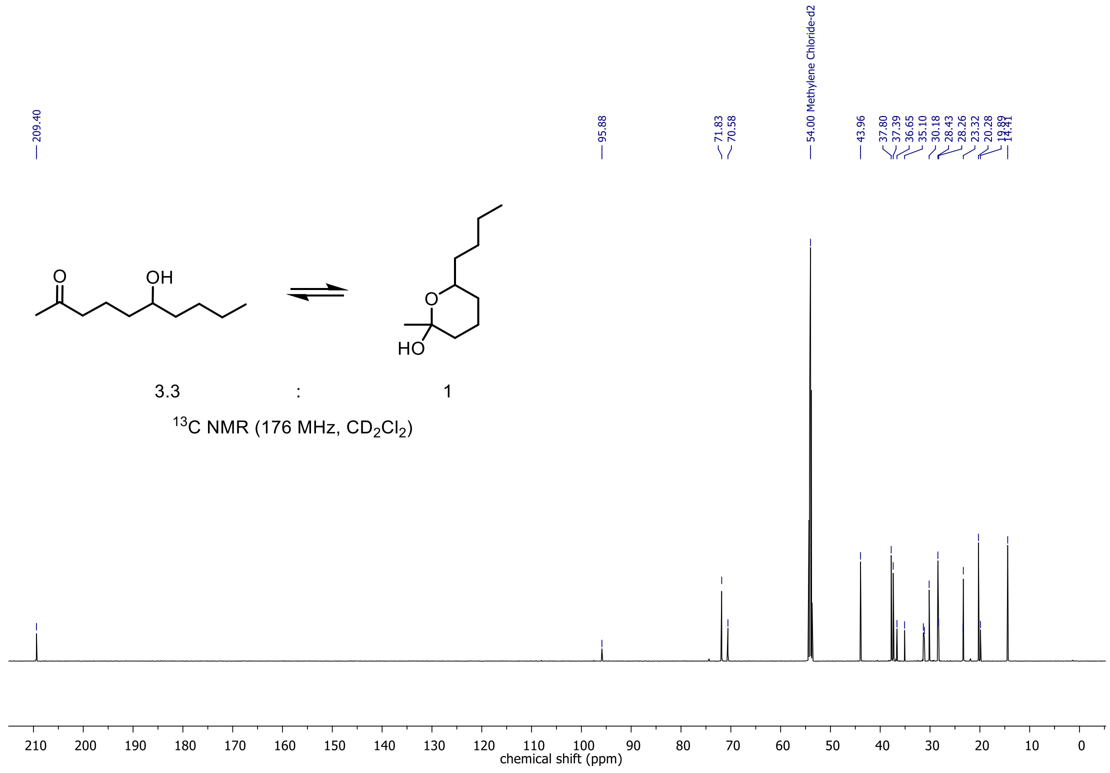

9-Hydroxytetradecan-5-one (1y)

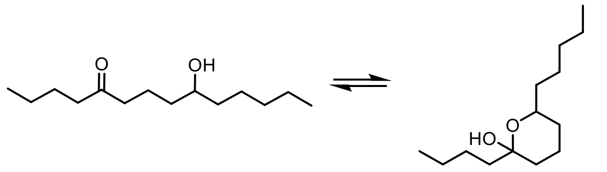

10 : 1

The title compound was prepared following general procedure F using tetradec-13-en-5-one (21.0 mg, 0.10 mmol, 1.00 equiv.) and TfOH (27 µL, 0.30 mmol, 3.00 equiv.). Purification by flash column chromatography (0 – 50% EtOAc in heptanes) afforded the title compound (14.0 mg, 61 µmol, 61%) as a colorless liquid and a mixture of regioisomers (δ:γ = 88:12). The δ-hydroxy ketone was observed to be in equilibrium with its hemiacetal form (ratio = 10:1).

Crude product: 85% NMR yield, r.r. ($\delta_{OH+Hemiacetal}$:γ_{OH}) = 87:13.

An asterisk (*) denotes signals that unambiguously arise from the hemiacetal. A full list of signals arising from the minor γ-regioisomer can be found in Section 2.5.

^{1}H NMR (400 MHz, CD_2Cl_2): δ 3.79 – 3.77 (m, 0.1H*), 3.55 – 3.44 (m, 0.9H), 2.53 (td, *J* = 7.1, 1.5 Hz, 0.2H), 2.46 – 2.32 (m, 3.8H), 1.71 – 1.49 (m, 5H), 1.44 – 1.27 (m, 12H), 0.93 – 0.86 (m, 6H).

Due to low abundance, only signals arising from the major component (δ-hydroxy ketone) are reported in ^{13}C NMR.

^{13}C NMR (101 MHz, CD_2Cl_2): δ 211.8, 71.8, 43.0, 43.0, 38.1, 37.5, 32.5, 26.5, 25.9, 23.2, 22.9, 20.3, 14.4, 14.2.

IR (neat) ν_{max}: 3411, 2955, 2928, 2859, 1709, 1458, 1377, 1128.

HRMS (ESI^{+}): exact mass calculated for $[M+Na]^{+}$ $(C_{14}H_{28}O_2Na)^{+}$ requires *m/z* 251.1982, found *m/z* 251.1972.

9-Hydroxytetradecan-5-one (1y)

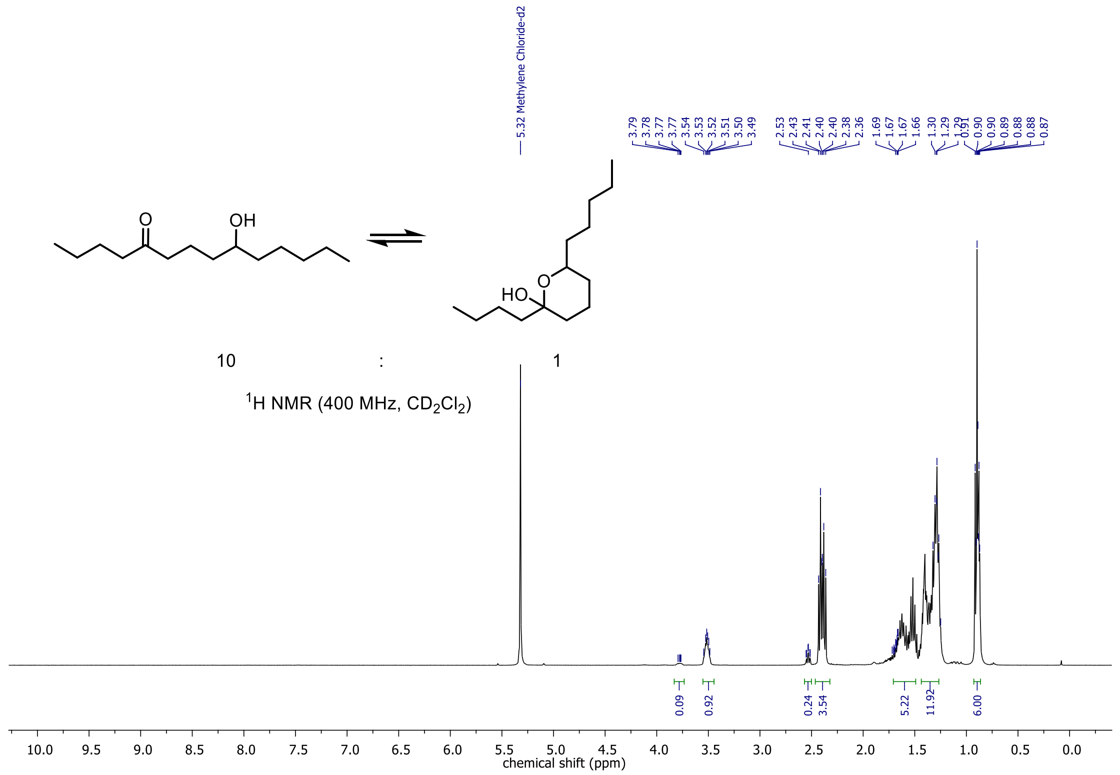

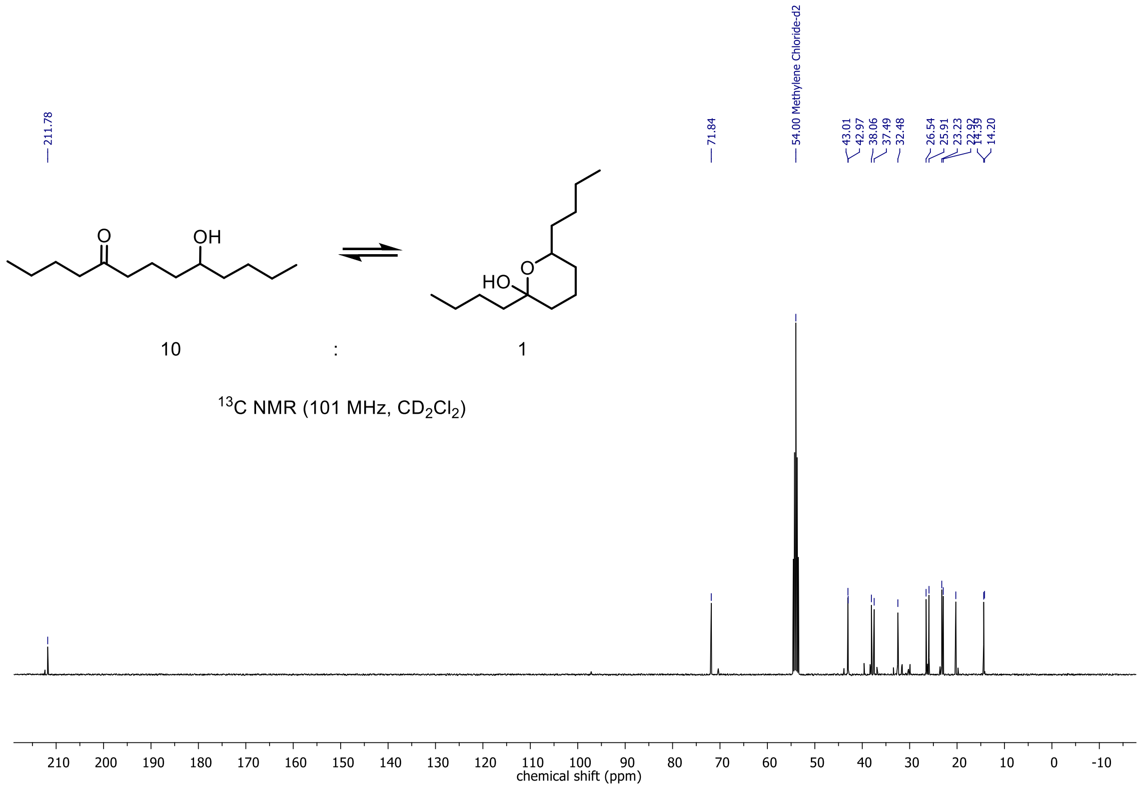

1-Cyclohexyl-5-hydroxydecan-1-one (1z)

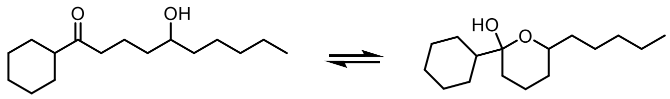

19.0 : 1

The title compound was prepared following general procedure F using 1-cyclohexyldec-9-en-1-one (24.6 mg, 0.10 mmol, 1.00 equiv.) and TfOH (27 µL, 0.30 mmol, 3.00 equiv.). Purification by flash column chromatography (10 – 40% EtOAc in heptanes) afforded the title compound (13.0 mg, 51 µmol, 51%) as a colorless oil and a single regioisomer. The δ-hydroxy ketone was observed to be in equilibrium with its hemiacetal form (ratio = 19.0:1).

Crude product: 71% NMR yield, r.r. ($\delta_{OH+Hemiacetal}$:γ_{OH}) = 89 : 11

An asterisk (*) denotes signals that unambiguously arise from the hemiacetal.

^{1}H NMR (400 MHz, CD_2Cl_2) δ 3.82 – 3.73 (m, 0.1H*), 3.50 (app br s, 0.9H), 2.45 (t, *J* = 7.1 Hz, 1.9H), 2.32 (ddd, *J* = 11.4, 7.3, 3.4 Hz, 0.9H), 1.85 – 1.73 (m, 4H), 1.68 – 1.53 (m, 4.2H), 1.45 – 1.17 (m, 15H), 0.89 (t, *J* = 6.9 Hz, 3H).

Due to low abundance, only signals arising from the major component (δ-hydroxy ketone) are reported in ^{13}C NMR.

^{13}C NMR (101 MHz, CD_2Cl_2) δ 214.5 (C), 71.7 (CH), 51.1 (CH), 40.7 (CH_2), 37.9 (CH_2), 37.4 (CH_2), 32.3 (CH_2), 29.0 (2CH_2), 26.3 (CH_2), 26.1 (2CH_2), 25.8 (CH_2), 23.1 (CH_2), 19.9 (CH_2), 14.2 (CH_3).

IR (neat) ν_{max}: 3422, 2926, 2854, 1702, 1449, 1375, 1145, 992, 739

HRMS (ESI$^+$): exact mass calculated for $[M+Na]^+$ $(C_{16}H_{30}O_2Na)^+$ requires *m*/*z* 277.2138, found *m*/*z* 277.2133.

1-Cyclohexyl-5-hydroxydecan-1-one (1z)

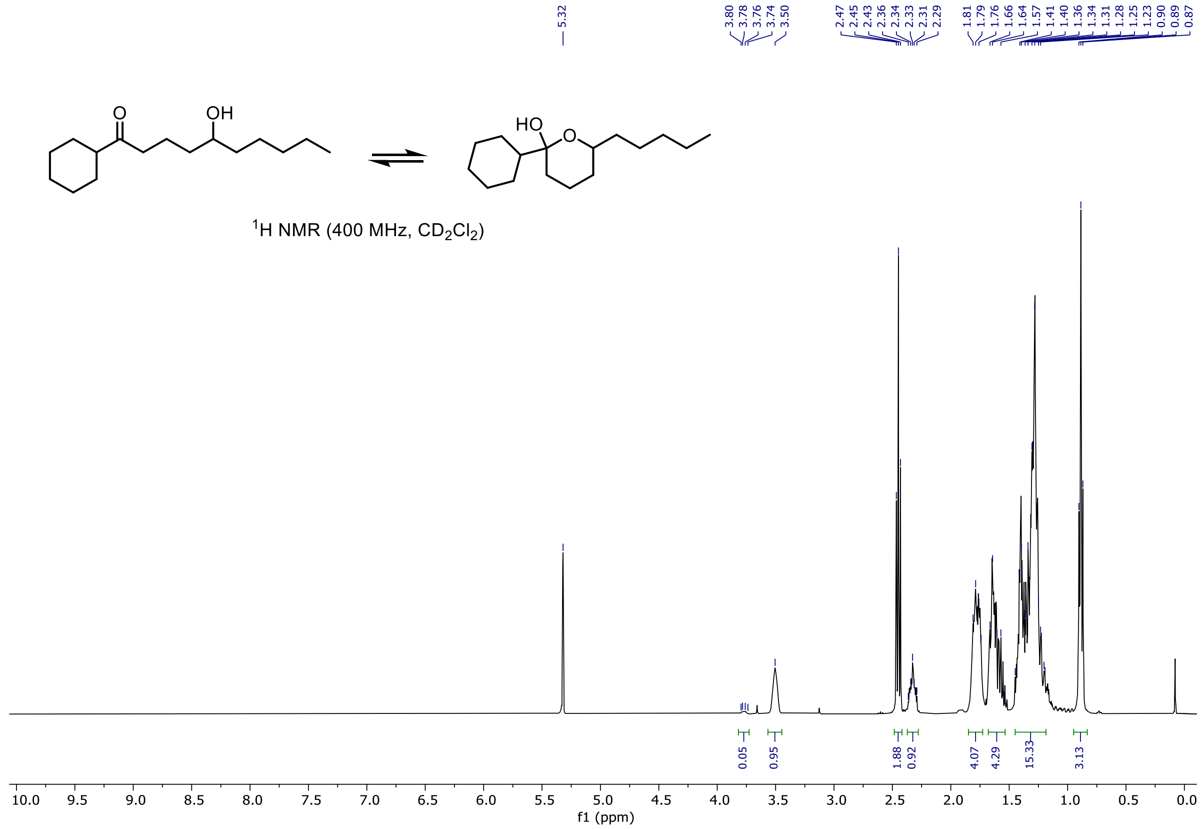

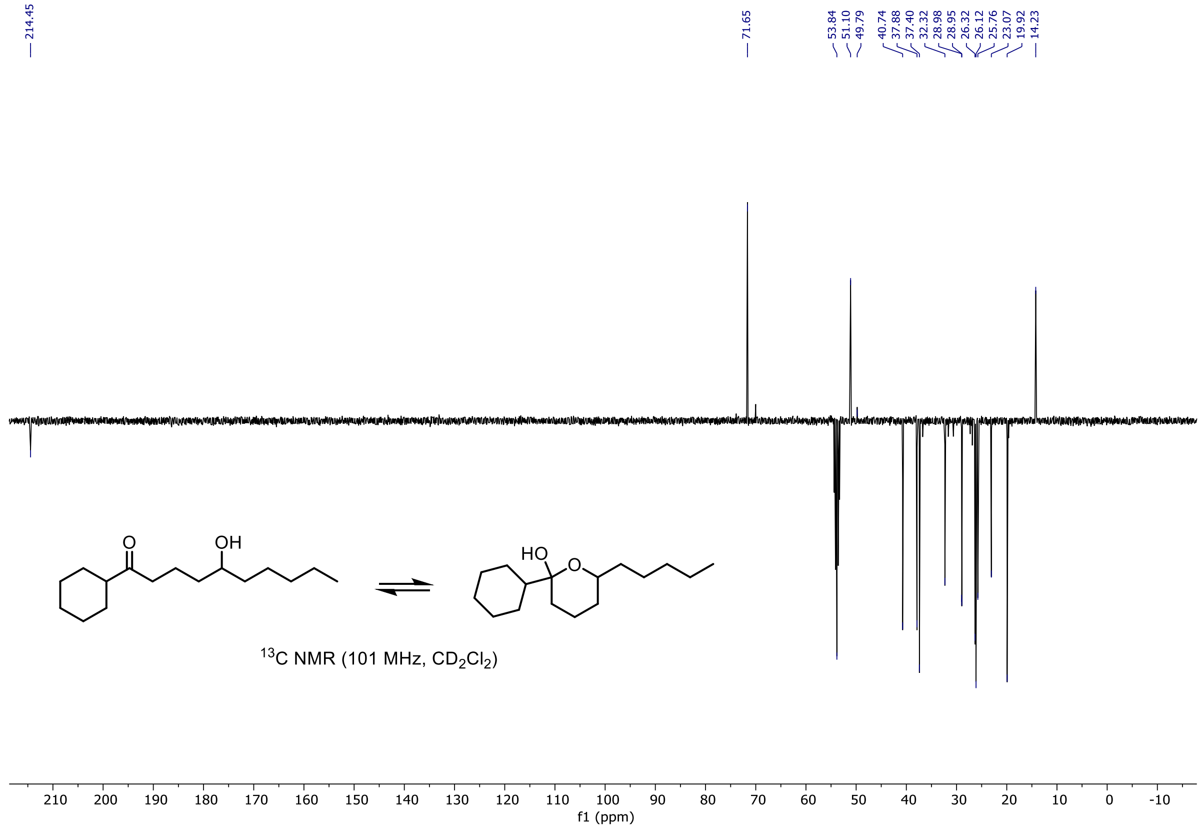

1-(Adamantan-1-yl)-5-hydroxynonan-1-one (1aa)

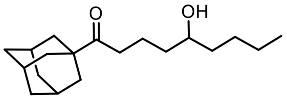

The title compound was prepared following general procedure F using 1-(adamantan-1-yl)non-8-en-1-one (27.4 mg, 0.10 mmol, 1.00 equiv.) and TfOH (27 µL, 0.30 mmol, 3.00 equiv.). Purification by flash column chromatography (0 – 100% EtOAc in heptanes) afforded the title compound (20 mg, 68 µmol, 68%) as a colorless liquid and a mixture of regioisomers (δ:γ = 93:7).

Crude product: NMR yield could not be determined due to signal overlap, r.r. (δ_{OH}:γ_{OH}) = 89:11.

A full list of signals arising from the minor γ-regioisomer can be found in Section 2.5.

^{1}H NMR (400 MHz, CD_2Cl_2): δ 3.56 – 3.44 (m, 1H), 2.46 (t, *J* = 7.1 Hz, 2H), 2.02 (s, 3H), 1.81 – 1.75 (m, 7H), 1.75 – 1.65 (m, 7H), 1.63 – 1.51 (m, 1H), 1.45 – 1.22 (m, 8H), 0.90 (t, *J* = 7.0 Hz, 3H).

^{13}C NMR (101 MHz, CD_2Cl_2): δ 216.1, 71.8, 46.8, 38.8 (3C), 37.8, 37.6, 37.1 (3C), 36.3, 28.7 (3C), 28.5, 23.3, 20.0, 14.4.

IR (neat) ν_{max}: 3440, 2903, 2850, 1695, 1452, 1407, 1344, 1266, 1198, 1159, 1051, 1005, 931, 741.

HRMS (ESI$^+$): exact mass calculated for $[M+Na]^+$ $(C_{19}H_{32}O_2Na)^+$ requires *m/z* 315.2294, found *m/z* 315.2289.

1-(Adamantan-1-yl)-5-hydroxynonan-1-one (1aa)

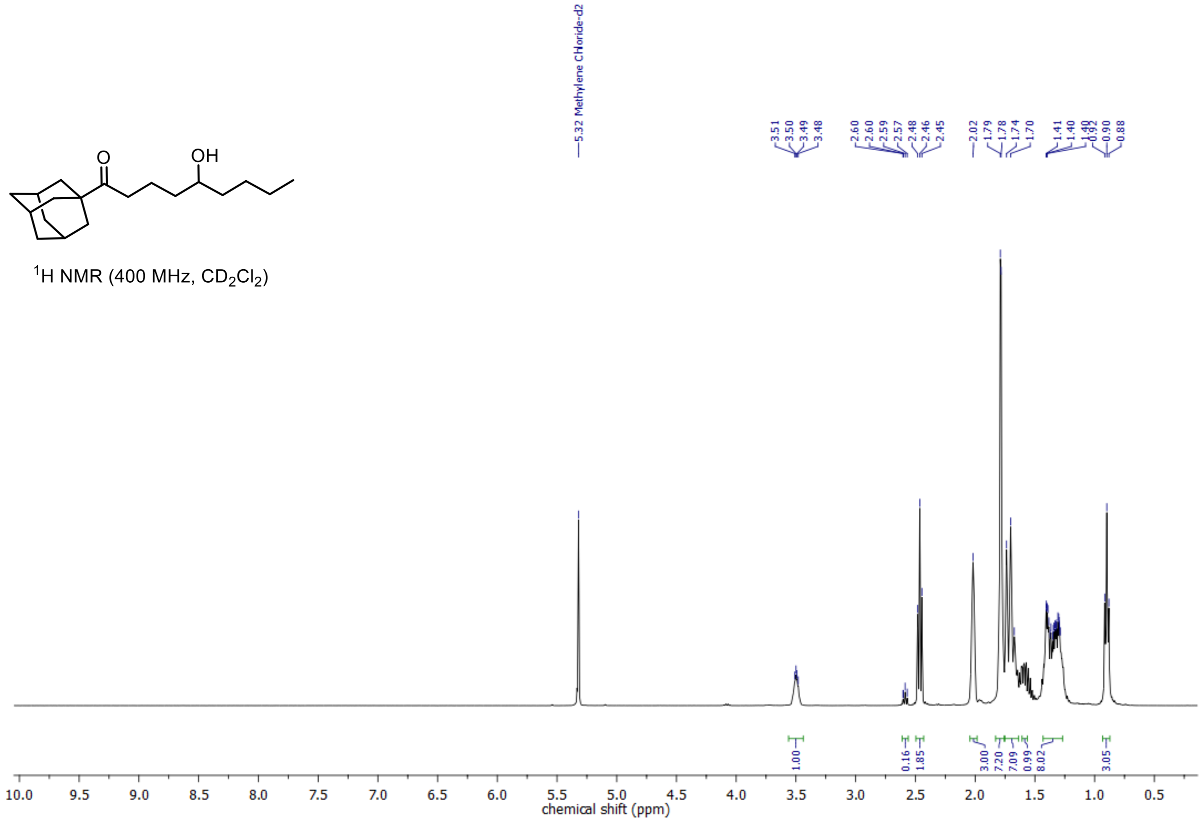

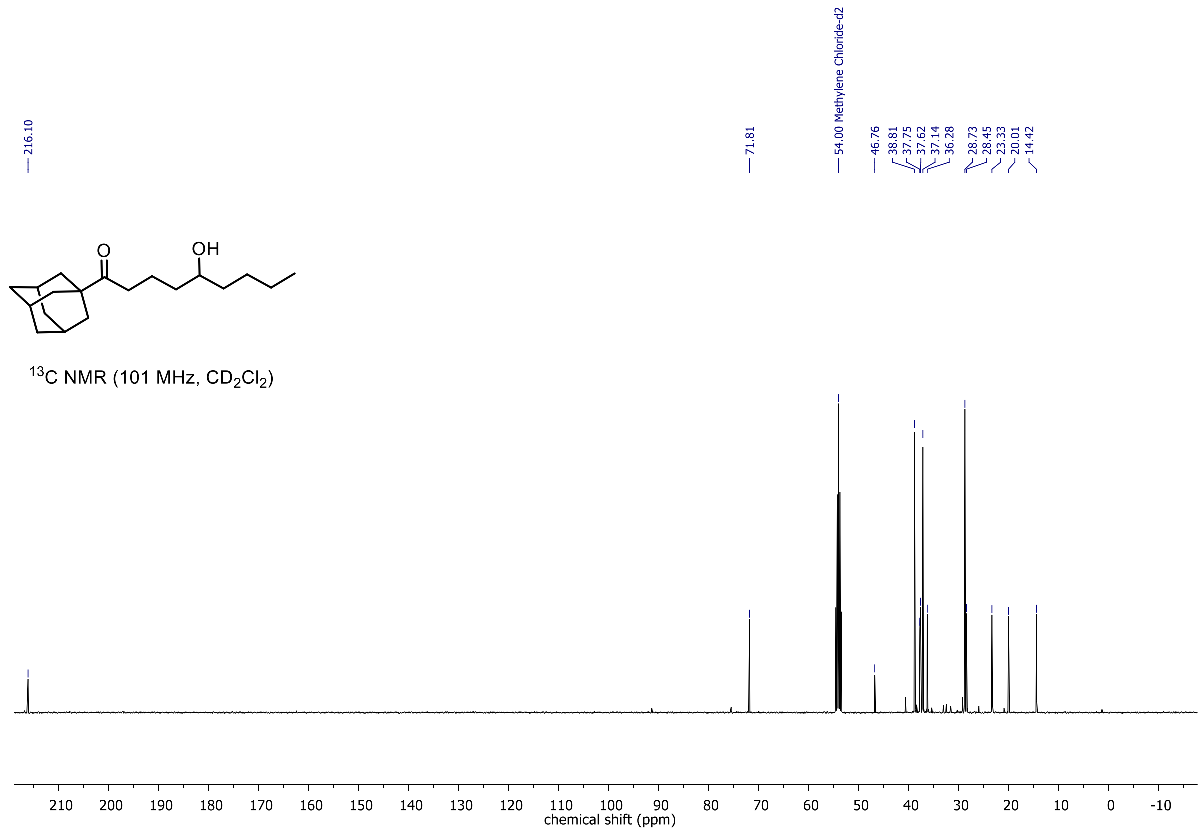

1-(4,5-Diphenyloxazol-2-yl)-7-hydroxyundecan-3-one (1ab)

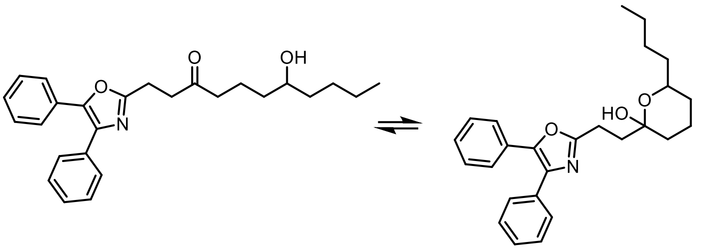

1.5 : 1

The title compound was prepared following general procedure F, with a modification to the reaction temperature (23 °C instead of 65 °C), using 1-(4,5-diphenyloxazol-2-yl)undec-10-en-3-one (38.7 mg, 0.10 mmol, 1.00 equiv.) and TfOH (27 µL, 0.30 mmol, 3.00 equiv.). Purification by flash column chromatography (0 – 50% EtOAc in heptanes) yielded the title compound (31.2 mg, 77 µmol, 77%) as a yellow oil and a single regioisomer. The δ-hydroxy ketone is in equilibrium with its hemiacetal form (ratio = 1.5:1).

Crude product: 97% NMR yield, r.r. ($\delta_{OH+Hemiacetal}$:γ_{OH}) = 91:9.

An asterisk (*) denotes signals that unambiguously arise from the hemiacetal.

^{1}H NMR (400 MHz, $CDCl_3$) δ 7.66 – 7.53 (m, 4H), 7.39 – 7.29 (m, 6H), 5.05 (s, 0.4H*), 3.97 – 3.83 (m, 0.4H*), 3.61 – 3.46 (m, 0.6H), 3.32 – 2.89 (m, 3H), 2.69 – 2.53 (m, 1H), 2.27 – 2.08 (m, 1H), 1.95 – 1.61 (m, 3.6H), 1.47 – 1.22 (m, 8H), 0.90 – 0.81 (m, 3H).

Owing to the comparable ratio of hydroxyketone and hemiacetal and frequent overlaps, only the distinctive ^{13}C resonances of the hemiacetal are marked with an asterisk. All remaining signals are reported collectively, as they could not be unambiguously assigned.

^{13}C NMR (101 MHz, $CDCl_3$) δ 209.0, 164.6, 162.5, 145.5, 145.3, 135.2, 134.6, 132.6, 132.3, 129.12, 129.09, 128.78 (2C), 128.77 (2C), 128.7 (3C), 128.63, 128.55, 128.2 (2C), 128.1 (2C), 128.0 (2C), 126.7 (3C), 126.6 (2C), 95.3*, 71.6, 69.8*, 42.8, 39.0, 38.7, 37.4, 36.8, 36.1, 35.2, 31.4, 27.9, 27.8, 22.9, 22.8, 22.3, 22.2, 19.9, 19.4, 14.22, 14.20.

IR (neat) ν_{max}: 3393, 2930, 2860, 1715, 1446, 1221, 1059, 1025, 964, 763, 694, 674.

HRMS (ESI$^+$): exact mass calculated for $[M+H]^+$ $(C_{26}H_{32}O_3N)^+$ requires *m/z* 406.2377, found *m/z* 406.2370.

1-(4,5-Diphenyloxazol-2-yl)-7-hydroxyundecan-3-one (1ab)

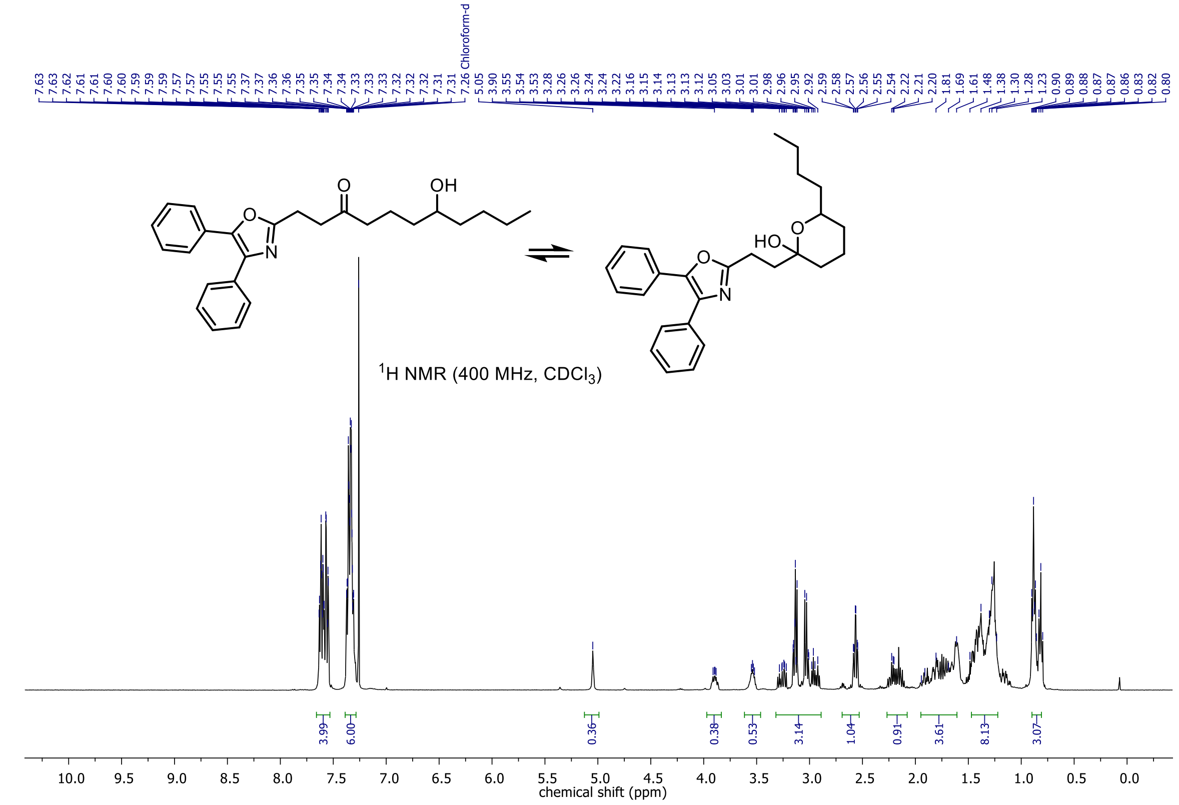

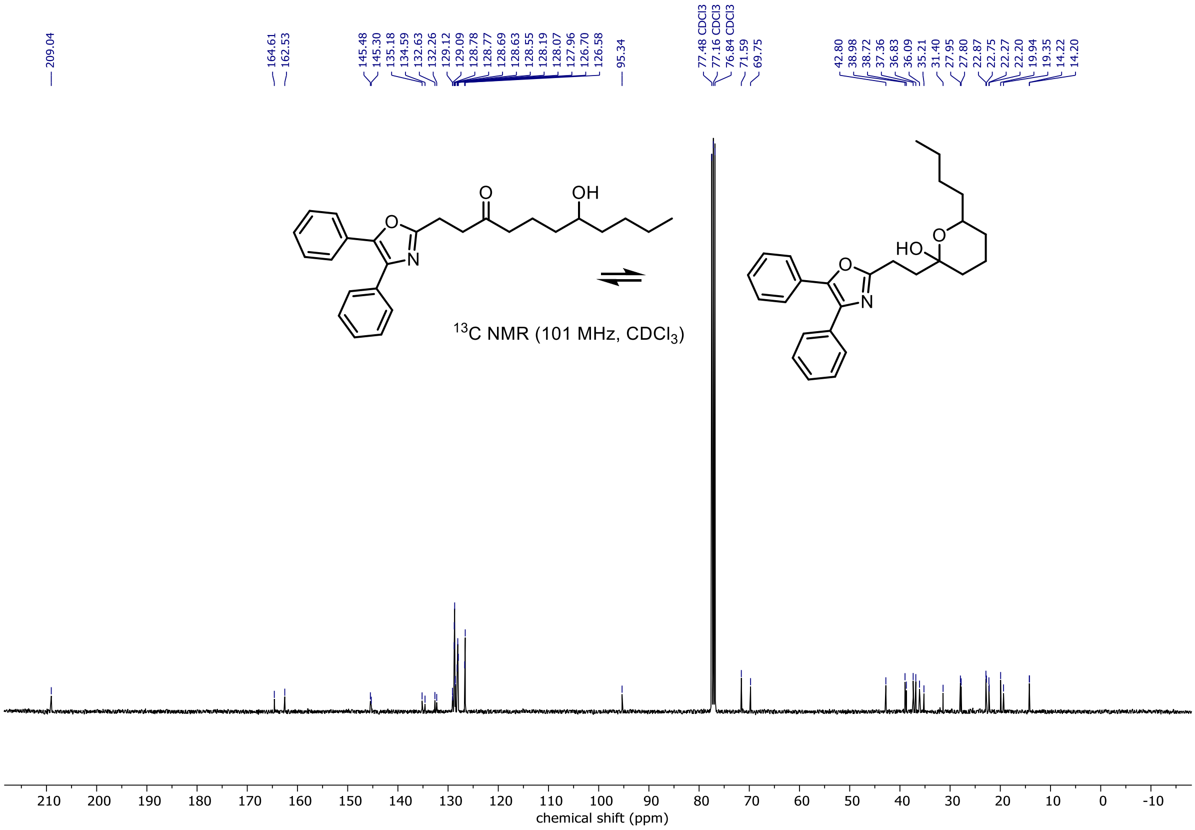

4-(5-Hydroxynonanoyl)-*N*,*N*-dipropylbenzenesulfonamide-4-(6-butyl-2-hydroxytetrahydro-2*H*-pyran-2-yl)-*N*,*N*-dipropylbenzenesulfonamide (1ac)

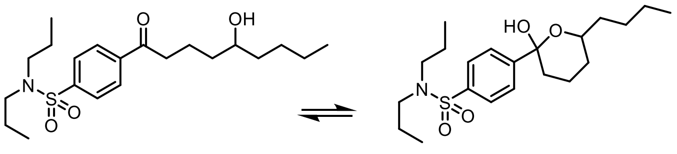

3.8 : 1

The title compound was prepared following general procedure F with a modification to the reaction temperature and time (35 °C instead of 65 °C, 2 h instead of 1 h) using 4-(non-8-enoyl)-*N*,*N*-dipropylbenzenesulfonamide (38 mg, 0.10 mmol, 1.00 equiv.) and TfOH (27 µL, 0.30 mmol, 3.00 equiv.). Purification by flash column chromatography (0 – 100% EtOAc in heptanes) afforded the title compound (18.6 mg, 47 µmol, 47%) as a colorless solid and a mixture of regioisomers (δ:γ = 93:7). The δ-hydroxy ketone is in equilibrium with its hemiacetal form (ratio = 3.8:1).

Crude product: 60% NMR yield, r.r. ($\delta_{OH+Hemiacetal}$:γ_{OH}) = 93:7

An asterisk (*) denotes signals that unambiguously arise from the hemiacetal. A full list of signals arising from the minor γ-regioisomer can be found in Section 2.5.

^{1}H NMR (400 MHz, CD_2Cl_2): δ 8.09 – 8.02 (m, 1.6H), 7.89 – 7.85 (m, 1.6H), 7.74 (m, 0.8H*), 4.10 – 4.00 (m, 0.2H*), 3.68 – 3.52 (m, 0.8H), 3.23 – 2.97 (m, 5.6H), 1.95 – 1.76 (m, 2H), 1.72 – 1.67 (m, 0.4H*), 1.57 – 1.50 (m, 6H), 1.49 – 1.40 (m, 3H), 1.36 – 1.29 (m, 4H), 0.93 – 0.89 (m, 3H), 0.86 (t, *J* = 7.4 Hz, 6H).

Due to low abundance, only signals arising from the major component (δ-hydroxy ketone) are reported in ^{13}C NMR.

^{13}C NMR (101 MHz, CD_2Cl_2): δ 199.8, 144.5, 140.3, 129.1 (2C), 127.8 (2C), 72.0, 50.5 (2C), 39.3, 37.9, 37.3, 28.4, 23.3, 22.5 (2C), 20.6, 14.4, 11.5 (2C).

IR (neat) ν_{max}: 3512, 2931, 2873, 1687, 1458, 1338, 1155, 990, 738, 600.

HRMS (ESI$^+$): exact mass calculated for $[M+Na]^+$ $(C_{21}H_{35}NO_4SNa)^+$ requires *m/z* 420.2179, found *m/z* 420.2175.

4-(5-Hydroxynonanoyl)-*N*,*N*-dipropylbenzenesulfonamide-4-(6-butyl-2-hydroxytetrahydro-2*H*-pyran-2-yl)-*N*,*N*-dipropylbenzenesulfonamide (1ac)

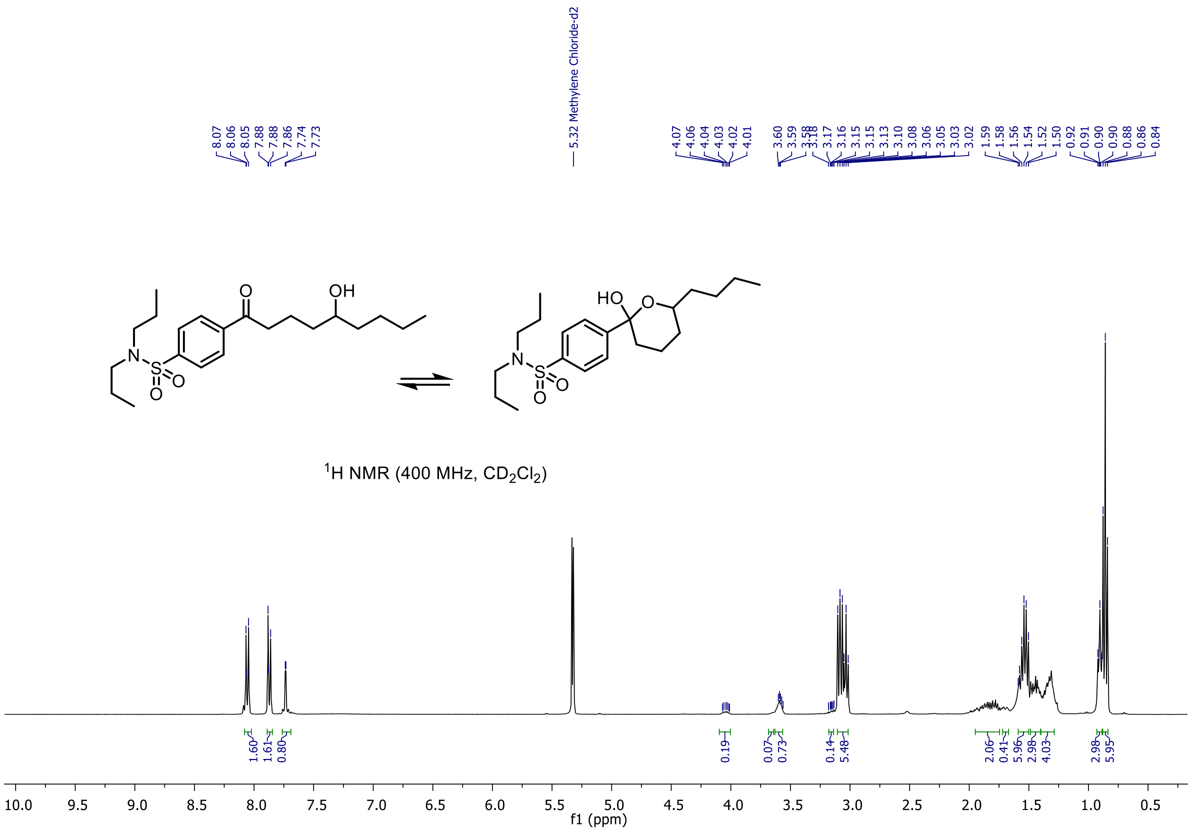

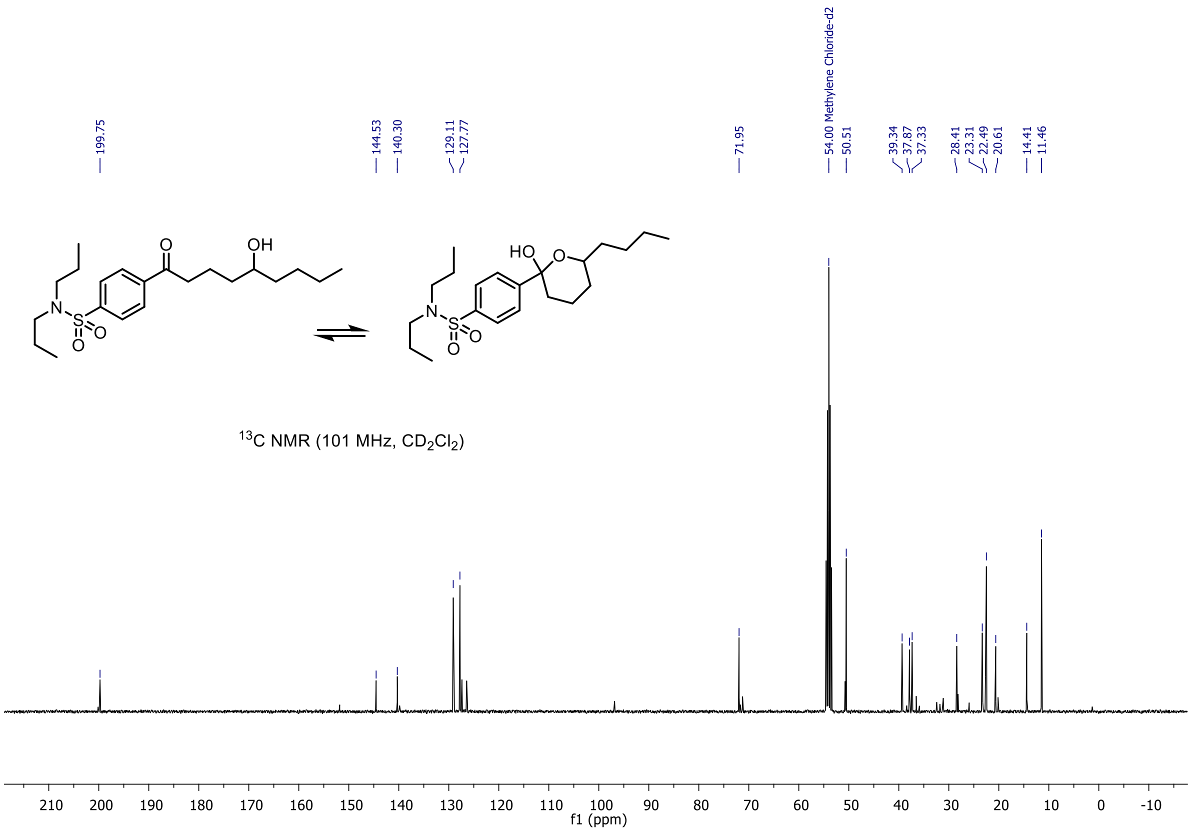

5-Hydroxy-1-(2,3,6,7-tetrahydro-1*H*,5*H*-pyrido[3,2,1-*ij*]quinolin-9-yl)nonan-1-one (1ad)

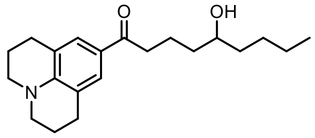

The title compound was prepared following general procedure F using 1-(2,3,6,7-Tetrahydro-1*H*,5*H*-pyrido[3,2,1-*ij*]quinolin-9-yl)non-8-en-1-one (6.2 mg, 20 µmol, 1.00 equiv.) and TfOH (5.3 µL, 6 µmol, 3.00 equiv.). Purification by flash column chromatography (0 – 100% EtOAc/EtOH/28% NH_4OH (3:1:0.02) in heptanes) afforded the title compound (3.4 mg, 10.3 µmol, 52%) as a yellow oil and a mixture of regioisomers (δ:γ = 89:11).

Crude product: Neither NMR yield nor r.r. could be determined due to signal overlap

Although the γ-isomer was not prepared independently, the minor product obtained in this reaction was determined to be the γ-isomer by analogy (chemical shifts & splitting patterns) with the other reported compounds.

^{1}H NMR (400 MHz, $(CD_3)_2CO$): δ 7.40 (s, 2H), 3.56 – 3.50 (m, 1H), 3.37 (d, *J* = 5.3 Hz, 1H), 3.33 – 3.21 (m, 4H), 2.83 – 2.73 (m, 2H), 2.76 – 2.69 (m, 4H), 1.96 – 1.89 (m, 4H), 1.83 – 1.66 (m, 2H), 1.52 – 1.37 (m, 5H), 1.33 – 1.29 (m, 3H), 0.89 (t, *J* = 7.1 Hz, 3H).

^{13}C NMR (101 MHz, $(CD_3)_2CO$): δ 197.9, 147.3, 128.4 (2C), 125.1, 120.6 (2C), 71.2, 50.4 (2C), 38.3, 38.2, 38.0, 28.8, 28.5 (2C), 23.5, 22.3 (2C), 22.0, 14.4.

IR (neat) ν_{max}: 3440, 2929, 2856, 2359, 1704, 1596, 1438, 1314, 1248, 1161.

HRMS (ESI$^+$): exact mass calculated for $[M+H]^+$ $(C_{21}H_{31}N_2O_2)^+$ requires *m/z* 330.2427, found *m/z* 330.2428.

5-Hydroxy-1-(2,3,6,7-tetrahydro-1*H*,5*H*-pyrido[3,2,1-*ij*]quinolin-9-yl)nonan-1-one (1ad)

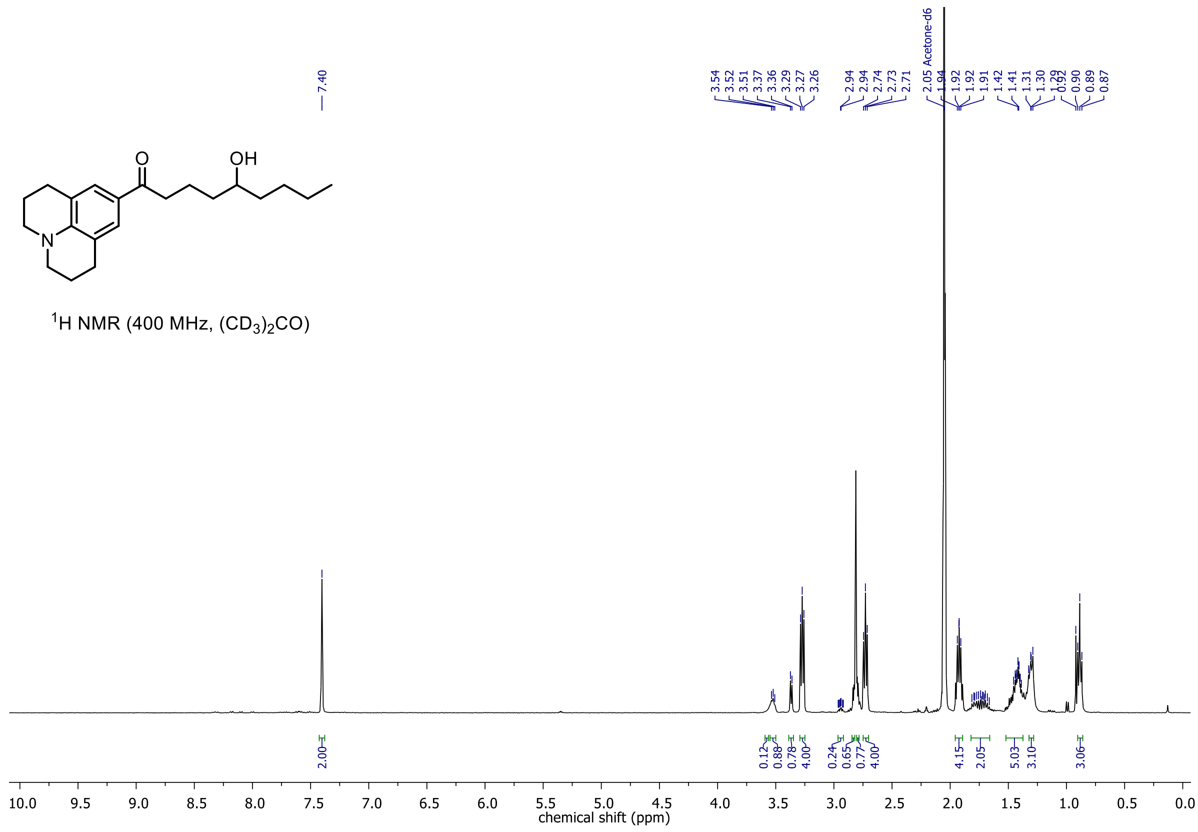

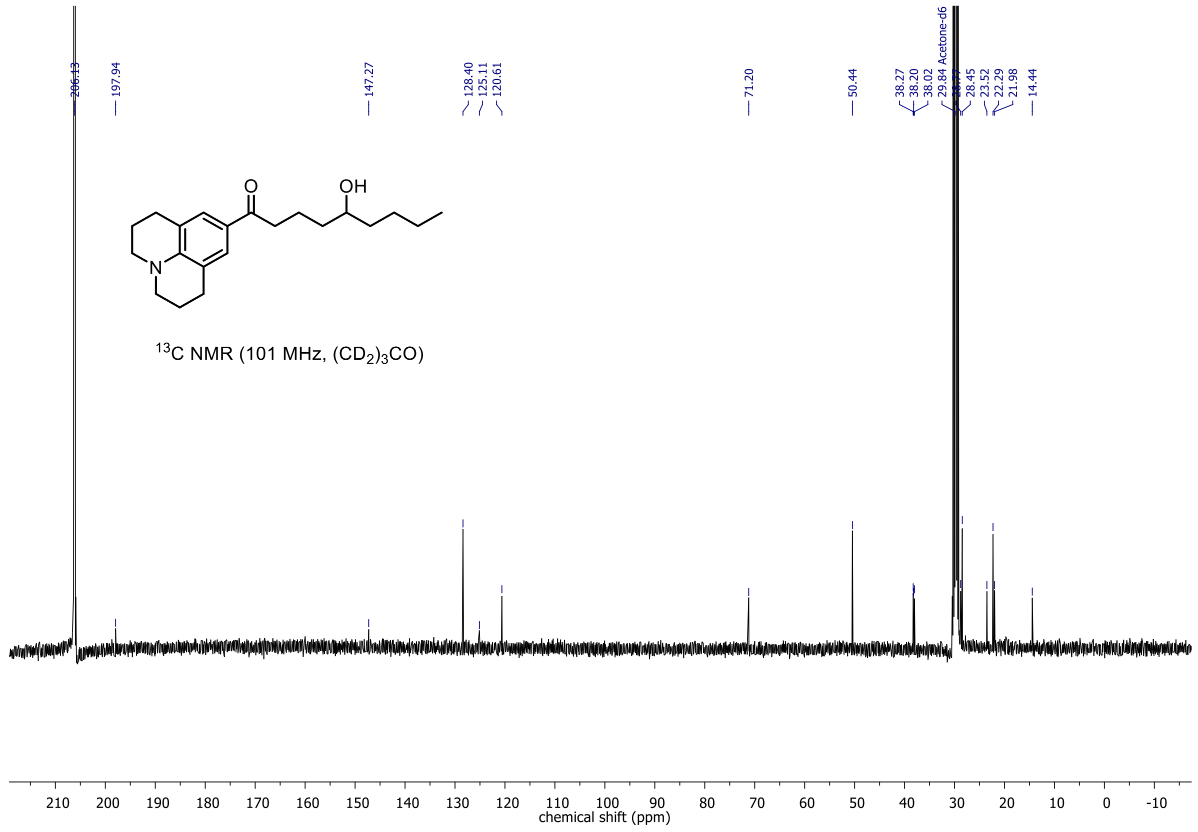

2.5 Characterization of γ-alcohols

4-Hydroxy-1-phenylheptan-1-one (2a)

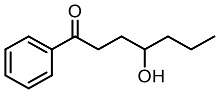

The title compound was prepared following general procedure G using 1-phenylhept-6-en-1-one (18.8 mg, 0.10 mmol, 1.00 equiv.) and TfOH (27 µL, 0.30 mmol, 3.00 equiv.). Purification by flash column chromatography (0 – 70% EtOAc in heptanes) afforded the title compound (9.1 mg, 44 µmol, 44%) as a yellow oil and single regioisomer.

Crude product: 47% NMR yield, r.r. (γ_{OH}:δ_{OH}) > 95:5.

All analytical data were in good agreement with those reported in literature.[14]

^{1}H NMR (400 MHz, CD_2Cl_2): δ 7.94 – 7.79 (m, 2H), 7.52 – 7.46 (m, 1H), 7.42 – 7.34 (m, 2H), 3.60 – 3.41 (m, 1H), 3.13 – 2.93 (m, 2H), 1.91 – 1.33 (m, 7H), 0.85 (t, *J* = 6.8 Hz, 3H).

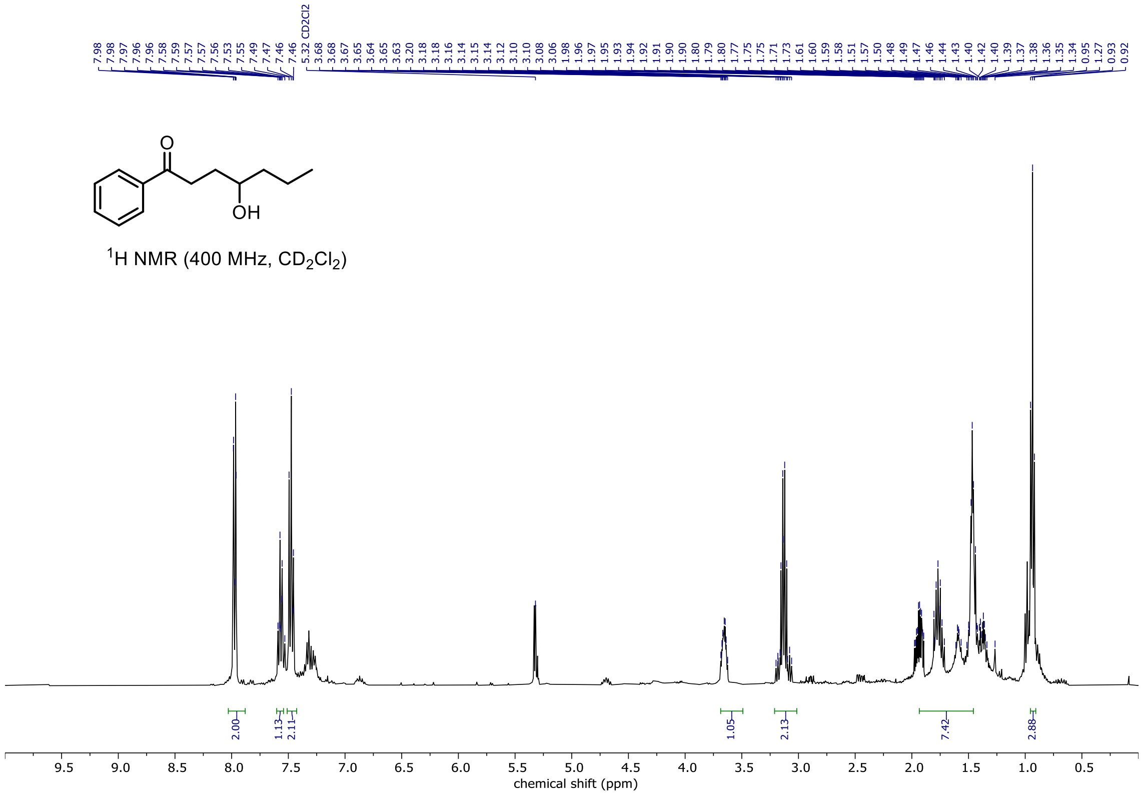

4-Hydroxy-1-phenylnonan-1-one (2b)

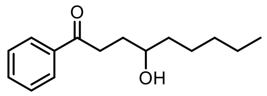

The title compound was prepared following general procedure G using 1-phenylnon-8-en-1-one (21.6 mg, 0.10 mmol, 1.00 equiv.) and TfOH (27 µL, 0.30 mmol, 3.00 equiv.). Purification by flash column chromatography (10 – 40% EtOAc in heptanes) afforded the title compound (15.6 mg, 67 µmol, 67%) as a colorless solid and a single regioisomer.

Crude product: 74% NMR yield; r.r. (γ_{OH}:δ_{OH}) = 94:6

All analytical data were in good agreement with those reported in literature.[14]

^{1}H NMR (400 MHz, CDCl$_3$): δ 8.01 – 7.94 (m, 2H), 7.58 – 7.53 (m, 1H), 7.50 – 7.42 (m, 2H), 3.67 (s, 1H), 3.23 – 3.07 (m, 2H), 2.03 – 1.93 (m, 1H), 1.85 – 1.77 (m, 2H), 1.53 – 1.40 (m, 3H), 1.38 – 1.27 (m, 5H), 0.89 (t, *J* = 6.7 Hz, 3H).

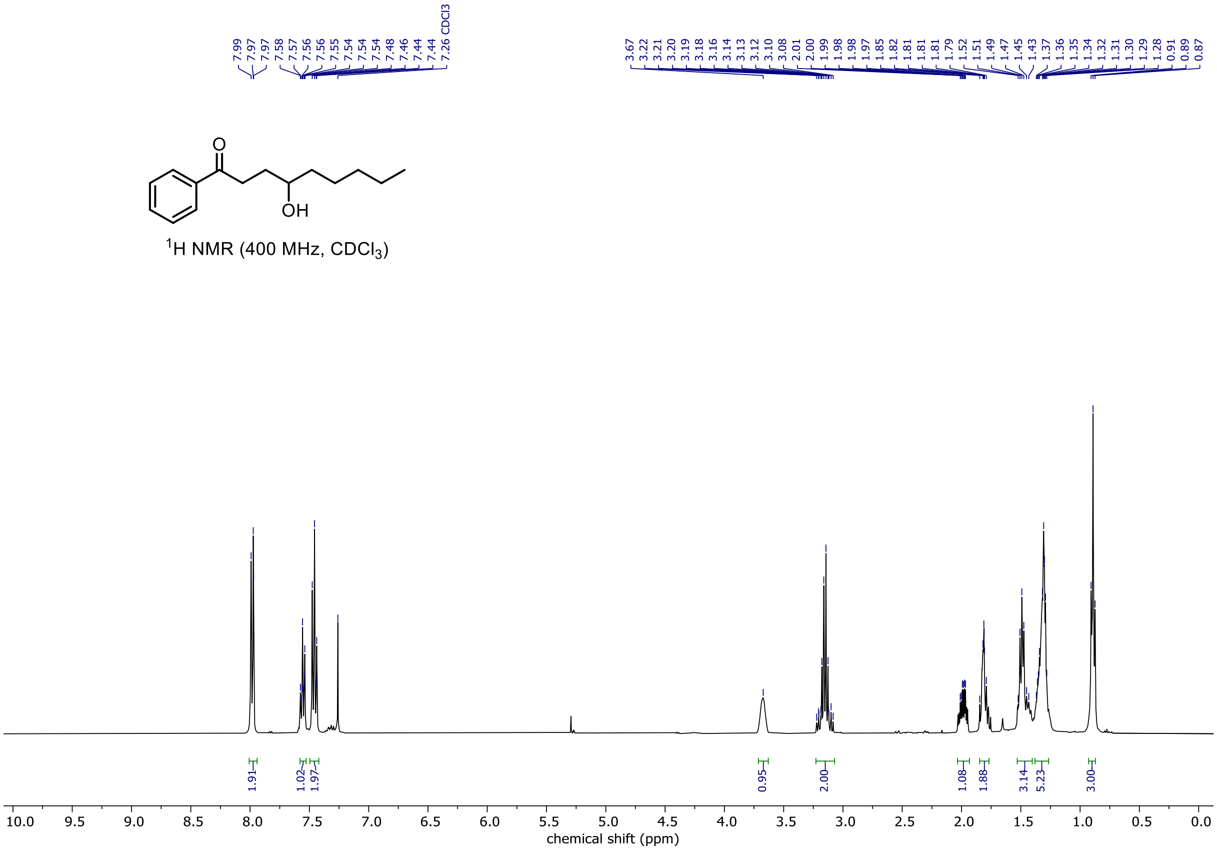

4-Hydroxy-1-phenyldecan-1-one (2c)

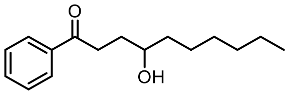

The title compound was prepared following general procedure G using 1-phenyldec-9-en-1-one (24.0 mg, 0.10 mmol, 1.00 equiv.) and TfOH (27 µL, 0.30 mmol, 3.00 equiv.). Purification by flash column chromatography (10 – 40% EtOAc in heptanes) afforded the title compound (12.3 mg, 50 µmol, 50%) as a colorless solid and a single regioisomer.

Crude product: 62% NMR yield; r.r. (γ_{OH}:δ_{OH}) = 93:7

^{1}H NMR (400 MHz, CD_2Cl_2) δ 8.02 – 7.93 (m, 2H), 7.61 – 7.54 (m, 1H), 7.52 – 7.44 (m, 2H), 3.64 (app br s, 1H), 3.21 – 3.05 (m, 2H), 1.99 – 1.90 (m, 1H), 1.82 – 1.71 (m, 2H), 1.53 – 1.27 (m, 10H), 0.89 (t, *J* = 6.8 Hz, 3H).

^{13}C NMR (101 MHz, CD_2Cl_2) δ 201.0 (C), 137.5 (C), 133.3 (CH), 129.0 (2CH), 128.4 (2CH), 71.7 (CH), 38.3 (CH_2), 35.4 (CH_2), 32.3 (CH_2), 31.9 (CH_2), 29.8 (CH_2), 26.1 (CH_2), 23.0 (CH_2), 14.3 (CH_3).

IR (neat) ν_{max}: 3465, 2953, 2927, 2856, 1685, 1449, 1272, 1026, 741, 712, 699, 690.

HRMS (ESI^{+}): exact mass calculated for $[M+Na]^{+}$ $(C_{16}H_{24}O_2Na)^{+}$ requires *m*/*z* 271.1669, found *m*/*z* 271.1666.

4-Hydroxy-1-phenyldecan-1-one (2c)

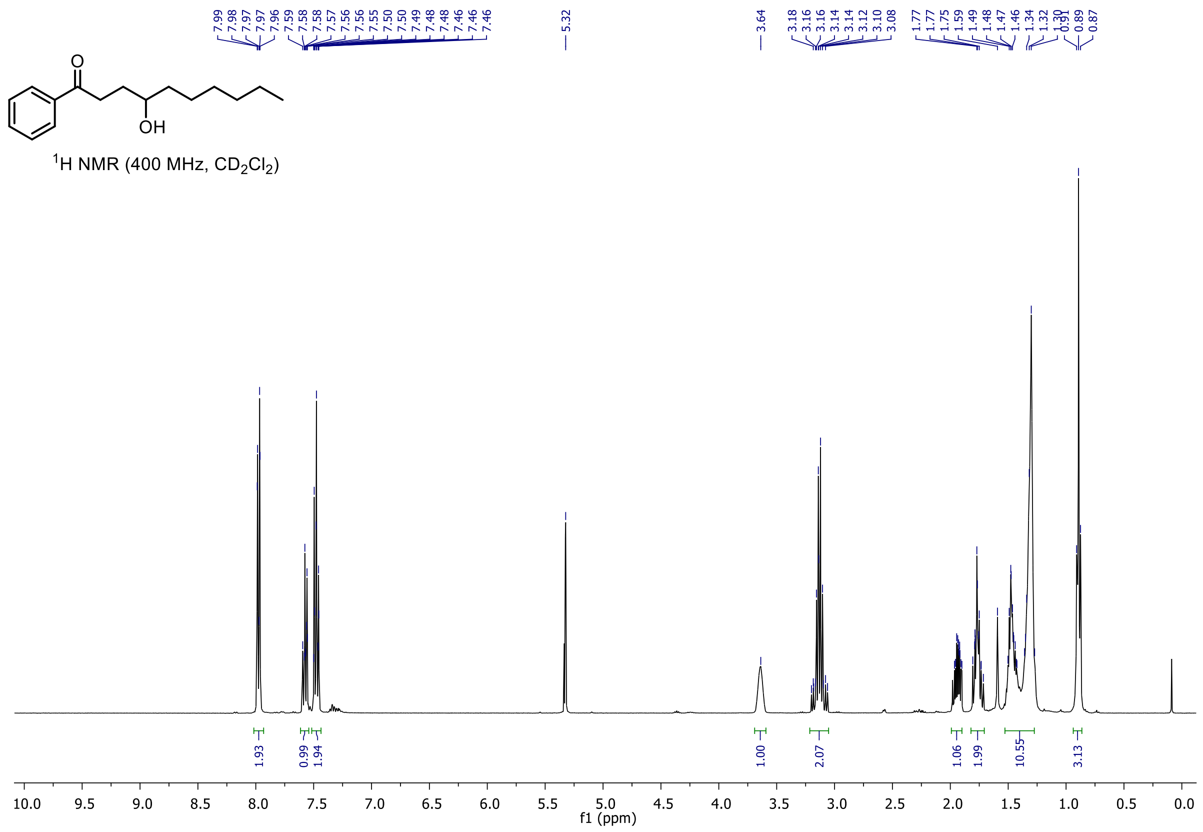

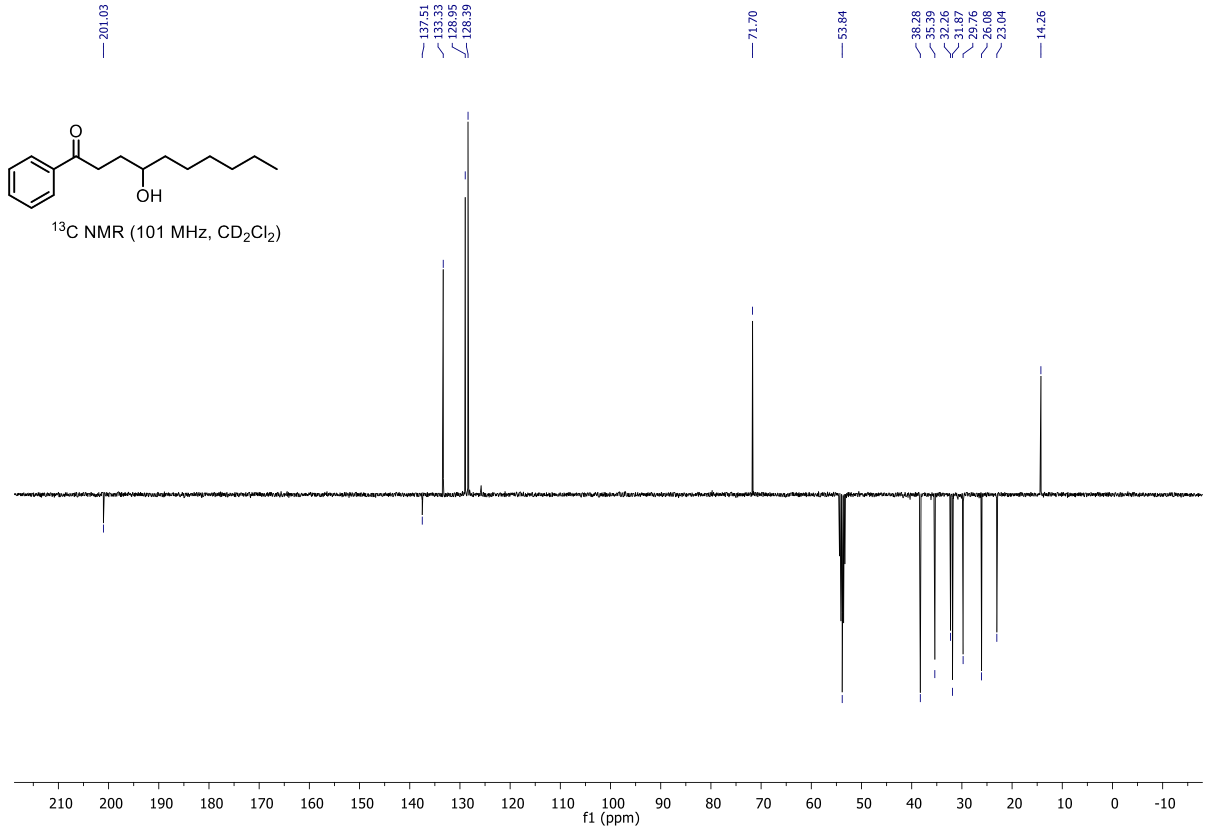

4-Hydroxy-1-phenyldodecan-1-one (2d)

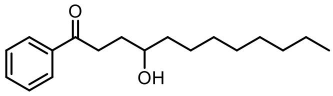

The title compound was prepared following general procedure H using 1-phenyldodec-11-en-1-one (25.8 mg, 0.10 mmol, 1.00 equiv.), TfOH (53 µL, 0.60 mmol, 6.00 equiv.) and NEt_3 (42 µL, 0.30 mmol, 3.00 equiv.). Purification by flash column chromatography (4 – 30% EtOAc in heptanes with 1% triethylamine additive) afforded the title compound (17.3 mg, 63 µmol, 63%) as a colorless solid and a single regioisomer.

Crude product: 71% NMR yield, r.r. (γ_{OH}:δ_{OH}) > 95:5

^{1}H NMR (400 MHz, CD_2Cl_2) δ 8.01 – 7.94 (m, 2H), 7.62 – 7.53 (m, 1H), 7.51 – 7.43 (m, 2H), 3.64 (br s, 1H), 3.22 – 3.04 (m, 2H), 1.98 – 1.88 (m, 1H), 1.81 – 1.70 (m, 2H), 1.53 – 1.40 (m, 3H), 1.37 – 1.21 (m, 11H), 0.88 (t, *J* = 6.9 Hz, 3H).

^{13}C NMR (101 MHz, CD_2Cl_2) δ 201.0 (C), 137.5 (C), 133.3 (CH), 128.9 (2CH), 128.4 (2CH), 71.7 (CH), 38.3 (CH_2), 35.4 (CH_2), 32.3 (CH_2), 31.9 (CH_2), 30.1 (CH_2), 30.0 (CH_2), 29.7 (CH_2), 26.1 (CH_2), 23.1 (CH_2), 14.3 (CH_3).

IR (neat) ν_{max}: 3379, 2951, 2916, 2848, 1684, 1446, 1270, 1092, 891, 738.

HRMS (ESI$^+$): exact mass calculated for $[M+Na]^+$ $(C_{18}H_{28}O_2Na)^+$ requires *m*/*z* 299.1982, found *m*/*z* 299.1978.

4-Hydroxy-1-phenyldodecan-1-one (2d)

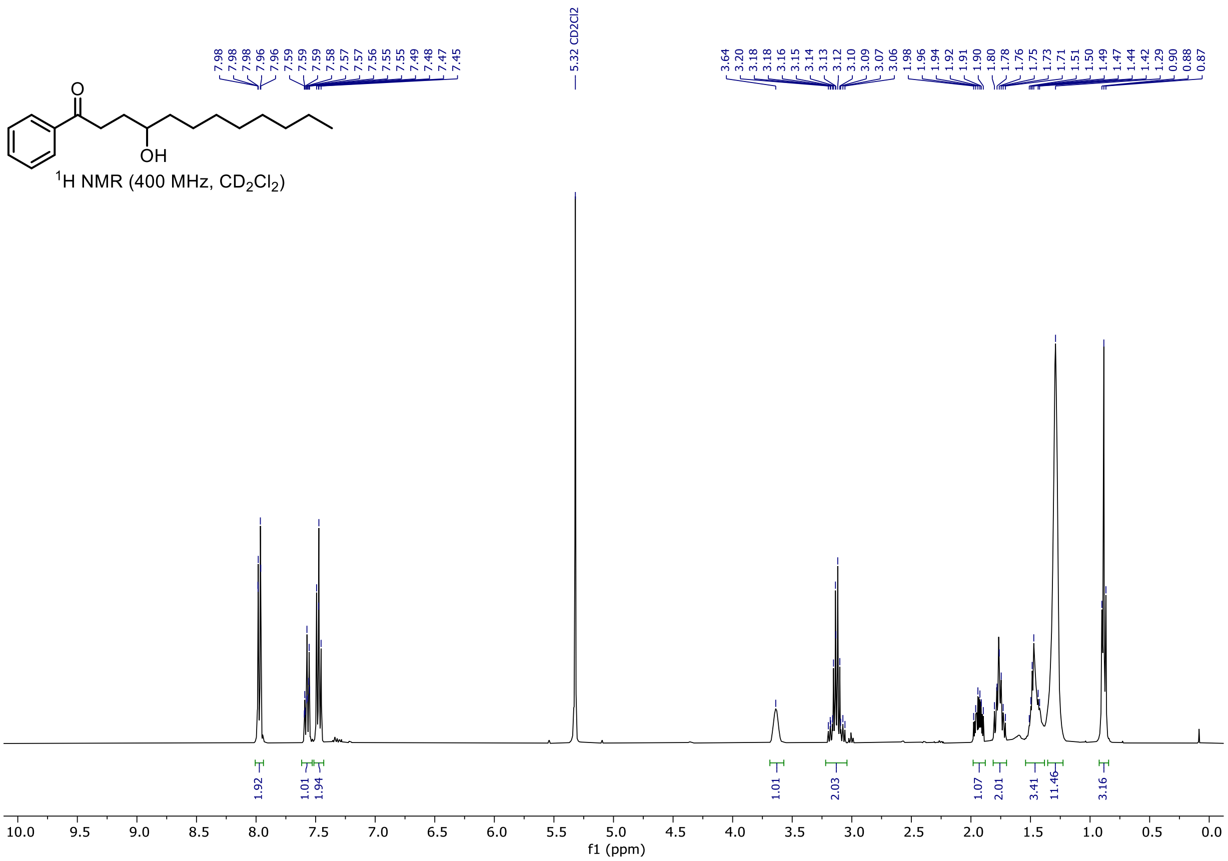

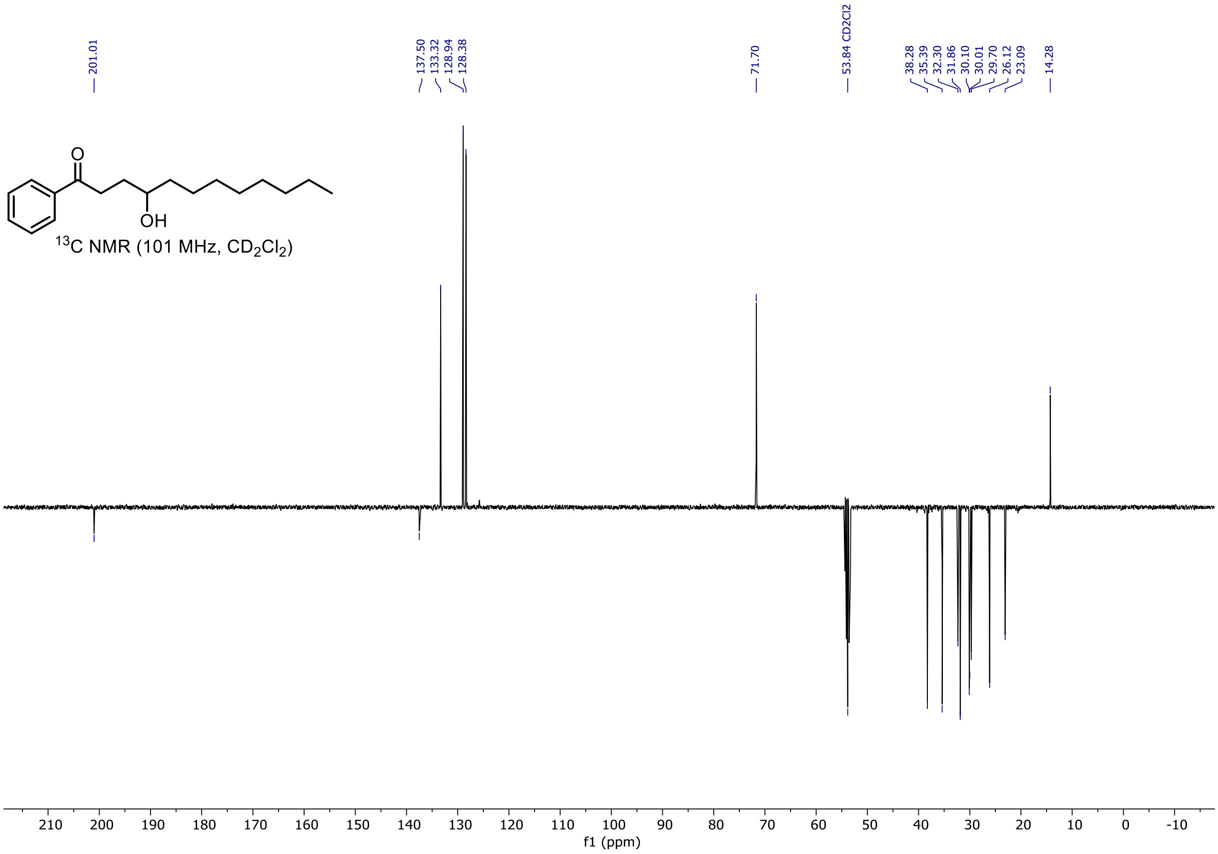

4-Hydroxy-1-phenylnonan-1-one (2b)

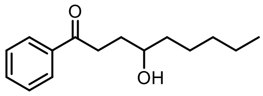

The title compound was prepared following general procedure G using 1-phenylnon-6-en-1-one (21.6 mg, 0.10 mmol, 1.00 equiv.) and TfOH (27 µL, 0.30 mmol, 3.00 equiv.). Purification by flash column chromatography (0 – 100% EtOAc in heptanes) afforded the title compound (12.8 mg, 55 µmol, 55%) as a yellow solid and single regioisomer.

Crude product: 61% NMR yield, r.r. (γ_{OH}:δ_{OH}) = 91:9

All analytical data were identical to those reported in Section 2.5.

4-Hydroxy-1-phenylundecan-1-one (2e)

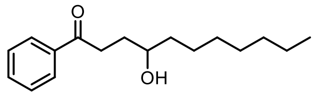

The title compound was prepared following general procedure G using 1-phenylundec-8-en-1-one (24.4 mg, 0.10 mmol, 1.00 equiv.) and TfOH (27 µL, 0.30 mmol, 3.00 equiv.). Purification by flash column chromatography (0 – 100% EtOAc in heptanes) afforded the title compound (13.0 mg, 50 µmol, 50%) as a yellowish solid and a single regioisomer.

Crude product: 55% NMR yield, r.r. (γ_{OH}:δ_{OH}) = 92:8.

^{1}H NMR (400 MHz, (CD_2Cl_2): δ 7.97 (m, 2H), 7.56 (m, 1H), 7.47 (m, 2H), 3.63 (app br s, 1H), 3.25 – 3.02 (m, 2H), 1.94 (m, 1H), 1.77 (m, 1H), 1.55 (br s, 1H), 1.47 (m, 3H), 1.30 (app br s, 9H), 0.88 (t, *J* = 6.8 Hz, 3H).

^{13}C NMR (101 MHz, (CD_2Cl_2): δ 201.2, 137.7, 133.5, 129.1 (2C), 128.5 (2C), 71.9, 38.4, 35.6, 32.4, 32.0, 30.2, 29.9, 26.3, 23.2, 14.4.

IR (neat) ν_{max}: 3393, 2923, 2853, 2362, 1685, 1597, 1448, 1272, 1077, 739, 688, 568.

HRMS (ESI$^+$): exact mass calculated for $[M+Na]^+$ $(C_{17}H_{26}O_2Na)^+$ requires *m/z* 285.1825, found *m/z* 285.1818.

4-Hydroxy-1-phenylundecan-1-one (2e)

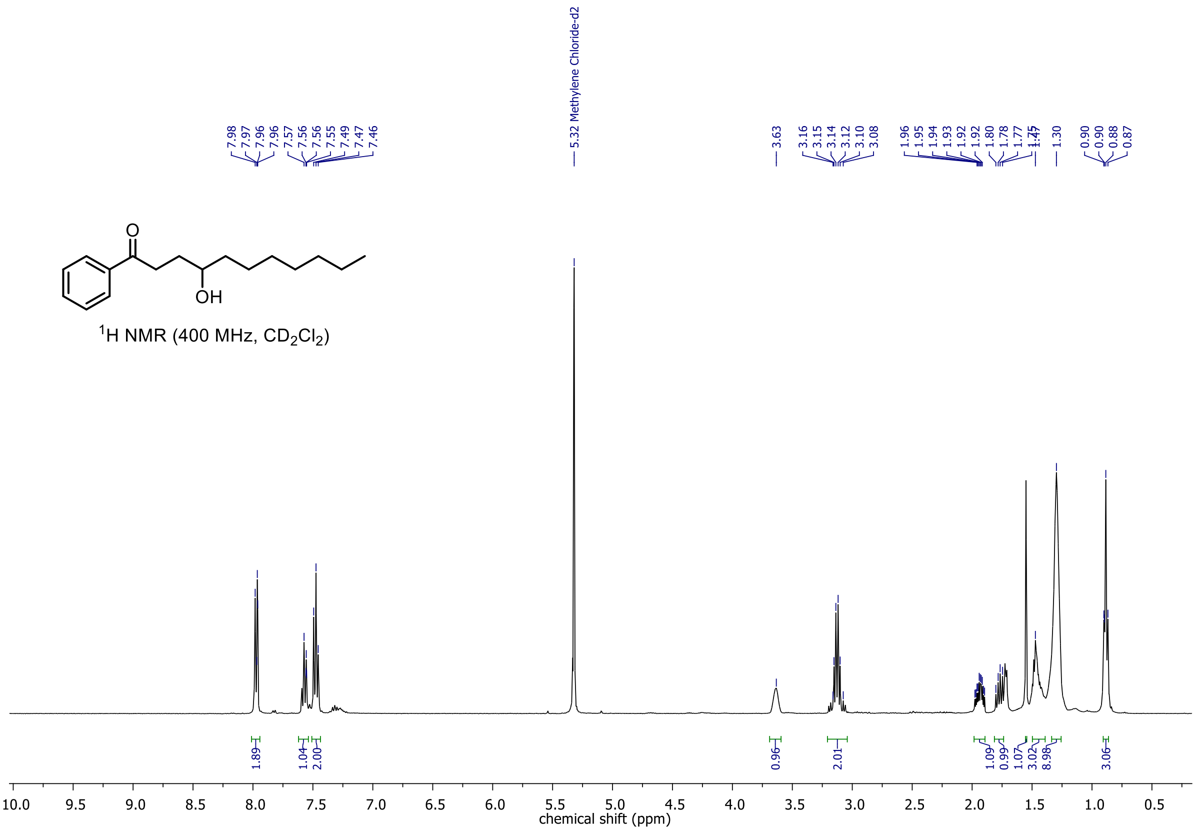

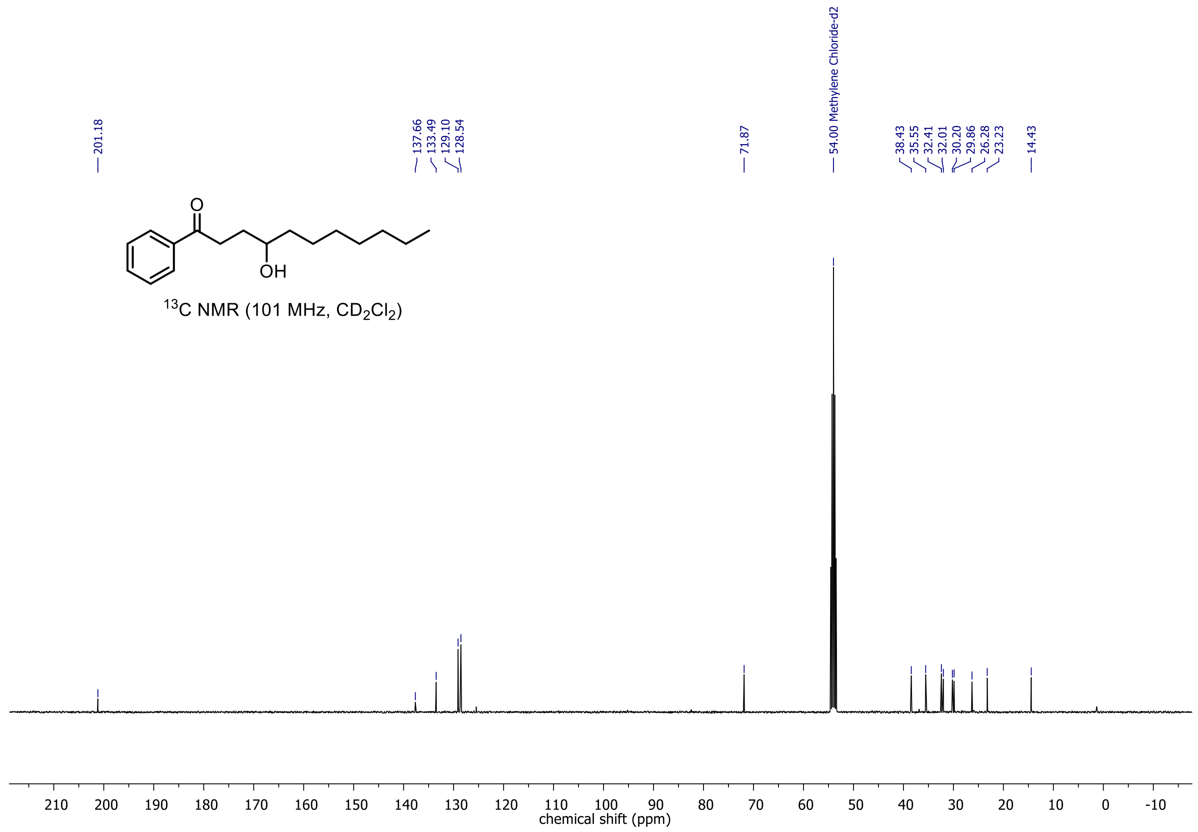

10,10,10-Trifluoro-4-hydroxy-1-phenyldecan-1-one (2f)

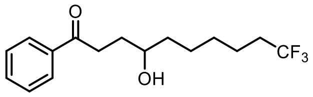

The title compound was prepared following general procedure G using (*E*)-10,10,10-trifluoro-1-phenyldec-7-en-1-one (28.4 mg, 0.10 mmol, 1.00 equiv.) and TfOH (27 µL, 0.30 mmol, 3.00 equiv.). Purification by flash column chromatography (0 – 70% EtOAc in heptanes) afforded the title compound (24.1 mg, 80 µmol, 80%) as a white solid and a mixture of regioisomers (γ:δ = 94:6).

Crude product: 96% NMR yield, r.r. (γ_{OH}:δ_{OH}) = 89:11.

A full list of signals arising from the minor δ-regioisomer can be found in Section 2.5.

^{1}H NMR (400 MHz, CDCl$_3$): δ 8.00 – 7.94 (m, 2H), 7.61 – 7.55 (m, 1H), 7.50 – 7.45 (m, 2H), 3.69 – 3.55 (m, 1H), 3.21 – 3.06 (m, 2H), 2.17 – 2.02 (m, 2H), 1.99 – 1.71 (m, 3H), 1.61 – 1.37 (m, 8H).

^{13}C NMR (101 MHz, CDCl$_3$): δ 201.2, 137.6, 133.6, 129.1 (2C), 128.6 (2C), 128.1 (q, *J* = 276.2 Hz), 71.7, 38.1, 35.5, 34.1 (q, *J* = 28.1 Hz), 32.0, 29.3, 25.9, 22.4 (q, *J* = 2.8 Hz).

^{19}F NMR (377 MHz, CDCl$_3$): δ -64.84 (t, *J* = 11.1 Hz).

IR (neat) ν_{max}: 3458, 1939, 2862, 1714, 1684, 1598, 1448, 1387, 1362, 1253, 1133, 1025, 760, 743, 700.

HRMS (ESI$^+$): exact mass calculated for $[M+Na]^+$ $(C_{16}H_{21}F_3O_2Na)^+$ requires *m/z* 325.1386, found *m/z* 325.1393.

10,10,10-Trifluoro-4-hydroxy-1-phenyldecan-1-one (2f)

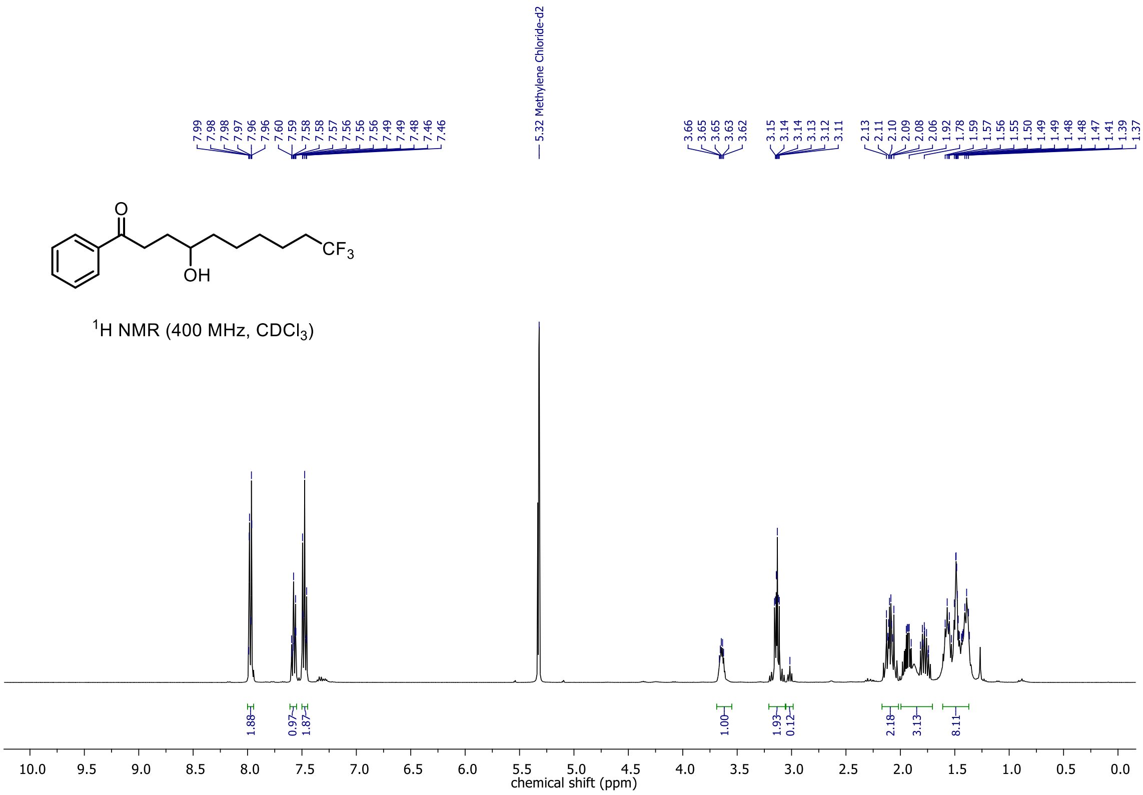

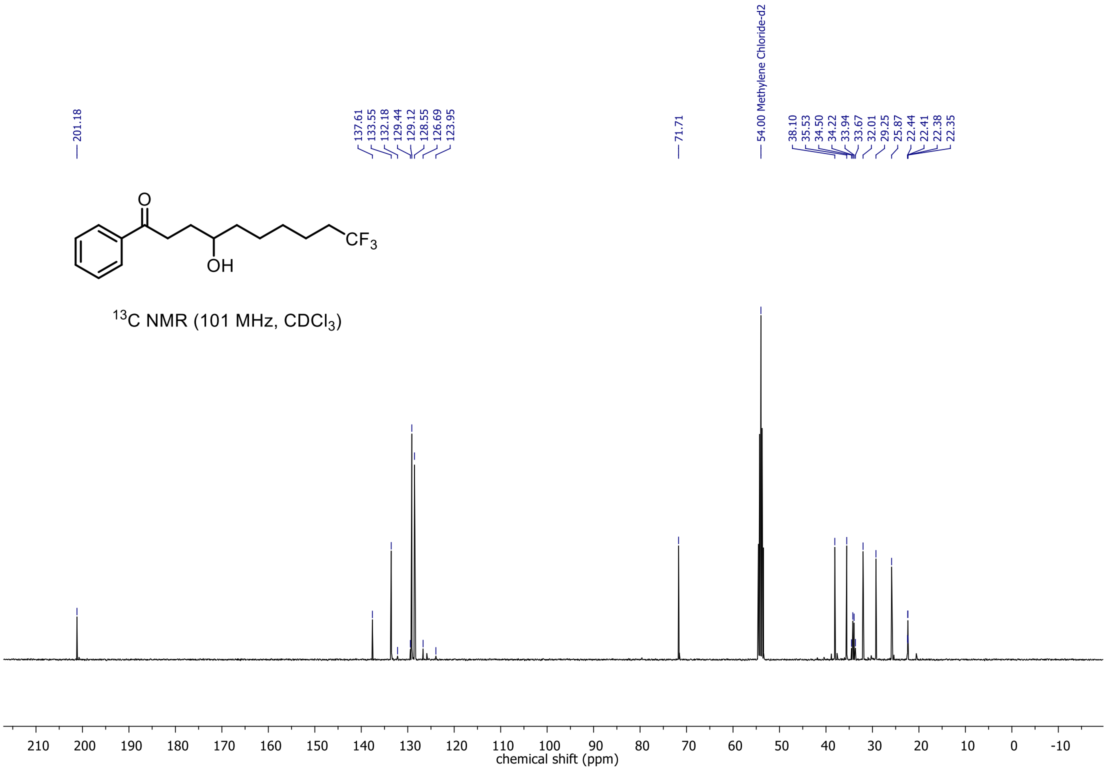

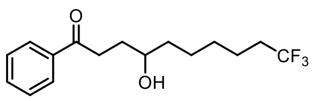

^{19}F NMR (377 MHz, $CDCl_3$)

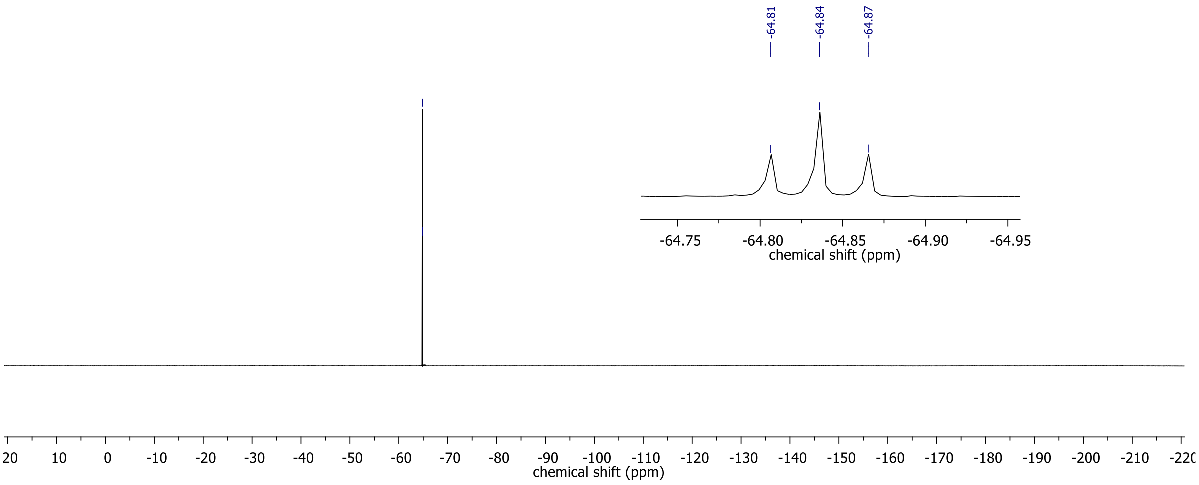

4-Hydroxy-1,16-diphenylhexadecane-1,16-dione (2g)

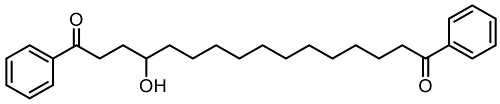

The title compound was prepared following general procedure G using 1,16-diphenylhexadec-8-ene-1,16-dione (37.7 mg, 0.10 mmol, 1.00 equiv.) and TfOH (27 µL, 0.30 mmol, 3.00 equiv.). Purification by flash column chromatography (0 – 100% EtOAc in heptanes) afforded the title compound (20.4 mg, 52 µmol, 52%) as orange solid and single regioisomer.

Crude product: 57% NMR yield, r.r. (γ_{OH}:δ_{OH}) = 93:7.

^{1}H NMR (400 MHz, CD_2Cl_2): δ 8.01 – 7.91 (m, 4H), 7.62 – 7.53 (m, 2H), 7.51 – 7.43 (m, 4H), 3.70 – 3.57 (m, 1H), 3.22 – 3.05 (m, 2H), 3.00 – 2.92 (m, 2H), 1.94 (m, 1H), 1.81 – 1.67 (m, 4H), 1.60 (s, 1H), 1.51 – 1.42 (m, 3H), 1.36 – 1.27 (m, *J* = 21.5 Hz, 14H).

^{13}C NMR (101 MHz, CD_2Cl_2): δ 201.2, 200.9, 137.8, 137.7, 133.5, 133.3, 129.10 (2C), 129.07 (2C), 128.6 (2C), 128.5 (2C), 71.9, 39.1, 38.4, 35.6, 32.0, 30.24, 30.18, 30.16 (2C), 30.07, 30.06, 29.9, 26.3, 24.9.

IR (neat) ν_{max}: 3473, 2921, 2849, 2360, 1720, 1682, 1596, 1365, 1207, 1071, 1001, 954, 736, 687.

HRMS (ESI$^+$): exact mass calculated for $[M+Na]^+$ $(C_{28}H_{38}O_3Na)^+$ requires *m/z* 445.2713, found *m/z* 445.2701.

4-Hydroxy-1,16-diphenylhexadecane-1,16-dione (2g)

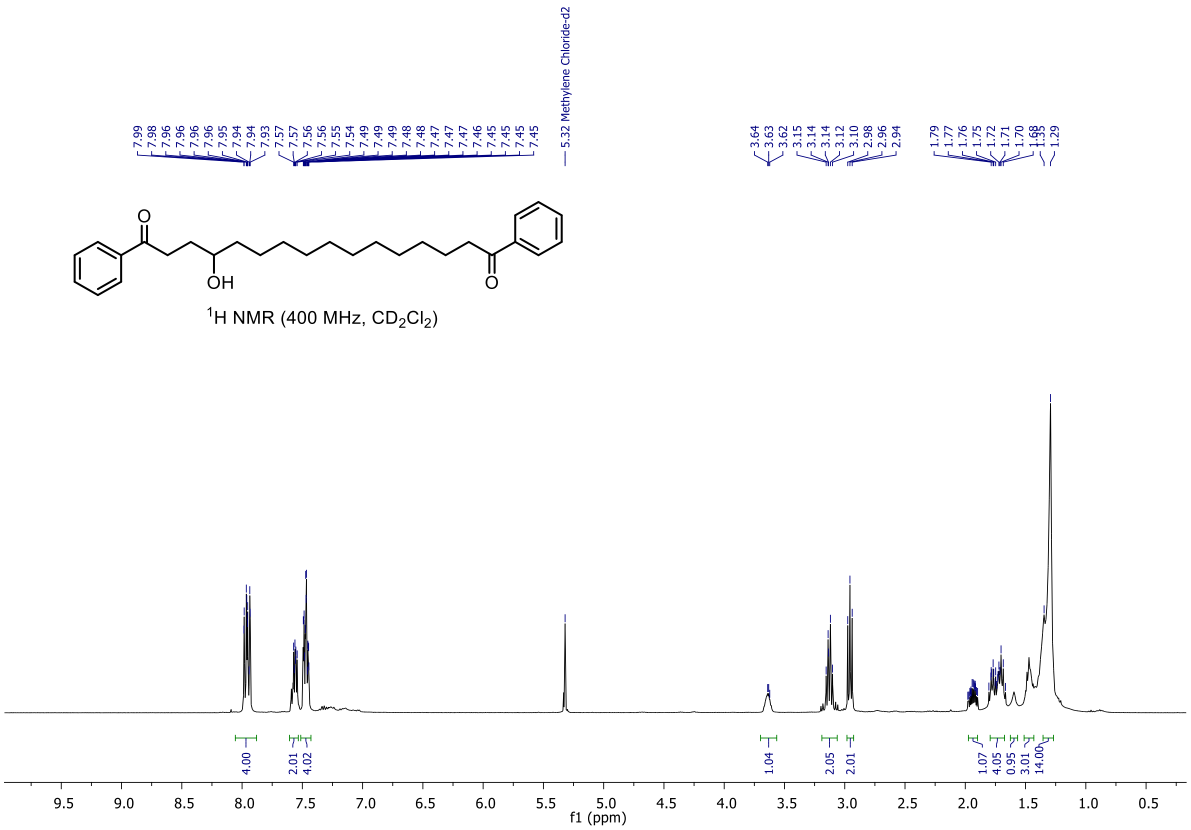

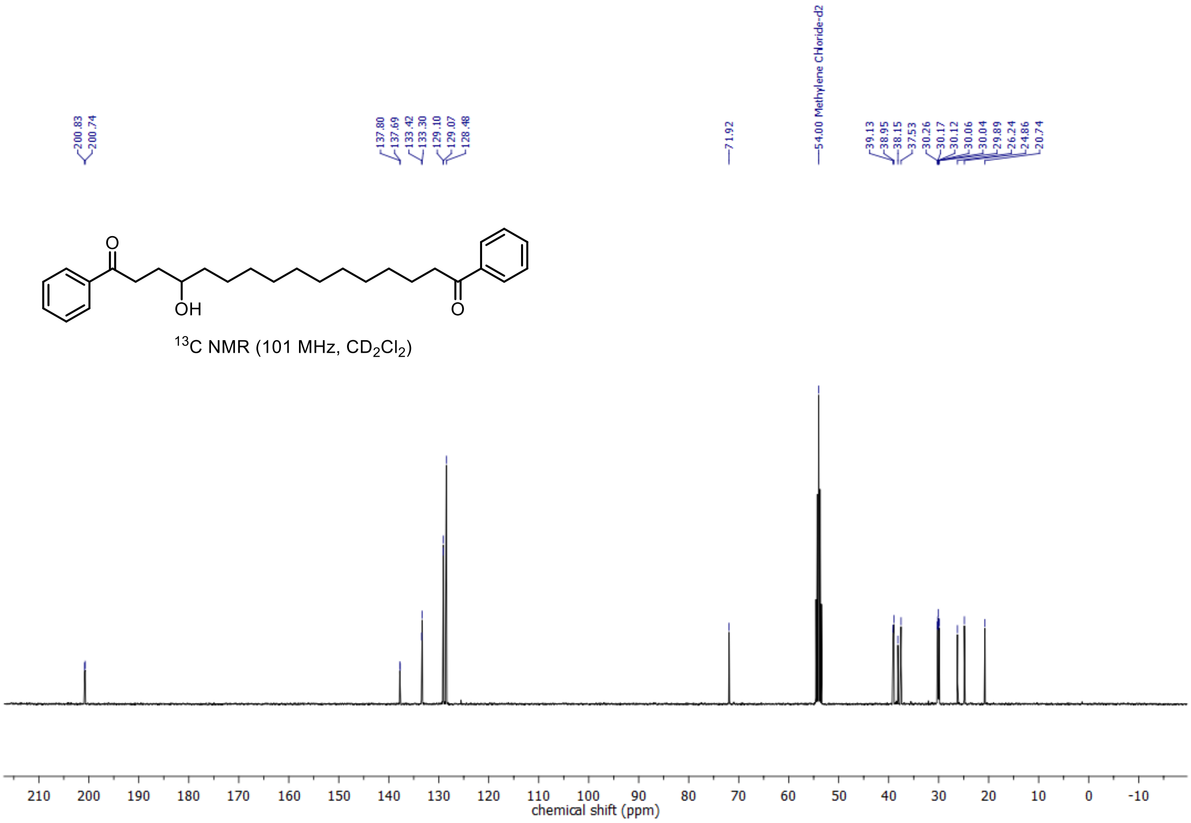

4-Hydroxy-1-phenyloctadecan-1-one (2h)

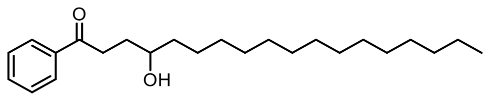

The title compound was prepared following general procedure H using (Z)-1-Phenyloctadec-9-en-1-one (34.3 mg, 0.10 mmol, 1.00 equiv.), TfOH (53 µL, 0.60 mmol, 6.00 equiv.) and NEt_3 (42 µL, 0.30 mmol, 3.00 equiv.). Purification by flash column chromatography (2 – 30% EtOAc in heptanes with 1% triethylamine additive) afforded the title compound (17.7 mg, 49 µmol, 49%) as a colorless solid and a single regioisomer.

Crude product: 54% NMR yield, r.r. (γ_{OH}:δ_{OH}) > 95:5

The acidic OH proton is not reported due to fast exchange processes.

^{1}H NMR (400 MHz, CD_2Cl_2) δ 8.01 – 7.94 (m, 2H), 7.61 – 7.53 (m, 1H), 7.52 – 7.43 (m, 2H), 3.63 (br s, 1H), 3.24 – 3.00 (m, 2H), 2.00 – 1.89 (m, 1H), 1.81 – 1.70 (m, 2H), 1.51 – 1.40 (m, 3H), 1.34 – 1.23 (m, 22H), 0.88 (t, *J* = 6.8 Hz, 3H).

One $^{13}CH_2$ resonance is not reported due to overlapping signals (see spectrum below).

^{13}C NMR (176 MHz, CD_2Cl_2) δ 201.0 (C), 137.5 (C), 133.3 (CH), 128.9 (2CH), 128.4 (2CH), 71.7 (CH), 38.3 (CH_2), 35.4 (CH_2), 32.3 (CH_2), 31.9 (CH_2), 30.10 ($3CH_2$), 30.09 (CH_2), 30.07 (CH_2), 30.06 (CH_2), 30.05 (CH_2), 29.8 (CH_2), 26.1 (CH_2), 23.1 (CH_2), 14.3 (CH_3).

IR (neat) ν_{max}: 3373, 2915, 2848, 1685, 1464, 1446, 738, 687.

HRMS (ESI$^+$): exact mass calculated for $[M+Na]^+$ $(C_{24}H_{40}O_2Na)^+$ requires *m*/*z* 383.2921, found *m*/*z* 383.2914.

4-Hydroxy-1-phenyloctadecan-1-one (2h)

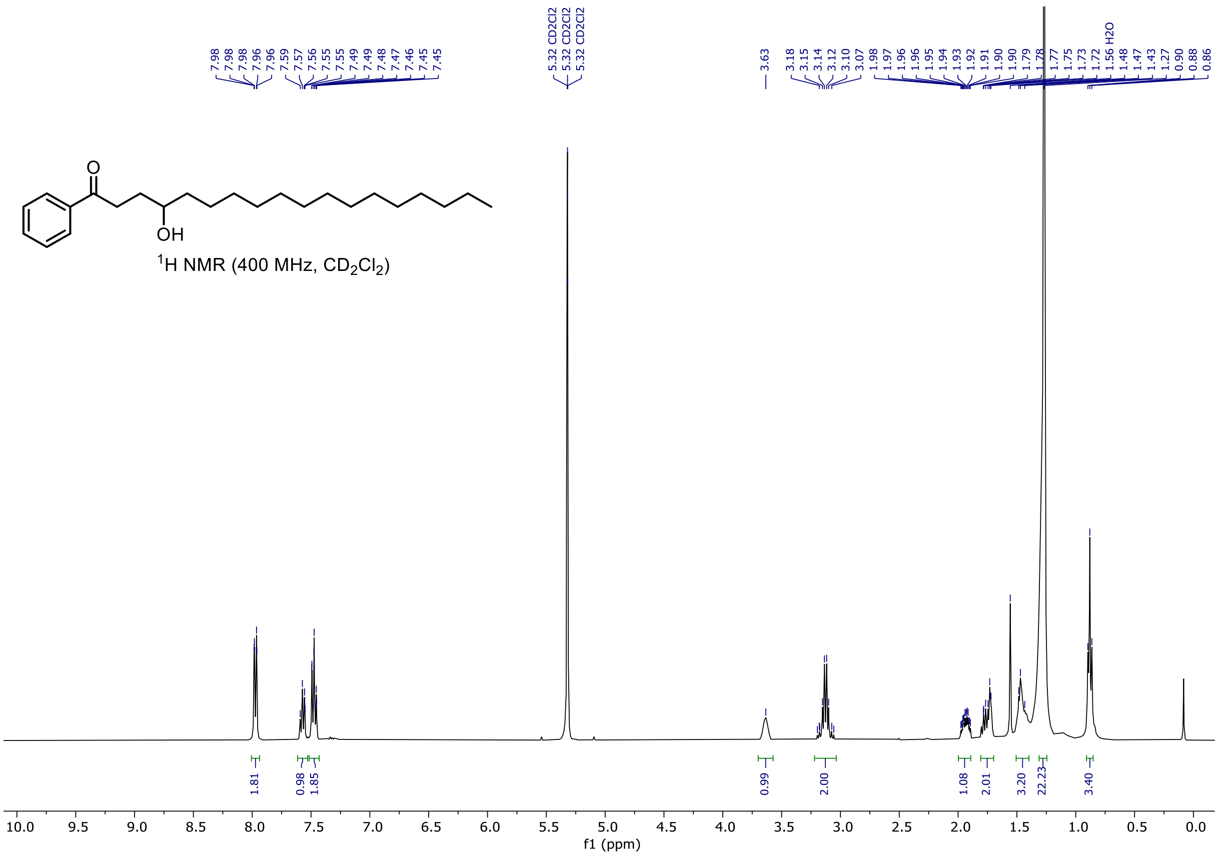

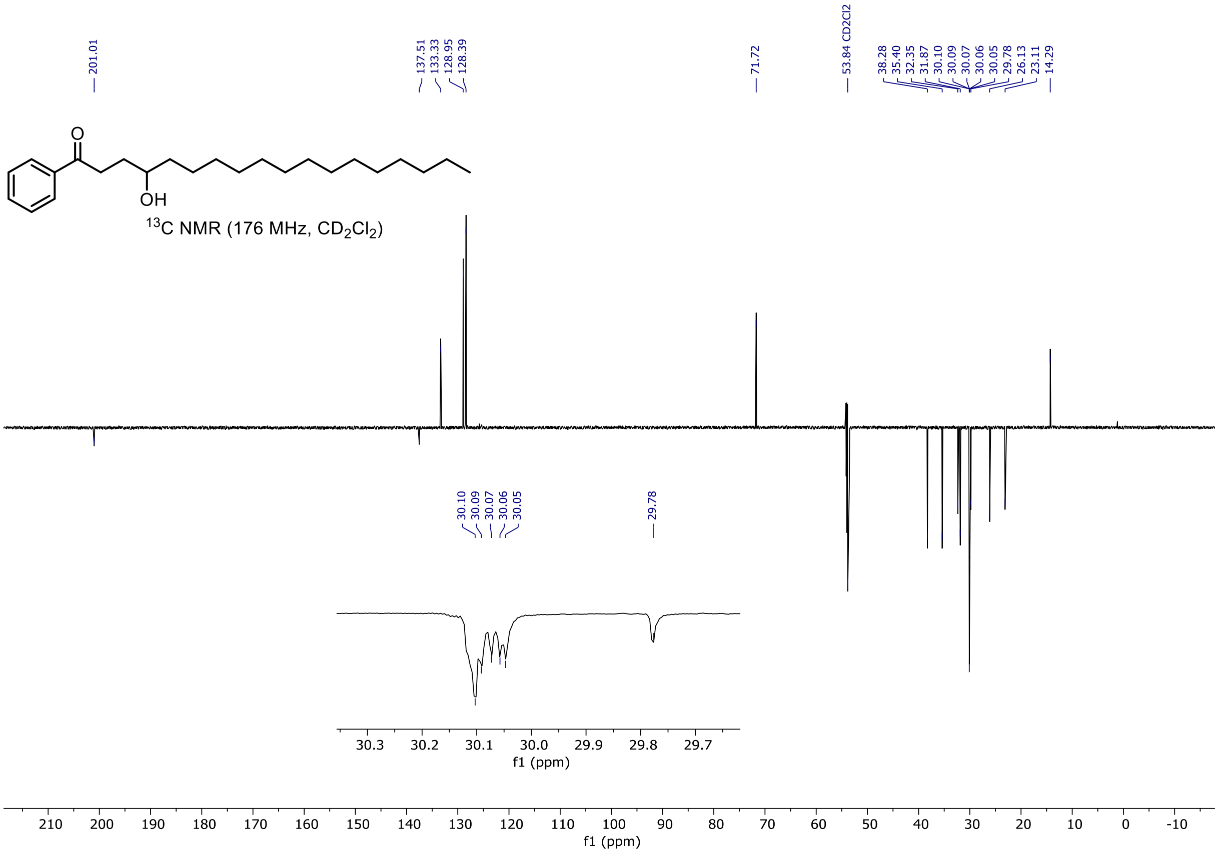

4-Hydroxy-1-(*o*-tolyl)nonan-1-one (2i)

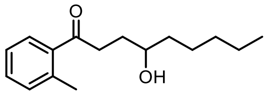

The title compound was prepared following general procedure G using 1-(*o*-tolyl)non-8-en-1-one (23 mg, 0.10 mmol, 1.00 equiv.) and TfOH (27 µL, 0.30 mmol, 3.00 equiv.). Purification by flash column chromatography (0 – 100% EtOAc in heptanes) afforded the title compound (16.6 mg, 67 µmol, 67%) as a yellowish oil and single regioisomer.

Crude product: 74% NMR yield, r.r. (γ_{OH}:δ_{OH}) > 95:5.

^{1}H NMR (400 MHz, $(CD_3)_2CO$): δ 7.77 – 7.72 (m, 1H), 7.39 (td, *J* = 7.5, 1.3 Hz, 1H), 7.34 – 7.25 (m, 2H), 3.65 – 3.55 (app br s, 1H), 3.50 (d, *J* = 5.5 Hz, 1H), 3.09 – 3.00 (m, 2H), 2.43 (s, 3H), 1.93 – 1.82 (m, 1H), 1.74 – 1.61 (m, 1H), 1.51 – 1.41 (m, 3H), 1.37 – 1.25 (m, 5H), 0.88 (t, *J* = 6.9 Hz, 3H).

^{13}C NMR (101 MHz, $(CD_3)_2CO$): δ 205.0, 139.8, 138.0, 132.4, 131.7, 129.2, 126.6, 70.9, 38.7, 38.6, 32.8, 32.7, 26.2, 23.4, 21.1, 14.4.

IR (neat) ν_{max}: 3433, 2927, 2857, 2359, 1681, 1600, 1571, 1455, 1379, 1256, 1126, 1034, 970, 751, 654, 458.

HRMS (ESI$^+$): exact mass calculated for $[M+Na]^+$ $(C_{16}H_{24}O_2Na)^+$ requires *m*/*z* 271.1669, found *m*/*z* 271.1661.

4-Hydroxy-1-(*o*-tolyl)nonan-1-one (2i)

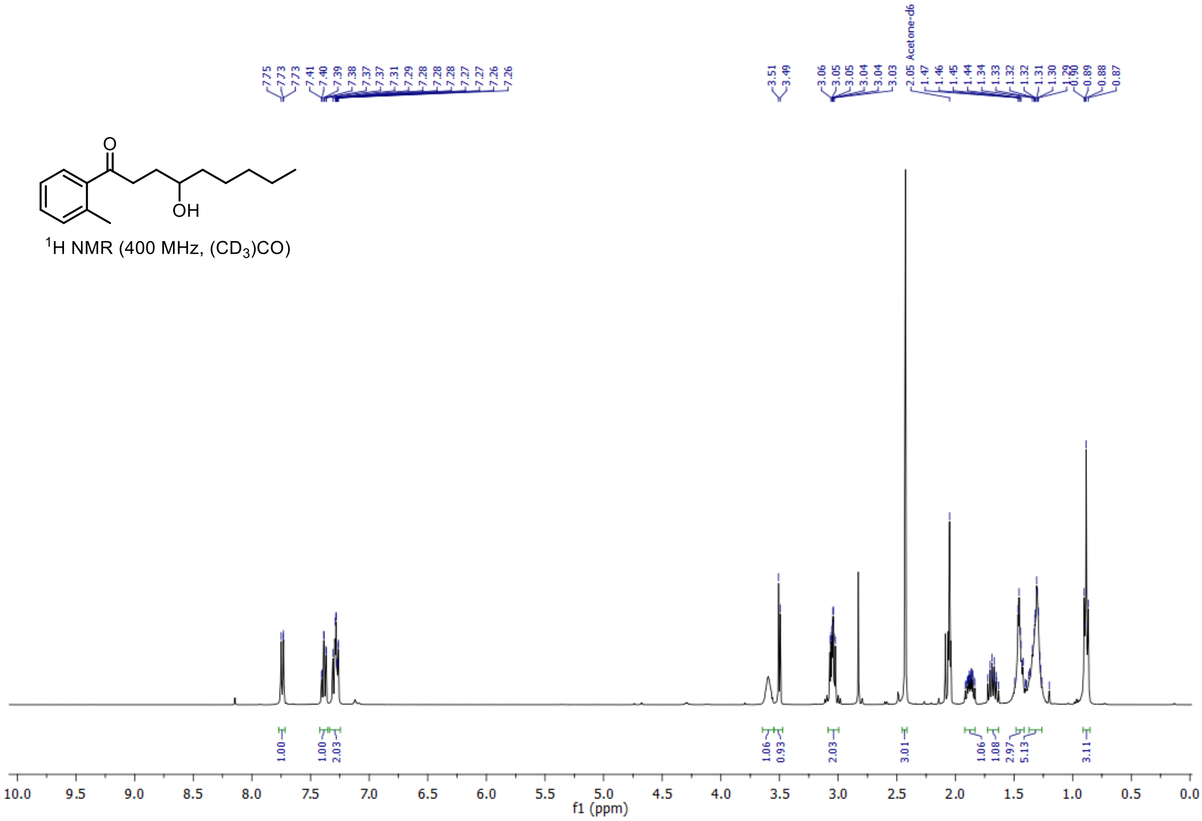

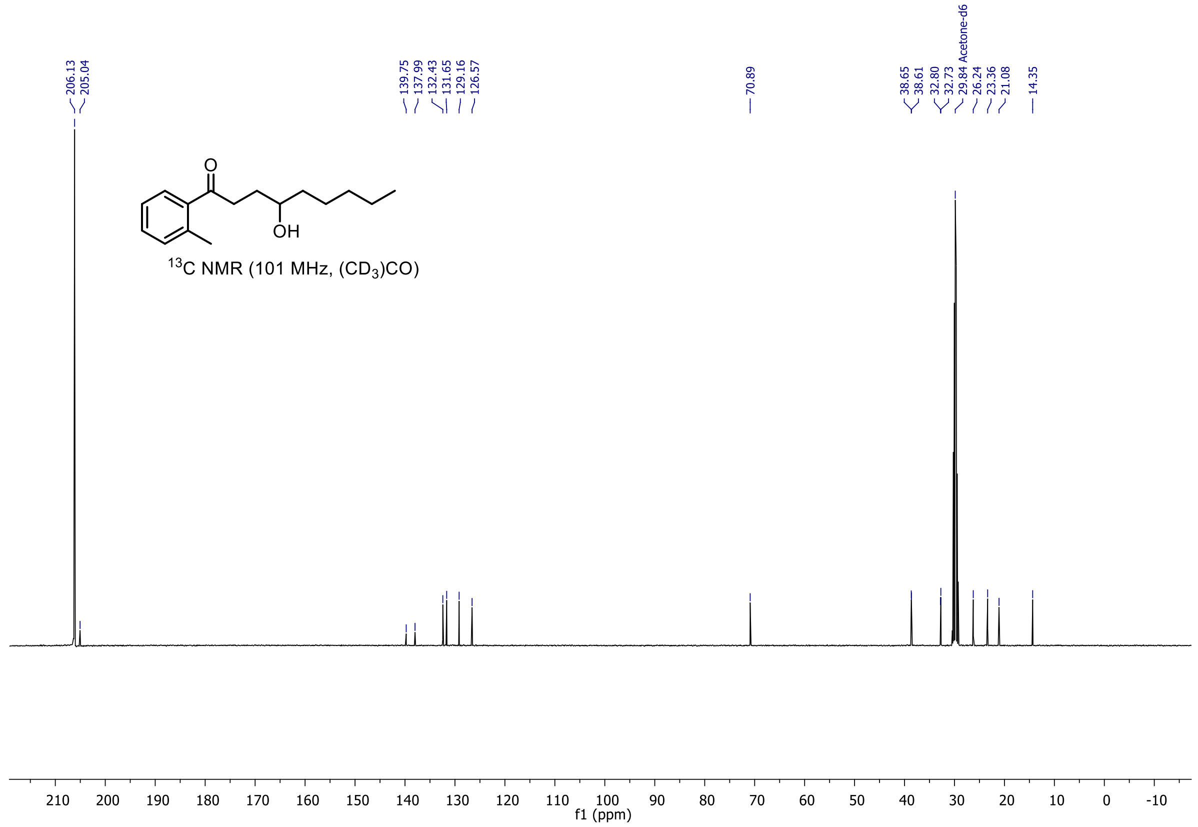

1-(4-(*tert*-Butyl)phenyl)-4-hydroxynonan-1-one (2j)

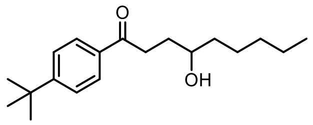

The title compound was prepared following general procedure G using 1-(4-(tert-butyl)phenyl)non-8-en-1-one (27.2 mg, 0.10 mmol, 1.00 equiv.) and TfOH (27 µL, 0.30 mmol, 3.00 equiv.). Purification by flash column chromatography (0 – 70% EtOAc in heptanes) afforded the title compound (21.0 mg, 72 µmol, 72%) as a yellow oil and a single regioisomer.

Crude product: 83% NMR yield, r.r. (γ_{OH}:δ_{OH}) > 95:5

^{1}H NMR (400 MHz, $CDCl_3$): δ 7.95 – 7.90 (m, 2H), 7.50 – 7.44 (m, 2H), 3.67 (s, 1H), 3.20 – 3.05 (m, 2H), 2.03 – 1.92 (m, 1H), 1.89 (app br s, 1H), 1.85 – 1.74 (m, 1H), 1.56 – 1.25 (m, 17H), 0.89 (t, *J* = 6.8 Hz, 3H).

^{13}C NMR (101 MHz, $CDCl_3$): δ 200.8, 157.0, 134.5, 128.2 (2C), 125.7 (2C), 71.7, 38.0, 35.3, 35.0, 32.0, 31.6, 31.2 (3C), 25.5, 22.8, 14.2.

IR (neat) ν_{max}: 2956, 2929, 1717, 1681, 1606, 1406, 1363, 1269, 1189, 1107, 1047, 1016, 835.

HRMS (ESI$^+$): exact mass calculated for $[M+Na]^+$ $(C_{19}H_{30}O_2Na)^+$ requires *m/z* 313.2138, found *m/z* 313.2139.

1-(4-(*tert*-Butyl)phenyl)-4-hydroxynonan-1-one (2j)

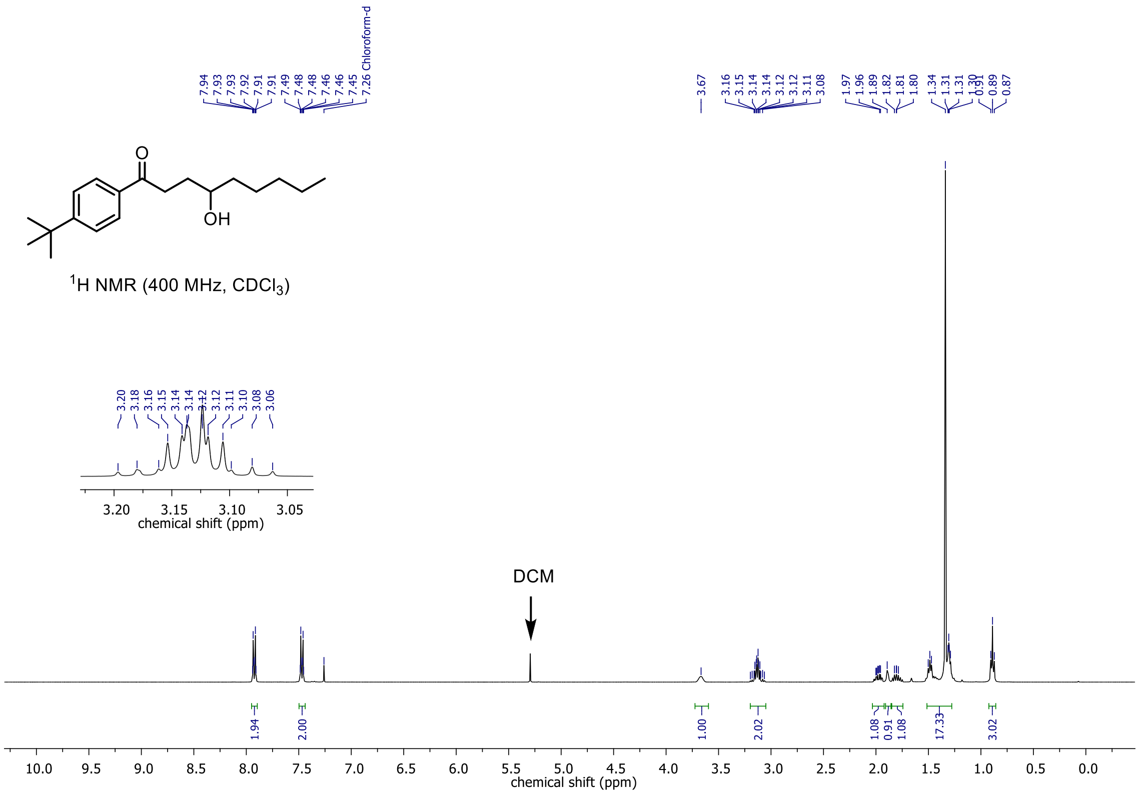

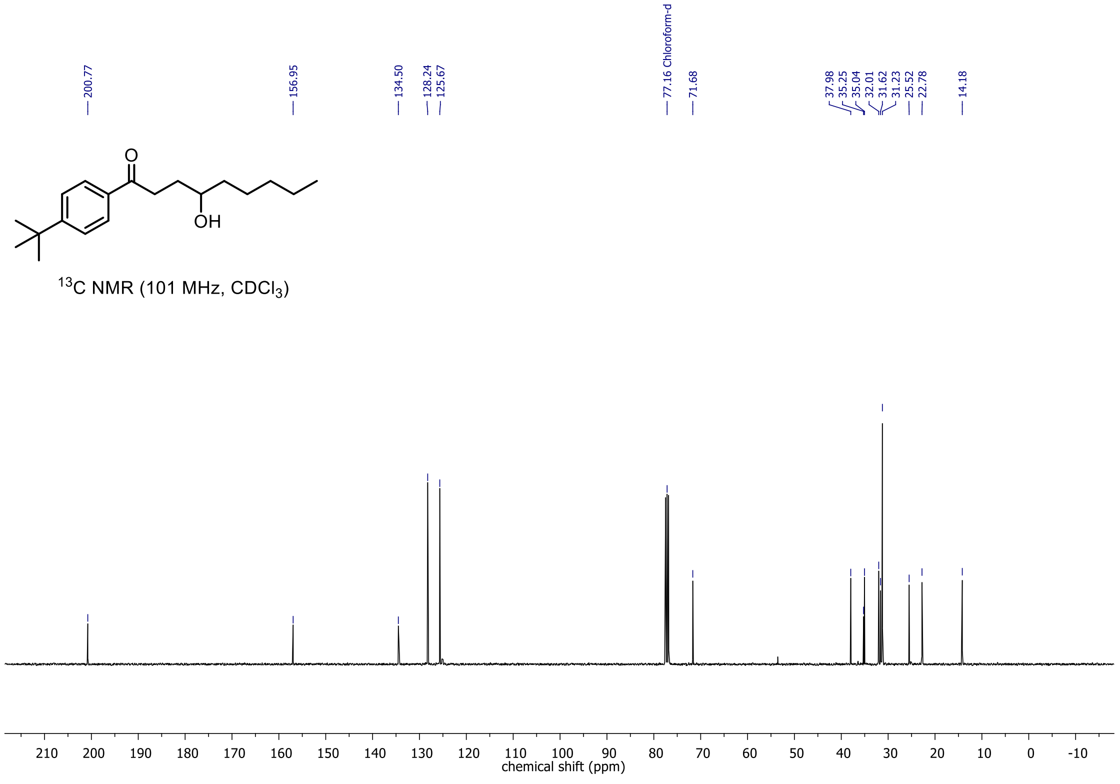

4-Hydroxy-1-(naphthalen-2-yl)heptan-1-one (2k)

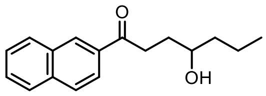

The title compound was prepared following general procedure G using 1-(naphthalen-2-yl)hept-6-en-1-one (23.8 mg, 0.10 mmol, 1.00 equiv.) and TfOH (27 µL, 0.30 mmol, 3.00 equiv.). Purification by flash column chromatography (0 – 70% EtOAc in heptanes) afforded the title compound (22.7 mg, 89 µmol, 89%) as a yellow oil and a single regioisomer.

Crude product: 90% NMR yield, r.r. (γ_{OH}:δ_{OH}) > 95:5.

^{1}H NMR (400 MHz, CD_2Cl_2): δ 8.53 (s, 1H), 8.03 – 7.86 (m, 4H), 7.64 – 7.55 (m, 2H), 3.70 (s, 1H), 3.37 – 3.13 (m, 2H), 2.07 – 1.95 (m, 1H), 1.87 – 1.72 (m, 2H), 1.52 – 1.36 (m, 4H), 0.95 (t, *J* = 6.5 Hz, 3H).

^{13}C NMR (101 MHz, CD_2Cl_2): δ 201.1, 136.1, 135.0, 133.2, 130.2, 130.1, 129.0, 128.9, 128.3, 127.3, 124.4, 71.6, 40.6, 35.6, 32.2, 19.5, 14.4.

IR (neat) ν_{max}: 3465, 2957, 2931, 2871, 1713, 1681, 1466, 1355, 1282, 1228, 1197, 1129, 1092, 780.

HRMS (ESI$^+$): exact mass calculated for $[M+Na]^+$ $(C_{17}H_{20}O_2Na)^+$ requires *m/z* 279.1356, found *m/z* 279.1355.

4-Hydroxy-1-(naphthalen-2-yl)heptan-1-one (2k)

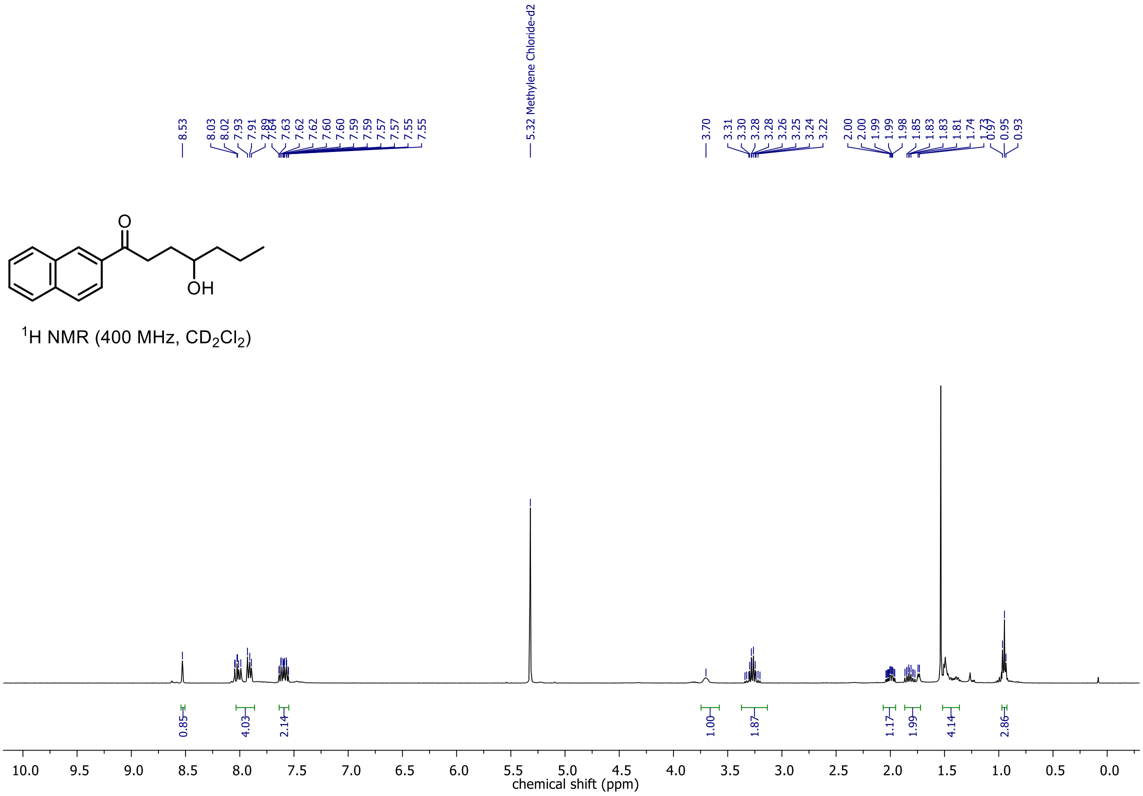

^{1}H NMR (400 MHz, CD_2Cl_2)

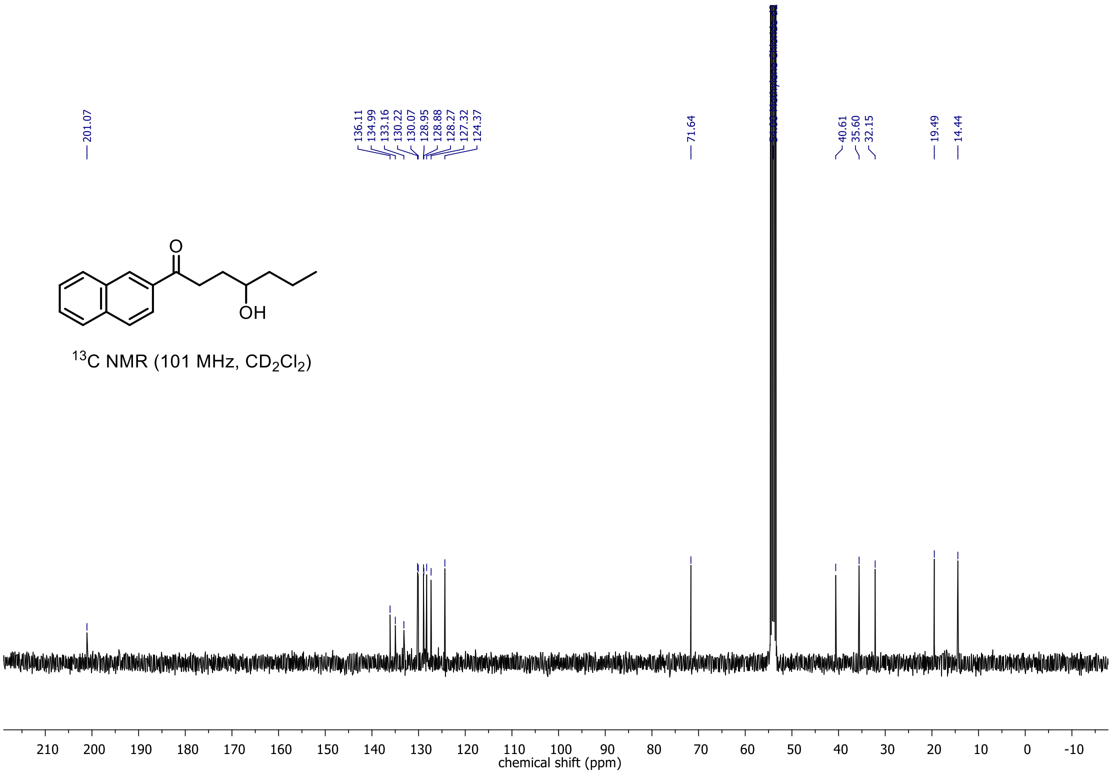

^{13}C NMR (101 MHz, CD_2Cl_2)

1-(4-Chlorophenyl)-4-hydroxyheptan-1-one (2l)

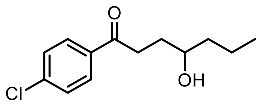

The title compound was prepared following general procedure G using 1-(4-chlorophenyl)hept-6-en-1-one (44.5 mg, 0.20 mmol, 1.00 equiv.) and TfOH (27 µL, 0.60 mmol, 3.00 equiv.). Purification by flash column chromatography (0 – 70% EtOAc in heptanes) afforded the title compound (28.6 mg, 0.12 mmol, 59%) as a white solid and a single regioisomer.

Crude product: 58% NMR yield, r.r. (γ_{OH}:δ_{OH}) > 95:5.

^{1}H NMR (400 MHz, CD_2Cl_2): δ 7.96 – 7.80 (m, 2H), 7.47 – 7.42 (m, 2H), 3.72 – 3.52 (m, 1H), 3.28 – 2.91 (m, 2H), 1.98 – 1.89 (m, 1H), 1.82 (s, 1H), 1.78 – 1.69 (m, 1H), 1.50 – 1.34 (m, 4H), 0.95 – 0.90 (m, 3H).

^{13}C NMR (101 MHz, CD_2Cl_2): δ 200.0, 139.7, 136.0, 130.1 (2C), 129.4 (2C), 71.5, 40.6, 35.5, 31.9, 19.5, 14.4.

IR (neat) ν_{max}: 3451, 2957, 2931, 2872, 1719, 1686, 1591, 1572, 1488, 1271, 1091, 1013, 830.

HRMS (ESI$^+$): exact mass calculated for $[M+Na]^+$ $(C_{13}H_{17}ClO_2Na)^+$ requires *m/z* 263.0809, found *m/z* 263.0807.

1-(4-Chlorophenyl)-4-hydroxyheptan-1-one (2l)

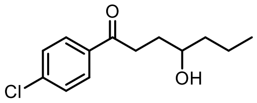

^1H NMR (400 MHz, CD_2Cl_2)

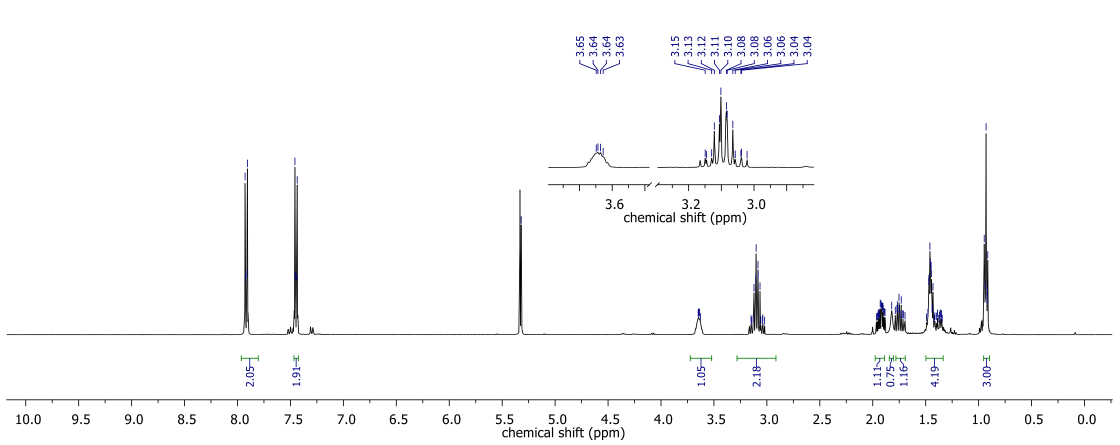

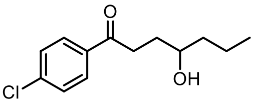

^{13}C NMR (101 MHz, CD_2Cl_2)

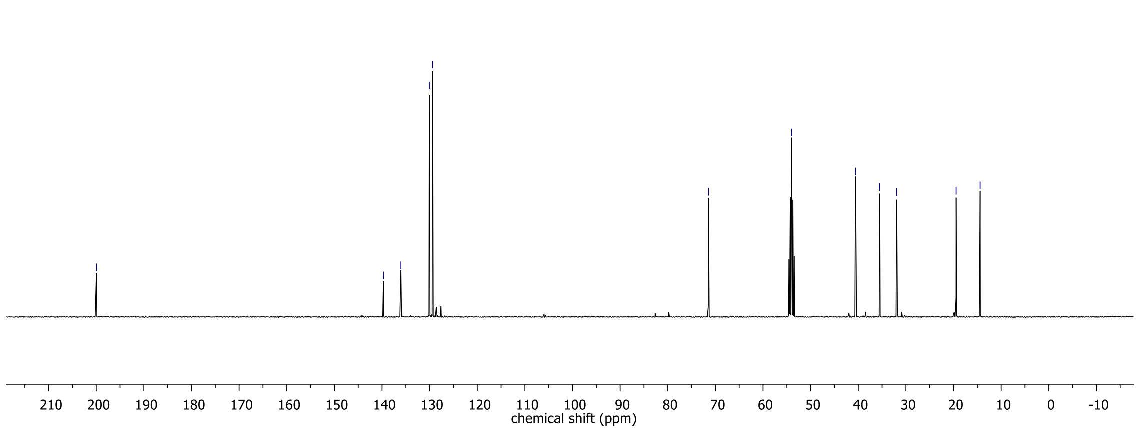

1-(3-Fluorophenyl)-4-hydroxynonan-1-one (2m)

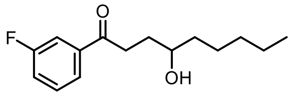

The title compound was prepared following general procedure G with a modification to the reaction temperature (100 °C instead of 120 °C) using 1-(3-fluorophenyl)non-8-en-1-one (23.4 mg, 0.10 mmol, 1.00 equiv.) and TfOH (27 µL, 0.30 mmol, 3.00 equiv.). Purification by flash column chromatography (0 – 100% EtOAc in heptanes) afforded the title compound (14.5 mg, 58 µmol, 58%) as a yellow solid and single regioisomer.

Crude product: 72% NMR Yield, r.r. (γ_{OH}:δ_{OH}) = 88:12.

^{1}H NMR (400 MHz, $(CD_3)_2CO$): δ 7.78 – 7.67 (m, 1H), 7.57 (ddd, *J* = 9.8, 2.6, 1.5 Hz, 1H), 7.44 (td, *J* = 8.0, 5.6 Hz, 1H), 7.26 (tdd, *J* = 8.4, 2.7, 0.9 Hz, 1H), 3.54 – 3.45 (m, 1H), 3.40 (d, *J* = 5.5 Hz, 1H), 3.11 – 2.96 (m, 2H), 1.82 – 1.72 (m, 1H), 1.64 – 1.51 (m, 1H), 1.39 – 1.31 (m, 3H), 1.25 – 1.15 (m, 5H), 0.75 (t, *J* = 6.9 Hz, 3H).

^{13}C NMR (101 MHz, $(CD_3)_2CO$): δ 199.6, 163.7 (d, *J* = 245.4 Hz), 140.5 (d, *J* = 5.9 Hz), 131.5 (d, *J* = 7.9 Hz), 124.8 (d, *J* = 2.9 Hz), 120.3 (d, *J* = 21.4 Hz), 115.1 (d, *J* = 22.3 Hz), 70.8, 38.6, 35.7, 32.7, 32.6, 26.2, 23.4, 14.3.

^{19}F (376 MHz, $(CD_3)_2CO$): -108.81.

IR (neat) ν_{max}: 3429, 2928, 2858, 1686, 1588, 1442, 1254, 1126, 886, 785, 681.

HRMS (ESI$^+$): exact mass calculated for $[M+Na]^+$ $(C_{15}H_{21}FO_2Na)^+$ requires *m/z* 275.1418, found *m/z* 275.1416.

1-(3-Fluorophenyl)-4-hydroxynonan-1-one (2m)

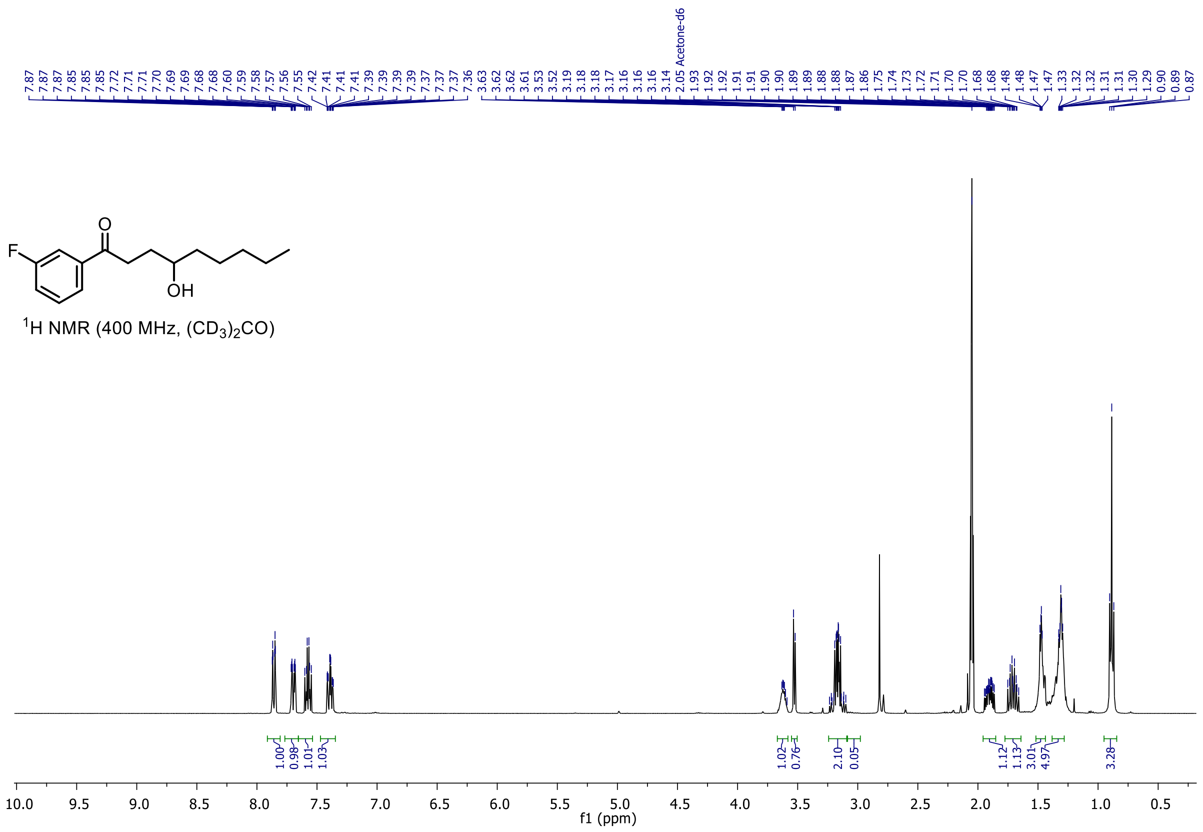

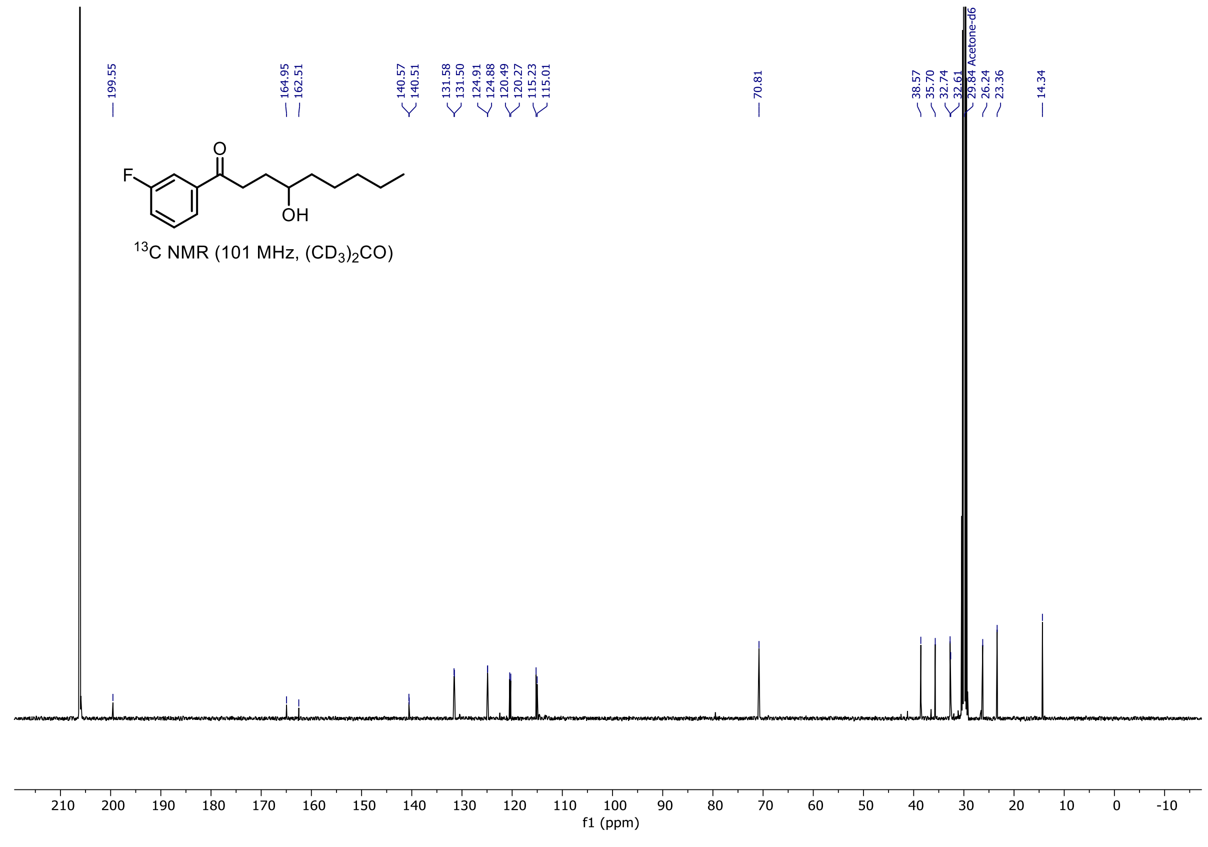

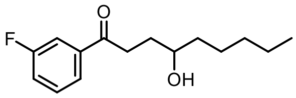

^{13}C NMR (376 MHz, $(CD_3)_2CO$)

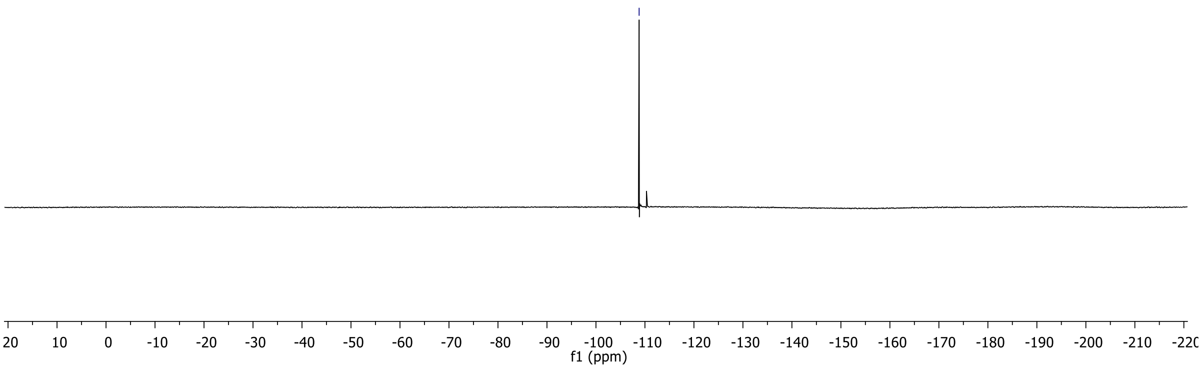

4-Hydroxy-1-(4-(trifluoromethoxy)phenyl)nonan-1-one (2n)

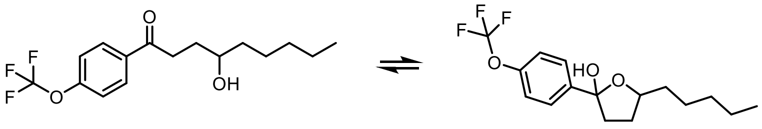

12.1 : 1
(2 diastereomers)

The title compound was prepared following general procedure G using 1-(4-(trifluoromethoxy)phenyl)non-8-en-1-one (25.9 mg, 0.10 mmol, 1.00 equiv.) and TfOH (27 µL, 0.30 mmol, 3.00 equiv.). The reaction was carried out in DCM (1 mL) and in a microwave (50 °C for 3 h, max. power = 100 W), the workup procedure was identical to general procedure G. Purification by flash column chromatography (6 – 30% EtOAc in heptanes with 1% triethylamine additive) afforded the title compound (19.7 mg, 62 µmol, 62%) as a colorless oil and a mixture of two regioisomers (γ:δ = 95:5). The γ-hydroxy ketone is in equilibrium with its hemiacetal form (ratio = 12.1:1, two diastereomers in a ratio of 1.3:1).

Crude product: 78% NMR yield, r.r. ($\gamma_{OH+Hemiacetals}$:δ_{OH}) = 93:7

An asterisk (*) denotes signals that unambiguously arise from the hemiacetal (2 diastereomers). A full list of signals arising from the minor δ-regioisomer can be found in Section 2.4.

^{1}H NMR (600 MHz, CD_2Cl_2) δ 8.06 – 7.99 (m, 1.8H), 7.63 – 7.57 (m, 0.2H*), 7.31 (dd, *J* = 8.8, 0.8 Hz, 1.8H), 7.21 – 7.17 (m, 0.2H*), 4.39 – 4.34 (m, 0.04H*), 4.28 – 4.22 (m, 0.03H*), 3.67 – 3.61 (m, 0.88H), 3.60 – 3.56 (m, 0.05H), 3.18 – 3.05 (m, 1.8H), 3.00 (t, *J* = 7.2 Hz, 0.1H), 2.64 (br s, 0.1H*), 2.34 – 2.22 (m, 0.1H), 2.18 – 2.10 (m, 0.1H*), 1.98 – 1.91 (m, 1H), 1.80 – 1.72 (m, 1H), 1.69 (d, *J* = 4.9 Hz, 0.8H), 1.51 – 1.40 (m, 3H), 1.37 – 1.26 (m, 5H), 0.93 – 0.88 (m, 3H).

Due to low abundance, only signals arising from the major component (γ-hydroxy ketone) are reported in ^{13}C NMR.

^{13}C NMR (151 MHz, CD_2Cl_2) δ 199.5 (C), 152.8 (q, *J* = 1.6 Hz, C), 135.9 (C), 130.5 (2CH), 120.82 (2CH), 120.77 (q, *J* = 258.4 Hz, C), 71.6 (CH), 38.3 (CH_2), 35.4 (CH_2), 32.28 (CH_2), 31.8 (CH_2), 25.8 (CH_2), 23.1 (CH_2), 14.2 (CH_3).

^{19}F NMR (565 MHz, CD_2Cl_2) δ -58.03 (3.00F), -58.25 (0.3F*).

IR (neat) ν_{max}: 3351, 2957, 2922, 2876, 2855, 1683, 1605, 1507, 1414, 1313, 1292, 1266, 1208, 1164.

HRMS (ESI$^+$): exact mass calculated for $[M+Na]^+$ $(C_{16}H_{21}F_3O_3Na)^+$ requires *m*/*z* 341.1335, found *m*/*z* 341.1322.

4-Hydroxy-1-(4-(trifluoromethoxy)phenyl)nonan-1-one (2n)

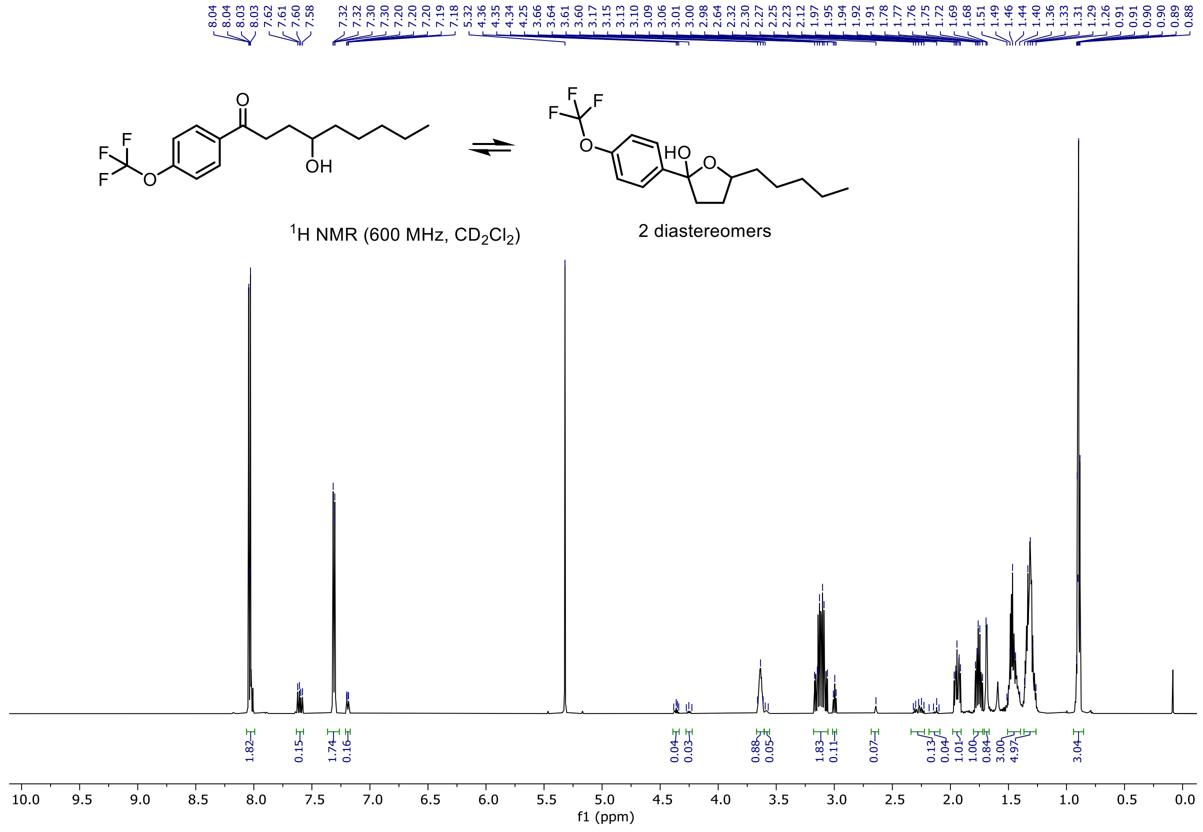

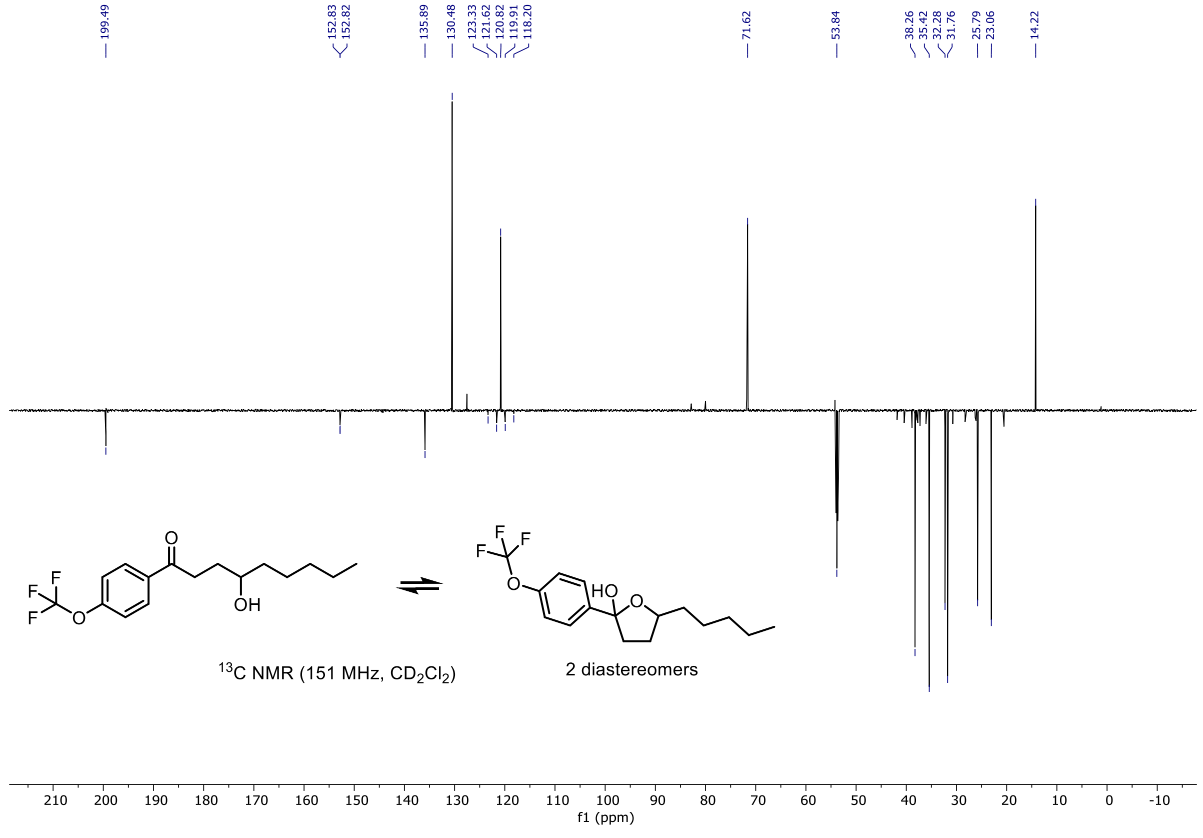

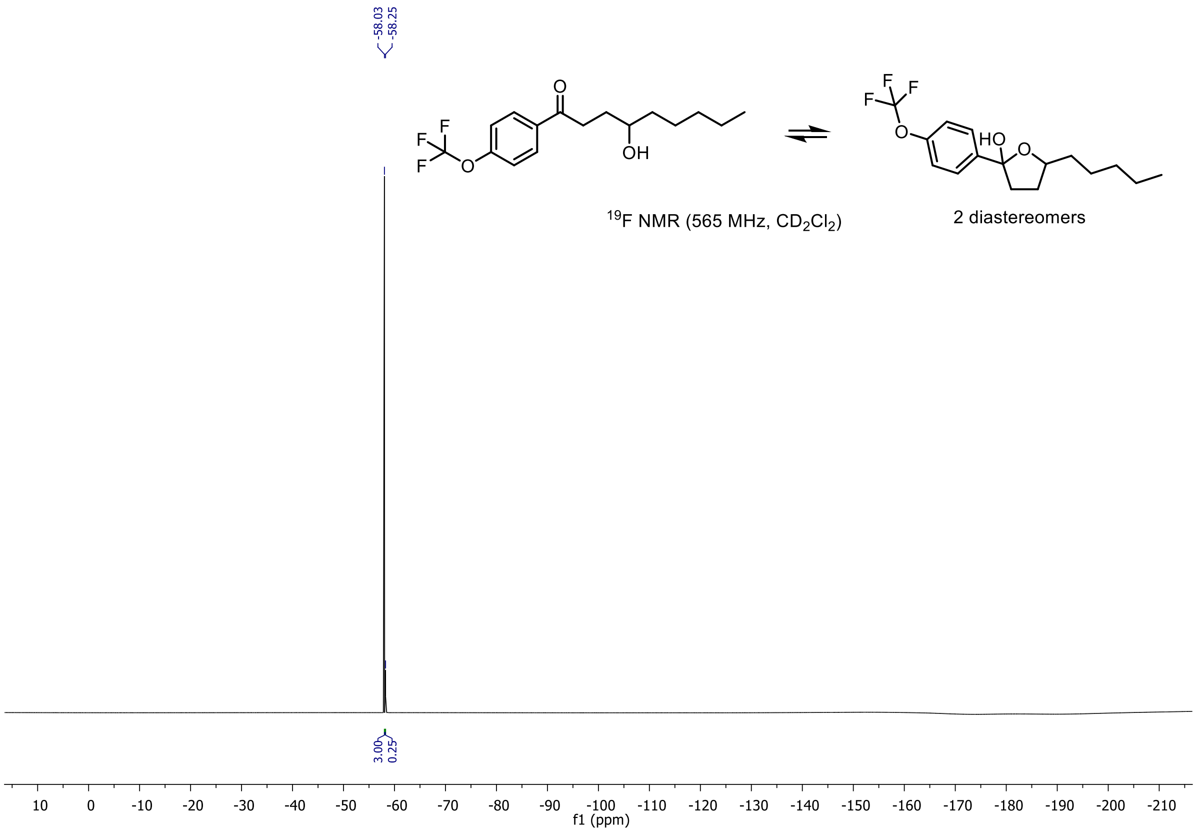
-58.03
-58.25
^{19}F NMR (565 MHz, CD_2Cl_2)
2 diastereomers
3.00
0.25
10 0 -10 -20 -30 -40 -50 -60 -70 -80 -90 -100 -110 -120 -130 -140 -150 -160 -170 -180 -190 -200 -210
f1 (ppm)

4-Hydroxy-1-(4-methoxyphenyl)decan-1-one (2o)

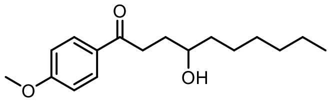

The title compound was prepared following general procedure G using 1-(4-methoxyphenyl)dec-9-en-1-one (26.0 mg, 0.10 mmol, 1.00 equiv.) and TfOH (27 µL, 0.30 mmol, 3.00 equiv.). Purification by flash column chromatography (10 – 40% EtOAc in heptanes) afforded the title compound (17.0 mg, 61 µmol, 61%) as a colorless solid and a single regioisomer.

Crude product: 70% NMR yield; r.r. (γ_{OH}:δ_{OH}) = 91:9

^{1}H NMR (400 MHz, CD_2Cl_2) δ 8.00 – 7.91 (m, 2H), 6.98 – 6.91 (m, 2H), 3.86 (s, 3H), 3.62 (app br s, 1H), 3.15 – 3.00 (m, 2H), 1.91 (dtd, *J* = 14.5, 7.3, 3.5 Hz, 2H), 1.80 – 1.69 (m, 1H), 1.50 – 1.26 (m, 10H), 0.88 (t, *J* = 6.8 Hz, 3H).

^{13}C NMR (101 MHz, CD_2Cl_2) δ 199.6 (C), 163.9 (C), 130.7 (2CH), 130.5 (C), 114.0 (2CH), 71.8 (CH), 55.9 (CH_3), 38.3 (CH_2), 35.1 (CH_2), 32.3 (CH_2), 32.0 (CH_2), 29.8 (CH_2), 26.1 (CH_2), 23.0 (CH_2), 14.3 (CH_3).

IR (neat) ν_{max}: 3413, 2954, 2927, 2856, 1600, 1510, 1253, 1167, 1100, 1028, 836.

HRMS (ESI$^+$): exact mass calculated for $[M+Na]^+$ $(C_{17}H_{26}O_3Na)^+$ requires *m*/*z* 301.1774, found *m*/*z* 301.1770.

4-Hydroxy-1-(4-methoxyphenyl)decan-1-one (2o)

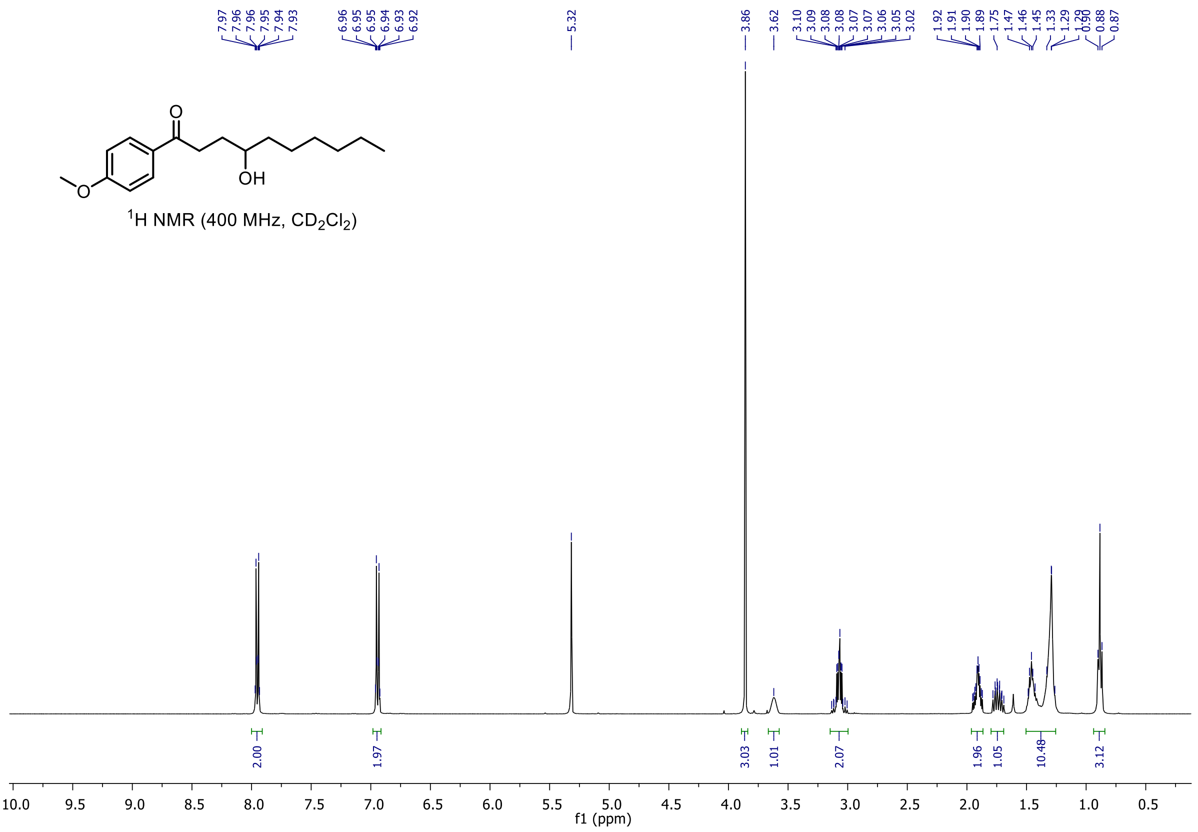

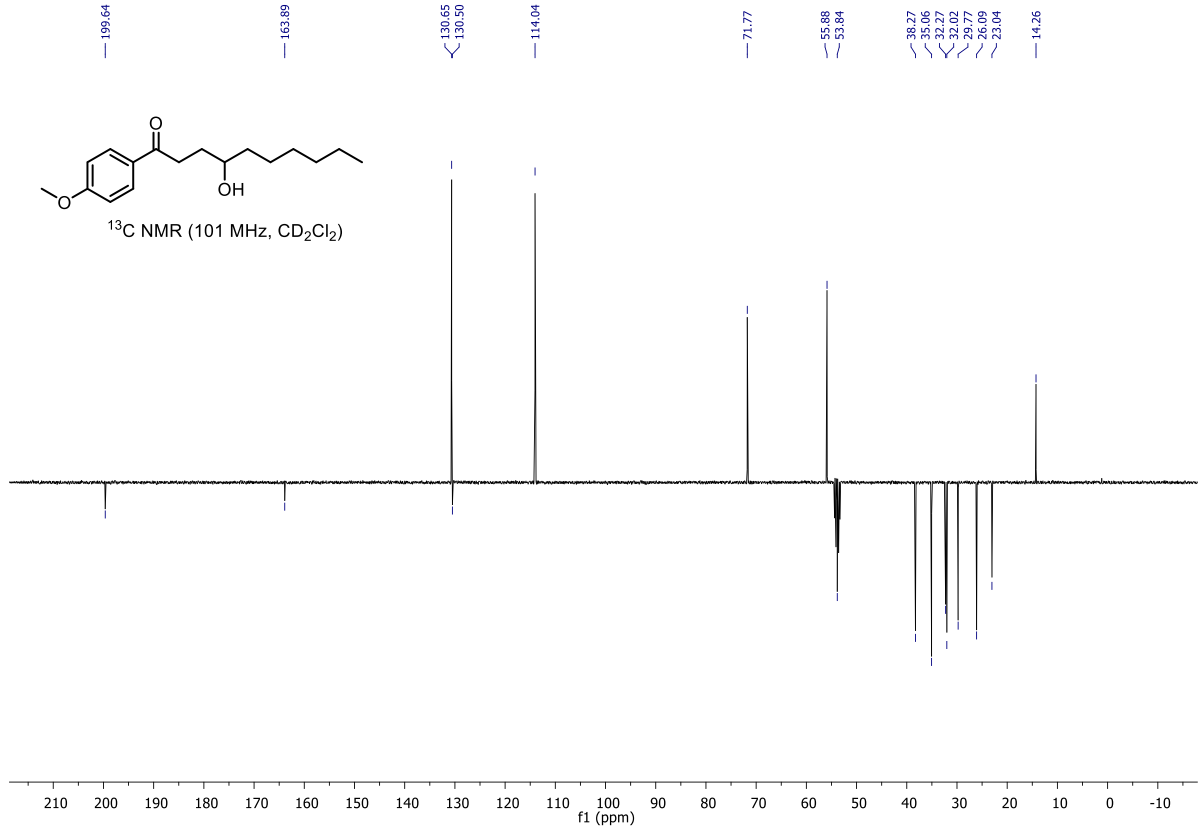

1-(4-(Dimethylamino)phenyl)-4-hydroxynonan-1-one (2p)

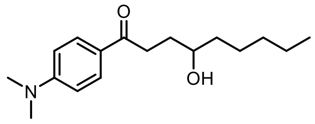

The title compound was prepared following general procedure G with a modification to the reaction temperature (80 °C instead of 120 °C) using 1-(4-(dimethylamino)phenyl)non-8-en-1-one (25.9 mg, 0.10 mmol, 1.00 equiv.) and TfOH (35 µL, 0.40 mmol, 4.00 equiv.). Purification by flash column chromatography (15 – 35% EtOAc in heptanes with 1% triethylamine additive) afforded the title compound (14.2 mg, 51 µmol, 51%) as a colorless solid and a single regioisomer.

Crude product: 52% NMR yield, r.r. (γ_{OH}:δ_{OH}) = 93:7

^{1}H NMR (400 MHz, CD_2Cl_2) δ 7.91 – 7.84 (m, 2H), 6.70 – 6.63 (m, 2H), 3.61 (app br s, 1H), 3.09 – 2.99 (m, 8H), 2.25 (br s, 1H), 1.89 (dtd, *J* = 14.3, 7.2, 3.5 Hz, 1H), 1.73 (ddt, *J* = 14.1, 8.4, 6.9 Hz, 1H), 1.49 – 1.40 (m, 3H), 1.38 – 1.25 (m, 5H), 0.89 (t, *J* = 6.9 Hz, 3H).

^{13}C NMR (101 MHz, CD_2Cl_2) δ 199.3 (C), 153.9 (C), 130.5 (2CH), 125.2 (C), 111.0 (2CH), 71.9 (CH), 40.2 (2CH_3), 38.2 (CH_2), 34.7 (CH_2), 32.3 (CH_2), 32.3 (CH_2), 25.8 (CH_2), 23.1 (CH_2), 14.2 (CH_3).

IR (neat) ν_{max}: 3419, 2925, 2856, 1656, 1595, 1371, 1188, 1169, 1126, 1065, 820.

HRMS (ESI$^+$): exact mass calculated for $[M+H]^+$ $(C_{17}H_{28}NO_2)^+$ requires *m*/*z* 278.2115, found *m*/*z* 278.2109.

1-(4-(Dimethylamino)phenyl)-4-hydroxynonan-1-one (2p)

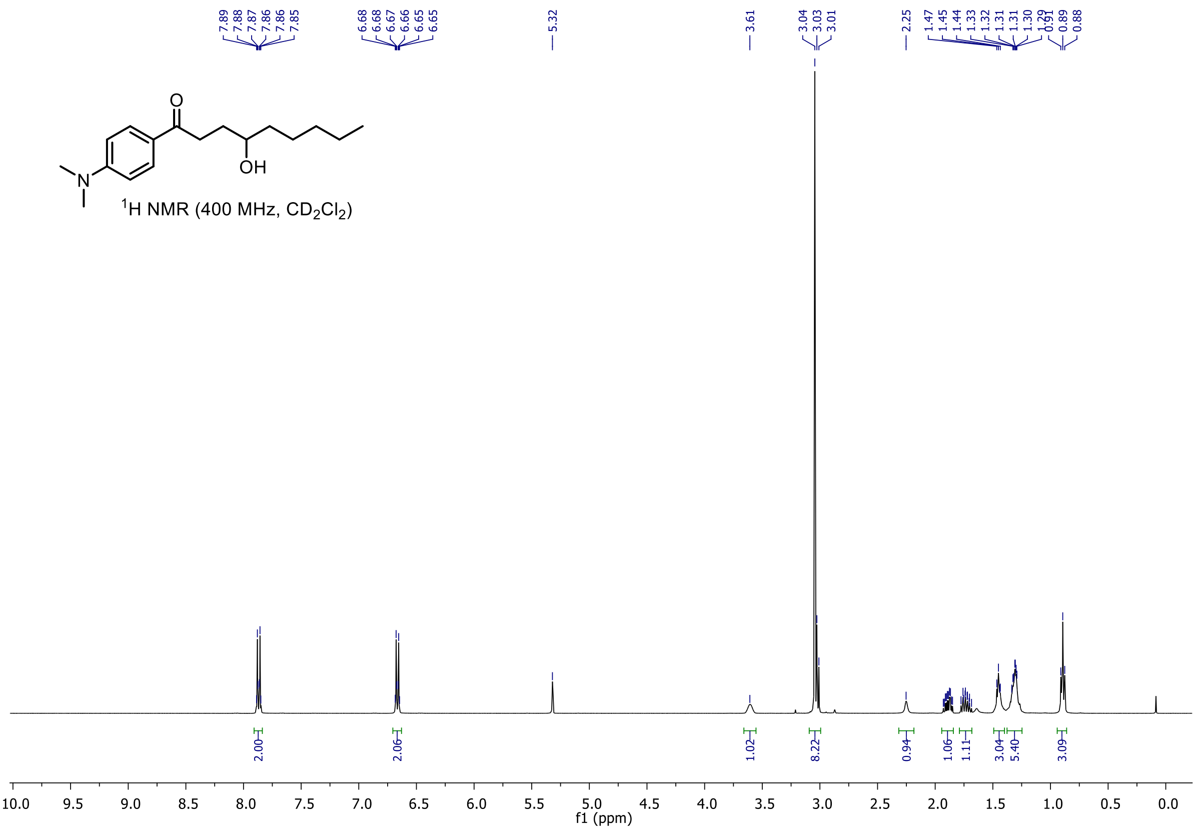

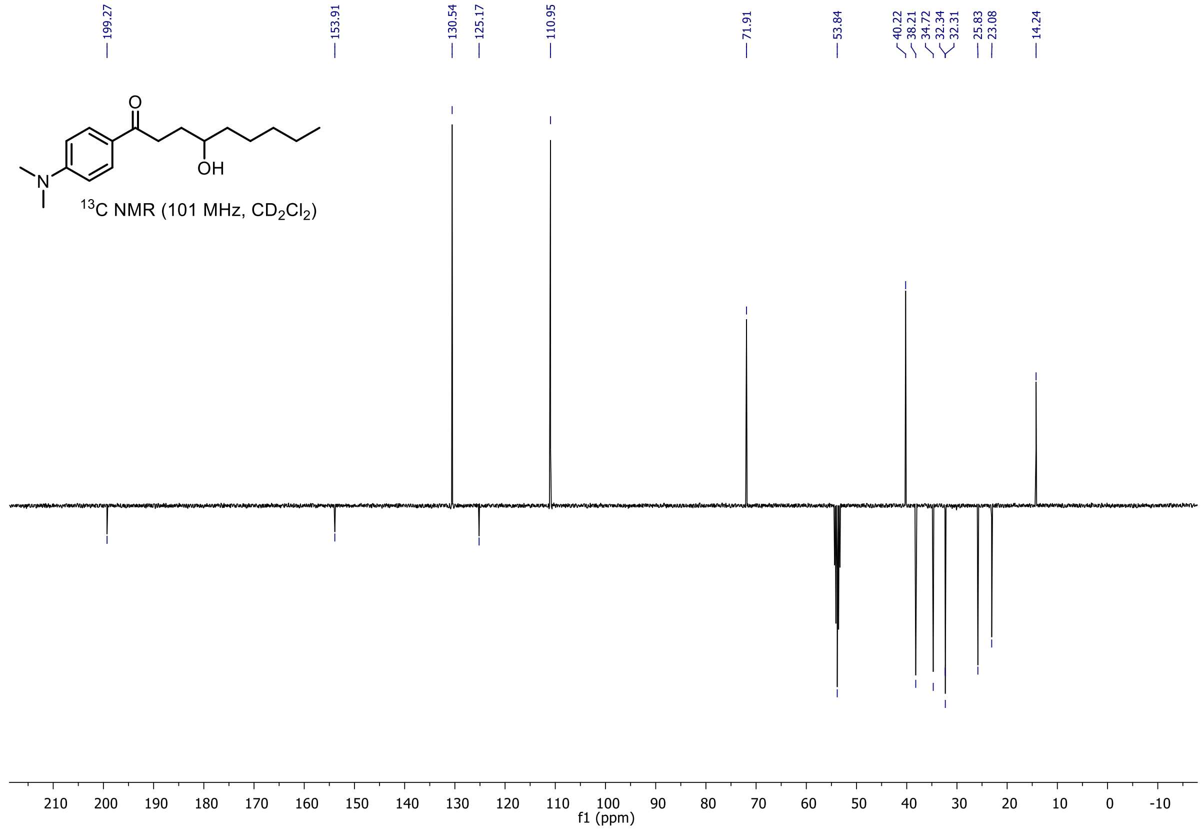

1-(2-Aminophenyl)-5-hydroxynonan-1-one (2q)

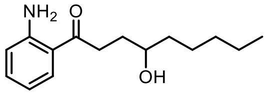

The title compound was prepared following general procedure G with modifications to the reaction temperature and time (65 °C instead of 120 °C for 1 h) using 1-(2-aminophenyl)non-8-en-1-one (17.8 mg, 77 µmol, 1.00 equiv.) and TfOH (20 µL, 0.23 mmol, 3.00 equiv.). Purification by flash column chromatography (0 – 100% EtOAc in heptanes) afforded the title compound (16.0 mg, 64 µmol, 64%) as a yellow solid and single regioisomer.

Crude product: 67% NMR yield, r.r. (γ_{OH}:δ_{OH}) = >95:5.

^{1}H NMR (400 MHz, $(CD_3)_2CO$): δ 7.83 (dd, *J* = 8.1, 1.2 Hz, 1H), 7.23 (ddd, *J* = 8.4, 7.1, 1.5 Hz, 1H), 6.94 (br s, 2H), 6.78 (dd, *J* = 8.4, 0.8 Hz, 1H), 6.57 (ddd, *J* = 8.1, 7.0, 1.2 Hz, 1H), 3.67 – 3.57 (m, 1H), 3.52 (d, *J* = 5.5 Hz, 1H), 3.17 – 3.00 (m, 2H), 1.93 – 1.83 (m, 1H), 1.75 – 1.63 (m, 1H), 1.54 – 1.43 (m, 3H), 1.37 – 1.25 (m, 5H), 0.89 (t, *J* = 6.8 Hz, 3H).

^{13}C NMR (101 MHz, $(CD_3)_2CO$): δ 203.4, 152.2, 134.7, 132.1, 118.3, 118.0, 115.6, 71.0, 38.6, 36.1, 33.4, 32.8, 26.3, 23.4, 14.4.

IR (neat) ν_{max}: 3452, 3342, 2926, 2856, 1703, 1614, 1581, 1547, 1484, 1450, 1319, 1203, 1159, 971, 518.

HRMS (ESI$^+$): exact mass calculated for $[M+Na]^+$ $(C_{15}H_{23}NO_2Na)^+$ requires *m/z* 272.1621, found *m/z* 272.1613.

1-(2-Aminophenyl)-5-hydroxynonan-1-one (2q)

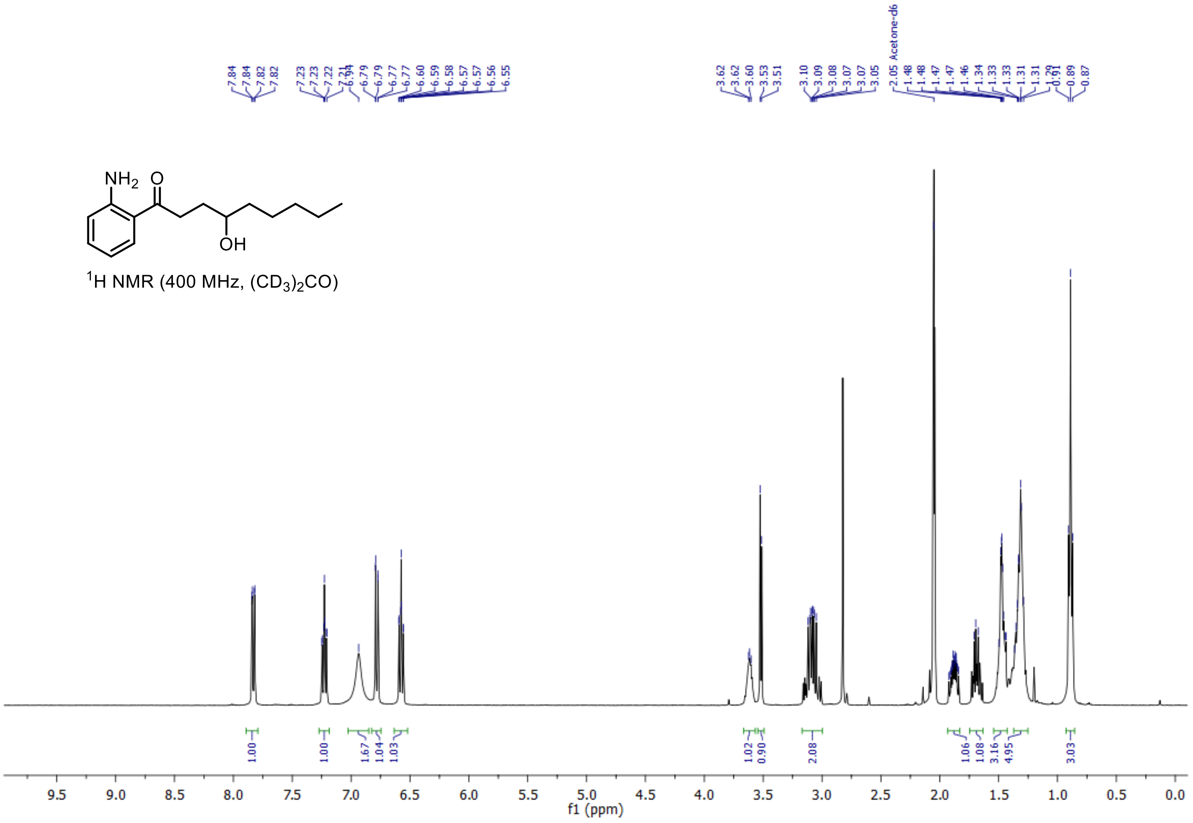

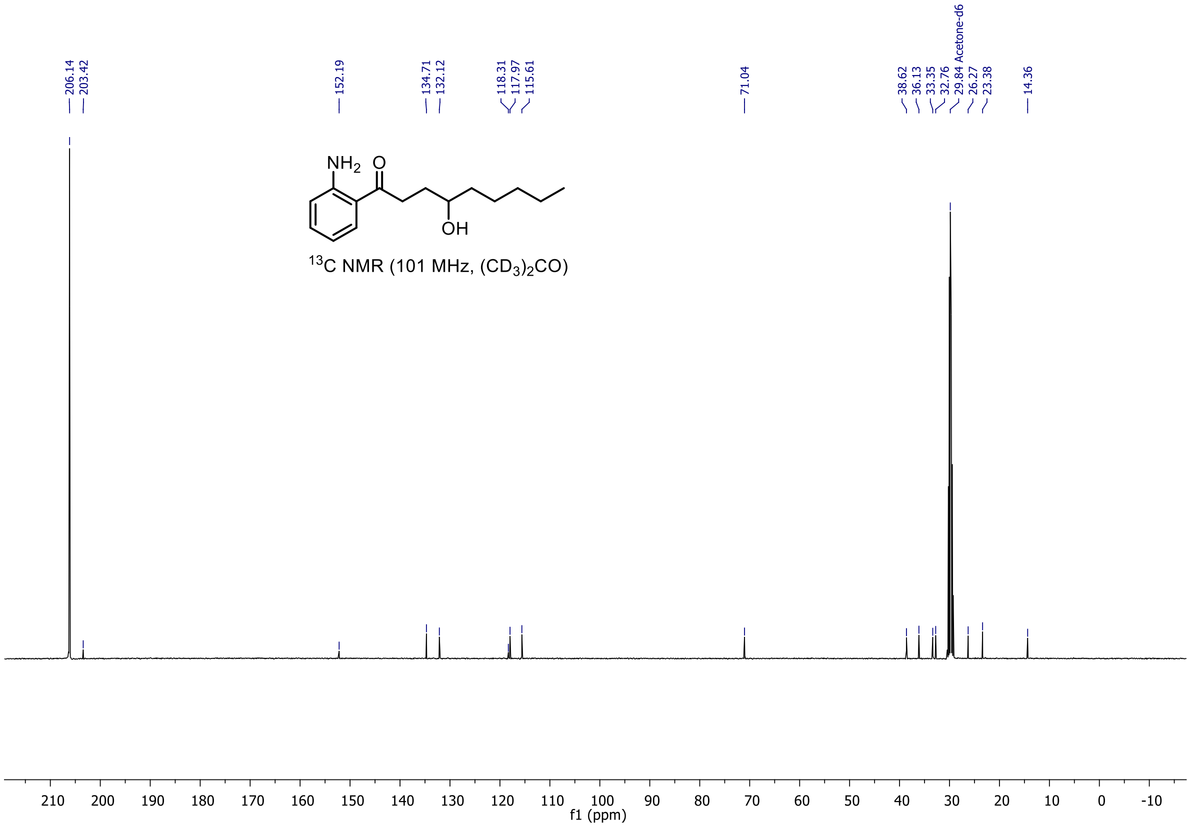

4-Hydroxy-1-(thiophen-2-yl)nonan-1-one (2r)

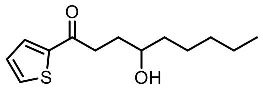

The title compound was prepared following general procedure G using 1-(thiophen-2-yl)non-8-en-1-one (22.2 mg, 0.10 mmol, 1.00 equiv.) and TfOH (27 µL, 0.30 mmol, 3.00 equiv.). Purification by flash column chromatography (0 – 70% EtOAc in heptanes) afforded the title compound (17.9 mg, 75 µmol, 75%) as a pale-yellow oil and a single regioisomer.

Crude product: 91% NMR yield, r.r. (γ_{OH}:δ_{OH}) = 94:6

^{1}H NMR (400 MHz, CDCl$_3$): δ 7.75 (dd, *J* = 3.8, 1.1 Hz, 1H), 7.63 (dd, *J* = 4.9, 1.1 Hz, 1H), 7.12 (dd, *J* = 4.9, 3.8 Hz, 1H), 3.74 – 3.53 (m, 1H), 3.08 (td, *J* = 7.3, 2.3 Hz, 2H), 1.97 (ddd, *J* = 14.5, 7.3, 3.4 Hz, 1H), 1.89 – 1.72 (m, 2H), 1.56 – 1.21 (m, 8H), 0.89 (t, *J* = 6.8 Hz, 3H).

^{13}C NMR (101 MHz, CDCl$_3$): δ 193.9, 144.4, 133.7, 132.2, 128.3, 71.5, 37.9, 35.8, 32.0, 31.8, 25.5, 22.8, 14.2.

IR (neat) ν_{max}: 3416, 2953, 2927, 2857, 1654, 1517, 1455, 1413, 1262, 1080, 856, 718.

HRMS (ESI$^+$): exact mass calculated for [M+Na]$^+$ ($C_{13}H_{20}O_2SNa$)$^+$ requires *m/z* 263.1076, found *m/z* 263.1071.

4-Hydroxy-1-(thiophen-2-yl)nonan-1-one (2r)

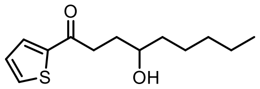

^{1}H NMR (400 MHz, $CDCl_3$)

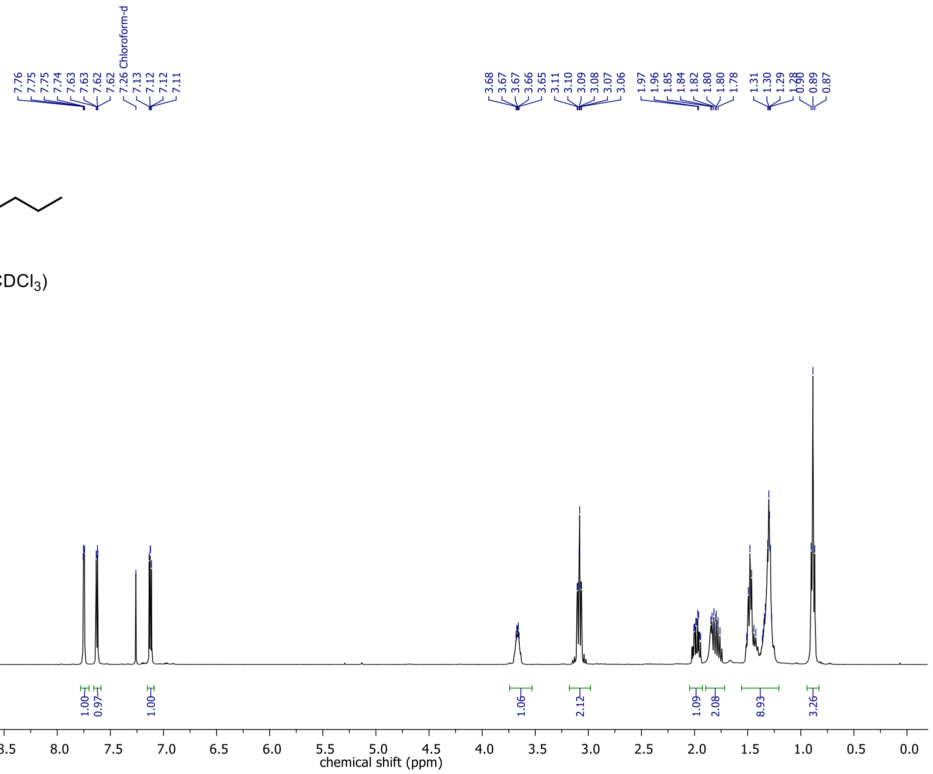

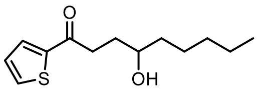

^{13}C NMR (101 MHz, $CDCl_3$)

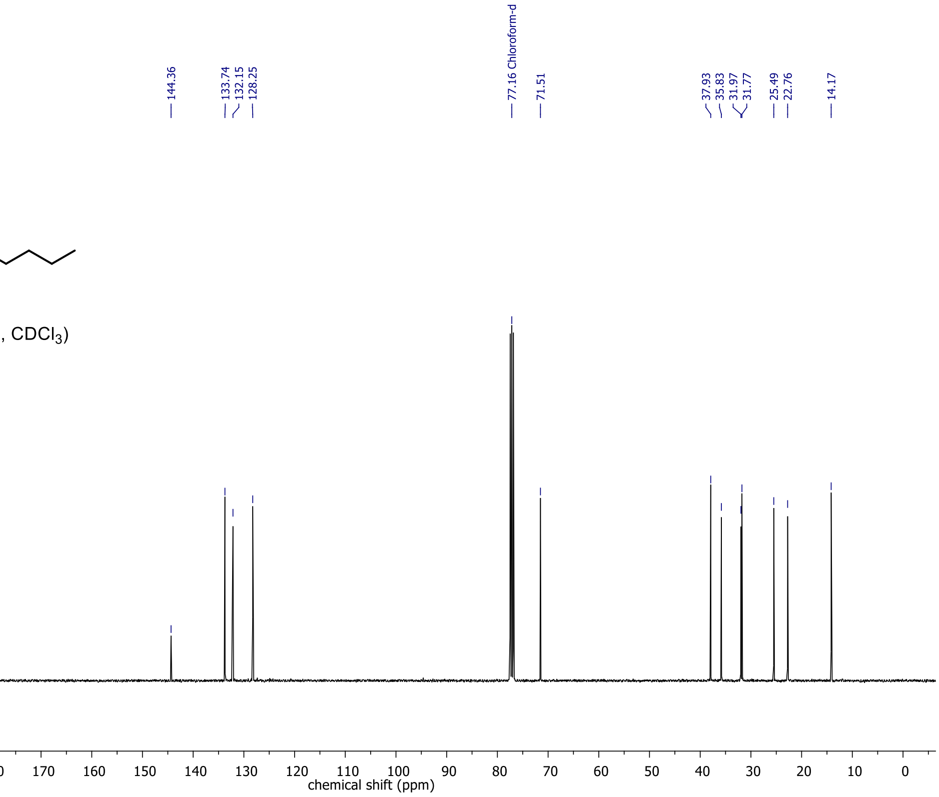

1-(Furan-2-yl)-4-hydroxynonan-1-one (2s)

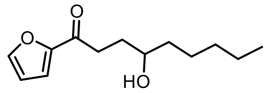

The title compound was prepared following general procedure G using 1-(furan-2-yl)non-8-en-1-one (20.6 mg, 0.10 mmol, 1.00 equiv.) and TfOH (27 µL, 0.30 mmol, 3.00 equiv.). Purification by flash column chromatography (20 – 50% EtOAc in heptanes) afforded the title compound (11.8 mg, 53 µmol, 53%) as a colorless solid and a single regioisomer.

Crude product: 63% NMR yield; r.r. (γ_{OH}:δ_{OH}) = 90:10

^{1}H NMR (400 MHz, CDCl$_3$) δ 7.58 (d, *J* = 1.1 Hz, 1H), 7.21 (d, *J* = 3.5 Hz, 1H), 6.53 (dd, *J* = 3.5, 1.7 Hz, 1H), 3.70 – 3.60 (m, 1H), 3.06 – 2.92 (m, 2H), 1.95 (dtd, *J* = 14.5, 7.3, 3.5 Hz, 1H), 1.88 – 1.71 (m, 2H), 1.52 – 1.40 (m, 3H), 1.37 – 1.26 (m, 5H), 0.88 (t, *J* = 6.8 Hz, 3H).

^{13}C NMR (101 MHz, CDCl$_3$) δ 190.1 (C), 152.8 (C), 146.5 (CH), 117.3 (CH), 112.3 (CH), 71.5 (CH), 37.9 (CH$_2$), 34.9 (CH$_2$), 32.0 (CH$_2$), 31.4 (CH$_2$), 25.5 (CH$_2$), 22.8 (CH$_2$), 14.2 (CH$_3$).

IR (neat) ν_{max}: 3437, 2953, 2927, 2857, 1667, 1568, 1467, 1394, 1014, 883, 761, 595.

HRMS (ESI$^+$): exact mass calculated for $[M+Na]^+$ $(C_{13}H_{20}O_3Na)^+$ requires *m*/*z* 247.1305, found *m*/*z* 247.1299.

1-(Furan-2-yl)-4-hydroxynonan-1-one (2s)

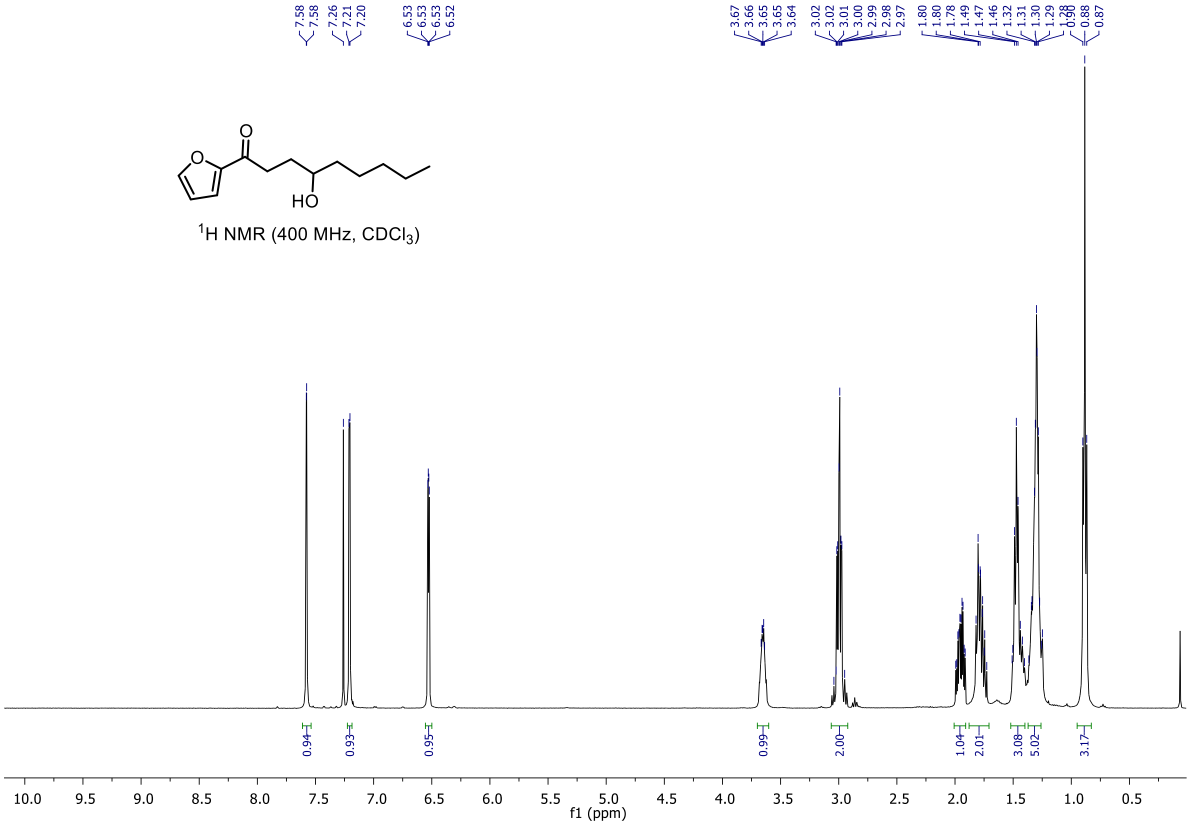

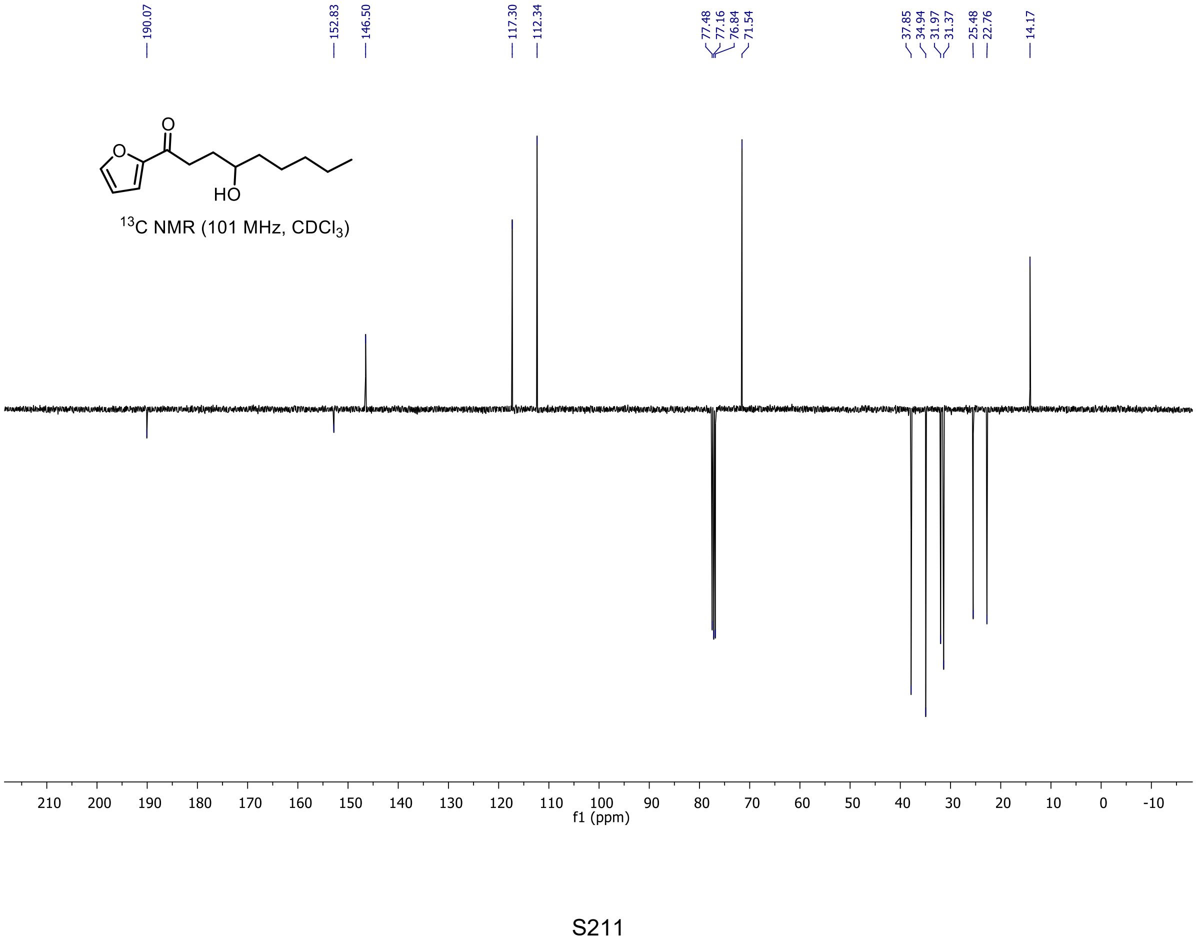

1-(3,5-Dimethylisoxazol-4-yl)-4-hydroxynonan-1-one (2t)

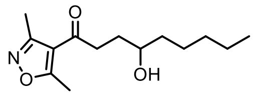

The title compound was prepared following general procedure G, with modifications to the reaction temperature and time (65°C instead of 120 °C for 1 h), using 1-(3,5-dimethylisoxazol-4-yl)non-8-en-1-one (23.5 mg, 0.10 mmol, 1.00 equiv.) and TfOH (27 µL, 0.30 mmol, 3.00 equiv.). Purification by flash column chromatography (0 – 100% EtOAc in heptanes) afforded the title compound (17.1 mg, 67 µmol, 67%) as a colorless oil and single regioisomer.

Crude product: 69% NMR yield, r.r. (γ_{OH}:δ_{OH}) > 95:5.

The acidic OH proton is not reported due to fast exchange processes.

^{1}H NMR (400 MHz, CDCl$_3$): δ 3.70 – 3.60 (m, 1H), 2.89 (td, *J* = 6.7, 2.8 Hz, 2H), 2.69 (s, 3H), 2.47 (s, 3H), 1.95 (dtd, *J* = 14.3, 7.1, 3.3 Hz, 1H), 1.82 – 1.68 (m, 1H), 1.53 – 1.41 (m, 2H), 1.40 – 1.23 (m, 6H), 0.89 (t, *J* = 6.7 Hz, 3H).

^{13}C NMR (101 MHz, CDCl$_3$): δ 195.5, 173.9, 159.3, 117.0, 71.43 38.8, 38.2, 32.0, 30.8, 25.5, 22.9, 14.5, 14.2, 12.8.

IR (neat) ν_{max}: 3433, 2974, 2913, 2877, 1702, 1420, 1411, 1077, 1013, 972, 865.

HRMS (ESI$^+$): exact mass calculated for [M+Na]$^+$ ($C_{14}H_{23}NO_3Na$)$^+$ requires *m/z* 276.1570, found *m/z* 276.1567.

1-(3,5-Dimethylisoxazol-4-yl)-4-hydroxynonan-1-one (2t)

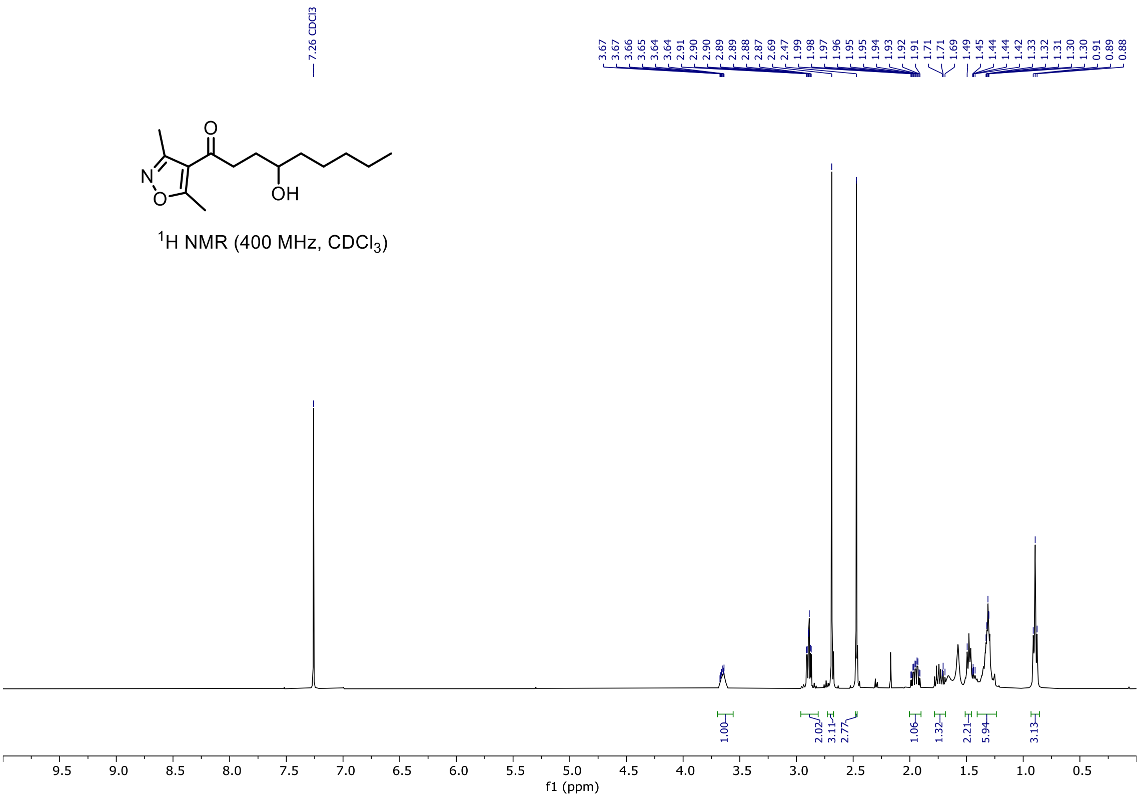

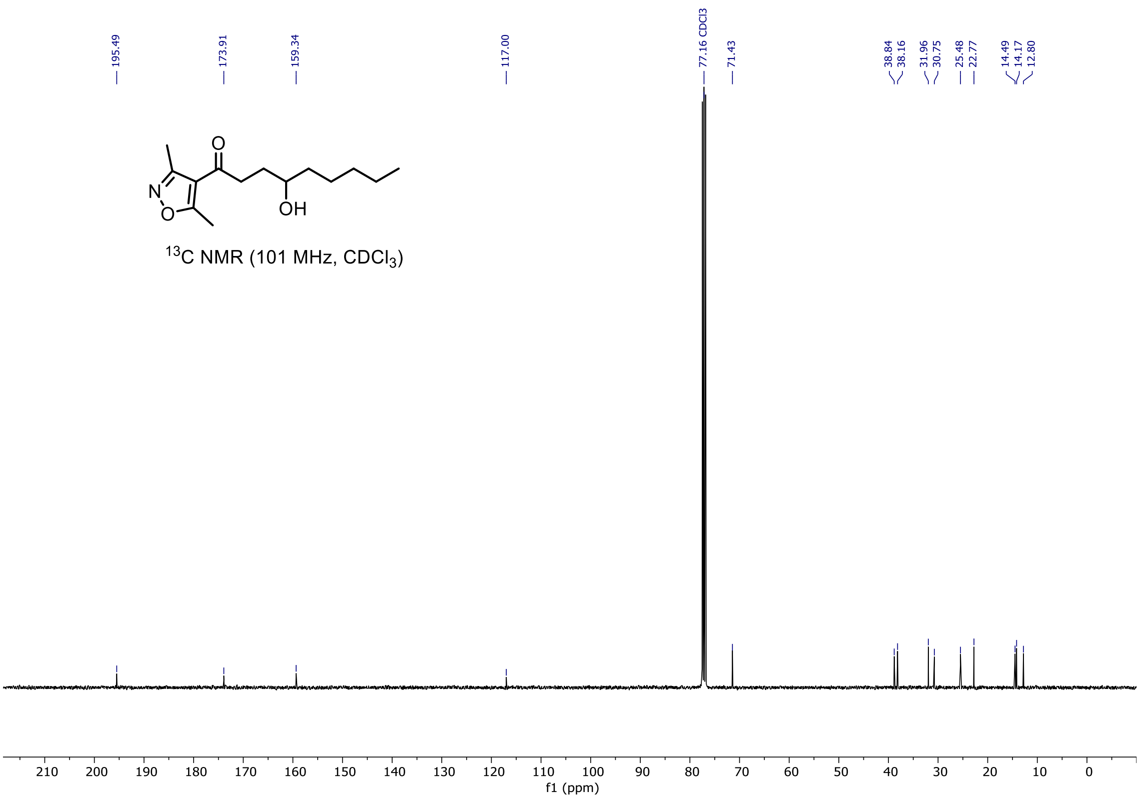

1-(3-(Difluoromethyl)-1-methyl-1H-pyrazol-4-yl)-4-hydroxynonan-1-one (2u)

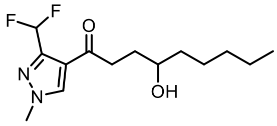

The title compound was prepared following general procedure G, with a modification to the reaction temperature (65 °C instead of 120 °C for 1 h), using 1-(3-(difluoromethyl)-1-methyl-1H-pyrazol-4-yl)non-8-en-1-one (27.0 mg, 0.10 mmol, 1.00 equiv.) and TfOH (27 µL, 0.30 mmol, 3.00 equiv.). Purification by flash column chromatography (0 – 100% EtOAc in heptanes) afforded the title compound (19.0 mg, 55 µmol, 55%) as a colorless oil and a mixture of regioisomers (γ:δ = 95:5).

Crude product: 60% NMR yield, r.r. (γ_{OH}:δ_{OH}) = 95:5.

A full list of signals arising from the minor δ-regioisomer can be found in Section 2.4.

The acidic OH proton is not reported due to fast exchange processes.

^{1}H NMR (400 MHz, CD_2Cl_2): δ 7.93 (s, 1H), 7.13 (t, *J* = 54.0 Hz, 1H), 3.95 (s, 3H), 3.62 – 3.57 (m, 1H), 2.97 – 2.83 (m, 2H), 1.96 – 1.83 (m, 1H), 1.81 – 1.62 (m, 2H), 1.48 – 1.25 (m, 7H), 0.89 (t, *J* = 6.9 Hz, 3H).

^{13}C NMR (101 MHz, CD_2Cl_2): δ 195.1, 146.2 (t, *J* = 24.0 Hz), 135.1, 121.7 (t, *J* = 3.4 Hz), 110.4 (t, *J* = 236.1 Hz), 71.7, 40.2, 38.4, 37.6, 32.4, 31.8, 25.9, 23.2, 14.4.

^{19}F NMR (376 MHz, CD_2Cl_2): δ -116.34.

IR (neat) ν_{max}: 3388, 3011, 2956, 2922, 1700, 1682, 1344, 1172, 1103, 1067, 866.

HRMS (ESI$^+$): exact mass calculated for $[M+H]^+$ $(C_{14}H_{23}F_2N_2O_2)^+$ requires *m/z* 289.1722, found *m/z* 289.1729.

1-(3-(Difluoromethyl)-1-methyl-1H-pyrazol-4-yl)-4-hydroxynonan-1-one (2u)

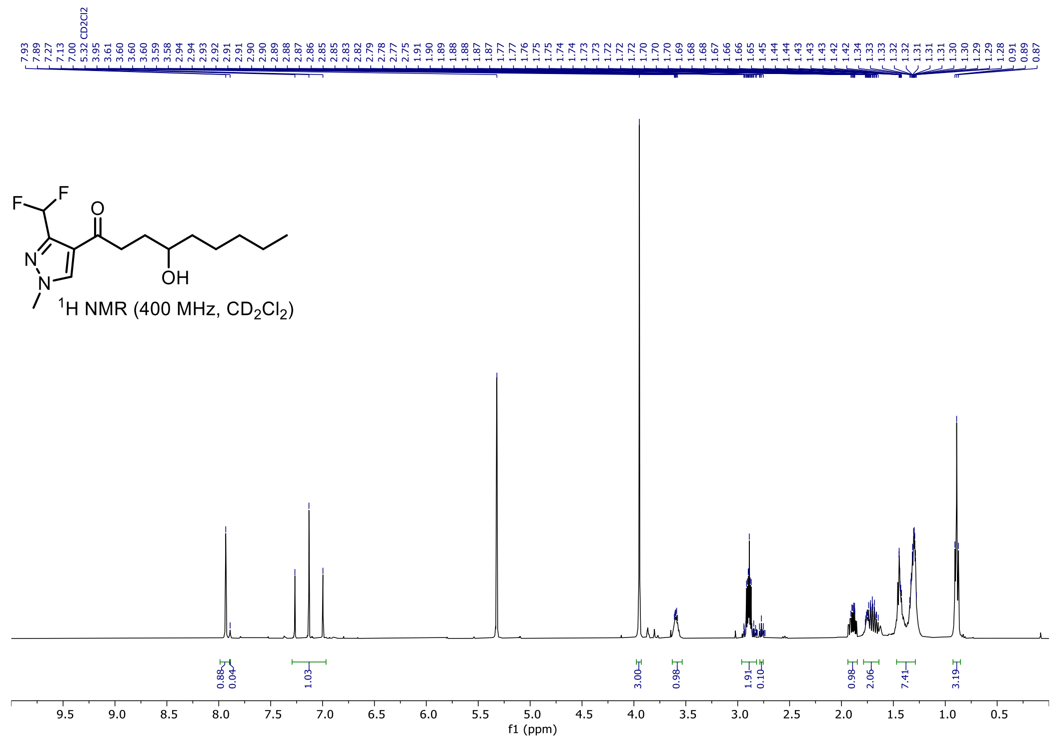

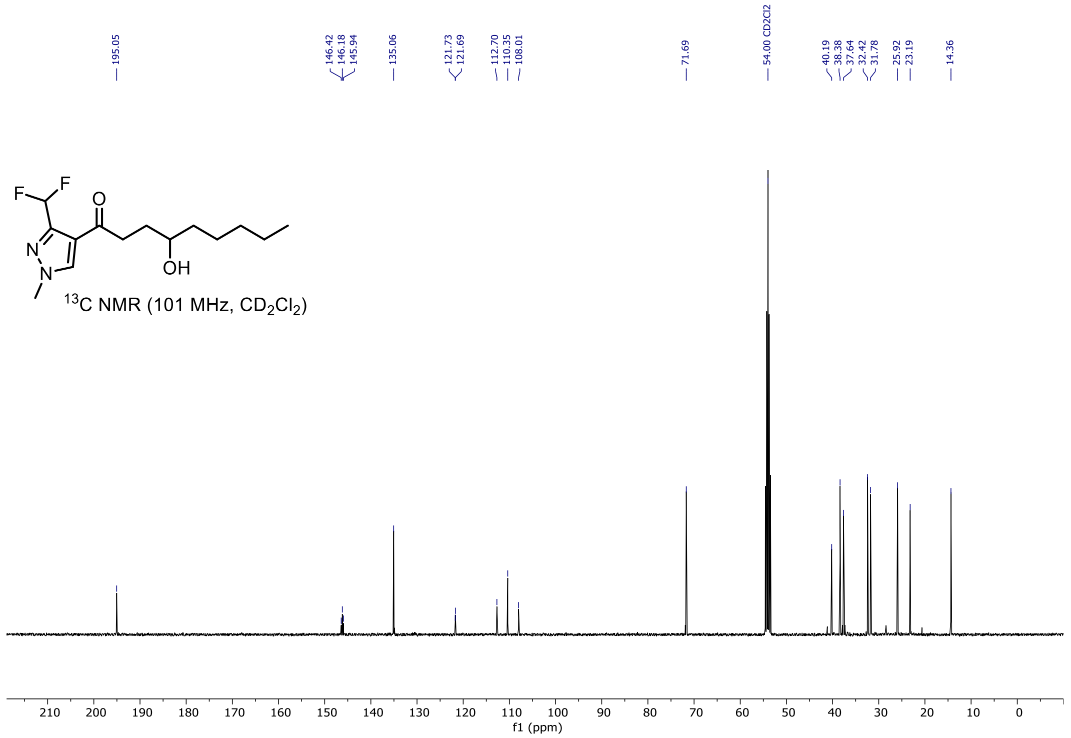

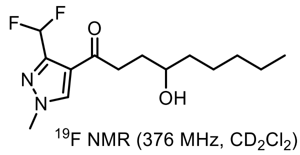

^{19}F NMR (376 MHz, CD_2Cl_2)

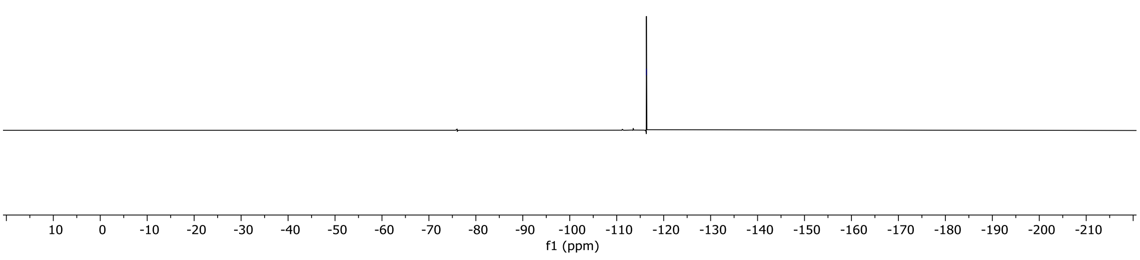

4-Hydroxy-1-(2-hydroxyphenyl)nonan-1-one (2v)

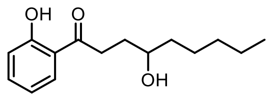

The title compound was prepared following general procedure G using 1-(2-hydroxyphenyl)non-8-en-1-one (23.2 mg, 0.10 mmol, 1.00 equiv.) and TfOH (27 µL, 0.30 mmol, 3.00 equiv.). Purification by flash column chromatography (0 – 100% EtOAc in heptanes) afforded the title compound (17.0 mg, 68 µmol, 68%) as a yellowish oil and single regioisomer.

Crude product: 70% NMR yield (γ_{OH}), r.r. (γ_{OH}:δ_{OH}) > 95:5.

^{1}H NMR (400 MHz, $(CD_3)_2CO$): δ 12.39 (d, *J* = 8.4 Hz, 1H), 7.99 (dd, *J* = 8.0, 1.5 Hz, 1H), 7.59 – 7.47 (m, 1H), 7.01 – 6.90 (m, 2H), 3.70 – 3.62 (m, 1H), 3.59 (d, *J* = 5.5 Hz, 1H), 3.33 – 3.14 (m, 2H), 1.99 – 1.87 (m, 1H), 1.82 – 1.67 (m, 1H), 1.53 – 1.45 (m, 3H), 1.40 – 1.28 (m, 5H), 0.89 (t, *J* = 6.8 Hz, 3H).

^{13}C NMR (101 MHz, $(CD_3)_2CO$): δ 208.6, 163.3, 137.1, 131.5, 120.3, 119.8, 118.8, 70.8, 38.6, 35.4, 32.8, 32.7, 26.2, 23.4, 14.3.

IR (neat) ν_{max}: 3394, 2929, 2858, 1713, 1639, 1614, 1581, 1488, 1447, 1358, 1282, 1249, 1223, 1199.

HRMS (ESI$^+$): exact mass calculated for $[M+Na]^+$ $(C_{15}H_{22}O_3Na)^+$ requires *m/z* 273.1461, found *m/z* 273.1453.

4-Hydroxy-1-(2-hydroxyphenyl)nonan-1-one (2v)

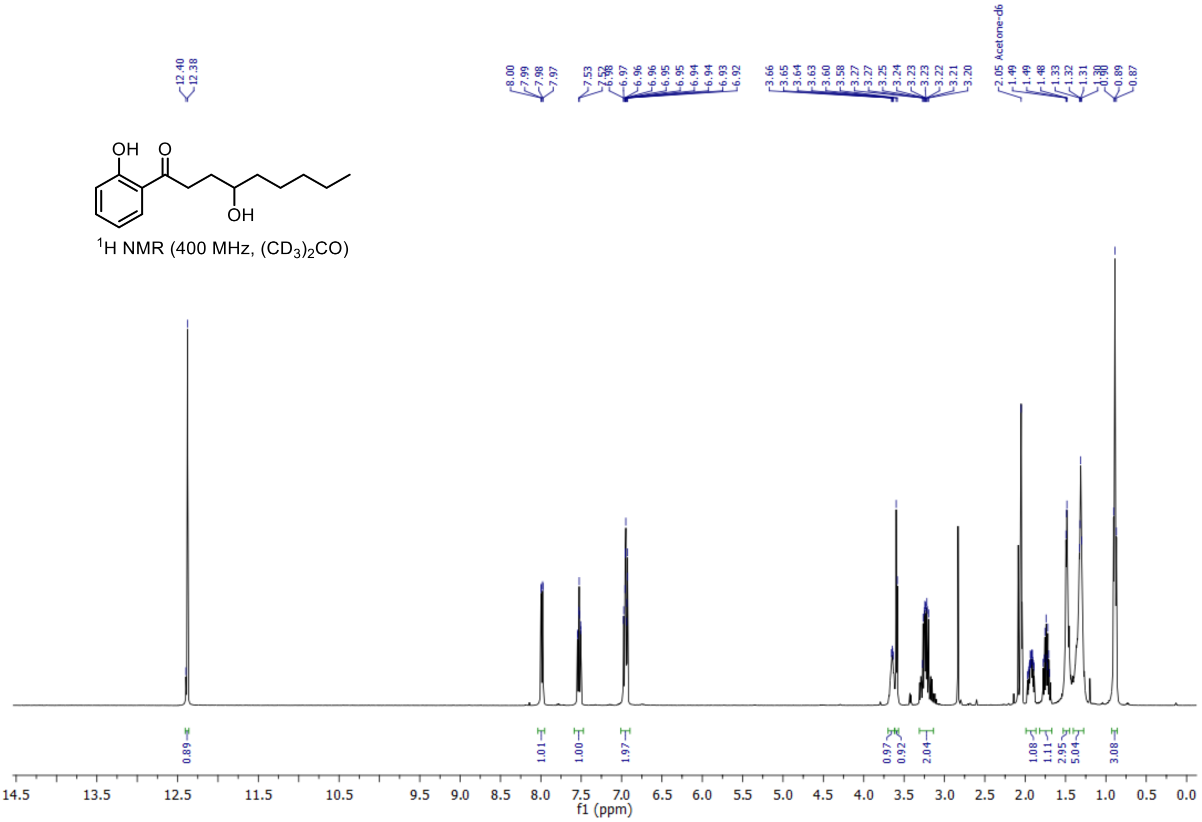

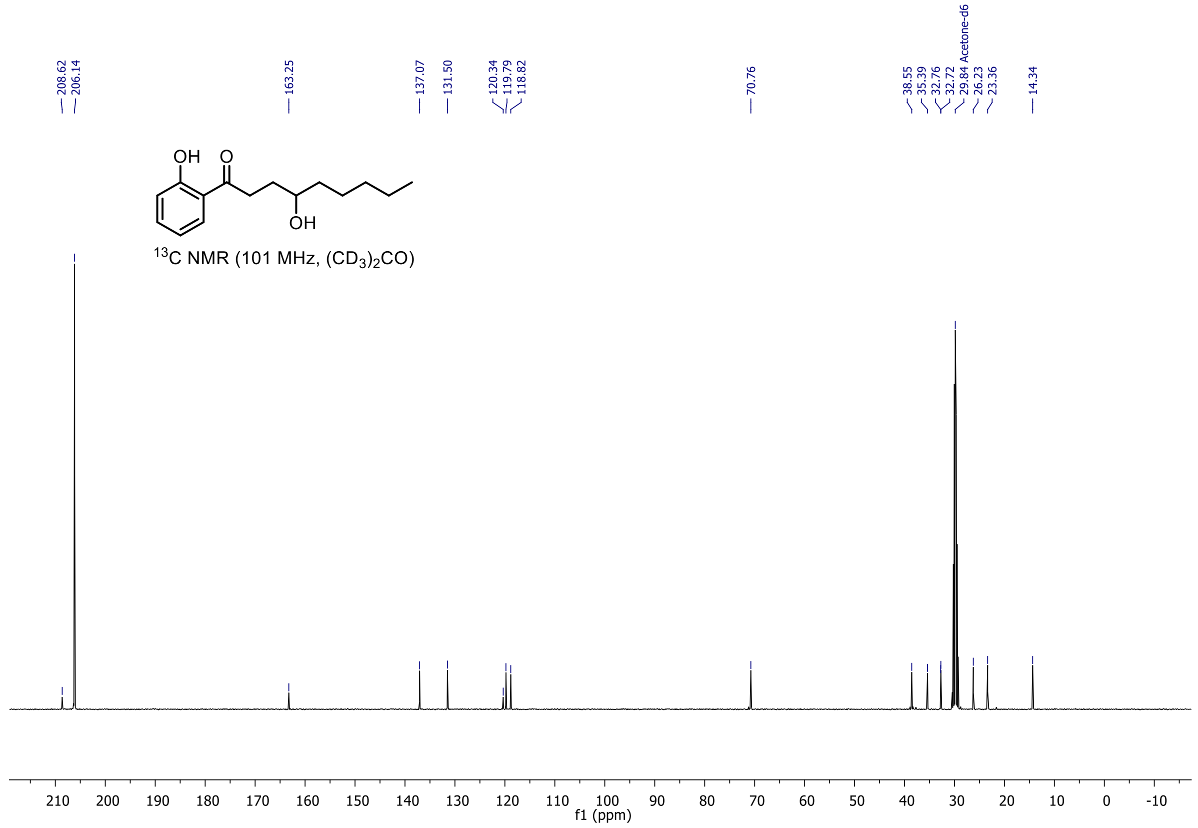

1-Cyclohexyl-4-hydroxydecan-1-one (2w)

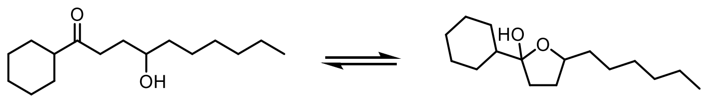

4.9 : 1
(2 diastereomers)

The title compound was prepared following general procedure G with a modification to the reaction temperature and time (100 °C instead of 120 °C, 1 h instead of 3 h) using 1-cyclohexyldec-9-en-1-one (24.6 mg, 0.10 mmol, 1.00 equiv.) and TfOH (27 µL, 0.30 mmol, 3.00 equiv.). Purification by flash column chromatography (10 – 40% EtOAc in heptanes) afforded the title compound (14.7 mg, 58 µmol, 58%) as a colorless oil and a single regioisomer. The γ-hydroxy ketone is in equilibrium with its hemiacetal form (ratio = 4.9:1, two diastereomers in ratio 1.3:1).

Crude product: 64% NMR yield, r.r. ($\gamma_{OH+Hemiacetal}$:δ_{OH}) = 94:6

An asterisk (*) denotes signals that unambiguously arise from the hemiacetal (2 diastereomers).

^{1}H NMR (400 MHz, $CDCl_3$) δ 4.21 – 4.13 (m, 0.1H*), 3.97 – 3.88 (m, 0.1H*), 3.54 (app br s, 0.8H), 2.60 (t, *J* = 7.0 Hz, 1.7H), 2.36 (tt, *J* = 11.2, 3.4 Hz, 0.8H), 1.89 – 1.72 (m, 6.1H), 1.71 – 1.56 (m, 2.3H), 1.45 – 1.22 (m, 15.1H), 0.87 (t, *J* = 6.7 Hz, 3H).

Due to low abundance, only signals arising from the major component (γ-hydroxy ketone) are reported in ^{13}C NMR.

^{13}C NMR (151 MHz, $CDCl_3$) δ 215.2 (C), 71.7 (CH), 51.1 (CH), 38.0 (CH_2), 37.2 (CH_2), 32.0 (CH_2), 31.0 (CH_2), 29.45 (CH_2), 28.7 ($2CH_2$), 26.40, 25.99 (CH_2), 25.82 ($2CH_2$), 25.78 (CH_2), 22.7 (CH_2), 14.2 (CH_3).

IR (neat) ν_{max}: 3420, 2924, 2853, 1702, 1449, 1042, 735.

HRMS (ESI$^+$): exact mass calculated for $[M+Na]^+$ $(C_{16}H_{30}O_2Na)^+$ requires *m*/*z* 277.2138, found *m*/*z* 277.2133.

1-Cyclohexyl-4-hydroxydecan-1-one (2w)

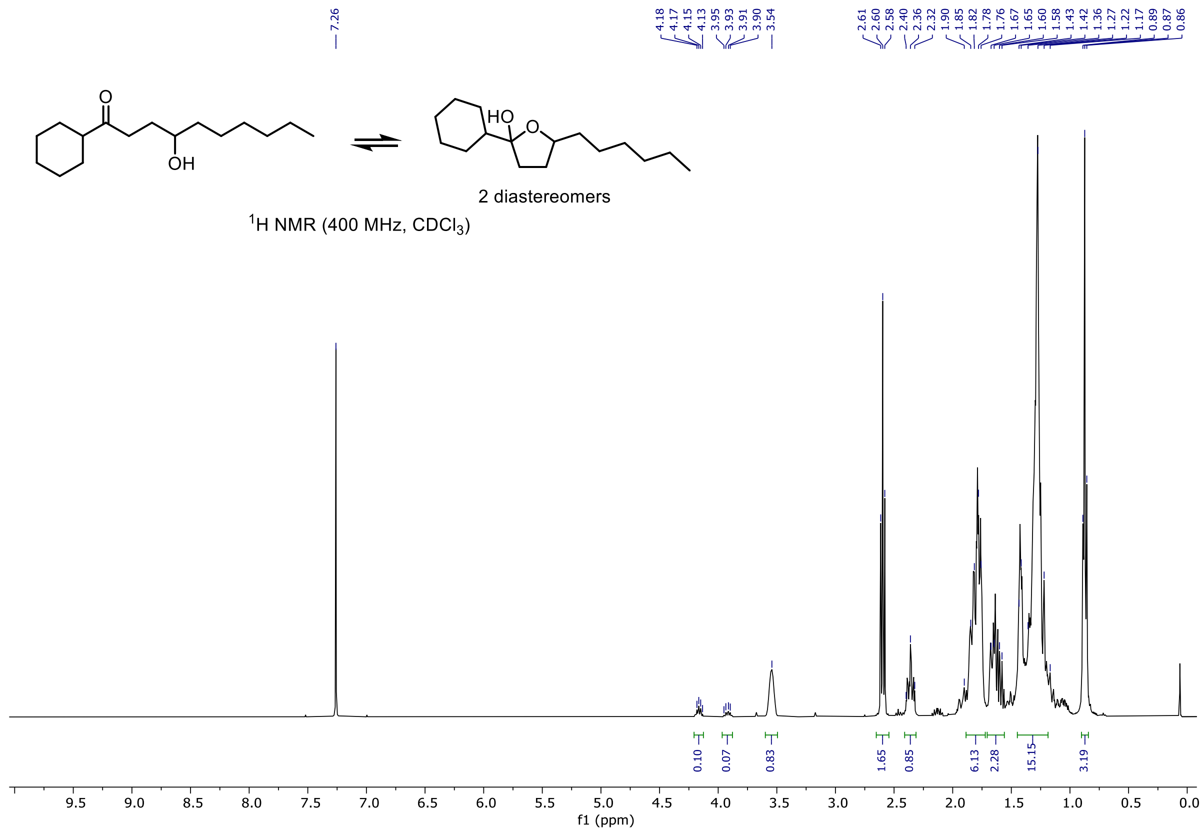

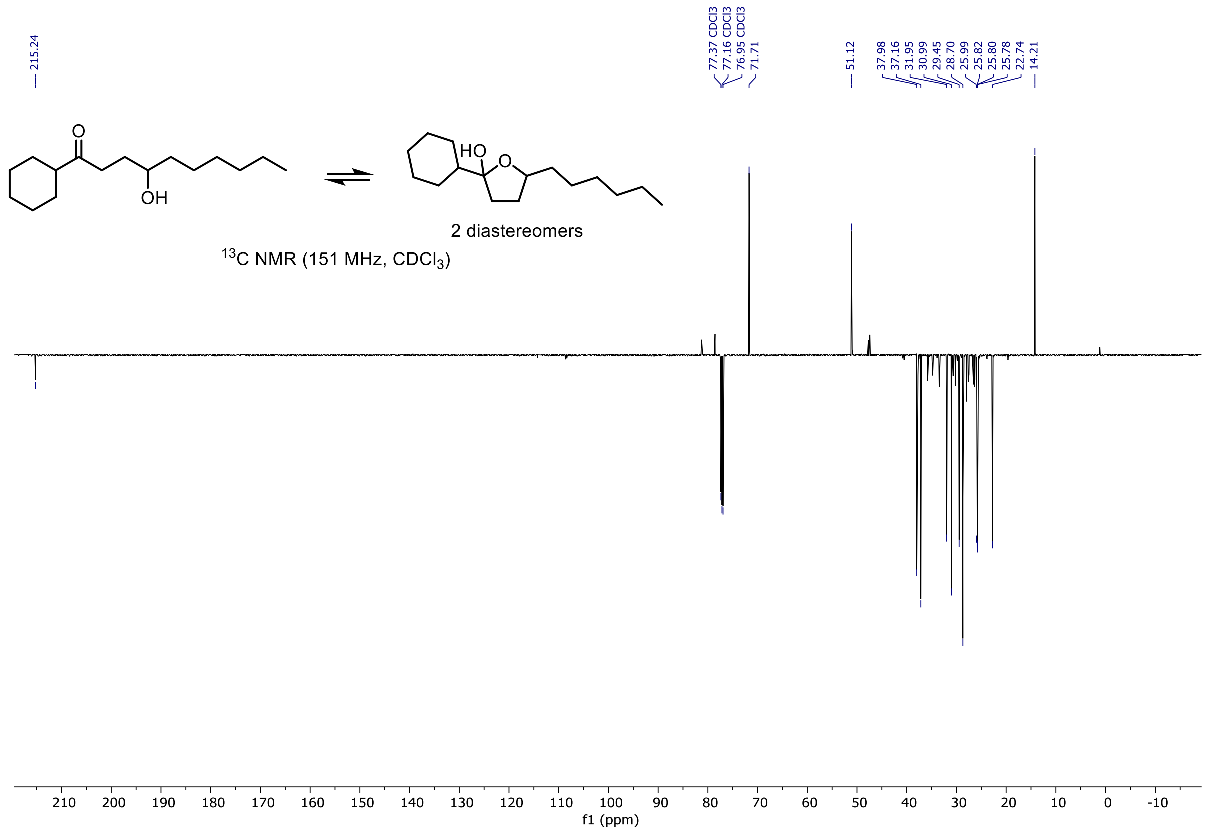

1-(Adamantan-1-yl)-4-hydroxynonan-1-one (2x)

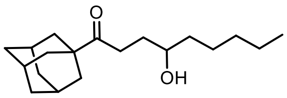

The title compound was prepared following general procedure F using 1-(adamantan-1-yl)non-8-en-1-one (27.4 mg, 0.10 mmol, 1.00 equiv.) and TfOH (27 µL, 0.30 mmol, 3.00 equiv.). Purification by flash column chromatography (0 – 100% EtOAc in heptanes) afforded the title compound (16.3 mg, 56 µmol, 56%) as a colorless liquid and a single regioisomer.

Crude product: 61% NMR yield, r.r. (γ_{OH}:δ_{OH}) = 90:10

^{1}H NMR (400 MHz, CD_2Cl_2): δ 3.50 (app br s, 1H), 2.62 – 2.56 (m, 2H), 2.10 – 2.00 (m, 3H), 1.80 (m, 7H), 1.76 – 1.65 (m, 7H), 1.55 (m, 2H), 1.44 – 1.36 (m, 2H), 1.34 – 1.27 (m, 5H), 0.89 (t, *J* = 6.9 Hz, 3H).

^{13}C NMR (101 MHz, CD_2Cl_2): δ 216.8, 72.0, 46.9, 38.8, 38.4 (3C), 37.1 (3C), 33.1, 32.5, 31.6, 28.7 (3C), 26.0, 23.2, 14.4.

IR (neat) ν_{max}: 3440, 2903, 2850, 1695, 1452, 1407, 1344, 1266, 1198, 1159, 1051, 1005, 931, 741.

HRMS (ESI^{+}): exact mass calculated for $[M+Na]^{+}$ ($C_{19}H_{32}O_2Na)^{+}$ requires *m/z* 315.2294, found *m/z* 315.2292.

1-(Adamantan-1-yl)-4-hydroxynonan-1-one (2x)

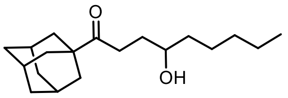

^{1}H NMR (400 MHz, CD_2Cl_2)

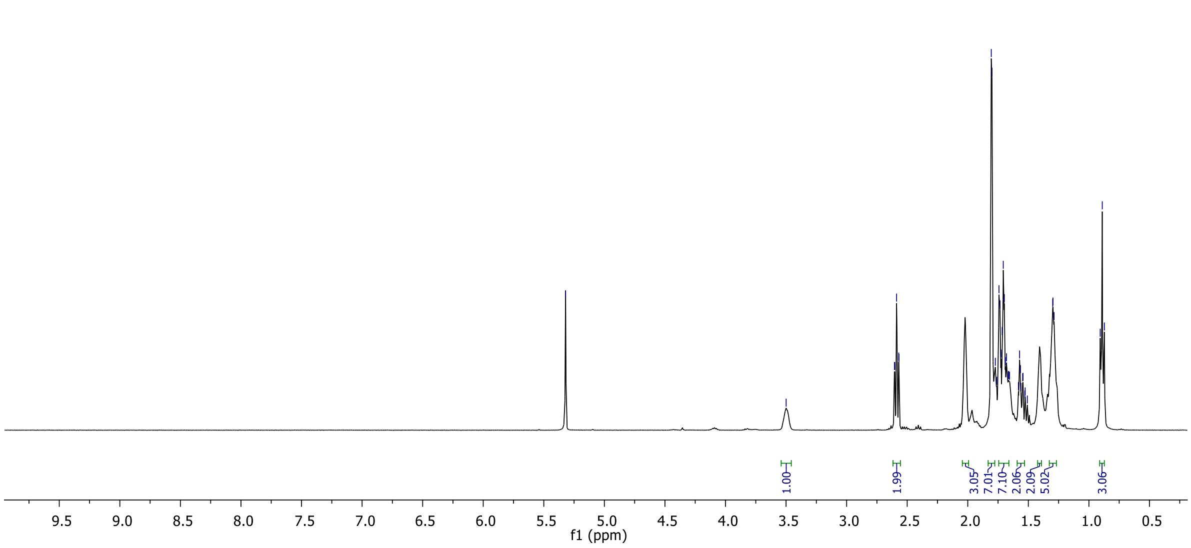

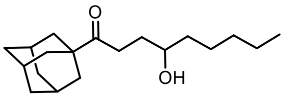

^{13}C NMR (101 MHz, CD_2Cl_2)

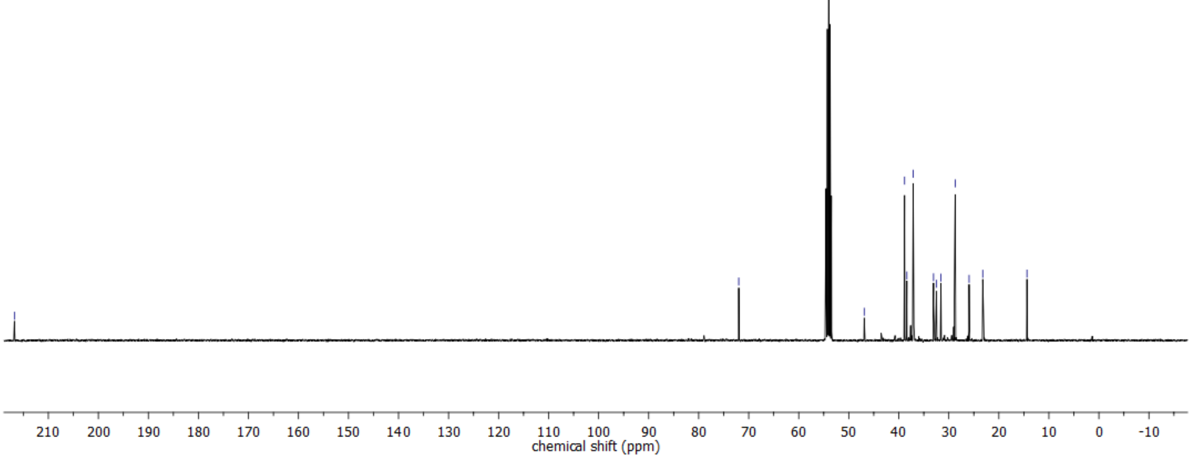

4-(4-Hydroxynonanoyl)-*N,N*-dipropylbenzenesulfonamide (2y)

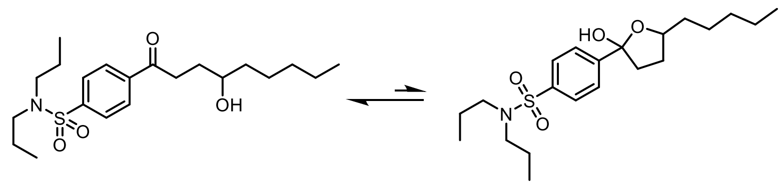

3.6 : 1

The title compound was prepared following general procedure G using 4-(non-8-enoyl)-N,N-dipropylbenzenesulfonamide (38.0 mg, 0.10 mmol, 1.00 equiv.) and TfOH (27 µL, 0.30 mmol, 3.00 equiv.). Purification by flash column chromatography (0 – 100% EtOAc in heptanes) afforded the title compound (15.7 mg, 40 µmol, 40%) as a yellow oil and a single regioisomer. The γ-hydroxy ketone is in equilibrium with its hemiacetal form (ratio = 3.6:1, two diastereomers in a ratio of 1.2:1).

Crude product: 55% NMR yield, r.r. (γ_{OH}:δ_{OH}) = 87:13.

An asterisk (*) denotes signals that unambiguously arise from the hemiacetal (2 diastereomers).

^{1}H NMR (400 MHz, CD_2Cl_2): δ 8.10 – 8.05 (m, 1.5H), 7.94 – 7.86 (m, 1.5H), 7.77 – 7.64 (m, 1H*), 4.43 – 4.23 (m, 0.2H*), 3.64 (app s, 0.8H), 3.22 – 3.02 (m, 5.6H), 2.61 (s, 0.2H*), 2.35 – 2.12 (m, 0.5H*), 2.01 – 1.87 (m, 1H), 1.81 – 1.29 (m, 13.8H), 0.93 – 0.83 (9H).

Due to low abundance and signal overlap, only some signals arising from the minor component (hemiacetal, 2 diastereomers) are reported.

^{13}C NMR (101 MHz, CD_2Cl_2): δ 200.0, 144.4, 140.2, 129.0 (2C), 127.6 (2C), 127.3* (2C), 126.6* (2C), 105.8*, 105.5*, 83.1*, 80.2*, 71.6, 50.52*, 50.50*, 50.4, 38.3, 35.7, 32.3, 31.6, 25.8, 23.1, 22.3, 14.2, 11.3.

IR (neat) ν_{max}: 3475, 2960, 2931, 2874, 1720, 1465, 1338, 1272, 1155, 1088, 1039, 1016, 989, 869, 839, 797, 777, 741, 598, 565.

HRMS (ESI$^+$): exact mass calculated for $[M+Na]^+$ $(C_{21}H_{35}NO_4SNa)^+$ requires *m/z* 420.2179, found *m/z* 420.2175.

4-(4-Hydroxynonanoyl)-*N,N*-dipropylbenzenesulfonamide (2y)

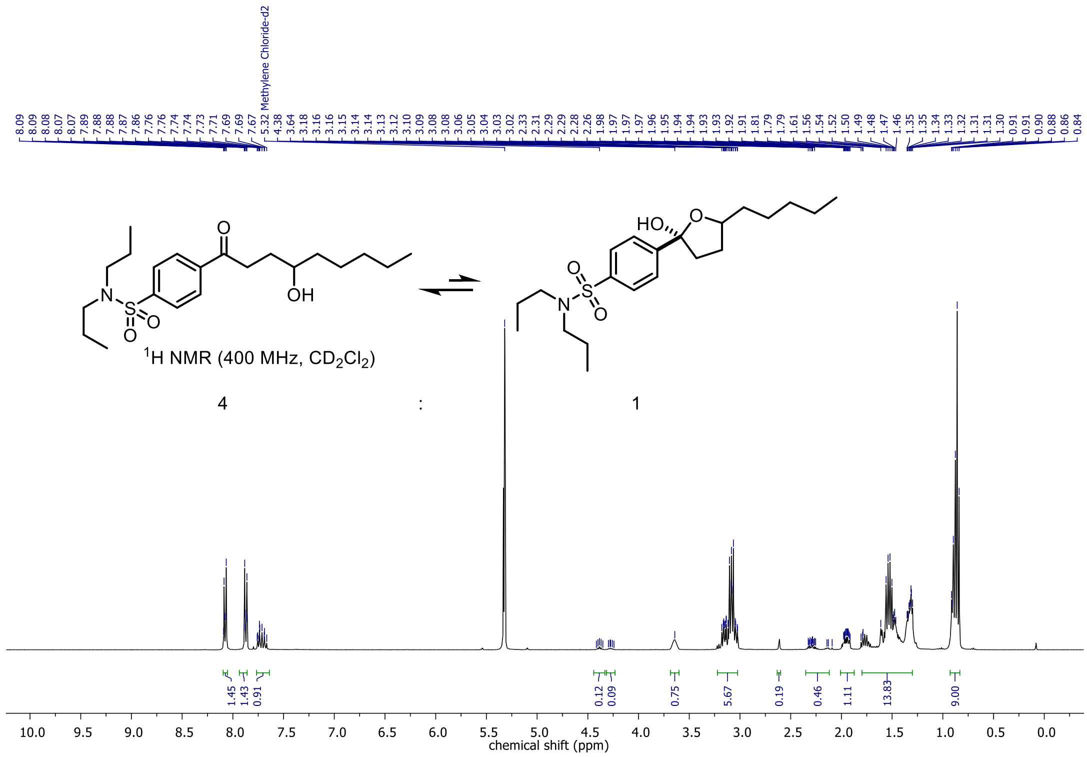

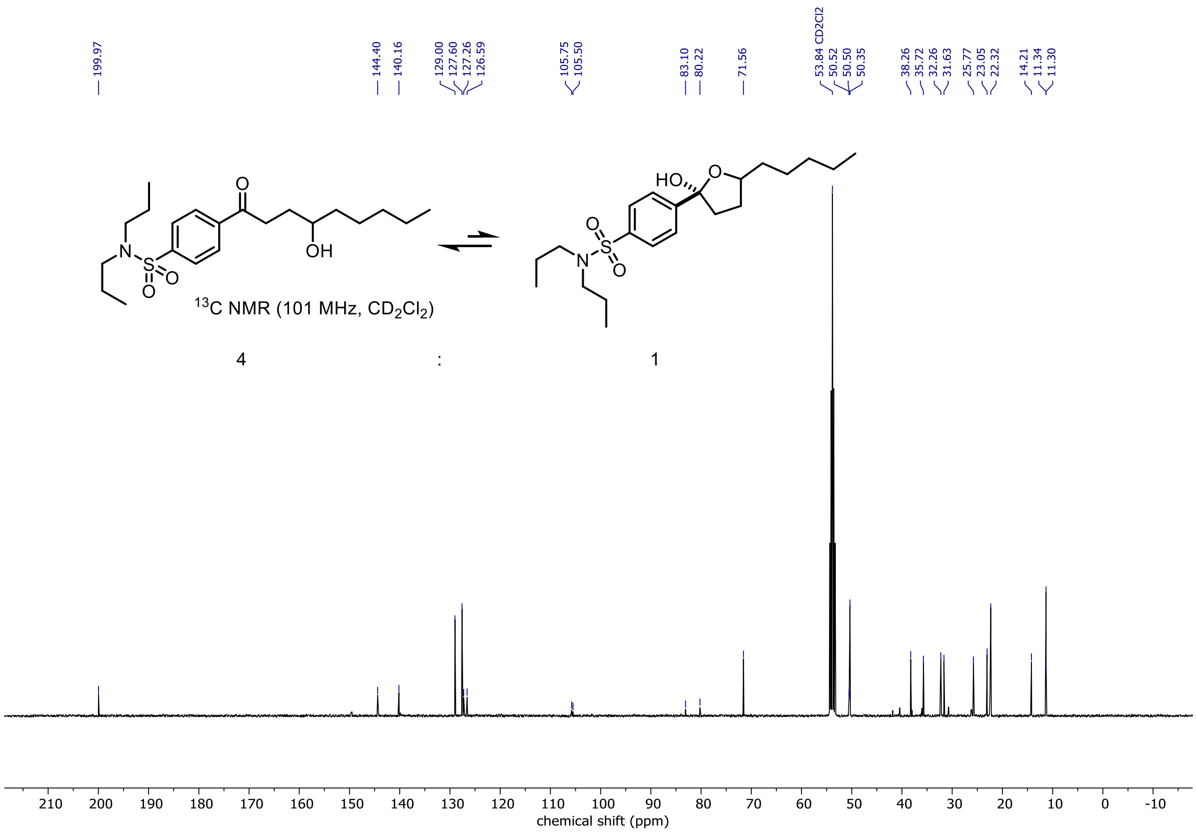

1-(1-(3-(Dimethylamino)propyl)-1-(4-fluorophenyl)-1,3-dihydroisobenzofuran-5-yl)-4-hydroxynonan-1-one (2z)

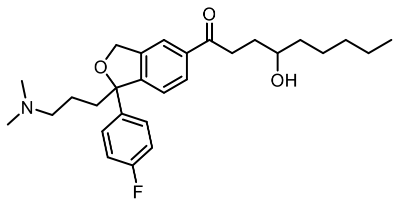

The title compound was prepared following general procedure G using 1-(1-(3-(dimethylamino)propyl)-1-(4-fluorophenyl)-1,3-dihydroisobenzofuran-5-yl)non-8-en-1-one (43.8 mg, 0.10 mmol, 1.00 equiv.) and TfOH (27 µL, 0.30 mmol, 3.00 equiv.). Purification by flash column chromatography (0 – 100% EtOAc/EtOH/28% NH_4OH (3:1:0.02) in heptanes) afforded the title compound (28.4 mg, 62 µmol, 62%) as a colorless solid and a single regioisomer.

Crude product: 67% NMR yield, r.r. (γ_{OH}:δ_{OH}) = 95:5

The acidic OH proton is not reported due to fast exchange processes.

^{1}H NMR (700 MHz, CD_2Cl_2) δ 7.94 – 7.89 (m, 1H), 7.83 (app br s, 1H), 7.51 – 7.46 (m, 2H), 7.38 (d, *J* = 8.0 Hz, 1H), 7.05 – 6.98 (m, 2H), 5.19 (dd, *J* = 27.7, 12.5 Hz, 2H), 3.62 (m, 1H), 3.17 – 3.04 (m, 2H), 2.24 (m, 2H), 2.22 – 2.16 (m, 2H), 2.18 – 2.14 (m, 2H), 2.12 (s, 6H), 1.94 – 1.89 (m, 1H), 1.78 – 1.72 (m, 1H), 1.49 – 1.42 (m, 3H), 1.34 – 1.27 (m, 5H), 0.89 (t, *J* = 7.0 Hz, 3H).

^{13}C NMR (176 MHz, CD_2Cl_2) δ 200.6, 162.5 (d, *J* = 244.6 Hz), 149.8, 141.31, 141.29 (d, *J* = 3.2 Hz), 140.4, 137.6, 128.5, 127.5 (d, *J* = 8.0 Hz, 2C), 122.5, 121.6, 115.5 (d, *J* = 21.3 Hz, 2C), 91.5, 72.2, 71.8, 59.9, 45.5, 39.5, 38.4, 35.7, 32.5, 32.1, 26.0, 23.2, 22.6, 14.4.

^{19}F NMR (659 MHz, CD_2Cl_2): δ -116.85.

IR (neat) ν_{max}: 3356, 2929, 2857, 1678, 1506, 1223, 1031, 954, 832, 709, 530.

HRMS (ESI$^+$): exact mass calculated for $[M+H]^+$ $(C_{28}H_{39}FNO_3)^+$ requires *m/z* 456.2909, found *m/z* 456.2908.

1-(1-(3-(Dimethylamino)propyl)-1-(4-fluorophenyl)-1,3-dihydroisobenzofuran-5-yl)-4-hydroxynonan-1-one (2z)

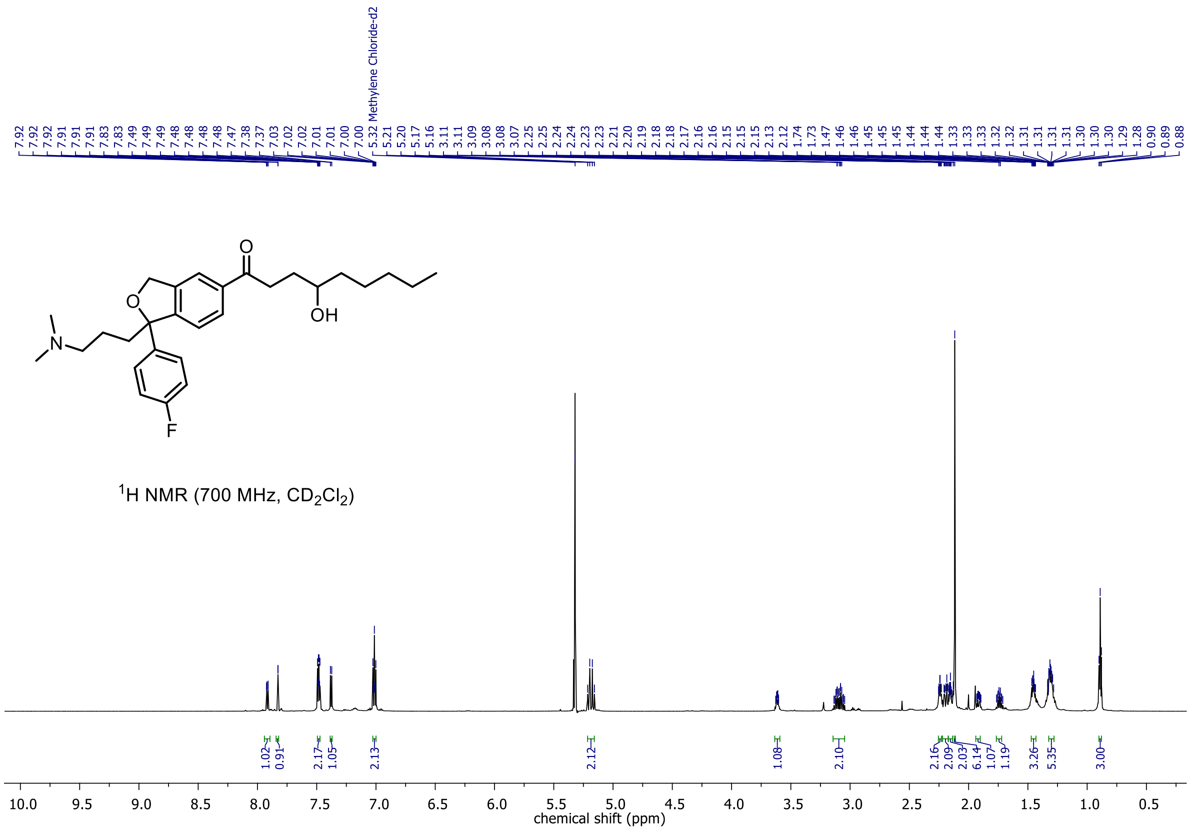

^1H NMR (700 MHz, CD_2Cl_2)

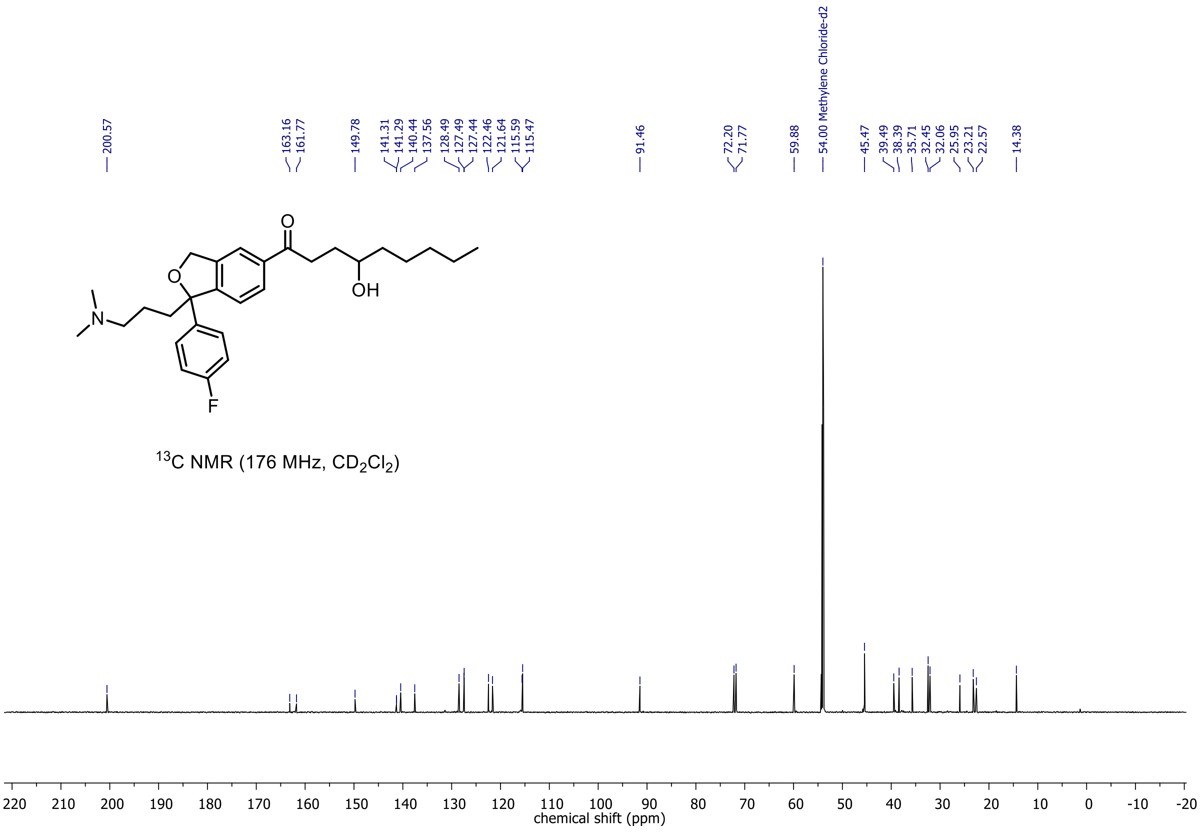

^{13}C NMR (176 MHz, CD_2Cl_2)

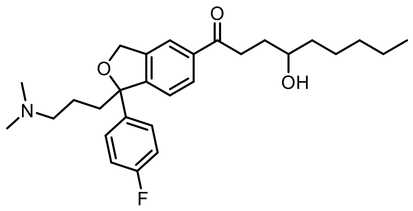

^{19}F NMR (659 MHz, CD_2Cl_2)

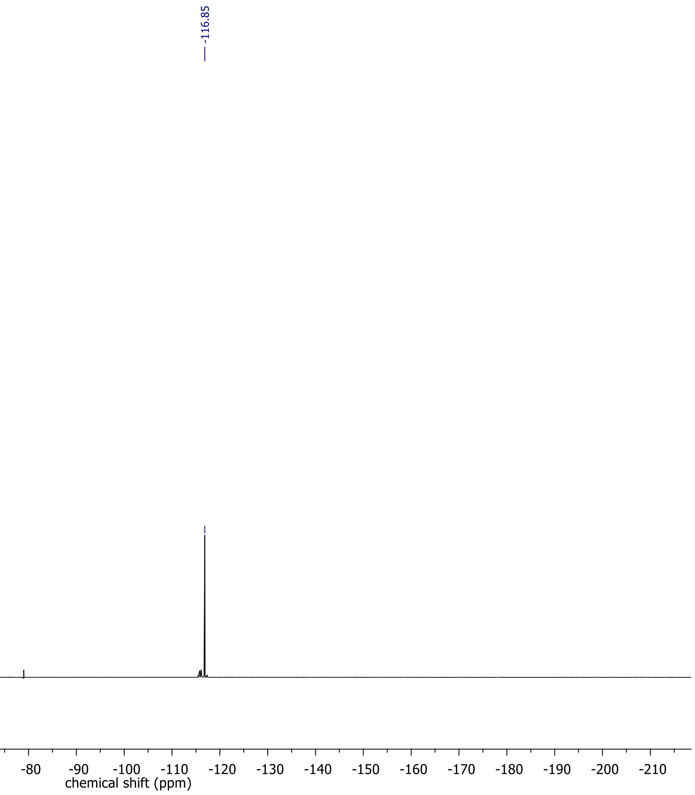

2.6 Unsuccessful examples

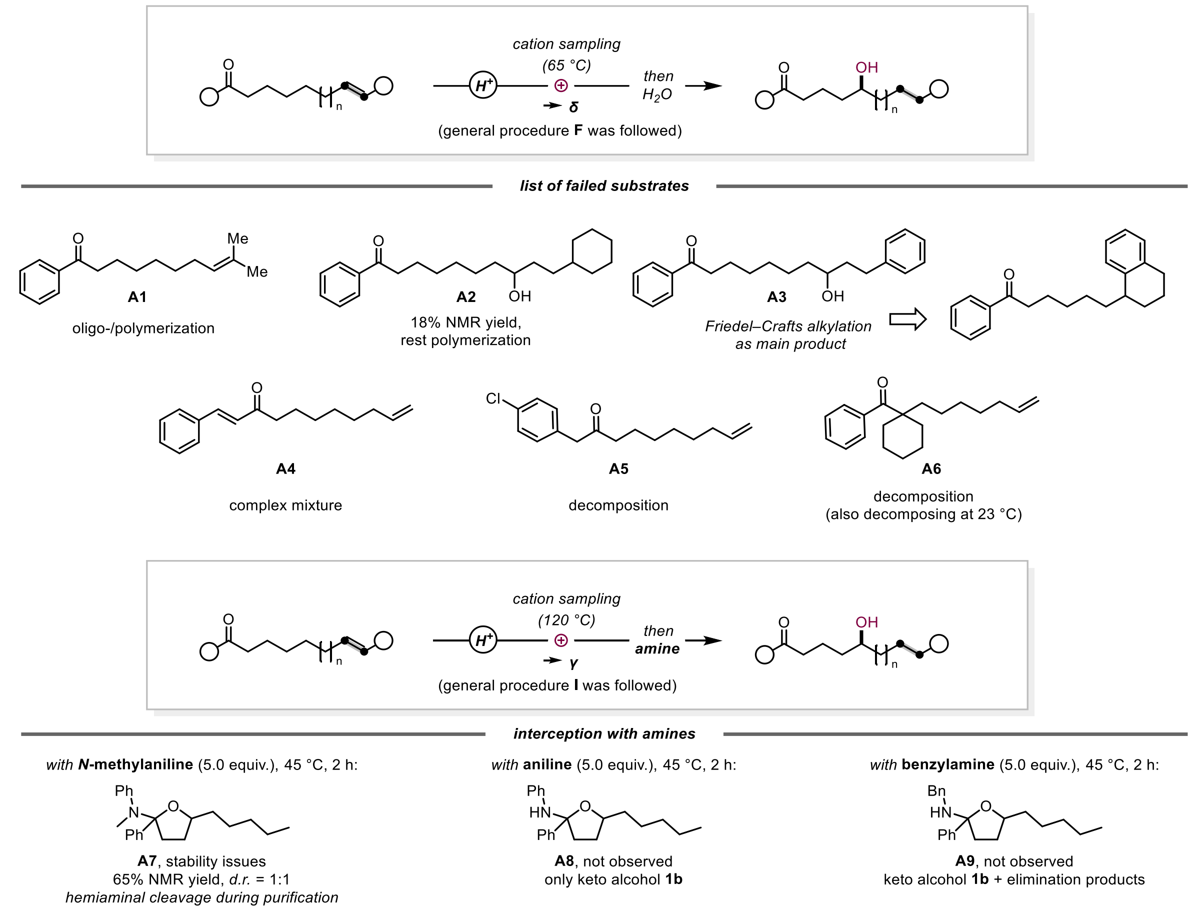

Figure S5. List of failed reactions.

As illustrated in Figure S5, issues were encountered with a few tested substrates. One challenge is that undesired oligo- or polymerization can occur, particularly with substrates where the migration of the positive charge is slow, *e.g.*, in cases where tertiary carbocations can be formed. This was observed in the case of a trisubstituted olefin (A1) and in an example where the alkyl chain was significantly elongated at the other end (A2), potentially forming a tertiary carbocation upon migrating to the undesired side of the chain (though the desired product was still observed). In a single case (A3), we observed that the positive charge was intercepted along the migration path, leading to Friedel–Crafts alkylation. For the substrate A4, a complex mixture was observed, likely due to competing protonation of the styrene. For A5 and A6, the materials fully decomposed under the reaction conditions. In the case of A5, we believe that the increased electron density on the aromatic ring results in the formation of intermolecular Friedel–Crafts products (complex mixture, due to preceding positive charge migration). In the case of A6, which decomposed even at 23 °C in the presence of TfOH, the ketone does not appear to offer sufficient stabilization of the positive charge, resulting in alternative unspecific reaction pathways.

Amines were also evaluated as alternative reagents for intercepting oxocarbenium ions. While *N*-methylaniline afforded hemiaminal **A7** in 65% NMR yield, cleavage of the hemiaminal was observed during column chromatography. Aniline did not lead to a productive reaction, since only hydrolysis product (keto alcohol **1b**) was observed in the crude reaction product. Benzylamine led to the formation of a mixture of **1b** and olefin products arising from deprotonation of the oxocarbenium species.

2.7 Large-scale synthesis of δ-alcohol 1b

5-Hydroxy-1-phenylnonan-1-one (1b)

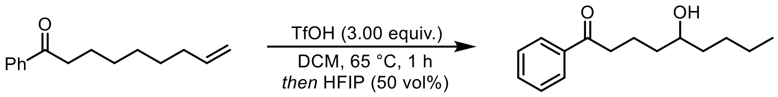

In a large thick-walled Schlenk flask (see Section 2.1.4) ketone 1-phenylnon-8-en-1-one (433 mg, 2.00 mmol, 1.00 equiv.) was dissolved in DCM (20 mL) at 23 °C. TfOH (0.53 mL, 6.00 mmol, 3.00 equiv.) was added with a glass syringe (see Section 2.1.5) in one portion. The mixture was stirred in a pre-heated oil bath (65 °C oil temperature, flask immersed to ~40% of total volume, see Section 2.1.4) for 1 h, after which the oil bath was removed, and the reaction mixture was cooled to 0 °C in an ice bath. The flask was opened and HFIP (10 mL) was added under stirring. The resulting dark-red solution was stirred for 10 min under air, after which an aqueous saturated solution of $NaHCO_3$ (25 mL) was added. The cooling bath was removed, and the biphasic mixture was vigorously stirred for 30 min at 23 °C and then poured into a 250 mL separatory funnel. The organic phase was separated, and the aqueous phase was extracted with DCM (3 × 20 mL). The combined organic layers were washed with 3 M NaOH[a] (50 mL) and then dried over anhydrous sodium sulfate. The dried solution was filtered, the filtrate was concentrated under reduced pressure (40 °C water bath) and the crude residue was purified by flash column chromatography (5 – 40% EtOAc in heptanes with 1% triethylamine additive), affording the title compound (403 mg, 1.72 mmol, 86%) as a colorless solid and single regioisomer.

[a]This step serves to remove HFIP from the mixture, due to its toxic effects. It can be omitted, if evaporating HFIP is not an issue.

Crude product: 91% NMR yield, r.r. (δ_{OH}:γ_{OH}) = 92:8.

All analytical data were identical with those reported in Section 2.4 (product **1b**).

2.8 Large-scale synthesis of γ-alcohol 2b

4-Hydroxy-1-phenylnonan-1-one (2a)

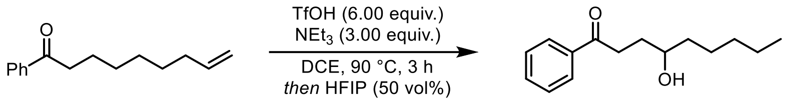

In a large thick-walled Schlenk flask (see Section 2.1.4) ketone 1-phenylnon-8-en-1-one (433 mg, 2.00 mmol, 1.00 equiv.) was dissolved in DCE (20 mL) at 23 °C. Triethylamine (0.84 mL, 6.00 mmol, 3.00 equiv.) was added, followed by TfOH (1.06 mL, 12.00 mmol, 6.00 equiv.), which was added with a glass syringe (see Section 2.1.5) in one portion. The mixture was stirred in a pre-heated oil bath (90 °C oil temperature, flask immersed to ~40% of total volume, see Section 2.1.4) for 3 h, after which the oil bath was removed, and the reaction mixture was cooled to 0 °C in an ice bath. The flask was opened and HFIP (10 mL) was added under stirring. The resulting dark-red solution was stirred for 10 min under air, after which an aqueous saturated solution of $NaHCO_3$ (25 mL) was added. The cooling bath was removed, and the biphasic mixture was vigorously stirred for 30 min at 23 °C and then poured into a 250 mL separatory funnel. The organic phase was separated and the aqueous phase was extracted with DCM (3 × 20 mL). The combined organic layers were washed with 3 M NaOH[a] (50 mL), dried over anhydrous sodium sulfate, the dried solution was filtered and the filtrate was concentrated under reduced pressure (40 °C water bath). The crude residue was purified by flash column chromatography (5 – 35% EtOAc in heptanes with 1% triethylamine additive), affording the title compound (307 mg, 1.31 mmol, 66%) as a colorless solid a single regioisomer.

[a]This step serves to remove HFIP from the mixture, due to its toxic effects. It can be omitted, if evaporating HFIP is not an issue.

Crude product: 71% NMR yield, r.r. (γ_{OH}:δ_{OH}) > 95:5

All analytical data were identical with those reported in Section 2.5 (product **2b**).

3. Alternative Interceptions of oxocarbenium ion

3.1 With halides

4-Iodo-1-phenylnonan-1-one (6)

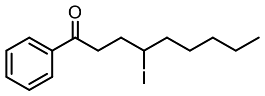

Following general procedure I, 1-phenylnon-8-en-1-one (21.6 mg, 0.10 mmol, 1.00 equiv.) was treated with TfOH (18 µL, 0.20 mmol, 2.00 equiv.) and heated to 120 °C for 3 h in DCE, providing a mixture that was subsequently reacted with tetrabutylammonium iodide (185 mg, 0.50 mmol, 5.00 equiv.) at 35 °C for 2 h. Purification by flash column chromatography (0–20% EtOAc in heptanes) afforded the title compound (20.2 mg, 59 µmol, 59%) as a red oil and a single regioisomer.

Crude product: 66% NMR yield, r.r. (γ_{OH}:δ_{OH}) > 95:5.

^{1}H NMR (400 MHz, $CDCl_3$): δ 8.03 – 7.94 (m, 2H), 7.61 – 7.54 (m, 1H), 7.51 – 7.42 (m, 2H), 4.29 – 4.17 (m, 1H), 3.33 – 3.12 (m, 2H), 2.27 – 2.13 (m, 2H), 2.02 – 1.90 (m, 1H), 1.83 – 1.74 (m, 1H), 1.55 – 1.30 (m, 6H), 0.90 (t, *J* = 6.9 Hz, 3H).

^{13}C NMR (101 MHz, $CDCl_3$): δ 199.1, 136.9, 133.3, 128.8 (2C), 128.2 (2C), 41.2, 40.0, 39.0, 34.8, 31.1, 29.4, 22.6, 14.2.

IR (neat) ν_{max}: 2926, 2857, 1684, 1597, 1448, 1371, 1262, 1216, 1180, 1002, 975, 911, 741, 689.

HRMS (ESI$^+$): exact mass calculated for $[M+Na]^+$ $(C_{15}H_{21}IONa)^+$ requires *m/z* 367.0529, found *m/z* 367.0532.

4-Iodo-1-phenylnonan-1-one (6)

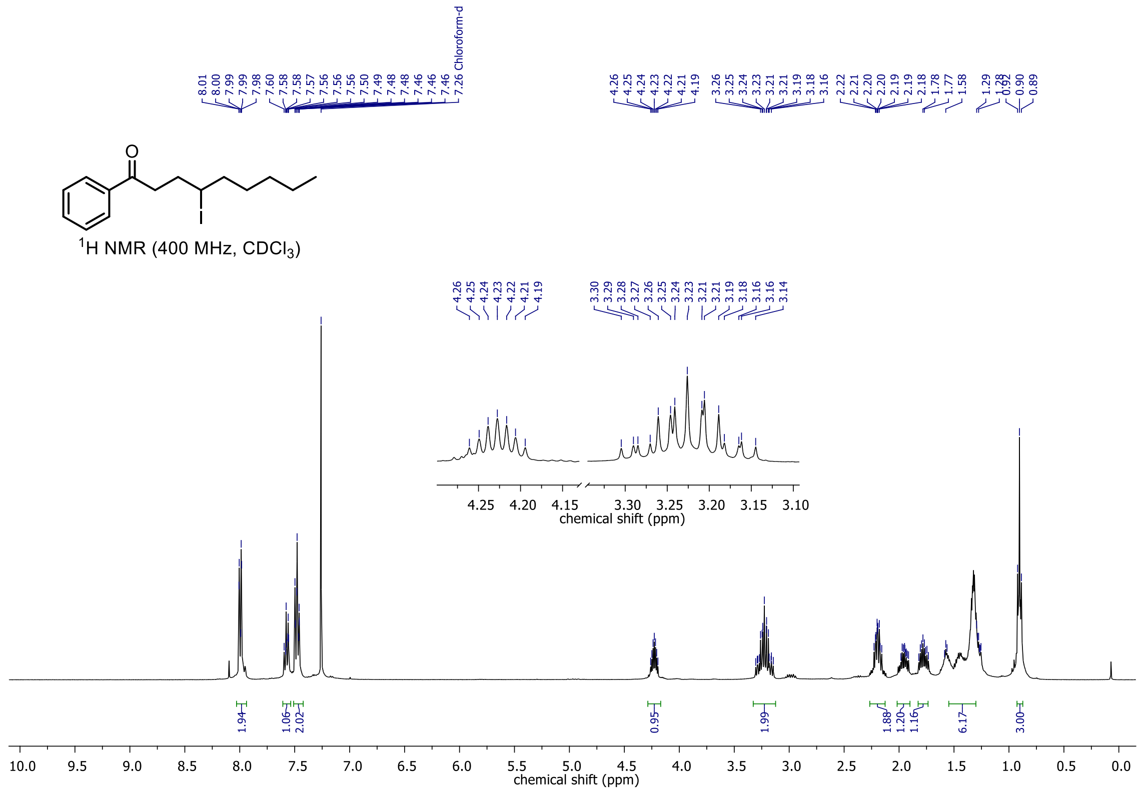

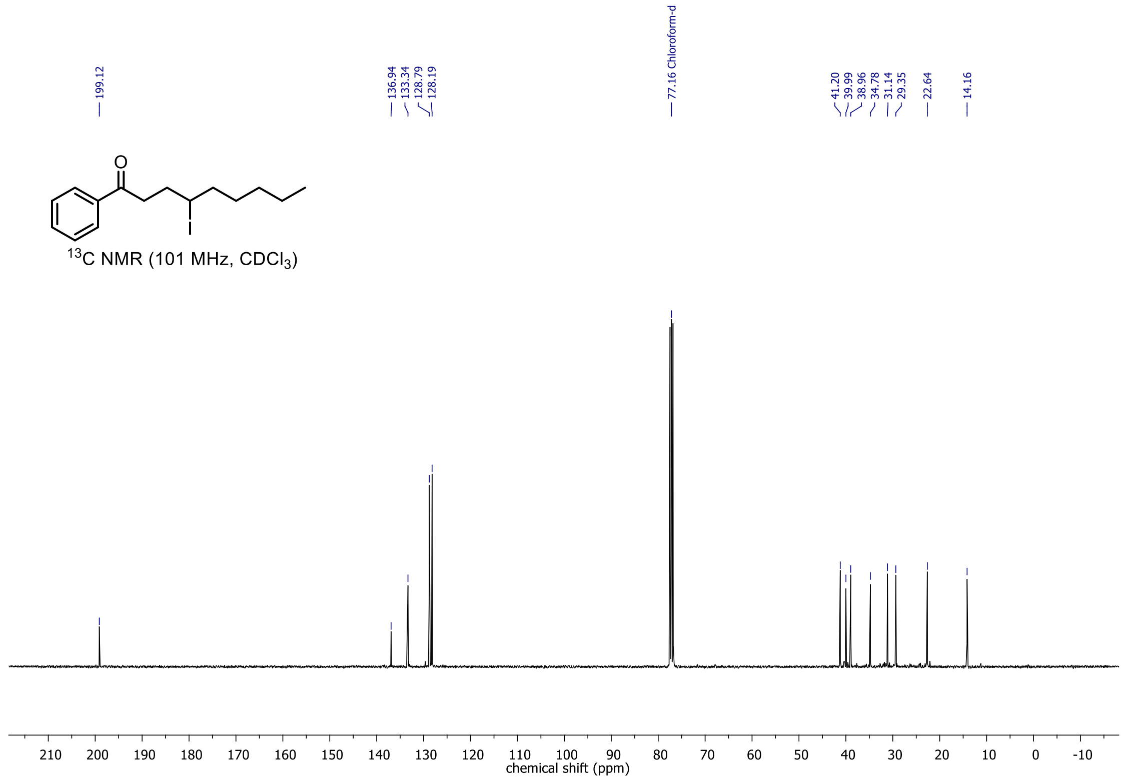

4-Chloro-1-phenylnonan-1-one (8)

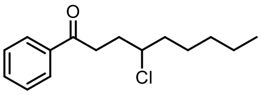

Following general procedure I, 1-phenylnon-8-en-1-one (21.6 mg, 0.10 mmol, 1.00 equiv.) was treated with TfOH (17.7 μL, 0.20 mmol, 2.00 equiv.) and heated to 120 °C for 3 h in DCE, providing a mixture that was subsequently reacted with HCl in diethyl ether (1.0 M, 0.50 mL, 0.50 mmol, 5.00 equiv.) at 45 °C for 16 h. Purification by flash column chromatography (0 – 20% EtOAc in heptanes) afforded the title compound (15.0 mg, 59 μmol, 59%) as an orange oil and a single regioisomer.

Crude product: 57% NMR yield, r.r. (γ_{Cl}:δ_{Cl}) > 95:5.

^{1}H NMR (400 MHz, CDCl$_3$): δ 7.99 (dd, *J* = 5.2, 3.3 Hz, 2H), 7.61 – 7.52 (m, 1H), 7.47 (dd, *J* = 10.4, 4.7 Hz, 2H), 4.08 – 3.92 (m, 1H), 3.37 – 3.10 (m, 2H), 2.40 – 2.23 (m, 1H), 2.08 – 1.94 (m, 1H), 1.85 – 1.75 (m, 2H), 1.62 – 1.53 (m, 1H), 1.49 – 1.41 (m, 1H), 1.37 – 1.28 (m, 4H), 0.95 – 0.89 (m, 3H).

^{13}C NMR (101 MHz, CDCl$_3$): δ 199.5, 137.0, 133.3, 128.8 (2C), 128.2 (2C), 63.9, 39.0, 35.6, 32.7, 31.5, 26.4, 22.7, 14.1.

IR (neat) ν_{max}: 2933, 2858, 1682, 1447, 1226, 742, 689.

HRMS (ESI$^+$): exact mass calculated for [M+Na]$^+$ ($C_{15}H_{21}OClNa$)$^+$ requires *m/z* 275.1173, found *m/z* 275.1171.

4-Chloro-1-phenylnonan-1-one (8)

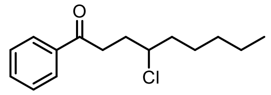

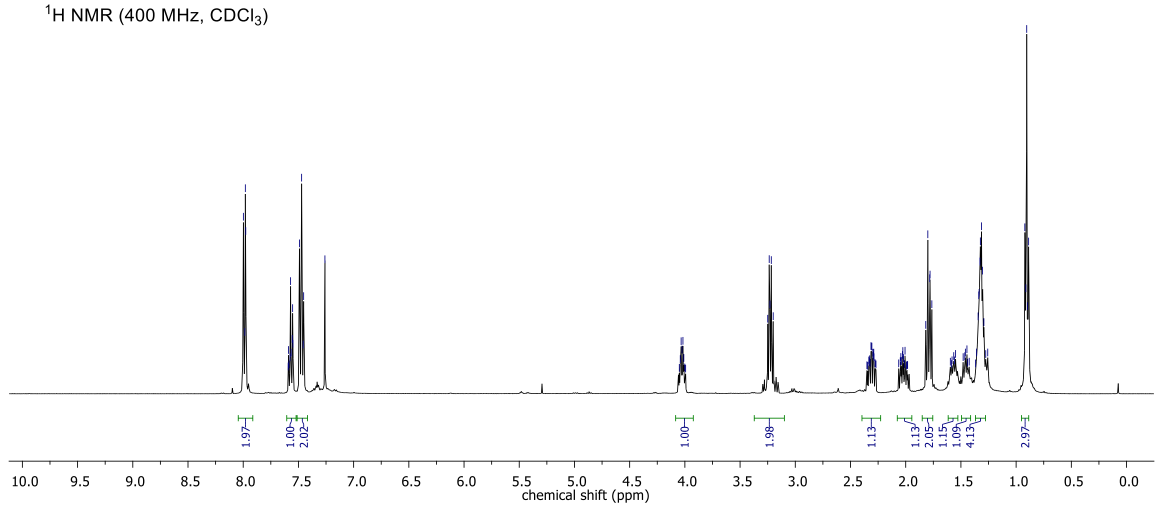

^{1}H NMR (400 MHz, $CDCl_3$)

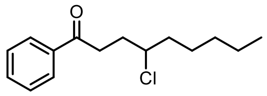

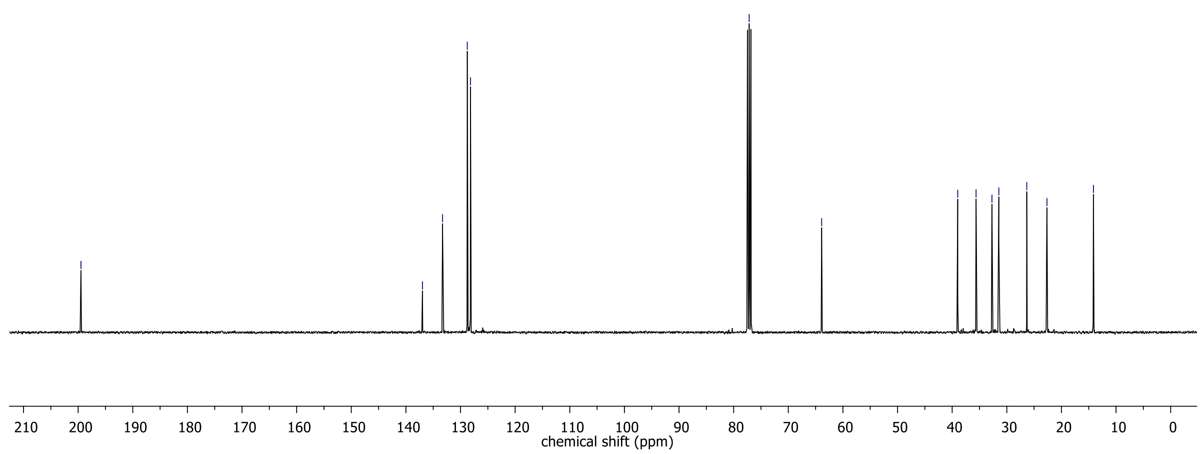

^{13}C NMR (101 MHz, $CDCl_3$)

4-Bromo-1-phenylnonan-1-one (7)

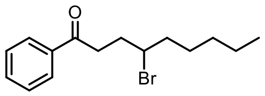

Following general procedure I, 1-phenylnon-8-en-1-one (21.6 mg, 0.10 mmol, 1.00 equiv.) was treated with TfOH (17.7 µL, 0.20 mmol, 2.00 equiv.) and heated to 120 °C for 3 h in DCE, providing a mixture that was subsequently reacted with tetrabutylammonium bromide (161 mg, 0.50 mmol, 5.00 equiv.) at 35 °C for 2 h. Purification by flash column chromatography (0 – 20% EtOAc in heptanes) afforded the title compound (19.2 mg, 65 µmol, 65%) as a yellow oil and a single regioisomer.

Crude product: 65% NMR yield, r.r. (γ_{Br}:δ_{Br}) > 95:5.

^{1}H NMR (400 MHz, CDCl$_3$): δ 8.05 – 7.88 (m, 2H), 7.57 (t, *J* = 7.4 Hz, 1H), 7.47 (t, *J* = 7.6 Hz, 2H), 4.25 – 4.06 (m, 1H), 3.34 – 3.15 (m, 2H), 2.45 – 2.29 (m, 1H), 2.20 – 2.08 (m, 1H), 1.97 – 1.84 (m, 2H), 1.63 – 1.28 (m, 6H), 0.90 (t, *J* = 6.8 Hz, 3H).

^{13}C NMR (101 MHz, CDCl$_3$): δ 199.3, 137.0, 133.3, 128.8 (2C), 128.2 (2C), 58.5, 39.7, 36.8, 33.3, 31.4, 27.4, 22.7, 14.2.

IR (neat) ν_{max}: 2955, 2928, 2858, 1686, 1448, 1266, 1222, 742, 690.

HRMS (ESI$^+$): exact mass calculated for $[M+Na]^+$ ($C_{15}H_{21}{}^{79}BrONa$)$^+$ requires *m/z* 319.0668, found *m/z* 319.0669.

4-Bromo-1-phenylnonan-1-one (7)

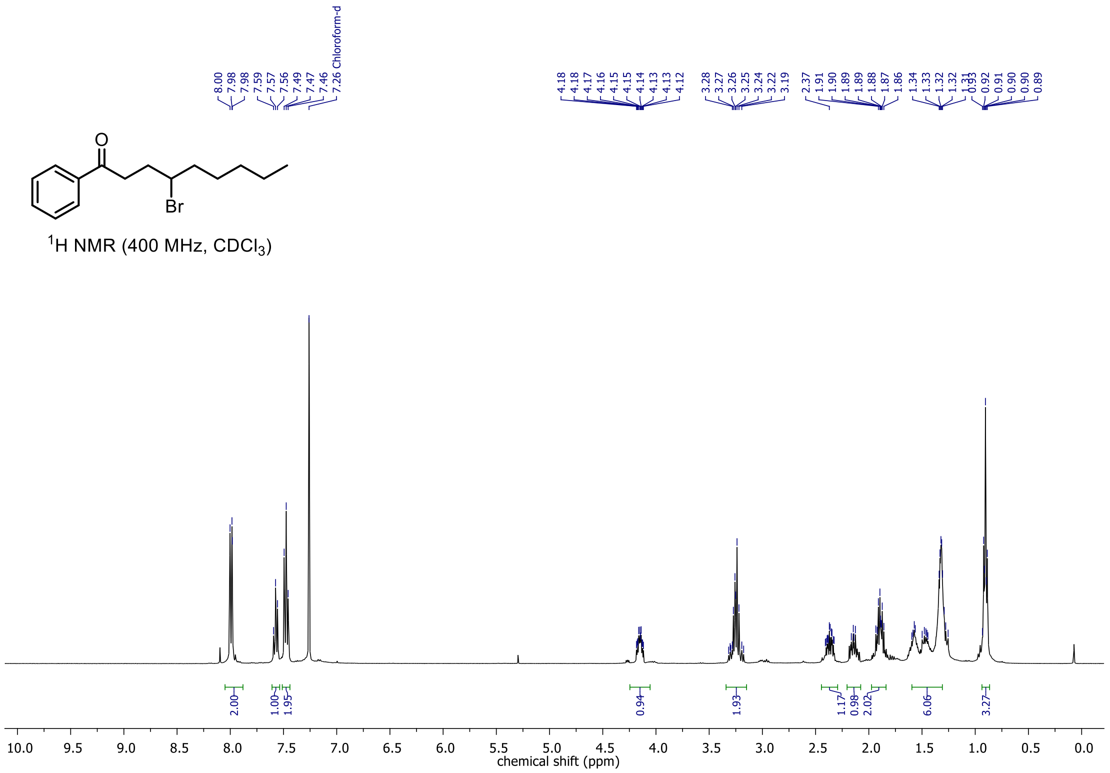

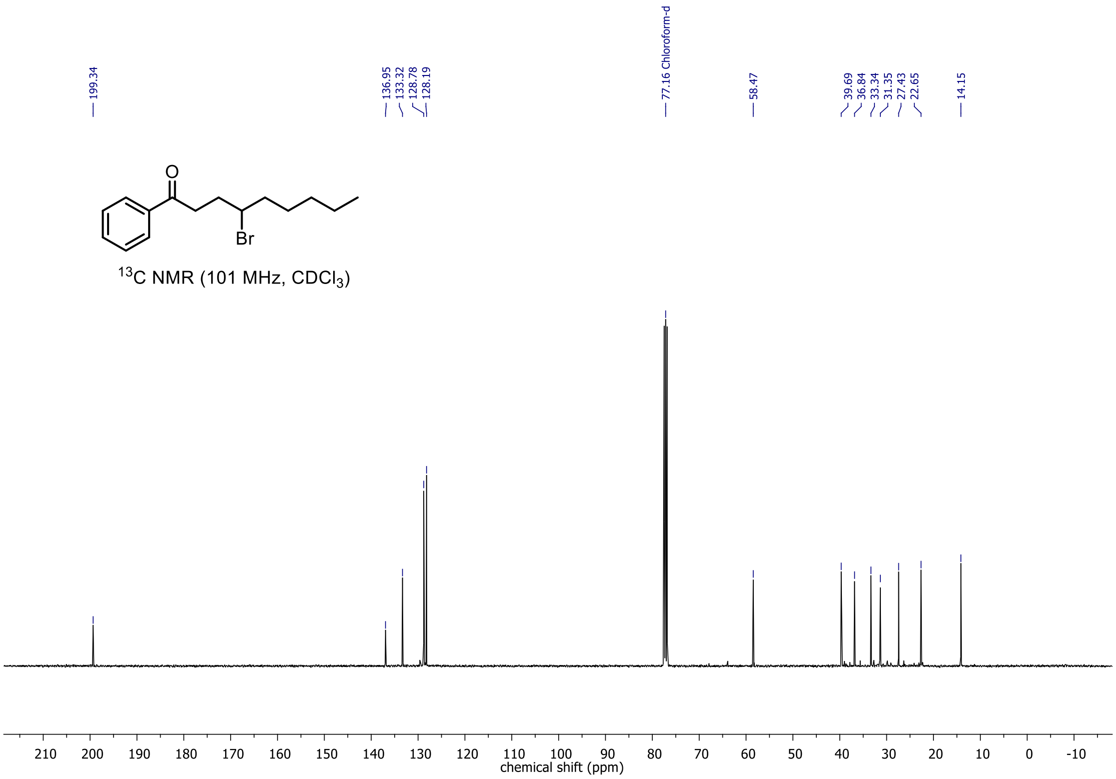

5-Iodo-1-(4-methoxyphenyl)nonan-1-one (3)

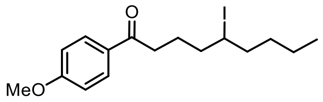

Following general procedure I, 1-(4-methoxyphenyl)non-8-en-1-one (24.6 mg, 0.10 mmol, 1.00 equiv.) was treated with TfOH (18 µL, 0.20 mmol, 2.00 equiv.) and heated to 65 °C for 7 h in DCM, providing a mixture that was subsequently reacted with tetrabutylammonium iodide (111 mg, 0.30 mmol, 3.00 equiv.) at 23 °C for 1 h. Purification by flash column chromatography (0 – 20% EtOAc in heptanes) afforded the title compound (30.0 mg, 80 µmol, 80%) as a colorless oil and a single regioisomer.

Crude product: 92% NMR yield, r.r. (δ_I:γ_I) = 95:5.

^{1}H NMR (400 MHz, $CDCl_3$) δ 7.94 (d, *J* = 8.2 Hz, 2H), 6.93 (d, *J* = 8.2 Hz, 2H), 4.15 (tt, *J* = 8.4, 4.5 Hz, 1H), 3.87 (s, 3H), 3.03 – 2.84 (m, 2H), 2.03 – 1.67 (m, 6H), 1.55 – 1.46 (m, 1H), 1.42 – 1.27 (m, 3H), 0.91 (t, *J* = 7.1 Hz, 3H).

^{13}C NMR (101 MHz, $CDCl_3$) δ 198.5, 163.6, 130.5 (2C), 130.1, 113.9 (2C), 55.6, 40.4, 40.3, 39.7, 37.3, 31.8, 24.6, 22.1, 14.1.

IR (neat) ν_{max}: 2955, 2929, 2870, 2858, 1675, 1600, 1575, 1509, 1457, 1308, 1257, 1169, 1030, 829.

HRMS (ESI$^+$): exact mass calculated for $[M+H]^+$ $(C_{16}H_{24}O_2{}^{127}I)^+$ requires *m/z* 375.0816, found *m/z* 375.0826.

5-Iodo-1-(4-methoxyphenyl)nonan-1-one (3)

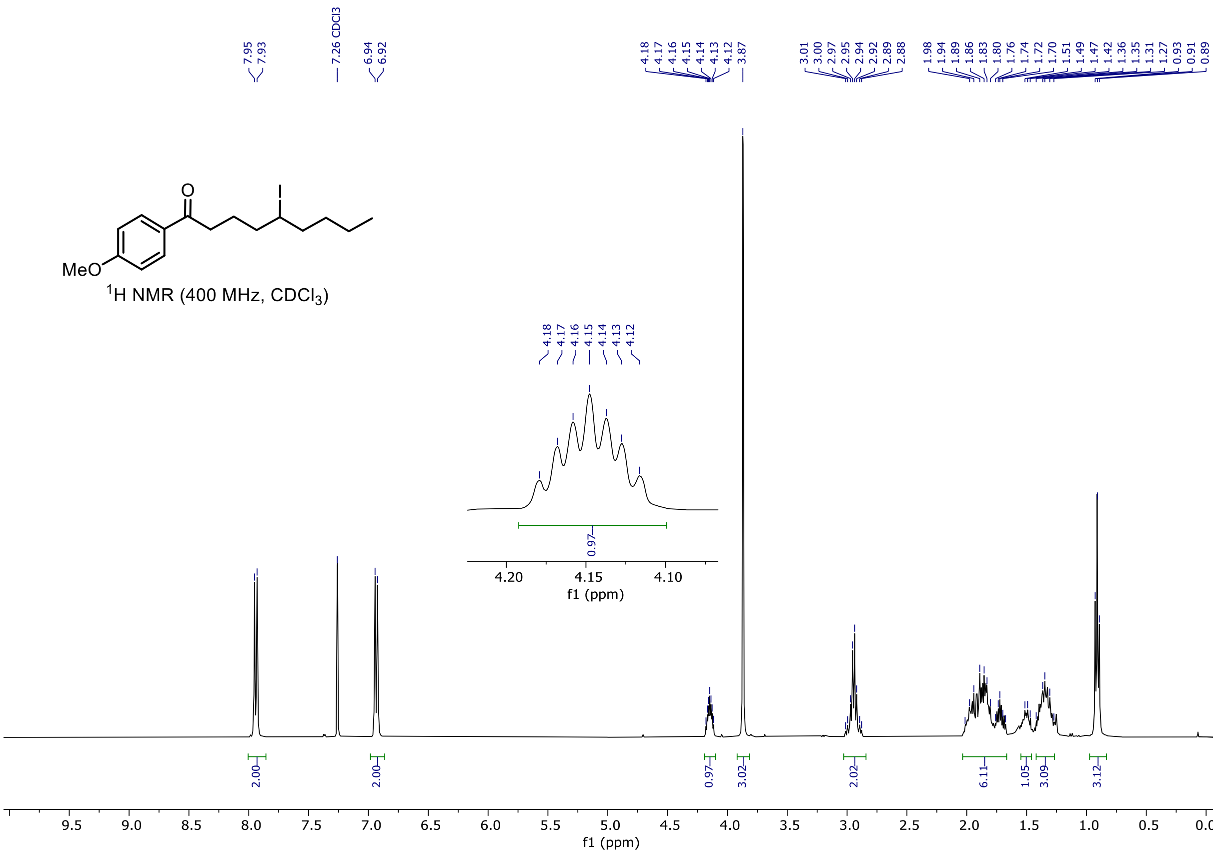

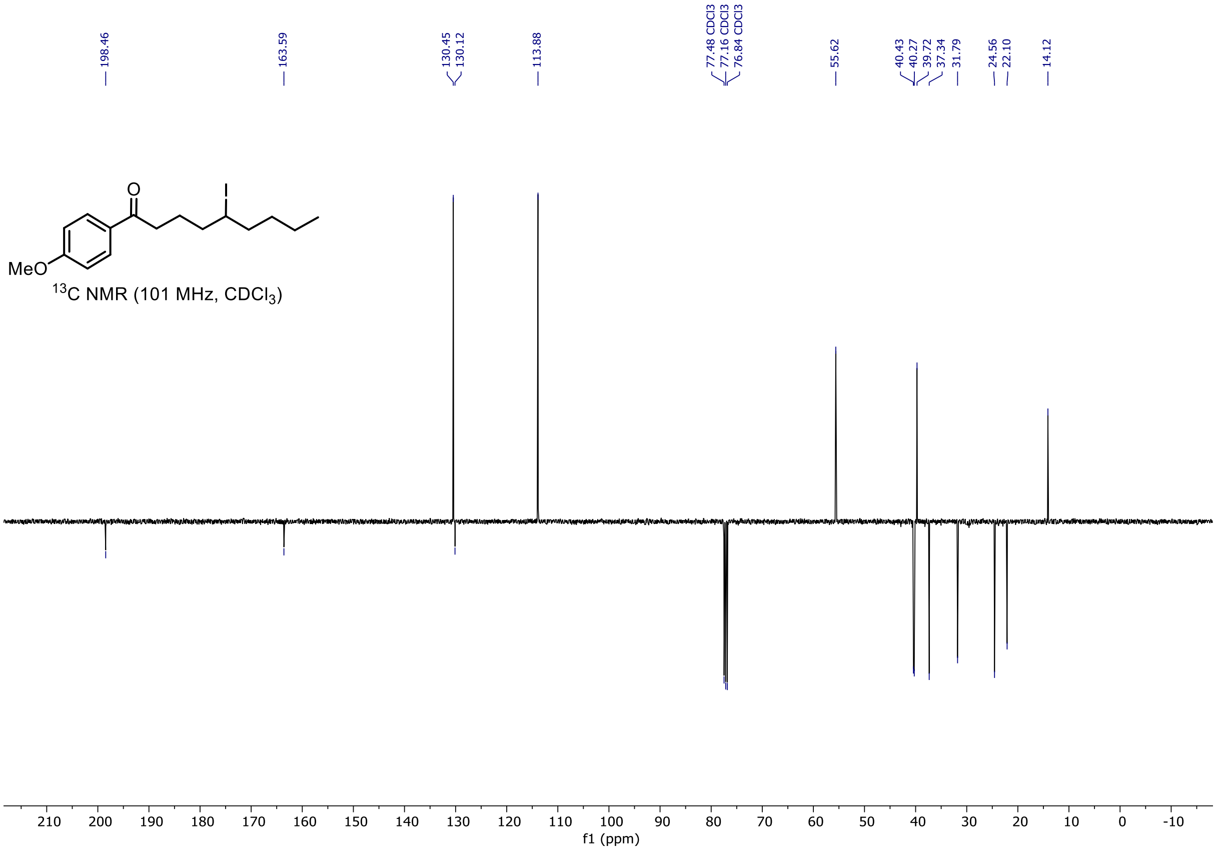

5-Bromo-1-(4-methoxyphenyl)nonan-1-one (4)

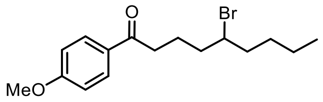

Following general procedure I, 1-(4-methoxyphenyl)non-8-en-1-one (24.6 mg, 0.10 mmol, 1.00 equiv.) was treated with TfOH (18 µL, 0.20 mmol, 2.00 equiv.) and heated to 65 °C for 7 h in DCM, providing a mixture that was subsequently reacted with tetrabutylammonium bromide (97 mg, 0.30 mmol, 3.00 equiv.) at 23 °C for 1 h. Purification by flash column chromatography (0 – 20% EtOAc in heptanes) afforded the title compound (26.3 mg, 80 µmol, 80%) as a colorless oil and a single regioisomer.

Crude product: 91% NMR yield, r.r. (δ_{Br}:γ_{Br}) = 95:5.

^{1}H NMR (400 MHz, CDCl$_3$) δ 7.94 (d, *J* = 8.7 Hz, 2H), 6.94 (d, *J* = 8.7 Hz, 2H), 4.07 (p, *J* = 7.1 Hz, 1H), 3.87 (s, 3H), 3.03 – 2.87 (m, 2H), 2.05 – 1.75 (m, 6H), 1.55 – 1.23 (m, 4H), 0.91 (t, *J* = 7.2 Hz, 3H).

^{13}C NMR (101 MHz, CDCl$_3$) δ 198.5, 163.6, 130.4 (2C), 130.1, 113.9 (2C), 58.4, 55.6, 39.0, 38.7, 37.5, 29.8, 22.6, 22.3, 14.1.

IR (neat) ν_{max}: 2955, 2932, 2871, 2860, 1676, 1600, 1575, 1509, 1308, 1257, 1170, 1030.

HRMS (ESI$^+$): exact mass calculated for $[M+H]^+$ $(C_{16}H_{24}O_2{}^{79}Br)^+$ requires *m/z* 327.0954, found *m/z* 327.0963.

5-Bromo-1-(4-methoxyphenyl)nonan-1-one (4)

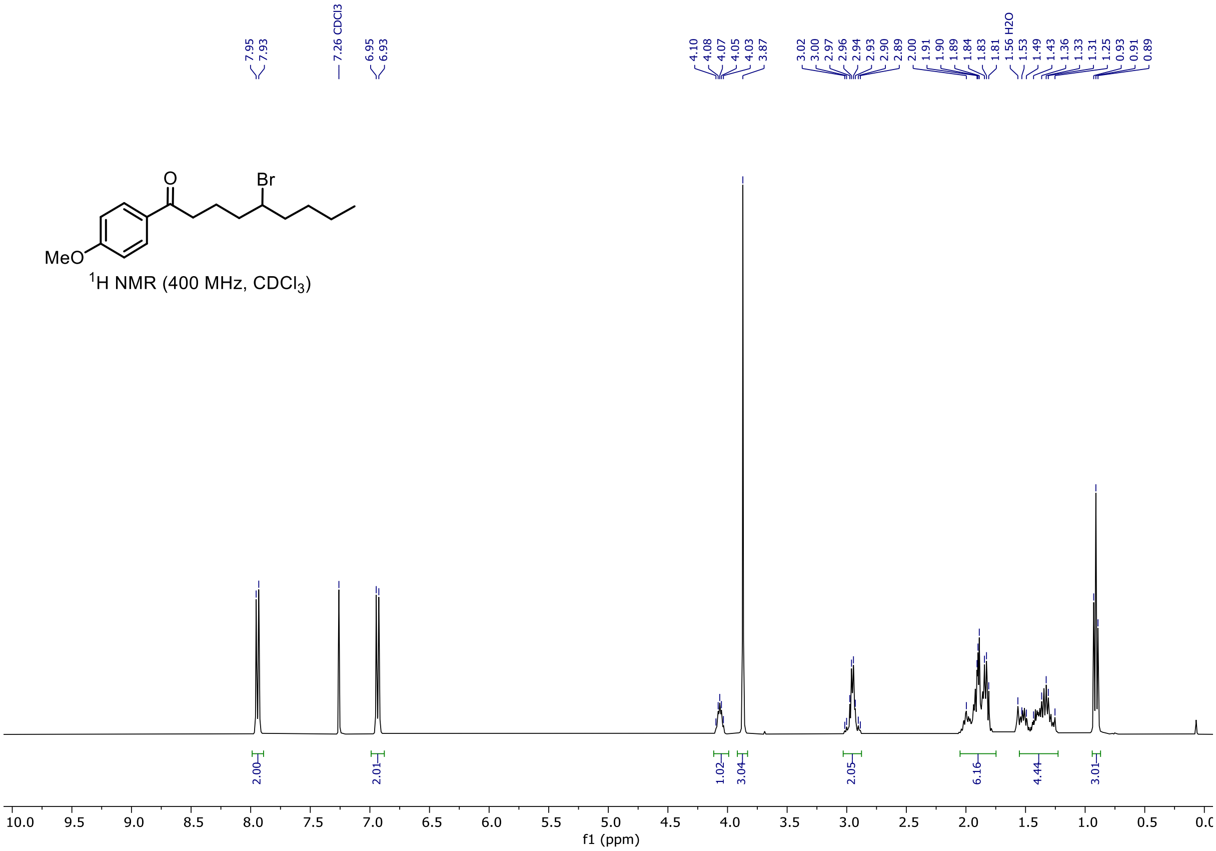

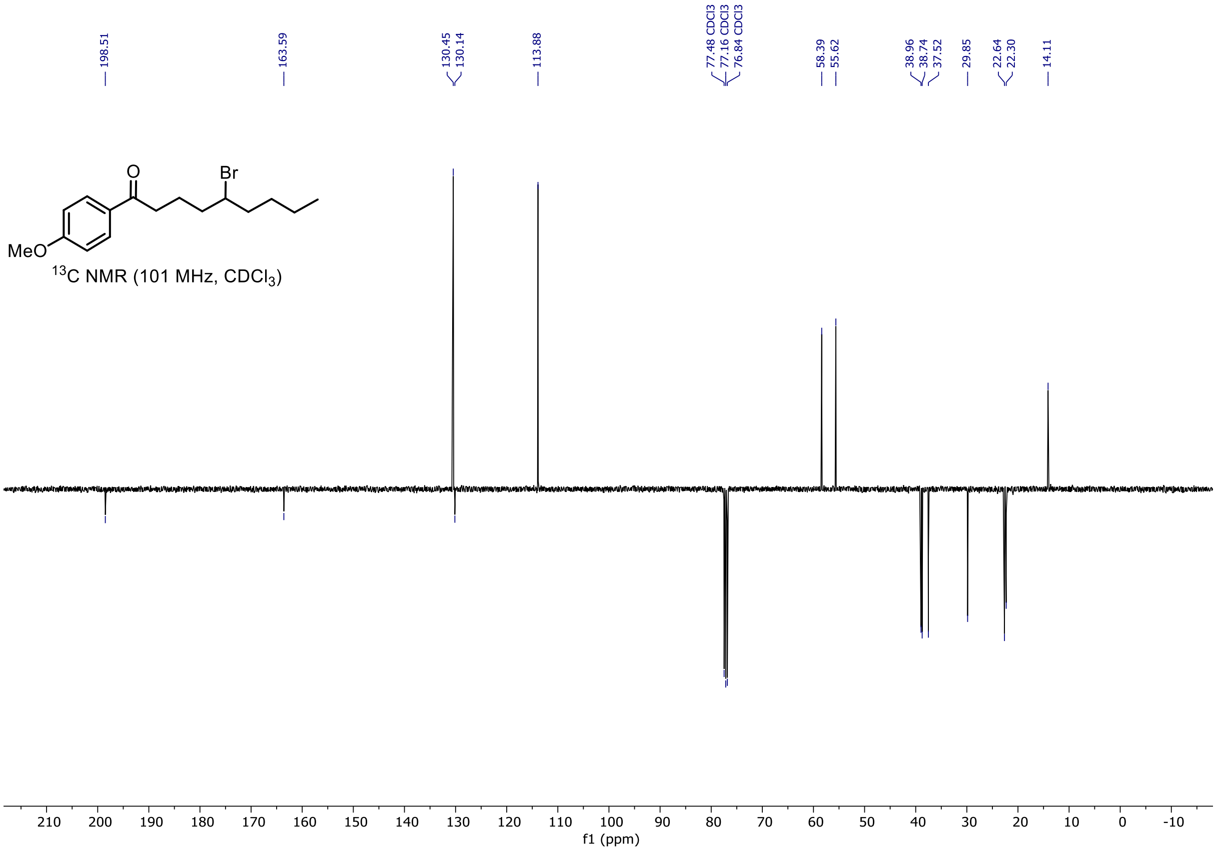

5-Chloro-1-(4-methoxyphenyl)nonan-1-one (5)

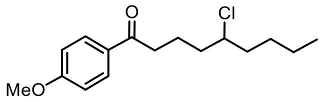

Following general procedure I, 1-(4-methoxyphenyl)non-8-en-1-one (24.6 mg, 0.10 mmol, 1.00 equiv.) was treated with TfOH (18 µL, 0.20 mmol, 2.00 equiv.) and heated to 65 °C for 7 h in DCM, providing a mixture that was subsequently reacted with tetrabutylammonium chloride[a] (83 mg, 0.30 mmol, 3.00 equiv.) at 45 °C for 16 h. Purification by flash column chromatography (0 – 20% EtOAc in heptanes) afforded the title compound (22.0 mg, 78 µmol, 78%) as a colorless oil and a single regioisomer.

[a]The salt is strongly hygroscopic and has to be added quickly after weighing. Alternatively, a stock solution of TBACl in anhydrous DCM (83 mg in 0.5 mL) can be prepared under inert conditions and then added into the reaction mixture.

Crude product: 88% NMR yield, r.r. (δ_{Cl}:γ_{Cl}) = 94:6.

^{1}H NMR (400 MHz, $CDCl_3$) δ 8.01 – 7.88 (m, 2H), 7.00 – 6.87 (m, 2H), 3.94 (tt, *J* = 8.4, 4.4 Hz, 1H), 3.87 (s, 3H), 3.01 – 2.86 (m, 2H), 2.04 – 1.68 (m, 6H), 1.56 – 1.46 (m, 1H), 1.45 – 1.26 (m, 3H), 0.91 (t, *J* = 7.2 Hz, 3H).

^{13}C NMR (176 MHz, $CDCl_3$) δ 198.6, 163.6, 130.4 (2C), 130.2, 113.9 (2C), 64.0, 55.6, 38.3, 38.1, 37.6, 28.8, 22.4, 21.6, 14.1.

IR (neat) ν_{max}: 2955, 2934, 2861, 1676, 1599, 1575, 1509, 1308, 1257, 1170, 1030.

HRMS (ESI$^+$): exact mass calculated for $[M+Na]^+$ $(C_{16}H_{23}O_2ClNa)^+$ requires *m/z* 283.1459, found *m/z* 283.1461.

5-Chloro-1-(4-methoxyphenyl)nonan-1-one (5)

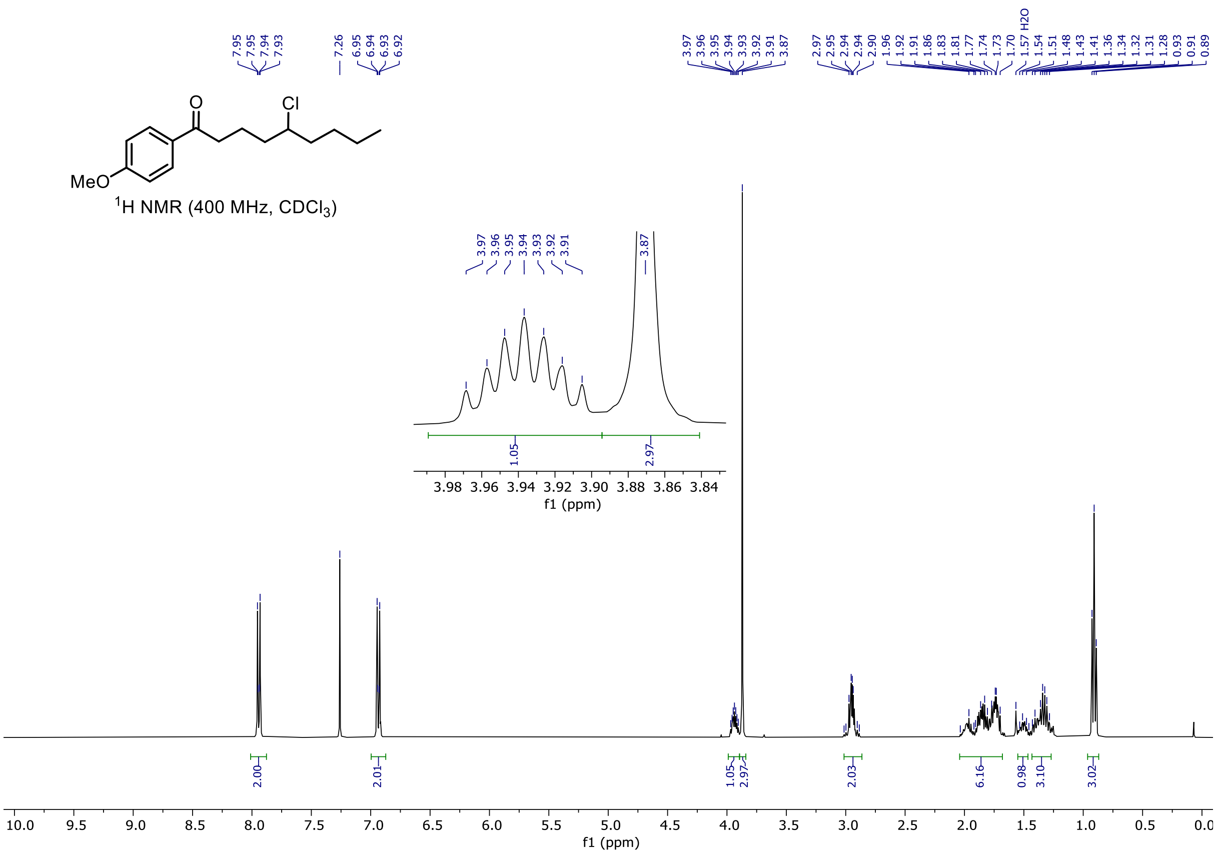

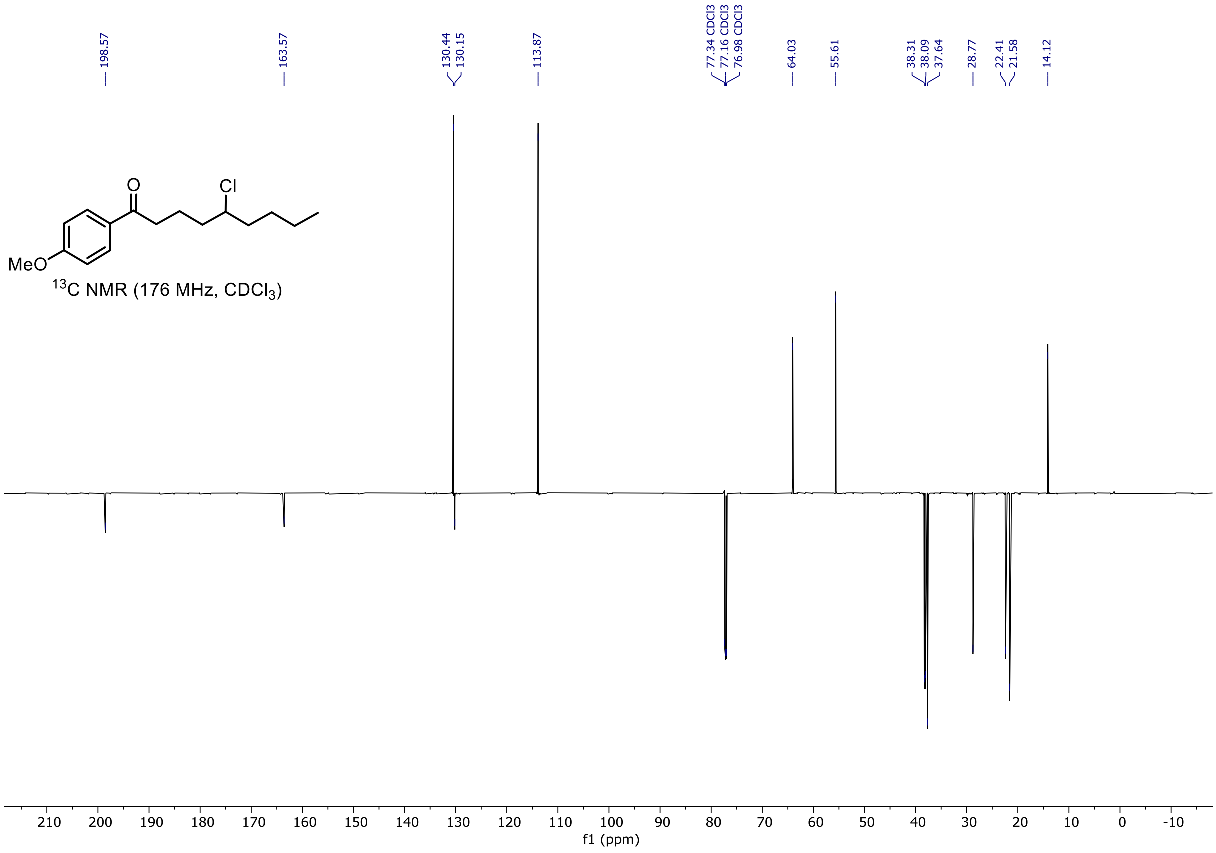

3.2 With reductants

***cis*-2-Butyl-6-phenyltetrahydro-2*H*-pyran (9)**

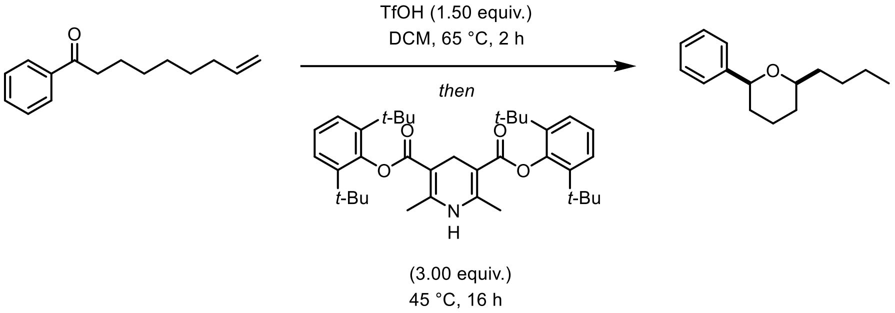

A 4 mL vial was charged with 1-phenylnon-8-en-1-one (32.4 mg, 0.15 mmol, 1.00 equiv.) and DCM (1.5 mL, 0.1 M). TfOH (20 µL, 0.23 mmol, 1.50 equiv.) was then added at 23 °C using a microsyringe. The vial was sealed with a screwcap and placed into a metal heating block (pre-heated to 65 °C, vial fully immersed, see Section 2.1.4), where the solution was stirred at 65 °C for 2 h. Subsequently, the mixture was cooled in a cold-water bath (10 – 15 °C) for 5 min, after which the Hantzsch ester reducing agent (258 mg, 0.45 mmol, 3.00 equiv.) was slowly added. The mixture was heated to 45 °C for 16 h. Upon completion, an aqueous saturated $NaHCO_3$ solution (2 mL) was added. The mixture was transferred into a separatory funnel, the phases were separated, and the aqueous phase was extracted with DCM (2 × 5 mL). The combined organic layers were dried over anhydrous magnesium sulfate. The dried solution was filtered, and the filtrate was concentrated under reduced pressure. The crude residue was purified using flash column chromatography (0 – 10% Et_2O in heptanes), affording the title compound as a colorless liquid (16.5 mg, 75 µmol, 50%) and as a single regio-/diastereomer.

Crude product: 72% NMR yield, r.r. (δ:γ) = 85:15, d.r. >95:5.

^{1}H NMR (400 MHz, $CDCl_3$): δ 7.39 – 7.30 (m, 4H), 7.25 – 7.21 (m, 1H), 4.36 (dd, *J* = 11.2, 1.9 Hz, 1H), 3.51 – 3.41 (m, 1H), 1.98 – 1.90 (m, 1H), 1.88 – 1.80 (m, 1H), 1.70 – 1.59 (m, 3H), 1.51 – 1.29 (m, 7H), 0.91 (t, *J* = 7.1 Hz, 3H).

^{13}C NMR (101 MHz, $CDCl_3$): δ 143.9, 128.3 (2C), 127.2, 126.0 (2C), 79.7, 78.5, 36.5, 33.8, 31.4, 27.9, 24.3, 23.0, 14.3.

IR (neat) ν_{max}: 2931, 2857, 1452, 1087, 1046, 746, 696.

HRMS - GC (EI$^+$): exact mass calculated for $[M]^+$ $(C_{15}H_{22}O)^+$ requires *m/z* 218.1665, found *m/z* 218.1662.

***cis*-2-Butyl-6-phenyltetrahydro-2*H*-pyran (9)**

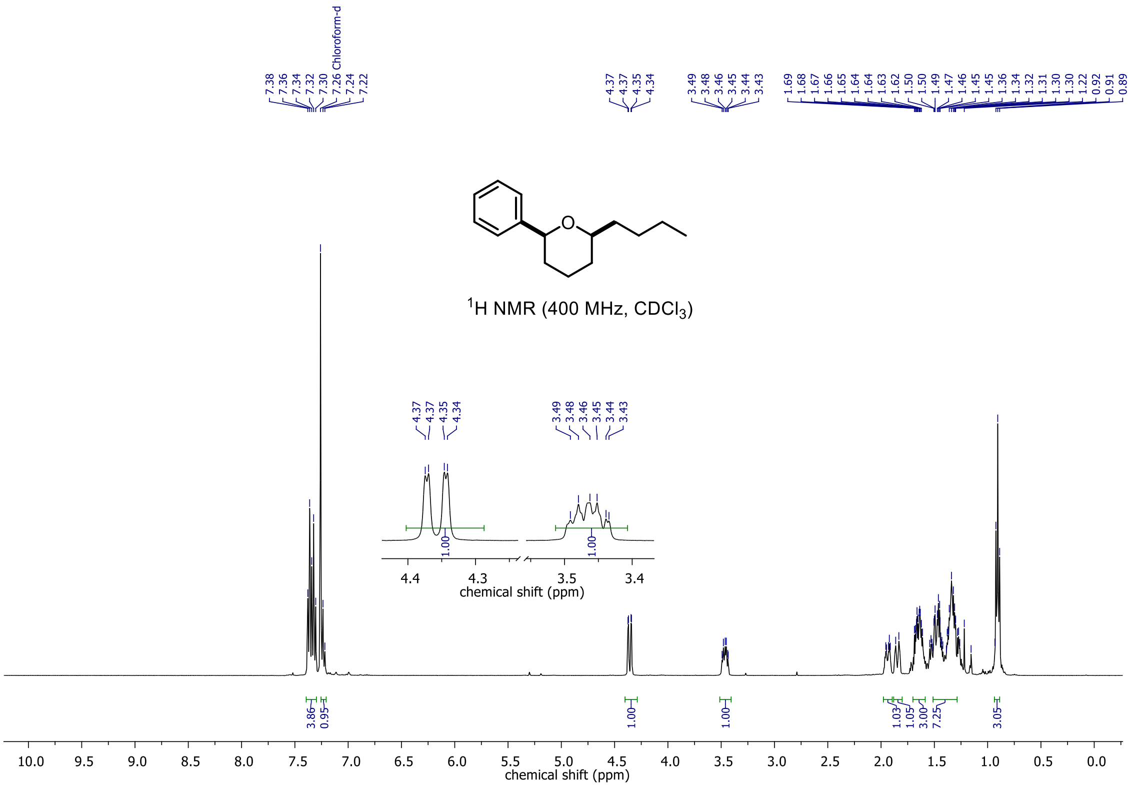

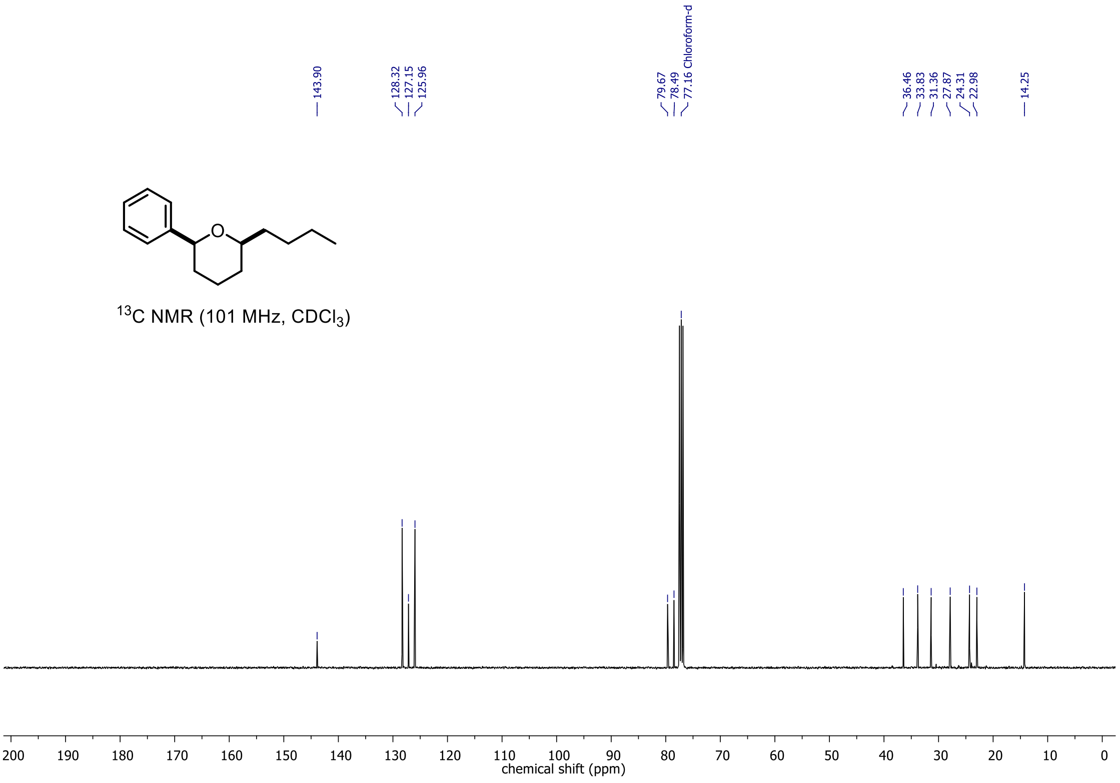

***cis*-2-Pentyl-5-phenyltetrahydrofuran (10)**

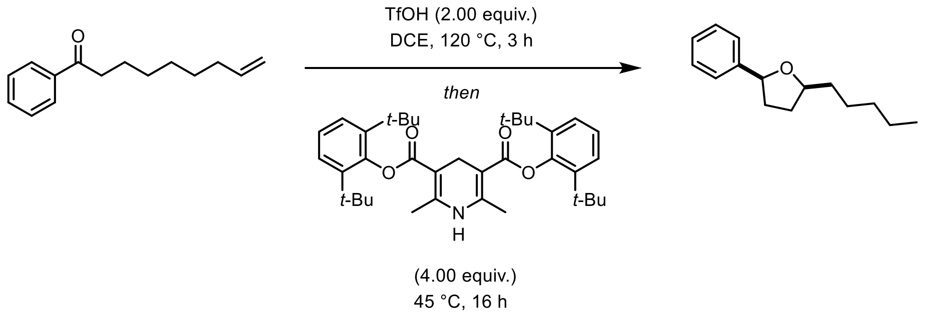

A 4 mL vial was charged with 1-phenylnon-8-en-1-one (32.4 mg, 0.15 mmol, 1.00 equiv.) and DCM (1.5 mL, 0.1 M). TfOH (27 µL, 0.30 mmol, 2.00 equiv.) was then added at 23 °C using a microsyringe. The vial was sealed with a screwcap and placed into a metal heating block (preheated to 120 °C, vial fully immersed, see Section 2.1.4), where the solution was stirred at 120 °C for 3 h. Subsequently, the mixture was cooled in a cold-water bath (10 – 15 °C) for 5 min, after which the Hantzsch ester reducing agent (344 mg, 0.60 mmol, 4.00 equiv.) was carefully added. The mixture was heated to 45 °C for 16 h. Upon completion, an aqueous saturated $NaHCO_3$ solution (2 mL) was added. The mixture was transferred into a separatory funnel, the phases were separated, and the aqueous phase was extracted with DCM (2 × 5 mL). The combined organic layers were dried over anhydrous magnesium sulfate, the dried solution was filtered, and the filtrate was concentrated under reduced pressure. The crude residue was purified using flash column chromatography (0 – 10% Et_2O in heptanes), affording the title compound as a colorless liquid (16.5 mg, 75 µmol, 50%) and as a single regio-/diastereomer.

Crude product: 62% NMR yield, r.r. (γ:δ) >95:5, d.r. = 90:10.

^{1}H NMR (400 MHz, CDCl$_3$): δ 7.39 – 7.28 (m, 4H), 7.26 – 7.21 (m, 1H), 4.87 (t, *J* = 7.3 Hz, 1H), 4.07 – 3.94 (m, 1H), 2.35 – 2.22 (m, 1H), 2.10 – 2.00 (m, 1H), 1.86 – 1.70 (m, 2H), 1.64 – 1.33 (m, 8H), 0.95 – 0.87 (m, 3H).

^{13}C NMR (101 MHz, CDCl$_3$): δ 143.8, 128.4 (2C), 127.2, 126.0 (2C), 80.9, 80.3, 36.2, 34.7, 32.2, 31.5, 26.1, 22.8, 14.2.

IR (neat) ν_{max}: 2927, 2857, 1452, 1090, 1055, 1027, 750, 697.

HRMS - GC (EI$^+$): exact mass calculated for $[M]^+$ $(C_{15}H_{22}O)^+$ requires *m/z* 218.1665, found *m/z* 218.1662.

cis-2-Pentyl-5-phenyltetrahydrofuran (10)

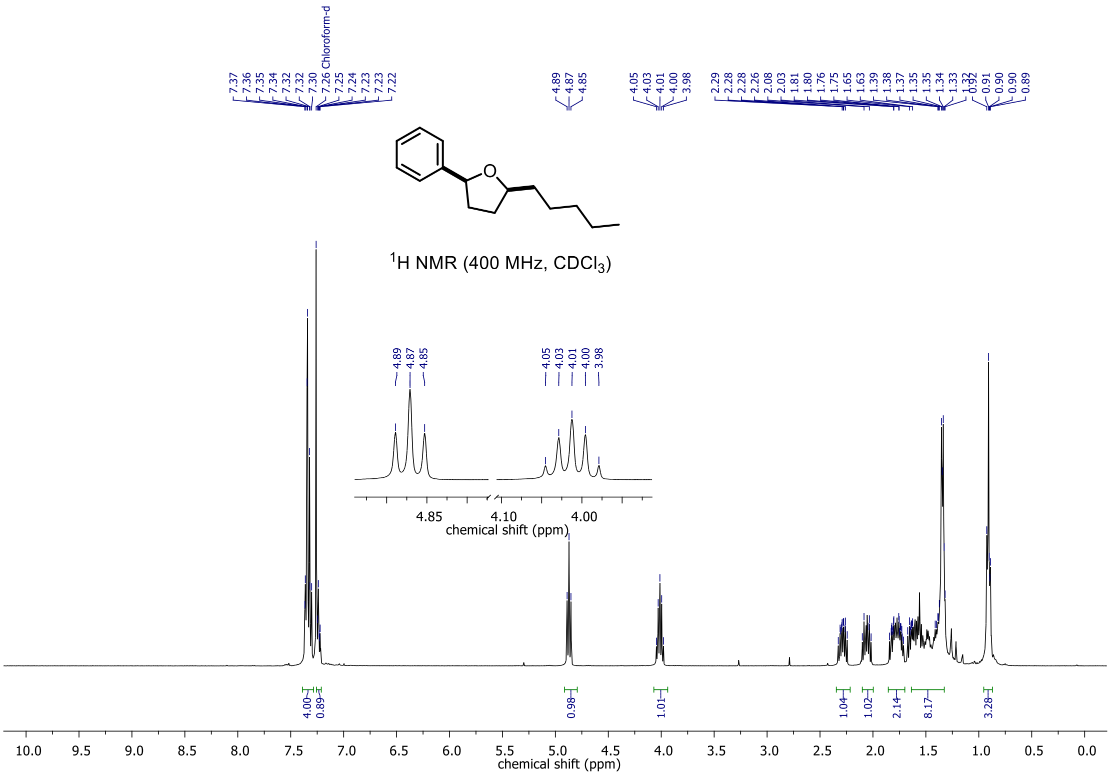

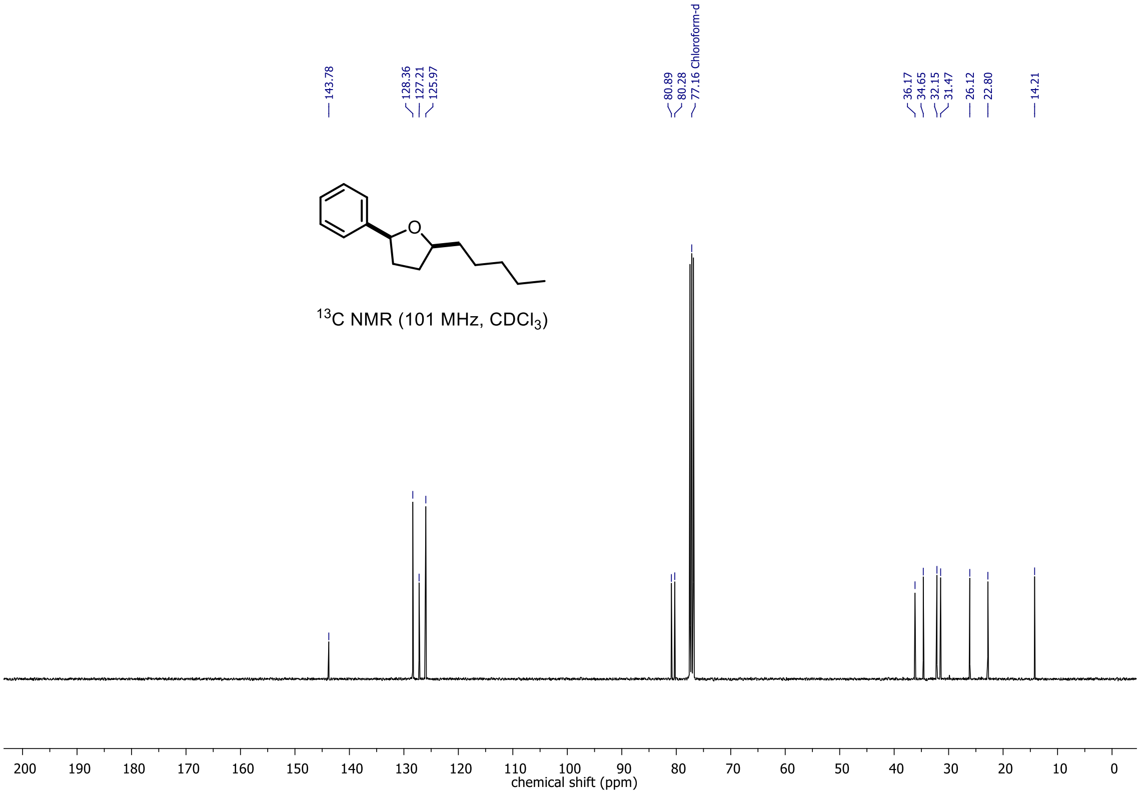

3.3 Reductive removal of ketone

1-Phenylnonan-5-ol (11)

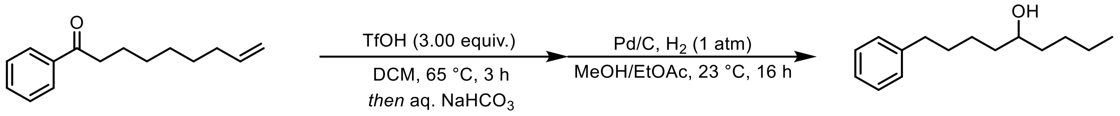

A 4 mL vial was charged with 1-phenylnon-8-en-1-one (43.3 mg, 0.20 mmol, 1.00 equiv.) and DCM (2.0 mL, 0.10 M). TfOH (53 µL, 0.60 mmol, 3.0 equiv.) was added at 23 °C using a microsyringe, the vial was sealed with a screwcap and placed into a metal heating block (pre-heated to 65 °C, vial fully immersed, see Section 2.1.4) and the solution was stirred at 65 °C for 1 h. The vial was then placed into a cold-water bath (10 – 15 °C) for 5 min, after which the vial was opened and an aqueous saturated $NaHCO_3$ solution (2 mL) was added. The mixture was transferred into a separatory funnel. The phases were separated, and the aqueous phase was extracted with DCM (2 × 2 mL). The combined organic layers were dried over anhydrous sodium sulfate, the dried solution was filtered and the filtrate was concentrated under reduced pressure (40 °C water bath). The crude residue was transferred into a Schlenk flask and dissolved in a mixture of dry MeOH/EtOAc (1:1, 1 mL). Palladium on carbon (10%, 106 mg, 0.10 mmol, 0.50 equiv.) was added, and the suspension was sparged with hydrogen gas for 30 min. After this time, the mixture was stirred for 16 h under hydrogen atmosphere (balloon pressure). Upon completion, the crude material was filtered through a Celite pad and rinsed with ethyl acetate (2 mL). Purification by flash column chromatography (0 – 30% EtOAc in heptanes) afforded the title compound as a colorless oil (26.2 mg, 0.12 mmol, 60%) and a single regioisomer.

All analytical data were in good agreement with those reported in literature.[15]

^{1}H NMR (700 MHz, $CDCl_3$): δ 7.30 – 7.27 (m, 2H), 7.21 – 7.17 (m, 3H), 3.62 – 3.56 (m, 1H), 2.64 (t, *J* = 7.8 Hz, 2H), 1.71 – 1.61 (m, 2H), 1.52 – 1.31 (m, 11H), 0.95 – 0.90 (m, 3H).

1-Phenylnonan-5-ol (11)

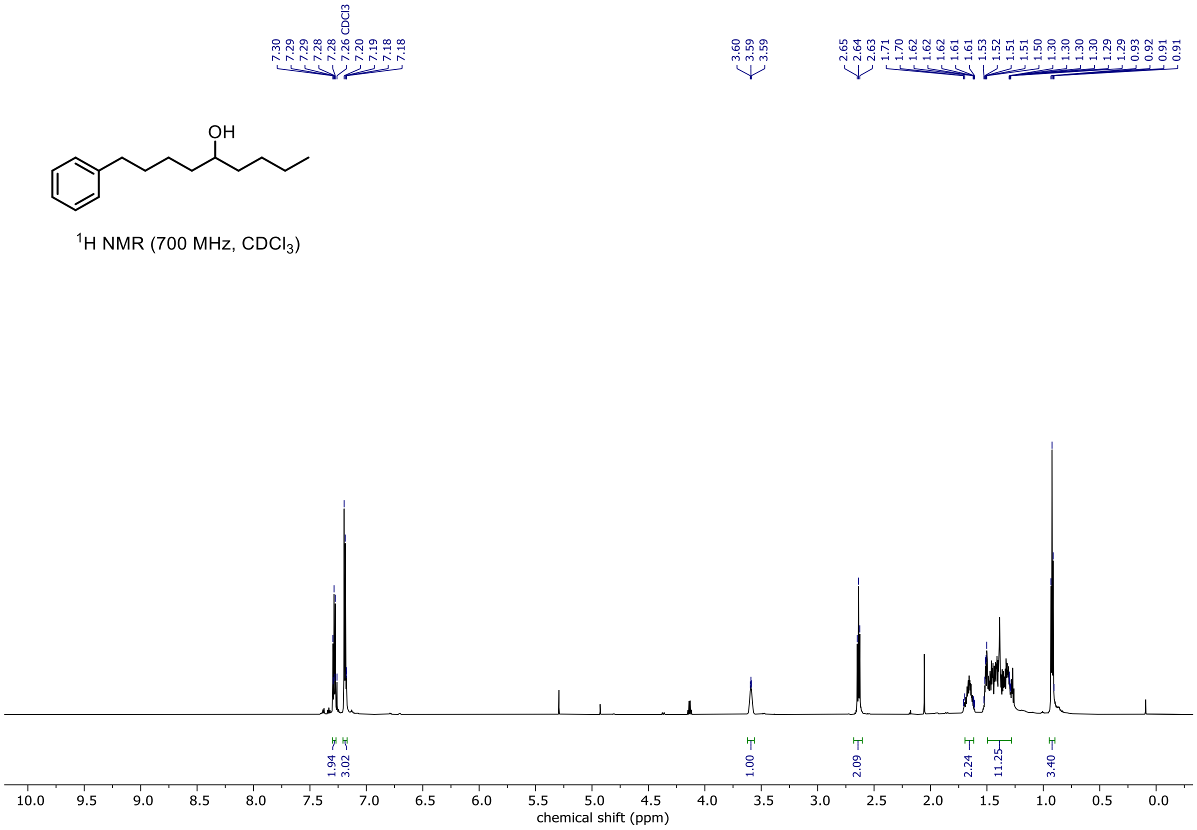

3.4 Allyl silane addition

2-allyl-6-butyl-2-phenyltetrahydro-2H-pyran (12)

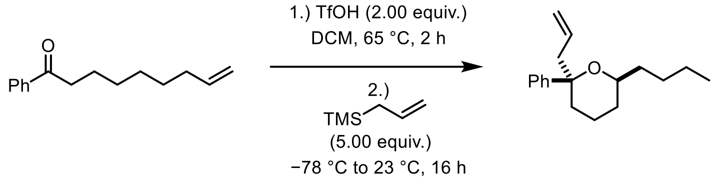

A 4 mL vial was charged with 1-phenylnon-8-en-1-one (21.6 mg, 0.10 mmol, 1.00 equiv.) and DCM (1.0 mL, 0.10 M). TfOH (17.7 µL, 0.20 mmol, 2.00 equiv.) was added at 23 °C using a microsyringe. The vial was sealed with a screwcap and placed into a metal heating block (pre-heated to 65 °C, vial fully immersed, see Section 2.1.4) and the solution was stirred at 65 °C for 2 h. Subsequently, the mixture was cooled to −78 °C, after which allyltrimethylsilane (79.5 µL, 0.50 mmol, 5.00 equiv.) was added dropwise, and the mixture was stirred for 16 h with slow warming to 23 °C. Upon completion, aqueous saturated $NaHCO_3$ solution (5 mL) was added. The mixture was transferred into a separatory funnel, and the aqueous phase was extracted with DCM (3 × 5 mL). The combined organic layers were dried over anhydrous magnesium sulfate, the dried solution was filtered, and the filtrate was concentrated under reduced pressure. The crude residue was purified using flash column chromatography (0 – 3% Et_2O in heptanes), affording the title compound as a yellow oil (13.2 mg, 51 µmol, 51%) as single diastereomer and a mixture of regioisomers ((δ:γ) = 87:13).

Crude product: 51% NMR yield, r.r. (δ:γ) = 86:14, d.r. >95:5.

An asterisk (*) denotes signals that unambiguously arise from the minor diastereomer. The spectrum contains small amount of grease as impurity.

^{1}H NMR (400 MHz, $CDCl_3$) δ 7.49 – 7.39 (m, 2H), 7.34 – 7.28 (m, 2H), 7.24 – 7.17 (m, 1H), 5.81 – 5.63 (m, 0.2H*) 5.52 – 5.35 (m, 0.8H), 5.02 – 4.95 (m, 0.4H*), 4.95 – 4.82 (m, 1.6H), 4.11 – 4.05 (m, 0.1H*), 3.95 – 3.90 (m, 0.1H*), 3.76 – 3.66 (m, 0.8H), 3.02 (dd, *J* = 14.5, 7.0 Hz, 0.8H), 2.63 – 2.48 (m, 0.4H*), 2.43 (dd, *J* = 14.6, 7.0 Hz, 0.8H), 2.19 – 2.12 (m, 0.4H*), 2.02 – 1.97 (m, 1H), 1.88 – 1.72 (m, 1.6H), 1.65 – 1.47 (m, 4H), 1.46 – 1.41 (m, 1H), 1.37 – 1.31 (m, 3H)1.24 – 1.15 (m, 1H), 0.93 (t, *J* = 7.2 Hz, 3H).

^{13}C NMR (151 MHz, $CDCl_3$): δ 148.1, 134.0, 127.9 (2C), 126.3, 125.0 (2C), 116.9, 76.4, 70.0, 38.9, 36.9, 35.4, 31.5, 28.0, 23.0, 20.2, 14.3.

IR (neat) v_{max}: 3063, 2930, 2858, 1640, 1493, 1446, 1379, 1267, 1201, 1076, 1046, 999, 910, 765, 736, 699.

HRMS (ESI$^+$): exact mass calculated for $[M+Na]^+$ $(C_{18}H_{26}ONa)^+$ requires *m/z* 281.1876, found *m/z* 281.1874.

2-allyl-6-butyl-2-phenyltetrahydro-2H-pyran (12)

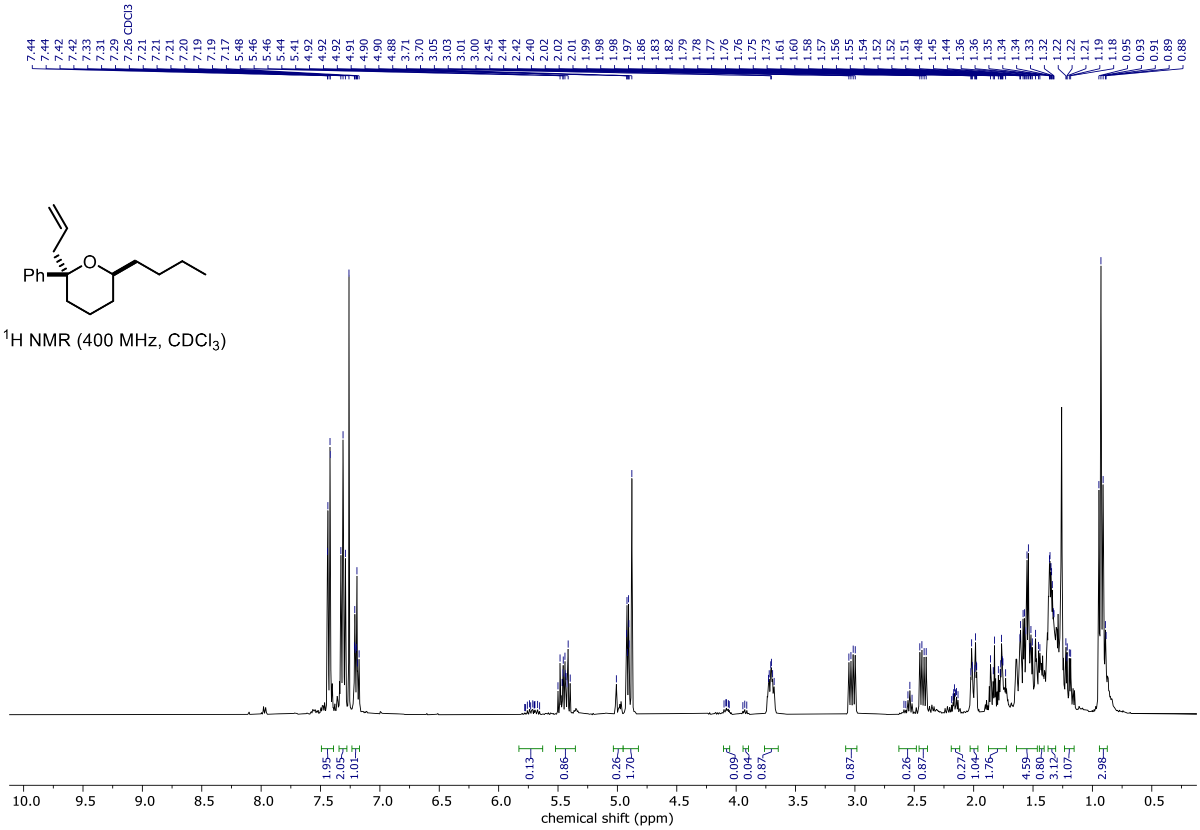

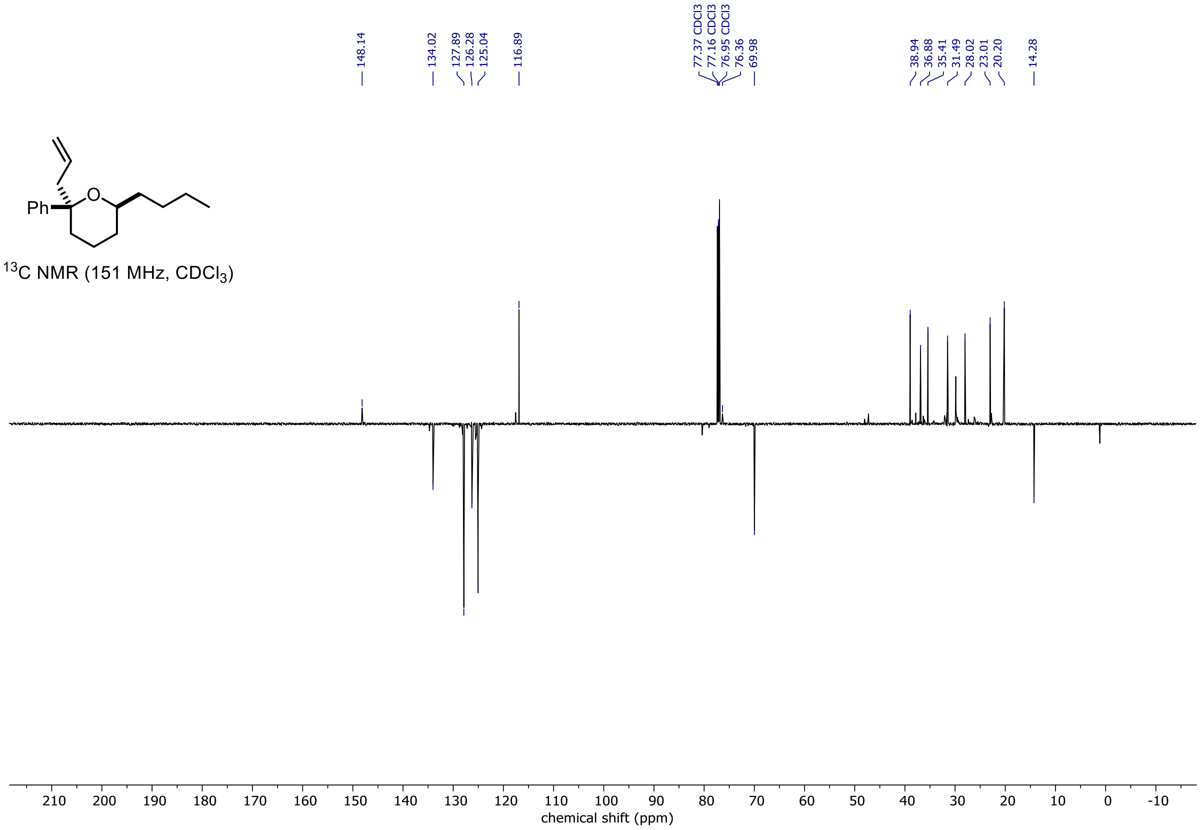

3.5 Grignard reagent addition

2-Methyl-5-pentyl-2-phenyltetrahydrofuran (13)

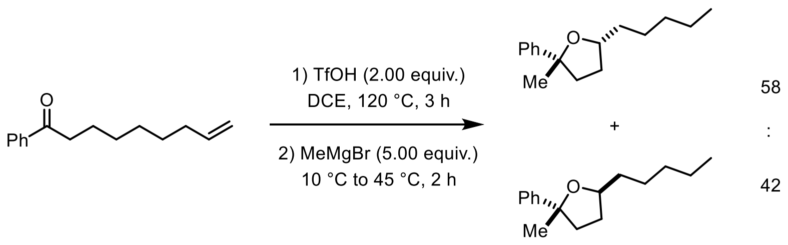

A small vial was charged with 1-phenylnon-8-en-1-one (32.4 mg, 0.15 mmol, 1.00 equiv.) and DCM (1.5 mL, 0.10 M). TfOH (27 µL, 0.30 mmol, 2.00 equiv.) was then added at 23 °C using a microsyringe. The vial was sealed with a screwcap, placed into a heating block (preheated to 120 °C, vial fully immersed, see Section 2.1.4) and the solution was heated to 120 °C for 3 h. Subsequently, the vial was cooled in a cold-water bath (10 – 15 °C) for 5 min, after which MeMgBr (3 M in Et_2O, 0.25 mL, 0.75 mmol, 5.00 equiv.) was added slowly. The resulting mixture was then heated to 45 °C for 2 h. Upon completion, aqueous saturation $NaHCO_3$ solution (5 mL) was added. The mixture was transferred into a separatory funnel, and the aqueous phase was extracted with DCM (2 × 5 mL). The combined organic layers were dried over anhydrous magnesium sulfate, the dried solution was filtered, and the filtrate was concentrated under reduced pressure. The crude residue was purified using flash column chromatography (0 – 10% Et_2O in heptanes), affording the title compound as a colorless oil (17.3 mg, 75 µmol, 50%), a single regioisomer and a mixture of two diastereomers (d.r. = 58:42).

Crude product: 54% NMR yield, r.r. (γ:δ) >95:5, d.r. = 58:42.

An asterisk (*) denotes signals that unambiguously arise from the minor diastereomer.

^{1}H NMR (400 MHz, $CDCl_3$): δ 7.49 – 7.37 (m, 2H), 7.35 – 7.28 (m, 2H), 7.21 (t, *J* = 7.3 Hz, 1H), 4.18 – 4.08 (m, 0.6H), 3.99 – 3.91 (m, 0.4H*), 2.25 – 2.16 (m, 1H), 2.11 – 1.99 (m, 1.5H), 1.93 – 1.83 (m, 0.5H), 1.78 – 1.59 (m, 2H), 1.52 (s, 3H), 1.48 – 1.31 (m, 7H), 0.90 (td, *J* = 6.8, 2.8 Hz, 3H).

^{13}C NMR (101 MHz, $CDCl_3$): δ 149.5, 149.0, 128.2* (2C), 128.0 (2C), 126.32, 126.29, 125.0 (2C), 124.8* (2C), 84.2, 84.1, 79.8, 79.2, 40.3, 39.5, 36.5, 36.4, 32.2, 32.1, 31.8, 31.6, 31.2, 30.1, 26.2, 26.0, 22.81, 22.78, 14.23, 14.20.

IR (neat) v_{max}: 2926, 2857, 1445, 1102, 1067, 1028, 761, 699.

HRMS (ESI$^+$): exact mass calculated for $[M+Na]^+$ $(C_{16}H_{24}ONa)^+$ requires *m/z* 255.1719, found *m/z* 255.1725.

2-Methyl-5-pentyl-2-phenyltetrahydrofuran (13)

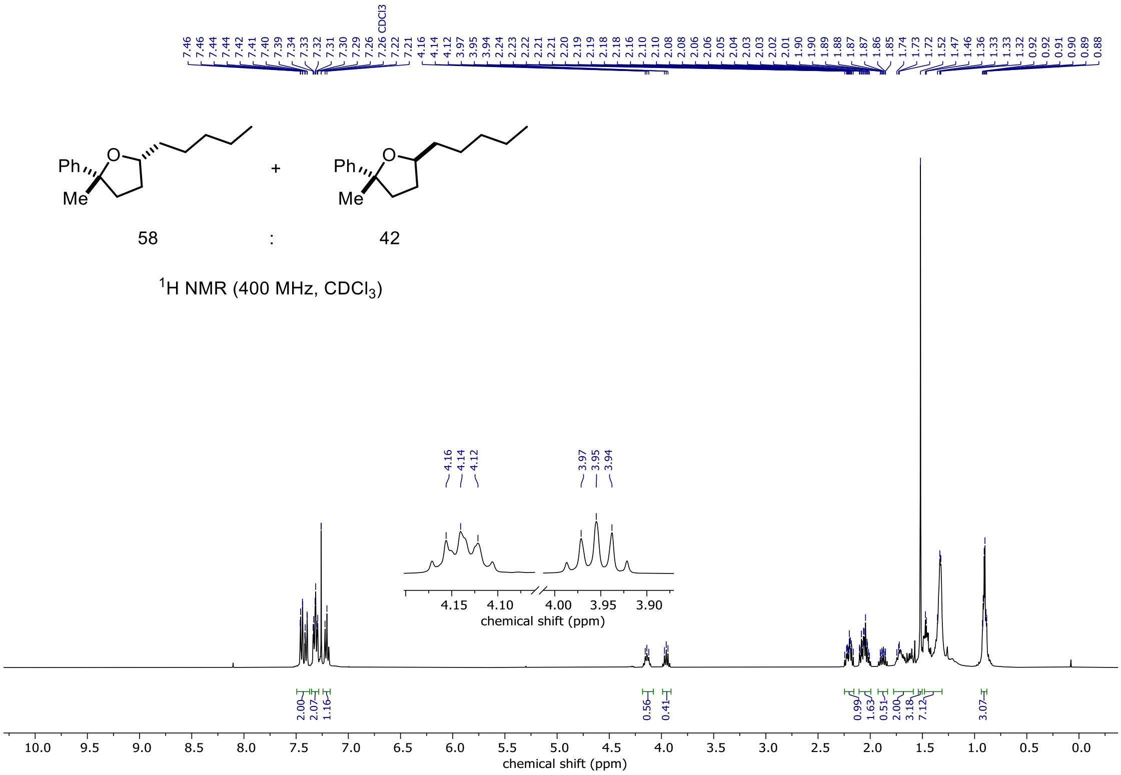

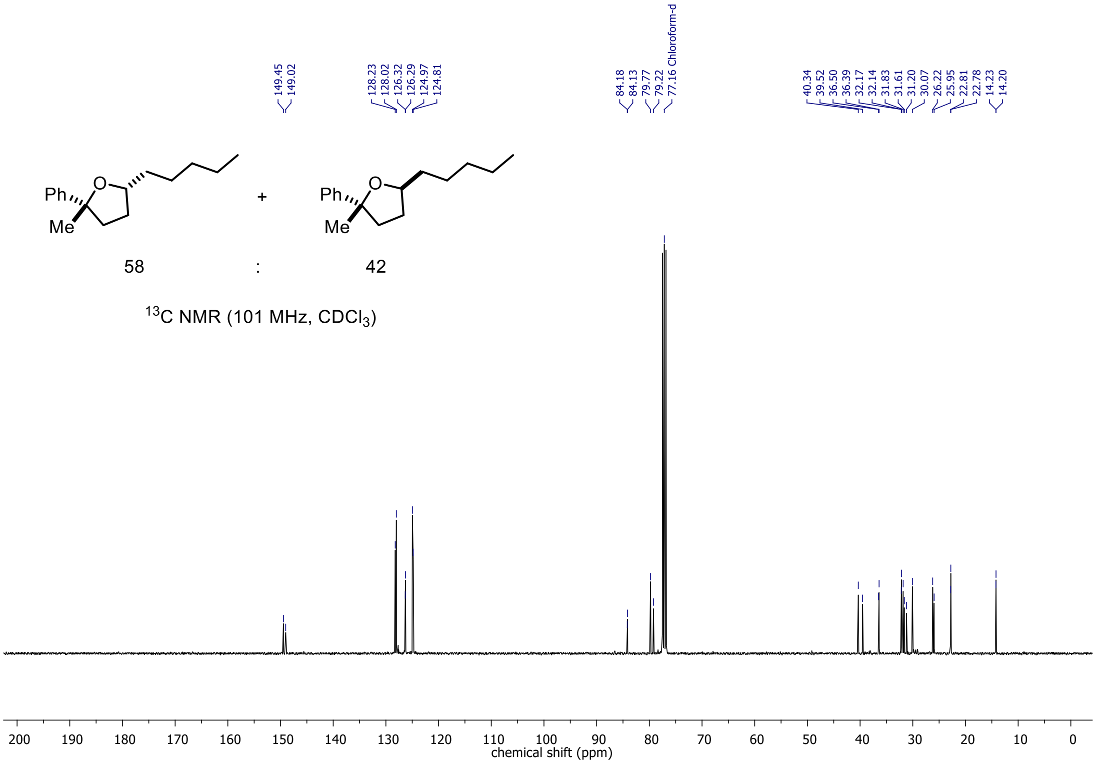

6-Butyl-2-methyl-2-phenyltetrahydro-2H-pyran (14)

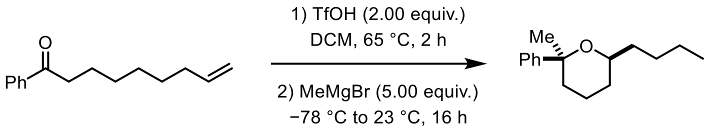

A small vial was charged with 1-phenylnon-8-en-1-one (21.6 mg, 0.10 mmol, 1.00 equiv.) and DCM (1 mL, 0.10 M). TfOH (27.7 µL, 0.20 mmol, 2.00 equiv.) was then added at 23 °C using a microsyringe. The vial was sealed with a screwcap, placed into a heating block (preheated to 65 °C, vial fully immersed, see Section 2.1.4) and the solution was heated to 65 °C for 2 h. Subsequently, the mixture was cooled to −78 °C, after which a solution of MeMgBr (3 M in Et_2O, 0.167 mL, 0.50 mmol, 5.00 equiv.) was added dropwise, and the mixture was stirred for 16 h with slow warming to 23 °C. Upon completion, aqueous saturated $NaHCO_3$ solution (5 mL) was added. The mixture was transferred into a separatory funnel, and the aqueous phase was extracted with DCM (2 × 5 mL). The combined organic layers were dried over anhydrous magnesium sulfate, the dried solution was filtered, and the filtrate was concentrated under reduced pressure. The crude residue was purified using flash column chromatography (0 – 3% Et_2O in heptanes), affording the title compound as a colorless oil (15.4 mg, 66 µmol, 66%) as single diastereomer and a mixture of regioisomers ((δ:γ) = 91:9).

Crude product: 82% NMR yield, r.r. (δ:γ) = 89:11, d.r. >95:5.

An asterisk (*) denotes signals that unambiguously arise from the minor regioisomer (2 diastereomers).

^{1}H NMR (600 MHz, $CDCl_3$): δ δ 7.51 – 7.47 (m, 2H), 7.35 – 7.30 (m, 2H), 7.21 (t, *J* = 7.3 Hz, 1H), 4.15 – 4.13 (m, 0.03H*), 3.98 – 3.93 (m, 0.05H*), 3.77 – 3.70 (m, 1H), 1.95 – 1.90 (m, 1H), 1.85 – 1.74 (m, 2H), 1.67 – 1.54 (m, 3H), 1.54 – 1.42 (m, 5H), 1.42 – 1.31 (m, 3H), 1.23 – 1.16 (m, 1H), 0.94 (t, *J* = 7.2 Hz, 3H).

Due to low abundance, only signals arising from the major diastereomer are reported in ^{13}C NMR.

^{13}C NMR (151 MHz, $CDCl_3$): δ 150.7, 128.1 (2C), 126.3, 124.3 (2C), 74.6, 70.3, 36.8, 36.4, 31.8, 28.0, 23.3, 23.0, 20.4, 14.3.

IR (neat) v_{max}: 2930, 2859, 1446, 1370, 1259, 1203, 1070, 1030, 1006, 760, 697, 549.

HRMS (ESI$^+$): exact mass calculated for $[M+Na]^+$ $(C_{16}H_{24}ONa)^+$ requires *m/z* 255.1719, found *m/z* 255.1722.

6-Butyl-2-methyl-2-phenyltetrahydro-2H-pyran (14)

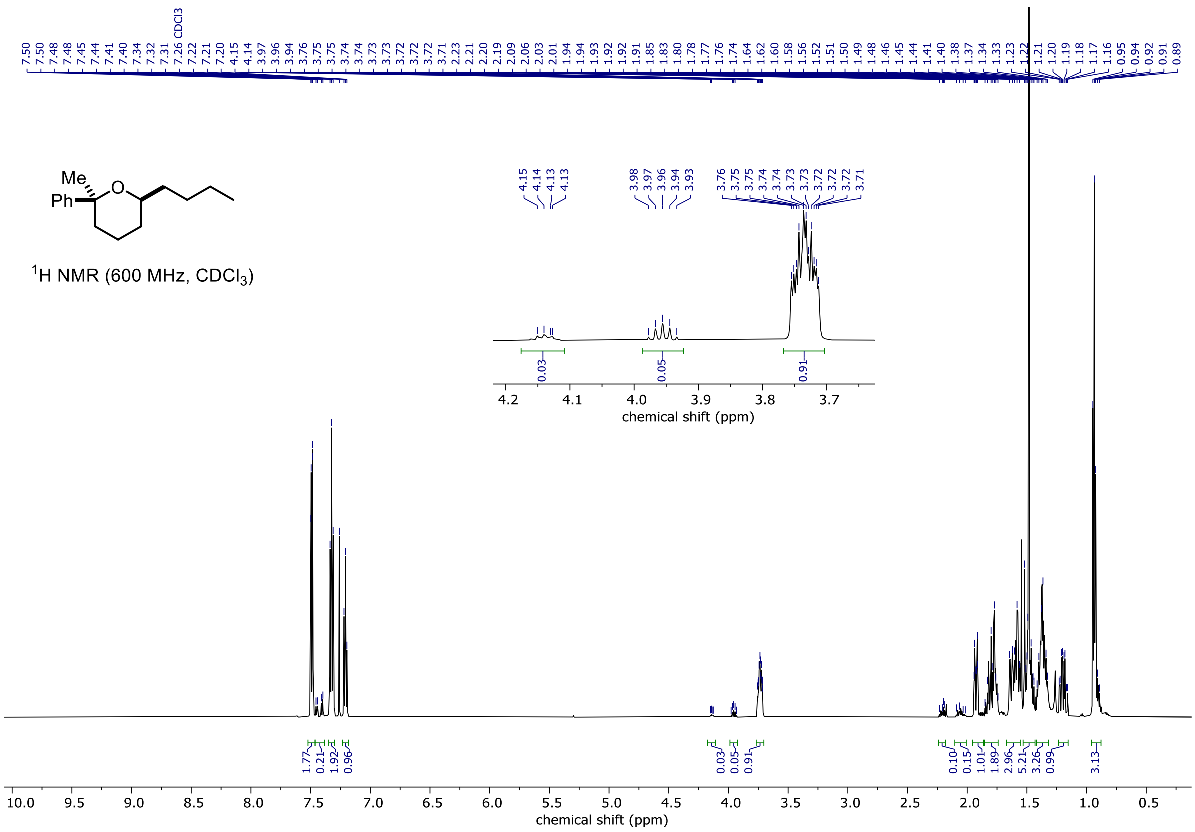

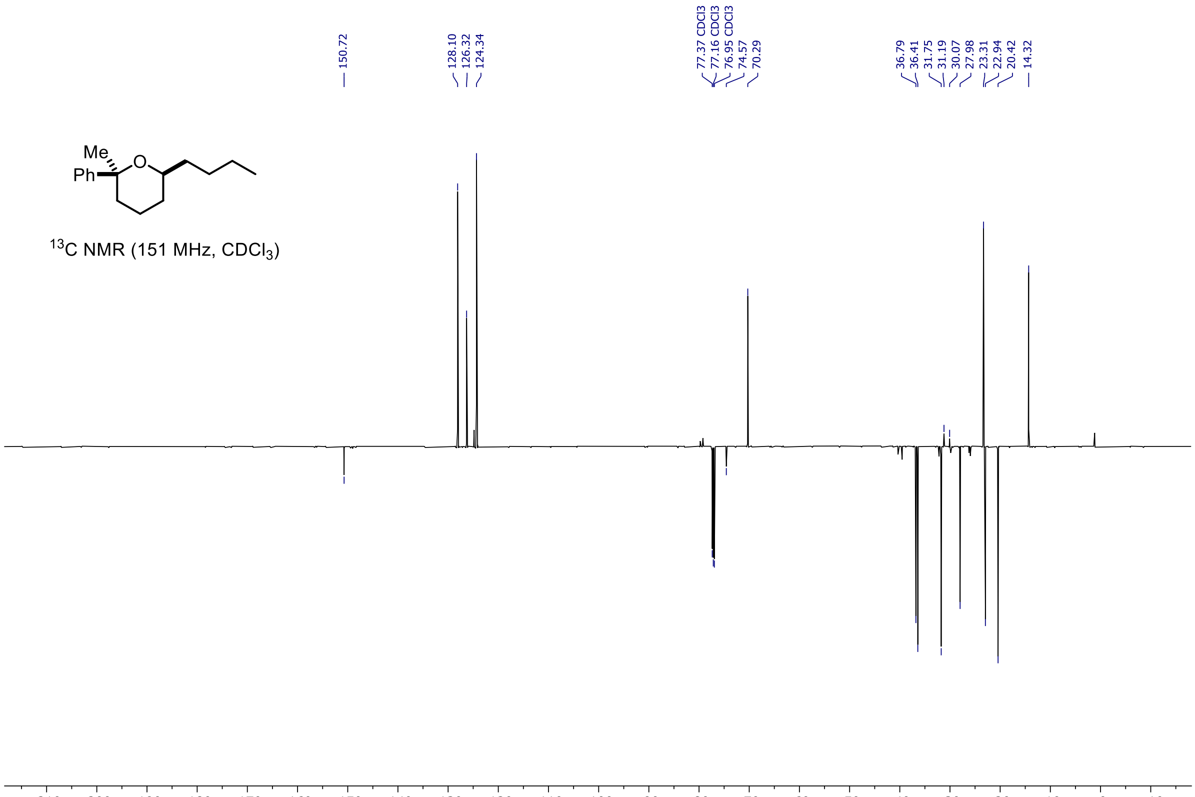

4. Mechanistic Studies

4.1 NMR study of cation sampling

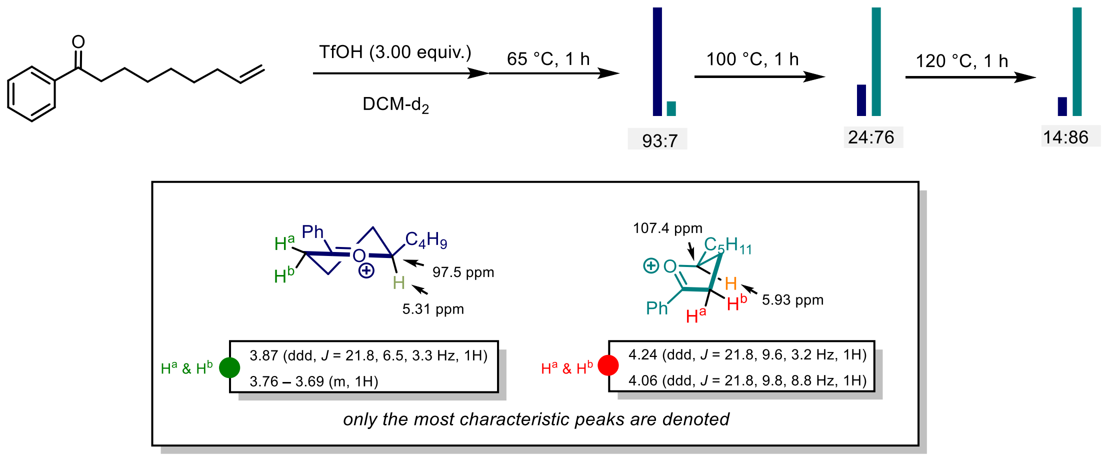

Figure S6. NMR study of the cation sampling process.

In our NMR study (Figure S6), we observed a smooth transition from a six-membered oxocarbenium ion to a five-membered oxocarbenium ion upon temperature increase (65 °C → 100 °C → 120 °C). These results indicate that the six-membered oxocarbenium ion is initially formed as the kinetic product, while the five-membered oxocarbenium ion represents the thermodynamic product.

On the following pages, we provide more details of this study along with a summary of the results.

Procedure of this NMR study:

1-Phenylnon-8-en-1-one (43.3 mg, 0.20 mmol, 1.00 equiv.) was weighed into a vial (4 mL total volume, no inert atmosphere required) and dissolved in DCM-d_2 (2 mL) at 23 °C. TfOH (53.1 µL, 0.60 mmol, 3.00 equiv.) was added, the vial was sealed with a screwcap, then placed into a metal heating block (pre-heated to 65 °C, vial fully immersed, see Section 2.1.4) and the solution was stirred for 1 h at 65 °C. The vial was then moved to a cold-water bath (10 °C) for 5 min, after which a small aliquot (0.2 mL) was withdrawn and transferred to an NMR tube, which was filled with additional DCM-d_2 (approx. 0.4 mL, see attached spectra for results). The reaction mixture was then heated at 100 °C for another 1 h (same procedure), and another aliquot was withdrawn by the same method. Finally, the reaction was heated at 120 °C for 1 h, after which the last sample was collected.

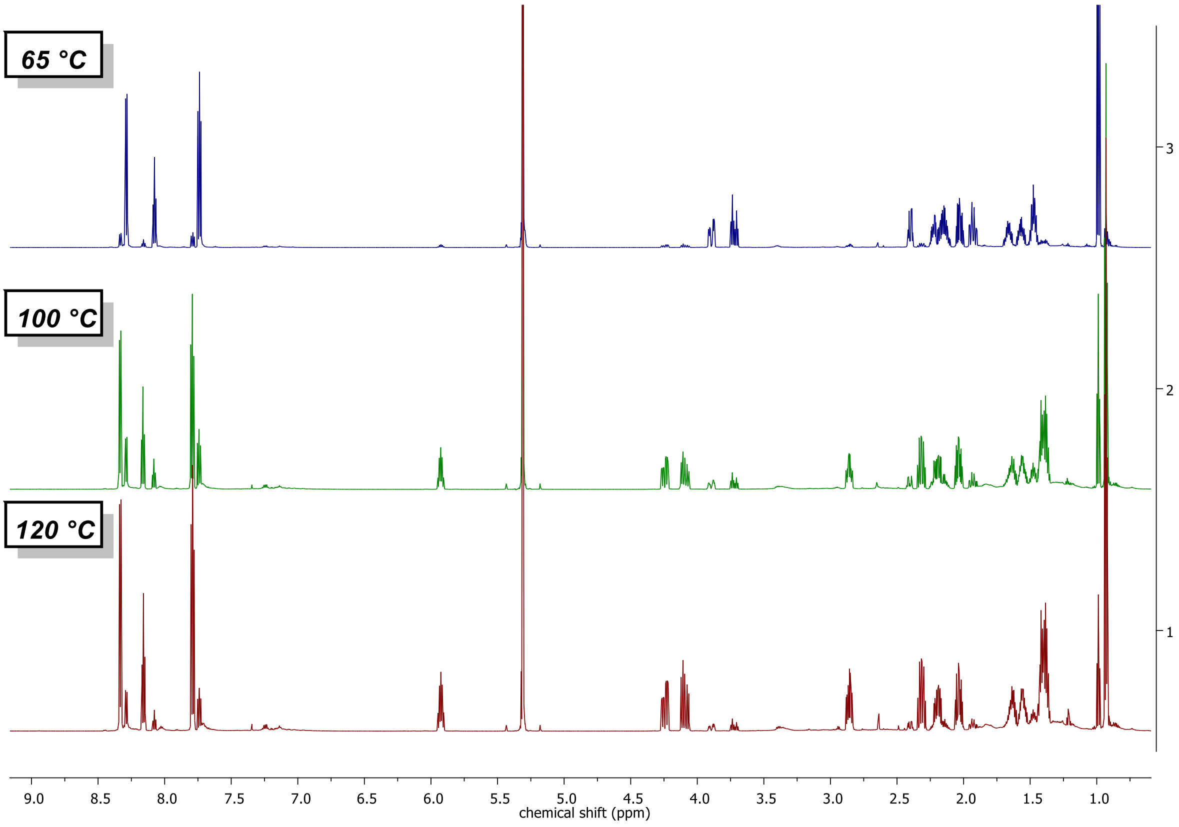
65 °C
100 °C
120 °C
9.0 8.5 8.0 7.5 7.0 6.5 6.0 5.5 5.0 4.5 4.0 3.5 3.0 2.5 2.0 1.5 1.0
chemical shift (ppm)
3
2
1

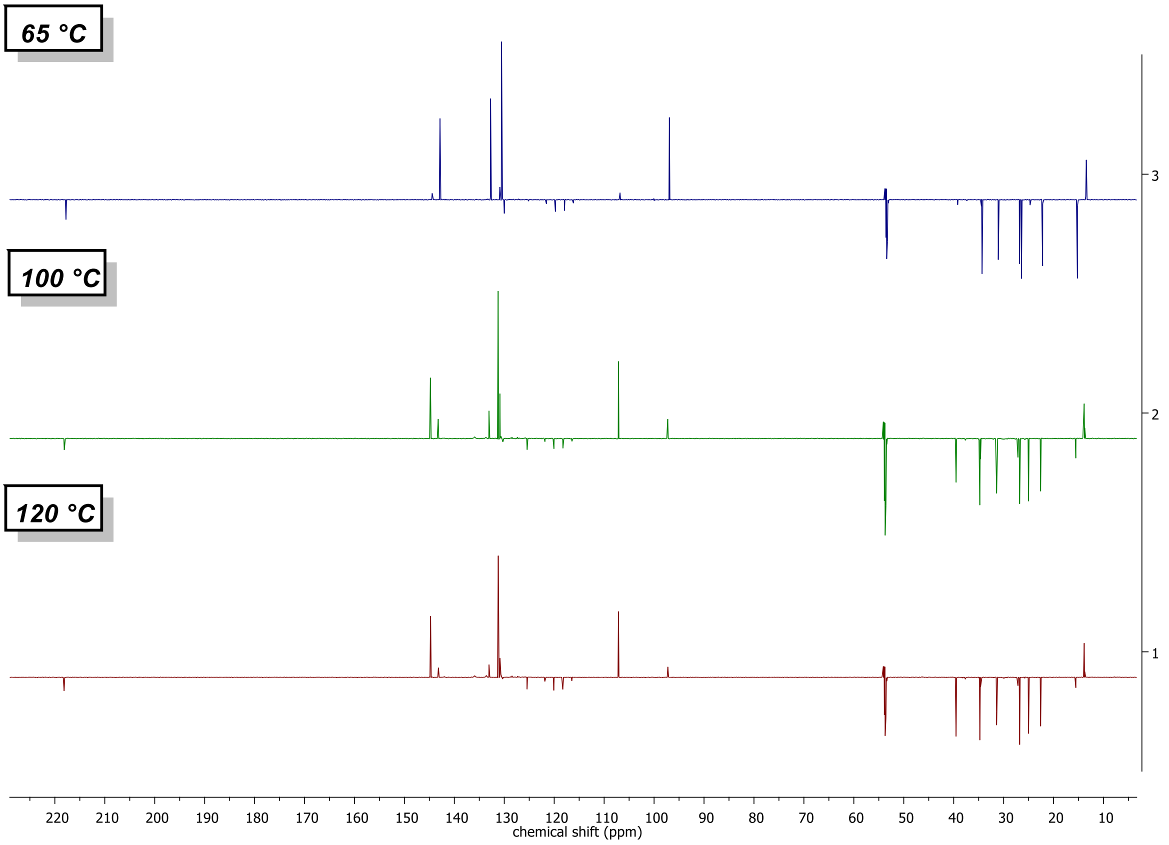
65 °C
100 °C
120 °C
220 210 200 190 180 170 160 150 140 130 120 110 100 90 80 70 60 50 40 30 20 10
chemical shift (ppm)
3
2
1

65 °C

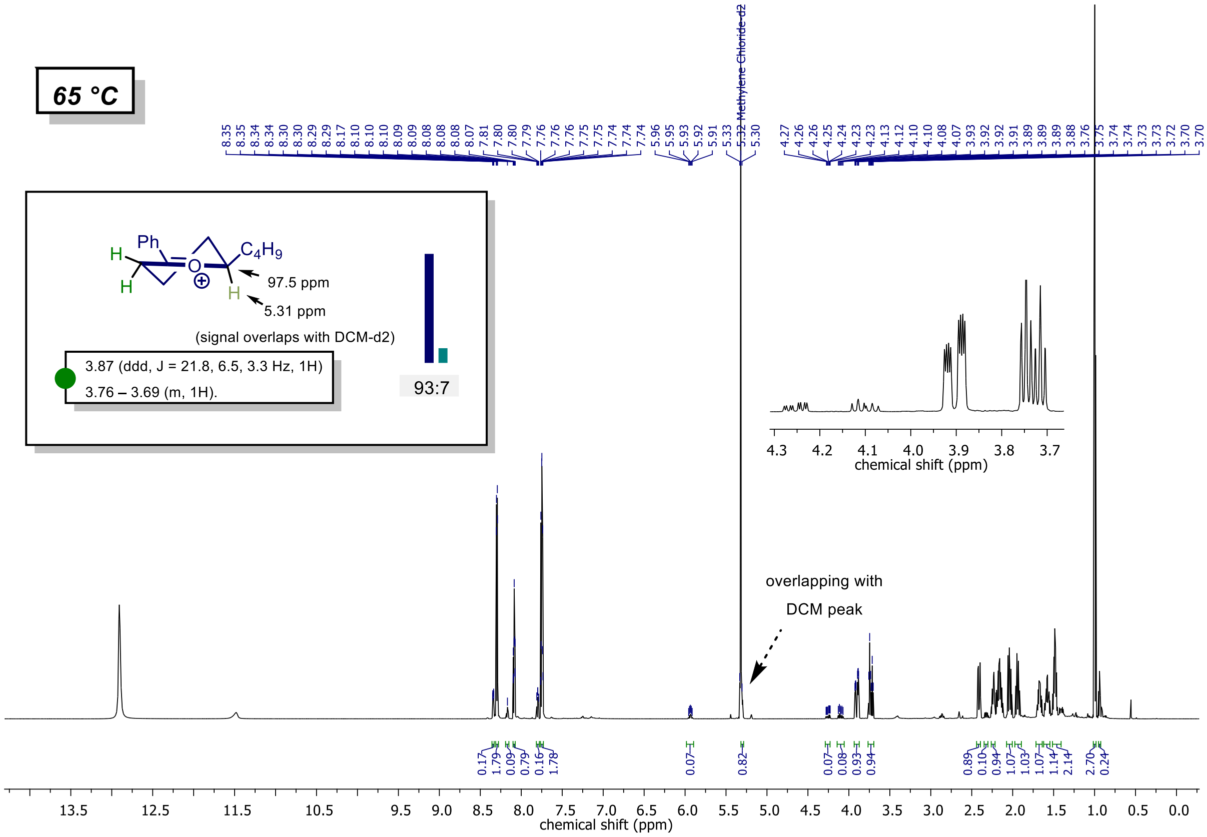

97.5 ppm
5.31 ppm
(signal overlaps with DCM-d2)
3.87 (ddd, J = 21.8, 6.5, 3.3 Hz, 1H)
3.76 – 3.69 (m, 1H).
93:7
overlapping with
DCM peak
chemical shift (ppm)

65 °C

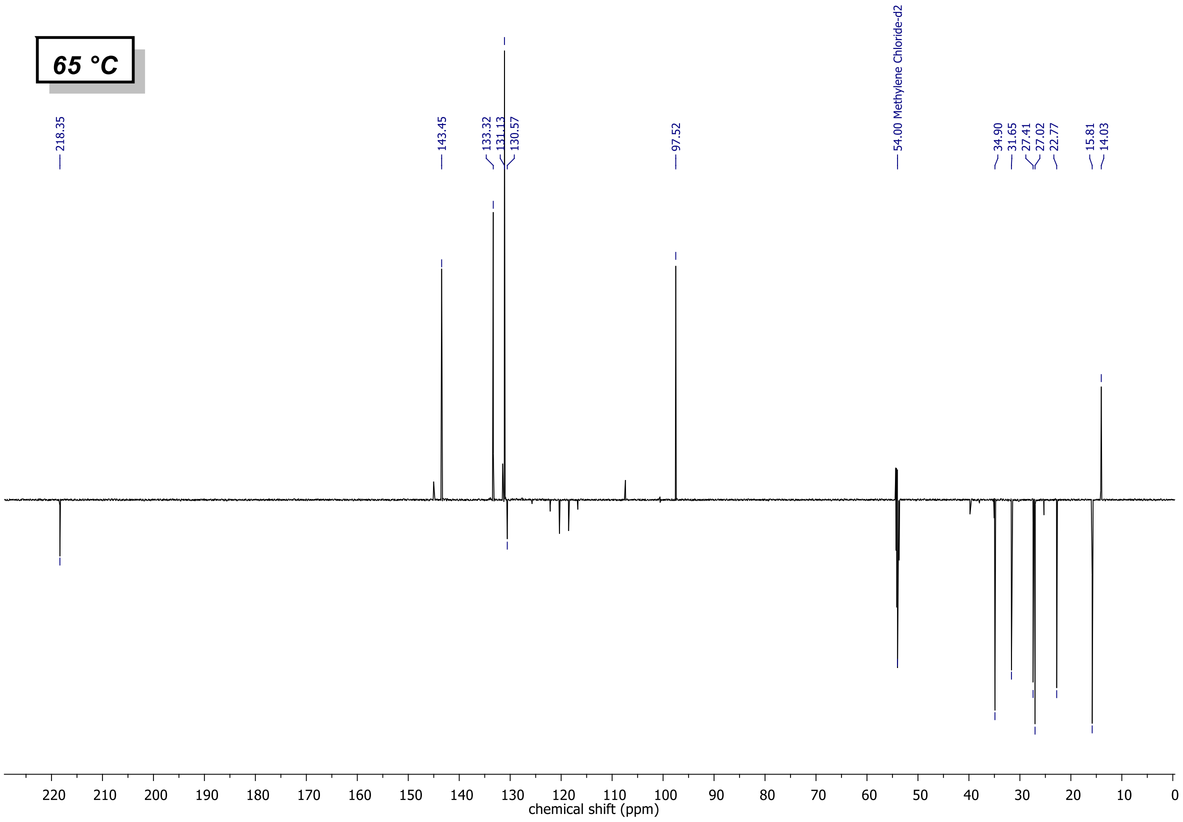

218.35
143.45
133.32
131.13
130.57
97.52
54.00 Methylene Chloride-d2
34.90
31.65
27.41
27.02
22.77
15.81
14.03
chemical shift (ppm)

120 °C

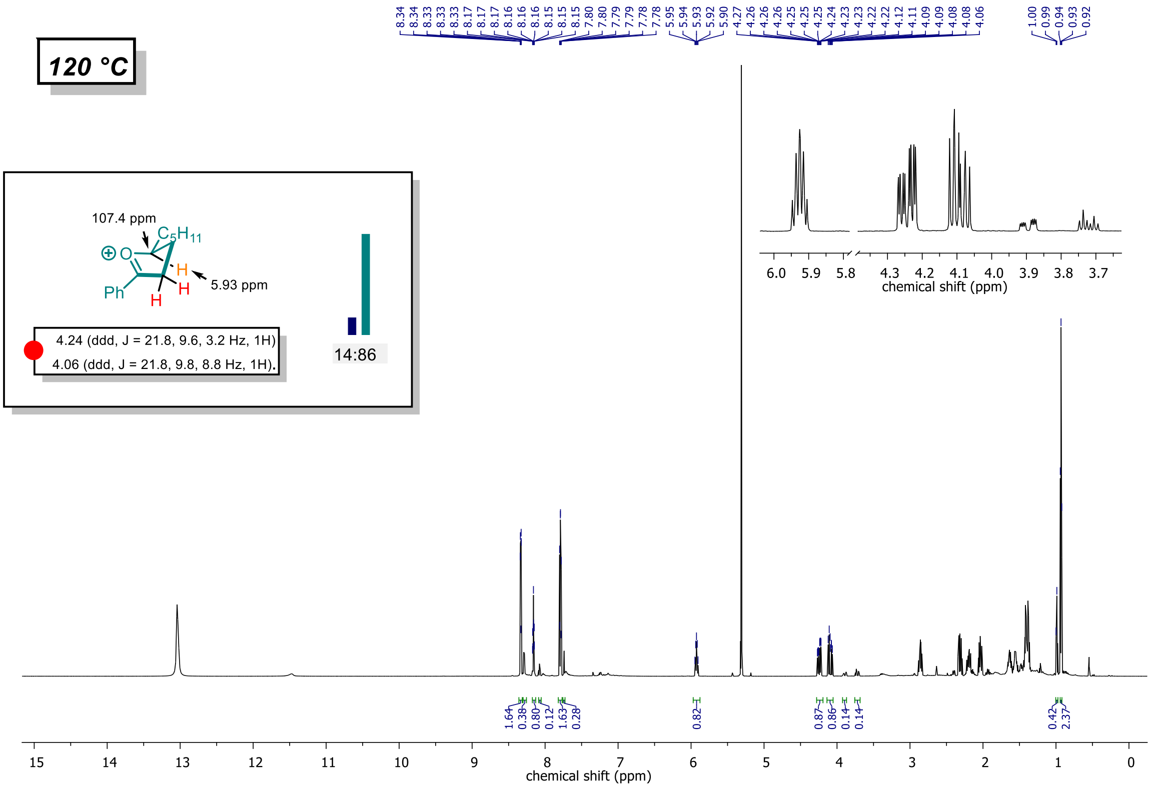
107.4 ppm
C5H11
O
Ph
H
H
H
5.93 ppm
4.24 (ddd, J = 21.8, 9.6, 3.2 Hz, 1H)
4.06 (ddd, J = 21.8, 9.8, 8.8 Hz, 1H).
14:86
chemical shift (ppm)
chemical shift (ppm)

120 °C

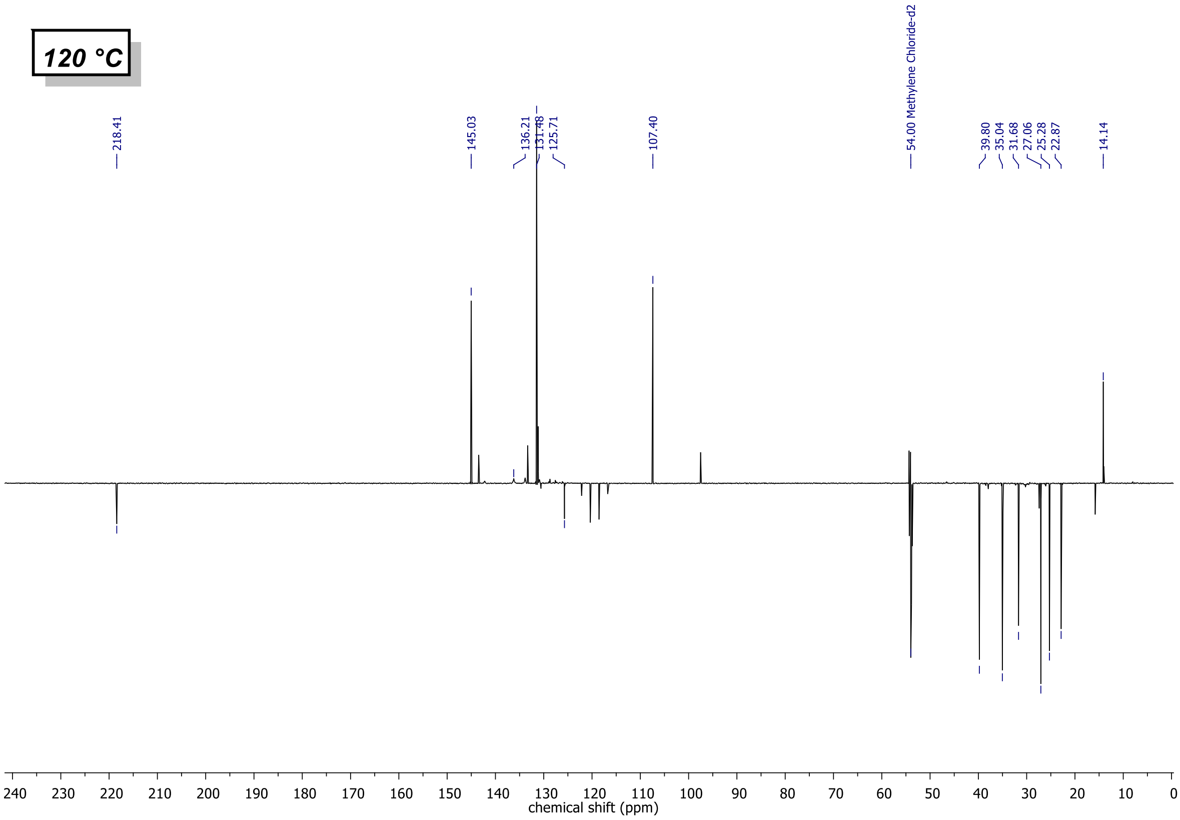
218.41
145.03
136.21
131.48
125.71
107.40
54.00 Methylene Chloride-d2
39.80
35.04
31.68
27.06
25.28
22.87
14.14
chemical shift (ppm)

4.2 Studies on the influence of HFIP

4.2.1 Explanation of the background

As outlined in the optimization table for δ-functionalization (Section 2.1), significant formation of dihydropyran alongside the desired δ-hydroxy ketone was observed when the reaction was carried out in DCM, followed by an aqueous basic quench. However, parallel studies showed that HFIP could help overcome this issue by selectively providing only the desired δ-hydroxy ketone—albeit at the cost of decreased mass balance.

To address both issues—dihydropyran formation in DCM and polymerization/decomposition in HFIP—we identified an intriguing synergy between the two solvents. Specifically, we performed the cation sampling in DCM and only added HFIP just before the aqueous basic quench. Unsure of HFIP's role in this quenching process, particularly whether it might affect the oxocarbenium ion, we were prompted to further investigate its influence in the quenching step.

4.2.2 NMR study in HFIP-d2 – evidence of oxocarbenium species in HFIP

Since, in our later studies, HFIP proved to be an equally feasible reaction solvent (see Section 2.1), we conducted a similar study, as presented in section 4.1, in HFIP-d_2. Based on the gathered data (Figure S7), the oxocarbenium ion remains the main intermediate in the cation sampling process. The most distinct signals, as previously observed in DCM-d_2 (see section 4.1), were the characteristic carbonyl peaks at 216.8 ppm and 216.7 ppm, corresponding to the five- and six-membered oxocarbenium species, respectively. Additionally, the C–H signal of the carbon attached to the oxygen (for 6-membered oxcarbenium) was detected, with a proton resonance at 5.62 ppm and a carbon signal at 96.4 ppm.

We also observed significant deuteration of the alkyl chain and the α-position of the ketone, which partially complicates interpretation.[16] This deuteration accounts for the reduced integrals of the alkyl proton signals relative to those of the phenyl ring in the NMR spectrum. Additionally, the ^{13}C spectra showed unchanged carbon signals for the phenyl ring but revealed a fading of various alkyl signals. Consequently, the ratio of the two oxocarbenium species was determined to be 84:16 (6-membered to 5-membered oxocarbenium) by analyzing the distinct chemical shifts of the phenyl protons associated with each species.

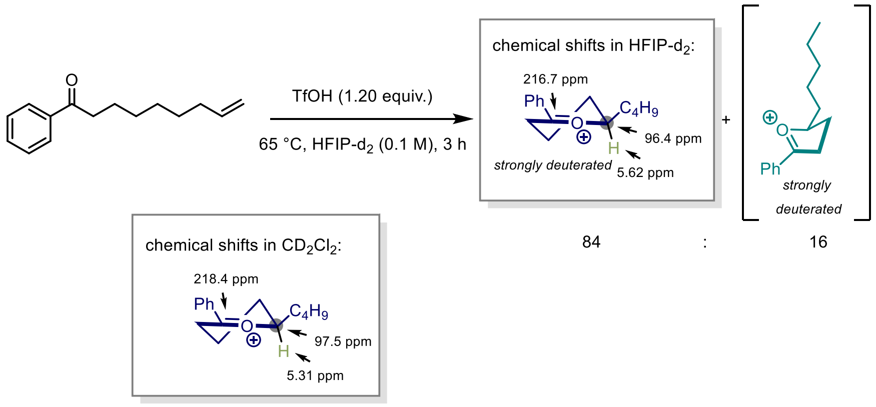

Figure S7. NMR study in HFIP-d2. We observed significant deuteration of the alkyl chain and α-position of the ketone (see below for more details).

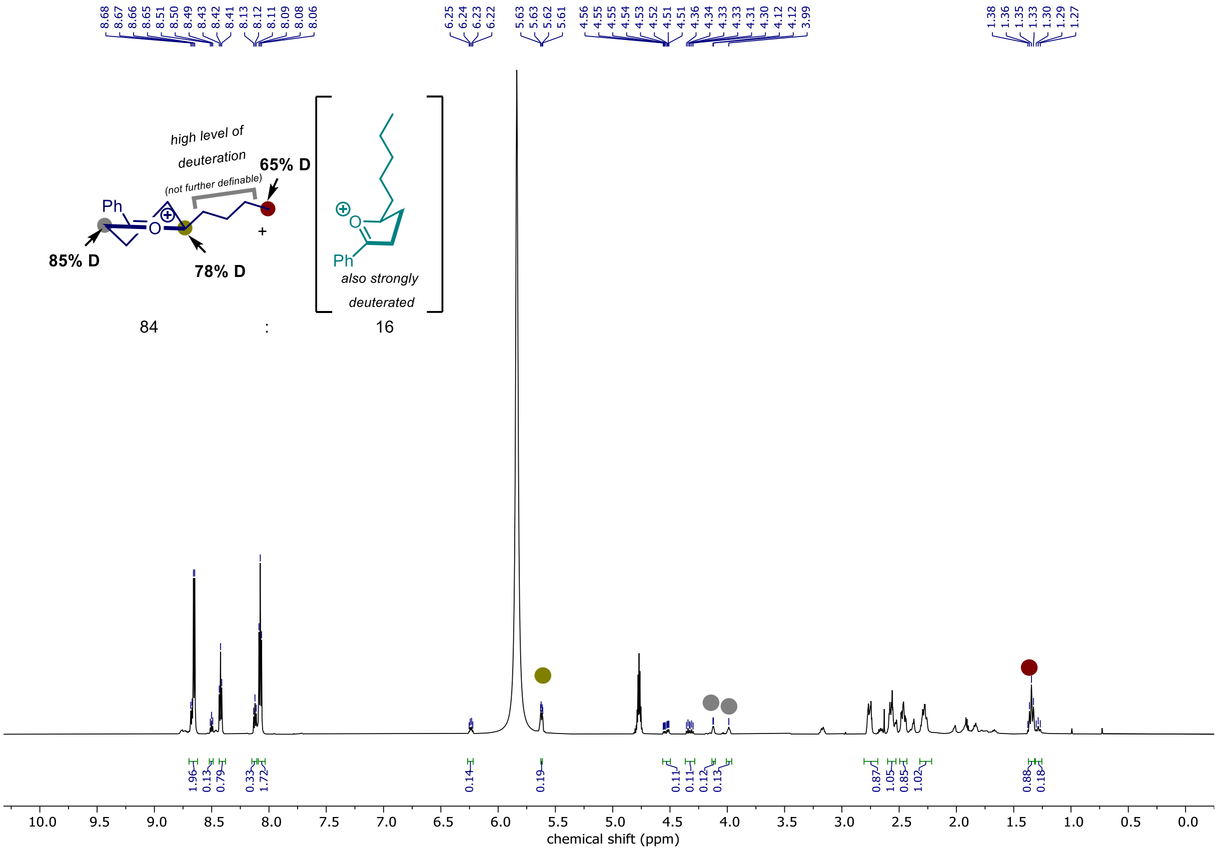
8.68 8.67 8.66 8.65 8.51 8.50 8.49 8.43 8.42 8.41 8.13 8.12 8.11 8.09 8.08 8.06
6.25 6.24 6.23 6.22
5.63 5.63 5.62 5.61
4.56 4.55 4.55 4.54 4.53 4.52 4.51 4.51 4.36 4.34 4.33 4.33 4.31 4.30 4.12 4.12 3.99
1.38 1.36 1.35 1.33 1.30 1.29 1.27
high level of
deuteration
(not further definable)
65% D
Ph
85% D
78% D
+
Ph
also strongly
deuterated
84
:
16
1.96 0.13 0.79 0.33 1.72 0.14 0.19 0.11 0.11 0.12 0.13 0.87 1.05 0.85 1.02 0.88 0.18
10.0 9.5 9.0 8.5 8.0 7.5 7.0 6.5 6.0 5.5 5.0 4.5 4.0 3.5 3.0 2.5 2.0 1.5 1.0 0.5 0.0
chemical shift (ppm)

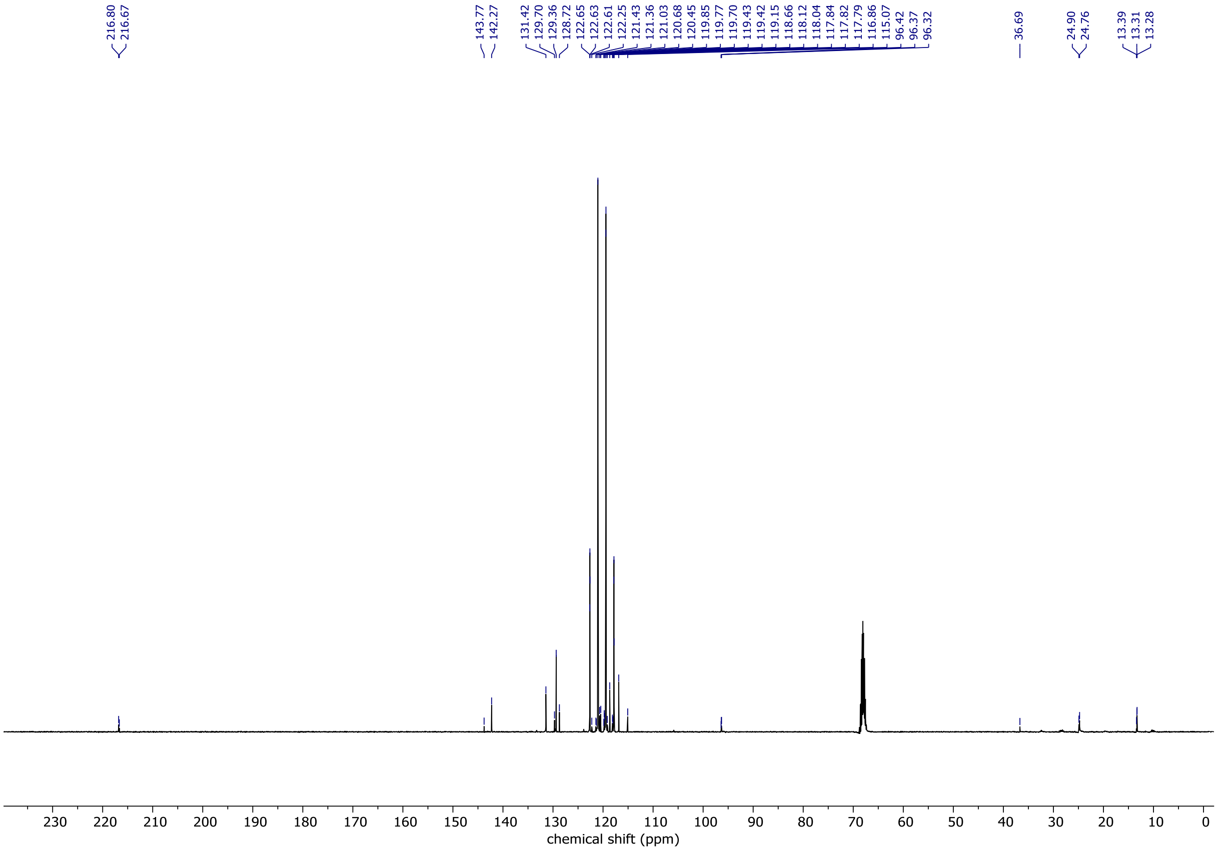
216.80 216.67
143.77 142.27
131.42 129.70 129.36 128.72 122.65 122.63 122.61 122.25 121.43 121.36 121.03 120.68 120.45 119.85 119.77 119.70 119.43 119.42 119.15 118.66 118.12 118.04 117.84 117.82 117.79 116.86 115.07 96.42 96.37 96.32
36.69
24.90 24.76
13.39 13.31 13.28
230 220 210 200 190 180 170 160 150 140 130 120 110 100 90 80 70 60 50 40 30 20 10 0
chemical shift (ppm)

4.2.3 NMR study with HFIP as additive

Seeking an explanation for the influence of HFIP in the quenching process, we performed an NMR study in DCM-d_2 and added only 3 equivalents of HFIP immediately after the cation sampling event (resembling our optimized reaction protocol). While the NMR data matched those observed previously (see Figure S8, in great accordance with the data shown in section 4.1), we also conducted a more in-depth analysis of the ^{19}F NMR data. This analysis further confirmed that HFIP does not form covalent oxocarbenium-HFIP adducts (*e.g.*, hemiacetal adducts) or any related species. The only detectable species were TfO^- and unaltered HFIP (Figure S9; a mixture of both was measured in-house for comparison).

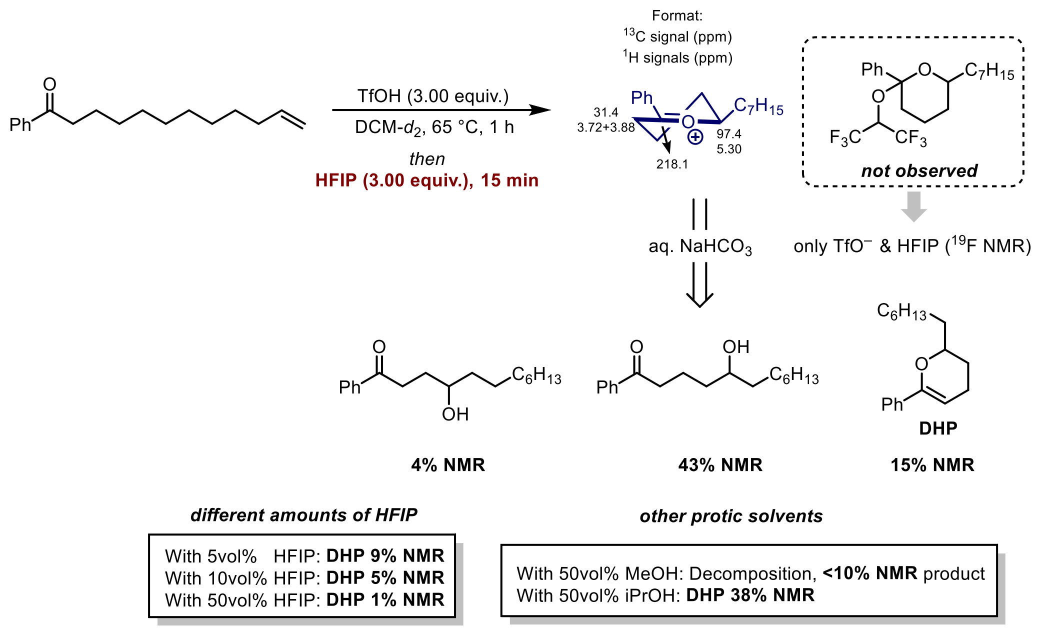

Figure S8. NMR study on the role of HFIP in the quenching process. No covalent HFIP adducts were detected. We found that HFIP produced substantial effects even at low quantities (5–10 vol%), and it could *not* be substituted by other protic additives.

Furthermore, quenching the crude material with a saturated aqueous $NaHCO_3$ solution demonstrated that even a much lower amount of HFIP produced a substantial effect compared to the same reaction performed without HFIP. However, the effect was less pronounced than when HFIP was used as a co-solvent prior to quenching. To exclude the possibility that the effects of HFIP are solely of a technical nature, such as simply altering the miscibility of the reaction solvent with aqueous $NaHCO_3$ solution, we attempted to substitute HFIP with MeOH or iPrOH. However, these substitutions resulted in either decomposition (MeOH) or the formation of a substantial amount of dihydropyran (iPrOH).

On this basis, we believe that the effect of HFIP could be to stabilize the cationic species during the quenching process in a way that disfavors undesired deprotonation and/or favors hydrolysis of the oxocarbenium species.[17]

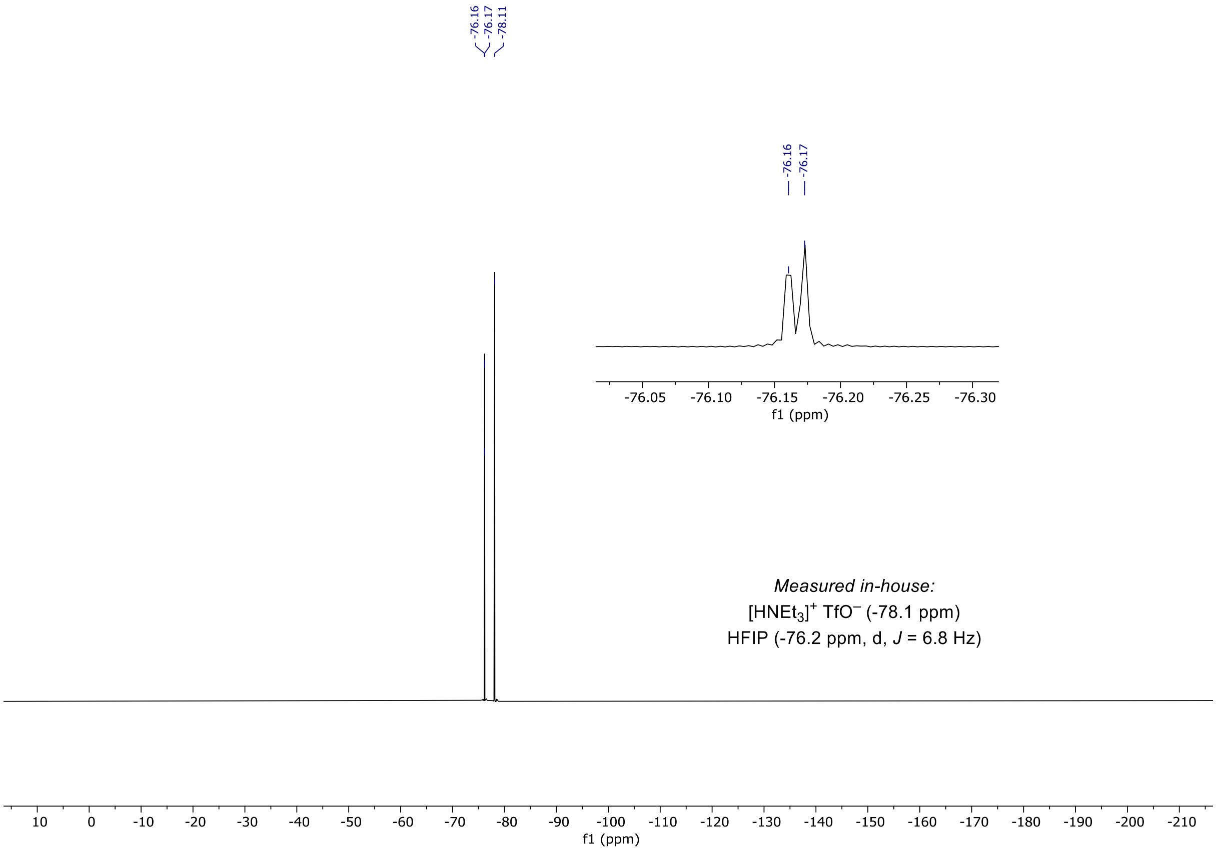

Figure S9. ^{19}F NMR of the reaction mixture after HFIP addition. The mixture was found to contain only unaltered HFIP and TfO^- as fluorine-bearing compounds. A mixture of both species (HFIP and triethylammonium triflate) was measured in-house to have an authentic sample for comparison.

4.3 Deuteration Study

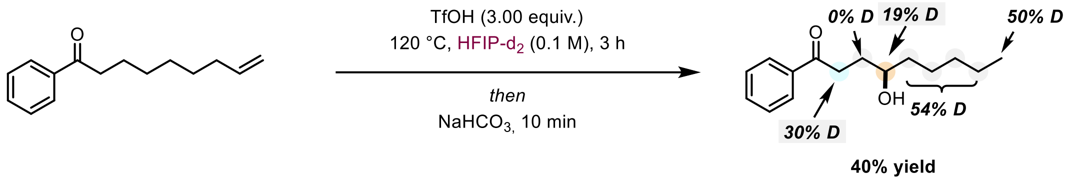

Procedure: 1-Phenylnon-8-en-1-one (21.6 mg, 0.10 mmol, 1.00 equiv.) was weighed into a vial (4 mL total volume, no inert atmosphere required) and dissolved in HFIP-d_2 (1 mL). TfOH (26.5 µL, 0.30 mmol, 3.00 equiv.) was added, the vial was closed with a screwcap and placed into a metal heating block (pre-heated to 120 °C, vial fully immersed, see Section 2.1.4) and the solution was stirred at 120 °C for 3 h. After this time, the vial was placed into a cold-water bath (10 °C) for 10 min and aqueous saturated $NaHCO_3$ solution (3 mL) was added. The mixture was transferred into a separatory funnel, the phases were separated, and the aqueous phase was extracted with DCM (2 x 5 mL). The combined organic phases were dried over anhydrous $MgSO_4$, the dried solution was filtered, and the filtrate was concentrated under reduced pressure. The crude residue was purified by flash column chromatography (0 – 60% EtOAc in heptanes) to yield the deuterated γ-hydroxy ketone **2b** (9.4 mg, 40 µmol, 40%) as yellowish oil with a high degree of deuteration (see attached NMR spectra for deuteration degree at each position and mass spectrum for additional evidence).

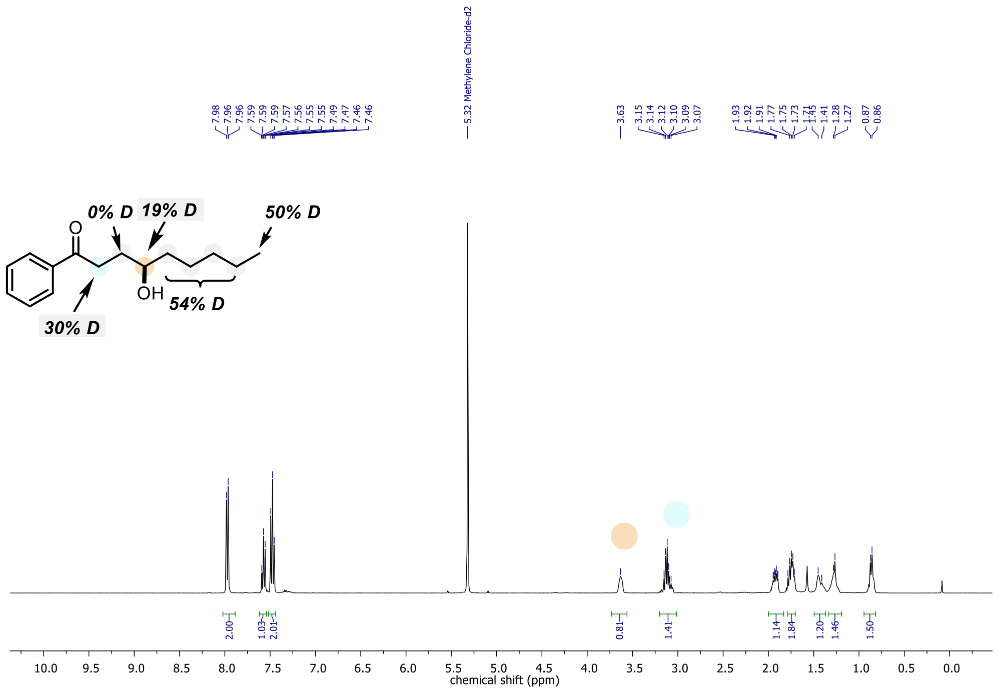

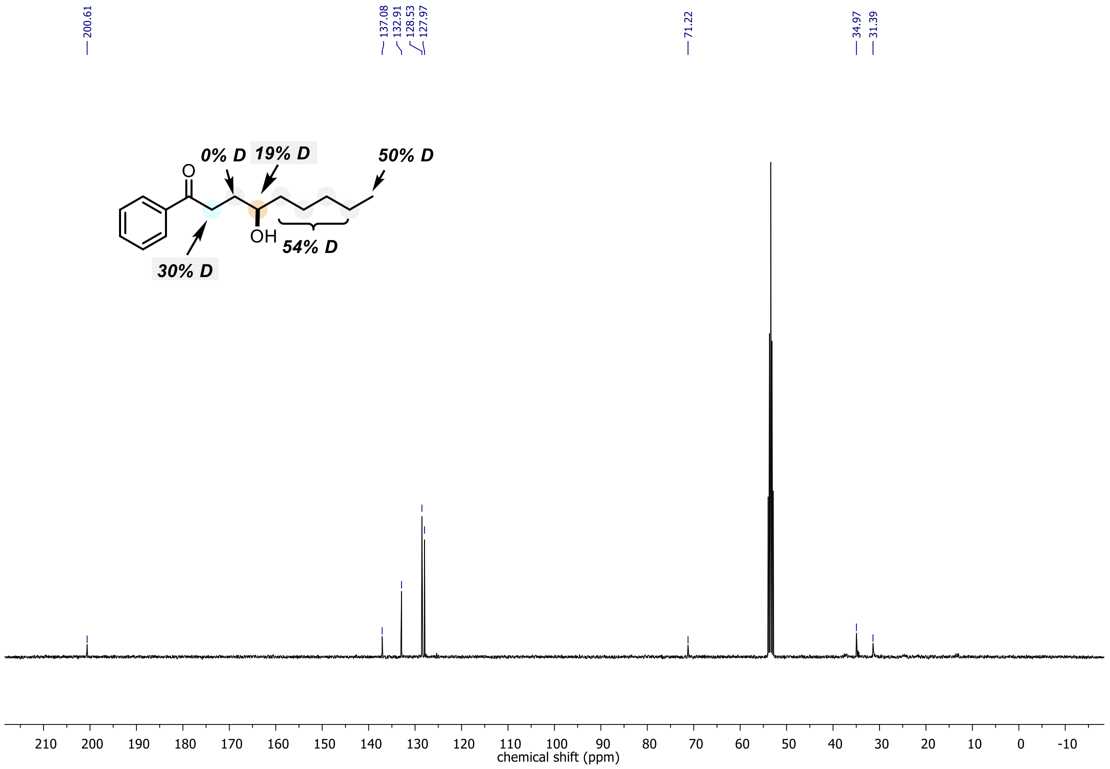
200.61
137.08
132.91
128.53
127.97
71.22
34.97
31.39
0% D
19% D
50% D
O
OH
30% D
54% D
210 200 190 180 170 160 150 140 130 120 110 100 90 80 70 60 50 40 30 20 10 0 -10
chemical shift (ppm)

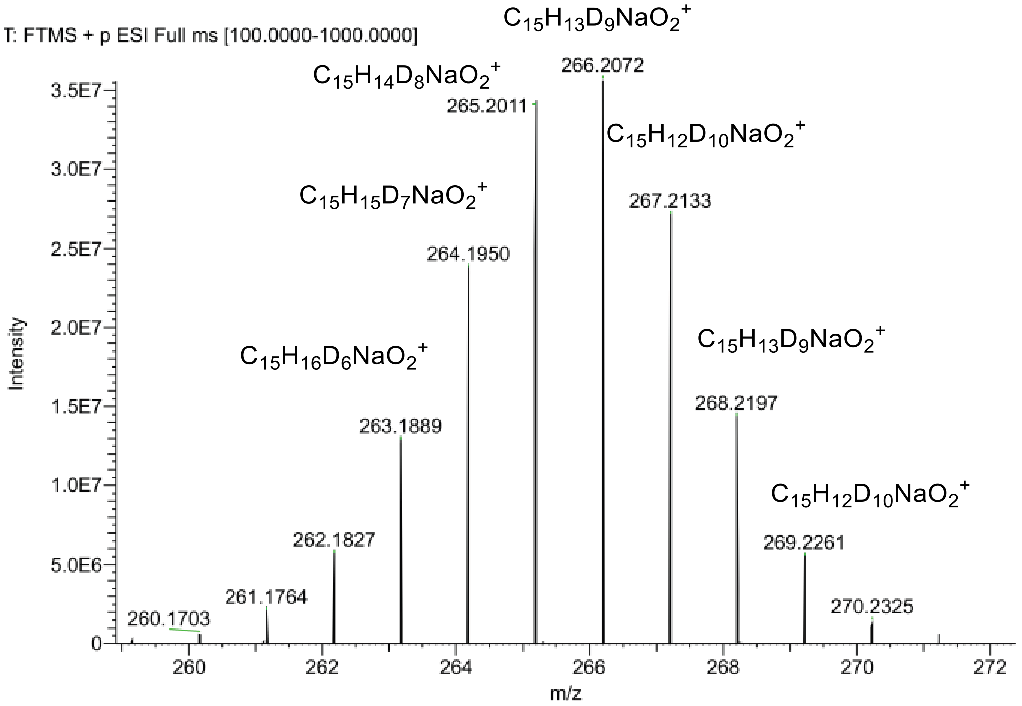
T: FTMS + p ESI Full ms [100.0000-1000.0000]
$C_{15}H_{13}D_9NaO_2^+$
266.2072
$C_{15}H_{14}D_8NaO_2^+$
265.2011
$C_{15}H_{12}D_{10}NaO_2^+$
$C_{15}H_{15}D_7NaO_2^+$
267.2133
264.1950
$C_{15}H_{16}D_6NaO_2^+$
$C_{15}H_{13}D_9NaO_2^+$
268.2197
263.1889
$C_{15}H_{12}D_{10}NaO_2^+$
262.1827
269.2261
261.1764
270.2325
260.1703
Intensity
m/z

4.4 Alternative groups for cation sampling

Motivated in part by previous literature reports employing strong mineral acids,[18,19] we sought to investigate the use of different functional groups to trap the positive charge. However, applying our cation sampling conditions to carboxylic acids or to the corresponding methyl esters led to excessive decomposition at >100 °C and to polymerization at <80 °C. Formation of estolides (and polyestolides) from unsaturated carboxylic acids under acid catalysis is a well-documented phenomenon in the literature.[20,21]

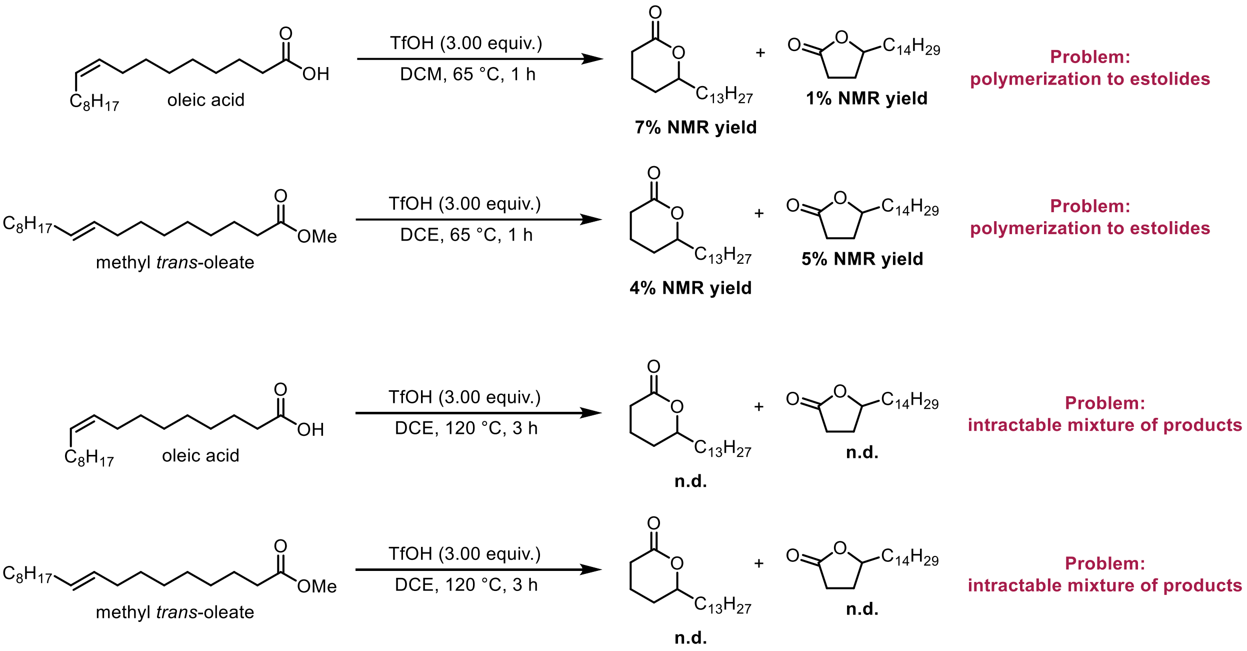

We also sought to reproduce the results reported in ref. 18 by subjecting oleic acid to sulfuric acid–mediated lactonization conditions (conditions #7, Table 1 from ref. 18). Under these conditions, δ-stearolactone was obtained in low yield and with a lower selectivity than reported.

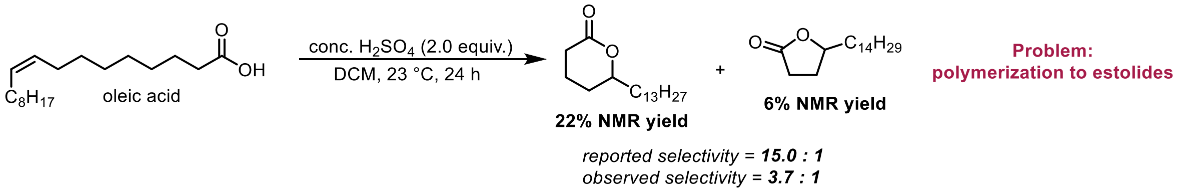

Although ref. 18 reports a column labeled “yield” in Table 1, we believe that these values correspond to product distribution ratios rather than isolated or calibrated yields. No calibration procedure or internal standard for the HPLC analysis is described, and the reported “yield” values consistently sum to 100.0%, further supporting the interpretation that they represent relative product ratios rather than absolute yields.

Additionally, we attempted to apply the conditions from General Procedures F and G to substrates bearing other functional groups, such as *O*-esters, primary and secondary amines, halides, and sulfoxides. In all cases, these conditions resulted in excessive decomposition, likely by cationic polymerization, as evidenced by the formation of insoluble material.

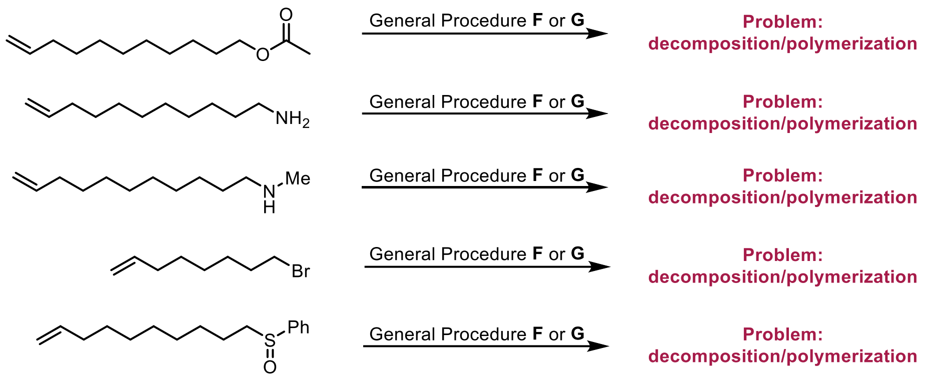

5. X-ray data

Compound 1h: CCDC2401182

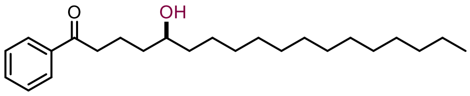

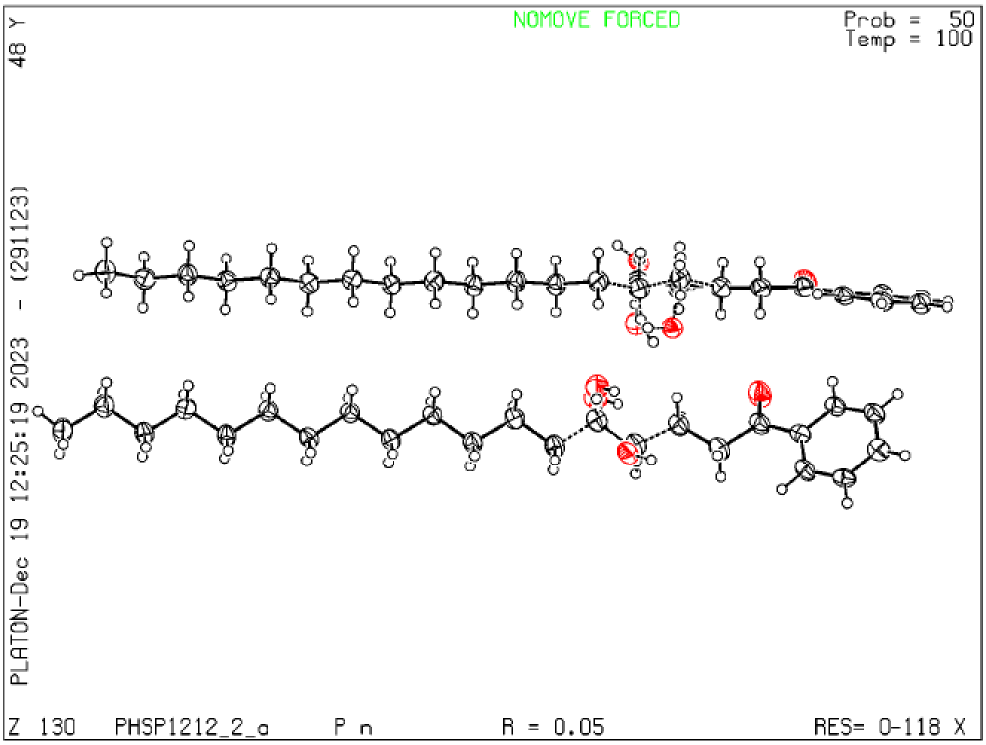

The crystal structure contains two independent molecules per asymmetric unit. Each molecule shows two conformations with the oxygen either at the C-gamma or the C-delta position. In addition, the oxygen at the C-delta position is visible with two populations, a major one and a minor one. The fractions of oxygen were determined from the crystallographic data, where the two molecules were treated completely independent from one another. Within each molecule, the total fraction of oxygen was restrained to 1.0, with the following percentages for the O-atoms:

<u>Molecule 1</u>

O-gamma:	0.1025	(esd=	0.0038)
O-delta(major):	0.7966	(esd=	0.0040)
O-delta(minor):	0.1009	(esd=	0.0037)

<u>Molecule 2</u>

O-gamma:	0.0831	(esd=	0.0037)
O-delta(major):	0.8175	(esd=	0.0041)
O-delta(minor):	0.0994	(esd=	0.0037)

From Molecule 1, the fraction of O-delta computes as 0.7966+0.1009 = 0.8975 (esd = 0.008).

From Molecule 2, the fraction of O-delta computes as 0.8175+0.0994 = 0.927 (esd = 0.0078).

The fractions determined from molecule 1 and molecule 2 are the same within the variation, which can be expected at the given data resolution and data quality.

The screenshot shows two molecules (upper: 1, lower: 2) with both configurations and the split population for O-delta (blue shade for O(major) and green shade of O(minor).

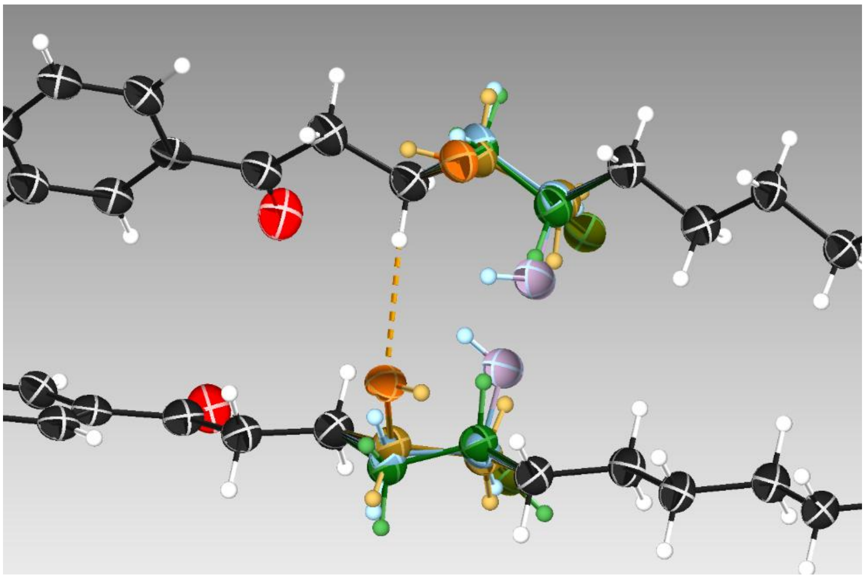

checkCIF/PLATON report

Structure factors have been supplied for datablock(s) PHSP1212_2_a

THIS REPORT IS FOR GUIDANCE ONLY. IF USED AS PART OF A REVIEW PROCEDURE FOR PUBLICATION, IT SHOULD NOT REPLACE THE EXPERTISE OF AN EXPERIENCED CRYSTALLOGRAPHIC REFEREE.

No syntax errors found. CIF dictionary Interpreting this report

Datablock: PHSP1212_2_a

```
Bond precision:   C-C = 0.0075 A           Wavelength=1.54178

Cell:              a=5.531(2)     b=7.499(6)          c=52.20(4)
                   alpha=90       beta=92.437(9)      gamma=90
Temperature:       100 K

                 Calculated                   Reported
Volume           2163(3)                      2163(2)
Space group      P n                          P n
Hall group       P -2yac                      P -2yac
Moiety formula   C24 H40 O2                   ?
Sum formula      C24 H40 O2                   C24 H40 O2
Mr               360.56                       360.56
Dx,g cm-3        1.107                        1.107
Z                4                            4
Mu (mm-1)        0.516                        0.516
F000             800.0                        800.0
F000'            802.04
h,k,lmax         4,6,46                       4,6,46
Nref             3156[ 1581]                  2403
Tmin,Tmax        0.896,0.983                  0.642,0.749
Tmin'            0.846

Correction method= # Reported T Limits: Tmin=0.642 Tmax=0.749
AbsCorr = EMPIRICAL

Data completeness= 1.52/0.76        Theta(max)= 43.154

R(reflections)= 0.0502( 2300)                         wR2(reflections)=
                                                      0.1277( 2403)
S = 1.027                  Npar= 585
```

```
The following ALERTS were generated. Each ALERT has the format
       test-name_ALERT_alert-type_alert-level.
Click on the hyperlinks for more details of the test.
```

Alert level A

```
THETM01_ALERT_3_A  The value of sine(theta_max)/wavelength is less than 0.550
            Calculated sin(theta_max)/wavelength =    0.4436
```

Author Response: Data were collected with a set of low resolution runs and some sets of high resolution runs. The high resolution runs could not be indexed, and peak picking failed, as though the crystal got damaged between the two sets. We could not process the high resolution data.

```
PLAT089_ALERT_3_A Poor Data / Parameter Ratio (Zmax < 18) .......        2.64 Note
```

Author Response: cf. Altert THETM01_PHSP1212_2_a - we could only process the low resolution runs to 1.13A

Alert level C

```
PLAT029_ALERT_3_C _diffrn_measured_fraction_theta_full value Low .      0.975 Why?
PLAT340_ALERT_3_C Low Bond Precision on  C-C Bonds ..............    0.00748 Ang.
PLAT911_ALERT_3_C Missing FCF Refl Between Thmin & STh/L=    0.444          39 Report
                 4  0  0,  -4  0  2,   4  0  2,  -4  0  4,   4  0  4,   3  0  5,
                 4  1  5,  -4  0  6,   4  0  6,   4  1  6,   4  1  7,  -4  0  8,
                 4  0  8,   4  1  8,   4  1  9,   4  0 10,   4  1 10,   4  1 11,
                 4  0 12,   4  1 12,   4  2 12,   4  2 13,   4  0 14,   4  2 14,
                 4  2 15,   4  3 15,   4  0 16,   4  0 18,  -4  0 20,   4  0 20,
                 4  2 21,  -4  0 22,   4  0 22,  -4  0 24,   4  0 24,   4  1 24,
                -4  0 26,   2  4 31,   2  2 39,
```

Alert level G

```
PLAT002_ALERT_2_G Number of Distance or Angle Restraints on AtSite         64 Note
PLAT003_ALERT_2_G Number of Uiso or Uij Restrained non-H Atoms ...         64 Report
PLAT007_ALERT_5_G Number of Unrefined Donor-H Atoms .............           6 Report
              H10#  H14#  H18#  H58#  H62#  H66#
PLAT072_ALERT_2_G  SHELXL  First  Parameter  in  WGHT  Unusually  Large     0.12  Report
PLAT172_ALERT_4_G  The  CIF-Embedded  .res  File  Contains  DFIX  Records     31  Report
PLAT173_ALERT_4_G  The  CIF-Embedded  .res  File  Contains  DANG  Records     35  Report
PLAT174_ALERT_4_G  The  CIF-Embedded  .res  File  Contains  FLAT  Records      4  Report
PLAT178_ALERT_4_G  The  CIF-Embedded  .res  File  Contains  SIMU  Records      1  Report
PLAT187_ALERT_4_G  The  CIF-Embedded  .res  File  Contains  RIGU  Records      1  Report
PLAT188_ALERT_3_G A Non-default SIMU Restraint Value has been used     0.0100 Report
PLAT301_ALERT_3_G Main Residue  Disorder .............. (Resd  1  )        12% Note
PLAT301_ALERT_3_G Main Residue  Disorder .............. (Resd  2  )        12% Note
PLAT367_ALERT_2_G Long?  C(sp?)-C(sp?)  Bond  C8_1     - C9_1     .       1.52 Ang.
PLAT367_ALERT_2_G Long?  C(sp?)-C(sp?)  Bond  C8_2     - C9_2     .       1.52 Ang.
PLAT367_ALERT_2_G Long?  C(sp?)-C(sp?)  Bond  C12_2    - C13_2    .       1.52 Ang.
PLAT414_ALERT_2_G Short Intra D-H..H-X       H9A_1^A  ..H2A_1^C   .       2.11 Ang.
```

```
                                                    x,y,z  =      1_555 Check
PLAT414_ALERT_2_G Short Intra D-H..H-X       H12B_2^A ..H2A_2^B  .       1.73 Ang.
                                                    x,y,z  =      1_555 Check
PLAT414_ALERT_2_G Short Intra D-H..H-X       H13B_2   ..H2A_2^C  .       2.03 Ang.
                                                    x,y,z  =      1_555 Check
PLAT415_ALERT_2_G Short Inter D-H..H-X       H9B_1^A  ..H2A_2^A  .       2.13 Ang.
                                                    x,y,z  =      1_555 Check
PLAT415_ALERT_2_G Short Inter D-H..H-X       H12A_1^A ..H2A_1^C  .       1.88 Ang.
                                                  1+x,y,z  =      1_655 Check
PLAT415_ALERT_2_G Short Inter D-H..H-X       H9A_2^A  ..H2A_2^B  .       1.87 Ang.
                                                 -1+x,y,z  =      1_455 Check
PLAT415_ALERT_2_G Short Inter D-H..H-X       H14A_2   ..H2A_2^C  .       1.88 Ang.
                                                  1+x,y,z  =      1_655 Check
PLAT480_ALERT_4_G Long H...A H-Bond Reported H8B_2    ..O1_2     .       2.64 Ang.
PLAT480_ALERT_4_G Long H...A H-Bond Reported H12B_2^A ..O2#      .       2.63 Ang.
PLAT720_ALERT_4_G Number of Unusual/Non-Standard Labels ..........        160 Note
              O1_1    C1_1    H1_1    C2_1    H2_1    C3_1    H3_1    C4_1
              H4_1    C5_1    H5_1    C6_1    C7_1    C8_1    H8A_1   H8B_1
              C9_1    H9A_1^A H9B_1^A O2_1^A  H2A_1^A C10_1^A H10A_1^AH10B_1^A
              C11_1^A H11_1^A O2'_1^B H2A'_1^BC10'_1^BH10A_1^BC11'_1^BH11A_1^B
              H11B_1^BO2_1^C  H2A_1^C C10_1^C H10A_1^CH10B_1^CC11_1^C H11_1^C
              C12_1   H12A_1^AH12B_1^AC13_1   H13A_1  H13B_1  C14_1   H14A_1
              H14B_1  C15_1   H15A_1  H15B_1  C16_1   H16A_1  H16B_1  C17_1
              H17A_1  H17B_1  C18_1   H18A_1  H18B_1  C19_1   H19A_1  H19B_1
              C20_1   H20A_1  H20B_1  C21_1   H21A_1  H21B_1  C22_1   H22A_1
              H22B_1  C23_1   H23A_1  H23B_1  C24_1   H24A_1  H24B_1  H24C_1
              O1_2    C1_2    H1_2    C2_2    H2_2    C3_2    H3_2    C4_2
              H4_2    C5_2    H5_2    C6_2    C7_2    C8_2    H8A_2   H8B_2
              C9_2    H9A_2^A H9B_2^A O2_2^A  H2A_2^A C10_2^A H10A_2^AH10B_2^A
              C11_2^A H11_2^A O2'_2^B H2A_2^B C10'_2^BH10_2^B C11'_2^BH11A_2^B
              H11B_2^BO2_2^C  H2A_2^C C10_2^C H10A_2^CH10B_2^CC11_2^C H11_2^C
              C12_2   H12A_2^AH12B_2^AC13_2   H13A_2  H13B_2  C14_2   H14A_2
              H14B_2  C15_2   H15A_2  H15B_2  C16_2   H16A_2  H16B_2  C17_2
              H17A_2  H17B_2  C18_2   H18A_2  H18B_2  C19_2   H19A_2  H19B_2
              C20_2   H20A_2  H20B_2  C21_2   H21A_2  H21B_2  C22_2   H22A_2
              H22B_2  C23_2   H23A_2  H23B_2  C24_2   H24A_2  H24B_2  H24C_2
PLAT860_ALERT_3_G Number of Least-Squares Restraints ............     2040 Note
PLAT909_ALERT_3_G Percentage of I>2sig(I) Data at Theta(Max) Still     89% Note
PLAT910_ALERT_3_G Missing # of FCF Reflection(s) Below Theta(Min).        1 Note
                0  0  2,
PLAT915_ALERT_3_G No Flack x Check Done: Low Friedel Pair Coverage      55 %
PLAT978_ALERT_2_G Number C-C Bonds with Positive Residual Density.        1 Info
```

---

  2 **ALERT level A** = Most likely a serious problem - resolve or explain
  0 **ALERT level B** = A potentially serious problem, consider carefully
  3 **ALERT level C** = Check. Ensure it is not caused by an omission or oversight
 30 **ALERT level G** = General information/check it is not something unexpected

  0 ALERT type 1 CIF construction/syntax error, inconsistent or missing data
 14 ALERT type 2 Indicator that the structure model may be wrong or deficient
 12 ALERT type 3 Indicator that the structure quality may be low
  8 ALERT type 4 Improvement, methodology, query or suggestion
  1 ALERT type 5 Informative message, check

---

6. References

(1) Fulmer, G. R.; Miller, A. J. M.; Sherden, N. H.; Gottlieb, H. E.; Nudelman, A.; Stoltz, B. M.; Bercaw, J. E.; Goldberg, K. I. NMR Chemical Shifts of Trace Impurities: Common Laboratory Solvents, Organics, and Gases in Deuterated Solvents Relevant to the Organometallic Chemist. *Organometallics* **2010**, *29* (9), 2176–2179.

(2) Love, B. E.; Jones, E. G. The Use of Salicylaldehyde Phenylhydrazone as an Indicator for the Titration of Organometallic Reagents. *J. Org. Chem.* **1999**, *64* (10), 3755–3756.

(3) Knochel, P.; Krasovskiy, A. Convenient Titration Method for Organometallic Zinc, Magnesium, and Lanthanide-Reagents. *Synthesis* **2006**, *2006* (05), 0890–0891.

(4) Volpe, R.; Aurelio, L.; Gillin, M. G.; Krenske, E. H.; Flynn, B. L. Mapping the Interactions of I2 , I(.) , I(-) , and I(+) with Alkynes and Their Roles in Iodocyclizations. *Chem. Eur. J.* **2015**, *21* (28), 10191–10199.

(5) Zhu, C.; Sun, M.-M.; Chen, K.; Liu, H.; Feng, C. Selective C-F Bond Allylation of Trifluoromethylalkenes. *Angew. Chem. Int. Ed.* **2021**, *60* (37), 20237–20242.

(6) Huang, F.; Zhang, S. Iminyl Radicals by Reductive Cleavage of N-O Bond in Oxime Ether Promoted by SmI2: A Straightforward Synthesis of Five-Membered Cyclic Imines. *Org. Lett.* **2019**, *21* (18), 7430–7434.

(7) Roslin, S.; Odell, L. R. Palladium and Visible-Light Mediated Carbonylative Suzuki-Miyaura Coupling of Unactivated Alkyl Halides and Aryl Boronic Acids. *Chem. Commun.* **2017**, *53* (51), 6895–6898.

(8) Denard, C. A.; Huang, H.; Bartlett, M. J.; Lu, L.; Tan, Y.; Zhao, H.; Hartwig, J. F. Cooperative Tandem Catalysis by an Organometallic Complex and a Metalloenzyme. *Angew. Chem. Int. Ed.* **2014**, *53* (2), 465–469.

(9) Parsons, A. T.; Buchwald, S. L. Copper-Catalyzed Trifluoromethylation of Unactivated Olefins. *Angew. Chem. Int. Ed.* **2011**, *50* (39), 9120–9123.

(10) Tsukahara, M.; Uyanik, M.; Ishihara, K. Hypoiodite-catalyzed Oxidative A-C–N Coupling of Ketones with Imides and Azoles. *Adv. Synth. Catal.* **2023**, *365* (16), 2724–2729.

(11) He, Y.; Cai, Y.; Zhu, S. Mild and Regioselective Benzylic C-H Functionalization: Ni-Catalyzed Reductive Arylation of Remote and Proximal Olefins. *J. Am. Chem. Soc.* **2017**, *139* (3), 1061–1064.

(12) Cosner, C. C.; Cabrera, P. J.; Byrd, K. M.; Thomas, A. M. A.; Helquist, P. Selective Oxidation of Benzylic and Allylic Alcohols Using Mn(OAc)3/Catalytic 2,3-Dichloro-5,6-Dicyano-1,4-Benzoquinone. *Org. Lett.* **2011**, *13* (8), 2071–2073.

(13) Xu, X.; Yang, C.; Li, S.; Meng, C.; Yu, J.; Yang, J.; Li, F. From Selective Transfer Hydrogenation to Selective Hydrogen Auto-Transfer Process: An Efficient Method for the Synthesis of Alkenyl Ketones via Iridium-Catalyzed α-Alkylation of Ketones with Alkenyl Alcohols. *J. Catal.* **2021**, *402*, 335–343.

(14) Brutiu, B. R.; Iannelli, G.; Riomet, M.; Kaiser, D.; Maulide, N. Stereodivergent 1,3-Difunctionalization of Alkenes by Charge Relocation. *Nature* **2024**, *626* (7997), 92–97.

(15) Koo, S. M.; Vendola, A. J.; Momm, S. N.; Morken, J. P. Alkyl Group Migration in Ni-Catalyzed Conjunctive Coupling with C(Sp3) Electrophiles: Reaction Development and Application to Targets of Interest. *Org. Lett.* **2020**, *22* (2), 666–669.

(16) Grant, P. S.; Vavrík, M.; Porte, V.; Meyrelles, R.; Maulide, N. Remote Proton Elimination: C–H Activation Enabled by Distal Acidification. *Science* **2024**, *384* (6697), 815–820.

(17) Pozhydaiev, V.; Power, M.; Gandon, V.; Moran, J.; Lebœuf, D. Exploiting Hexafluoroisopropanol (HFIP) in Lewis and Brønsted Acid-Catalyzed Reactions. *Chem. Commun.* **2020**, *56* (78), 11548–11564.

(18) Cermak, S. C.; Isbell, T. A. Synthesis of δ-Stearolactone from Oleic Acid. *J. Am. Oil Chem. Soc.* **2000**, *77* (3), 243–248.

(19) Shepherd, I. S.; Showell, J. S. The Mechanism of the Aqueous Perchloric Acid Isomerization of Oleic Acid to γ-Stearolactone. *J. Am. Oil Chem. Soc.* **1969**, *46* (9), 479–481.

(20) Isbell, T. A.; Frykman, H. B.; Abbott, T. P.; Lohr, J. É.; Drozd, J. C. Optimization of the Sulfuric Acid-Catalyzed Estolide Synthesis from Oleic Acid. *J. Am. Oil Chem. Soc.* **1997**, *74* (4), 473–476.

(21) Isbell, T. A.; Kleiman, R.; Plattner, B. A. Acid-catalyzed Condensation of Oleic Acid into Estolides and Polyestolides. *J. Am. Oil Chem. Soc.* **1994**, *71* (2), 169–174.
